# The asymmetric cell division machinery in the spiral-cleaving egg and embryo of the marine annelid *Platynereis dumerilii*

**DOI:** 10.1186/s12861-017-0158-9

**Published:** 2017-12-11

**Authors:** Aron B. Nakama, Hsien-Chao Chou, Stephan Q. Schneider

**Affiliations:** 1 Department of Genetics, Development and Cell Biology, Iowa State University, 503 Science Hall II, Ames, IA 50011 USA; 2 current address: Center for Cancer Research, National Institutes of Health, Bethesda, MD 20894 USA

**Keywords:** Maternal contribution, Spiral cleavage, Cortical domains, PAR genes, Spindle orientation, Cell polarity, Crumbs complex, Planar cell polarity, Neural adhesion complexes, Transcriptional profiles

## Abstract

**Background:**

Over one third of all animal phyla utilize a mode of early embryogenesis called ‘spiral cleavage’ to divide the fertilized egg into embryonic cells with different cell fates. This mode is characterized by a series of invariant, stereotypic, asymmetric cell divisions (ACDs) that generates cells of different size and defined position within the early embryo. Astonishingly, very little is known about the underlying molecular machinery to orchestrate these ACDs in spiral-cleaving embryos. Here we identify, for the first time, cohorts of factors that may contribute to early embryonic ACDs in a spiralian embryo.

**Results:**

To do so we analyzed stage-specific transcriptome data in eggs and early embryos of the spiralian annelid *Platynereis dumerilii* for the expression of over 50 candidate genes that are involved in (1) establishing cortical domains such as the partitioning defective (*par*) genes, (2) directing spindle orientation, (3) conveying polarity cues including *crumbs* and *scribble*, and (4) maintaining cell-cell adhesion between embryonic cells. In general, each of these cohorts of genes are co-expressed exhibiting high levels of transcripts in the oocyte and fertilized single-celled embryo, with progressively lower levels at later stages. Interestingly, a small number of key factors within each ACD module show different expression profiles with increased early zygotic expression suggesting distinct regulatory functions. In addition, our analysis discovered several highly co-expressed genes that have been associated with specialized neural cell-cell recognition functions in the nervous system. The high maternal contribution of these ‘neural’ adhesion complexes indicates novel general adhesion functions during early embryogenesis.

**Conclusions:**

Spiralian embryos are champions of ACD generating embryonic cells of different size with astonishing accuracy. Our results suggest that the molecular machinery for ACD is already stored as maternal transcripts in the oocyte. Thus, the spiralian egg can be viewed as a totipotent yet highly specialized cell that evolved to execute fast and precise ACDs during spiral cleaving stages. Our survey identifies cohorts of factors in *P. dumerilii* that are candidates for these molecular mechanisms and their regulation, and sets the stage for a functional dissection of ACD in a spiral-cleaving embryo.

**Electronic supplementary material:**

The online version of this article (10.1186/s12861-017-0158-9) contains supplementary material, which is available to authorized users.

## Background

Asymmetric cell division (ACD) is the fundamental process that subdivides a mother cell into two daughter cells that exhibit differences in cell fate, cell size, and/or cell position. As such, it constitutes a central developmental mechanism essential for cell lineage diversification during early embryogenesis, formation of organs, and division of stem cells (reviewed in [1–4]). Much of our current knowledge about ACD has been established over the last two decades exploiting two genetic invertebrate model organisms, the nematode worm *Caenorhabditis elegans (C. elegans)* and the arthropod fruit fly *Drosophila melanogaster (Drosophila)*. In both systems, a detailed understanding of embryonic and postembryonic processes on a cellular level, and the use of forward and reverse genetic tools, has led to the identification of mostly conserved cohorts of genes. These genes execute ACDs in various developmental contexts including the formation of diversified cell lineages within embryos, the nervous system, sensory organs, wing development, and epithelia.

The significance of ACD becomes unmistakable when considering the earliest steps during metazoan embryogenesis. Mitotic cell divisions subdivide the fertilized egg into embryonic cells with different fates and defined positions that foreshadow the three emerging body axes of the embryo. This process has been studied on the molecular level extensively, in *C. elegans,* where all three body axes anterior/posterior (A/P), dorsal/ventral (D/V), and right/left (R/L) are determined by the third round of embryonic cell divisions [2, 5]. Complementary studies in *Drosophila*, and in various vertebrate models including frog and mouse embryos, have identified similarities on the molecular level [4, 6, 7]. However, comparisons are complicated by the derived nature of the molecular machinery for ACD in *C. elegans* and/or the divergence of early developmental processes between metazoan models. Although studies on ACD in various new models are emerging [8, 9], there are still large gaps in our understanding of the nature, utilization, conservation, and evolution of the ACD mechanisms; especially in regards to establishing cellular asymmetries and cell lineages during metazoan embryogenesis.

Here we introduce the marine annelid *Platynereis dumerilii* as a model to study ACD in a spiral-cleaving embryo [10]. Spiral cleavage is a widespread developmental mode among approximately one-third of all animal phyla including mollusks (snails and clams), and annelids (leeches and earthworms) [11]. This mode is characterized by a series of asymmetric cell divisions that subdivide the fertilized egg into embryonic cells of different size and fates with an invariant, stereotypic spiral arrangement. Indeed, early observations of spiral cleaving embryos and their cell size asymmetries were instrumental for the development of early cellular theories on egg-to-embryo development nearly 140 years ago [12]. Although cellular aspects of ACD, including the role of the cytoskeleton, have been studied in annelid embryos by the Shimizu and Weisblat laboratories [13–17], surprisingly little is known about the molecular machinery that executes ACD in spiralians, even though these embryos exhibit some of the most readily observable and extreme cell size asymmetries in all metazoans.

Developing *P. dumerilii* embryos produce the typical hallmarks of spiral cleavage. Every cell division, beginning with the one-celled zygote, is asymmetric. ACD continues through early cleavage stages and beyond generating daughter cells of different sizes [18, 19]. For example, the first mitotic division always generates the smaller AB and the larger CD blastomeres [10, 19, 20]. This first division defines the D/V axis. As in other spiral cleaving embryos, the developmental program in *P. dumerilii* is recognizable through the pattern of cell divisions based upon the stereotypic orientation of the mitotic spindle. Starting with the 3rd cell division the alternating mitotic spindles are oriented either clockwise or counter clockwise, with respect to the animal pole, generating animal-pole and vegetal-pole sister cell pairs [10, 18–20]. Thus, the embryonic cells of differing size and position are assembled in a stereotypic and invariant pattern in each stage.

The generation of the spiral cleavage pattern along the animal-vegetal axis requires an asymmetric cue, cellular polarization, and/or some initial symmetry-breaking event in oocyte or zygote that leads to subsequent ACDs. In some organisms, the unfertilized egg/oocyte is polarized as observed in the intrinsically well-defined polarity axes within the *Drosophila* egg [21]. In other organisms, early asymmetric cues are introduced through fertilization e.g. the sperm entry point defines the future A/P axis in *C. elegans* [22, 23], and the D/V axis in *Xenopus laevis* [24]*.* Currently, it is not known if internal polarity of the oocyte exists in *P. dumerilii*. However, dramatic reorganization of the egg upon sperm contact, termed ooplasmic segregation, has been described [18, 19].

**Fig. 1:**
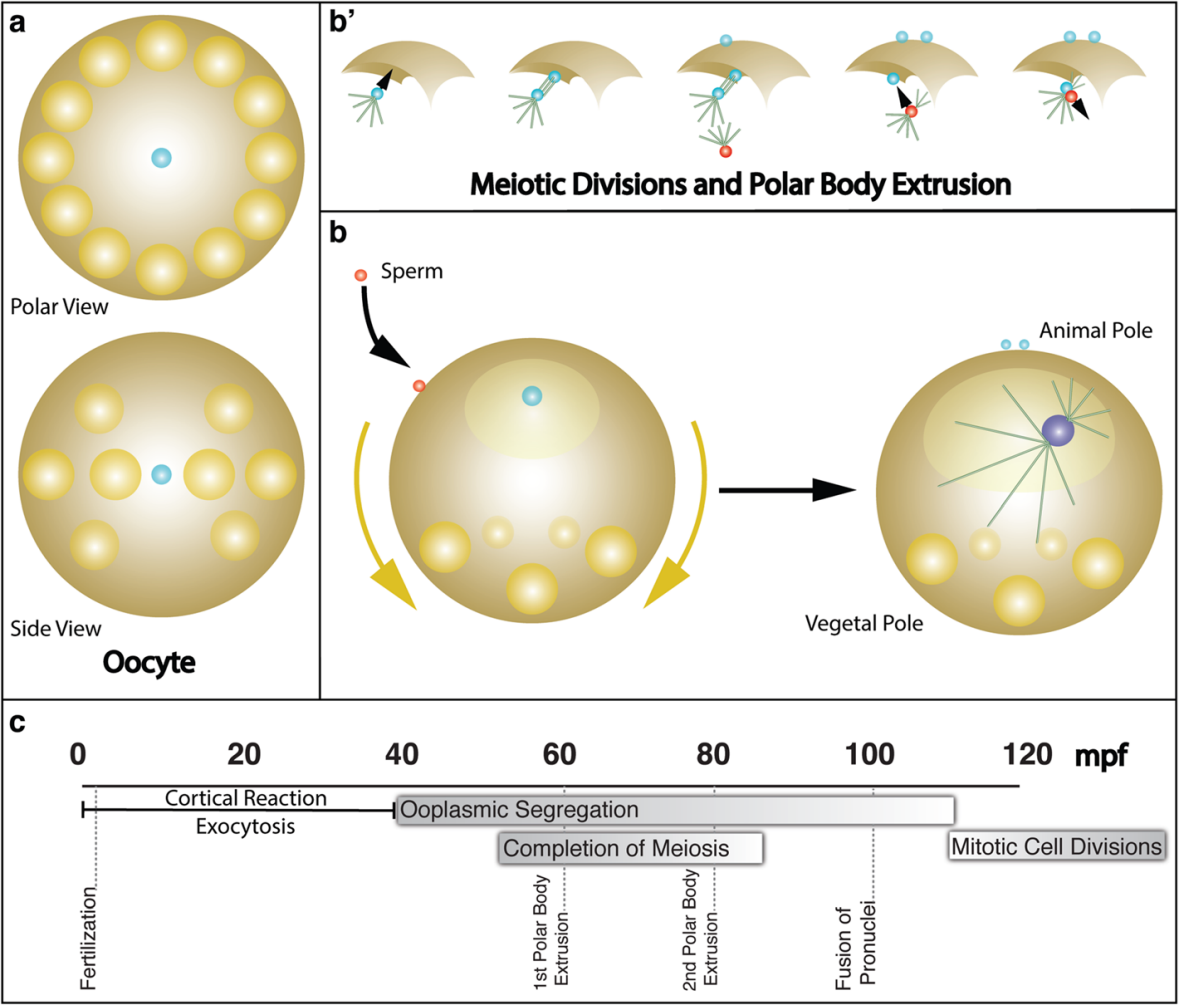
Polarization of the *P. dumerilii* Zygote upon Fertilization. **a** Mature Oocytes of *P. dumerilii*: Peripheral lipid droplets (yellow) surround the central oocyte nucleus (light blue) exhibiting a slight concentration within the equatorial plane. **b** Ooplasmic segregation triggered by fertilization. Cortical lipid droplets and yolk granules stream towards the vegetal pole while clear cytoplasm, including the female pronucleus, is segregated towards the animal pole establishing an A/V polarity axis. **b‛** Completion of the first and second meiotic cell division generates two polar bodies defining the animal pole. Male (red) and female (light blue) pronuclei fuse forming the nucleus of the zygote. **b** Asymmetry of the first mitotic spindle: The smaller aster of one spindle pole maintains its proximity to an animal cortical domain foreshadowing the first asymmetric mitotic cell division. **c** Timeline of early events that polarize the zygote upon fertilization in *P. dumerilii*. Mass exocytosis of cortical granules generating a surrounding jelly coat occupies the first 40 min post fertilization (mpf). Ooplasmic segregation leads to a redistribution of cytoplasm along the A/V axis, completion of meiotic division at the animal pole with the formation of the first and second polar body at ~60 and ~80 mpf, and subsequent fusion of pronuclei. Asymmetries in spindle pole sizes and unipolar spindle attachment to the animal cortex forecast the first asymmetric cell division parallel to the A/V axis generating a smaller AB cell and a larger CD cell (see Fig. 2)

After sperm contacts the egg, cortical streaming pushes yolk granules and lipid droplets towards the future vegetal pole. At the same time, an area of clear cytoplasm forms at the future animal pole, harboring the oocyte nucleus. About one-hour post fertilization (hpf), the oocyte nucleus completes meiotic cell divisions, via two extreme ACDs, that lead to the extrusion of two polar bodies. The site of polar body extrusion defines the animal pole and determines the animal/vegetal (A/V) axis [10, 19] (Fig. 1). Subsequently, the male pronucleus migrates towards the female pronucleus (now located at the animal pole). Pronuclear fusion and duplication of the male centrioles mark the beginning of the first mitotic cell division of the zygote [25]. The mitotic spindle pole located nearest the animal pole (nearest to the polar bodies) maintains its proximity to the animal pole membrane as the nucleus migrates vegetally [25]. This creates an asymmetric mitotic spindle arrangement leading to the cell size asymmetry generated during the first cell division. Therefore, the capture of the mitotic spindle pole that remains near the cortex, through association with a distinct animal pole associated cortical domain, may be causative for the subsequent first zygotic ACD.

**Fig. 2:**
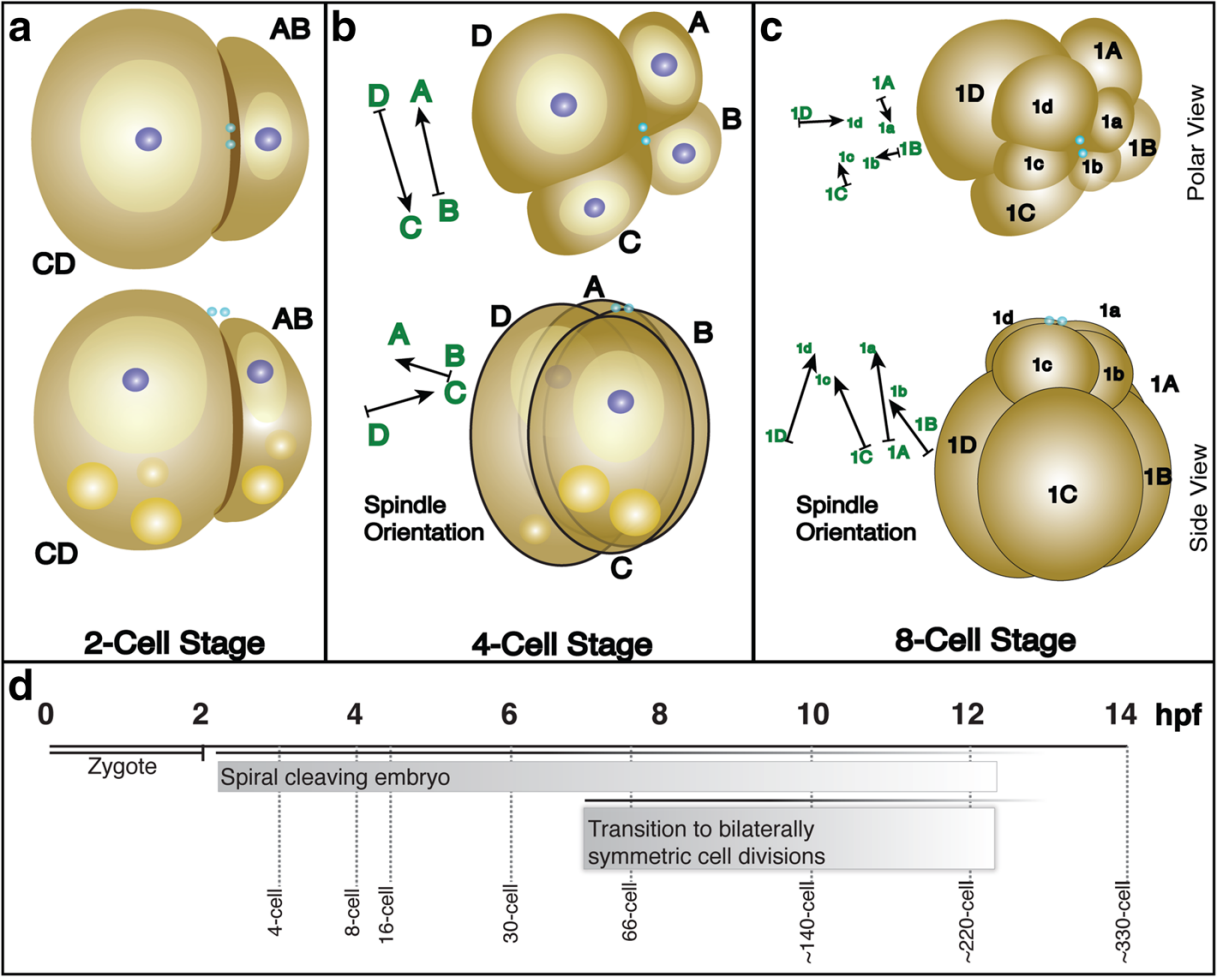
Early embryonic development of the marine annelid *P. dumerilii*. **a** 2-cell stage: The first mitotic ACD generates the larger CD and smaller AB blastomeres (CD is dorsal side, AB is ventral). **b** 4-cell stage: The second round of mitotic ACDs produces the four founder cells: A, B, C, and D. The D blastomere, the largest, contains the largest quantity of clear cytoplasm. **c** 8-cell stage: The third set of ACDs produces the smaller animal-pole sister cells, micromeres 1a, 1b, 1c, 1d, and the larger vegetal-pole sister cells, macromeres 1A, 1B, 1C, and 1D. Polar view of the spindle orientations (arrows) shows the counterclockwise offset of the micromeres from the macromeres generating the spiral arrangement of cells by the spiral cleavage. Upper row in (**a**), (**b**), and (**c**) shows view from animal pole, and lower row shows side views. Arrows in (**b**) and (**c**) indicate displacement angles of mitotic spindles with respect to viewing direction. The arrowheads point to the sister cell located closer to the animal pole. Arrow bases indicate the sister cell located closer to the vegetal pole. Polar bodies (teal spheres) mark the animal pole. Lipid droplets (yellow spheres) are localized at vegetal pole. Nuclei (purple) are located within the clear cytoplasm (lighter yellow) closer to the animal pole, surrounded by yolk-rich cytoplasm (light brown). **d** Developmental timeline of *P. dumerilii* from zygote to the ~330-cell stage (modified from Pruitt et al. 2014). The first mitotic cell division occurs shortly after 2 hpf, and subsequent stereotypic ACDs generate the spiral arrangement of cells during the next 5 h. After 7 hpf, cell divisions become more bilaterally symmetric in *P. dumerilii* embryos. Dashed lines indicate approximate age of various cell stages. Developmental expression profiles for each gene were generated by RNA-seq collected from embryos at seven time points (2, 4, 6, 8, 10, 12, and 14 hpf)

The first ACD, occurring in a plane parallel to the A/V axis, generates the larger CD blastomere (71% of the egg volume) and the smaller AB blastomere (29% of the egg volume) [19] establishing the secondary D/V axis with CD demarcating dorsal and AB ventral (Fig. 2a). The second round of cell divisions, generates four founder cells (A, B, C, D). The AB blastomere cleaves almost equally, whereas CD cleaves asymmetrically with the D blastomere retaining the largest volume [19]. Although the cleavage planes are parallel to the A/V axis, the mitotic spindle poles in A and C are slightly closer to the animal pole, producing slightly tilted mitotic spindles (Fig. 2b) [18, 19]. Therefore, this cleavage has been suggested as ‘spiral’ [18, 19]. The third round of cell divisions in A, B, C and D occurs perpendicular to the A/V axis, generating four sister cell pairs (8 cells), with large cell size asymmetries, producing the smaller animal-pole micromeres 1a, 1b, 1c, and 1d, and the much larger vegetal-pole macromeres 1A, 1B, 1C, and 1D, respectively (Fig. 2c) [18, 19]. Viewing from the animal pole, all four mitotic spindles are rotated dextrally, which offsets the micromeres from the macromeres in a clockwise manner [18]. The mitotic spindle orientations are flipped stereotypically in subsequent cell divisions [18]. During the fourth cell division, the four micromere mitotic spindles assume a counter clockwise orientation, generating four daughter cell pairs with precisely defined asymmetric cell sizes, and with the animal-pole daughter cells assuming sinistral positions relative to the four vegetal-pole daughter cells. Similarly, the four macromeres at the vegetal pole orient their mitotic spindles dextrally when viewed from the animal pole, producing a counterclockwise rotation of the most vegetal-pole daughters of the macromeres (clockwise for the more animal pole daughters of the macromeres) [18, 19]. ‘Spiral’ cleavages continue through the fifth and sixth cell division after which embryonic cells transition towards producing more bilaterally symmetric spindle orientations [19, 26]. However, stereotypic and invariant ACDs continue throughout early embryogenesis in *P. dumerilii*, continuing to generate cell size asymmetries.

Thus, the observed early symmetry breaking events including the meiotic divisions and the precise execution of ACDs during the spiral cleavage program implicate the activities of molecular machinery that (1) generates asymmetric spatial cues to establish distinct cortical domains, (2) direct meiotic and mitotic spindle orientation, (3) act upon various polarity cues to determine cell size, and (4) maintain cellular polarities via diverse cell-cell adhesion systems with early embryonic cells. As mentioned above, previous investigations in other model systems have identified strong candidates for each of these four ACD machineries. These include the well characterized Partitioning defective (Par) polarity network proteins, Par3 and Lethal (2) giant larvae (L(2)gl) that function both in establishing polarity in one-celled zygotes and mediate polarity events in other polarized tissue environments such as epithelial tissues (reviewed in [2, 4, 27]). Cellular asymmetries are also generated through mitotic spindle orientation proteins, including Nuclear Mitotic Apparatus (NuMA), Inscuteable (Insc), and Partner of Inscuteable (Pins) that asymmetrically orient the mitotic spindle along an existing polarity axis (reviewed in [28–31]). Molecular machinery, well characterized in epithelial tissues, includes the polarity complex proteins Crumbs and Scribble, which maintain subcellular domains (reviewed in [32–36]). Finally, cell adhesion proteins such as Van Gogh Like (Vangl), the adhesion G-protein coupled receptor (GPCR) Flamingo (Celsr1), Fat, and Dachsous are also implicated in the establishment and maintenance of cell polarity in epithelial tissues (reviewed in [37–40]). Candidates for each of these four ACD machineries, based upon studies in other model systems, were considered here. Other modules that generate ACDs exist [41], however, these four categories and our non-exhaustive list of 50 candidates within have been chosen based upon the rationale below, and are described here and summarized in Additional file 1:

1.  Cortical domain establishment: Symmetry breaking in a single celled zygote requires the establishment of polarity axes that have been shown to involve cortically associated protein complexes and small effector proteins. Soon after fertilization, polarity axes are established through cortically associated protein complexes that define specific domains. Domain establishment is upstream of spindle orientations and serves as the starting point for a determination of polarity axes beginning in a single cell. For a list of candidate genes see Additional file 1: Table S1.
2.  Spindle orientation: Following cortical domain establishment, the mitotic spindle orients itself using cues provided by the opposing cortical domains (reviewed in [29, 31, 42, 43]). Orientation requires a dynamic process to link the mitotic spindle to polarity cues provided by the cortical domain proteins (reviewed in [30, 44, 45]), to shuttle motor proteins towards the microtubule capture machinery (reviewed in [46, 47]), and finally, to activate the motor proteins that function to pull microtubules towards the plasma membrane (reviewed in [48]). In silico searches in various model organisms generated a list of 13 key proteins/complexes that mediate spindle orientation. Here we divide the list into two groups: (1) Spindle orientation proteins that mediate the transfer of polarity cues from cortical domains to proteins anchored in the plasma membrane, and other proteins that mediate microtubule capture at the plasma membrane orientation complex, and (2) Spindle orientation proteins that constitute, or shuttle, the motor complex to the microtubule plus end and activate the force generating minus end tracking proteins. For a list of candidate genes see Additional file 1: Table S2.
3.  Polarity complexes: Polarity complex proteins are known for establishing and maintaining two opposing subcellular plasma membrane domains. These complexes are typically found in polarized cells such as epithelial cells, neural cells, and early embryos (reviewed in [4, 32, 35, 49]). The juxtaposed domains are segregated into an apical and basolateral domain (reviewed in [4, 32, 50]). The creation of an apical domain is also a prerequisite for the subsequent formation of Adherens Junctions (AJs) and Septate Junctions (SJs) [32, 33, 49] (Tight Junctions in vertebrates), as well as for reinforcing protein complexes that maintain the basolateral domain [32, 33, 49]. For a list of candidate genes see Additional file 1: Table S3.
4.  Cell-cell adhesion and cell recognition complexes: Cell-Cell adhesion proteins form a recognition code that orients the cell, in relation to other cells, within its microenvironment [37, 51]; contact interactions that can be transmitted internally through second messengers (as demonstrated by adhesion G-Protein coupled receptors [52]). In addition, internalization of polarity cues can occur through interactions with scaffold proteins [53]. Epithelial and cortical complex proteins can also form permanent or transient junctions with homologous (protein binding actions include homophilic, heterophilic, or both) complexes on adjacent cells that communicate with other internal polarity cues [53–55]. These complexes include Septate Junctions (Tight Junctions in vertebrates) and Adherens Junctions that serve as an extracellular polarity cue that is transmitted internally to establish an Apical/Basal polarity. Additionally, some cell adhesion proteins interface with Planar Cell Polarity (PCP) proteins to establish other forms of internal polarity [37]. For a list of candidate genes see Additional file 1: Table S4.

To gain insight into the molecular machinery that contributes to ACDs in a spiral-cleaving embryo, we exploited recently established comprehensive RNA-seq based transcriptome data for the zygote, and spiral cleavage stages of the annelid *Platynereis dumerilii* [56]. Here we use various Gene Ontology (GO) term analyses and BLAST searches to identify mRNAs that code for proteins implicated in ACD. Interestingly, we found that transcripts of most ACD implicated genes exhibit a strong maternal contribution, and subsequently, slow decrease in later stages during the transition to bilaterally symmetric cell divisions. Intriguingly, a few core candidate genes exhibit early zygotic onset of expression suggesting potential regulatory roles. This analysis also discovered high levels of co-expressed ‘neural’ adhesion complexes during early embryogenesis previously described to have specialized functions in cell-cell recognition in the nervous system. Overall, our analyses suggest that this spiralian oocyte constitutes a highly specialized cell that contains extremely elevated transcript levels for ACD factors; presumably to facilitate rapid ACDs during subsequent early spiral cleavage stages.

## Methods

### *Platynereis dumerilii* Culture

Iowa State University maintains a culture of the marine annelid *Platynereis dumerilii* through sexual maturation and mating including embryos and larval stages according to the protocols described at www.platynereis.de [10, 57]. To ensure constant temperature and synchronize development, embryos were immediately placed in an 18 °C incubator upon fertilization. Post fertilization, batches of developing embryos were checked at 1 hpf for the proper formation of a jelly coat, and in a retained subset of control embryos at 24 hpf and 48 hpf for normal morphology and stage specific behavior. Only fertilization batches in which >80% of eggs were fertilized, and developed normally through 48 hpf were used for RNA isolation and embryo fixation. RNA isolation and embryo fixation procedures were performed as previously described [56–58].

### Transcriptome assembly

Methods and data sets were previously described in [56, 59–62].

### Identification of orthologous genes by in silico searches

Categories of genes related to ACD were initially identified through searches using specific Gene Ontology terms (see results), on Pdumbase http://pdumbase.gdcb.iastate.edu/platynereis/controller.php?action=home [63]. Secondly, comprehensive lists of candidate genes implicated in different aspects of ACD in several model organisms including *C. elegans*, *Drosophila*, and vertebrates were compiled from the primary and secondary literature (see results; Additional file 1: Tables S1–4). Orthologous *P. dumerilii* genes were identified both via (1) searches for distinct ACD candidate gene names in the automatically annotated *Platynereis* database, and (2) TBLASTN searches utilizing conserved vertebrate and invertebrate protein sequences retrieved from the NCBI website (https://www.ncbi.nlm.nih.gov/ [64]) against the assembled sequences deposited in the early *Platynereis* developmental transcriptome database. Identified *P. dumerilii* sequences were translated through the ExPASy website (https://web.expasy.org/translate/ [65]), and orthology to distinct ACD genes was confirmed by reciprocal best BLAST-hit analysis to the NCBI protein database. Thus, initial orthology assignments based on the automated *Platynereis* database annotations and phylogenetic analyses of homologous gene families were further scrutinized and corroborated manually including reciprocal best BLAST-hit analyses, and consideration of percent sequence homology, and structural conservation of protein domains.

Additionally, novel candidate genes potentially contributing to embryonic ACD in *P. dumerilii* were identified through their characteristic expression profile during early development. Candidate genes that belong in these categories exhibited a high level of expression at 2 hpf (the one-cell zygote) that we interpret as high maternal contribution, and were implicated by annotation in epithelial and/or neural adhesion and/or polarity mechanisms including planar cell polarity and neural recognition code components (see results; Additional file 1: Table S4). These *P. dumerilii* candidate genes were fully scrutinized and corroborated for orthology as described above.

### Cloning of ACD genes

Sequences for ACD genes were obtained from the assembled transcriptome [56]. Primer design occurred using online Primer designer Primer 3 http://biotools.umassmed.edu/bioapps/primer3_www.cgi [66]. Primers were designed to target coding regions of genes, averaged 1 kb of target sequence, and ranged from ~0.5 kb to 2 kb (see Additional file 2 for primer sequences). Gene specific primers were used in PCRs to amplify gene fragments from 2 hpf (one cell zygote) cDNA libraries. The amplified targets were verified for size on a 1% agarose gel. The PCR products were purified (Thermo Scientific purification kit) and ligated into the pGEM-T Easy cloning vector and subsequently used to transform competent *E. coli* bacteria. The transformed cells were plated on agar plates containing ampicillin. Selected colonies were grown in ampicillin Lennox Broth cultures. Cultures were then stored in a 1:1 ratio of culture and 87% glycerol for storage at -80 °C. Plasmids were isolated using the Promega miniprep kit. Isolated plasmids inserts were PCR amplified using primers designed to utilize the vector’s inserted sequences that allow for subsequent transcription of synthetic anti-sense RNA. Insert targets were then sequenced at the Iowa State DNA sequencing facility. Sequences were verified through the *Platynereis dumerilii* database [63]. Sequence information for each ACD gene is summarized in Additional file 2, and has been submitted to Genbank (Accession numbers: MG197654 to MG197703).

### Whole mount in situ hybridization

RNA probe templates were generated from plasmids with inserted clones using plasmid specific primers. Antisense and Sense RNA probes were generated using Sp6 (Roche), T3 (Thermo Scientific), or T7 (New England Biolabs) RNA polymerase kits. Probes were labeled using DIG RNA labeling mix (Roche). Probe length averaged ~1000 nucleotides. Oocytes, embryos, and larvae were subjected to an overnight fix on a nutator at 4 °C and stored in methanol. Protocols for Whole-mount in situ hybridization (WMISH) were followed with modifications described here [57, 67]. Each antisense probe was tested and validated for specificity by WMISH in late larval stages (48hpf; Additional file 3: Figure S1). Background controls, (1) sense probes for the two highest expressed maternal transcripts (*pcd11x* and *l(2)gl*) (Additional file 4: Figure S2), and (2) antisense probes for transcripts (e.g. *nodal*) that exhibit no maternal expression based on RNA-seq data did not show any expression by WMISH in unfertilized (0 hpf) and (2 hpf) specimen (data not shown) validating the specificity of the observed maternal expression of ACD genes. Specimens were stored in 87% glycerol for storage at 4 °C. Embryos were imaged on Zeiss Imager A2 microscope with a Zeiss Axiocam 503 Color camera. Contrast and brightness image adjustments were performed in Adobe Photoshop.

## Results

### Identification and classification of ACD genes

**Fig. 3:**
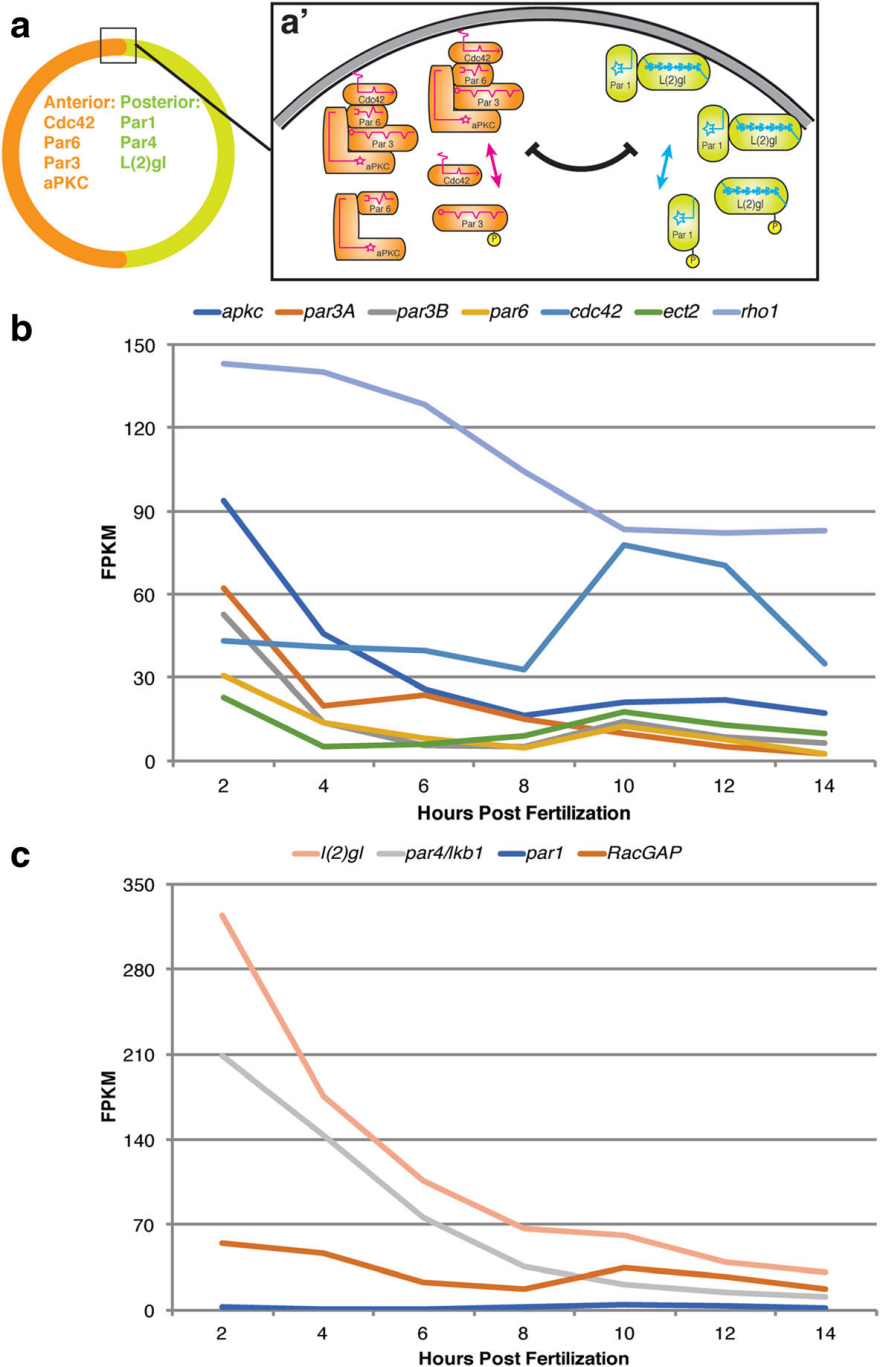
ACD components establishing cortical domains. **a** Schematic drawing illustrating localization of ACD components establishing two opposing asymmetric cortical domains within one cell based upon previous work including *C. elegans*. **a‛** Detail of (**a**) illustrating the juxtaposed cortical ACD complexes. Anterior Par network proteins (Cdc42, Par3, Par6, and aPKC) form an anterior cortical complex (orange). Posterior network Par proteins (L(2)gl, and Par1) associate to form a posterior cortical complex (green). Arrows indicate assembly of cortical complexes from individual ACD components. Both complexes mutually repress each other through reciprocal phosphorylation of components. **b**, **c** Transcriptional profiles of cortical domain components during early development of *P. dumerilii* based on RNA-seq: x-axis shows time in hours post fertilization (hpf), y-axis shows median level of transcripts in fragments per kilobase per million reads (FPKM) from two independent biological replicates (Additional file 5). Expression levels for most components are high at 2 hpf and decrease during later stages. **b** Expression profiles of anterior cortical domain components. **c** Expression profiles of posterior cortical domain components. For error bars see Additional file 9

**Fig. 4:**
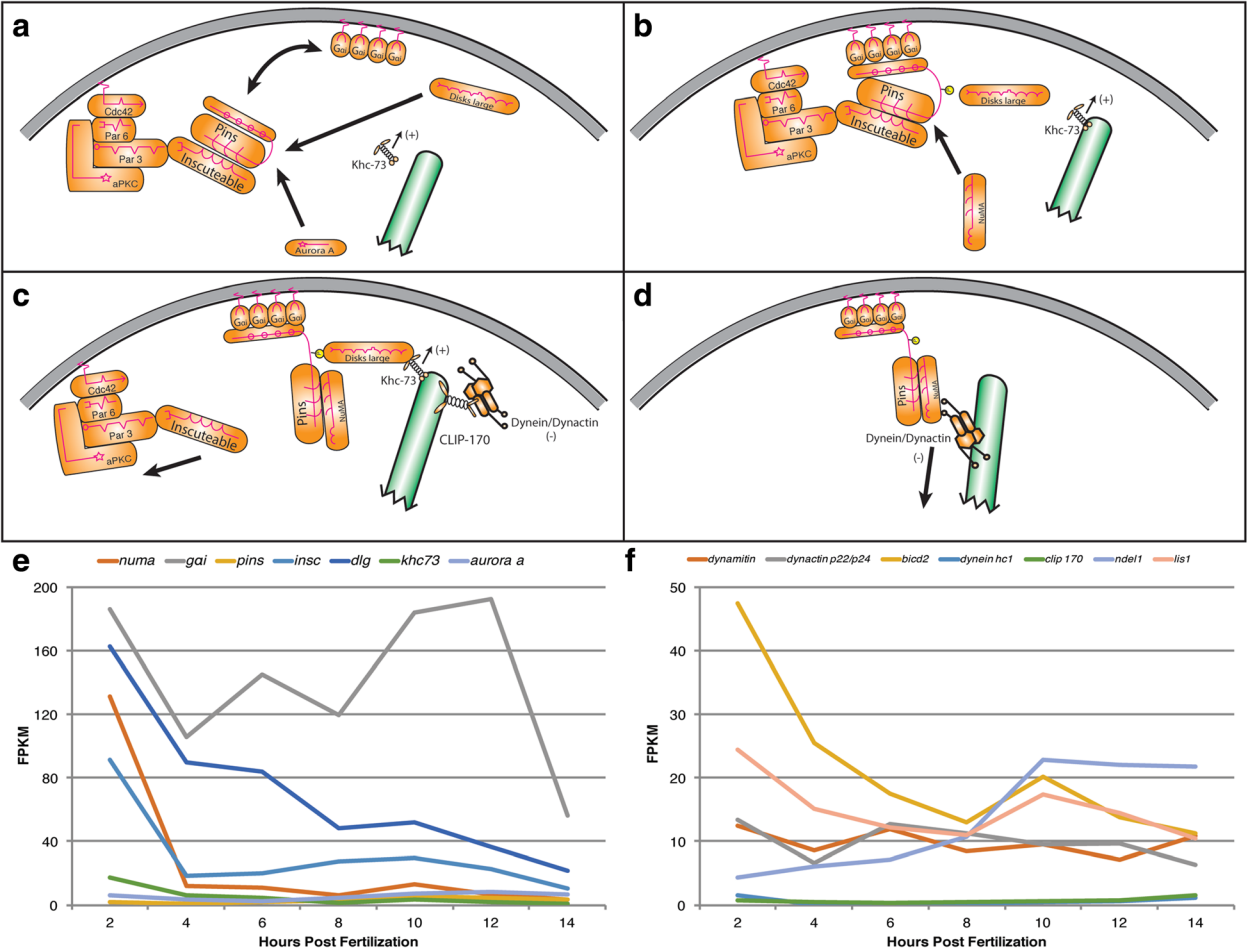
ACD components that control mitotic spindle orientation. **a**-**d** Schematic drawings illustrating dynamic molecular mechanisms of spindle orientation derived from previous studies in *C. elegans* and *Drosophila.* Individual ACD components are shown (orange), spindle microtubule (green). **a** Mitotic spindle orientation proteins Insc and Pins bind Par3 (cortical domain complex). Pins binds G_αi_ linking Pins to cortex. Aurora A phosphorylates Pins linker region allowing Dlg binding. **b** Plus end tracking microtubule-associated protein Khc-73 binds Dlg capturing microtubules at the cortex. **c** NuMA out-competes Insc to bind Pins. Dynein/Dynactin complex is transported to microtubule plus end by CLIP-170. **d** NuMA binds Dynactin complex while BicD (not shown) activates Dynein initiating pulling forces. **e**, **f** Transcriptional profiles of spindle orientation components during early development of *P. dumerilii* based on RNA-seq: x-axis shows time in hours post fertilization (hpf), y-axis shows median level of transcripts in fragments per kilobase per million reads (FPKM) (Additional file 5). Expression levels for most components are high at 2 hpf, decreasing during later stages. **e** Expression profiles for ACD components known to connect cortical cues to motor complexes. *g*_*αi*_ shows atypical expression possibly due to multiple functions as a G-protein. *pins* exhibits low level expression. **f** Expression profiles for components encoding force-generating motor complexes. Dynactin complex contains 23 subunits including Dynamitin, and Dynactin 4 (two representative transcripts shown here). Dynein contains multiple subunits, represented by transcripts coding for Dynein Heavy Chain 1. Transcripts for six Dynactin and multiple Dynein complex components show similar expression patterns (data not shown). *clip-170*, which codes for a protein that aids in Dynein/Dynactin recruitment to the cortex, is expressed at low levels. Dynein cofactors BicD2 and Lis1, which regulate Dynein function and processivity, show similar expression patterns with *bicd2* expressed at high levels at 2 hpf. *ndel1* shows increasing expression throughout early embryogenesis. For error bars see Additional file 10

**Fig. 5:**
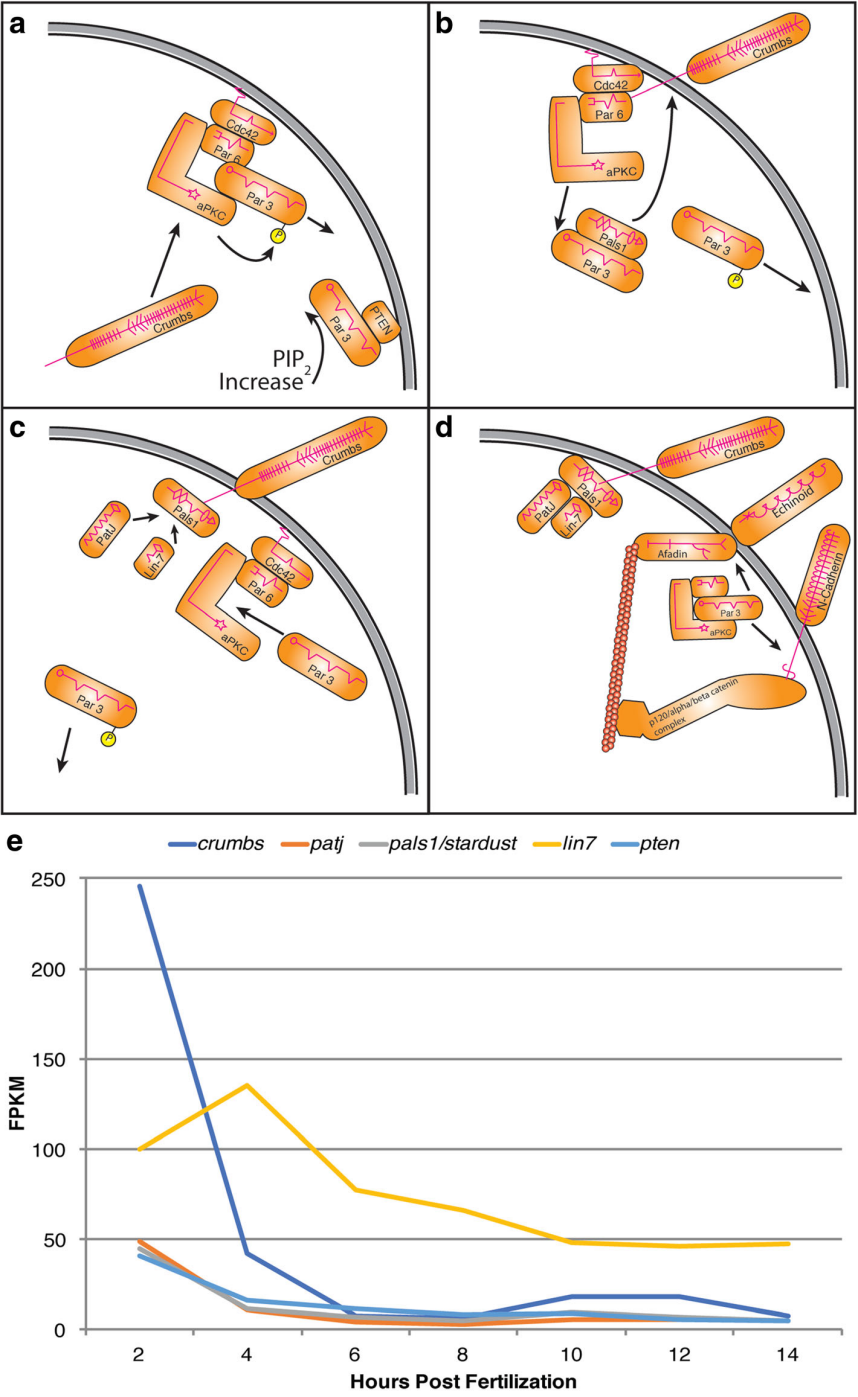
ACD components that convey apical associated polarity cues. Schematic drawings illustrating proposed mechanisms establishing apical/basal cell polarity derived from previous studies in *C. elegans* and *Drosophila.* **a**-**d** Dynamic interactions establishing apical Crumbs complex. **a** aPKC phosphorylates Par3 weakening Par3 bond with aPar complex. Crumbs outcompetes phosphorylated Par3. Unphosphorylated Par3 binds PTEN increasing PIP_2_ in apical domain. **b** Par3 recruits Pals1 to apical membrane where aPKC phosphorylates Par3, weakening association of Par3 to Pals1. Pals1 outcompetes aPKC-Par6-Cdc42 complex with Crumbs. **c** PatJ and Lin7 are recruited to Pals1 to stabilize Crumbs complex. Par3 reestablishes itself with aPKC-Par6-Cdc42 complex. **d** aPar complex interacts with Afadin to recruit Echinoid to lateral domain. Likewise, aPAR complex also interacts with p120/α/β-catenin complex to establish immature AJs with N-Cadherin. Afadin and p120/α/β-catenin adaptors link extracellular domain proteins Echinoid and N-Cadherin to actin cytoskeleton. Establishment of Crumbs complex stabilizes apical domain and defines lateral domain. **e** Transcriptional profiles of Crumbs complex components, early stages of *P. dumerilii* based on RNA-seq: x-axis—time in hours post fertilization (hpf), y-axis— median level of transcripts in fragments per kilobase per million reads (FPKM) (Additional file 5). Transcript levels for *crumbs*, *patj*, and *pals1* show high expression at 2 hpf. *lin7* exhibits elevated zygotic transcription in early cleavage stages. For error bars see Additional file 11

**Fig. 6:**
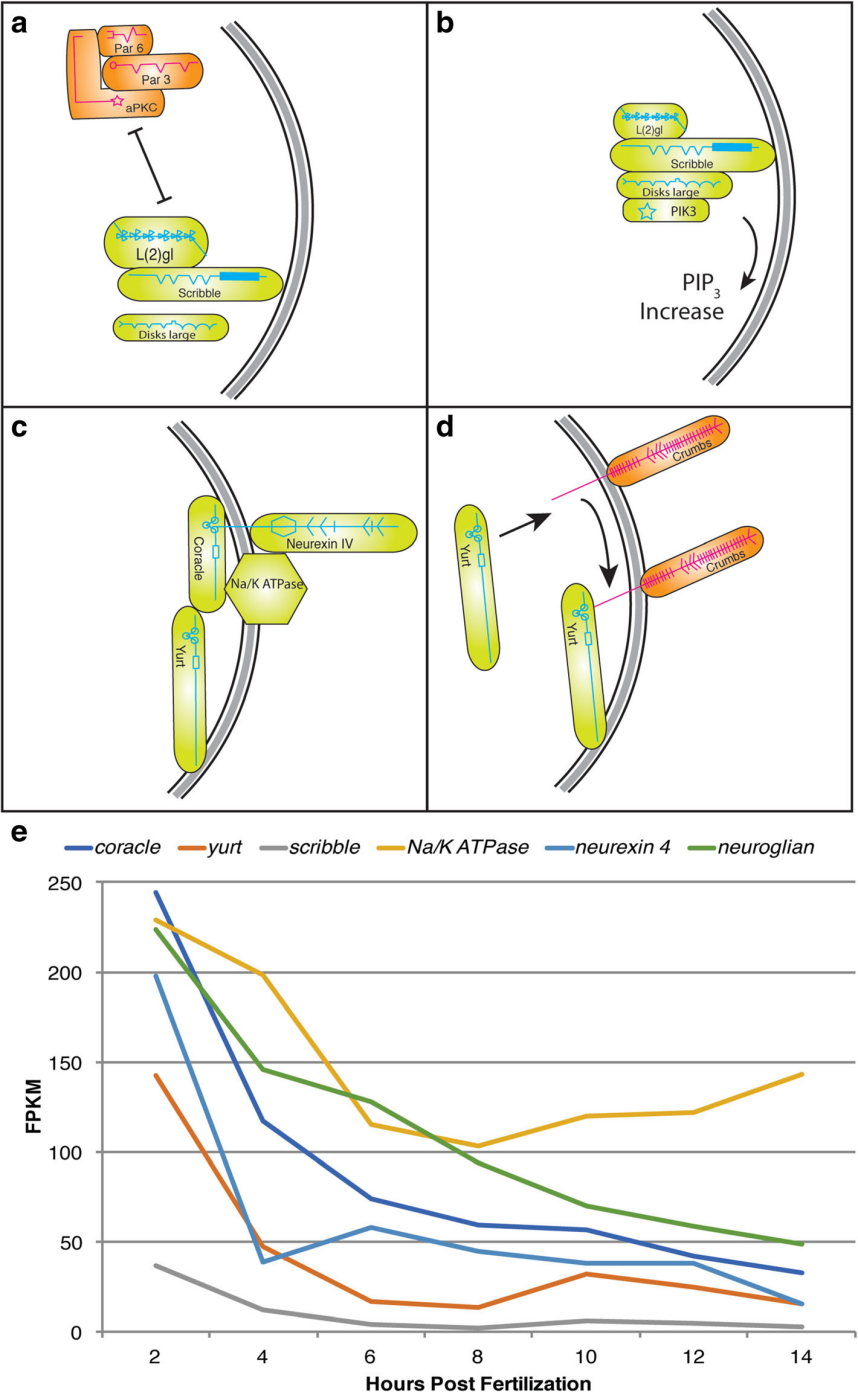
ACD components that convey basolateral associated polarity cues. **a**, **b** Dynamic interactions establishing basolateral Scribble complex. **a** Scribble interacts with Dlg and L(2)gl to antagonize aPAR complex, maintaining basolateral domain. **b** Scribble complex protein Dlg binds PIK3, increasing PIP_3_ concentration in basolateral domain. **c**, **d**. Formation of basolateral Yurt/Coracle complex. **c** Yurt/Coracle complex includes ion pump Na^+^/K^+^- ATPase and extracellular domain protein Neurexin IV. **d** Yurt antagonizes Crumbs complex by binding intracellular domain of Crumbs. **e** Expression profiles for components forming complexes in basolateral domain. Transcript levels for all genes are high at 2 hpf relative to later time points. *na* ^*+*^ */k* ^*+*^ *-atpase* shows an increase at 4 hpf. The genes *numb* and *brat* were included with this group but are not presented in diagrams or as curves on the time (hpf) vs FPKM values graphs. Their data has been included in Additional file 5. For error bars see Additional file 11

**Fig. 7:**
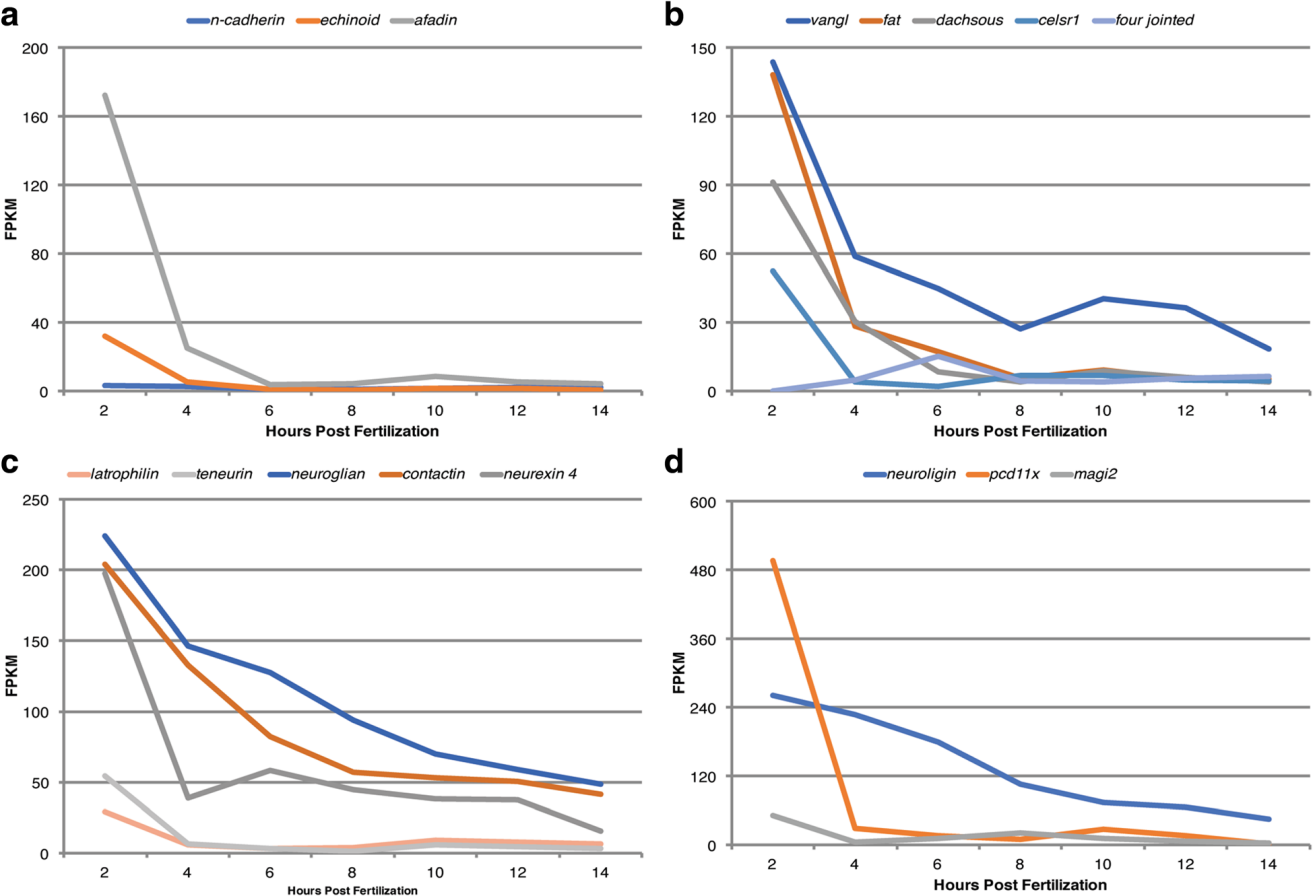
Maternally provided cell adhesion related complexes. (a-d) Transcriptional profiles of components for various cell adhesion related complexes during early development of *P. dumerilii* based on RNA-seq: x-axis shows time in hours post fertilization (hpf), y-axis shows median level of transcripts in fragments per kilobase per million reads (FPKM) (Additional file 5). **a** Expression profiles for components of cadherin-mediated cell-cell adhesion complex. *Echinoid* and *afadin* exhibit high levels of transcripts at 2 hpf. *n-cadherin* shows slightly elevated levels of expression at 2 hpf compared with later time points. **b** Expression profiles for selected components of the planar cell polarity (PCP) pathway. Four-jointed is an intracellular kinase that regulates Fat and Dachsous through phosphorylation. Transcripts for extracellular domain proteins Vangl, Fat, Dachsous, and Celsr1/Flamingo exhibit high levels of expression at 2 hpf, whereas levels for *four-jointed* shows early zygotic expression beginning around 4 hpf and peaking at 6 hpf. **c**, **d** Expression profiles for components with previously described functions in neural cell recognition. Latrophilin 1 and its binding partner Teneurin translate polarity cues to internal signaling cascades, whereas Neuroglian and Contactin mediate cell-surface polarities at the juxtanodal region of Nodes of Ranvier in myelinated neurons. **c** Components for each complex exhibit high maternal contribution and similar developmental expression profiles in *P. dumerilii*. At synaptic connections, Neuroligin binds Magi2 intracellularly and is asymmetrically in postsynaptic neurons. Also, the protocadherin Pcd11x negatively regulates dendritic branching in neurons. **d** All three genes are expressed at high levels at 2 hpf relative to later time points in *P. dumerilii*. For error bars see Additional file 12

The identification of genes that are involved in ACD, in *P. dumerilii,* was made using the previously established developmental transcriptome [56]. This recent work identified and quantified expression level for every transcript at 2-h intervals during the first 14 h of early embryogenesis in *P. dumerilii* (see timeline in Fig. 2d). Importantly, each stage-specific data point was generated by RNA-seq from polyA selected mRNA extracted from batches of thousands of synchronously developing embryos, with two biological replicates for each stage collected from independent individual matings. The combined sequencing data of the collected >750 million reads was used to generate an early *P. dumerilii* transcriptome by de novo assembly including ~28,000 predicted protein coding transcripts corresponding to ~19,000 genes. Subsequently, 25 to 40 million reads for each sample were mapped to the gene models to quantify the stage-specific expression levels for each transcript. Notably, stage-specific measurements for each individual transcript were highly reproducible between biological replicates (Spearman correlation coefficient > 0.91; see Additional file 5). Therefore, the median expression level for each developmental time point can be used to establish a developmental expression profile for each gene (data shown in Figs. 3, 4, 5, 6 and 7).

Developmental stages targeted in the previous study, and analyzed here, were timed by hours of development after fertilization occurred (hours post fertilization, hpf) at a standardized temperature of 18 °C. Seven stages were included 2, 4, 6, 8, 10, 12, and 14 hpf (from the fertilized egg to the ~330-cell stage (Fig. 2d)). The 2 hpf time point corresponds to the fertilized egg/zygote, after the completion of the meiotic cell divisions as marked by the extrusion of two polar bodies at the animal pole, but before the first mitotic cell division. During the next six hours of development, the core program of spiral cleavages unfolds with the transition from the 8- to 16- cell stage at 4 hpf, the transition through ~30-cell stage at 6 hpf, and the completion of the main spiral cleavages (by the end of the 6th cell division) shortly before 8 hpf [19, 26]. After the initial spiral cleavages, ACDs continue exhibiting stereotypic spindle orientations and invariant cell size asymmetries. However, later stage ACDs generate a more bilaterally symmetric cleavage pattern transitioning through the ~140-cell stage at 10 hpf, continuing through the ~220-cell stage at 12hpf, and ~330-cell stage at 14 hpf [19, 26].

Our previous description of global gene expression in early *P. dumerilii* embryogenesis defined several large cohorts of maternally contributed transcripts at 2 hpf, the staggered early zygotic expression of smaller clusters of genes at 4 hpf, 6 hpf, and 8 hpf respectively, and the zygotic onset of expression of several larger cohorts of genes after 8 hpf, the presumed ‘midblastula transition’ [56]. Here, the focus on the expression levels of transcripts encoding ACD components at the different time points will be informative to determine the presence and input of transcripts that encode the ACD machinery within the fertilized egg at 2 hpf (maternal contribution; see discussion). Furthermore, ACD expression levels will also indicate the maternal and zygotic inputs into the spiral cleavage program (2 hpf to 8 hpf), into the transition to embryonic bilateral symmetry (8 hpf to 14 hpf) coinciding with onset of gastrulation after 8 hpf, and hatching of a ciliated protrochophore larvae at 12 to 14 hpf [19, 20, 26].

The identification of potential components for ACD utilized two strategies, general and specific, to discover candidate genes. Our general approach used the GO term annotations for each of the ~13,600 annotated genes in the dataset [56] to find genes with the GO terms: cell polarity (Additional file 6), cell adhesion (Additional file 7), and cell junction (Additional file 8). These additional files include genes in these Search categories that have a minimum expression of 1 FPKM in at least one of the seven stages, an arbitrary but inclusive cut-off empirically chosen based on the observation that expression in individual cells (as determined by Whole Mount in situ Hybridization (WMISH)) generally require an FPKM larger than 5 in our RNA-seq data set [56–58]. In general, these gene cohorts exhibit high levels of transcripts in the 2 hpf one-celled embryo (FPKMs generally between 20 and 300), followed by a slow decrease in expression levels in later stages. This suggests a high maternal contribution of mRNAs encoding potential components for ACD in the zygote.

Our searches by GO terms in categories potentially associated with ACD discovered scores of genes present in early stages, and draw attention to genes strongly expressed in the cell-cell adhesion category that current literature generally does not implicate in embryonic development. However, GO terms are not always comprehensive, depend on the previous automated annotations of the early *P. dumerilii* transcripts, and often fail to include well studied components of ACD described in other model systems. Therefore, we implemented a second specific search strategy for individual candidate genes that were functionally implicated in core mechanisms of ACD in various models. We focused on four ACD categories including establishing cortical domains prior to asymmetric cell division, the molecular mechanism to orient mitotic spindles, well-defined cell polarity complexes, and specific components of the cell-cell adhesion machinery. Candidates in each category were further subdivided, based upon previous work in other model systems that describe subcellular domain locations, molecular interactions, and/or mode of activity, action, or pathway. We used the protein sequence for each component from various organisms for TBLASTN searches within the early *P. dumerilii* transcriptome database, identified predicted gene models with the highest degree of conservation as potential homologs, validated orthology assignments by subsequent reciprocal best BLAST-hit analyses against NCBI databases, and through phylogenetic tree constructions (data not shown). Each of the identified predicted *P. dumerilii* gene models were confirmed by establishing complete or partial cDNA clones from cDNA generated from embryonic 2 hpf mRNA, and subsequent sequencing (Additional file 2). Expression was validated by WMISH at 2 hpf and selected later stages (see below).

### Category I. Establishing cortical domains: The par gene complexes

To gain insights into presence and expression levels of transcripts encoding the Par gene interaction network in early *P. dumerilii* embryos, we identified orthologous genes and expression profiles within our database (Fig. 3, Additional file 1: Table S1, and Additional files 5 and 9). Each of the Par complex components exhibits high transcript levels in the 2 hpf zygote, a steep decrease between 2 hpf and 4 hpf, followed by a slower decrease until 8 hpf, an increase or steady maintenance between 8 hpf and 10 hpf, and further decreases in later stages. The mRNA expression levels for the small effector proteins Cdc42 and Rho-1 shows similar high expression at 2 hpf but varies more in later stages, possibly due to multiple functions unrelated to a shared potential role in cortical domain establishment. Expression levels range from 30 to >300 in FPKM (*par6*: 30; *par3B*: 53; *par3A*: 62; *apkc*: 94; *par4/lkb1*: 209; *l(2)gl*: 325) at 2 hpf, to 2 to 31 FPKM (*par6*: 3; *par3B*: 6; *par3A*: 2; *apkc*: 17; *par4/lkb1*: 11; *l(2)gl*: 31) at 14 hpf. Interestingly, a second *P. dumerilii* Par3 homolog *par3A* shows a substantial increase between 4 hpf and 6 hpf suggesting perhaps a role for this Par gene duplicate during early embryogenesis in *P. dumerilii*. Curiously, transcript levels for *par1* are low throughout early *P. dumerilii* development (*par1*: 1 to 4 FPKM). In conclusion, almost all components of the Par gene network, required to set up initial cortical asymmetries, are present in the fertilized egg as transcripts with elevated copy numbers suggesting high maternal contribution for each. If the Par network operates in early embryogenesis to initiate ACDs as suggested by our findings it might do so without or with a reduced role for Par1.

### Category II: The machinery to orient the mitotic spindle

Searches for orthologs for each component in this category within the *P. dumerilii* transcriptome confirmed the identity and presence for every component encoding ACD machinery in *P. dumerilii* embryos at early stages (Fig. 4, Additional file 1: Table S2, and Additional files 5 and 10). Levels for transcripts encoding proteins that link spindle orientation machinery to cortical domain proteins, with the exception of *pins*, are strongly elevated at 2 hpf ranging from 6 to >180 FPKM (*aurora a*: 6; *khc-73*: 17; *insc:* 91; *numa*: 131; *dlg*: 162; *g*_*αi*_: 186) and decrease in later embryonic stages (Fig. 4e). Decrease between 2 hpf and 4 hpf is largest for *numa* and *insc* with a drop from 131 to 12 and 91 to 19 FPKM, respectively, and more moderate for others (*dlg, khc-73*). Interestingly, transcripts for *pins*, a central component of the ACD machinery in *Drosophila* [68–74], appear to be present at very low levels. Transcripts encoding components of the motor complex vary in their respective expression profiles (Fig. 4f). Some exhibit moderate expression at 2 hpf including two of six dynactin subunits, dynamitin and p22/p24, between 12 to 13 FPKM respectively, compared to four other subunits that are expressed at varying levels during early time points at 2 to 30 FPKM (data not shown), and *bicd2* and *lis1* with 47 and 24 FPKM, respectively. Most components in this subcategory remain expressed at more constant, moderate expression levels, throughout early embryogenesis with Ndel1 being the exception as *ndel1* increases expression consistently throughout early stages before leveling off at 10 hpf from 4 to ~22 FPKM. Two notable exceptions are the essential motor component *dynein heavy chain*, *tctex-type 1*, and the Dynein/Dynactin Microtubule plus end transporter *clip-170,* both expressed at consistent low levels. In conclusion, the expression of these genes shows that spindle orientation components are present and maternally provided in *P. dumerilii*. Their conspicuous expression suggests that they may supply the machinery necessary to mediate the alternating and stereotypic spindle orientations, typical for spiral cleavage programs.

### Category III: Establishing and maintaining cell polarity

The potential roles of these complexes to establish embryonic cell polarity and/or contribute to cell size asymmetry mechanisms during ACD motivated our search in early *P. dumerilii* embryos (Figs. 5 and 6, Additional file 1: Table S3, and Additional files 5 and 11). Indeed, we were able to identify each component within the transcriptome of *P. dumerilii* at 2 hpf, and found a similar pattern of mRNA expression as with the *par* genes, *l(2)gl*, and *dlg* (see Figs. 3 and 4). Transcript levels for several key components are very high at 2 hpf ranging from 143 to 246 FPKM (*yurt*: 143; *neurexin-IV*: 198; *cora*: 244; *crumbs*: 246), showing a steep decline between 2 hpf and 4 hpf, at 47, 39, 117 and 42 FPKM respectively, and a lower expression level ranging from 7 to 49 FPKM, depending on the transcript, in later stages (Figs. 5e, 6e). Several components (*patj*, *pals1/stardust*, *pten*, and *scribble*) exhibit elevated expression at 2 hpf of 37 to 48 FPKM, a steeper decrease to 10 to 12 FPKM at 4 hpf, and a lower steady expression level between 3 and 5 FPKM in later stages (Figs. 5e, 6e). The exception is *lin-7* showing a strong increase between 2 hpf and 4 hpf from 100 to 135 FPKM, putting *lin-7* into the rare category of very early zygotically expressed genes within the *P. dumerilii* embryo (only <50 genes of 28,400 belong into this category) [56]. Although *lin-7* shows the typical decline throughout later stages, expression levels remain elevated relative to other transcripts within the Crumbs complex ranging from 80 to 40 FPKM. In conclusion, the fertilized egg exhibits a high maternal contribution for all components that form the three polarity complexes (Crumbs, Scribble, and Yurt/Cora), with some components conceivably serving as limiting factors, suggesting potential regulatory roles in ACDs in early embryogenesis for *P. dumerilii*.

### Category IV: Cell-cell adhesion and cell recognition complexes

Previously discussed polarity complexes define the boundaries of the immature Zonula Adherens that, subsequently, promote the formation of Adherens Junctions (AJs) and Septate Junctions. A critical part in junction formation, in various model systems, is the establishment and stabilization of transient cell-cell contacts and this motivated our search for similar components in early *P. dumerilii* embryos (Fig. 7, Additional file 1: Table S4, and Additional files 5 and 12).

In *P. dumerilii*, the AJs component *n-cadherin,* is represented by low constant mRNA levels (Fig. 7a). Interestingly, *echinoid,* and especially *afadin,* mRNA can be found in elevated, 32 FPKM, and very high, 156 FPKM levels respectively, in 2 hpf *P. dumerilii* embryos (Fig. 7a, Additional file 12). It would be difficult to imagine mature AJs forming during early embryonic divisions. However, due to the signature expression levels one may speculate that immature spot AJs could play an important role during polarization to establish and regulate cell-cell contacts required to generate invariant spiral cleavage patterns.

The early transcriptome of *P. dumerilii* contains mRNA expression profiles for each component of the PCP pathway (Fig. 7b, Additional file 12). At early time points, *P. dumerilii* embryos transcript expression levels for *fat*, *vangl*, *dachsous*, and *celsr1* are very high at 2 hpf ranging from 52 to 144 FPKM, followed by a steep decrease at 4 hpf, and less pronounced decreases in later stages to an average level of 4 to 10 FPKM while *vangl* stays elevated in relation to other PCP components between 19 and 41 FPKM throughout later stages. Intriguingly, a key regulator of the PCP pathway, *four-jointed,* is not maternally provided but increases steadily from 2 hpf to 6 hpf, with 0 to 16 FPKM respectively, before decreasing to a lower level between 4 and 7 FPKM in later stages. Therefore, in conclusion, the high maternal contribution of the major PCP components to early *P. dumerilii* embryos and the early zygotic expression of *four-jointed* may suggest a role for the PCP pathway and Four-Jointed to mediate cellular asymmetries during early pattern formation and ACDs during spiral cleavage stages.

Curiously, several groups of genes that are generally thought to perform cell adhesion mediated polarity functions specifically within the nervous system (see background, Additional file 1: Table S4) are also prominently present. In early *P. dumerilii* embryos one group including *neuroglian*, *contactin*, and *neurexin-IV/caspr* are highly expressed at 2 hpf with 224, 204, and 197 FPKM, respectively (Fig. 7c, Additional files 5 and 12). Specifically, *neuroglian* and *contactin* display very similar developmental expression profiles with a steady, moderate decrease in transcript levels from 2 hpf to 14 hpf. Transcript levels for a second group of genes, *latrophilin* and *teneurin* are elevated at 2 hpf with 29 and 55 FPKM respectively in *P. dumerilii*. Both transcripts decrease between 2 hpf and 4 hpf, and remain constant at ~2 to 9 FPKM during later stages. Intriguingly, a third group of three ‘neuronal’ adhesion genes also exhibit significant expression at early time points in *P. dumerilii* embryos (Fig. 7d, Additional file 12). *pcd11x* stands out with one of the highest expression levels at 2 hpf with 497 FPKM, a dramatic decrease between 2 hpf and 4 hpf to ~30 FPKM, and a constant level of expression between 10 and 28 FPKM in later stages. In contrast, *neuroligin* remains at a consistent high-level with slow decrease between 2 hpf to 6 hpf from 263 to 180 FPKM, and a steady decrease to 46 FPKM at 14 hpf. A similar early stage pattern is present for the Neuroligin intracellular binding partner *magi2* with 52 FPKM at 2 hpf and decreases to 5 FPKM at 4 hpf. However *magi2* exhibits increasing zygotic expression to 21 FPKM between 4 hpf and 8 hpf, before a slow steady decrease in later stages similar to *neuroligin*.

It is intriguing that several key components known to mediate cell-cell interactions on surface contacts between neuronal cells, and the machinery to transfer this information internally are all present in high copy numbers during early spiral cleavage stages. Although, studies of their roles in the nervous system are consistent with roles to convey polarity and subcellular spatial information, more general roles outside their function in the nervous system in cell polarity have not yet been fully described. The conspicuous high maternal contribution in *P. dumerilii* embryos suggests broader roles for these cell-cell adhesion mediators during spiral cleaving stages in *P. dumerilii* development.

### Transcripts for ACD components in the unfertilized egg (0 hpf) and zygote (2 hpf)

**Fig. 8:**
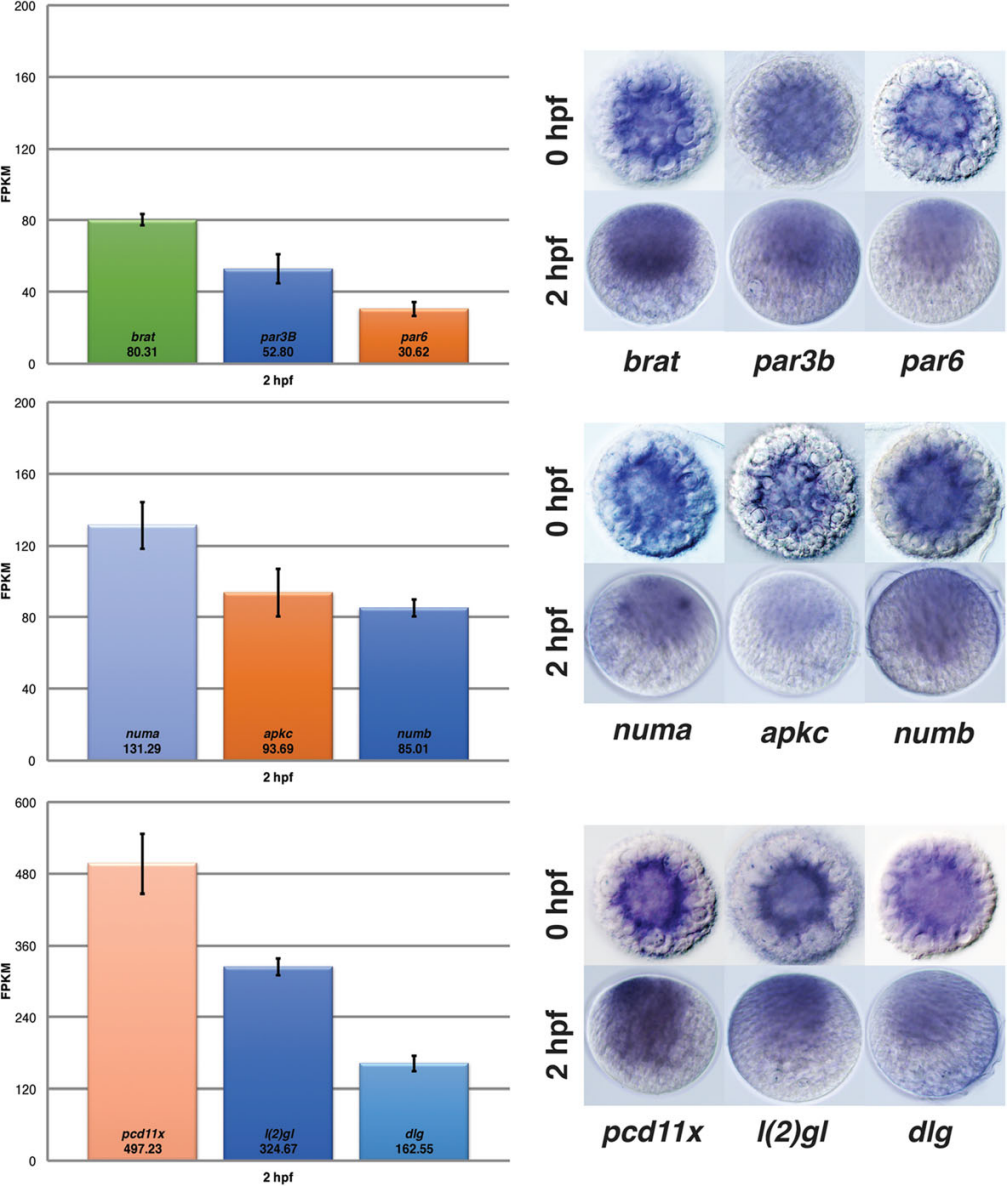
Validation of maternal ACD transcript levels in oocytes and zygotes. Expression levels in FPKM at 2 hpf of selected ACD components in *P. dumerilii* zygotes based on RNA-seq are shown on the left. WMISH of selected ACD transcripts in mature oocytes/unfertilized eggs (0 hpf; upper row) and zygotes (2 hpf; lower row) are shown on the right. Importantly, WMISH procedures including timing of substrate development and washes were performed simultaneously for 0 and 2 hpf specimen for each individual probe to ensure comparable staining intensities between both stages. In oocytes, transcripts are concentrated in the clear cytoplasm surrounding the oocyte pronucleus. In zygotes transcripts are localized in the clear cytoplasm at the animal pole after ooplasmic segregation (compare to Fig. 1). Every tested gene exhibits similar or higher expression levels in oocytes than zygotes validating the RNA-seq data at 2hpf, and confirming the high maternal contribution for each ACD transcript. Background control WMISH using sense probes for the two highest expressed genes *pcd11x* and *l(2)gl* did not show any staining in early and late stages (Additional file 4: Figure S2)

To validate ACD transcripts in the 2 hpf embryo and confirm the presence of high maternal contribution for ACD components in unfertilized eggs (0 hpf) we performed WMISH comparing both stages (Fig. 8). The stage ‘0 hpf’ is defined in this study as the unfertilized egg that has fully matured and is ready to be fertilized upon sperm contact. To test maternal contribution, we performed WMISH for nine selected ACD components on fixed 0 hpf and 2 hpf specimen in parallel, under identical conditions, or together in one vial. Importantly, to make these in situ results comparable between 0 hpf and 2 hpf when stained in separate vials, we kept the substrate incubation time the same for both vials during the staining procedure. Indeed, WMISH of 0 hpf and 2 hpf stages showed that the mRNA levels of ACD components were, in all cases, similar or even stronger at 0 hpf than 2 hpf (Fig. 8), although differentially localized. Due to the dramatic reorganization of the oocyte’s cytoplasm upon fertilization (ooplasmic segregation; see Fig. 1), the central clear cytoplasm in the unfertilized egg is redistributed and accumulates in a yolk free area at the animal pole in the 2 hpf zygote [19]. In mature oocytes (0 hpf), most transcripts, including ones encoding ACD components, localize to this central clear cytoplasm surrounding the maternal pronucleus. However, transcripts for ACD components in 2 hpf zygotes are more ubiquitously distributed with a preference for the clear cytoplasm that has segregated towards the animal pole. Background controls including sense probes for the two highest expressed maternal transcripts (*pcd11x* and *l(2)gl*) did not show any expression by WMISH in early and later stages (Additional file 4: Figure S2) validating the specificity of the observed expression of ACD genes. These results suggest that transcripts for ACDs are present from maternal sources at high levels. In summary, these results suggest that the high levels for transcripts of ACD components at 2 hpf are indeed maternally provided transcripts, inherited from the oocyte. Likewise, the results also suggest that the actual maternal transcript levels at 0 hpf are similar or even higher than the ones reported at 2 hpf by RNA-seq. Thus, our presented study demonstrates that an extensive machinery, to potentially execute ACDs, is maternally provided in *P. dumerilii*. The identification of the components in this study will provide a roadmap to functionally dissect their contribution to the molecular mechanism for ACDs during the spiral cleavage program.

## Discussion

### Survey of ACD components in the spiralian annelid *P. dumerilii*

**Fig. 9:**
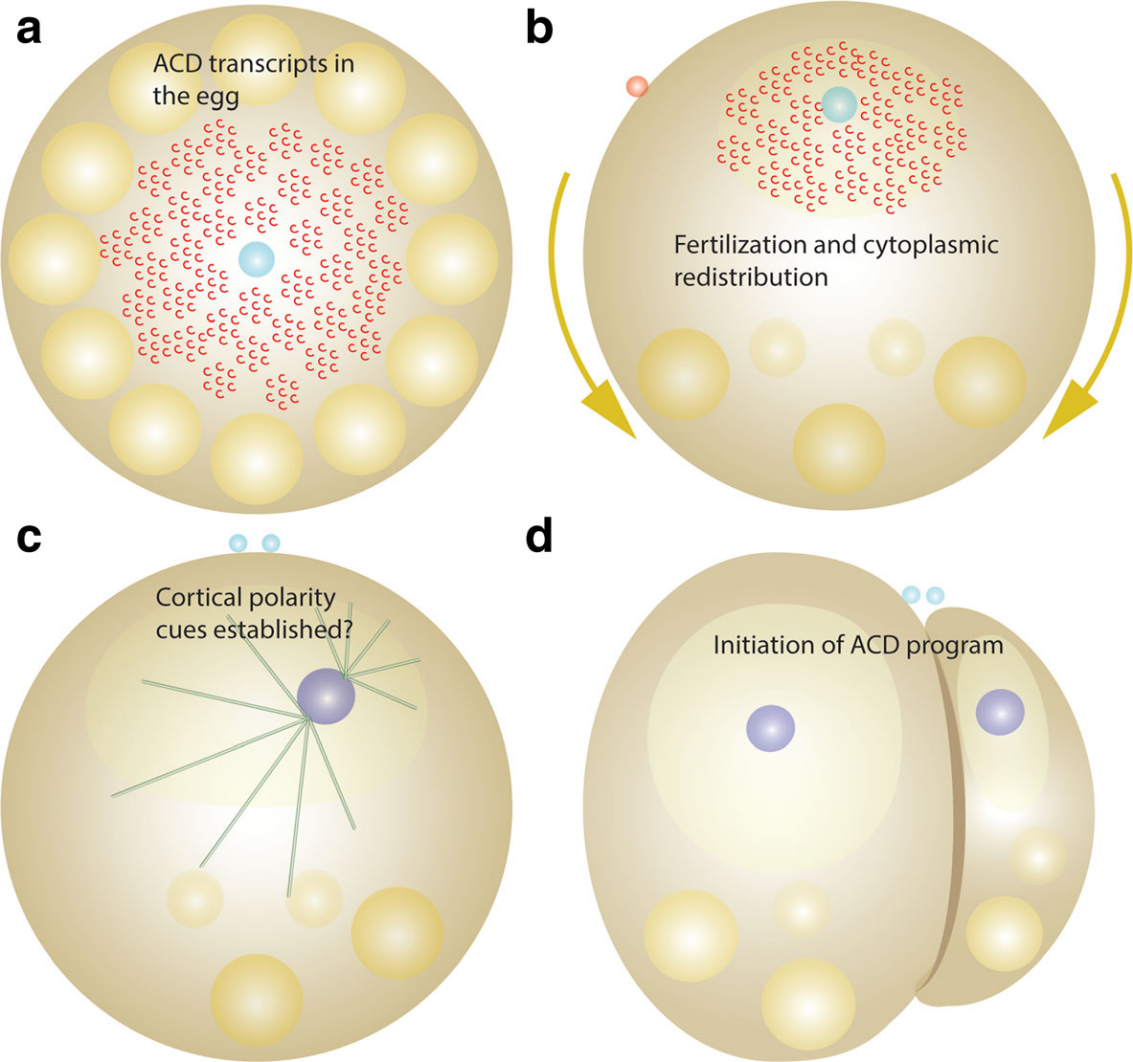
ACD transcripts in the spiralian egg and embryo. **a**-**d** Schematic illustrations of early embryonic stages, the mature oocyte (**a**), the zygote after ooplasmic segregation (**b**), prophase of the first mitotic cell division (**c**), and 2-cell stage (**d**). Compare to Figs. 1 and 2 for details. **a** The oocyte is loaded with centrally located mRNA transcripts (red) encoding ACD components. **b** Upon fertilization ACD transcripts within the clear cytoplasm relocate towards the animal pole in the wake of the cytoplasmic rearrangement within the zygote. **c**, **d** Hypothetical mechanism for ACDs in spiralians. Maternal ACD components form cortical domains, set up polarity cues, and generate asymmetries in orientation and architecture of the first mitotic spindle prior to the first cell division. Maternally provided ACD components provide the cellular machinery to perform the ACDs during early development generating the precise cell size asymmetries and spiral arrangements of embryonic cells in spiral cleaving embryos

The presented survey for ACD components in the spiralian annelid *P. dumerilii,* based on stage specific RNA-seq data, identifies (1) a highly conserved metazoan ACD gene set encoded in the *P. dumerilii* genome, and demonstrates (2) the presence of most of these components as highly expressed transcripts in the one-cell stage embryo. Thus, a full complement of ACD machinery including components to establish cortical domains, spindle orientation, cell polarity and various adhesion complexes are in place to execute the elaborate spiralian cleavage program. We suggest that the high maternal contribution of each ACD transcript constitutes a ‘maternal transcriptional ACD signature’ present within this spiralian egg. We speculate that this maternal provision is a requirement for the rapid execution of the subsequent asymmetric mitotic cell divisions, generating the iconic spiral cleavage pattern (Fig. 9).

### Asymmetric cell division in other spiralians

To the best of our knowledge, no comprehensive survey of ACD genes has been conducted in any spiralian species. Previously, some studies have illuminated certain aspects of ACDs in spiralians [13–17]. In the leech *Helobdella robusta*, Par network proteins have been implicated in ACD mechanisms [75, 76]. The asymmetric localization of Par1 and Par6 in the zygote is consistent with a potential role of the Par complex symmetry breaking mechanism during the first mitotic cell division [76]. Also, Cdc42 has been shown to be involved in ACDs in later stages [75]. Lambert and colleagues (2007) discovered a novel mechanism that asymmetrically segregates RNA transcripts in the snail *Illyanassa obsoleta*. During ACDs following the four-cell stage, 3–4% of transcripts localize and segregate asymmetrically to interphase centrosomes [77]. Although this mechanism might be a potent driver for ACD and cell fate specification in this early spiral-cleaving embryo, it is currently unknown whether other spiralian embryos use asymmetric mRNA segregation. It is also not known how transcript segregation might be related to the more broadly conserved ACD machinery including the activity of Par gene networks outside of *C. elegans* [78, 79].

### Asymmetric cell divisions in *P. dumerilii*

Previous work in *P. dumerilii* and closely related nereid species have described ACDs in *P. dumerilii* focusing on the remarkable invariant cell size asymmetries [19], stereotypic mitotic spindle orientations [18, 19, 80], and the establishment of polarity of egg and zygote [25]. Schneider and Bowerman (2007) discovered a binary β-catenin mediated cell fate specification mechanism that operates after every cell division in early stage *P. dumerilii* embryos to distinguish animal-pole versus vegetal-pole daughter cell fates [26]. Whether, and how, the maternal ACD machinery identified in this survey is involved in, and linked to the establishment of early zygote polarity, is unknown. Similarly, the molecular mechanisms for the subsequent asymmetric spiral cleavage divisions including the asymmetric binary β-catenin switches are also unknown, and will be an important target, and fertile ground, for future investigations.

### High maternal contribution of ACD components: A general phenomenon?

One of the central discoveries of this survey is the high maternal contribution of most ACD components within the *P. dumerilii* egg and zygote as shown by RNA-seq and WMISH. Although not unexpected, similar data has not been compiled for other model species. We suggest a closer investigation and quantification of transcript and protein levels in eggs and early embryos may be important to define similar and divergent features of various metazoan eggs. How similar is the content of various eggs and how do they differ? The bias for certain transcripts in eggs for differing species including different modes of early development will certainly be informative for the understanding of diverse types of cleavages and various reproductive strategies. Transcript biases will also shed light on any ‘specializations’ and/or unique ‘signatures’ reflecting the adaptations of egg content.

In *P. dumerilii* the mitotic asymmetric cell divisions occur rapidly, approximately every 20 to 40 min [19, 25], starting after completion of the meiotic cell divisions at 2 hpf and continuing well beyond the ~330 cell-stage at 14 hpf [19, 25] (Fig. 2d). Therefore, a high maternal contribution of ACD transcripts may establish a requirement for the rapid ACDs during early spiral-cleavage stages within *P. dumerilii*. Indeed, most ACD transcripts are expressed at exceptionally high values including the main Par polarity network transcripts *l(2)gl* and *par4*, spindle orientation transcripts *numa*, *dlg*, and *g*_*αi*_, and polarity complex transcripts *crumbs*, *cora*, *yurt*, *neurexin IV,* and *na/k atpase*. Cell adhesion components such as *pcd11x*, *afadin*, *neuroglian*, *neuroligin*, and *contactin* are all expressed above 170 FPKM at 2 hpf, and are among the 400 highest expressed transcript species in the *P. dumerilii* zygote [56]. Closer investigation revealed that transcripts encoding other cellular machinery implicated in rapid embryonic cell divisions including components of the cell cycle, and β-catenin switch, are also found in this category (Schneider, unpublished observation). The data is consistent with observations that the transcriptional levels for embryonic cyclins are very high in other species (reviewed in [81]). Thus, we suggest that this maternal transcriptional signature of ACDs identifies the *P. dumerilii* egg as a specialized cell that is primed for fast ACD through high expression of ACD components.

### Do the developmental expression profiles reflect actual transcript levels?

It is important to emphasize that the presented developmental expression profiles are measurements of polyadenylated transcripts only, and do not capture transcripts that are not polyadenylated. Indeed, transcriptional data for the unfertilized egg (0 hpf, data not shown), suggests that some transcript species are being polyadenylated shortly after fertilization while others are not. This intriguing phenomenon is being investigated and requires additional approaches. However, this has only a minor effect on the 2 hpf /one-celled zygote data in *P. dumerilii* that we suggest reflect the ‘maternal contribution’ for each transcript most closely. WMISHs to both individual unfertilized (0 hpf) and fertilized eggs (2 hpf) support this view (Fig. 8). Thus, the high maternal transcript levels reported here at 2 hpf should be interpreted as similar or lower than the actual maternal contribution to the egg.

### Fast removal of maternal ACD transcripts

Although the maternal level of most ACD components in the *P. dumerilii* zygote is high, possibly indicating a requirement for fast ACD, the transcript levels for most drop dramatically between 2 hpf and 4 hpf, at a time when the first ACDs are underway. Therefore, the ACD transcript levels decline sharply before most ACDs have occurred in *P. dumerilii.* This shows that lower levels of ACD transcripts are sufficient to support later embryonic ACDs. Lower transcript levels may also indicate that a larger pool of early synthesized ACD proteins exists before the spiral cleavage stages can execute subsequent ACDs in later stages.

Indeed, one can distinguish two groups of ACD transcripts based upon their depletion profile. One group exhibits very steep decline in transcript levels by a factor of >5 between 2 hpf and 4 hpf, and includes *numa*, *pcd11x*, *celsr1*, *magi2*, *neurexin-IV, echinoid, afadin*, and *teneurin.* It is tempting to speculate that the fast removal of transcripts of the first group might point to a specific requirement for their removal e.g. a certain cellular or pattering mechanism may depend on the rapid depletion of these transcript species to ensure that development proceed properly. The second group of high maternal transcripts exhibits a much slower decline during early cleavage stages, and includes all Par polarity network transcripts, *scribble*, spindle orientation dynamic complex components, and several cell recognition components. The slow rate of decline may point to more general or diverse functions required to execute ACDs during early development.

The two described categories for maternal transcript removal may also define different mRNA clearance mechanisms operating in the early *P. dumerilii* embryo [56]. Clearance of maternal transcripts has been found to be a crucial and characteristic mechanism for the maternal-to-zygotic-transition in developing metazoan embryos [82]. However, although clearance mechanisms have been studied to some extent, general patterns based on genome-wide data are just emerging [82]. To our knowledge, our study is the first example that points to transcripts for ACD components as potential targets for mRNA clearance during early embryogenesis.

### Early zygotic transcription of key ACD components

Conspicuously, some key ACD components exhibit early onset of zygotic expression. This implicates a contribution for specific regulatory functions in ACD complexes timed by their onset of expression in *P. dumerilii* embryos. One transcript exhibits high-to-moderate maternal contribution including *lin7*, and others with no or very low maternal contribution including *ndel1* and *four-jointed,* all of which show an increase in transcript level between 2 hpf and 4 hpf. Transcript levels for others increase from 4 hpf to 6 hpf including *par3a*, *ect2, g*_*ai*_ *, ndel1, dynamitin, dynactin p22/p24, magi2*, *neurexin IV,* and *four-jointed. P. dumerilii* embryos, during this time-period, conduct the third and fourth set of ACDs to establish the animal-pole and vegetal-pole cell lineages, including the micromeres 1a-1d, and the macromeres 1A-1D, respectively. These increases in transcript levels coincide with cell polarities and asymmetries that generate the early spiral cleavage pattern. Furthermore, other ACD transcripts increase expression from 6 hpf to 8 hpf including *ect2*, *insc*, *ndel1, afadin, celsr1*, and *magi2*. Thus, several ACD components exhibit early onset of gene expression with the potential to serve as key regulators of ACD functions during early cleavage stages. Interestingly, early zygotic transcription occurs prior to the broad activation of zygotic transcription that has been suggested to represent the maternal-to-zygotic transition in the *P. dumerilii* embryo, between 8 hpf and 10 hpf [56], further implicating the regulatory roles of these genes.

A specific example, for a conspicuous interplay of expression of ACD complex components, is the force-generating machinery implicated in spindle orientation (Fig. 4e). Transcript levels encoding motor proteins of the Dynein/Dynactin complex are high at 2 hpf, reduce at 4 hpf, and increase at 6 hpf (*dynactin complex*), or 8 hpf (*dynein heavy chain*). Three transcript levels for known Dynactin coactivators *lis1, bicd2*, and *aurora a* are high at 2 hpf, steadily decline to 8 hpf, and increase at 10 hpf. By contrast, expression levels for another essential Dynactin cofactor, *ndel1,* is low at 2 hpf followed by a steady increase. Therefore, the observed differences in expression profiles between components of this particular cell machinery, during early embryogenesis, may warrant a targeted investigation of *ndel1* for any potential early role in ACDs.

A second specific example is the Crumbs complex, implicated in establishing cell polarity and the formation of immature adhesive structures (Fig. 5e). The Crumbs complex components including *crumbs, patj,* and *pals1/stardust* all exhibit a similar expression pattern in *P. dumerilii,* including high maternal contribution followed by a steady decrease. Standing out is the early zygotic expression of *lin7* showing a dramatic increase from 100 to 135 FPKM between 2 hpf and 4 hpf. This atypical profile may identify Lin7 as a regulatory factor within the highly conserved Crumbs complex and a worthy target for subsequent studies.

Intriguingly, zygotic transcription has been shown to be required for spindle orientation in some ACDs during early spiral cleavages in the leech embryo [83–85]. General inhibition of zygotic transcription by injection of α-amanitin interfered with distinct unequal cleavages suggesting that one or more zygotic transcripts are required for specific ACDs to occur. No specific zygotic ACD transcript was identified in these pioneering studies, but it is tempting to speculate that the identified early zygotic ACD transcripts in *P. dumerilii* from our study might be excellent candidates.

### Scarce ACD transcripts: Missing ACD components?

Several component that are essential for ACD functions in other model systems [2, 4, 42] including Par network protein Par1, and spindle orientation component Pins, exhibit very low expression levels during early time points in *P. dumerilii* embryos. Transcript levels for *par1* and *pins* do not increase above 3 FPKM during early cleavage stages. Considering that *par1* is present at low levels and has been confirmed by PCR with 2 hpf cDNA (data not shown), we suggest that Par1 has a minor function, is required in low concentrations, could have been replaced by an unknown mechanism, or serves as the rate limiting step. For Pins, with its known pivotal role in linking polarity cues to spindle orientation machinery in *Drosophila* neuroblasts [74], we suggest that *P. dumerilii* may exhibit a Pins independent mechanism as found in *Drosophila* imaginal wing disc epithelium development [86].

Not surprisingly, *P. dumerilii* does not encode a Par2 ortholog, a prominent posterior Par network component in early *C. elegans* embryos. Although we cannot exclude the possibility that highly derived versions exist that escape bioinformatic detection, Par2 has not been identified outside of closely related nematodes. Based on a minor, but overlapping role of Lgl in posterior domain establishment in *C.elegans* [87], and Lgl’s dominant posterior role replacing Par2’s function in *Drosophila* embryos [4, 79, 88, 89], we suggest that the prominent expression of *l(2)gl* may point to a similar replacement scenario in *P. dumerilii*.

### Similarity in expression domains: Co-expression of ACD components

The comparison between the seven stages investigated by RNA-seq in this study provides only limited insights into co-expressed groups of genes based on expression profiles. However, several sets of transcripts display conspicuous similarity in developmental regulation when compared to components within the same complex. These include the Crumbs complex, as discussed above, to a limited extent the anterior (*par3b, par6*) and posterior Par complex (*par4, l(2)gl*), the *dynactin* complex, *cora* and *yurt*, and several components of the PCP pathway including *vangl, fat, dachsous*, and *celsr1*. Other striking examples are *neuroglian* and c*ontactin*, and also *latrophilin* and *teneurin*, both pairs of which have been described as directly interacting binding partners [54, 90]. Therefore, we suggest that the observed similar regulation of gene expression in *P. dumerilii* might suggest similar roles for these co-expressed components within conserved complexes; executing functions that have been previously described in other models.

### Neural cell recognition complexes in early embryogenesis

Interestingly, this study discovered the maternal contribution and co-expression of cell adhesion related genes whose function has previously only been described in the nervous system. These include genes that comprise a potential cell recognition code including several extracellular recognition partners, the co-expressed genes *neuroglian, contactin,* and *neurexin IV* known to work together between myelin sheaths and axons at the paranodal region, and *latrophilin* and its binding partner *teneurin* [54, 90–92]. It is curious that many of these ‘specialized’ cell recognition proteins, with well-described neuronal functions, are expressed with the same characteristic patterns as other ACD components and polarity regulators. In fact, neurons are also extremely polarized and, by design, express proteins in a highly asymmetric fashion between axons, dendrites, and glial cells [54, 93]. This study describes the expression of those same transcripts in an early embryonic context, and it is tempting to speculate that they contribute to potential mechanisms for embryonic cells to orient themselves. Furthermore, the prominent early embryonic expression of components that comprise neuronal recognition systems may suggest an evolutionarily older general embryonic function to facilitate embryonic polarity and adhesion; which were later co-opted and optimized to generate neuronal polarity functions in the evolving brain.

## Conclusion

Our survey for various ACD and adhesion components in *P. dumerilii*, and the establishing of their stage specific expression profiles during early spiral cleavages by RNA-seq enabled us to determine a comprehensive inventory and quantify their presence in early *P. dumerilii* embryos. Remarkably, most components are highly maternally provided suggesting a requirement for this spiralian egg to contain most of the ACD components for the subsequent rapid succession of ACDs in spiralian development. Additionally, comparison of expression profiles of the ACD machinery enabled a systems-level analysis of each contributing module identifying several key regulatory components that are zygotically expressed, and others that are absent. Intriguingly, several cell adhesion modules including the PCP pathway and several neuronal recognition system complexes, are highly maternally provided; also suggesting early embryonic functions in this spiral-cleaving embryo. Therefore, this survey of ACD components may serve as a fertile ground, and starting point, to investigate the particular contribution of each ACD module for their ability to execute the spiral cleavage program, and to generate the accuracy in cell size asymmetries, cell position, and cell fate specification including its link to the global asymmetric β-catenin-mediated cell fate specification module in the *P. dumerilii* embryo.

## Additional files

Additional file 1: Asymmetric Cell Division Genes: Review of the four categories of ACD genes including proposed functions, references, and four supplementary tables (Tables S1–4) that list each gene. Category 1: Cortical Domain Establishment; Category 2: Spindle Orientation; Category 3: Polarity Complexes; Category 4: Cell-cell adhesion and Cell recognition complexes, respectively. (PDF 260 kb)

Additional file 2: FASTA Files for all gene sequences, cloned sequences, and translated sequences. Here we present the gene models for ACD genes in *Platynereis dumerilii* that were used to generate primers for the cloning of gene fragments ~1 kb. All genes were isolated from 2 hpf cDNA and fragments cloned into competent *E. coli*. All cloned fragments were sequenced and verified by aligning the cloned sequence against the reference genome. Included are proposed translated sequences that were translated from ExPASy online translator https://web.expasy.org/translate/ [65]. Translated sequences were subjected to a reciprocal BLASTP (NCBI) to verify conservation of a gene https://blast.ncbi.nlm.nih.gov/Blast.cgi [64]. (DOCX 224 kb)

Additional file 3: Figure S1. Late stage WMISH for ACD genes shown in Fig. 8 as specificity controls at 2-day old *P.dumerilii* larvae. (PDF 6099 kb)

Additional file 4: Figure S2. WMISH negative controls using sense probes for the two most highly expressed ACD genes shown in Fig. 8. Antisense probes were compared to negative control sense probes for *pcd11x* and *l(2)gl* in WMISH. Each probe was used on specimens of early and late stages: 0 hpf, 3 hpf (2-cell stage), and 48 hpf. (PDF 7613 kb)

Additional file 5: RNA-seq data for each ACD gene within the four categories during early development of *P.dumerilii.* Quantitative expression levels are shown as FPKM for each gene at two-hour time points from 2 to 14 hpf (related to Figs. 3-7). Independent measurements for two biological replicates of each embryonic stage and for technical replicates of eight samples are shown. Data can be found on the on Pdumbase http://pdumbase.gdcb.iastate.edu/platynereis/controller.php?action=home [63]. (XLSX 54 kb)

Additional file 6: Gene Ontology Search Query: Cell Polarity Genes. List of all genes and expression profiles for time points 0–14 hpf generated for a GO Search: Cell Polarity (related to Figs. 3-7). Search hits are generated for the Biological Process GO category that contains a Cell Polarity annotation. The table was organized by sorting the 2 hpf FPKM expression values from large-to-small. All values below 1 FPKM at 2 hpf were excluded. The expression profiles and the annotation information based on the BLAST results against the Swiss-Prot database are also included. (XLSX 76 kb)

Additional file 7: Gene Ontology Search Query: Cell Adhesion. List of all genes and expression profiles for time points 0–14 hpf generated for a GO Search: Cell Adhesion (related to Figs. 5-7). Search hits are generated for the Biological Process GO category that contains a Cell Adhesion annotation. The table was organized by sorting the 2 hpf FPKM expression values from large-to-small. All values below 1 FPKM at 2 hpf were excluded. The expression profiles and the annotation information based on the BLAST results against the Swiss-Prot database are also included. (XLSX 135 kb)

Additional file 8: Gene Ontology Search Query: Cell Junction. List of all genes and expression profiles for time points 0–14 hpf generated for a GO Search: Cell Junction (related to Figs. 5-7). Search hits are generated for the Biological Process GO category that contains a Cell Junction annotation. The table was organized by sorting the 2 hpf FPKM expression values from large-to-small. All values below 1 FPKM at 2 hpf were excluded. The expression profiles and the annotation information based on the BLAST results against the Swiss-Prot database are also included. (XLSX 144 kb)

Additional file 9: Category 1. Cortical Domain genes. Individual developmental expression profiles for each ACD gene including standard deviation error bars for all time points are shown. (XLSX 88 kb)

Additional file 10: Category 2. Spindle Orientation genes. Individual developmental expression profiles for each ACD gene including standard deviation error bars for all time points are shown. (XLSX 102 kb)

Additional file 11: Category 3. Polarity Complexes genes. Individual developmental expression profiles for each ACD gene including standard deviation error bars for all time points are shown. (XLSX 121 kb)

Additional file 12: Category 4. Cell-cell Adhesion and cell recognition complexes genes. Individual developmental expression profiles for each ACD gene including standard deviation error bars for all time points are shown. (XLSX 103 kb)

## Abbreviations

A/P: Anterior/posterior
A/V: Animal/vegetal
ACD: Asymmetric cell division
aGPCR: Adhesion GPCR
AJ: Adherens junction
BLAST: Basic Local Alignment Search Tool
cDNA: complementary DNA
CRIB: Protein domain: Cdc42/RAC interactive binding
D/V: Dorsal/ventral
F-actin: filamentous actin
FERM: Protein domain: 4.1 protein, ezrin, radixin, moesin
FLRT: Protein domain: fibronectin leucine-rich transmembrane.
FPKM: Fragments Per Kilobase per Million mapped reads
GAP: GTPase activating protein
GEF: Guanine exchange factor
GO: Gene ontology
GPCR: G-protein coupled receptor
GPI: Protein domain: glycosylphosphatidylinositol
Hpf: Hours post fertilization
Ig: Immunoglobulin
Kb: Kilobase pairs
MT: Microtubules
PAR: Partitioning defective
PB1: Protein domain: phox, bem1
PCP: Planar cell polarity
PCR: Polymerase chain reaction
PDZ: Protein domain: postsynaptic density, discs large, zonula adherens
PIP_2_: Phosphatidyl inositol diphosphate
PIP_3_: Phosphatidyl inositol triphosphate
R/L: Right/left
RNA-Seq: RNA sequencing
Ser/Thr: Serine/Threonine
SJ: Septate junction
TBLASTN: Translated nucleotide BLAST
TM: Transmembrane
TPR: Protein domain: tetratricopeptide repeat
WMISH: Whole-mount in situ hybridization
Wnt: Wingless
