# Supplementary material for: The asymmetric cell division machinery in the spiral-cleaving egg and embryo of the marine annelid Platynereis dumerilii
Source: BMC Dev Biol. 2017 Dec 11;17:16. doi: 10.1186/s12861-017-0158-9 (PMC5725810; doi:10.1186/s12861-017-0158-9)
Supplement: Supplementary file 1 — Asymmetric Cell Division Genes: Review of the four categories of ACD genes including proposed functions, references, and four supplementary tables (Tables S1–4) that list each gene. Category 1: Cortical Domain Establishment; Category 2: Spindle Orientation; Category 3: Polarity Complexes; Category 4: Cell-cell adhesion and Cell recognition complexes, respectively. (PDF 260 kb) [file 12861_2017_158_MOESM1_ESM.pdf]

Additional File 1—Asymmetric Cell Division Genes: Review of the four categories of ACD genes including proposed functions, references, and four supplementary tables (Tables 1-4) that list each gene. Category 1: Cortical Domain Establishment; Category 2: Spindle Orientation; Category 3: Polarity Complexes; Category 4: Cell-cell adhesion and Cell recognition complexes, respectively.

Category 1: Cortical Domain Establishment

Two decades of intensive research starting with the initial discovery of the Par genes as crucial regulators of ACD in early *C. elegans* embryos, has established a mostly conserved, protein interaction network that creates an initial cortical asymmetry of components as a prelude to an ACD. The Par polarity network defines cortical domains in a dividing embryonic cell as anterior and posterior in *C. elegans* and *Drosophila* that are maintained through mutual inhibition (Figure 3a). For a list of candidate genes see Table 1 and Figure 3b, c.

The Par polarity protein network divides the cortex into two opposing domains and includes: anterior Par (aPAR) proteins, and the posterior Par (pPAR proteins)[1–6]. The aPARs form a complex of proteins that includes: Par3 [7–11], Par6 [7,10,12,13], atypical Protein Kinase C (aPKC) [9–11,14,15], the small GTPase Cell division Cycle 42 (Cdc42) [10,13,16], and small effector proteins GTPase Rho (Rho-1) [17], and the Guanine Exchange Factor (GEF) Ect-2 [17]. Par3 contains multiple PDZ domains [8,9,18], is used in several polarity contexts [3], and serves a dynamic role in organizing different forms of polarity throughout early development. Par6 is the central scaffold for the aPAR polarity group that

Table 1. Cortical Domain Proteins

Drosophila Name	Mammalian Name	Function	Reference
aPKC	PKC ι, **ζ**	Maintains domain through repression of L(2)gl and Par1 (Mark1) through phosphorylation	[1,3,9–11,14,15,19]
Bazooka	Par3	Multiple PDZ domain protein that serves as a dynamic regulator of aPars, apical, and lateral complexes	[2,3,8–11,18]
Cdc42	Cdc42	Rho family GTPase that binds Par6 and can associate with the plasma membrane, is required for aPar localization	[3,10,13,16]
Par6	Par6 **α**, **β**, **γ**	Central scaffolding aPar that binds Par3, aPKC, and Cdc42	[2,3,10,13,20]
Pbl	Ect2	Rho family Guanine Exchange Factor that activates Rho-1	[3–5,17]
Rho1	RhoA	GTPase that is a positive regulator of myosin, generally associated with the aPar complex	[3–5,17]
Lgl	Lgl 1,2	Inhibits aPars by binding Par6 and aPKC to form a cytosolic complex, localizes with Par1	[3,21–25]
Par1	Mark1	Ser/Thr kinase, removes Par3 from plasma membrane through phosphorylation, contains lipid binding domain	[1,3,26,27]
Lkb1	Stk11	Ser/Thr kinase, master kinase that can activate Par1, cytoplasmic localization	[2,3,28–30]
Rac GTPase	RacGAP	Negatively regulates Rho-1	[3–5,31]

Table 1: Table of Cortical Domain Proteins List of genes identified through *in silico* searches for Cortical Domain proteins that influence cell polarity (related to Fig. 3). Table includes gene names and general functions as demonstrated in various model organisms.

binds Par3 through its PDZ domain [10–13], aPKC through its PB1 domain [2,9–11], and Cdc42 through its CRIB domain [10,11,13,16,32] (Figure 3a'). aPKC helps to maintain the aPAR domain by repression of pPARs via phosphorylation [19,21,22]. Cdc42 is required for

aPAR localization at the cortex [10,16]. In *C. elegans*, Rho-1 and Ect-2 are associated withthe aPARs [4,17]. Rho-1 is a positive regulator of myosin and is not active at the posterior pole in *C. elegans*. Ect-2 regulates Rho-1 through its GEF interaction [4,17].

The pPAR proteins include: L(2)gl [21–25], the Serine/Threonine Kinase Par1 (Mark1) [26,27,33], Serine/Threonine Kinase Par4 (Lkb1/Stk11) [28–30], and the small effector protein GTPase Activating Factor (GAP) RacGAP [31]. Lgl serves a redundant role in determination of posterior polarity in *C. elegans* [23,25]. The Par protein Par2 works independently to determine posterior polarity in *C. elegans* [7,18,23]. However, Par2 has not been found outside of *C. elegans* [21,25]. In other organisms, including *Drosophila*, L(2)gl is required for the establishment and maintenance of the posterior domain [21,22]. L(2)gl is a scaffolding protein that allows Par1 to accumulate in the posterior domain and inhibit Par3 in the posterior domain via phosphorylation, which removes Par3 from the cortex [26,27]. RacGAP negatively regulates Rho-1, causing Rho-1 to be inhibited at the posterior pole [31]. Therefore, the establishment and maintenance of the two cortical domains in *C. elegans* and *Drosophila* is conducted through mutual repression via reciprocal phosphorylation [1–4,24].

Category 2: Spindle Orientation

As mentioned in the background, we have divided Spindle Orientation proteins into two groups based upon the following criteria: (1) proteins that mediate the transfer of polarity cues from cortical domains to proteins anchored in the plasma membrane, and other proteins that mediate microtubule capture at the plasma membrane orientation complex,

Table 2. Spindle Orientation Proteins

Drosophila Name	Mammalian Name	Function	Reference
Gαi	Gαi	Binds plasma membrane, also binds Pins via Go-loco motifs, helps anchor Pins at cortex	[34–43]
Mud	NuMA	Mediates dynamic interaction binding Pins to Dynactin at the cortex	[35,36,40,41,43–48]
Inscuteable	Inscuteable	Binds aPARs and competes with NuMA to bind TPR repeats, translating polarity cue	[41,46,49–52]
Pins	LGN	Connects Dynein to cortex through dynamic interactions of aPARs, Insc, NuMA, and Dynactin	[35–37,40,41,43,44,46, 53,54]
Disks Large	Dlg 1-4	Mediates MT capture via Khc-73 and cortical targeting through binding of phosphorylated Pins	[37,40,55–58]
Khc-73	KIF13B	Localizes to MT plus ends and binds Dlg	[37,57,58]
Aurora	Aurora A	Phosphorylates Pins to activate MT capture pathway at the cortex	[56,59,60]
BicD	BicD2	Dynein cofactor that activates non-processive dynein to processive through binding cargo and dynein/dynactin complex	[61–63]
Dynactin complex	Dynactin complex	Large multi-subunit interacting protein that binds Dynein and NuMA	[63,64]
Dynein complex	Dynein complex	Minus end directed MT motor protein, binds Dynactin to mediate interactions with NuMA	[65,66]
CLIP-190	CLIP-170	Transports the Dynein/Dynactin complex to the cortex by associating with MT plus ends	[67–69]
Nudel	Ndel1 (NudE)	Promotes recruitment of Dynactin to Dynein through facilitation of Lis1/Dynein interaction	[63,70–73]
Lis1	Lis1	Cofactor of BicD2 that activates Dynein processivity at the cortex	[62,63,70–73]

Table 2: Table of Spindle Orientation Proteins List of genes identified through *in silico* searches for Spindle Orientation proteins that influence mitotic spindle orientations (related to Fig. 4,). Table includes gene names and functions that have been described in various model organisms.

and (2) proteins that constitute, or shuttle, the motor complex to the microtubule plus end and activate the force generating minus end tracking proteins.

The first group includes: Pins [35–37,42,43,46,53,54], Insc [46,50–52,74], the α-subunit of G-protein$_i$ ($G_{\alpha i}$) [34–39,42,43], Aurora A [56,59,60], NuMA [35,36,43–48], Dlg [37,55–58], and Khc-73 [37,57] (Table 2). In order to capture microtubules at the cortex, a dynamic interaction, involving the proteins of group 1 (Figure 4e), is required to orient mitotic spindle microtubules. The scaffolding protein Insc physically links the aPAR complex to Pins [38,46,50–52,54,75] (Figure 4a). The GoLoco domains of Pins binds to $G_{\alpha i}$ to link the orientation complex to the plasma membrane through $G_{\alpha i}$'s association with the plasma membrane [34,37,38,42,51,53,54,76,77] (Figure 4b). Aurora A phosphorylates Pins, in Pins unstructured domain [56], allowing Dlg to bind Pins [37,56,57]. Dlg interacts with the microtubule plus end tracking protein, Khc-73, to capture microtubules at the plasma membrane [37,58] (Figure 4c). Finally, Pins requires a method of interacting with the motor complex. NuMA is recruited to the cortex and outcompetes Insc to bind Pins [35,36,46,48,51,53,54]. NuMA is able to interact with the Dynein/Dynactin complex, the force generating proteins of the motor complex [46,47,78] (Figure 4d).

The second group includes the motor complex and motor protein regulatory proteins: Bicaudal D2 (BicD2) [61–63], Dynactin complex (11 proteins with 23 subunits)[63,64,70], Dynein complex (multiple subunits) [65,66], CLIP-170 [67–69], and includes Dynactin/Dynein cofactors NudEL (Ndel1) [63,70–73] and Lissencephaly-1 (Lis1) [62,63,70–73,79] (Table 2, Figure 4f). The first task is to transport the motor complex,

comprised of the Dynein/Dynactin complex. CLIP-170 (CLIP 190 in *Drosophila*) shuttles the large Dynein/Dynactin complex to the microtubule plus end [63,67,69,80–82]. The Dynein/Dynactin complex is kept relatively inactive during transport and requires cofactors to activate force generation [61,62,70,71,73,79,83]. Bicaudal D2 (BicD2) serves to facilitate the minus-end tracking function of Dynein and is localized at the cortex [62,84]. BicD2 requires two other cofactors to function. Lis1 serves a dynamic role, in conjunction with Ndel1, as a Dynein cofactor [63,70,85]. Ndel1 facilitates loading of Lis1 on to Dynein [63,70–73]. Finally, Dynactin completes the association between the motor complex and cortical attachment as Dynactin binds NuMA [46,47,78] (Figure 4d).

Category 3: Polarity Complexes

One of the essential complexes that establishes and maintains the apical domain in many model systems is the Crumbs complex [86–90]. Curiously, the Crumbs complex, which includes the transmembrane protein Crumbs, and intracellularly associated partners Lin7 [91–93], Partner of Lin-7 (Pals1) (Stardust in *Drosophila*) [94–96], and PatJ [90,97,98], requires a dynamic process that replaces the apical Par complex (aPARs in *C. elegans*) proteins to maintain the apical domain [89,94,99–101] (Figure 5a-c). The central component of this process, in *Drosophila*, is Bazooka (Baz) (*Drosophila* homolog of Par3) [89,101–103]. aPKC phosphorylates Baz, which relaxes the association of Baz with the Par6-aPKC-Cdc42 complex [89,104]. This allows Crumbs, which accumulates at the cortex through recycling endosomes and exocyst activity [105,106], to outcompete Baz for binding to Par6 [89,104]. Cytoplasmic Baz also recruits Stardust (Std) to the cortex [94]. Std then binds to Crumbs at the cortex after aPKC phosphorylates Bazooka to release Std

Table 3. Polarity Complex Proteins

Drosophila Name	Mammalian Name	Function	Reference
Crumbs	Crumbs 1-3	A large transmembrane protein that dynamically replaces the aPAR complex to establish apicobasal polarity	[86–90,101,103]
PatJ	PatJ	Required for stability of the Crumbs complex via binding to Pals1	[90,97,98,101,103]
Stardust	Pals1	Recruited to the cortex via Bazooka, competitively binds Crumbs to stabilize complex, recruits PatJ and Lin7	[94–96,101,103]
Lin7	Lin7	Recruited to the Crumbs complex as a core component	[91–93,101]
PTEN	PTEN	Phosphatase that converts PIP_3 to PIP_2, PIP_2 enrichment is located in the apical domain where it interacts with apically localized proteins for domain maintenance	[103,107,108]
Coracle	EPB41 1-3	Core component of Septate Junctions that stabilize the basolateral domains, Cora binds Neurexin IV (TM protein)	[109–113]
Yurt	EPB41 4-5	Negative regulator of the Crumbs complex via binding of Crumbs cytoplasmic tail, cooperates with Cora to regulate basolateral domain, also binds Neuroglian (TM protein)	[110,113–116]
Scribble	Scribble	Basolateral polarity regulator that influences PCP components, retromer trafficking and Crumbs, and complexes with Lgl and Dlg	[117–123]
Na/K ATPase	Na/K ATPase	Binds Cora and serves as a core component of Septate Junctions	[110,124–126]
Caspr 1,2	Neurexin IV	Core component of Septate Junctions (TM protein), binds Cora through its intracellular domain, cooperates with Yurt to establish and maintain basolateral polarity	[110,127–130]
Neuroglian	Neurofascin	Binds Yurt and forms a component of Septate Junctions, Glial localized binding partner for paranodal complex at Nodes of Ranvier	[131–133]
Numb	Numb	Asymmetrically localized, antagonized by aPKC, and Notch signaling inhibitor	[134–137]
Brat	Trim3	mRNA translational repressor that localizes asymmetrically	[138–142]

Table 3: Table of Polarity Complex Proteins List of genes identified through *in silico* searches for Polarity Complex proteins that influence the establishment of cell polarity through protein complexes usually found in epithelial systems (related to Fig. 5, 6). Table includes gene names and functions that have been shown in various model organisms.

[94,101]. Also, PatJ and Lin-7 are recruited to stabilize the Crumbs complex [91,95,143]. Baz then becomes instrumental in establishing junctional complexes in the lateral membrane [11,89,104,144] (Figure 5d).

Other mechanisms, that serve redundant polarity generating roles, are also present in many model systems. Altering the composition of the inner leaflet of the plasma membrane is one such mechanism [108]. Phospholipid composition directly impacts cytoskeletal dynamics [107]. Enzymes that interact with phosphatidyls is one such mechanism that plays a modulatory role in polarity generating mechanisms [103,108]. Phosphatase and TENsin homolog protein (PTEN), which removes a phosphate group from Phosphatidylinositol (3, 4, 5) triphosphate (PIP_3), converting PIP_3 to Phosphatidylinositol (4, 5) diphosphate (PIP_2) [107,108], has also been shown to directly bind Par3 [107,145]. PTEN increases the concentration of PIP_2 in the apical membrane playing a role in the regulation of actin organization helping to maintain polarity domains and immature AJs [100,107,146,147] (Figure 5a, d).

Opposing the apical domain polarity complexes are the basolateral domain complexes. Scribble, a membrane scaffolding protein [117–123] involved in endocytic pathways [117,148], interacts with Dlg and L(2)gl to reinforce the basolateral domain in epithelial and developmental model systems [149,150] (Figure 6a, b). Dlg is also associated with phosphoinositide kinase-3 (PIK3) to increase PIP_3 concentration in the basolateral domain [151]. This interaction negatively regulates the Crumbs complex and is required for Adherens Junction stability [3,108,151,152]. Although physical interactions have not been

shown within the complex, with the exception of Scribble and Dlg in *Drosophila* synapses [118,123,153], genetic interaction show that mutants lacking any of the three components lead to depletion of the other two [118,123,153], and a loss of basolateral polarity and cell size asymmetries [118,123,153].

A fourth module includes the FERM domain proteins Yurt and Cora that work together to establish the basolateral domain independent of the Scribble/Dlg/L(2)gl complex [109–116] (Figure 6c). Yurt works to maintain domains by antagonizing Crumbs through physical interaction [110,114,115] (Figure 6d). Although the exact mechanisms have yet to be elucidated, Yurt binds both aPKC and Crumbs and reciprocally antagonize each other, with aPKC exerting influence through phosphorylation of Yurt [110,114,115]. Yurt and Cora are also instrumental in establishing the lateral domains and mediating the formation of SJs [109,110,113,154]. Additionally, Cora is localized to SJs, basal to AJs, and is required for SJ stability [109,113,154]. Cora helps to establish a basolateral domain with help from interaction partners Neurexin IV [110,127–130], a transmembrane domain protein capable of cell-cell interactions, the ion exchange pump Na^+K^+ATPase [110,124–126], as well as the Yurt-Neuroglian interaction found in Septate Junctions [131–133].

Category 4: Cell- cell Adhesion and Cell recognition complexes

Previously discussed polarity complexes define the boundaries of the immature Zonula Adherens that, subsequently, promote the formation of AJs and SJs [3,147,155]. A critical part in junction formation, in various model systems, is the establishment and stabilization of transient cell-cell contacts [156–158]. The Classical Cadherin, N-cadherin, a cell-

Table 4. Cell Adhesion Proteins

Drosophila Name	Mammalian Name	Function	Reference
Classical Cadherin	Classical Cadherin	Major component of AJs, typically homophilic adhesion, clusters with Nectins at AJs, can interact with Wnt signaling receptors	[156,159–162]
Echinoid	Hemicentin 1,2	Ig cell adhesion molecule, homophilic adhesion, required for upstream for the formation of primordial AJs	[157,158,163,164]
Canoe	Afadin	Binds Nectin intracellularly and serves as the adapter that connects Nectin to the actin cytoskeleton, required for the formation of AJs	[144,165–169]
Strabismus	Van Gogh Like 1-2 (Vangl)	4 pass TM core PCP component that binds Frizzled, Vangl accumulates proximally and anteriorly, requires Flamingo/Starry Night	[170–179]
Fat	Fat 1-4	Core PCP component that binds Dachsous, accumulates proximally in Ft-Ds gradient signaling	[177,179–186]
Dachsous	Dachsous 1, 2	Core PCP component that binds Fat, accumulates distally in Ft-Ds gradient signaling	[177,179,181–183,185,187]
Flamingo/Starry Night	Celsr 1-3	7 pass TM core PCP component that is required for proper recruitment of Flamingo/Starry Night and Frizzled, is not polarized and exhibits homophilic adhesion	[177–179,188–193]
Four-jointed	Four-jointed box protein 1	Phosphorylates Fat and Dachsous in the Golgi apparatus to regulate PCP	[177,179,182,184,194–197]
Latrophilin (CIRL)	Latrophilin 1-3	Presynaptic adhesion GPCR that binds Teneurins and FLRTs (both are post synaptic)	[198–201]
Teneurin a,m	Teneurin 1-4	Post synaptic TM binding parter of Latrophilin 1	[200,202–207]
Neurexin IV	Caspr 1,2	Core component of Septate Junctions (TM protein), binds Cora through its intracellular domain, cooperates with Yurt to establish and maintain basolateral polarity	[110,127–130]
Neuroglian	Neurofascin	Binds Yurt and forms a component of Septate Junctions, Glial localized binding partner for paranodal complex at Nodes of Ranvier	[131–133]

Table 4 continued

Drosophila Name	Mammalian Name	Function	Reference
Contactin	Contactin	GPI anchored protein that forms part of the paranodal adhesion complex	[133,208–210]
Neuroligin	Neuroligin	Asymmetrically expressed on the post synaptic terminal in neurons, binds classical neurexins, can dimerize (homo or hetero), and clustering has been shown in certain cell types	[211–218]
-	Protocadherin 11x	Negatively regulates dendritic branching in neurons, promotes neuronal stem cell proliferation and suppresses differentiation	[219–223]
Magi	Magi 1-3	Intracellular scaffolding binding partner of Neuroligin at synapses, maintains AJs and apical domain in *Drosophila*	[224–229]

Table 4: Table of Cell Adhesion Proteins List of genes identified through *in silico* searches for Cell Adhesion proteins that influence the establishment and maintenance of cell polarity through cell-cell contact (related to Fig. 7). Table includes gene names and functions that have been shown in various model organisms.

adhesion protein present at AJs [159–162], serves to bind cells together through mostly homophilic interactions [159–161,230].

The protein Echinoid, which functions in *Drosophila*, works in concert with the internal restructuring of domain proteins to recruit and stabilize Classical Cadherins at spot AJs [163]; this role is analogous to Nectin's role in forming AJs in vertebrates [157,158,161,164,168,169]. Whereas both Echinoid and Nectin both trans-interact to adhere to opposing cells [158,163,164], Nectin (vertebrates) first cis-dimerizes (associating with other Nectin proteins on the same cell surface) before trans-interacting [158,164]. Cadherins are then recruited to Echinoid/Nectin contact sites to form the spot

AJs [158,163,164]. Both Echinoid and Nectins are also linked to internal polarity cues to help direct them to the correct lateral domains, particularly through interactions with Bazooka (Par3 in vertebrates), however, the mechanism is unclear [104,144,163] (Figure 5d). Further, Echinoid and Nectins link to the cytoskeleton protein F-actin, the major cytoskeletal component of AJs, through the adaptor protein Canoe/AF-6/Afadin [144,165–169].

Planar Cell Polarity (PCP) proteins define another important cell-adhesion mediated polarity subdivision. The transmembrane proteins Fat [180–186], Dachsous [181–183,185,187], Van-Gogh Like (Vangl) [170–176,178], and Celsr1 [178,188–193] are several of the conserved core of PCP proteins that function to polarize cells along a planar axis [177,179]. Fat and Dachsous contain large extracellular domains and can interact with many intracellular binding partners [186], while Vangl and Celsr1 have smaller extracellular domains, and serve to organize protein complexes that help to determine planar polarity [177,179]. The core PCP proteins function as a group to establish polarity [177,179]. A mutation with Vangl, Celsr1, or several of their intracellular binding partners causes total loss of planar polarity [177,231]. PCP proteins are also integrated into the Wnt signaling system through both the non-canonical and canonical pathways to mediate cell fate decisions [178,179].

In *Drosophila*, Four-jointed (Fj) functions to regulate Fat and Dachsous through phosphorylation of extracellular cadherin repeat domains [177,182,184,194–197]. During *Drosophila* wing development, a gradient of Fj is essential to cause Dachsous to accumulate

on a lateral subcellular location opposite of Fat [177,196,197]. This asymmetry is patterned along the Fj gradient with high Fj levels in the distal wing and low in the proximal wing [177,197]. Correspondingly, Dachsous accumulates on the distal side of each cell and Fat on the proximal side of each cell [177,197]. In this manner, a grouping of cells becomes polarized within a plane.

Our third subdivision of cell adhesion mediated polarity contains two groups of proteins that are generally considered to be nervous system specific and have prominent functions within the nervous system. The first group includes Neuroglian [131–133], Neurexin-IV/Caspr [127–130], and Contactin [133,208–210], with roles in mediating axon-axon and axon-glial interactions in many model systems [128–130,132,133,208–210,232]. Neuroglian binds Neurexin-IV/Caspr at the paranodal region of Nodes of Ranvier to mediate very specific boundaries in the segregation of potassium and sodium voltage gated ion channels [128,130,132,209,232]. This interaction requires the glycan phosphatidyl inositol (GPI)-anchored protein Contactin in *Drosophila* and vertebrates [209]. As discussed in the Polarity complex section, Neurexin-IV has been shown in other systems to be present in a basolateral complex with Cora [110,127–130], while Neuroglian has been shown to interact with the basolateral protein Yurt [131–133]. Additionally, the Neuroglian/Neurexin-IV/Contactin group also comprise components of SJs, along with Cora and Dlg [110,127,129–132]. As SJs form basal to AJs, it is not surprising that basolateral complex proteins Cora and Dlg also localize with this group in other model systems [109,110,113,132,154].

The second group of cell-adhesion mediated polarity proteins (in the third subdivision), that mediate polarized interactions in the nervous system, consists of Latrophilin 1 [198–201] and its binding partner Teneurin [200,202–204,206,207] (Figure 7c). Both proteins are typically associated with establishing specific neuronal connectivity in the developing nervous system in many model systems [198–201,203,204,206,207]. Significantly, Latrophilin 1 is classified as an adhesion G-protein coupled receptor (aGPCR). aGPCRs initiate intracellular signaling cascades upon binding with their respective ligands. The Latrophilin 1/Teneurin interaction includes a Calcium signaling cascade with mechanisms still being elucidated [201].

A fourth subdivision of proteins was constituted to showcase proteins that have been characterized in an asymmetric neuronal function, but do not fit with any of the previous cell-adhesion mediated polarity subgroups. These proteins include the dendritic branching regulator Protocadherin 11x (Pcd11x) [219–223], the post-synaptic protein Neuroligin 4Y [211–218], and the synaptic scaffolding protein Magi2 that can interact with Neuroligin 4Y intracellularly [224–229].

Supplementary References

1. Goldstein B, Macara IG. The PAR Proteins: Fundamental Players in Animal Cell Polarization. Dev. Cell. 2007;13:609–22.
2. Macara IG. Parsing the polarity code. Nat. Rev. Mol. Cell Biol. 2004;5:220–31.
3. St Johnston D, Ahringer J. Cell polarity in eggs and epithelia: Parallels and diversity. Cell. 2010. p. 757–74.
4. Rose L, Gonczy P. Polarity establishment, asymmetric division and segregation of fate determinants in early C. elegans embryos. WormBook. 2014;1–43.
5. Iden S, Collard JG. Crosstalk between small GTPases and polarity proteins in cell

polarization. Nat. Rev. Mol. Cell Biol. 2008;9:846–59.
6. Goehring NW, Hoege C, Grill SW, Hyman AA. PAR proteins diffuse freely across the anterior-posterior boundary in polarized C. elegans embryos. J. Cell Biol. 2011;193:583–94.
7. Cheng NN, Kirby CM, Kemphues KJ. Control of cleavage spindle orientation in Caenorhabditis elegans: The role of the genes par-2 and par-3. Genetics. 1995;139:549–59.
8. Kuchinke U, Grawe F, Knust E. Control of spindle orientation in Drosophila by the Par-3-related PDZ-domain protein Bazooka. Curr. Biol. 1998;8:1357–65.
9. Suzuki A, Yamanaka T, Hirose T, Manabe N, Mizuno K, Shimizu M, et al. Atypical protein kinase C is involved in the evolutionarily conserved par protein complex and plays a critical role in establishing epithelia- specific junctional structures. J. Cell Biol. 2001;152:1183–96.
10. Joberty G, Petersen C, Gao L, Macara IG. The cell-polarity protein Par6 links Par3 and atypical protein kinase C to Cdc42. Nat. Cell Biol. 2000;2:531–9.
11. Yamanaka T, Horikoshi Y, Suzuki A, Sugiyama Y, Kitamura K, Maniwa R, et al. PAR-6 regulates aPKC activity in a novel way and mediates cell-cell contact-induces formation of the epithelial junctional complex. Genes to Cells. 2001;6:721–31.
12. Petronczki M, Knoblich JA. DmPAR-6 directs epithelial polarity and asymmetric cell division of neuroblasts in Drosophila. Nat. Cell Biol. 2001;3:43–9.
13. Johansson A, Driessens M, Aspenström P. The mammalian homologue of the Caenorhabditis elegans polarity protein PAR-6 is a binding partner for the Rho GTPases Cdc42 and Rac1. J. Cell Sci. 2000;113:3267–75.
14. Wodarz A, Ramrath A, Grimm A, Knust E, Wodarz A, Ramrath A, et al. Drosophila Atypical Protein Kinase C Associates with Bazooka and Controls Polarity of Epithelia and Neuroblasts. J. Cell Biol. 2000;150:1361–74.
15. Izumi Y, Hirose T, Tamai Y, Hirai S, Nagashima Y, Fujimoto T, et al. An Atypical PKC Directly Associates and Colocalizes at the Epithelial Tight Junction with ASIP, a Mammalian Homologue of Caenorhabditis elegans Polarity Protein PAR-3. J. Cell Biol. 1998;143:95–106.
16. Hutterer A, Betschinger J, Petronczki M, Knoblich JA. Sequential roles of Cdc42, Par-6, aPKC, and Lgl in the establishment of epithelial polarity during Drosophila embryogenesis. Dev. Cell. 2004;6:845–54.
17. Motegi F, Sugimoto A. Sequential functioning of the ECT-2 RhoGEF, RHO-1 and CDC-42 establishes cell polarity in Caenorhabditis elegans embryos. Nat. Cell Biol. 2006;8:978–85.
18. Etemad-Moghadam B, Guo S, Kemphues KJ. Asymmetrically distributed PAR-3 protein contributes to cell polarity and spindle alignment in early C. elegans embryos. Cell. 1995;83:743–52.
19. Rolls MM, Albertson R, Shih HP, Lee CY, Doe CQ. Drosophila aPKC regulates cell polarity and cell proliferation in neuroblasts and epithelia. J. Cell Biol. 2003;163:1089–98.
20. Kemphues K. PARsing embryonic polarity. Cell. 2000;101:345–8.
21. Betschinger J, Mechtler K, Knoblich JJA. The Par complex directs asymmetric cell division by phosphorylating the cytoskeletal protein Lgl. Nature. 2003;422:326–30.
22. Plant PJ, Fawcett JP, Lin DCC, Holdorf AD, Binns K, Kulkarni S, et al. A polarity complex of mPar-6 and atypical PKC binds, phosphorylates and regulates mammalian Lgl. Nat. Cell Biol. 2003;5:301–8.
23. Hoege C, Constantinescu AT, Schwager A, Goehring NW, Kumar P, Hyman AA. LGL can partition the cortex of one-cell caenorhabditis elegans embryos into two domains. Curr. Biol. Elsevier Ltd; 2010;20:1296–303.

24. Hoege C, Hyman AA. Principles of PAR polarity in Caenorhabditis elegans embryos. Nat Rev Mol Cell Biol. 2013;14:315–22.
25. Beatty A, Morton D, Kemphues KJ. The C. elegans homolog of Drosophila Lethal giant larvae functions redundantly with PAR-2 to maintain polarity in the early embryo. Development. 2010;4004:3995–4004.
26. Benton R, St Johnston D. Drosophila PAR-1 and 14-3-3 inhibit Bazooka/PAR-3 to establish complementary cortical domains in polarized cells. Cell. 2003;115:691–704.
27. Drewes G, Ebneth A, Preuss U, Mandelkow EM, Mandelkow E. MARK, a novel family of protein kinases that phosphorylate microtubule-associated proteins and trigger microtubule disruption. Cell. 1997;89:297–308.
28. Martin SG, St Johnstone D. A role for Drosophila LKB1 in anterior-posterior axis formation and epithelial polarity. Nature. 2003;421:379–84.
29. Lizcano JM, Göransson O, Toth R, Deak M, Morrice NA, Boudeau J, et al. LKB1 is a master kinase that activates 13 kinases of the AMPK subfamily, including MARK/PAR-1. EMBO J. 2004;23:833–43.
30. Watts JL, Morton DG, Bestman J, Kemphues KJ. The C. elegans par-4 gene encodes a putative serine-threonine kinase required for establishing embryonic asymmetry. Development. 2000;127:1467–75.
31. Jenkins N, Saam JR, Mango SE. CYK-4/GAP Provides a Localized Cue to Initiate Anteroposterior Polarity upon Fertilization. Science (80-.). 2006;313:1298–301.
32. Garrard SM, Capaldo CT, Gao L, Rosen MK, Macara IG, Tomchick DR. Structure of Cdc42 in a complex with the GTPase-binding domain of the cell polarity protein, Par6. EMBO J. 2003;22:1125–33.
33. Kemphues KJ, Priess JR, Morton DG, Cheng NS. Identification of genes required for cytoplasmic localization in early C. elegans embryos. Cell. 1988;52:311–20.
34. Schaefer M, Petronczki M, Dorner D, Forte M, Knoblich JA. Heterotrimeric G proteins direct two modes of asymmetric cell division in the Drosophila nervous system. Cell. 2001;107:183–94.
35. Siller KH, Cabernard C, Doe CQ. The NuMA-related Mud protein binds Pins and regulates spindle orientation in Drosophila neuroblasts. Nat. Cell Biol. 2006;8:594–600.
36. Bowman SK, Neumüller RA, Novatchkova M, Du Q, Knoblich JA. The Drosophila NuMA Homolog Mud Regulates Spindle Orientation in Asymmetric Cell Division. Dev. Cell. 2006;10:731–42.
37. Siegrist SE, Doe CQ. Microtubule-induced pins/Gai cortical polarity in Drosophila neuroblasts. Cell. 2005;123:1323–35.
38. Williams SE, Ratliff LA, Postiglione MP, Knoblich JA, Fuchs E. Par3-mInsc and Gαi3 cooperate to promote oriented epidermal cell divisions through LGN. Nat. Cell Biol. 2014;16:758–69.
39. Willard FS, Kimple RJ, Siderovski DP. Return of the GDI: the GoLoco motif in cell division. Annu. Rev. Biochem. 2004;73:925–51.
40. Siller KH, Doe CQ. Spindle orientation during asymmetric cell division. Nat. Cell Biol. 2009;11:365–74.
41. Gönczy P. Mechanisms of asymmetric cell division: flies and worms pave the way. Nat. Rev. Mol. Cell Biol. 2008;9:355–66.
42. Gotta M, Ahringer J. Distinct roles for Galpha and Gbetagamma in regulating spindle position and orientation in Caenorhabditis elegans embryos. Nat. Cell Biol. 2001;3:297–

300.
43. Colombo K, Grill SW, Kimple RJ, Willard FS, Siderovski DP, Gönczy P. Translation of polarity cues into asymmetric spindle positioning in Caenorhabditis elegans embryos. Science. 2003;300:1957–61.
44. Guan Z, Prado A, Melzig J, Heisenberg M, Nash HA, Raabe T. Mushroom body defect, a gene involved in the control of neuroblast proliferation in Drosophila, encodes a coiled-coil protein. Proc. Natl. Acad. Sci. U. S. A. 2000;97:8122–7.
45. Lydersen BK, Pettijohn DE. Human-specific nuclear protein that associates with the polar region of the mitotic apparatus: distribution in a human/hamster hybrid cell. Cell. 1980;22:489–99.
46. Lechler T, Fuchs E. Asymmetric cell divisions promote stratification and differentiation of mammalian skin. Nature. 2005;437:275–80.
47. Radulescu AE, Cleveland DW. NuMA after 30 years: The matrix revisited. Trends Cell Biol. Elsevier Ltd; 2010. p. 214–22.
48. Srinivasan DG, Fisk RM, Xu H, Van den Heuvel S. A complex of LIN-5 and GPR proteins regulates G protein signaling and spindle function in C. elegans. Genes Dev. 2003;17:1225–39.
49. Kraut R, Chia W, Jan LY, Jan YN, Knoblich JA. Role of inscuteable in orienting asymmetric cell divisions in Drosophila. Nature. 1996. p. 50–5.
50. Schaefer M, Shevchenko a, Shevchenko a, Knoblich J a. A protein complex containing Inscuteable and the Galpha-binding protein Pins orients asymmetric cell divisions in Drosophila. Curr. Biol. 2000;10:353–62.
51. Zhu J, Wen W, Zheng Z, Shang Y, Wei Z, Xiao Z, et al. LGN/mInsc and LGN/NuMA Complex Structures Suggest Distinct Functions in Asymmetric Cell Division for the Par3/mInsc/LGN and Gai/LGN/NuMA Pathways. Mol. Cell. Elsevier Inc.; 2011;43:418–31.
52. Žigman M, Cayouette M, Charalambous C, Schleiffer A, Hoeller O, Dunican D, et al. Mammalian inscuteable regulates spindle orientation and cell fate in the developing retina. Neuron. 2005;48:539–45.
53. Du Q, Macara IG. Mammalian Pins is a conformational switch that links NuMA to heterotrimeric G proteins. Cell. 2004;119:503–16.
54. Du Q, Stukenberg PT, Macara IG. A mammalian Partner of inscuteable binds NuMA and regulates mitotic spindle organization. Nat. Cell Biol. 2001;3:1069–75.
55. Bergstralh DT, Lovegrove HE, St Johnston D. Discs large links spindle orientation to apical-basal polarity in drosophila epithelia. Curr. Biol. Elsevier; 2013;23:1707–12.
56. Johnston CA, Hirono K, Prehoda KE, Doe CQ. Identification of an Aurora-A/PinsLINKER/Dlg Spindle Orientation Pathway using Induced Cell Polarity in S2 Cells. Cell. 2009;138:1150–63.
57. Hanada T, Lin L, Tibaldi E V., Reinherz EL, Chishti AH. GAKIN, a novel kinesin-like protein associates with the human homologue of the Drosophila Discs large tumor suppressor in T lymphocytes. J. Biol. Chem. 2000;275:28774–84.
58. Lu MS, Johnston CA. Molecular pathways regulating mitotic spindle orientation in animal cells. Development. 2013;140:1843–56.
59. Lee CY, Andersen RO, Cabernard C, Manning L, Tran KD, Lanskey MJ, et al. Drosophila Aurora-A kinase inhibits neuroblast self-renewal by regulating aPKC/Numb cortical polarity and spindle orientation. Genes Dev. 2006;20:3464–74.
60. Gallini S, Carminati M, De Mattia F, Pirovano L, Martini E, Oldani A, et al. NuMA

phosphorylation by aurora-a orchestrates spindle orientation. Curr. Biol. Elsevier Ltd; 2016;26:458–69.
61. Claußen M, Suter B. BicD-dependent localization processes: From Drosophilia development to human cell biology. Ann. Anat. 2005;187:539–53.
62. Splinter D, Razafsky DS, Schlager MA, Serra-Marques A, Grigoriev I, Demmers J, et al. BICD2, dynactin, and LIS1 cooperate in regulating dynein recruitment to cellular structures. Mol. Biol. Cell. 2012;23:4226–41.
63. Kardon JR, Vale RD. Regulators of the cytoplasmic dynein motor. Nat. Rev. Mol. Cell Biol. Nature Publishing Group; 2009;10:854–65.
64. Urnavicius L, Zhang K, Diamant AG, Motz C, Schlager MA, Yu M, et al. The structure of the dynactin complex and its interaction with dynein. Science (80-.). 2015;347:1441–6.
65. Roberts AJ, Kon T, Knight PJ, Sutoh K, Burgess SA. Functions and mechanics of dynein motor proteins. Nat. Rev. Mol. Cell Biol. Nature Publishing Group; 2013;14:713–26.
66. Vallee RB, McKenney RJ, Ori-McKenney KM. Multiple modes of cytoplasmic dynein regulation. Nat. Cell Biol. Nature Publishing Group; 2012;14:224–30.
67. Dzhindzhev NS, Rogers SL, Vale RD, Ohkura H. Distinct mechanisms govern the localisation of Drosophila CLIP-190 to unattached kinetochores and microtubule plus-ends. J. Cell Sci. 2005;118:3781–90.
68. Perez F, Diamantopoulos GS, Stalder R, Kreis TE. CLIP-170 Highlights Growing Microtubule Ends In Vivo. Cell. 1999;96:517–27.
69. Galjart N. CLIPs and CLASPs and cellular dynamics. Nat. Rev. Mol. Cell Biol. 2005;6:487–98.
70. Wang S, Ketcham SA, Schön A, Goodman B, Wang Y, Bement W, et al. Nudel/NudE and Lis1 promote dynein and dynactin interaction in the context of spindle morphogenesis. Mol. Biol. Cell. 2013;24:3522–33.
71. Sasaki S, Shionoya A, Ishida M, Gambello MJ, Yingling J, Wynshaw-Boris A, et al. A LIS1/NUDEL/cytoplasmic dynein heavy chain complex in the developing and adult nervous system. Neuron. 2000;28:681–96.
72. Niethammer M, Smith DS, Ayala R, Peng J, Ko J, Lee MS, et al. NUDEL is a novel Cdk5 substrate that associates with LIS1 and cytoplasmic dynein. Neuron. 2000;28:697–711.
73. Feng Y, Olson EC, Stukenberg PT, Flanagan LA, Kirschner MW, Walsh CA. LIS1 regulates CNS lamination by interacting with mNudE, a central component of the centrosome. Neuron. 2000;28:665–79.
74. Kraut R, Campos-Ortega JA. inscuteable,A Neural Precursor Gene ofDrosophila,Encodes a Candidate for a Cytoskeleton Adaptor Protein. Dev. Biol. 1996;174:65–81.
75. Izaki T, Kamakura S, Kohjima M, Sumimoto H. Two forms of human Inscuteable-related protein that links Par3 to the Pins homologues LGN and AGS3. Biochem. Biophys. Res. Commun. 2006;341:1001–6.
76. Gotta M, Dong Y, Peterson YK, Lanier SM, Ahringer J. Asymmetrically Distributed C. elegans Homologs of AGS3/PINS Control Spindle Position in the Early Embryo. Curr. Biol. 2003;13:1029–37.
77. Nipper RW, Siller KH, Smith NR, Doe CQ, Prehoda KE. Galphai generates multiple Pins activation states to link cortical polarity and spindle orientation in Drosophila neuroblasts. Proc. Natl. Acad. Sci. U. S. A. 2007;104:14306–11.
78. Merdes A, Ramyar K, Vechio JD, Cleveland DW. A complex of NuMA and cytoplasmic dynein is essential for mitotic spindle assembly. Cell. 1996;87:447–58.

79. Dix CI, Soundararajan HC, Dzhindzhev NS, Begum F, Suter B, Ohkura H, et al. Lissencephaly-1 promotes the recruitment of dynein and dynactin to transported mRNAs. J. Cell Biol. 2013;202:479–94.
80. Carvalho P, Gupta ML, Hoyt MA, Pellman D. Cell cycle control of kinesin-mediated transport of Bik1 (CLIP-170) regulates microtubule stability and dynein activation. Dev. Cell. 2004. p. 815–29.
81. Miller RK, D'Silva S, Moore JK, Goodson H V. The CLIP-170 Orthologue Bik1p and Positioning the Mitotic Spindle in Yeast. Curr. Top. Dev. Biol. 2006. p. 49–87.
82. Howard J, Hyman AA. Dynamics and mechanics of the microtubule plus end. Nature. 2003;422:753–8.
83. Sheeman B, Carvalho P, Sagot I, Geiser J, Kho D, Hoyt MA, et al. Determinants of S. cerevisiae dynein localization and activation: Implications for the mechanism of spindle positioning. Curr. Biol. 2003;13:364–72.
84. Jha R, Surrey T. Regulation of processive motion and microtubule localization of cytoplasmic dynein. Biochem. Soc. Trans. 2015;43:48–57.
85. Liang Y, Yu W, Li Y, Yang Z, Yan X, Huang Q, et al. Nudel functions in membrane traffic mainly through association with Lis1 and cytoplasmic dynein. J. Cell Biol. 2004;164:557–66.
86. Tepass U, Theres C, Knust E. crumbs encodes an EGF-like protein expressed on apical membranes of Drosophila epithelial cells and required for organization of epithelia. Cell. 1990;61:787–99.
87. Tepass U. Crumbs, a component of the apical membrane, is required for zonula adherens formation in primary epithelia of Drosophila. Dev. Biol. 1996;177:217–25.
88. Bachmann a, Schneider M, Theilenberg E, Grawe F, Knust E. Drosophila Stardust is a partner of Crumbs in the control of epithelial cell polarity. Nature. 2001;414:638–43.
89. Walther RF, Pichaud F. Crumbs/DaPKC-dependent apical exclusion of bazooka promotes photoreceptor polarity remodeling. Curr. Biol. Elsevier Ltd; 2010;20:1065–74.
90. Lemmers C, Médina E, Delgrossi MH, Michel D, Arsanto JP, Le Bivic A. hINAd1/PATJ, a homolog of discs lost, interacts with crumbs and localizes to tight junctions in human epithelial cells. J. Biol. Chem. 2002;277:25408–15.
91. Bachmann A, Timmer M, Sierralta J, Pietrini G, Gundelfinger ED, Knust E, et al. Cell type-specific recruitment of Drosophila Lin-7 to distinct MAGUK-based protein complexes defines novel roles for Sdt and Dlg-S97. J. Cell Sci. 2004;117:1899–909.
92. Irie M, Hata Y, Deguchi M, Ide N, Hirao K, Yao I, et al. Isolation and characterization of mammalian homologues of Caenorhabditis elegans lin-7: localization at cell-cell junctions. Oncogene. 1999;18:2811–7.
93. Jo K, Derin R, Li M, Bredt DS. Characterization of MALS/Velis-1, -2, and -3: a family of mammalian LIN-7 homologs enriched at brain synapses in association with the postsynaptic density-95/NMDA receptor postsynaptic complex. J. Neurosci. 1999;19:4189–99.
94. Krahn MP, Bückers J, Kastrup L, Wodarz A. Formation of a Bazooka-Stardust complex is essential for plasma membrane polarity in epithelia. J. Cell Biol. 2010;190:751–60.
95. Roh MH, Makarova O, Liu CJ, Shin K, Lee S, Laurinec S, et al. The Maguk protein, Pals1, functions as an adapter, linking mammalian homologues of crumbs and discs lost. J. Cell Biol. 2002;157:161–72.
96. Kamberov E, Makarova O, Roh M, Liu A, Karnak D, Straight S, et al. Molecular cloning

and characterization of Pals, proteins associated with mLin-7. J. Biol. Chem. 2000;275:11425–31.
97. Bhat MA, Izaddoost S, Lu Y, Cho K-O, Choi K-W, Bellen HJ. Discs Lost, a Novel Multi-PDZ Domain Protein, Establishes and Maintains Epithelial Polarity. Cell. 1999;96:833–45.
98. Nam SC, Choi KW. Domain-specific early and late function of Dpatj in Drosophila photoreceptor cells. Dev. Dyn. 2006;235:1501–7.
99. Chen J, Zhang M. The Par3/Par6/aPKC complex and epithelial cell polarity. Exp. Cell Res. Elsevier; 2013. p. 1357–64.
100. Horikoshi Y, Suzuki A, Yamanaka T, Sasaki K, Mizuno K, Sawada H, et al. Interaction between PAR-3 and the aPKC-PAR-6 complex is indispensable for apical domain development of epithelial cells. J. Cell Sci. 2009;122:1595–606.
101. Laprise P, Tepass U. Novel insights into epithelial polarity proteins in Drosophila. Trends Cell Biol. Elsevier Ltd; 2011;21:401–8.
102. Bulgakova NA, Knust E. The Crumbs complex: from epithelial-cell polarity to retinal degeneration. J. Cell Sci. 2009;122:2587–96.
103. Bryant DM, Mostov KE. From cells to organs: building polarized tissue. Nat. Rev. Mol. Cell Biol. 2008;9:887–901.
104. Morais-de-Sá E, Mirouse V, St Johnston D. aPKC Phosphorylation of Bazooka Defines the Apical/Lateral Border in Drosophila Epithelial Cells. Cell. Elsevier Ltd; 2010;141:509–23.
105. Blankenship JT, Fuller MT, Zallen J a. The Drosophila homolog of the Exo84 exocyst subunit promotes apical epithelial identity. J. Cell Sci. 2007;120:3099–110.
106. Roeth JF, Sawyer JK, Wilner DA, Peifer M. Rab11 helps maintain apical crumbs and adherens junctions in the drosophila embryonic ectoderm. PLoS One. 2009;4.
107. von Stein W, Ramrath A, Grimm A, Müller-Borg M, Wodarz A. Direct association of Bazooka/PAR-3 with the lipid phosphatase PTEN reveals a link between the PAR/aPKC complex and phosphoinositide signaling. Development. 2005;132:1675–86.
108. Martin-Belmonte F, Gassama A, Datta A, Yu W, Rescher U, Gerke V, et al. PTEN-Mediated Apical Segregation of Phosphoinositides Controls Epithelial Morphogenesis through Cdc42. Cell. 2007;128:383–97.
109. Ward IV RE, Schweizer L, Lamb RS, Fehon RG. The protein 4.1, ezrin, radixin, moesin (FERM) domain of drosophila coracle, a cytoplasmic component of the septate junction, provides functions essential for embryonic development and imaginal cell proliferation. Genetics. 2001;159:219–28.
110. Laprise P, Lau KM, Harris KP, Silva-Gagliardi NF, Paul SM, Beronja S, et al. Yurt, Coracle, Neurexin IV and the Na(+),K(+)-ATPase form a novel group of epithelial polarity proteins. Nature. Nature Publishing Group; 2009;459:1141–5.
111. Fehon RG, Dawson I a, Artavanis-Tsakonas S. A Drosophila homologue of membrane-skeleton protein 4.1 is associated with septate junctions and is encoded by the coracle gene. Development. 1994;120:545–57.
112. Marfatia SM, Lue RA, Branton D, Chishti AH. Identification of the protein 4.1 binding interface on glycophorin C and p55, a homologue of the Drosophila discs-large tumor suppressor protein. J. Biol. Chem. 1995. p. 715–9.
113. Tepass U. FERM proteins in animal morphogenesis. Curr. Opin. Genet. Dev. 2009. p. 357–67.
114. Laprise P, Beronja S, Silva-Gagliardi NF, Pellikka M, Jensen AM, McGlade CJ, et al. The

FERM Protein Yurt Is a Negative Regulatory Component of the Crumbs Complex that Controls Epithelial Polarity and Apical Membrane Size. Dev. Cell. 2006;11:363–74.
115. Gamblin CL, Hardy ÉJ-L, Chartier FJ-M, Bisson N, Laprise P. A bidirectional antagonism between aPKC and Yurt regulates epithelial cell polarity. J. Cell Biol. 2014;204:487–95.
116. Gosens I, Sessa A, den Hollander AI, Letteboer SJF, Belloni V, Arends ML, et al. FERM protein EPB41L5 is a novel member of the mammalian CRB-MPP5 polarity complex. Exp. Cell Res. 2007;313:3959–70.
117. de Vreede G, Schoenfeld JD, Windler SL, Morrison H, Lu H, Bilder D. The Scribble module regulates retromer-dependent endocytic trafficking during epithelial polarization. Development. 2014;141:2796–802.
118. Bilder D, Li M, Perrimon N. Cooperative regulation of cell polarity and growth by Drosophila tumor suppressors. Science. 2000;289:113–6.
119. Yoshihara K, Ikenouchi J, Izumi Y, Akashi M, Tsukita S, Furuse M. Phosphorylation state regulates the localization of Scribble at adherens junctions and its association with E-cadherin-catenin complexes. Exp. Cell Res. Elsevier Inc.; 2011;317:413–22.
120. Murdoch JN, Henderson DJ, Doudney K, Gaston-Massuet C, Phillips HM, Paternotte C, et al. Disruption of scribble (Scrb1) causes severe neural tube defects in the circletail mouse. Hum. Mol. Genet. 2003;12:87–98.
121. Elsum I, Yates L, Humbert PO, Richardson HE. The Scribble-Dlg-Lgl polarity module in development and cancer: from flies to man. Essays Biochem. 2012;53:141–68.
122. Assémat E, Bazellières E, Pallesi-Pocachard E, Le Bivic A, Massey-Harroche D. Polarity complex proteins. Biochim. Biophys. Acta - Biomembr. 2008. p. 614–30.
123. Su WH, Mruk DD, Wong EWP, Lui WY, Cheng CY. Polarity protein complex scribble/lgl/dlg and epithelial cell barriers. Adv. Exp. Med. Biol. 2013;763:149–70.
124. Paul SM, Ternet M, Salvaterra PM, Beitel GJ. The Na+/K+ ATPase is required for septate junction function and epithelial tube-size control in the Drosophila tracheal system. Development. 2003;130:4963–74.
125. Krupinski T, Beitel GJ. Unexpected roles of the Na-K-ATPase and other ion transporters in cell junctions and tubulogenesis. Physiology (Bethesda). 2009;24:192–201.
126. Rajasekaran S a, Palmer LG, Moon SY, Peralta Soler a, Apodaca GL, Harper JF, et al. Na,K-ATPase activity is required for formation of tight junctions, desmosomes, and induction of polarity in epithelial cells. Mol. Biol. Cell. 2001;12:3717–32.
127. Baumgartner S, Littleton JT, Broadie K, Bhat MA, Harbecke R, Lengyel JA, et al. A Drosophila neurexin is required for septate junction and blood-nerve barrier formation and function. Cell. 1996;87:1059–68.
128. Einheber S, Zanazzi G, Ching W, Scherer S, Milner TA, Peles E, et al. The axonal membrane protein Caspr, a homologue of neurexin IV, is a component of the septate-like paranodal junctions that assemble during myelination. J. Cell Biol. 1997;139:1495–506.
129. Gollan L, Sabanay H, Poliak S, Berglund EO, Ranscht B, Peles E. Retention of a cell adhesion complex at the paranodal junction requires the cytoplasmic region of Caspr. J. Cell Biol. 2002;157:1247–56.
130. Bellen HJ, Lu Y, Beckstead R, Bhat M. Neurexin IV, caspr and paranodin—novel members of the neurexin family: encounters of axons and glia. Trends Neurosci. 1998;21:444–9.
131. Genova JL, Fehon RG. Neuroglian, Gliotactin, and the Na+/k+ ATPase are essential for septate junction function in Drosophila. J. Cell Biol. 2003;161:979–89.

132. Poliak S, Peles E. The local differentiation of myelinated axons at nodes of Ranvier. Nat. Rev. Neurosci. 2003;4:968–80.
133. Charles P, Tait S, Faivre-Sarrailh C, Barbin G, Gunn-Moore F, Denisenko-Nehrbass N, et al. Neurofascin is a glial receptor for the paranodin/Caspr-contactin axonal complex at the axoglial junction. Curr. Biol. 2002;12:217–20.
134. Rhyu MS, Jan LY, Jan YN. Asymmetric distribution of numb protein during division of the sensory organ precursor cell confers distinct fates to daughter cells. Cell. 1994;76:477–91.
135. Zhong W, Feder JN, Jiang MM, Jan LY, Jan YN, Artavanis-Tsakonas S, et al. Asymmetric localization of a mammalian numb homolog during mouse cortical neurogenesis. Neuron. 1996;17:43–53.
136. Shen Q, Zhong W, Jan YN, Temple S. Asymmetric Numb distribution is critical for asymmetric cell division of mouse cerebral cortical stem cells and neuroblasts. Development. 2002;129:4843–53.
137. Yan B. Numb - From flies to humans. Brain Dev. Elsevier B.V.; 2010;32:293–8.
138. Sonoda J, Wharton RP. Drosophila Brain Tumor is a translational repressor Drosophila Brain Tumor is a translational repressor. 2001;762–73.
139. Betschinger J, Mechtler K, Knoblich JA. Asymmetric Segregation of the Tumor Suppressor Brat Regulates Self-Renewal in Drosophila Neural Stem Cells. Cell. 2006;124:1241–53.
140. Lee CY, Wilkinson BD, Siegrist SE, Wharton RP, Doe CQ. Brat is a Miranda cargo protein that promotes neuronal differentiation and inhibits neuroblast self-renewal. Dev. Cell. 2006;10:441–9.
141. Chen G, Kong J, Tucker-Burden C, Anand M, Rong Y, Rahman F, et al. Human brat ortholog TRIM3 is a tumor suppressor that regulates asymmetric cell division in glioblastoma. Cancer Res. 2014;74:4536–48.
142. Hyenne V, Desrosiers M, Labbé JC. C. elegans Brat homologs regulate PAR protein-dependent polarity and asymmetric cell division. Dev. Biol. 2008;321:368–78.
143. Bulgakova N a, Kempkens O, Knust E. Multiple domains of Stardust differentially mediate localisation of the Crumbs-Stardust complex during photoreceptor development in Drosophila. J. Cell Sci. 2008;121:2018–26.
144. Ooshio T, Fujita N, Yamada A, Sato T, Kitagawa Y, Okamoto R, et al. Cooperative roles of Par-3 and afadin in the formation of adherens and tight junctions. J. Cell Sci. 2007;120:2352–65.
145. McKinley RF a., Yu CG, Harris TJC. Assembly of Bazooka polarity landmarks through a multifaceted membrane-association mechanism. J. Cell Sci. 2012;125:1177–90.
146. Bezanilla M, Gladfelter AS, Kovar DR, Lee W-L. Cytoskeletal dynamics: A view from the membrane. J. Cell Biol. 2015;209:329–37.
147. Rodriguez-Boulan E, Macara IG. Organization and execution of the epithelial polarity programme. Nat. Rev. Mol. Cell Biol. 2014;15:225–42.
148. Román-Fernández A, Bryant DM. Complex Polarity: Building Multicellular Tissues Through Apical Membrane Traffic. Traffic. 2016;17:1244–61.
149. Yamanaka T, Ohno S. Role of Lgl/Dlg/Scribble in the regulation of epithelial junction, polarity and growth. Front. Biosci. 2008;13:6693–707.
150. Qi HS, Liu SM, Li S, Wei ZJ. Molecular expression of the scribble complex genes, Dlg, Scrib and Lgl, in silkworm, Bombyx mori. Genes (Basel). 2013;4:264–74.

151. Laprise P, Viel A, Rivard N. Human homolog of disc-large is required for adherens junction assembly and differentiation of human intestinal epithelial cells. J. Biol. Chem. 2004;279:10157–66.
152. Martin-Belmonte F, Perez-Moreno M. Epithelial cell polarity, stem cells and cancer. Nat. Rev. Cancer. Nature Publishing Group; 2012;12:23–38.
153. Mathew D, Gramates LS, Packard M, Thomas U, Bilder D, Perrimon N, et al. Recruitment of Scribble to the synaptic scaffolding complex requires GUK-holder, a novel DLG binding protein. Curr. Biol. 2002;12:531–9.
154. Lamb RS, Ward RE, Schweizer L, Fehon RG. Drosophila coracle, a member of the protein 4.1 superfamily, has essential structural functions in the septate junctions and developmental functions in embryonic and adult epithelial cells. Mol. Biol. Cell. 1998;9:3505–19.
155. Tepass U, Hartenstein V. The development of cellular junctions in the Drosophila embryo. Dev. Biol. 1994. p. 563–96.
156. McGill MA, McKinley RFA, Harris TJC. Independent cadherin-catenin and Bazooka clusters interact to assemble adherens junctions. J. Cell Biol. 2009;185:787–96.
157. Takai Y, Nakanishi H. Nectin and afadin: novel organizers of intercellular junctions. J. Cell Sci. 2003;116:17–27.
158. Takai Y, Miyoshi J, Ikeda W, Ogita H. Nectins and nectin-like molecules: roles in contact inhibition of cell movement and proliferation. Nat. Rev. Mol. Cell Biol. 2008;9:603–15.
159. Iwai Y, Usui T, Hirano S, Steward R, Takeichi M, Uemura T. Axon Patterning Requires D N-cadherin, a Novel Neuronal Adhesion Receptor, in the Drosophila Embryonic CNS. Neuron. 1997;19:77–89.
160. Hatta K, Takeichi M. Expression of N-cadherin adhesion molecules associated with early morphogenetic events in chick development. Nature. 1986;320:447–9.
161. Takeichi M. The cadherin superfamily in neuronal connections and interactions. Nat. Rev. Neurosci. 2007;8:11–20.
162. Shan W, Tanaka H, Phillips GR, Arndt K, Yoshida M, Colman DR, et al. Functional cis-Heterodimers of N- and R-Cadherins. J. Cell Biol. 2000;148:579–90.
163. Wei SY, Escudero LM, Yu F, Chang LH, Chen LY, Ho YH, et al. Echinoid is a component of adherens junctions that cooperates with DE-cadherin to mediate cell adhesion. Dev. Cell. 2005;8:493–504.
164. Takai Y, Irie K, Shimizu K, Sakisaka T, Ikeda W. Nectins and nectin-like molecules: Roles in cell adhesion, migration, and polarization. Cancer Sci. 2003;94:655–67.
165. Matsuo T, Takahashi K, Suzuki E, Yamamoto D. The Canoe protein is necessary in adherens junctions for development of ommatidial architecture in the Drosophila compound eye. Cell Tissue Res. 1999;298:397–404.
166. Ikeda W, Nakanishi H, Miyoshi J, Mandai K, Ishizaki H, Tanaka M, et al. Afadin: A key molecule essential for structural organization of cell- cell junctions of polarized epithelia during embryogenesis. J. Cell Biol. 1999;146:1117–31.
167. Mandai K, Nakanishi H, Satoh A, Obaishi H, Wada M, Nishioka H, et al. Afadin: A Novel Actin Filament-binding Protein with One PDZ Domain Localized at Cadherin-base Cell-to-Cell Adherens Junction. J. Cell Biol. 1997;139:517–28.
168. Takahashi K, Nakanishi H, Miyahara M, Mandai K, Satoh K, Satoh A, et al. Nectin / PRR : An Immunoglobulin-like Cell Adhesion Molecule Recruited PDZ Domain – containing Protein. 1999;145:539–49.

169. Tachibana K, Nakanishi H, Mandai K, Ozaki K, Ikeda W, Yamamoto Y, et al. Two cell adhesion molecules, nectin and cadherin, interact through their cytoplasmic domain-associated proteins. J. Cell Biol. 2000;150:1161–75.
170. Wolff T, Rubin G. Strabismus, a novel gene that regulates tissue polarity and cell fate decisions in Drosophila. Development. 1998;125:1149–59.
171. Taylor J, Abramova N, Charlton J, Adler PN. Van Gogh: A new Drosophila tissue polarity gene. Genetics. 1998;150:199–210.
172. Bastock R, Strutt H, Strutt D. Strabismus is asymmetrically localised and binds to Prickle and Dishevelled during Drosophila planar polarity patterning. Development. 2003;130:3007–14.
173. Wu J, Mlodzik M. The Frizzled Extracellular Domain Is a Ligand for Van Gogh/Stbm during Nonautonomous Planar Cell Polarity Signaling. Dev. Cell. 2008;15:462–9.
174. Park M, Moon RT. The planar cell-polarity gene stbm regulates cell behaviour and cell fate in vertebrate embryos. Nat. Cell Biol. 2002;4:20–5.
175. Kibar Z, Vogan KJ, Groulx N, Justice MJ, Underhill DA, Gros P. Ltap, a mammalian homolog of Drosophila Strabismus/Van Gogh, is altered in the mouse neural tube mutant Loop-tail. Nat. Genet. 2001;28:251–5.
176. Murdoch JN, Doudney K, Paternotte C, Copp AJ, Stanier P. Severe neural tube defects in the loop-tail mouse result from mutation of Lpp1, a novel gene involved in floor plate specification. Hum. Mol. Genet. 2001;10:2593–601.
177. Goodrich L V, Strutt D. Principles of planar polarity in animal development. Development. 2011;138:1877–92.
178. Sokol SY. Spatial and temporal aspects of Wnt signaling and planar cell polarity during vertebrate embryonic development. Semin. Cell Dev. Biol. Elsevier Ltd; 2015;42:78–85.
179. Wallingford JB. Planar Cell Polarity and the Developmental Control of Cell Behavior in Vertebrate Embryos. Annu. Rev. Cell Dev. Biol. 2012;28:627–53.
180. Yang CH, Axelrod JD, Simon MA. Regulation of Frizzled by Fat-like cadherins during planar polarity signaling in the Drosophila compound eye. Cell. 2002;108:675–88.
181. Casal J, Lawrence P a, Struhl G. Two separate molecular systems, Dachsous/Fat and Starry night/Frizzled, act independently to confer planar cell polarity. Development. 2006;133:4561–72.
182. Brittle A, Thomas C, Strutt D. Planar polarity specification through asymmetric subcellular localization of fat and dachsous. Curr. Biol. Elsevier; 2012;22:907–14.
183. Matakatsu H, Blair SS. Interactions between Fat and Dachsous and the regulation of planar cell polarity in the Drosophila wing. Development. 2004;131:3785–94.
184. Saburi S, Hester I, Fischer E, Pontoglio M, Eremina V, Gessler M, et al. Loss of Fat4 disrupts PCP signaling and oriented cell division and leads to cystic kidney disease. Nat. Genet. 2008;40:1010–5.
185. Mao Y, Mulvaney J, Zakaria S, Yu T, Morgan KM, Allen S, et al. Characterization of a Dchs1 mutant mouse reveals requirements for Dchs1-Fat4 signaling during mammalian development. Development. 2011;138:947–57.
186. Matis M, Axelrod JD. Regulation of PCP by the fat signaling pathway. Genes Dev. 2013. p. 2207–20.
187. Adler PN, Charlton J, Liu J. Mutations in the cadherin superfamily member gene dachsous cause a tissue polarity phenotype by altering frizzled signaling. Development. 1998;125:959–68.

188. Chae J, Kim MJ, Goo JH, Collier S, Gubb D, Charlton J, et al. The Drosophila tissue polarity gene starry night encodes a member of the protocadherin family. Development. 1999;126:5421–9.
189. Usui T, Shima Y, Shimada Y, Hirano S, Burgess RW, Schwarz TL, et al. Flamingo, a seven-pass transmembrane cadherin, regulates planar cell polarity under the control of Frizzled. Cell. 1999;98:585–95.
190. Chen WS, Antic D, Matis M, Logan CY, Povelones M, Anderson GA, et al. Asymmetric Homotypic Interactions of the Atypical Cadherin Flamingo Mediate Intercellular Polarity Signaling. Cell. 2008;133:1093–105.
191. Curtin JA, Quint E, Tsipouri V, Arkell RM, Cattanach B, Copp AJ, et al. Mutation of Celsr1 Disrupts Planar Polarity of Inner Ear Hair Cells and Causes Severe Neural Tube Defects in the Mouse. Curr. Biol. 2003;13:1129–33.
192. Formstone CJ, Little PFR. The flamingo-related mouse Celsr family (Celsr1-3) genes exhibit distinct patterns of expression during embryonic development. Mech. Dev. 2001;109:91–4.
193. Tissir F, De-Backer O, Goffinet AM, Lambert de Rouvroit C. Developmental expression profiles of Celsr (Flamingo) genes in the mouse. Mech. Dev. 2002;112:157–60.
194. Strutt H, Mundy J, Hofstra K, Strutt D. Cleavage and secretion is not required for Four-jointed function in Drosophila patterning. Development. 2004;131:881–90.
195. Zeidler MP, Perrimon N, Strutt DI. The four-jointed gene is required in the Drosophila eye for ommatidial polarity specification. Curr. Biol. 1999;9:1363–72.
196. Ishikawa HO, Takeuchi H, Haltiwanger RS, Irvine KD. Four-jointed is a Golgi kinase that phosphorylates a subset of cadherin domains. Science. 2008;321:401–4.
197. Hale R, Brittle AL, Fisher KH, Monk NAM, Strutt D. Cellular interpretation of the long-range gradient of Four-jointed activity in the Drosophila wing. Elife. 2015;2015:1–21.
198. Scholz N, Gehring J, Guan C, Ljaschenko D, Fischer R, Lakshmanan V, et al. The Adhesion GPCR Latrophilin/CIRL Shapes Mechanosensation. Cell Rep. The Authors; 2015;11:866–74.
199. Boucard AA, Maxeiner S, Südhof TC. Latrophilins function as heterophilic cell-adhesion molecules by binding to teneurins: Regulation by alternative splicing. J. Biol. Chem. 2014;289:387–402.
200. Woelfle R, D'Aquila AL, Pavlović T, Husić M, Lovejoy DA. Ancient interaction between the teneurin C-terminal associated peptides (TCAP) and latrophilin ligand-receptor coupling: A role in behavior. Front. Neurosci. 2015. p. 1–10.
201. Silva J, Ushkaryov YA. The latrophilins, "split-personality" receptors. Adv. Exp. Med. Biol. 2010;706:59–75.
202. Baumgartner S, Chiquet-Ehrismann R. Tena, a Drosophila gene related to tenascin, shows selective transcript localization. Mech. Dev. 1993;40:165–76.
203. Baumgartner S, Martin D, Hagios C, Chiquet-Ehrismann R. Tenm, a Drosophila gene related to tenascin, is a new pair-rule gene. EMBO J. 1994;13:3728–40.
204. Levine A, Bashan-Ahrend A, Budai-Hadrian O, Gartenberg D, Menasherow S, Wides R. odd Oz: A novel Drosophila pair rule gene. Cell. 1994;77:587–98.
205. Tucker RP, Drabikowski K, Hess JF, Ferralli J, Chiquet-Ehrismann R, Adams JC. Phylogenetic analysis of the tenascin gene family: evidence of origin early in the chordate lineage. BMC Evol. Biol. 2006;6:60.
206. Oohashi T, Zhou XH, Feng K, Richter B, Mörgelin M, Perez MT, et al. Mouse ten-m/odz

is a new family of dimeric type II transmembrane proteins expressed in many tissues. J. Cell Biol. 1999;145:563–77.
207. Rubin BP, Tucker RP, Martin D, Chiquet-Ehrismann R. Teneurins: a novel family of neuronal cell surface proteins in vertebrates, homologous to the Drosophila pair-rule gene product Ten-m. Dev. Biol. 1999;216:195–209.
208. Banerjee S, Sousa AD, Bhat MA. Organization and function of septate junctions: an evolutionary perspective. Cell Biochem. Biophys. 2006;46:65–77.
209. Labasque M, Faivre-Sarrailh C. GPI-anchored proteins at the node of Ranvier. FEBS Lett. Federation of European Biochemical Societies; 2010. p. 1787–92.
210. Faivre-Sarrailh C, Banerjee S, Li J, Hortsch M, Laval M, Bhat M a. Drosophila contactin, a homolog of vertebrate contactin, is required for septate junction organization and paracellular barrier function. Development. 2004;131:4931–42.
211. Banovic D, Khorramshahi O, Owald D, Wichmann C, Riedt T, Fouquet W, et al. Drosophila Neuroligin 1 Promotes Growth and Postsynaptic Differentiation at Glutamatergic Neuromuscular Junctions. Neuron. Elsevier Ltd; 2010;66:724–38.
212. Sun M, Xing G, Yuan L, Gan G, Knight D, With SI, et al. Neuroligin 2 is required for synapse development and function at the Drosophila neuromuscular junction. J. Neurosci. 2011;31:687–99.
213. Dean C, Scholl FG, Choih J, DeMaria S, Berger J, Isacoff E, et al. Neurexin mediates the assembly of presynaptic terminals. Nat. Neurosci. 2003;6:708–16.
214. Graf ER, Zhang X, Jin SX, Linhoff MW, Craig AM. Neurexins induce differentiation of GABA and glutamate postsynaptic specializations via neuroligins. Cell. 2004;119:1013–26.
215. Jamain S, Quach H, Betancur C, Råstam M, Colineaux C, Gillberg IC, et al. Mutations of the X-linked genes encoding neuroligins NLGN3 and NLGN4 are associated with autism. Nat. Genet. 2003;34:27–9.
216. Bemben MA, Shipman SL, Nicoll RA, Roche KW. The cellular and molecular landscape of neuroligins. Trends Neurosci. Elsevier Ltd; 2015. p. 496–505.
217. Dean C, Dresbach T. Neuroligins and neurexins: Linking cell adhesion, synapse formation and cognitive function. Trends Neurosci. 2006. p. 21–9.
218. Li Y, Zhou Z, Zhang X, Tong H, Li P, Zhang ZC, et al. Drosophila neuroligin 4 regulates sleep through modulating GABA transmission. J Neurosci. 2013;33:15545–54.
219. Zhang P, Wu C, Liu N, Niu L, Yan Z, Feng Y, et al. Protocadherin 11 x regulates differentiation and proliferation of neural stem cell in vitro and in vivo. J. Mol. Neurosci. 2014;54:199–210.
220. Priddle TH, Crow TJ. Protocadherin 11X/Y a human-specific gene pair: An immunohistochemical survey of fetal and adult brains. Cereb. Cortex. 2013;23:1933–41.
221. Wu C, Niu L, Yan Z, Wang C, Liu N, Dai Y, et al. Pcdh11x Negatively Regulates Dendritic Branching. J. Mol. Neurosci. 2015;56:822–8.
222. Frank M, Kemler R. Classification and genomic organization of protocadherins. Curr. Opin. Cell Biol. 2002;14:557–62.
223. Morishita H, Yagi T. Protocadherin family: diversity, structure, and function. Curr. Opin. Cell Biol. 2007;19:584–92.
224. Zaessinger S, Zhou Y, Bray SJ, Tapon N, Djiane a. Drosophila MAGI interacts with RASSF8 to regulate E-Cadherin-based adherens junctions in the developing eye. Development. 2015;1102–12.
225. Barmchi MP, Samarasekera G, Gilbert M, Auld VJ, Zhang B. Magi is associated with the

par complex and functions antagonistically with bazooka to regulate the apical polarity complex. PLoS One. 2016;11:1–24.
226. Hirao K, Hata Y, Ide N, Takeuchi M, Irie M, Yao I, et al. A Novel Multiple PDZ Domain-containing Molecule Interacting with N-Methyl-d-aspartateReceptors and Neuronal Cell Adhesion Proteins. J. Biol. Chem. 1998;273:21105–10.
227. Nagashima S, Kodaka M, Iwasa H, Hata Y. MAGI2/S-SCAM outside brain. J. Biochem. 2015. p. 177–84.
228. Lynch AM, Grana T, Cox-Paulson E, Couthier A, Cameron M, Chin-Sang I, et al. A Genome-wide functional screen shows MAGI-1 Is an L1CAM-Dependent Stabilizer of Apical Junctions in C. Elegans. Curr. Biol. Elsevier Ltd; 2012;22:1891–9.
229. Stetak A, Hajnal A. The C. elegans MAGI-1 protein is a novel component of cell junctions that is required for junctional compartmentalization. Dev. Biol. Elsevier Inc.; 2011;350:24–31.
230. Katsamba P, Carroll K, Ahlsen G, Bahna F, Vendome J, Posy S, et al. Linking molecular affinity and cellular specificity in cadherin-mediated adhesion. Proc Natl Acad Sci U S A. 2009;106:11594–9.
231. Wong LL, Adler P. Tissue Polarity Genes of. J. Cell Biol. 1993;123:209–21.
232. Girault JA, Oguievetskaia K, Carnaud M, Denisenko-Nehrbass N, Goutebroze L. Transmembrane scaffolding proteins in the formation and stability of nodes of Ranvier. Biol. Cell. 2003. p. 447–52.
