# Supplementary material for: The asymmetric cell division machinery in the spiral-cleaving egg and embryo of the marine annelid Platynereis dumerilii
Source: BMC Dev Biol. 2017 Dec 11;17:16. doi: 10.1186/s12861-017-0158-9 (PMC5725810; doi:10.1186/s12861-017-0158-9)
Supplement: Supplementary file 2 — FASTA Files for all gene sequences, cloned sequences, and translated sequences. Here we present the gene models for ACD genes in Platynereis dumerilii that were used to generate primers for the cloning of gene fragments ~1 kb. All genes were isolated from 2 hpf cDNA and fragments cloned into competent E. coli. All cloned fragments were sequenced and verified by aligning the cloned sequence against the reference genome. Included are proposed translated sequences that were translated from ExPASy online translator https://web.expasy.org/translate/ [65]. Translated sequences were subjected to a reciprocal BLASTP (NCBI) to verify conservation of a gene https://blast.ncbi.nlm.nih.gov/Blast.cgi [64]. (DOCX 224 kb) [file 12861_2017_158_MOESM2_ESM.docx]

**aPKC**

Gene model: 4408 nucleotides

ORF : 499 – 2226 = 1728 nucleotides

>apkc_full

ATGGAGTTTTTGAATGTCTTACCATCTTCAGAATCGTCCAAGAAAGTTCAAGATTAAACGAGGAAGCTCTTCTGACTTGCCGGCAATATGGCGGCGGATCCGGAGTGAGCAAAACTTCCACCAATCAGAGGCCGTGTTTGTCGATTTCCTTATATGGTCATTTTGTAAATGTGAGAAACAGTTCGTGCCATAATTTATTATTTCATTTTCATCAATGAAAGTCACGAACATTTTATGCAAAATTGGTTTTGGGGTTTCTATCAAAAAATAAATTCGGCGAGGCGTGCCGGTGATGAAATGAAACACGCACTCTCATTGGCTGAATCCATCCACATGACTCTACCAAGTCCAAGATGGAGAACTGGTAGAAAGTTGGTCACAAATTGGTGTCAAACTTTATTGATCGATTGAGTCTGTTAATCATCTCAAGACTTGTTTTTAGTCGTAATTCGTTCATTGCTCATATCCTTGGCTCTAGAAGAAATTTCAGAAACAAGGATGCCTTCTCAACAGAAGATAGGTGAAAATGAAAAAGTTATTAGACTCAAAGTTGTCTATGGCGGGGATGTCATGGTCACGAATGTTGATTGTGACATCACATTTGAAGGACTGTGTGCAGAAGTGCGCGACATTTGTCGATTTGGAGAGGAGCAGCCTTTTACCTTAAAGTGGCTTGATGAAGAAGGTGATCCCTGCACGATTTCATCCCAGCTGGAATTAGATGAAGCAGTGAGACTCCACGAAATCAACAAGGATACAGAGATCGTCATCCATGTGTTCCCCAACATACCAGAAAAACCTGGACTACCAAACATCGGCGAAGACCGTAATATGTACAGGAGGGGTGCCAGGAGGTGGAGAAAACTGTACAGGGTCAACGGTCATTTATTCCAAGCCAAAAGATTCTCTCGGAGAGCAGTGTGCGCCTATTGCACGGACCGAATCTGGGGTTTAGGTCGCCAGGGGTACAAATGCATCAACTGCAAATTACTCGTTCACAAAAAATGTCACAAACTCATCAAATACGCCTGTGGTGCTAACGTTGATAACCTGCCAGTCCCAGAAATCGACGAACAAAATGGAAATCCATCCAGAAGTTCAGGACGGGCGAGGAATGACACTTCCCACTCATCCCAGTCTTCTTCAAATAATCAGTCAAGTTCGAGTGAAAACGCTCTGATTGGCTTAAGCGATTTTGAACTCTTGCGCGTGATTGGTCGAGGAAGTTATGCCAAAGTTCTAATGGTGGAACAGAAAAGAACGAAACGAATCTACGCCATGAAAGTCATCAAGAAGGAAATCATGAACGATGATGAGGACATTGACTGGGTCCAAACGGAAAAGCATGTCTTTGAGATCGCCTCCAATCACCCCTTCCTCGTCGGACTCCATTCTTGTTTCCAGACGCCCAGCAGATTATTCTTTGTGATAGAGTTTGTCAACGGTGGCGACCTCATGTTTCATATGCAGAGGCAACGGAAACTCCCGGAGGAACACGCTCGCTTCTACGCTGCTGAAATCTGTCTCGCTCTCAACTTCCTTCATGAGAAAGGAATAATTTACAGAGATCTGAAACTGGACAATGTACTGCTCGACTCCGAAGGTCACATAAAGTTAACAGATTATGGAATGTGCAAGGAAGGGCTTCGAACAGGCGACACAACGAGTACATTTTGCGGAACGCCAAACTACATCGCTCCAGAAATGTTACGTGGAGAAGAATATGCTTTCAGTGTCGACTGGTGGGCGTTAGGAGTCCTGATGTTCGAGATGTTGGCCGGGAGATCGCCCTTTGACATTGTCGGACAGACGGATAACCCAGATCAGAACACGGAGGACTACCTCTTCCAAGTTATTTTGGAGAAGACCATTCGAATCCCTCGATCGCTTTCCGTGAAAGCTGCTGCTGTCTTGAAAGGATTCCTCAATAAGAACCCTGACGAGCGTCTTGGTTGCCATCCTCAAACCGGATTCTCGGACATCCAAGGTCACCCATTTTTCCGTAGCATCGACTGGGATCTACTTTACGAGAAGCAGATTACTCCACCCTACAAGCCAACTGTCAAGTCGGACCGAGATCTGGAACATTTCGATCCTCAGTTCACCAACGAGCCTGTGGAGTTGACGCCCGATGATTCGAAAGTCATCTCGAAGATCGACCAGTCTGAATTTGATGGATTCGAGTACATTAATCCTCTTCTTATGTCGCAAGAAGACTGCGTGTAGACGCAAAAATAATCATCGTCAAATTTTTGTCTTTCCTGTTAATATTCACTGCATGAGGCTTATATATATAATCTGTACAGTATATAGAAATATATATATCAAATATATATTGAAAGTGTATATTGAAAATGTATATTGACATTGTATATGGAAAATATGTGAAAGTATTTAGAAAATATGTGTTGATTGGAAGATTTACAGAGCATACGACGTGTAAAAAAGTTGTATGCTTTTTAGGGTTTAGCAAAAATGGCGAAGTACTTTAAAAAATGAATTGAAACATATACAATTCGAAGGACCTGAAACTCAAATATATATGATCACAAATGACATTAAAATATATATGAAAATGACATTAAAATATATATGATCACAAATGACATTAACAGGGGCCATCTTAACTTGCATGGAAATGACAAGAAATAAGGCACGGATGTTCATGGTTAATTATCTACAATTTATTTGGATGAATTGGTTTCTATGTGAATTTTCTATGGCCTTTATTATTATAATATATCTTGAAGATTTGAACGTTATACTCACAACGAAAAATAAAGTAGATGAATAGCTTTCGGAGCTCATCCAAGCTCCTTCTTCAGTTCTTATGTTTGAAATTTGAGCGGTCACGTGACGGTGTTGATGACGTGGTGTCACGTGACGCTAGTATCTCCCGGTATATTGCCGGGAGGGTGTGCCTCCCCCTGTCTCTATTATTATAATATAGTTATTGATAATTGAGCCAAACATTGAAAACCATTAAATTGAATGGTTATGGCATTTGTCGAAAGTTCAAAAACATGCAAGTTTTCCAATAGATAGAAATGATATATTTTATTGCCGAATTGTATTACTTCCTATCGATACCATTTTACTTTTATTCGAGTAACGAAATATGTTGTAATGATTGAATATTTCAAGTTTTTCAACATGAATTTGTGTGTGAAATACTGGTAATTTTGTGTACCAAAGATTTGTATATACACACAACAGCGTATTTGTAAGACGCACATTTAGGAGTGCTCCATTACATTTCGTTTACTGCAGTTGGCGACATATTTAGTTGAAAATGGCGTAATCGTATCTGATCAGTGTGTATAAGTATGATATATGCCAAATGTAAGCATCTTTAAATGTGCTCTTTTATTAGAATCAATGTGAGCTCAAAACTTCTAAGTGTCTAAAGAAATTAAGTGATATTAAATTTCTACTGCCAAGTGGTCATCAGTGTATTGTGCAAAATTAGAAAATTAGATGGAAATCAAATTTCTCGATTGACGCGTTCATGTGCCGAAGCTTGACAAAAGTAAAATGTGATGATGTATAGAATATTTTATGTTACAGTTGAGTAAGCAAAATACTATAATTATATTCCTCATTAGGCACGCCCTGCTCAGAAACAGTGACTGGGTGCACCCATGAATATATTTAAAACAAATATAATGACATCGAAACAGTTACAAATGCTTCGAAGTAAAAAAAAAAAGTTCACATTAAATTGTAGAAAGCCTGTCAATATAATTGAAATTTTCACTGAATACCTGCCTACTTAAACTCTTTAATTGTACTAAGTTTCACCTTCGTACGATACCTACTATTAAAATATCAACAAAAATGTGACCCGCGTTGAAGTGCGAAAGGCAGTTAAGATAGTGCGTGCCTAAAGGGAATATGGTAACGGTATATACATGTACCTTCAACTTTTGGTTACAGCTCTGTAACATTATGTAATGCAACTTTGAAAGTTTTAAGAGTCCTTGATATCGAATTTATTCATGTCAAATGGTTGACAGTGTGATAATGAGATTTTCACTATTCATCTATCTGTTAAAATGTCCTGTCAAATGTCAAGTCTTTCAAAATTGAGGATAAGTAATCTTTATATCGTAGGAAAGTAATTTCTTAATTGACTCTTATGCATGAAATAGGCAATGAATGGCAATTAGGCGATTAAGAGTCGTTGTAACTAATGAGTGATCGCAGAACTTTATTAATTTGATTACTTGCCGTATTTCCTCGAATATAAGCCCGGGCTTCTATTATTTTAGAGACGTAGATACTCCGGCGTTTGTACGAGGCCCGACGTGTAATTGAGACCTGCATCTGTTATAATTTATTTTAATGTAGGCCCCTACCCAGGCCTCTAAACGAGAGAGGCTTTTATTTGAGACCAGGCTTCTATTCG

Protein: 575 aa

>apkc_protein

MPSQQKIGENEKVIRLKVVYGGDVMVTNVDCDITFEGLCAEVRDICRFGEEQPFTLKWLDEEGDPCTISSQLELDEAVRLHEINKDTEIVIHVFPNIPEKPGLPNIGEDRNMYRRGARRWRKLYRVNGHLFQAKRFSRRAVCAYCTDRIWGLGRQGYKCINCKLLVHKKCHKLIKYACGANVDNLPVPEIDEQNGNPSRSSGRARNDTSHSSQSSSNNQSSSSENALIGLSDFELLRVIGRGSYAKVLMVEQKRTKRIYAMKVIKKEIMNDDEDIDWVQTEKHVFEIASNHPFLVGLHSCFQTPSRLFFVIEFVNGGDLMFHMQRQRKLPEEHARFYAAEICLALNFLHEKGIIYRDLKLDNVLLDSEGHIKLTDYGMCKEGLRTGDTTSTFCGTPNYIAPEMLRGEEYAFSVDWWALGVLMFEMLAGRSPFDIVGQTDNPDQNTEDYLFQVILEKTIRIPRSLSVKAAAVLKGFLNKNPDERLGCHPQTGFSDIQGHPFFRSIDWDLLYEKQITPPYKPTVKSDRDLEHFDPQFTNEPVELTPDDSKVISKIDQSEFDGFEYINPLLMSQEDCV

Domains

PB1: 14 - 96

C1: 129 - 181

STKc: 237 - 564

Clone

Partial ORF

Primers

Forward: GGATGTCATGGTCACGAATG

Reverse: CACCAGTCGACACTGAAAGC

Nucleotide# 564 – 1745 = 1182 nucleotides

>apkc_clone

GGATGTCATGGTCACGAATGTTGATTGTGACATCACATTTGAAGGACTGTGTGCAGAAGTGCGCGACATTTGTCGATTTGGAGAGGAGCAGCCTTTTACCTTAAAGTGGCTTGATGAAGAAGGTGATCCCTGCACGATTTCATCCCAGCTGGAATTAGATGAAGCAGTGAGACTCCACGAAATCAACAAGGATACAGAGATCGTCATCCATGTGTTCCCCAACATACCAGAAAAACCTGGACTACCAAACATCGGCGAAGACCGTAATATGTACAGGAGGGGTGCCAGGAGGTGGAGAAAACTGTACAGGGTCAACGGTCATTTATTCCAAGCCAAAAGATTCTCTCGGAGAGCAGTGTGCGCCTATTGCACGGACCGAATCTGGGGTTTAGGTCGCCAGGGGTACAAATGCATCAACTGCAAATTACTCGTTCACAAAAAATGTCACAAACTCATCAAATACGCCTGTGGTGCTAACGTTGATAACCTGCCAGTCCCAGAAATCGACGAACAAAATGGAAATCCATCCAGAAGTTCAGGACGGGCGAGGAATGACACTTCCCACTCATCCCAGTCTTCTTCAAATAATCAGTCAAGTTCGAGTGAAAACGCTCTGATTGGCTTAAGCGATTTTGAACTCTTGCGCGTGATTGGTCGAGGAAGTTATGCCAAAGTTCTAATGGTGGAACAGAAAAGAACGAAACGAATCTACGCCATGAAAGTCATCAAGAAGGAAATCATGAACGATGATGAGGACATTGACTGGGTCCAAACGGAAAAGCATGTCTTTGAGATCGCCTCCAATCACCCCTTCCTCGTCGGACTCCATTCTTGTTTCCAGACGCCCAGCAGATTATTCTTTGTGATAGAGTTTGTCAACGGTGGCGACCTCATGTTTCATATGCAGAGGCAACGGAAACTCCCGGAGGAACACGCTCGCTTCTACGCTGCTGAAATCTGTCTCGCTCTCAACTTCCTTCATGAGAAAGGAATAATTTACAGAGATCTGAAACTGGACAATGTACTGCTCGACTCCGAAGGTCACATAAAGTTAACAGATTATGGAATGTGCAAGGAAGGGCTTCGAACAGGCGACACAACGAGTACATTTTGCGGAACGCCAAACTACATCGCTCCAGAAATGTTACGTGGAGAAGAATATGCTTTCAGTGTCGACTGGTG

Cloning info

PCR rxn on 5/11/15 using GoTaq polymerase mix

| Template | Initial denature | Denature | Anneal | Extension | #cycles | Final extension |
| --- | --- | --- | --- | --- | --- | --- |
| 2 hr cDNA | 4min @ 94C | 30sec @ 94C | 30sec @ 55C | 2min 15 sec @ 72C | 35 | 10min @ 72C |

Purified PCR Product with Thermo Scientific kit on 5/13/15, Second PCR from purified template on 7/29/15

Ligated PCR product into Pgem T Easy vector and transformed into competent cells on 7/29/15

Picked colonies for overnight cultures on 7/30/15

Isolated plasmids using Promega miniprep kit on 8/10/15

Confirmed clone aPKC1 by sequencing on 8/13/15

aPKC4 is sense to T7. RNA probe generated using Sp6 on 1/20/16

**Par3B**

Gene model: 5633 nucleotides

ORF: 499 – 5286 = 4788 nucleotides

>Par3B_full

ATCTCATTGGACTCGTGTTCCCGGATGACCACAGGGAGGCGCTGCAAAGATGGTCACGCTCAGTTCTTGTCTCGGCCACATGAGGCGCTTGAGTTGAGGGGAAGTTCCGACATTATTCATTTGGACTTGTTGTGATGCTCACCTGAGGATAGCTGGCGGTCGTTTATGAGGCTGCATGAGCCGTAATTTCGCAAAATGAAGTCTTCGGAAGATAAGGAAGACACGTAGATTAATTCAGCGTAATTTGGTGAGCCATAAATGAACTGGAAGTGAATTTACCAAGGAACAATGCACCTGAAGAAAGTTACAAACTTGGCCACCTGCCTTCTCACAACTGTGGCGTTTACAACTAGTCTCAAATAAAAGTGGGTCGCTGAAAAACGTTGACTGGTGGATTAATGTCGCTTTACCTTTTGTGGTCATGATCATGTGGATCAGTGTTTACGATATACGGACTTATTTAGGCGTTTCGTGAAGAAATCTGTCAAATATATCGCCATGAAAGTGACAGTTTGTTTCGGGGCCACTCGCGTTGTTGTGCCTTGTGGAAATGGGGATTTACCAATCAGCGAACTCATTGACATGGCCATCACCCGGTACAAGAAGGCTTCTGGAAAGGCCTTGGATTACTGGGTGACTGTGCATACTTTGAAGTCTCAGTCAGATGGAGGAATTCTTGACCCTGACGACCTGCTCACTGATGTTGCTGATGACAGAGAACAGTTAATCGCGGAATATGAAGAACAAGATGCGCCCCTGGTGCCACACAACGGAGGGGACGGGACGAGCGCCAGTTCTGTGGGGACGGCCAGCCCCGATATGTTCCACGCTCCAGAAATCAACAACCAACAACACAACCTCAACAACAACAACAAGCCCTACGCTCCGTCCAGCTACGATGTTGTCATCACCCAGAATGACTTGAACAATGTTTCCTCTAGGTTAATTGTTAGAAGAGGAAGCGAACCTGCCTTGATCAACATCGGAATTGAAAAGGAAAATGAAAATCCTCAGTTATCTTTGAAAAACAGCAACAAACGATGGTCAGCTATTTCTTTGGTGGACAATGGAAGGATACCAAAGCAAGAGGATGCTGATGATGAGGAGAGTGATGAGGAAGGAGGACTGTACCCGCCTCAGCAGCGCACGGGAGGCAATGGTCAGGAGAAGGACAAAGAAGTGCGCAGTGCCTTCACCAGGTTTGCAAGAGACTCTGCTCGCCAGTCACTGGCCAGCACCAACTCAGCCATGTACAGGTGGCTGGAGGCGCAGGAGAGGACTGAGGAAAGAGCCATTGAGATGCAGGTGAGCAGAAAAGAGCCGTTAGGAGGATCAGGGAAGGCAGAGGATGAAAGTGAAGAAGAAGACGAACAAAGCAAAGAGAACAATGTTGACTTTAGAGACAAAGCTGAAATTATAGCCCTGAAAAATACTGGTGGGGCACTAGGAATTCATGTAGTGCCTGAATATGATGAGAAAGGAAGAGAGTTAGGCTTAGTAGTTCAAGGGGTTGAAGCTGGCGGACGCATTTCGAGAGACGGCAGACTCAAAGTGAAGGATCAAATTATTGAGATCAATGGAACAAGCTTAGTTGGAGTTGATTTCCTCAAGGCACAAGAAATTTTCAAGCAAGCTCTTCAAACGGAAGAGATTCGTATGAGGGTGGTTAAAGCCGACCCTGAACACTCTGACAATTCATCTCCCGAAAAACTGCCTTCTCCTGCCAAAAAGAAACCTGATGATTTAGACTTATCACAAATACCTGGTTCTCCTGGTTTGAATTCTCCTCAGAGGCCGGAAGGGGCCGAATCTCCTGGTAAGAGAGCCCCTCCCGTCCCCGTAAGAAATCCCAGCACGGCATTATCCAGTGGTGCTTCTTCTCCAACCAGGTCTGCCCTCATCCAACCCACCAACACTCGCAGTATAGGCGAGATCATCACCATTGAACTCCATAAAGGTAATGAGGGTCTTGGCTTCAGTGTCACCACAAGAGACAACATGCCAGGCTCAGAAATCCCAGTCTATGTCAAGACGATACAGAACCGTGGTCCCGCTATCAAAGATGGTCGCCTCAAGTCTTGGGACAGATTGTTAGAGGTAAATGGCATCCAGATGACCGGCAAGACCCAAACGGAGGCTGTGAAGGTGCTGAGGGAAATCCCAATTGGAACTGATGCCGTCTTGAAGGTTTCTCGGCAAACGACAGTCACCAAACCGAAGTTCACAATGCCCAGAGAATTGCCTCCTGGCTCTTCTTCTCCCCCTTCATCCACAACAACTGTCTCACTACAGCCAACAGACAAAGCTGCCGAGGAGCTGACCACCCTCGCCCCCAACGCCACCAAGGAGGTCCACTGCTTCAACATTCTCCTCAACGACTCAGGCTCGGCCGGATTGGGAGTCAGCGTCAAGGGAAAGACAACGACCAACCCAGAAGGAACCACGGACCTCGGCATTTTCATCAAGTCAATCATAAGCGGTGGAGCTGCTCATAGGGATGAGCGCTTGCGAGTGAACGACCAGCTTGTGGAGGTCAACAACCAGCTCCTTGTTGGCAAGTCCAACACGGAGGCCATGGAGACCTTGAGGAACGCCATGCAGAGGGAGAGTCCCGTTCCGGGCCACATCAAGTTGGTCGTAGCTCGGAGAAAGGCCTTACCCGCTCCTGACGAAGCAGACCAATCTCCTGTAGACTCTAACGAGGTGCGGATAAGTGCCACTGACAATGTGGACGGTGGGGATAAGTGGGATGATGAAGGTTTCAACAACCTCCACTCCACTTTCAAGAGTGATGAAAAGCCCCAATCTAACACTGCCCCCGCTGCCGCTCCTAAGCCCTTGCTTGACAGAATCACAAACGTCACCACGACGCCCTCCCCCCGCAGTAACAAAGCCAGCAACATGCAGTCCCCCACCATTCACGCTAACCGAGCCGAAACTGTACTAATAGAGGGAGAGAACTACCAGGTCCAGATGCGCCAGACAGACAAACACAAGCCGCGACCAGCCTCTACTCTAGGGTTCCTGCAGAAGGCACCAGATGGCAGCACTAAGCTCTCAAGGAGTTCCGAGAATCTTGCCCAAGAAATCGAGTGGGATGCTGAGTCTAATATGTCCACAGACGTCGATCCCCAAATAATATTTTCAAGGGAAGGATTTGGGCGGCAGAGTATGTCTGAAAAACGCAAGGGTCACCTCGATCCTAAGTCTACGGAACTCTACCAAAAAATCAAACTGAACAAAGCTCAAGGAAACCAGTACTTATCAGCCACTTGGGAACGCAGGCGTTCAGCATCTTATAAAGAGCCAGGTTCTAGAGTAGATGCACCTTGTGTGTATTTCCCTCGCAGAGCTGGTTCAGTAGAGTCCCTGCTTAACAGTAACTACTCTAACCTGCAAGACTTGCATGAACAGAAGAAGGATGGCGTTGTGGGACCTGCCCTGGGACTGACTAAATCCAGCTCCCTAGAAAGTCTTCAGACTGCAATTCAGATTGCAGAGGAGGAAGACAGCGAAGTCACTTCGGCCTTTAAGTCTCCAACCCGGTCGATGGTCAGAGGACGCGGATGTAACGAGAGTTTCCGAGCAGCGGTTGATAGATCTTACGAAGGCGCAAATGACGCCGAGGCTATGGATACCTTGGAAGAGGAGAGCAGCGAGACGGGATCGTTCGGCCGTGGCCCTGGGGGTGGGTCAGTGCGGTCCTCACACTCTGGCAGTGAGCTGGACGACAAGCGAAAGAAGGCTGGCTCCAAGTCAAAGAAAGACAAAGGATTGTTCAAATTTTTCAAGTTTGGGAAGCAGAGCAAAAAGGACGGATCTGAAGCTGGTCCTTCCGAAATCAAAGCTGCTGATGTTGACTTTGCCAAACTCAAACAAGAAGATATTCCTAAATACACACCTAAGACTGAAGAGACTGATAGCAAAATCCAGGAACAATTTCGAGAGCTCCAAGCACAAGTTCTGGAGCAGCAGAAACTGCACGACATGCAGGTGGCCGAGATACAACGACAGAACGAGAAGCAGCGATACCTTGAGGCGCAGATGAGAGCGAGGTCTCCTTACGGAGCTCCCCCTCCTGTGCCCCAGGAACCTCCTCCCAGTTACCAGGCCACCTCTCCCTCTCGGACGCGCCAATCTACTCAAGCAGAGAGACTGCAGAATCTACGAGCGCATCATCAGCGTATCCATCAAGAGAGACAAGGACGCTACCCCAATGAAGATGTGGAGGAGCAGTATGAAAGACATCTGTTGCAGCTTGAACGACAGCGGCAGATGGCAGCACAGAGAGCTTCAGCCTCAACAGTTCCCCCTGGTGGGGAGTTCCAACTTCCTCCTCGTCGGCCTCAAGATCGCAATTACAGCGGACCCCAGAGCAGACAAGGATACGCTGACCCCGAACGATACAGTCATTACCAGAATCTGGGAGAAATCCAAGCGCAGCTGCAGAAGCATGCGCACTACCAGCAAATGTACAAGCAGCAAACGGGACAACCCCCCTCTCGGTCGATGGTTGATCAGCAACGACAGCTGGTTGACTCCTCTCGGCACCTGGTTGATCAGCAACAGCAAAGAGCTCTTGCCGAGCAAAGACGTTTACTGGCTGAGCACCAGCAGAGGCAAATAAATGACCATCCACCACAAAGGGAGCTTTCCTCACAGAGGGAACTGCCCCCTCAAAGAGAACTGCCCCCGCAAAGGGAACTGCCCCCTCAAAGAGAACTGCCCCCACAAAGAAACCCTCAGTATATGCACCCGCAACAGATGCACGCCCAGCAGATGAGGCAGGGAGGTTACATGGCCCCTCAGCATCACCAACGCCCCCTGCACCACCCTCCCCACTCGGGGGTCTTCCACAGCCACTCTGGGTCAGACCCAGCCAGGCATTACGGACTAACAGAGACAAGAAGTGGGGGATTGCCCCATTATTACTCTCAGCCTGACACAAGATACCTCCACCCCAGCCTCCAACCTCGCAGTATCACACCGGGTGCCTCATCGTCCACCAGGGGGAGATCGCCTTCCCCTGGCACCACCAGAGCGCGCTCTCATACCCCCATCAACCAGGTGGCGTACCTGTCCCAACACACCGCTGCCAAGTCTCACTCTAGTGCCTTACTGCCCCCTTCCACTAACGGTGGTCACCAGAAACGCTCTTACTCCACAGACTATGCCGAGTATGATGACCCTCATTCCGGCTACCGGGACCCTGGGTTCAATTCCTTGCCCCGGAGACCCAGGGAGAGAGCGTCCAGTGAGATCATATCCGGTCCTGCCCAAGTATGAAGTCTTCCGTTGTTAATATATCACTACGTCCAAAATTGAAGTTAATATATTTTGTTAGCTGTTGTCTTTCTGTTTACATTAGGTAAACATGAATTTTACGAGTTGTAGTAATAGATACTACTAATGTTTTTAGAAAACTGCTGTTAAATTCTATTCCATCCATATATTCTTCACTGTTTCACTCTCTTCATGAAGTTTGCTCCAGCTTTAAATTTTAGCCATGTTAAATATTCGTACTTTGTTAGTACTCGATTTTAAGCGGGACATAATTTTACTCTGTTAACATTGTATGTTCAATGGAAACTTATCTTTTATTGTTAAATGTTGAGTGAAAAAGACTGTACAGT

Protein: 1595 aa

>Par3B_protein

MKVTVCFGATRVVVPCGNGDLPISELIDMAITRYKKASGKALDYWVTVHTLKSQSDGGILDPDDLLTDVADDREQLIAEYEEQDAPLVPHNGGDGTSASSVGTASPDMFHAPEINNQQHNLNNNNKPYAPSSYDVVITQNDLNNVSSRLIVRRGSEPALINIGIEKENENPQLSLKNSNKRWSAISLVDNGRIPKQEDADDEESDEEGGLYPPQQRTGGNGQEKDKEVRSAFTRFARDSARQSLASTNSAMYRWLEAQERTEERAIEMQVSRKEPLGGSGKAEDESEEEDEQSKENNVDFRDKAEIIALKNTGGALGIHVVPEYDEKGRELGLVVQGVEAGGRISRDGRLKVKDQIIEINGTSLVGVDFLKAQEIFKQALQTEEIRMRVVKADPEHSDNSSPEKLPSPAKKKPDDLDLSQIPGSPGLNSPQRPEGAESPGKRAPPVPVRNPSTALSSGASSPTRSALIQPTNTRSIGEIITIELHKGNEGLGFSVTTRDNMPGSEIPVYVKTIQNRGPAIKDGRLKSWDRLLEVNGIQMTGKTQTEAVKVLREIPIGTDAVLKVSRQTTVTKPKFTMPRELPPGSSSPPSSTTTVSLQPTDKAAEELTTLAPNATKEVHCFNILLNDSGSAGLGVSVKGKTTTNPEGTTDLGIFIKSIISGGAAHRDERLRVNDQLVEVNNQLLVGKSNTEAMETLRNAMQRESPVPGHIKLVVARRKALPAPDEADQSPVDSNEVRISATDNVDGGDKWDDEGFNNLHSTFKSDEKPQSNTAPAAAPKPLLDRITNVTTTPSPRSNKASNMQSPTIHANRAETVLIEGENYQVQMRQTDKHKPRPASTLGFLQKAPDGSTKLSRSSENLAQEIEWDAESNMSTDVDPQIIFSREGFGRQSMSEKRKGHLDPKSTELYQKIKLNKAQGNQYLSATWERRRSASYKEPGSRVDAPCVYFPRRAGSVESLLNSNYSNLQDLHEQKKDGVVGPALGLTKSSSLESLQTAIQIAEEEDSEVTSAFKSPTRSMVRGRGCNESFRAAVDRSYEGANDAEAMDTLEEESSETGSFGRGPGGGSVRSSHSGSELDDKRKKAGSKSKKDKGLFKFFKFGKQSKKDGSEAGPSEIKAADVDFAKLKQEDIPKYTPKTEETDSKIQEQFRELQAQVLEQQKLHDMQVAEIQRQNEKQRYLEAQMRARSPYGAPPPVPQEPPPSYQATSPSRTRQSTQAERLQNLRAHHQRIHQERQGRYPNEDVEEQYERHLLQLERQRQMAAQRASASTVPPGGEFQLPPRRPQDRNYSGPQSRQGYADPERYSHYQNLGEIQAQLQKHAHYQQMYKQQTGQPPSRSMVDQQRQLVDSSRHLVDQQQQRALAEQRRLLAEHQQRQINDHPPQRELSSQRELPPQRELPPQRELPPQRELPPQRNPQYMHPQQMHAQQMRQGGYMAPQHHQRPLHHPPHSGVFHSHSGSDPARHYGLTETRSGGLPHYYSQPDTRYLHPSLQPRSITPGASSSTRGRSPSPGTTRARSHTPINQVAYLSQHTAAKSHSSALLPPSTNGGHQKRSYSTDYAEYDDPHSGYRDPGFNSLPRRPRERASSEIISGPAQV

Domains

DUF 3534: 1 – 157

PDZ: 310 - 390

PDZ: 478 - 567

PDZ: 623 – 715

Clone

Partial ORF

Primers

Forward: CATCACCCGGTACAAGAAGG

Reverse: CCTGGTTGGAGAAGAAGCAC

Nucleotide# 588 – 1890 = 1303 nucleotides

>Par3B_clone

CATCACCCGGTACAAGAAGGCTTCTGGAAAGGCCTTGGATTACTGGGTGACTGTGCATACTTTGAAGTCTCAGTCAGATGGAGGAATTCTTGACCCTGACGACCTGCTCACTGATGTTGCTGATGACAGAGAACAGTTAATCGCGGAATATGAAGAACAAGATGCGCCCCTGGTGCCACACAACGGAGGGGACGGGACGAGCGCCAGTTCTGTGGGGACGGCCAGCCCCGATATGTTCCACGCTCCAGAAATCAACAACCAACAACACAACCTCAACAACAACAACAAGCCCTACGCTCCGTCCAGCTACGATGTTGTCATCACCCAGAATGACTTGAACAATGTTTCCTCTAGGTTAATTGTTAGAAGAGGAAGCGAACCTGCCTTGATCAACATCGGAATTGAAAAGGAAAATGAAAATCCTCAGTTATCTTTGAAAAACAGCAACAAACGATGGTCAGCTATTTCTTTGGTGGACAATGGAAGGATACCAAAGCAAGAGGATGCTGATGATGAGGAGAGTGATGAGGAAGGAGGACTGTACCCGCCTCAGCAGCGCACGGGAGGCAATGGTCAGGAGAAGGACAAAGAAGTGCGCAGTGCCTTCACCAGGTTTGCAAGAGACTCTGCTCGCCAGTCACTGGCCAGCACCAACTCAGCCATGTACAGGTGGCTGGAGGCGCAGGAGAGGACTGAGGAAAGAGCCATTGAGATGCAGGTGAGCAGAAAAGAGCCGTTAGGAGGATCAGGGAAGGCAGAGGATGAAAGTGAAGAAGAAGACGAACAAAGCAAAGAGAACAATGTTGACTTTAGAGACAAAGCTGAAATTATAGCCCTGAAAAATACTGGTGGGGCACTAGGAATTCATGTAGTGCCTGAATATGATGAGAAAGGAAGAGAGTTAGGCTTAGTAGTTCAAGGGGTTGAAGCTGGCGGACGCATTTCGAGAGACGGCAGACTCAAAGTGAAGGATCAAATTATTGAGATCAATGGAACAAGCTTAGTTGGAGTTGATTTCCTCAAGGCACAAGAAATTTTCAAGCAAGCTCTTCAAACGGAAGAGATTCGTATGAGGGTGGTTAAAGCCGACCCTGAACACTCTGACAATTCATCTCCCGAAAAACTGCCTTCTCCTGCCAAAAAGAAACCTGATGATTTAGACTTATCACAAATACCTGGTTCTCCTGGTTTGAATTCTCCTCAGAGGCCGGAAGGGGCCGAATCTCCTGGTAAGAGAGCCCCTCCCGTCCCCGTAAGAAATCCCAGCACGGCATTATCCAGTGGTGCTTCTTCTCCAACCAGG

Cloning info

PCR rxn on 6/15/15 using GoTaq polymerase mix

| Template | Initial denature | Denature | Anneal | Extension | #cycles | Final extension |
| --- | --- | --- | --- | --- | --- | --- |
| 2 hr cDNA | 4min @ 94C | 30sec @ 94C | 30sec @ 55C | 2min 15 sec @ 72C | 35 | 10min @ 72C |

Purified PCR Product with Thermo Scientific kit on 6/22/15, Second PCR from purified template on 7/1/15

Ligated PCR product into Pgem T Easy vector and transformed into competent cells on 7/1/15

Picked colonies for overnight cultures on 7/8/15

Isolated plasmids using Promega miniprep kit on 8/10/15

Confirmed clone P3B1 by sequencing on 8/13/15

Par3B is sense to SP6. RNA probe generated using T7 on 11/9/15

**Par6**

Gene model: 6945 nucleotides

ORF: 499 – 1608 = 1110 nucleotides

>Par6_full

TTCTTTAACCACGTCTCCATCTTAGGTCTTTACACACACGAAAATCAAGGCATATTTTCGAAGGAATCATGGACTTTCTTGCGAGATAAACATTTCACATTTCTGTAGAAATGGCGCCAGTTGCAGCTTCCGGTAAACTTTAGGGGGTTTCCCGGTGGGCGGCTCACTCCAAAATGACGTCACTCTTTATCTGCCGTGTCCTACTTAAGATTTTTTGTGTCTGATGACTAAATGCTGGTATCTATTTCTTGTTGGATGATCAAGGCAGCTGCCTAGACTCTTGTGAGACAACATTAGCAACTTTTTGAAGAACTTTTCTCATTTGTTCCATTTGTTTACCTGGGAATTCCTTCACAGGTGCCCGATCTGGGAACGAGGCTTGGAAATCGCTAGCAAGTTGTGTCTTGATGAAAAGAGACAGTTTATCGGCTCCAATGTGGAATTTACCTGGATTTAACCGGTGACCAAGCCCTGGAGGAGGGCACAAAGTCATCCACGATGTCTATGAACGGGAAATCTGGCCAAGTACTGGCCAACAGAGTCATTGAAGTCAAGAGCAAGTTTGATGCTGAATACAGAAGGTTTTCAATCAACAAGTCGAAAATTCTTCGATATGAGGAGTTTTACCAACTCTTACAAGACCTGCACAGATTAAAAGACATTCCATTTATTGTGTGTTACACGGACCCCAAAGATGGGGATTTATTACCAATCAACAATGGGGACAACTACATGCTCGCAATCAACACCACGCCAAGTATATTGCGGCTCACTGTACAGCGAAAAGGCGAGAGTATATTCGAAGTCAACGGCTATGGAACATTGGGGCGACCGAAGAAGAACCCCATTACCAAATTTATGGCCAGCGAGCAACCGATTAAACCAAAAATACCAATTAGCATGCCAGAGGACTTTAGGCGAGTGTCGGCCATTATTGACGTGGATATTATTCCCGAGACACAGCGAAGAGTTAAGCTGATGAAGAACGGTAGTGATAAACCCCTCGGATTTTATATTAGGGATGGTACGAGCGTACGTGTGACCCCTCACGGATTGGAGAAAGTCCCGGGGGTGTTCATATCGCGATTAGTGCCGGGAGGACTAGCGGAGAGCACGGGGTTAGTGGCCGTCAACGACGAGGTTTTGGAAGTGAACGGCATCGAGGTTGCTGGTAAGACTCTCGATCAAGTGACTGACATGATGGTGGCTAACAGCTCCAATCTAATCATCACGGTGAAACCCGTGAACCAGCGGAACAACATTGCTCAAAAGAGCAGCAACGCCAGCAGTACTAACTCGATGCGGCAGAGTGTGATTTCGGGGTCGTCGCAACAAAGTTCGACTCACTCGTACGACTCGGACGAGATCCATGACTACGAGGAAGACGAGGACGAAGTCAAAGACCATCTCCAGAAAGATCACTTGATGAAGAAATCGAGCTCCTCTTCCTCTAATCCTCATCACAAAGATACCACATCCCTCTCTTCCATGAACAGCAATTCCCAAAAAGACTCGCCAAAATCTGAAGCGAACGATTCCGCATTATCCGCTTCCCCCTCAACGTCACAGTCGGCCCAAGATAAAGACAGCCCCATGGCCATGTTATAGCTAGGCCTGACTGTGCTGCTCGCTCTGGCTATAGCCTATATCAACGGATTCTAATGTCGAAAGATATATATGCAAGATATCTCAGAGATATCATCTCAGATATGGGATGCGAGATACGGGATAGTATCGTAAACATGTGATGTGTATTCTTGGATGAAGACTGGAGATGTGTTTTATGAAAGATGATCGAAGATCGCGTGTCTTAAAGGTGTTATCTACTAATTTCTTAGAAATGAGTATCATGAAGATGTGTCATTCGAGAAAGATGTGTTACCTGAAGATGTGTATCTTGAAGATGTGGAAGATGTGTCTTAAAGGTGTAATCTTGATAGATGTGAGATGTGTATCTTGAAAGATGTGTTTCCTGAAGATGTGTTTAAAGATGTTGATCTTGAAATATATGTCAATTCCTGAAGATGTGTATTGATCTTGAAGATGTGTCTTAGAGATATTTATCCTGAAAAATATGTGTCTTGAATAGTGTGTCTTAAAACATGTTTATCTGAAAGGATTGGTATCTTGAAAGATGTGTTTCCCAAAGATGTGTATCCCAAAAAATGTGTATCCCTAAGATGCGTATTTCAAAAGATGTGTATTTTGACGATGTGTAATTCCGGAAAGCAATCAGTGAGATATGTCGAGAAAGATATCAAAAGATACATAATATTATTTCTTGAAAGATATCAAAAGATACATAATTTTATTTTTTGAAAGATGTCCTTAGAAACACATCAGAAACATATTTCTTAAAGACGCAAACCTTGATTGATATTGAGGTATGTTTCTTGAAAGACTCGTATTAAAGATCTCTGGAAGATGTGTTTATAAGGTACTATTGAAAGGTGTGTCACGTAAACATGGTAAAATGCGCAGCACATGTTGTGAGCTATTTATAGTCACAGGAAATCATCTTAGACCTACATTTCATCATCTTCAATATCCACAATTTCAAGGATACCGCTTGTACAAGTCACTAATCGGCAGGAAAACACTTTGGTAATGATAGGCCTACTACATTTCTACGGAATCATGTTTTATACGTTTCACGAAGTCAAAGAACACAGTCAATAGTACTCAATACACAGCACAGAAGGTACACGAAGGATACTAAACATGCTGAATAATGTTATGAATTGATGTTTTTCGATACAAATGTTACACAAGTTGTTACGGAGGAATATTCTTATAACCTGAGATTTTAACGTCACTCATTAAAGCACTGGTGCCTCGTTTGTTTTTACAGAAGATGTGATGTCATGTTAGTGGTGTGACGTCACTGGTGATAATGTCATTCGTATGATTCTATTCAATTGTGACGTCACGTGTCATGTGATGTCGAAATGGTTATGTTACGTCATTCATGGAACTTGTCATGTGACATTCTATTGTGACATCATACTTTTCAATTGTGATGTCATAATAATGTCATGTGATCATTTGTGACGTCACTGGATGCCTCTGTTAATCTTGTTAATGCTGAAAGATGAGAAATGATACTTTGACAAGTTTTAATAATTGAACTATGATTGGATCACAATCAGATGATAAAATCTGTGATTAATCGATTGATTATGGATTGATTAATTAATCAAAGTCACTAAAATGATCATGACTACATCATGACAAACACTAATCCCTATTCCTATTGCCAACTATTGTTGTTACTATAAATAGCTGACAAGTCTAAATAGACTTTAGCTTTGTTGATTTTGCGATATTCGTTAATTTACAACAAAGCTGTTTTTACTTTTTATGATTTTTATTGCCGTGAATTTTCACTTATTGTGTATTGACATAGGGTGAGAGGGACCTGTACATTTTTCTGCATATAAAAAAGTATTAAATTTTTAGCATTGGGATATGACATGAAAATATCACTACATATAATTATTATTATATTGATGATAACTGTAATGGTGATAAAACACGTATTAAATATGTTATAAATTAAATCAAAATTTGATAAAATTTTGATTTACGTTGACCATAACAGGATATGACACATATGCTAATTTTAAGGTCATGTACATTATGTAAATTATAACACCTAATTATGTAAATTTATGCTTAGTTATTTGGTGAAATTGAATTTGTAATCTCAAGATTAAACTGTTTAAACAGTAAGATATGATTCACTGATACATGAATCATAGTCAATATATGGTCCGTTAGTCCATTGACAACATAAAAAGTTAGATAAAGTTTATTGTATGTTGACAATTGTTATCGCATCCCAATTTATTGAGTTGATTTGTATTTTGCTTGAGTCTCGTTACAGTATGGAGAAAATTAAACCTCAAAAGAAAACCATTCCATCCACATAATTGCCTTATTTCGAAGCATTTCTACGTACTTATGTGTTTGTGCATTTGTTCTTATGATTAATGCGAAATTAATCAAGAAATTCAGTACTTAAAAATGTCATTTGTTATTGAAAACACATACAAATTTCGTCCTGTAATTATACGTTTCATGCAGTAATTACAAAATTTGATTCACCTGTACATTTCTCTCTTGAGTCTCAAAATTGTTGATGCCTTTTGTTAAAATAAGTTAATACGCCTCACTTTAAAATGTTGGCTGACAAAGTTACTGAATTAGGACAGAGTTACATCTGTTACTTGAAGAGTTGTAAAATTCCATATCAACTCAAACTGAGTTAGAGACTTGTTAAACTATGTTAAAATATTTGGACTAAATATCATTGTTATATTTTCATTGTACCAAGTTGAAATATTACATGTACATTGTATTGCTAAAGATGCTGTTATGTTGCTAAAGTATTTATATTCTTGCCATATTATAAGATTGAATATCTGCTAAGTTATATGCTAAAAGGTGCATTTCTTAATATTTACTAAGAAGCAAGAATACATAGAACCAATTAAACAATTATGAATTGATTAACTAGTATCTAACATTAAGTATAAAAGTCCAGATTGGAAATTACGATTTAATTTTAATTTTCTCCTATCCAACAAACAACACCTATTAATTTATGTTGTGTTGCCCTCTTCAGCAACGTGCATTTTAGACGTATTTATCATATCCGATGAACGAAACAGTCTATCTAGAATTAAGTATGAATTTTAAGGTTGAATTTTTATTATTGAACAATCATCATCATAATTGAAATATGACTGATCTCTATGATGCTACAAATTTACTAAAATTTAATTGCTATTTAAAAAACTTTGTATTTATTAATTGATCACAAGTTCACAATCATTTGTCGCACTTGCATTTTTCATTTGAAAGTACAAATAGAAGTAACAGAAGATGATGGTTCTTTTCATACAACCATAAAAATATTTATTAAATTGTGACATGTTAGTCCTTTGAATGTTTAATTTTCAGAAATGAAAGTCTAAAAAATGTTTGTTGAACTATTATACACTTGTTGGAACGAGATTATTTAAATGTTTTACAGAAAAAAATATCATGCTGACCAAATTAACTGTTATCTGTGCCTCTTAAGTGGTGACAATTAAAGCCTTTAAATATAAATGTGATGAAATGCATCAATGTCAAAGTACCTTATATAATGCCTTTTCAAAATATTAGGTTGAAATTGTATTTATTTTGAAGAATCAAATTTTAAAACTGTGGCCTTGAGGGACCTATTAACAAAATTTAATTGTAAATATTTAAAGTGTTTCATCATTCATGAGTTAGTTGAATTTGGAAAAACGGGATGTTAAGTACTTAACAACATTAAATTATCATTTGTTTATTCATTTTTATAAAACTAGTTGTATGAGGCTCATAATTTTTATATTACGTCAAATTAAAAATATATGCTTCTTCGATTATGTCATTGTGACCTTAATTATAATAACATTAATTGAAATGGTAGTCGAGTAGATTACCTACAAATTATGAATCACTGGCGTACTTGAACTATTTTATATTTAACGTTTGTAAAGTTTTGCACGATATGTAGCAATGTTGACTATTTTTAAAATTTGCTGTATTGGGAGGGTAAAGTAAACTGTATGTGTTTGTATAATATTATATGCAGTACCATGTACTTAGTGTGATAACATATCAACCAATCCATCCACTAACTTAAAAAAGTTCAATTTAACTCGAGATGCTCATTGTATATGAAATCACTGTTATTTTACGAATAGTGTGGTTTTGAATCATGTGAATCTTAGAATGTTTACCAGCTACTATTGTCGTTAAAAAGATTAAATTCATGACCTAATTTTGTACTTAAAATATAATTTTCGGGAGAGGTACTGAAGTTGTTTTTCACTACAGTGACTTGATATTTTGGATTGTAAGTGATAAAGAGAGTAAATTAGAATTAATTTTAAGTTGCTGGAAAATATTTAACAGAAAAATTCTTTGAATTTTCGTTGGGCAGGAGGTCAATTCTAACAGTATAAATTTAAGCAGTCATATTAAATACAGTAATGAGTTACAACAGTTTGGTTTGGCAAATTTTTAAAATATGGTTAATCAAAAATCTCCGCAATATTCAATATTACAAAGTCTGAACAAAAATATTGGCTGCCTATGACGATAAGTCATATGGAATCAACATTTGTTATTTTGTACATTTAATATTTTCACATGATGCTACATTGGGGGTACATTCTGTCATTTTATGTCGATTGTTATTTTGGGAATACACTCGCAAATGTGACACGATGTTATTTTATGAAAAATGTTACATTGCTTAATCACAAAATGCTGTACACATTGTATATAAACATATATGACAATAACACAGATCATTTGCATGTTTTGTTGATCATTCACATTTTAGCATAGATTTTAAAACTGCTAGCATATTATGTTAGATTCGATTAATTGATAGAACACTTGATTTTATTGTGACACACTTGAATTGTGTCTGCGATGTTATTGGGTTGATTTTTTTTTACTCGAAATAAATCTCGGCTCTGTATATCAGACTTTTTGAGTCTCAAATCTGCGTCATATCGTTATCATGCCTATTTACTCGTATTAATTTTAAGTTTTTTAGTTGTTTTTTTTTCCACGGCATACTTTTAAAGTAAACAGTAGTCGCTTCTACTCCTGTACTTCAATAACACCTGATAATAAATATTATATATGAAACTGCATTACATTGTATTTTTGTCTTTCACTAATTTGAAAATAGTTTACCTATGCTAACTACTTATGTTATAAATATTGATGGAGTTCGGTTTACAACTAAAGTGCTCTTGCTGGATTTCTAGCCTACCTAATTTGGGATGATGGTCAATGGGATCGGCACCAAAAACTGGTATGAGTATTTGATGTAGAATTGATGTAGAACTATCGATATCGGTGGTATCGGCACAAGTTACAGCTGTACCTGAA

Protein: 369 aa

>Par6_protein

MSMNGKSGQVLANRVIEVKSKFDAEYRRFSINKSKILRYEEFYQLLQDLHRLKDIPFIVCYTDPKDGDLLPINNGDNYMLAINTTPSILRLTVQRKGESIFEVNGYGTLGRPKKNPITKFMASEQPIKPKIPISMPEDFRRVSAIIDVDIIPETQRRVKLMKNGSDKPLGFYIRDGTSVRVTPHGLEKVPGVFISRLVPGGLAESTGLVAVNDEVLEVNGIEVAGKTLDQVTDMMVANSSNLIITVKPVNQRNNIAQKSSNASSTNSMRQSVISGSSQQSSTHSYDSDEIHDYEEDEDEVKDHLQKDHLMKKSSSSSSNPHHKDTTSLSSMNSNSQKDSPKSEANDSALSASPSTSQSAQDKDSPMAML

Domains

PB1: 16 – 94

PDZ: 156 - 247

Clone

Partial ORF

Primers

Forward: GCTATAGCCAGAGCGAGCAG

Reverse: GAGGAGGGCACAAAGTCATC

Nucleotide# 475 - 1643 = 1169 nucleotides

>Par6_clone

GCTATAGCCAGAGCGAGCAGCACAGTCAGGCCTAGCTATAACATGGCCATGGGGCTGTCTTTATCTTGGGCCGACTGTGACGTTGAGGGGGAAGCGGATAATGCGGAATCGTTCGCTTCAGATTTTGGCGAGTCTTTTTGGGAATTGCTGTTCATGGAAGAGAGGGATGTGGTATCTTTGTGATGAGGATTAGAGGAAGAGGAGCTCGATTTCTTCATCAAGTGATCTTTCTGGAGATGGTCTTTGACTTCGTCCTCGTCTTCCTCGTAGTCATGGATCTCGTCCGAGTCGTACGAGTGAGTCGAACTTTGTTGCGACGACCCCGAAATCACACTCTGCCGCATCGAGTTAGTACTGCTGGCGTTGCTGCTCTTTTGAGCAATGTTGTTCCGCTGGTTCACGGGTTTCACCGTGATGATTAGATTGGAGCTGTTAGCCACCATCATGTCAGTCACTTGATCGAGAGTCTTACCAGCAACCTCGATGCCGTTCACTTCCAAAACCTCGTCGTTGACGGCCACTAACCCCGTGCTCTCCGCTAGTCCTCCCGGCACTAATCGCGATATGAACACCCCCGGGACTTTCTCCAATCCGTGAGGGGTCACACGTACGCTCGTACCATCCCTAATATAAAATCCGAGGGGTTTATCACTACCGTTCTTCATCAGCTTAACTCTTCGCTGTGTCTCGGGAATAATATCCACGTCAATAATGGCCGACACTCGCCTAAAGTCCTCTGGCATGCTAATTGGTATTTTTGGTTTAATCGGTTGCTCGCTGGCCATAAATTTGGTAATGGGGTTCTTCTTCGGTCGCCCCAATGTTCCATAGCCGTTGACTTCGAATATACTCTCGCCTTTTCGCTGTACAGTGAGCCGCAATATACTTGGCGTGGTGTTGATTGCGAGCATGTAGTTGTCCCCATTGTTGATTGGTAATAAATCCCCATCTTTGGGGTCCGTGTAACACACAATAAATGGAATGTCTTTTAATCTGTGCAGGTCTTGTAAGAGTTGGTAAAACTCCTCATATCGAAGAATTTTCGACTTGTTGATTGAAAACCTTCTGTATTCAGCATCAAACTTGCTCTTGACTTCAATGACTCTGTTGGCCAGTACTTGGCCAGATTTCCCGTTCATAGACATCGTGGATGACTTTGTGCCCTCCTC

Cloning info

PCR rxn on 10/27/15 using GoTaq polymerase mix

| Template | Initial denature | Denature | Anneal | Extension | #cycles | Final extension |
| --- | --- | --- | --- | --- | --- | --- |
| 2 hr cDNA | 4min @ 94C | 30sec @ 94C | 30sec @ 55C | 2min 15 sec @ 72C | 35 | 10min @ 72C |

Purified PCR Product with Thermo Scientific kit on 11/9/15, Second PCR from purified template on 11/9/15

Ligated PCR product into Pgem T Easy vector and transformed into competent cells on 11/9/15

Picked colonies for overnight cultures on 11/12/15

Isolated plasmids using Promega miniprep kit on 11/24/15

Confirmed clone Par6 by sequencing on 12/4/15

Par6 is sense to T7. RNA probe generated using SP6 on 1/14/16

**Par3A**

Gene model: 2479 nucleotides

ORF: 499 - 1905= 1407 nucleotides

>Par3A_full

ACATTTTGGTGGCAAGGCGGCATAATGTTCAGTTGTCATGCATGAGGGCCTGCTAATCAGATCAGCGCACTGTGATCGACTTAGATCTCTGCTGTGATCAATAACACATTATCGATAAGGATCAGAGAGATCAGGAACAACTGTGGCTTACATGGTAGCCCCTCCCTATACTTTTATCATATCAACTTTAGCAGTTGACCATGACTCTACAGACAGCATGAAGTCATTGCTCATTTTCAGATGGACTATGCAATACATTACACATGGGACTTTCTCTGAATTTCTGAGATAGAAAGACTTTTGACTTTGGATACAGAGAAATAGACACAACAAAACCTGACTTCTCTTGATAAGTAGGTTAAGTAACTATGACTGTGTTTATAACAGCTTAGTGCATGATAGCAGTGTTTACATTAAAATCCTTTTCTCTGTGGATACTGCGTTTTATCTGGCTGGTTAATCCAGTGAATTCTACCACAGAAAAGCACAGGAAGGAATATGGTCTTAGAGAACATTATGGATTTACCACAAGAAAACTTTAAGGGTAACCACAACCTTCTTATGAAGGACTCCGAAGGGCTCCCCTTCAGCAGAAAAGGGCAGAGGAAGTCCCTGGCACCCAGCAGCCCTTCCTTCTACAAGTGGATTGAGGCTCAGGAGATACAGGAGCACAAGGTTCGCAATGACTACTCTCCCAGCCAAGACAACTTTAAAGAAAATGTCTCAAACAACTGCCAGACTTCCCTCAGGGAACAAAACAAAGCCATGGTCTTCCAGGAGGAGAATATGCAATCTGTGATATCAAGGAATGGGTACATCCACCCGACCAACACAAGGAACATAGGAGAGGTCATCCACATTGAATTGCTCAAAGGCTCAGAGGGCCTTGGGTTCAGTGTGACCACCAGGGACAACATGCCAGGCTCAGAGGTTCCAGTCTACGTGAAAGCCATCCTGCCAAGAGGTCCTGCCCTCAGAGATGGCCGCTTGAGGTCCTGGGACAGAATCTTGACTGTGAATGGCATAGAAATGACAGGAAAGTCCCAGAAGGAAGCAGTTCAAGTATTGAGGGAGATCCCAGTCAACAGCTACGTCCACCTGGAGGTCTCCAGGCAAACTAAAGACAACAATGATCTGGACTCAGAACAGACAACAGAAATGGCAATCAAAGAAGTCTCCCTGATCGAGCAGCATATTCCAGAAGAGCCGAGAGTTTCTCCCCATAAGGAGAAGGTCCAGGTGCTGGTGGAAAGGAAGGGAAGGAGGCTGGGGATTGCTGTCAGGGGAAAGACTACAGAGAGTGGAAATGGACACCAGGATGCTGGCATCTACATCAGGGCCATCTGTGCTGATGGGGCAGCTGCCCAGGACGGCAGAGTGAAGGTGAACGACCAACTCCTGGAGGTCAATGGCCAAGTCCTAACAGGGATTTCTAATGCGGATGCCCTGGCTACTATGAGGACAGTTCTGCAAAGGGCAGGATCATCCCAAAGAGTTGCCCTTGTCCTGGCTAGGAGAAGGAAGGCCCCTCCTCCCCCTGGTCAAGCAGGCTCTAGCCCTGTAGGTCAGCCCAGTCAACCTTTGTCACACTCCGCATTGATGAACAATGCTCTGGCAAGAAGCCTGGAAAGCCTTTTGGAGAGTGAATCTGACTATGTTAATGAACTGGCTATGGATGTAGATCCCATGGCAGCTTTCGCAAGGGAGGGCTTTGGAAGGAAGAGTATGTCGGAGAAGAGGAAAGGACACTTGGATCCTAAGATCAGTGAATTATACCGGAAGATCAGATCATCTCGAAACATTGTCACAACATTGAAAGACCAACAGTTACGACGAACGGTGTCCATGTGTGCCCTAGACAAGTGGCAAAGACCCTCGGAAATTTTATTTTGCACTCGATTGTGAAATTACGAACAAAAACAGACATTTGCCTAGTCAACATATTTGTACATACCTTACTGAAATTGAATTGTACATAAATTGTTTGCCTGATTGTTGAGAGTTGTTTTTCTGTGCAATATTGTCATATGTTTACGATGACTCAAATGTTTTACATTCAAGATATTTTTTATTAATCATGTTTATTGACATTGCAAATCATAATACACTTATTATTGACTAAATGATTAGTCTTATAACTTTAATCGAACAAGTTAATGCTAATCTAGTCAGCTAATAATAGCGATAAATGATATTGTCTACTTTTTAAGATATTCATTAACTGGCACATCTATTAATTTTATTTATGTATTTTAAGGAAACTGTAAACATCATAAATGAGTACATTTAATGTTAAAATAAATTTTATTTTCATAATACTAGTCGCCTAGACAAAACTCTTTATTTATTTGTACATTCTGTATATTATTTTCCATTGTATTATTGATGTGAAATGTTTAATAAAAATCCAAAAATTAAGTGTTTTGATATTTTTTGCTGAAAAGTCAACAACCCATTATTAAGGTAACACATAATTGAT

Protein: 468 aa

>Par3A_protein

MVLENIMDLPQENFKGNHNLLMKDSEGLPFSRKGQRKSLAPSSPSFYKWIEAQEIQEHKVRNDYSPSQDNFKENVSNNCQTSLREQNKAMVFQEENMQSVISRNGYIHPTNTRNIGEVIHIELLKGSEGLGFSVTTRDNMPGSEVPVYVKAILPRGPALRDGRLRSWDRILTVNGIEMTGKSQKEAVQVLREIPVNSYVHLEVSRQTKDNNDLDSEQTTEMAIKEVSLIEQHIPEEPRVSPHKEKVQVLVERKGRRLGIAVRGKTTESGNGHQDAGIYIRAICADGAAAQDGRVKVNDQLLEVNGQVLTGISNADALATMRTVLQRAGSSQRVALVLARRRKAPPPPGQAGSSPVGQPSQPLSHSALMNNALARSLESLLESESDYVNELAMDVDPMAAFAREGFGRKSMSEKRKGHLDPKISELYRKIRSSRNIVTTLKDQQLRRTVSMCALDKWQRPSEILFCTRL

Domains

PDZ: 117 - 206

PDZ: 244 - 340

Clone

Partial ORF

Primers

Forward: ACCACAGAAAAGCACAGGAAG

Reverse: GAGGGTCTTTGCCACTTGTC

Nucleotide# 474 – 1877 = 1404 nucleotides

>Par3A_clone

ACCACAGAAAAGCACAGGAAGGAATATGGTCTTAGAGAACATTATGGATTTACCACAAGAAAACTTTAAGGGTAACCACAACCTTCTTATGAAGGACTCCGAAGGGCTCCCCTTCAGCAGAAAAGGGCAGAGGAAGTCCCTGGCACCCAGCAGCCCTTCCTTCTACAAGTGGATTGAGGCTCAGGAGATACAGGAGCACAAGGTTCGCAATGACTACTCTCCCAGCCAAGACAACTTTAAAGAAAATGTCTCAAACAACTGCCAGACTTCCCTCAGGGAACAAAACAAAGCCATGGTCTTCCAGGAGGAGAATATGCAATCTGTGATATCAAGGAATGGGTACATCCACCCGACCAACACAAGGAACATAGGAGAGGTCATCCACATTGAATTGCTCAAAGGCTCAGAGGGCCTTGGGTTCAGTGTGACCACCAGGGACAACATGCCAGGCTCAGAGGTTCCAGTCTACGTGAAAGCCATCCTGCCAAGAGGTCCTGCCCTCAGAGATGGCCGCTTGAGGTCCTGGGACAGAATCTTGACTGTGAATGGCATAGAAATGACAGGAAAGTCCCAGAAGGAAGCAGTTCAAGTATTGAGGGAGATCCCAGTCAACAGCTACGTCCACCTGGAGGTCTCCAGGCAAACTAAAGACAACAATGATCTGGACTCAGAACAGACAACAGAAATGGCAATCAAAGAAGTCTCCCTGATCGAGCAGCATATTCCAGAAGAGCCGAGAGTTTCTCCCCATAAGGAGAAGGTCCAGGTGCTGGTGGAAAGGAAGGGAAGGAGGCTGGGGATTGCTGTCAGGGGAAAGACTACAGAGAGTGGAAATGGACACCAGGATGCTGGCATCTACATCAGGGCCATCTGTGCTGATGGGGCAGCTGCCCAGGACGGCAGAGTGAAGGTGAACGACCAACTCCTGGAGGTCAATGGCCAAGTCCTAACAGGGATTTCTAATGCGGATGCCCTGGCTACTATGAGGACAGTTCTGCAAAGGGCAGGATCATCCCAAAGAGTTGCCCTTGTCCTGGCTAGGAGAAGGAAGGCCCCTCCTCCCCCTGGTCAAGCAGGCTCTAGCCCTGTAGGTCAGCCCAGTCAACCTTTGTCACACTCCGCATTGATGAACAATGCTCTGGCAAGAAGCCTGGAAAGCCTTTTGGAGAGTGAATCTGACTATGTTAATGAACTGGCTATGGATGTAGATCCCATGGCAGCTTTCGCAAGGGAGGGCTTTGGAAGGAAGAGTATGTCGGAGAAGAGGAAAGGACACTTGGATCCTAAGATCAGTGAATTATACCGGAAGATCAGATCATCTCGAAACATTGTCACAACATTGAAAGACCAACAGTTACGACGAACGGTGTCCATGTGTGCCCTAGACAAGTGGCAAAGACCCTC

Cloning info

PCR rxn on 10/19/15 using GoTaq polymerase mix

| Template | Initial denature | Denature | Anneal | Extension | #cycles | Final extension |
| --- | --- | --- | --- | --- | --- | --- |
| 2 hr cDNA | 4min @ 94C | 30sec @ 94C | 30sec @ 55C | 2min 15 sec @ 72C | 35 | 10min @ 72C |

Purified PCR Product with Thermo Scientific kit on 10/20/15, Second PCR from purified template on 11/9/15

Ligated PCR product into Pgem T Easy vector and transformed into competent cells on 11/10/15

Picked colonies for overnight cultures on 11/13/15

Isolated plasmids using Promega miniprep kit on 11/19/15

Confirmed clone Par3A by sequencing on 12/4/15

Par3A is sense to T7. RNA probe generated using SP6 on 1/14/16

**Cdc42**

Gene model: 4202 nucleotides

ORF: 373 - 948 = 576 nucleotides

>Cdc42_full

GACCGATCGAAAAATCAGCCGGTATGTATTGAAATAAGAACACTTGAAGGATGATGGTGCAATTTATCAATCGACCACAATCTGTAGCATGTTTGTAGCTTTAAATCGGACTTCCTAGAGGTGTTTTTAAGCTTTAAAACGGGTCTGAAGTGAGAGGCACTGCCGCCGCCATTTAACGGAAGTGAATGTGATCAGGAAAAAGTCCACCCAAAAATAAGTCGATTTCACGTGAATAACATCTGTGATTCTTCTTCAAGATTTCAATAACTCATGTTCTTGATGAAGTGACACTTTTGGTAGTGAAAATTTGACACATTAGTCCATTCTTTTGATTATTTTGACACTCCTCTCTGGCCTGGAACAACATGAAAAATGCAGACGATAAAGTGTGTCGTGGTGGGCGATGGAGCCGTGGGTAAAACCTGCCTACTTATTTCATACACTACCAACAAATTCCCCTCAGAATATGTCCCCACAGTATTTGACAATTACGCTGTCACTGTTATGATCGGAGGAGAACCTTACACATTAGGATTATTTGACACAGCTGGTCAGGAGGATTACGACAGACTCCGACCCCTCAGCTACCCTCAGACCGATGTATTTCTCGTTTGTTTCTCAGTAGTCTCTCCCTCTTCTTTCGAAAACGTCAAGGAAAAGTGGGTCCCAGAGATTGCCCACCATTGTCAGAAGACCCCCTTCCTCCTTGTCGGCACACAGATAGATTTGAGAGATGACGCTTCAACTGTCGAGAAACTGGCCAAGAACCGACAGAAGCCAATCACGTGTGAGATGGGAGAGAAGCTTGCCAAAGAACTGCGTGCTGTCAAATATGTAGAATGTTCAGCTCTAACTCAGAAAGGACTGAAGAATGTCTTTGACGAGGCTATTCTTGCAGCCCTCGAGCCCCCAGAGCCCCCAAAGAAGAAGAAGTGCGTTATCTTGTAGATCTTCGTCTTATTTCGCAAGGACCAAAATTCAATTTAGGAACTTATATGACACAAACAAACACTGCTTGTTTTTGCTATGGATGATTATGAAAAGAAACGAAACTATGGATGACGGGGAAGAGAGAACTCCGAAGACTCTAACGAAGAACTCTGAAGAAGATTATGAAGAACCGTGAAGAAGATTATGACGAACAAGTGAAGAAGCTTGGAAATGCATTTAAATTAAAAAGTTCTAAGTATGTCGTAAGTTCGCTACAGCTGACTGCAGTCTTGTAGTTGAGGTCTGTGTAAGAACAACATCCAAAGTAAAAGTTCTGCATCTAAGTTATGCTCTGTTCAAGTTATAAGTTAACATGAAAGTTACAGAAGTTATACTACAAAAGTTATCTAGTACTGCAGCTGAAAATTACACAGCTCTACTAAACTTCAAATTCTGCCTAAATTATCAATACTCTGTTCAAGTTATAATTTCAACCTGGATAGAACTACGGAAATACAATTTTAAAAAGTACTACTAAAGTTATCTACAGTACGAGAGCTCAGAGCTCCAAAGTGAACTTTAACAGAGGCTCAGAATGTTCGACATAGGTTCTACGAAGGTCAGTTCTACTGAAGTTAAATTCTTCTAAAGTTAACTACAAAAGTGTCCATTCCTAATATAAAAAATGACACTCAGGGTTTGTAGTGGAAATCCCTGTAACTTCATAAAATGAGATGTTTTCTCCTTTTGTGAAATTGTTTTAAAAAATTTGTTTCTTTACTTTCAAATTTCTTTGCAATTGTGCTGTACTGTAATATGTTACAATTGCAGTTGACAAATGTCCGTTTTTGTGATTTTCGATTGTTCTGTTTTAAGTTAGTGCACATTCATTGAACAATATGTTATATCAGCTAAAATATTGTAATAATTCGGTTACTGAAGTTTGAAATCGATTTACTTTTCAGTTATTTTATGAATTTAGTAGCGTGATAATGTGATAATGGATTGGATTTTCCCTCGTATTATATCCAAGATTCTAGTGGTTACCTTCAAATACGTCATCTATGAAATGAAGGGTCTTACTCTGTGTGTTTTAATGATCAAATATATACTATCTTATGAATTTATGGTCGTAATGTTGCTAGTCTTCAAGTCGTGAGAGCTATGAATTTGAAACGATGGTGTGACTCGCATAATCATTTAATTGCCGTGGATGTCTGAGACTATCAGGTTGTAATCTTAAAATTCCAACAGTCGACACATTTACTTAGACAGCTTTTAACCTTTAAGATAACTGTACAAAATTTCAGATGACTAAAAATATATTGACATTATTGTAAACAGATCTTGTAATGTTAAATGCTGCGTTAATTCCAGGGTCGTTTATGAAGGATTTTAATAATTCATTAGACGACCACTTTCGCGCTCAATTGTTCTCCTTTGTTATGACTCACTTATGAGATAGTTTAGTTAGTTGTCGAAAGAAGACGACTCCTATAATAAATTCATGATTGTTGATTTGAAATTTTTACGTCAATGACTACAGTACTACTACATCACATTAGTTGTGTTCAAATCAAAATTGTTAATTGGTACGATTACTTATTTAGATATGAAATAGTGATTTGATTAGCCTTACATTATATTTGTTATATTCTCACTTGAGCCTCTTCGGAGGGACCTTTAAACTTGTGCGATTGGTAATATGTGTGCTTAGAAATACTAGGTTAAAATTCTATTTAATCGACTGAATCATGTACTGGTACTCTACTAAAGCTGAATTATTTTTGCCTTTGCACTCCCAAGTTCGGACGAGTCTCGTGGCAGTAAATGTTGTTGATCGAAGTCCAATGGCCTTTATTTCGAACCTTTGATAGGTATAGTTGGCGTTGCAGTCAGCTTATTAACTTTCGATGATCGTATTATTGCTGGTAAAGTGTTTTACACACGGGCTGCAATCATTATCAAGGATAGGTCCCATTAAAGTGTCTGAAAGTTAAGCAGGACTAAGTCTTGTTAGCAGAAGTGTAACATAGCTACGCAGGATTGTGTTTAAATCAAACTATGCTTCTGCTTGAATTGGCGTTATTTTTTCTCAGTATCCCAATTCTTATAGATTCACGTCTTGCTGTGAGGTGTCATACTACAATGCTGACATTACATGCAAGATGGTAACATCCAACTAAGGTTATAAAATAAGAACTTAGAATTGAAATATAAACCTTTTTATTATTGTCAGTCATGCAATAAAAAAATGAGGAATTATGGAATTAAAAATGGCCATTTACGATCGCGGCTTGTAAGATCATACAGAATTGATTAGTTTCATTACTTAGTGAGACTGGCCATAGAAATATGGTTTTAAAATTTTCGATGAGCAGTTGGGACCGAGCATTGATGTCAATCTACTCAAATGGCCACAGAAACGTGATCTTAAAAATCCACTTTCGATAATCGTTTACATTCTAAGAAAGTCTCGAACATTGCTGAAAGATCCTTAGAATGTCTCAGGCCCTGGAAAAGTCTCATGAGCTTGTTTCAATGCTGGAAGGGAGAGTGTCTCTAGCAATTGCTAATTCCGAGACCGTAGGTAATTCCGGAGAGTCCGAAGAAAATTGAGAGCCTTCTGATTGCCTCGAGATAATTTAGAGATCTTCATCTTTGCTTGAGACTTTCAGAACTTCTCAGTAAATTTAGAGACTTTTAGTAAGAACTTCAAAAGCCAAAGTAGAATATTAGAAGTTGATCTTTTTTTCTATTCTCCTAAAGTTCTACTTTATAACTTAGAGACTAGAAGCTCGTATATGGTTTGGTTTGTTGTGTGATAAGCATGGGAGCATGTGTATCATATTTTTTAGCCAGTGTTTTCTGTTTCTGCTATGTTGATCATACTATTGCGAAAGGTTTTATTTAGATATTTTATTTTTCGCTATCAAATAAATACAGCTATGAATTAGTATAATTATTCGTATGCTGACCAAGCAACTGAAATAACCATTCCTCGCATTAAAAGAGATGTTGTATATCTATTTTGGCAAATAATGGCGAGATTATAAATTATTAGGTCGTCAATGATTCATTCAATTAAAGGTTTCCAAGAAGACTGTACAGTTCAGAATTAACGTCGCATAAAAATTTTTTTAGAAAGGTGGCAAAACATTTAAGACAGCTGTCTTAAACTTCATGTGTACATGTAATTTATCATTGATTATGTTGAACCTATTCTATTAAACACAACAGCTACATATTATTTTCTAGTAATGATTTCTAGT

Protein: 191 aa

>Cdc42_protein

MQTIKCVVVGDGAVGKTCLLISYTTNKFPSEYVPTVFDNYAVTVMIGGEPYTLGLFDTAGQEDYDRLRPLSYPQTDVFLVCFSVVSPSSFENVKEKWVPEIAHHCQKTPFLLVGTQIDLRDDASTVEKLAKNRQKPITCEMGEKLAKELRAVKYVECSALTQKGLKNVFDEAILAALEPPEPPKKKKCVIL

Domains

Cdc42-Rho family GTPase: 3 - 177

Clone

Partial ORF

Primers

Forward: CGTGGGTAAAACCTGCCTAC

Reverse: CCCGTCATCCATAGTTTCGT

Nucleotide# 411 – 1066 = 656 nucleotides

>Cdc42_clone

CGTGGGTAAAACCTGCCTACTTATTTCATACACTACCAACAAATTCCCCTCAGAATATGTCCCCACAGTATTTGACAATTACGCTGTCACTGTTATGATCGGAGGAGAACCTTACACATTAGGATTATTTGACACAGCTGGTCAGGAGGATTACGACAGACTCCGACCCCTCAGCTACCCTCAGACCGATGTATTTCTCGTTTGTTTCTCAGTAGTCTCTCCCTCTTCTTTCGAAAACGTCAAGGAAAAGTGGGTCCCAGAGATTGCCCACCATTGTCAGAAGACCCCCTTCCTCCTTGTCGGCACACAGATAGATTTGAGAGATGACGCTTCAACTGTCGAGAAACTGGCCAAGAACCGACAGAAGCCAATCACGTGTGAGATGGGAGAGAAGCTTGCCAAAGAACTGCGTGCTGTCAAATATGTAGAATGTTCAGCTCTAACTCAGAAAGGACTGAAGAATGTCTTTGACGAGGCTATTCTTGCAGCCCTCGAGCCCCCAGAGCCCCCAAAGAAGAAGAAGTGCGTTATCTTGTAGATCTTCGTCTTATTTCGCAAGGACCAAAATTCAATTTAGGAACTTATATGACACAAACAAACACTGCTTGTTTTTGCTATGGATGATTATGAAAAGAAACGAAACTATGGATGACGGG

Cloning info

PCR rxn on 11/25/15 using GoTaq polymerase mix

| Template | Initial denature | Denature | Anneal | Extension | #cycles | Final extension |
| --- | --- | --- | --- | --- | --- | --- |
| 2 hr cDNA | 4min @ 94C | 30sec @ 94C | 30sec @ 55C | 2min 15 sec @ 72C | 35 | 10min @ 72C |

Purified PCR Product with Thermo Scientific kit on 11/25/15, Second PCR from purified template on 12/1/15

Ligated PCR product into Pgem T Easy vector and transformed into competent cells on 12/1/15

Picked colonies for overnight cultures on 12/7/15

Isolated plasmids using Promega miniprep kit on 12/8/15

Confirmed clone Cdc42 by sequencing on 1/15/16

Cdc42 is sense to T7. RNA probe generated using SP6 on 1/20/16

**ECT2**

Gene model: 5056 nucleotides

ORF: 275 - 3037 = 2763 nucleotides

>ECT2_full

TGGAGAACTCTGGGATATTTTTGTGCGCTCTCGACCAATCAGCGCGCAGATCGCCATTTTGAATGGCCGGTAAATTCAAAAGCGAAAAAGGTATGTGCCGCTGAAGAATTTCACGAAAATATGACGATTAATTTACAAAAGGATAATAATTGGATCGGCGGGACGAAGAACAGAGTTGGTAGAAACTGAGGAACGCTAATTTACAGGCCCTGAGCCTCTAGAAATTTTGAATTTCGCCTGGAAAACTTCCGAGCAAGGATCAAAACTTTAAAAAATGGCGGATTTTAGCTGTCGGAGCAGCACTGCAACAGAAAGCGTTTGTGGACGGGATATTACGGTGGATCCAGAAGAAGATCTAGATGTCCGATTTGTGGTTGTTGGCCAGAAATGCATGAACAATGAAGAACTACACAGAGCTTTGGAATATTACAACTGCCCGGTTGAGCAGTCGGACACTGGTCTGGAGTACCTCCGAGAGGCCCTGGATGATGACACGGTTTTCATCATGGATAATTTTGACGGACAACTTTTCCAACAGTTACATTCTGCTGGTGCTCGCATTATGGGGCCGGCTGTTATTATTAAGAATGCTAACTTGGGCAAGTCTTTGCCCAACAACACACGTCCCTTGTACTGCACACACCTCCGTCCGGTTATTTTGTGTTTCACTGGATTCAAAGTGAAACAGGACTTGGCTGAACTGGCAATGTTGGTTCACCACATGGGAGGTGGCATAAGGAGGGACATCAGTGCCAGGGTGACCCATCTGGTTGCTAATAGTACTTCAGGAGAGAAGTATAGGCTTGCAGTCAGCATGGGGATCAAAATCATGACGGAGGACTGGATCCACAGATGTTGGGAGAACAAAGAGAACCTGGAGTTCAATCCTGTCGATGAGGACATTATCCACAACTACCAGCTGAGCCCTTTTTCTTTCTCCACGTTGAGTTTCTACGGCTTCAGCAAAGAAGAGCAGAGAGACATGGAGGACTTGACCGTAGCTAATGGGGGAACATGTGCGGAACCTGGTGCCCCACAGTGCACACATCTTGTGGTCGACGAACACACTGTAAAGAAAATACCATGTGAGATCCATCCCAAGTTACAGGTCGTCAAATCGGAGTGGTTCTGGGGAAGTATACAAATGGATGCCTGTGCAAATGAACAGATTTACAAATTTGAAGTGCCTCAGTCAGCAGATACACCTTGCGAAAACAGACCAACCAAGTCAACACCCAAGACGTTATCAGGGGGTAAATCAAGAAAGCGCAAACGGTTAAGGGACAACCTCGCACAACTAGCGGCTGAAGGAGAGGTCGATTCTCCCTTTACAAATACTAAAAGACGTTCCAATGAGGTCGGACATATGTCCGTCAGTGGGTCCTTCTTGGATGCCACGGCTGATATGTCTTTACCTGTTAGTACACCAACAGATAAAGATGCAGGAAAGGAGAATGAAAGTACGCCCAAAGGAAATGTGGGGAAGATGAGTCCTCGTTACTTGACAATCAGAGAATTGCTACAAACCGAGAAAAACTACGTTGCTATCATGCAGACAGTATTAAAAACATTCAGAGATGAGATAATCAAACCAGACCAGCCGGGAGGTCCTCTGCTCGACCCCACGGATGTCAAAATGATCTTTGGCAAGATTCCTCCCATTTACGAAGTCCACACGAAAATACGTGATGAACTGGAATCTCTTCTTGTGAACTGGTCCGAGAGTAAATGCGTCGGCCAGGTGTTCTTGAATCATGCCGATGCTCTTATGAAAGCTTATCCTCCATTTGTGAATTTCTTTGAAAATACAAAAGAGACGATTCAACATTGTGATAAAGCGAAACCACGATTCCATGCATTCCTCAAGGTTTGTTTGAGTCGCCATGATTGTTGGAGACAGTCGCTGACTGAGCTGTTGATCAGACCCGTCCAGCGGCTGCCTAGTATTTTGTTGTTACTGAAAGATTTGTTAAAGAGGACGGACCGCAACAACGCGGACCACGCCAACCTGGAGAAAGCCATTGCTACCCTGGAGGAAGTCATGACTCATATCAATGAAGATAAGAGGAGAGCCGAGGGTCATCAGAGAATGTTTGACATCGTCAATGACATTGATAATTGTCCTCCGAATCTTCTTTCCTCACATCGCAGTTTTTCTATGAAAGTTGATGTTGTTGAAGTCACGGATGAATTGAGTGGAAGAGGAGAGAATATCACCCTGTTTGTTTTTAGTGATAGTATAGAGATGTGTAAAAGACGAGTGAAGGTGTTGAACTCGAAAAGTCCTGCCGCCCATGGTGCTAAAACCCCCCAAAAAGCTTACAAACACATAGAAATGGTGCCCCTCACGTTCGTCAAGAGGCTTGTCAATATCACTGATATTCGAGACGAGCGACAAGTATTTGCAATGGTAATCAAAGAGCTGGACAAGCGAGAGAAGTTGTACGCCTTCATGGTGGTAGAAGAAGACTTGGAGGAGAGGGATGAGTTGCTGATGAGGATGGCCAAACATATATGTGCTAACACTTGCAGAACTGACTATGAGAATTTGCTGGCAGATTACGTAGCAGCTGACTTGGACATCGACTTGAATGATTTCCAAAACAACACATTGAGTCGTGCAGCAACAAGGTTTGGCCGTCGAGTGTCAAGGGCATTCTCCTTCAACCGAACACCTCGGAAACTTCGCCGAGCTATGTCATCAATGACGCAAGTGATGTCACCGTTTTCAAGACGAGATAGCATGACGTTTGCCCACCCTGGTAGTGTAAATACAATCACACCAAGAAAAACCGGTCATGAGTTGCGACAGAGAATTGCGTCCACCAACGATTTGACGGACACTGATGACTCACAATTTGGCCTCAACAATATTCCCGAGGATTTCGGAATGCGGCCCCATAGTCCTAAGAGGCGGCGGGAATCTATCGGTGAGTCCTCCGAATCCCCTCTTCCCTCGGCTCCCAGCACCCCTAACTTAGCGTCTTCTATAAACACAACGCCGTGGAAATTCTCCCGTTCTGCAAGCGGCCGGTGATTGACGCTGTCTCCGCTGAAACTAACTGTGCATGAAATTCAAGTAAGACATCAAAACGTGGAAAAACCATTCCAATCGAGTGACTTCTATGGTTCATTGTAACGTAACCAAGAAAACTACAAATTACCATGGACTTAAAATGTTAAGTTATGGTGATAACTCTTTGTAAAATTGTGTAAAATAAATGTTTGTATAGTACATAATTTATGTAGTAGTCCCCTGTTAATCCTGGTATAGCGTCCTTCTAATTGTACTTAAAAAGTTGACTGTCCACTGAACGTGTTACCTGGCGTGTTGGAGAAATGAGCTGTGTATCAAACATGTTGTGAATTGTGTGTTGGTCTGTGTCAATATTGTGACGAGGTGTAAAATGAGGAGCAAGTCCGATTTTTTTCCAAAAAATTCCTAGATTACAAAAATATCTTGTTTCAAATTTTTTCCAAAATATGTTACAAAATTTATTTTTTCTATTAATAATTAACCATTTTTATTCATTATCCAATTTTCTCAGTTGCGAAAAATAGTTTAAATTTGAAAGCTCAGACACTAATGTTTGACAAACATCTTTGATTTCCTTATCCTATCTTAGTTTTCTAAATCATTCTTTGCAAAATCAATCCTGGTAATGCATATTTGTTAATTGGATATATTAGATGTCTCTGTGAGTTATAAGCAATGACTCCGTGAGTCATTTGTGAGTGAAGAATTGACTAAAAATTTCTGATGAATGTGTTGATTTGACGCGCTGGGTAACAATAGAATATGAGTAAAAGCAAAGATTTTTCACTGCAAGATGTACTGTGATGGTCGCCAAAAGATTTCTACATTTGTCATTTGGTAACTAGTGTTTGAGAGTTTTAGTTCTGAACATAAACAAAGACTGGCTGATTTGTCCTACAGACTGACTCAACTCCCCTCCTGATGTTGAAAATTTGAAATTCTTTGAAGTTTTCTATGTTCTCAACTACTGAGAACTTTTTAGACTATATCTCTGATGACTTTTCTCCCTTCTTTCTCCTCTTTCTTTCTTCTTTTCTTCCCCCTAATCCTTACCCCATCCCTCTCCCCCTCTCCTCGGTCATCCCCCCTCACCCTTCCCCCCTTTGCAGTTCTTTGATCAGTTTCATCACTCTCGTTATATCTCCTTTCCATACCCTTGTCCACCTCTTTCCGCCCCCTCCCCCTTCTTCTCCAAAACCAACCCTCTCCCTTGCTCTTATTAGCACCACACACATGCATGTCCAAGTAGCCCCATCACAGCGTCTTGCGGTGAATTAGCTTTGTATAAACATCCGTTGTTGTTGTAGGAGTTTCTTTAATTCCCTCCATTGTAAATCGAGCGAATGTGGCGTTGGAAAGATGTGAGAATGGCGAGTGAATGGTTTCATAAACATAAATCAATGTTTCGTGTATATGTCTGACACTGTCTGTGTGTGTGTAAATTTGTATATATTTCTCCGTTGATTTTTGTAAATACATTTCTTTATTGTATGTATATGGATTTCTCAATGACAAACCTTGCTGTGGACCATACTTTGAGTACTGTACTATACAACATTACTATTCATTTGATGATAATATGATTACATTTGTATATTTAAGTTAAAATTAGATGCTACATATATATATACTTTGCTATGGCGTTCATATATACAATATAGTTTTAACACCAAAGATGTCCTGTATAACATACTATCGTCCGATGTACATTTTGCTTAATGTAAGATTGTGTATCTCTTATATCTTAGATTCCTTATTAAGTAACATTGAGACGGAACAATCGCATATATATTCAGTAGACCTTGCGAAAAGCAATCCTTACAAAAATTGGAGAACTTATCAAAACTTTAAATCTGTTTGGCTCAGCATTCGCAGTCCATTGTTATTATTTTTATTTAAGTTTTAAATTTGCTCCCAATGTCTGATATCCGAGGTTTGTAAATGTTAAAATACAAGCAGAAGGTTGTAATCCAATAAACAGAAATTTATTCTCTTTTATATTAAAAAAAATAGATCGGA

ECT2: 920 aa

>ECT2_protein

MADFSCRSSTATESVCGRDITVDPEEDLDVRFVVVGQKCMNNEELHRALEYYNCPVEQSDTGLEYLREALDDDTVFIMDNFDGQLFQQLHSAGARIMGPAVIIKNANLGKSLPNNTRPLYCTHLRPVILCFTGFKVKQDLAELAMLVHHMGGGIRRDISARVTHLVANSTSGEKYRLAVSMGIKIMTEDWIHRCWENKENLEFNPVDEDIIHNYQLSPFSFSTLSFYGFSKEEQRDMEDLTVANGGTCAEPGAPQCTHLVVDEHTVKKIPCEIHPKLQVVKSEWFWGSIQMDACANEQIYKFEVPQSADTPCENRPTKSTPKTLSGGKSRKRKRLRDNLAQLAAEGEVDSPFTNTKRRSNEVGHMSVSGSFLDATADMSLPVSTPTDKDAGKENESTPKGNVGKMSPRYLTIRELLQTEKNYVAIMQTVLKTFRDEIIKPDQPGGPLLDPTDVKMIFGKIPPIYEVHTKIRDELESLLVNWSESKCVGQVFLNHADALMKAYPPFVNFFENTKETIQHCDKAKPRFHAFLKVCLSRHDCWRQSLTELLIRPVQRLPSILLLLKDLLKRTDRNNADHANLEKAIATLEEVMTHINEDKRRAEGHQRMFDIVNDIDNCPPNLLSSHRSFSMKVDVVEVTDELSGRGENITLFVFSDSIEMCKRRVKVLNSKSPAAHGAKTPQKAYKHIEMVPLTFVKRLVNITDIRDERQVFAMVIKELDKREKLYAFMVVEEDLEERDELLMRMAKHICANTCRTDYENLLADYVAADLDIDLNDFQNNTLSRAATRFGRRVSRAFSFNRTPRKLRRAMSSMTQVMSPFSRRDSMTFAHPGSVNTITPRKTGHELRQRIASTNDLTDTDDSQFGLNNIPEDFGMRPHSPKRRRESIGESSESPLPSAPSTPNLASSINTTPWKFSRSASGR

Domains

PTCB: 127 - 190

BRCT: 221-289

RhoGEF: 412 - 595

PH_Ect2: 586 - 760

Overlapping domains

Clone

Partial ORF

Primers

Forward: GGGACGAAGAACAGAGTTGG

Reverse: ATGTCCGACCTCATTGGAAC

Nucleotide# 160 – 1366 = 1207 nucleotides

>ECT2_clone

GGGACGAAGAACAGAGTTGGTAGAAACTGAGGAACGCTAATTTACAGGCCCTGAGCCTCTAGAAATTTTGAATTTCGCCTGGAAAACTTCCGAGCAAGGATCAAAACTTTAAAAAATGGCGGATTTTAGCTGTCGGAGCAGCACTGCAACAGAAAGCGTTTGTGGACGGGATATTACGGTGGATCCAGAAGAAGATCTAGATGTCCGATTTGTGGTTGTTGGCCAGAAATGCATGAACAATGAAGAACTACACAGAGCTTTGGAATATTACAACTGCCCGGTTGAGCAGTCGGACACTGGTCTGGAGTACCTCCGAGAGGCCCTGGATGATGACACGGTTTTCATCATGGATAATTTTGACGGACAACTTTTCCAACAGTTACATTCTGCTGGTGCTCGCATTATGGGGCCGGCTGTTATTATTAAGAATGCTAACTTGGGCAAGTCTTTGCCCAACAACACACGTCCCTTGTACTGCACACACCTCCGTCCGGTTATTTTGTGTTTCACTGGATTCAAAGTGAAACAGGACTTGGCTGAACTGGCAATGTTGGTTCACCACATGGGAGGTGGCATAAGGAGGGACATCAGTGCCAGGGTGACCCATCTGGTTGCTAATAGTACTTCAGGAGAGAAGTATAGGCTTGCAGTCAGCATGGGGATCAAAATCATGACGGAGGACTGGATCCACAGATGTTGGGAGAACAAAGAGAACCTGGAGTTCAATCCTGTCGATGAGGACATTATCCACAACTACCAGCTGAGCCCTTTTTCTTTCTCCACGTTGAGTTTCTACGGCTTCAGCAAAGAAGAGCAGAGAGACATGGAGGACTTGACCGTAGCTAATGGGGGAACATGTGCGGAACCTGGTGCCCCACAGTGCACACATCTTGTGGTCGACGAACACACTGTAAAGAAAATACCATGTGAGATCCATCCCAAGTTACAGGTCGTCAAATCGGAGTGGTTCTGGGGAAGTATACAAATGGATGCCTGTGCAAATGAACAGATTTACAAATTTGAAGTGCCTCAGTCAGCAGATACACCTTGCGAAAACAGACCAACCAAGTCAACACCCAAGACGTTATCAGGGGGTAAATCAAGAAAGCGCAAACGGTTAAGGGACAACCTCGCACAACTAGCGGCTGAAGGAGAGGTCGATTCTCCCTTTACAAATACTAAAAGACGTTCCAATGAGGTCGGACAT

Cloning info

PCR rxn on 10/19/15 using GoTaq polymerase mix

| Template | Initial denature | Denature | Anneal | Extension | #cycles | Final extension |
| --- | --- | --- | --- | --- | --- | --- |
| 2 hr cDNA | 4min @ 94C | 30sec @ 94C | 30sec @ 55C | 2min 15 sec @ 72C | 35 | 10min @ 72C |

Purified PCR Product with Thermo Scientific kit on 10/20/15, Second PCR from purified template on 11/9/15

Ligated PCR product into Pgem T Easy vector and transformed into competent cells on 11/9/15

Picked colonies for overnight cultures on 11/19/15

Isolated plasmids using Promega miniprep kit on 11/20/15

Confirmed clone ECT2 by sequencing on 12/4/15

**Rho1**

Gene model: 3774 nucleotides

ORF: 293 – 871 = 579 nucleotides

>Rho1_full

TCATGCAATTTCTGTCAAGGCTTCAACAAGGGTCATCTCCAAGTGATCATGATCCTTTGACGCGGGAAGGGAGGGGCACTTTTAAGGCATGTGGCGTCCTCTTGTGGCAGGAAGTGAGTCATGTTCCCAACATGCACTGGGCGTGACGTCACAGAGCCAATTTCTACCAATTGTTGCATCCCCCCTATAGGGCCAATTGAGGTGTGGTCTGACTGGTCGGTCAAAGAAACAACATTGCTGCAAGAATTTCACTGCTTCTATTCTCATATTTTGTCATTGAGCAAGGTGCAAGATGGCAGCGATCAGAAAGAAGCTGGTGATAGTGGGAGATGGTGCTTGTGGTAAAACCTGTCTCCTGATTGTGTTCAGCAAAGACCAGTTCCCTGAAGTCTATGTGCCTACAGTATTTGAAAACTATGTCGCAGACATAGAAGTAGATGGTAAACAAGTTGAACTAGCATTGTGGGATACTGCTGGACAGGAGGATTATGACAGACTGCGTCCACTCTCCTACCCCGACACGGATGTCATCTTGATGTGTTTCAGCATTGACAGCCCCGACAGTTTAGAAAACATCCCAGAGAAGTGGACGCCGGAGGTGAAACATTTCTGTCCCAACGTGCCCATCATTCTCGTGGGCAACAAGAAAGATCTTCGCAATGACGAGAACACACGACGTGAGTTGGCCAAGATGAAGCAGGAACCAGTGAGACCAGAAGAAGGACGGGCCATGGCGGAGAAGATCAACGCTTACCACTACCTGGAGTGCTCGGCCAAGACAAAGGATGGAGTGAGAGAAGTTTTCGAGACCGCAACTAAAGCTGCCCTTCAGACGAAAAAGAAAAAGAAGCCCAAGTGCAGCATTTTGTAAAAGGCCAAGTGATGATAGCTTCAGTGATGCATTACCTTCTTGTTGTACCAATGAGGCATACAAAATATTTGGTAGGTATTCTTAATGCTTGATGTGATAGCGCTGGGCACCGCCTGCCACGTCTACGATTACCATGGACCTCAATGACAATCTTTTGATGGCAAGGTCCCAGCCTGCCTCGGTGAACATCAGCCGAGCAATTGATGCCTCTGGTATTGTGGCTTGTCAACATTTAGGGCTTTTATTTCAACATGTTACGTGGACTTAAATTAATTAACTCTGAACCGAACTTCCTTCAGTCTTTGTGTAGTTATACCAACTGGTGATTCAAGACTTCATTGAAGCACAGGGACTCAATTTTATTGTTTTTAGATTATTTGTTACATTAGCATAACTCTGTCATGAATTTTAATAGCCTACTTGAACTGAATTGGTATAATCTATTTTCTTGATGTTAAACATCAAATTCTGTGGTAATTGCTTTGTACAGTTTGTGAGTTATATTTCAATATTGCTTCAAATGAAAGGATAAGATGAGGCTCCGCTACAGACTGAATGATTGTAAGGAATTCTTGTGTAATATTTTTCTGTATCTAACATGACTGTTTGTCCCACATTTCTACTCATTTCATCTCATTGCTGTCTTCATTAAAACTCATTTGATTTTCTCTCTTGCATATTTTTATGTTCAGTGGACTGTATATGTAGTACTCTGTAAGCCGAGGGCAATTCTGAAGGGTTGATCGGGAGGATCGGTGAATTGCCCTAGTAAACATTCTACTGTTGGCTGTTACATTGCAATATAATATAATCAAAGATGCAATCAAAGATAGGTTGTTCAGACTACACATTGAATTGAGTTCTGTACGAAGTGTAAACCTCCACTATGACCCTTCAGACTTGCCCTCGGCTCAACATTATTGGTGCCACTCAACACAAATTCTGCAGTAGTTGTATTTACTGTATATGTTGCGCTCAATAGGATTGTAGTTTCATCTGACAAGGACAGGAAGGTAGTGAGAAAATCGCGAGTGTCTCTTCAAGACACGAGTGACGACCCATTACACACCCACCCAGTCACACGTACACACCTTATGTAATATACACATGGACTCTCAACCTGCCAAACGTCAAGTATTCAACAGTTTCATGATTTAAGTTGTGGAAGTTTTTATTAGGTGTCGAAATTGCTGGAAATACATGATTAATGTATGTGCTGTCTTAGGTTGGAAGCCAGAAATAGGTAATTGTAATTTTGTAATTGTGAATCAGTGTATTGAATCACTTTGATCTTGTTGCCTTGATCACTGCAGCGGAGGATAGACAGTATTTGTTGAAGGTTTGCGTCTGCACAGCGGCTAATACTCCAGTTGTGCAGACCCTAATCGTTTCTCGCTGAAATTACCATGTCCCAGTGAATCATAGAGTATAAGTGTTGCAGACTATGTCATTACATTTTCTTGATCCAGCCAATGTTTTCCAGTGTTCATTTGCAATGTCTACATTGTGTACTGAATACTGCTGTGACTGTGTAGCAAGTATCAAATAACATGCATCGTACAAGGTGATATTTAACCATGTGGAGAAGTTAGTTTGCTAGTATTTGAGAGGCGCCTGGCTGCAGTTACATGCAGATTGCGAGATGCTCGTTGTTGAGCCTAACCCCTGGGTGTGTCATTTGCTTGATCCCAGAATTTAGTTGATCAGAAATTGTGACAAAGGTCAGTGGTAAAAGCCTTAAATCTTCTTGAGAGCATGGAAAGATAGCCTTTGCCAAATGTTTAAGAATTGACACATCTCGTTGTGTAGTCATCTTAGTGATTTTTCTTACAAATAAAAAAGTATATTCAAATATGTATTTGATGTTCTGTTTTTGATGTTCATTGATTTATTAGTTGAACATGTGTCTTGGTCCTTCTCAATCTGCCTATTGGTCATGCCTCATTGCAAGTGTTCCTTCTCAATTTTCATATGTGTATTGATTGATGGATATTTACTCTGCGGAATCGGATTTCAGAAAATTTACAAATTATTGCTTGACGTGACAAGAATAGTCTCATTTGACCATTTGATAGAAGCACTCTTGTGACGTGACACTAAAAAATTCTTCGATAATATGATTGACAAAATTCGAGTGGATTGAAGGCTACTGTGGACTATTAAAGTTTAATCGATGGACATCAAGGCTTCCTGTGAAATGAGCCAGATGTGTATATATGGCTTGAGTGGAATTTATGCCAGTGATGGCTGGAGTGAAGTTATGATCTAGGAAAATTTGCCAATATGATTAACAAACTTGGGCAATGAAACTGCATGCGGTTGAATTTTGGCATATCTTTGATATAATTTGTTCATGCCAATGGAAAAGGCTATAGGACCCAAGCTGAATTTGTTCGAAGATGAGACAAAGGTGATAAGAACATTACAGGGAAAGATTGCCGCTTGTGAATAGATGTGGCAAAAATAAATTTATGTTAGATTTTTCTGTGGTCACGAAACTAGTGTGTGGCATCTATCTAATCTAACTACAGAATAATAGTATTAATATGGTCATGAACCTCACAAATTTCCTTAATACTTGCGACCAGGTGTGGAAATCGTAAATTAATTCTGAAGGGCATGTGGTGAATGCTCCTTTCATCAGCTTTTAGCAAAATGGCATTATTCCAGAGAAAACAATTCTGAAAGGACAATTGGTCTTGAACGAAGATAGAACAAGTTTGCTACCCTATTGGCTCCGAACTCAGGGAATGGACTTGAAAACTGGCGGGATACTAATCAGCTTGTATGCATGCATTTCCATATTTTATATTATTTACAGCTAAGATTGAATAAGTTTTATGTGCATAAAACCTAACACATTTTTGCCTCACTTTGCAGGTTGAACTAGCATTGTGGGATAC

Protein: 192 aa

>Rho1_protein

MAAIRKKLVIVGDGACGKTCLLIVFSKDQFPEVYVPTVFENYVADIEVDGKQVELALWDTAGQEDYDRLRPLSYPDTDVILMCFSIDSPDSLENIPEKWTPEVKHFCPNVPIILVGNKKDLRNDENTRRELAKMKQEPVRPEEGRAMAEKINAYHYLECSAKTKDGVREVFETATKAALQTKKKKKPKCSIL

Domains

RhoA-Like: 5 - 179

Clone

Full ORF

Primers

Forward: ATAGGGCCAATTGAGGTGTG

Reverse: TTGTATGCCTCATTGGTACAACA

Nucleotide# 187 – 934 = 748 nucleotides

>Rho1_clone

ATAGGGCCAATTGAGGTGTGGTCTGACTGGTCGGTCAAAGAAACAACATTGCTGCAAGAATTTCACTGCTTCTATTCTCATATTTTGTCATTGAGCAAGGTGCAAGATGGCAGCGATCAGAAAGAAGCTGGTGATAGTGGGAGATGGTGCTTGTGGTAAAACCTGTCTCCTGATTGTGTTCAGCAAAGACCAGTTCCCTGAAGTCTATGTGCCTACAGTATTTGAAAACTATGTCGCAGACATAGAAGTAGATGGTAAACAAGTTGAACTAGCATTGTGGGATACTGCTGGACAGGAGGATTATGACAGACTGCGTCCACTCTCCTACCCCGACACGGATGTCATCTTGATGTGTTTCAGCATTGACAGCCCCGACAGTTTAGAAAACATCCCAGAGAAGTGGACGCCGGAGGTGAAACATTTCTGTCCCAACGTGCCCATCATTCTCGTGGGCAACAAGAAAGATCTTCGCAATGACGAGAACACACGACGTGAGTTGGCCAAGATGAAGCAGGAACCAGTGAGACCAGAAGAAGGACGGGCCATGGCGGAGAAGATCAACGCTTACCACTACCTGGAGTGCTCGGCCAAGACAAAGGATGGAGTGAGAGAAGTTTTCGAGACCGCAACTAAAGCTGCCCTTCAGACGAAAAAGAAAAAGAAGCCCAAGTGCAGCATTTTGTAAAAGGCCAAGTGATGATAGCTTCAGTGATGCATTACCTTCTTGTTGTACCAATGAGGCATACA

Cloning info

PCR rxn on 10/19/15 using GoTaq polymerase mix

| Template | Initial denature | Denature | Anneal | Extension | #cycles | Final extension |
| --- | --- | --- | --- | --- | --- | --- |
| 2 hr cDNA | 4min @ 94C | 30sec @ 94C | 30sec @ 55C | 2min 15 sec @ 72C | 35 | 10min @ 72C |

Purified PCR Product with Thermo Scientific kit on 10/20/15, Second PCR from purified template on 11/9/15

Ligated PCR product into Pgem T Easy vector and transformed into competent cells on 11/9/15

Picked colonies for overnight cultures on 11/19/15

Isolated plasmids using Promega miniprep kit on 11/20/15

Confirmed clone Rho1 by sequencing on 12/4/15

**L(2)gl**

Gene model: 6029 nucleotides

ORF: 434 – 4162 = 3729 nucleotides

>L(2)gl_full

GGTCACGTGACTATGCGTCACTTAATGCGGATGAATCTGTTGAAAAGCTGTCAGAGGGTTTTCCCCATTTCTGCCAGACAATAAAATAGTCCCAAATATTCTCCCTGTTCACGTGTAAAGTACGTTATAACAGCACTGGATCGGGGGTATCTTAGCTTTTGACGGAAAATATATAGAATTTCCAAAATTGTTTAGGGTGAATAAGCGGGTGATTTTGACGAGGTCTGGAAAATATAGCGGATGGATTTACTTTGAGTGGTGCAGTGGCCGTCTGCCTGTTCAAGTCTTGAGAAAATTCTCATTGGCGCCATTTCCAACATTGCCACTACTCTGTGGGGAATTTACCCTGTGCTTTTGTGATTATTTATAGGACCCTTTACCTCCTATAAGCAGGATAATCGATTTTAAACAGTAAACTGGCAAGACAACCACAATGTTGAAGAAGTTTGGATTCAAGAAGTCTGAGCCGAGGGAAAGTGAAGCCCGCCTGAAGCTGCAGAAGGAGCTTTTCGCCTTTAACAGGGTCGGAGAACATGGATTTCCTCACAAACCTAGCTCATTTGCTTATGAGCCTAAACTGAAGCTTATTGCTATTGGAACCAAGACAGGAGCTCTGAGAGTTTATGGGGCCCCTGGAGTGGAGTTCTCAGCTGAACACAAAGAAGATGTGGCTGTCACCAAACTGACCTTCGTACCTGAACAGGCAAGAATTATCAGCCAGTGCAGTGACAACTCTCTCCACTTGTGGGAAATTAACAACTCCGAAAATGGATCCGTTCTTGAGGAGATCCGCGAATTCTCAATGGAGGCAAACAAACTCAAGACAATCTCATCCACCTGCCTGACAGCGGACAGTGAGAATCTTCTGATTGGAACAGAGGGAGGAAGCATCTACAAGCTCCACGTGAACACCTTCAAGCTCTCGGAGGACGTCATTTACCAGGACGTTGTCACTCAAAACATCCCTGAGGATTTCAAAGTCAACCCTGGAGCGGTGGAGACATTGAGTTTGCAGCCTGGCAAACCTGATAAGGTACTGATTGGTTACAACAGAGGACTGGTTGTCTTATGGGACATCGCTGAGAGCAATGCTGACCAGACATACAATGCTTCACAGGTTCAGTCAGGATCAGCTGATGATGGATCTGTAGTTAGAACACAACTGGAGTCAGTCTCATGGTACAAGGATGGCAGCCAGTTCATGAGCGCCCATGCTGATGGAAGTTACGTGGTATGGAGCAGCACAGACTCAAGCAAGCCCAAGGAGCAGGCCCAGACCCCTTATGGTCCTTTCCCTTGCAAGGCAATCAACAAAGTGGAATGGAAGGCTGCCAAAGGAGACAACTACATTGTGTTTGCTGGTGGTATGCCCCGACCCAGCTACGCAGACCGCCACACAATCTCCGTGACGCAGGGGACCAAGCAGGTTGTGTTCGACTTCACCTCCAGGGTGGTTGACTTTGCTGTTATTTCAACAGCTGACAGTTTGGAAGATCCGGACAGCACTGAGTTTGATGACCCCCAAGCAGTCTTGGTCTTGGCAGAAGAAGAAATAGTCGGAATTGACCTTCAGTCCGAGAACTGGCCAACCTACAGACCTCCCTATCTGTCCAGCCCCCACTCCTCTGCTATCACTACTACCGCCCACGTGGTCAGTGTCCCTGAACAGCTCTGGAATAAGATTGTAGAAGCTGGTGACAGTCAGACTACACAGTACTCCAGCCGTGACTGGCCCATCACTGGCGGAAATGCTAATATTCCTCCTGTGGAAAGCCGCGATCTTTTGCTCACTGGTCATGAAGATGGCAGTGTGCGTTTCTGGGATGTGTCCTCGGTGGCCATGAAGATGATCTACAAGTTGAACACGGCCGGAGTGTATGGGATGGACTACAGTCAGAATGCGGACGCAGCTGAGGATGGTGATGAGGAATGGCCTCCATTCAGAAAGATTGGGACCTTTGATCCATACAGTGACGACCCGCGCCTTGGCATCCAGAAGATCAGTTTCTGTCCTTTGAGTGAAAGTCTTGTAGTTGGAGGCACTTCTGGCCAGGTTATTGGCTTCCAATTCGAGAGGGAGGAATCTGCCAAAGAGCTGAGCCCAGTGTCAGTCAACATCGTCAGCGACAGAGACAACTTTGTGTGGAAGGGACATGAGTCCTTGAAAGTGGCGGAAGGAGAACAGAAATTTGTGGGAGGATTCCAGCCGACCTGTGTTGTGCAGCTTCAGCCTCCCGCGGCCGTCACTGCTCTGGCTATGCACTCAGAATGGCAGCTGTTTGCCGCTGGAACGGCCCATGGTCTGGCCGTTGTTGACTTCGCCCAGAAGAAAGAGGTCTCCACTAAGTGCACCCTCAACCCCAGTGACCTAACAGGCACTGGAGAGAGTGCCATGTCCCGCAGAAAGTCCTTCAAGAAATCTCTGAGAGAATCGTTCCGTCGTTTAAGGAAGAGGAGGTCAGAAAGAAGGAAGAAAGGAGAGGAGAAAGAGAAGACAGAGTCACCAAAGAAATCCTCAGCTTCAGCTTCAGAGGAAAAGAAGGAAGAGAGCTCACCTGCAGCTACTTCAGTGGAGGAGAAGAAAGAGGAGGGAGCGGAGGGAGTGGCCCCTGTAGCCGAGTCGGAAACAACTCCCACGGAAGCCAAGTCCTCTCCTTCCAAGGATTCCCAGAGATCCACCCCCAAGACCTCCCCGGTCACCACCCCCTCCGAGGGCACTCGAACAATTGAGAGAGCTATCGAAGCTCGCAACACGGACGACAGCATGGCTTCTATGGTCAGATACTTGTACTTCGCAGACACCTTCCTCATCAATGGACAGAGCCACACCCCAACTTTGTGGGCAGGAACCAATGCTGGCGCCATCTATGTGTTCCAAATCAACATGCCTGAAGCAGACAAGAGAGACTCCGGAGAAATCGATTGCCAACTCGGCAAAGAAATCCATCTTAAACACAGAGCCCCCGTTCTGTTCATTGCTGTCATTGACAAGACTGCCACCCCCTTGCCATCTGCCCTTGAGACCCAAAACGGTCGGGCCAAGGATCCTGACATGTCAGGAGGACACTTTGTGTTGATTGTCTCTGAGGAACAATTCAAGATCTTCAGCCTGCCTAACCTAAAGCCCCAGAACAAGTTCAAACTGACAGCCCACGAGGGATCCAAGGTGCGCAGAATGGCCCTGGTTGATTTCCACAGCAAGAGTGATGACAACTACAAGGAGAACGACCTGGTCATCCTCACCAACCAGGGAGACACCCAAGTGTTCACTATTCCTCATCTACGCAGACAACTCAAGTCGCAGGTCATTCGCAAAGATAACATCAATGGCATTGTCTCAGCACTCCTCACCTCCACGGGCGAAGGCTTCTACCCCAATTCTCCATCTGAGTTTGTCCGTTTCTCTCTGTCTCCTCGCCGAGTGTGTCGGGCTAATTGTTCAGTTGAAATCAAGGAGGGAGTTCGTCCCCCTCCCCCAGAGCCCGAACCAGAACCCGAGACCCCAGCAGCCGAAGCAGAGGGTGCGACCGCTGAGCCAACGGCTGATGCAGCCACCGGAGATGCTACAACTGAAGCCACAGAAGGAGCAACCCCAGATGCTGAAACGGCAGTCGCGGAGTCTCAAGAAGGTGGAGCTGCCTCTGAGGGCGACAAAGCTGACGAACAGGCAGCAGCCGATGGTGCTAATGACACCATCGGTGCTAATGACACTATCGCATCTGCCGACCTCACTATCGACAGCGTCAAGTGCCATCTCAGTGAGGACTCGAATGTGACAACCACCTCGTCGCGAGTTGTCTCCTCCAGCATGTCCCGCACGGTCGTCACAAAGAGAGTCGTGACCCAATCATCAGACGAAGGAACTCGAGTCATCTCCTCAGAAACAACCACAGTCACAACAAATGACGGTGATGGCGGAGCAGCCGTGGAAACTACTACTACTACAGCCACTGTTGAAGGATCTTCGGAAGCACCTAGCACCCAAGACCAGAGCCTTGAACAACCAGTAACAAATGGAGTAGAGATGAACGGAGATGCGGAATTGCCAGAACTGAGAATTACGGAGGAGCAGGAGGGCGTTCAGACCACGGACAAAATCAACGTCAGTGCAACCGCAAACGGTCAACCAGCCAGCGAAGTAGTGCAGGAAGCTTCGTCCTAAATATATATGCTACTACACAAAAAATTTTCTAGGTCTATTTTACTACTTTCGAAAATTCAGAACAATTGATGAAATGTTCACAATATTTCGCATTGATAAATGGTGTTTCGCATTTATAGTATGAATAAATGGTTCATAGTTTATTCATGATCTTCATAATATCTTCATAAATACATTATGAATGGAGTTTCTGCCATAGATGACTTATATATATCTAGTTCTATGGTGCATCAGGTCTTGATATTGACTTTGAGAAAATGTCTTATTCCACATTCGCATTTCATGTACAACACTTGTTCAACAGTTAGAAAGATGGTTGACAGTGAGTTGTCTGTTTCTGTTTGTTATGTAGTAGGATAAAATGTTATTGGTTAATGTTGCAATGCATTGCATGTCGTTTCATTGGACTGGTTCGCTAATTTGAAATCATGATAAATGGTTCTCTGTGATCGTTTTGCATGCACTGTGAAATCTGTTTTTGTTGGTGTGTTTATATCTCTGTTTGTACTTTGTTTTAGCTTTGATGCTTTTATCATTGTAGTCCATTACACCAACAAGGGATTGGAATATATACATTACCCTTTCTCACAGATCTGCAATCTCCCCAAGCGGTGAACATGGCAAAAAGAAAGTGGTGTCTGCCATTCTTCTGCTTTTGTAATTCAACTTTTACACTGTTTTTATGCCTTCTACATGTCTCTTTAGTTCAGTTTTATATCAATATTTTAGGTGAAGCAAAATGTAGGACTGCGCAGATGATTGGTGGAGTTGTCAAATTATTCAGTGTGGAAACTGTTAAATATTCTATGTGTACAATTGATCTATAGTACAATAAATATACGTGTAATGTCTCATCATGACACTGGTGTAATGTGAAAGTTTTAACCTGAAGGACTATTAATGATAAGTGATTTGTGTGTTAAATGATCTTGTAAATTATGAATGAAGGTAACAAGGGTTGTATTTGTTTCTGTTAGGGTTGTTGAATGCTGACTGAGACTTTTGTAAATGGTTTAGTTGGTGTTGAGGCCAGTAGTGGAGCACGTCTAGATACACATAGTCCTTTAGAAATACAATGAGAATATTGCAGTGTCACCACGGCTTCTCCCTTTGTTAATTATAGTAGATTTTATATGTACAAGGACAGTAATATATGTTGTATATTATACAAAACACTAAATCATTTATATTATAGGGAGAGCTGATAGGAATTTTATTTTTAACCTGTGTTTAGAGATGATAATTTTGTTTATATACATGTTGTTCCTACAGAATTTTGAATAATGTTAAACTGTCAATGTTTAATGGACTAGAAGTGATGTAACTTCAATATTTGCTGCCGATCTTTGCCTTTTGATGTGCTAACTGCCATGTTGGTCTTAAGCGCCTTTTGTACTTGATCGTGTTCAGCTATGCTACCAGGTGAGACCCTGGGAACTGTTGTTCTAGTGGTTTGCTTAATTTGCATACAAGTGAATAAATTTGCATACATTACAAGAGTACGGCTGGTGGTGGAGCCACCTACAGTTGGGCCACTGAACAGTTGGACTATGTACTGATGTATAGAAAACCTTACAAGTAACTGAGTTTGCAATTTGGTTGCTGATGGTGGTTGCTCGTAGAATCACTGCCTTTGGATGAAACTTGAATTCATTGTCCAGTAACCATTCCATGTCCTCACCATCTTGGACAACTACTCTTTGATATTTGTGCTTTTGTAAGCAAGAGTCGTATCCGTTTCTATTTAGATATATAAGTGTTGGAGCTGCATATTGTAGATTCTCATCTTGAAATTTCACTTTTTAGTACCATGTTTTTACCATGCCATTTGACATGAATGTAATAAAGCTATCCAAATGTAAAAAAA

Protein: 1242 aa

>L(2)gl_protein

MLKKFGFKKSEPRESEARLKLQKELFAFNRVGEHGFPHKPSSFAYEPKLKLIAIGTKTGALRVYGAPGVEFSAEHKEDVAVTKLTFVPEQARIISQCSDNSLHLWEINNSENGSVLEEIREFSMEANKLKTISSTCLTADSENLLIGTEGGSIYKLHVNTFKLSEDVIYQDVVTQNIPEDFKVNPGAVETLSLQPGKPDKVLIGYNRGLVVLWDIAESNADQTYNASQVQSGSADDGSVVRTQLESVSWYKDGSQFMSAHADGSYVVWSSTDSSKPKEQAQTPYGPFPCKAINKVEWKAAKGDNYIVFAGGMPRPSYADRHTISVTQGTKQVVFDFTSRVVDFAVISTADSLEDPDSTEFDDPQAVLVLAEEEIVGIDLQSENWPTYRPPYLSSPHSSAITTTAHVVSVPEQLWNKIVEAGDSQTTQYSSRDWPITGGNANIPPVESRDLLLTGHEDGSVRFWDVSSVAMKMIYKLNTAGVYGMDYSQNADAAEDGDEEWPPFRKIGTFDPYSDDPRLGIQKISFCPLSESLVVGGTSGQVIGFQFEREESAKELSPVSVNIVSDRDNFVWKGHESLKVAEGEQKFVGGFQPTCVVQLQPPAAVTALAMHSEWQLFAAGTAHGLAVVDFAQKKEVSTKCTLNPSDLTGTGESAMSRRKSFKKSLRESFRRLRKRRSERRKKGEEKEKTESPKKSSASASEEKKEESSPAATSVEEKKEEGAEGVAPVAESETTPTEAKSSPSKDSQRSTPKTSPVTTPSEGTRTIERAIEARNTDDSMASMVRYLYFADTFLINGQSHTPTLWAGTNAGAIYVFQINMPEADKRDSGEIDCQLGKEIHLKHRAPVLFIAVIDKTATPLPSALETQNGRAKDPDMSGGHFVLIVSEEQFKIFSLPNLKPQNKFKLTAHEGSKVRRMALVDFHSKSDDNYKENDLVILTNQGDTQVFTIPHLRRQLKSQVIRKDNINGIVSALLTSTGEGFYPNSPSEFVRFSLSPRRVCRANCSVEIKEGVRPPPPEPEPEPETPAAEAEGATAEPTADAATGDATTEATEGATPDAETAVAESQEGGAASEGDKADEQAAADGANDTIGANDTIASADLTIDSVKCHLSEDSNVTTTSSRVVSSSMSRTVVTKRVVTQSSDEGTRVISSETTTVTTNDGDGGAAVETTTTTATVEGSSEAPSTQDQSLEQPVTNGVEMNGDAELPELRITEEQEGVQTTDKINVSATANGQPASEVVQEASS

Domains

WD40: 34 – 269

WD: 447 - 496

Clone

Partial ORF

Primers

Forward: CGTTTCTGGGATGTGTCCTC

Reverse: TGTTACTGGTTGTTCAAGGCTCT

Nucleotide# 1814 – 4012 = 2199 nucleotides

>L(2)gl_clone

CGTTTCTGGGATGTGTCCTCGGTGGCCATGAAGATGATCTACAAGTTGAACACGGCCGGAGTGTATGGGATGGACTACAGTCAGAATGCGGACGCAGCTGAGGATGGTGATGAGGAATGGCCTCCATTCAGAAAGATTGGGACCTTTGATCCATACAGTGACGACCCGCGCCTTGGCATCCAGAAGATCAGTTTCTGTCCTTTGAGTGAAAGTCTTGTAGTTGGAGGCACTTCTGGCCAGGTTATTGGCTTCCAATTCGAGAGGGAGGAATCTGCCAAAGAGCTGAGCCCAGTGTCAGTCAACATCGTCAGCGACAGAGACAACTTTGTGTGGAAGGGACATGAGTCCTTGAAAGTGGCGGAAGGAGAACAGAAATTTGTGGGAGGATTCCAGCCGACCTGTGTTGTGCAGCTTCAGCCTCCCGCGGCCGTCACTGCTCTGGCTATGCACTCAGAATGGCAGCTGTTTGCCGCTGGAACGGCCCATGGTCTGGCCGTTGTTGACTTCGCCCAGAAGAAAGAGGTCTCCACTAAGTGCACCCTCAACCCCAGTGACCTAACAGGCACTGGAGAGAGTGCCATGTCCCGCAGAAAGTCCTTCAAGAAATCTCTGAGAGAATCGTTCCGTCGTTTAAGGAAGAGGAGGTCAGAAAGAAGGAAGAAAGGAGAGGAGAAAGAGAAGACAGAGTCACCAAAGAAATCCTCAGCTTCAGCTTCAGAGGAAAAGAAGGAAGAGAGCTCACCTGCAGCTACTTCAGTGGAGGAGAAGAAAGAGGAGGGAGCGGAGGGAGTGGCCCCTGTAGCCGAGTCGGAAACAACTCCCACGGAAGCCAAGTCCTCTCCTTCCAAGGATTCCCAGAGATCCACCCCCAAGACCTCCCCGGTCACCACCCCCTCCGAGGGCACTCGAACAATTGAGAGAGCTATCGAAGCTCGCAACACGGACGACAGCATGGCTTCTATGGTCAGATACTTGTACTTCGCAGACACCTTCCTCATCAATGGACAGAGCCACACCCCAACTTTGTGGGCAGGAACCAATGCTGGCGCCATCTATGTGTTCCAAATCAACATGCCTGAAGCAGACAAGAGAGACTCCGGAGAAATCGATTGCCAACTCGGCAAAGAAATCCATCTTAAACACAGAGCCCCCGTTCTGTTCATTGCTGTCATTGACAAGACTGCCACCCCCTTGCCATCTGCCCTTGAGACCCAAAACGGTCGGGCCAAGGATCCTGACATGTCAGGAGGACACTTTGTGTTGATTGTCTCTGAGGAACAATTCAAGATCTTCAGCCTGCCTAACCTAAAGCCCCAGAACAAGTTCAAACTGACAGCCCACGAGGGATCCAAGGTGCGCAGAATGGCCCTGGTTGATTTCCACAGCAAGAGTGATGACAACTACAAGGAGAACGACCTGGTCATCCTCACCAACCAGGGAGACACCCAAGTGTTCACTATTCCTCATCTACGCAGACAACTCAAGTCGCAGGTCATTCGCAAAGATAACATCAATGGCATTGTCTCAGCACTCCTCACCTCCACGGGCGAAGGCTTCTACCCCAATTCTCCATCTGAGTTTGTCCGTTTCTCTCTGTCTCCTCGCCGAGTGTGTCGGGCTAATTGTTCAGTTGAAATCAAGGAGGGAGTTCGTCCCCCTCCCCCAGAGCCCGAACCAGAACCCGAGACCCCAGCAGCCGAAGCAGAGGGTGCGACCGCTGAGCCAACGGCTGATGCAGCCACCGGAGATGCTACAACTGAAGCCACAGAAGGAGCAACCCCAGATGCTGAAACGGCAGTCGCGGAGTCTCAAGAAGGTGGAGCTGCCTCTGAGGGCGACAAAGCTGACGAACAGGCAGCAGCCGATGGTGCTAATGACACCATCGGTGCTAATGACACTATCGCATCTGCCGACCTCACTATCGACAGCGTCAAGTGCCATCTCAGTGAGGACTCGAATGTGACAACCACCTCGTCGCGAGTTGTCTCCTCCAGCATGTCCCGCACGGTCGTCACAAAGAGAGTCGTGACCCAATCATCAGACGAAGGAACTCGAGTCATCTCCTCAGAAACAACCACAGTCACAACAAATGACGGTGATGGCGGAGCAGCCGTGGAAACTACTACTACTACAGCCACTGTTGAAGGATCTTCGGAAGCACCTAGCACCCAAGACCAGAGCCTTGAACAACCAGTAACA

Cloning info

PCR rxn on 5/5/15 using GoTaq polymerase mix

| Template | Initial denature | Denature | Anneal | Extension | #cycles | Final extension |
| --- | --- | --- | --- | --- | --- | --- |
| 2 hr cDNA | 4min @ 94C | 30sec @ 94C | 30sec @ 55C | 2min 15 sec @ 72C | 35 | 10min @ 72C |

Purified PCR Product with Thermo Scientific kit on 5/6/15, Second PCR from purified template on 7/29/15

Ligated PCR product into Pgem T Easy vector and transformed into competent cells on 7/29/15

Picked colonies for overnight cultures on 8/7/15

Isolated plasmids using Promega miniprep kit on 8/10/15

Confirmed clone L(2)gl by sequencing on 8/13/15

L(2)gl is sense to SP6. RNA probe generated using T7 on 11/9/15

**Par1**

Gene model: 9547 nucleotides

ORF: 499 – 2214 = 1716 nucleotides

>Par1_full

ATTAAATGAGTGACGTCATTGATCGTCCAGCACCAATCACAATAACCAGCCACATAAGTCTGCGCATGACCGCTGCGCATTCGCCTCTTGCAAGTCGGCCATTTTGTTTCCTCTTGTATGGAAAAATATTTGGATCATGAGAAAGCAGGGCTTTGAATGATTTTCTGGGACTTGAAAATACTCGATGTTGAAGCCAAGGAGTTGGCACATCCGAGGAAGCAGTCTAGACCAGTCCCCAGCTCCTCTAGACTGCAGGATCATAGCTTGAAGTGCTCATGTTCGCCGATGTGCAAGCAAGGGTTTGCTTTGACATTTTGTTACAAAGGCTGCCCGATTTCCAGGGGTCGATCTCAGAACACACAACTCAAATTCCTGCAGTTGCAGCACCGCCTCACAAATGAGCATTTGCTCTATTGCATAATACCCTGCATCCCGGATTTAATTGTGTTATTGTCCTGAAAACGGCCTGGCGAAAAATAACGTCATTTATTATTCAAGATGTCTACCACGAGAGGCCCGCTGCAAACTGTTCACGAATCTCTCACTTCAGACCATGTCGTTAAAACGACTGATGACAATGCCAACCCTCGCATTTCGTCTAGGAGCCGAAGTACTGATGAGCCTCACATAGGAAAATACCGATTGATCAAAACCATTGGGAAAGGTAACTTCGCTAAGGTGAAGTTGGCCAAACATGTCCCTACAGCTAGAGAGGTTGCCATTAAAATAATTGACAAGGCTCAGTTGAATCCATCCAGTCTTCAAAAGTTATACAGAGAAGTGAAAATAATGAAGGTGCTCAATCATCCAAATATAGTTAGGTTGTTCGAAGTCATAGAAACGGAAAAGACACTTTATTTAGTAATGGAGTATGCAAGTGGAGGGGAAGTATTTGATTATTTAGTTGCACATGGGAGAATGAAGGAGAAAGAAGCTAGGGCCAAATTCAGACAAATTGTGTCTGCTGTTCAGTATTGTCATCAGAAGCACATTGTACATAGAGACTTGAAGGCTGAAAACTTGTTACTAGATGGTGATATGAACATCAAAATTGCAGACTTTGGGTTTAGCAATGAGTTCACGCCGGGAAACAAATTGGACACGTTTTGTGGTAGTCCTCCTTACGCTGCTCCTGAGCTGTTTCAAGGAAAAAAATATGACGGTCCTGAAGTAGATGTATGGAGTTTAGGAGTCATTTTGTATACTCTAGTCAGTGGTTCCTTGCCCTTTGATGGCCAAAACTTAAAAGAATTAAGAGAAAGAGTCCTCAGAGGAAAATACCGAATCCCATTTTACATGTCAACAGACTGCGAAAATCTTCTTAAGAAATTTTTGGTTCTCAATCCTCAAAAAAGAGCCAGTTTAGAGACAATAATGAGGGACAAGTGGATGAATATAGGTTATGAAGATGAGGAGCTTAAGCCGTTTATTGAGCCTGAAGCTGATTTTAATGATCCTGTTCGAATAGAAATCATGATCAACATGGGATACTCAAGAAAAGACATCGAAGATTCATTAGTGCAAAATAAATACGATGACATCACGGCGACATATCTATTGTTGGGGAGAAGAAGTAATGAGTTGGAAAGTAGTGAGTCAAGATCAGGGAGTAGTCTCTCATTACGCCAACTGCAGATGACCCATCGGCAGAACAGCGAGATGAATGCCAACAGTTCGCAGTCACCCTCGCACGGAGGCAGCAAGGTCCAGAGAAGCGTATCGGGGTCCAATTCCAAAACCAGACGATCGTCGTTCGGTGACAACAAAACGCCCTCTTCAAAGAACAGTACGTCCCCATACTCGTATGGTGGTGTGCCTTCCAGTGCCTCATACACTAAGCGGTCTTCACAAGGAGTAGACAGTGCTTCCAAAGACAATGCTATCTCGGGTGGGTCACGGTCGAAGGGACCATCGTCAGGCGGGTCAACGCCCACCTCATCCACTTCAGCCGCCTCTTCCATGCCCCAAGGAGAGTCTCCCAAGATTCCTGCTGCAAGCAGTCCTGGCAAAGCGGCCATTCCCAAAAAGCCCCAGTCAAAATCGGGAACGGTGGGAGTAACGCGCAGGAACACTTTTGGTTACGGTGATAATAAGGGTCCTGCCATCGAGCGAACAAACAAAACACAAACACCGACCCCGTCTTCTAACAATTCAGACTTTCGAAGTTCTGCTCGGGCTCCACGCCGGGAACCGTTACAGCGGAGCTTCAGGTGAAAACCACTTGCATTTAAATTAGATTATGTTCGCACTGCACGGTTTTGGGTTAATTCATGGTGCACTGGGATGATTAATGCATCGCTGCTGGTTTCAGTCATTTCTTAATCCTTTTTGCTCTTCAACTCAATCTCTTTCCTCGACTAACTAATCTGCATCAATCTGTTAAAATTAGTAAAGGATCCAAAGAACTAAGTTCTGCTTCAGTATCTGCCCGTTTCTCGGGGCCATACTCTGTATTGAGTCTCATACAAAGCTACTTGTCTGCACTTTTGTCAGTCCTGTGTCATGAGTGGTAGATATGTAAGATGACTTCAAACTCTCCATGAGACAGTTGCTCTCTTAATGCATCCCTTGTCTCTTTACGGCAACCATATCTATTGAATAACTATCTTTTGTTGTGTTGATGAGTTGCTTCATTGTCTTTTCATGAACGAGGTTTGTGTTCTGGAATAAGTCTTGAATGCTTTATGATAAGATAATGAAACTCTGTAGAGCTCTGGAAGTGTATTGAATAAATATGGTATGCTGGATACGATTAAGACAAACTTTCAATAACTTACGAAGCATGTAAATAAGTGATGATTCATCTGTCGCTGTTGAATCAAATTTTTGTTGTCCAAACTCTAAAAACTGGCATTTCATCATGTTGTGATGCTGTCTTAAATTCAACAGGATGATGCAAACTACCCCAAACTTTAAAAAAAAGGGGATAAAAAAAGGGGGAGAACAAAATTTTATGAAAAAAAAGTCAATTAAGTAATGAAACTGTAGCTTCGGTGGTGGTTCGGCAACACTATATGGTAGGTGTAATATGGTATTTAACCAAATACGGTGGAAAGATCATAATCATTCATTAAAAATCAGGGATGTGACATTTTCACTTCTATTTCTGGTCATTCTTGGGAATATAACCAAAAAAAAACTTAGCAGAATCTAGTGTTTCTTCCAATTCTATCCTGTCTTATTTTTCACATCCCTGAAATGTACTTATAGTATAGATATTTAACTTGTATCTCATTCTGTAAAATACTAACCATGATCATCAATAGGAATGCGTTGTATTAGTCACTGACTTCATACTGGAATGGTTTACTGATGAATATTCATTATGAACTATGCGTGCTTGTTTAGTGATAATGATCCCATTTGAATGTGAATCGTGCCATTTTGCAGTGTGGTAATTGTAAGGCCCTAATTAATGATAGTATAAGCCCCAAGCTGTGAAAGCTTTGATTAGGTGTTTTGCATTGCCCAAGATATTGCCCTATGACCAAAGTTAAGCGCAGCCGAACCATTTCAGTATGTAATTTGTTGACGCCAAGTGATGTGCTGTCTGTTTTGGACTGTGCGTCACGAGTCTGTCCCTCATCTAGAATTCTAGATCTGTGAGTCGTCACTGTGAGTCGTCACTCTGCTGTGAGTCGTTCTGTGAATCACTTGTGTCGTGATGACTCGTACTGCTCGCACACATACTAACACTCACCTGAATCACCTCGTTCTGTTCCTTCTGTATTGACATACGTCCTCAATACCCTGTTTAACTTCACTGTTGGTCGTACTAACTGTTGGCCATACCTGTGCCATACCTAACTGTTGGCCATACTAACTGTCAGTCTACTCACTGTCCACACCCTAACTGTTGGTCCACCCTGATTGACTCTTTGCAATGTTATCACTTGTCAGTACGACCTCTTGCATTTTGTGTACTGACTCCTGTACTCTTTGATTCTCTCCAACCAATGCCAACCAATAGCAAGAATAGGAGAAGCTATTCGCAACATGTTGTTTATAGGTATACTATCAGCTTTACCTCCAAACTTTTTCATATATATTTGTTTCTATTTGTCTATTGTGTCTCATTGATTGCGTACTCTCATTTGTACTACAGATTTTGTAGAGAACTTTTATTTCGAGTCATGACCTTCTATATTATCTACATTTGTATATATATCTGAAAGTCTATTCCTCCTAAGCGGGGAATACCCCTCGTTCAGCATGTGTCTGATGTGTCCCCATTCGCCCGGCGAATGGATTTTAGCTCGGCTAGATTTGCTACTAGATGAGAAGAGTACAAGTAGCATGTCGGGGCAGGGATCTGCGCTCATTCTTTTGCTCATTACTTTTCACTCGTTAATCTCATTAACTAATTCATGGTAATCTACATTATCCACTATTTATCATTATCGTTACTCATAATTAGCTGCTCTCGAGCACACACTTGGTCTCATACGAGACCCTAACTTATAGTTTACTCATGAAATCGTCAATTAACTCGGTATTTTGAATTATCATTTTCTTGTCGAATCATTTGACAATTGTTTAATCTGTGACAGGTTTGGTTTGAACTATTTATTTTGTTCTTGAAATATACTGTAAAAAAGTTTTTAAAATGACAATATTAATTGTTTGCAATTAATTGATCGATTGTGAGTAATCAATTTGAAAGTAAAGTAATAATTTATTGACTATAATAAGACATTTGCACTTTGATTTTGATTGTTATGATCAATTTGCTCAAAGACGGGACTTAGAGATAACAAGCTTTGTGAGCATGGGATGGGCTTAAAGTGTGAAAGTTGTGTGGCTTTAATTTGCATGCCTTGTATTTGAAAGTTCTAGCAAAGCTTTTTGTGAATAATTATCTTGTATATTATAGTCTGAACAACAACTACCCTTCTTATTTACACTTAGTCCTTTCTTAAAAGTCATTGCTTTTGCCGTTTTGTCTTTTGCAGTCATTTTTATAGAAGTTACTTTGTGTAAGTCTAGGTTAAATTGTTAGGTTCTAGAATGTTCTGGTGATCTGTCAAATGCTTGTTTAATATGTCGAAGGTAATTAACAATGCATATTGATAATGTTAACAGTTTTAAATCACTAATCTAACAATTATTGAGTCGCACTGATTAATCGAAGGAGTAAGGAGTTACAGATGTATTTCATTGGTTGTTAATGTTATGGGTCTAACCAAGCTCTTGATAATTCTTGATAATACGCCTTGTCGGTTCTAATAATAAACTAAAGTCTTTCCCTTTAGACTTATATTTAATTGTCACTGATACTAAACTGGTGGATATTGTATTTACTCATGCAGACATCCCGAGAAACGTCTGGTGTTCAGTCAGACTACCATGTATACATTCAACTTTATTTCTTACTGTTGAAGCTCTTAGATGTGATCCTAGCATGCCGTTTGCCGTTTTATCTAGCAGAGACATCCCTACCTTTTAACATCGTCAAGCTGGCTAGACTTCGTTCTAGACTTTGTCTATATCACTGGATCTGTTAAAAAAAAATACTAAGACAGTCCTGCCCCTCGCCTTTTTCCATTTAGCGATTTTCTAAAAACTTTTTCTTGGATTTTGCATGCTCGGATTTCTCTCTCTCTCTCTTTCATAGATGTCTTTAAATTTCATCTTGTAGTTACTCGTCTAGGTACTAGAAAATATTGTGATGCAATGGGTTTTATGTGTAAAGCAATGGAGCCTATAATTATCATAATTTAATGTTAAGACGCTAAATTATTTCATAGTGTCAATCACATAGAGCACTGAATTAAATGGTCTATAGTAAATTGCAATAGGATAATCACAGTTGCTGTACATATAATGAGTTAGCATGTAATGTTTGAGGACAGAGTTTTGTTTTTCATTGCGTGCTTTAAATTCAGGCATGTGCACTGTTTGGGGTCGGGTGCCAGGACTCGAGTCTATCAAATGTACGATCTATTAAATGCAATCATGAGCTTAAGCTTTTCCTCTGAAATTTTCTGGCTTCTATTACGCTGAAGTCTTTAAAGTTATAGCATGATTAATGCTGGAAGCAGCCCTGAGCCTCTATCCTTGCAAACCTGCACAGGTCTGCTTTTTTGCGTCATTTCTTTGCCTGTATAGAGACGGGACTAGAAGGGGTTTAGTTTGCCTCGTAGCCCATTTATATATAATGTTAGCAGTCGTTAAGTTAGTAGTATACTAGCTGATAAGCCTCGATGTAATGTTGTTCCGTAGCCGGGGCCGCCGGCCAGGGTGTTAGGTTGGTGGCTGGATTTGTGGCATGTGTTGTGTTTGGCGGTGAGGCATGCAGCCCCTGCTCTAGATCTCTGTCCTCACAATCATCTTGTGGCTGGCTAGCTCAGATATCTCTGTCCCCTCTACTGCTGCTGCTGCTGCCTGGAATGCTCACTGCCTGCTCTCTGGTCTTGTTGTAGTAACATCCCATCCACACCTGCCAGCCAGGCCTCTGACGACCGCCCTGCAGCCCGACCTAAGGGTCACCAGAAATCTGCCAGCACCTCCCATGCTGTTAGAGACTTGGGCCTTCCGCATGCTGAACCTGCTAGTGGGGCTGACCCCCTGCGTCAGACGTAAGTAGCCCTCGCCCAACCTGTAGCCCTCCTGCGCGGTCTCTTTCCCCTATCAAGGGCTGCTTAGCCTTATCGGTCCATCTTCAACATCTTCAACATGTTTCAAGTTGATTTAGAGTCTCTAGTTGCGAGATGTCTCTCTGTCTCGTAGACCCCCACGACCAGCGCCGTTTTGACTCAGCAGTCAGCTACAAGTTGGGGCTCAGTGCTGCCTTATTGTAACCAAACCACACTGCAATTTCACATTCACTTTTAAATGTGACCTACGAAACCTGCTGGCTGGAATCCTTCTTCTCAATCTACTTCTCTCTGTATGGGTTCCTTCGTTTAAATACTTTGCCAAATATAAAGGTCTGAGGTTTTAGCCAAATGCTGTAAGAGATCTGAGGTCTATTGCAAATGGCTTCTGATAGACAAATTCTCGGATGTCCCACAAGTAAACGTCTCAAGTCAGATACCCCAGTCGTGAAATGTACTCTGTTGGAGATTGAGATAGACACCCAATTGTATATGGACTCAATGGGAGATTACCCACTAAGTAAGCTTAAGATGGTGTATAAAATCCTCTCCTCAATGAATAAGATACTGTCCTTCTTTGTCTATAACTCTTGAATCAATGAATCTGACATTTGCATCCCTCGATAAGAGCATGTCATAGAATGACATTAATGATTTCCTCTGGAATGATTCTAGAAACTATCTCAAGTCTGCATCTTGAAGAGATTCAGTGATTATGCTCTGACGTATTCCTTTGAGAGAATTTAATCAATTATAGTGATAACTCCTGTCAATTCCTTACTGAGAGTCACGCTGATACGATAGGCTATTTAAAGCCGATTTGACACATTAAAAGCCGATTTGACACATCGCTAAAGTTACTATATACTGATATGATCTTATGCTTCAGCAGCACCATATAACATGCGTGTAATCCTGAGTGCATGCCCTTTACCCATGCATTAAACGCTGCGATGGTACCCTCATAAACCGATTCACCCATCCTGCCTTTTATTAATCGGATTGAAAGTTGTTGCAGGTCGGATTGTGATCCTTTATAATGTAATCCACGTTGCTGATAACCAATGATAATTGCATTTGTCTAACATTTAATTCTCTTGGATTGGTTCTAGTAGAAGGTTGTTGTATTTACTGCATGCAGGATGCATACGTTTATCTAAAAGTAGGTGGTGGACCGTACTCGTTTGATTTAATAATGACACGTTTCTCTATCGTCATTTCTCTCCGCAGGACGACCAGCTCCTCGCAGAAGTCGTCGGCGATCCCCGCGTCTCCCATGGCAACCAGACAGTTCCCCCGTAGCACCCCAGCACGCAGCACATTCCATGCTGGGCAGGTGCGTGACCGACGGGCCACGACCAATGCCTACAATGGGCCTGGGGGTCTGCCAACACACACGCAGGACACGTCGGCCATGGCGGCTCACAATCGTTTGTCTTTCTTCAACAAAATCACCTCCAAGTTCAGCAGGAGGTACGTACCTGGCCAAGAACTGGAGCCCAACAGGTAAACAGCCAGGCAAACTGCATCATTCATTCGTACTTTTTTTATTATTAAAAATTCTCTGACACCTTGGTACTCTTTGCACCACAGAAAAGAAAATAACCAAGCTTTCATCAATTAGTTGTTTTAGTGTGTAGTGAATCACCATCACCGAGTTCTGAGAAATCACCTTGCCGAGTCTCTGTTCACCGTACTTTTCAAACTATCTTAATATACTTATATCTTTTTGTGTATAAACTTAATGTCTCACAGTGTGTACCTTTTGAGCCACTTTATAATCACGTGGACTTGACTAATTTCCAACTTTCATGCTACTTTTACCCCCATTTTCATTACGCGTGTTTCAGAATTATTTGATCGCAACTAAATTTTTGCTGCCTAAGTGGTTCTGCGCTTAATTCAGGCGTTCAGTTCTCCTTGAAATTGAGTTGTTAATTTGCATTAAAATTCTCGTTGGAAGAAATGCACCTCATCGATACTCAGAAAGGCGGGAATTGAAACTGATGGATTCAACAACTGAAGTCTAATTTTGAAATAAATATATTTGCAAGAGAATTATAATAAATGCAAAACATTGTTCTTCATAATTTTACCAAATATTGGTTGTTTTGTATTCCTGTGTTACCCCCTCATTGGCATTGGGAAATTTGGTTGCCTTTTCTTTTGGCTGCAAAGGACATCATGGTGCCAGAAGCCTTATGCATAAATGTCAAAAGTGTTGGAATTTTGTTAAACAATGTTGTCAGTCTAAGTTCAGTTGGATGTTAATTTTTTTTCTATTTTTTTTTCCTCAAGGAAGTTAGAAGACTATTAAATGAATTTGTCAAAATTCCTCCCATTATTTTCCGATCAGTGCTTCATCAAATTGATTTATCATGGATTTATTTATGATGCAGTTTGCTTTGGTCTTTCCAAGTTGTGAAGGTCTCCAAATGGAAGATATTTTTTTAAAGACTTTGGGCTGTAAAATTTCAAGGACAGTGTTTTTAGGTAACGCAAAAAAATCACAAATTAACAAAATCATCATAACCTGTGCTTCTTTGCCACAAATTTCAACCAAAAACTTTTAAGTCTGCGATATTTTACTATATTGCTGCACGCACTCATTTAGACTTAAGAAATATTATCGAATATCATATGGTGATACCTTCGGAGCACTGTTCATATATTGCATGGAAAGCACTGTTCCTTCAAACTCTTCTGGGTACTGGAATTCTTTCTTTTATATCACCTGCACTGGTTTACTTTACCCCTCACTGCTAGTCAAGATATAGACTGACTTTGGCTTGTGTTCTTCATAATTCTATTAAATCGCCTTTTAAAATTAATTGCACTGATTCGTGGAAATTGCTACGAGATGCTCCAGCGTCCTCAAAAGATTACATTCTCAATGACCATGCATGTCTGCGCATCAAAATTTTATTTTAGAGATCGGAAGAGCGTCGTGT

Protein: 571 aa

>Par1_protein

MSTTRGPLQTVHESLTSDHVVKTTDDNANPRISSRSRSTDEPHIGKYRLIKTIGKGNFAKVKLAKHVPTAREVAIKIIDKAQLNPSSLQKLYREVKIMKVLNHPNIVRLFEVIETEKTLYLVMEYASGGEVFDYLVAHGRMKEKEARAKFRQIVSAVQYCHQKHIVHRDLKAENLLLDGDMNIKIADFGFSNEFTPGNKLDTFCGSPPYAAPELFQGKKYDGPEVDVWSLGVILYTLVSGSLPFDGQNLKELRERVLRGKYRIPFYMSTDCENLLKKFLVLNPQKRASLETIMRDKWMNIGYEDEELKPFIEPEADFNDPVRIEIMINMGYSRKDIEDSLVQNKYDDITATYLLLGRRSNELESSESRSGSSLSLRQLQMTHRQNSEMNANSSQSPSHGGSKVQRSVSGSNSKTRRSSFGDNKTPSSKNSTSPYSYGGVPSSASYTKRSSQGVDSASKDNAISGGSRSKGPSSGGSTPTSSTSAASSMPQGESPKIPAASSPGKAAIPKKPQSKSGTVGVTRRNTFGYGDNKGPAIERTNKTQTPTPSSNNSDFRSSARAPRREPLQRSFR

Domains

STK_c MARK: 46 – 298

UBA MARK: 318 - 357

Clone

Partial ORF

Primers

Forward: GCATCCCGGATTTAATTGTG

Reverse: TACGCTTCTCTGGACCTTGC

Nucleotide# 428 – 1719 = 1292 nucleotides

>Par1_clone

GCATCCCGGATTTAATTGTGTTATTGTCCTGAAAACGGCCTGGCGAAAAATAACGTCATTTATTATTCAAGATGTCTACCACGAGAGGCCCGCTGCAAACTGTTCACGAATCTCTCACTTCAGACCATGTCGTTAAAACGACTGATGACAATGCCAACCCTCGCATTTCGTCTAGGAGCCGAAGTACTGATGAGCCTCACATAGGAAAATACCGATTGATCAAAACCATTGGGAAAGGTAACTTCGCTAAGGTGAAGTTGGCCAAACATGTCCCTACAGCTAGAGAGGTTGCCATTAAAATAATTGACAAGGCTCAGTTGAATCCATCCAGTCTTCAAAAGTTATACAGAGAAGTGAAAATAATGAAGGTGCTCAATCATCCAAATATAGTTAGGTTGTTCGAAGTCATAGAAACGGAAAAGACACTTTATTTAGTAATGGAGTATGCAAGTGGAGGGGAAGTATTTGATTATTTAGTTGCACATGGGAGAATGAAGGAGAAAGAAGCTAGGGCCAAATTCAGACAAATTGTGTCTGCTGTTCAGTATTGTCATCAGAAGCACATTGTACATAGAGACTTGAAGGCTGAAAACTTGTTACTAGATGGTGATATGAACATCAAAATTGCAGACTTTGGGTTTAGCAATGAGTTCACGCCGGGAAACAAATTGGACACGTTTTGTGGTAGTCCTCCTTACGCTGCTCCTGAGCTGTTTCAAGGAAAAAAATATGACGGTCCTGAAGTAGATGTATGGAGTTTAGGAGTCATTTTGTATACTCTAGTCAGTGGTTCCTTGCCCTTTGATGGCCAAAACTTAAAAGAATTAAGAGAAAGAGTCCTCAGAGGAAAATACCGAATCCCATTTTACATGTCAACAGACTGCGAAAATCTTCTTAAGAAATTTTTGGTTCTCAATCCTCAAAAAAGAGCCAGTTTAGAGACAATAATGAGGGACAAGTGGATGAATATAGGTTATGAAGATGAGGAGCTTAAGCCGTTTATTGAGCCTGAAGCTGATTTTAATGATCCTGTTCGAATAGAAATCATGATCAACATGGGATACTCAAGAAAAGACATCGAAGATTCATTAGTGCAAAATAAATACGATGACATCACGGCGACATATCTATTGTTGGGGAGAAGAAGTAATGAGTTGGAAAGTAGTGAGTCAAGATCAGGGAGTAGTCTCTCATTACGCCAACTGCAGATGACCCATCGGCAGAACAGCGAGATGAATGCCAACAGTTCGCAGTCACCCTCGCACGGAGGCAGCAAGGTCCAGAGAAGCGTA

Cloning info

PCR rxn on 10/19/15 using GoTaq polymerase mix

| Template | Initial denature | Denature | Anneal | Extension | #cycles | Final extension |
| --- | --- | --- | --- | --- | --- | --- |
| 2 hr cDNA | 4min @ 94C | 30sec @ 94C | 30sec @ 55C | 2min 15 sec @ 72C | 35 | 10min @ 72C |

Purified PCR Product with Thermo Scientific kit on 10/20/15, Second PCR from purified template on 11/9/15

Ligated PCR product into Pgem T Easy vector and transformed into competent cells on 11/9/15

Picked colonies for overnight cultures on 11/19/15

Isolated plasmids using Promega miniprep kit on 11/20/15

Confirmed clone Par1 by sequencing on 12/4/15

Par1 is sense to T7. RNA probe generated using SP6 on 1/20/16

**RacGAP**

Gene model: 3715 nucleotides

ORF: 181 – 2109 = 1929 nucleotides

>RacG_full

GCATAAGTCAACAATTTGTAAACTTGTCTGTGATTGGTCGAGTTCAAAACTGAGGAACCGCTGGATTTGAATGATTTGGGAAGACCGCACCTGCATTGTTTTTCGTGGTATTTTCAGATATTGATTGTGTTGGATCCCATCATTGTATGCACCTTGGCTCAATAAGGTCTATCATTTACGATGGGTGACTATCAACTCACTCTGGTGGCTGAGTATGATGACATCGTAAGAAACAATGCTATTTTGACAGCGGGGATTGAAACAGAGTTTCTGCGGTTTTTGCAAAGTCAGGAGACTTGTCGTAAGCGCTGGTTGGAAAGTGAAGATGGAGCCAAGGAAATGAGAAACCGTCTCAAGAATCTGGAAAACGAAAACGCTACACTCACAACCAGACTGAGACACGCCAGAAGTACGATCGATAACGAGATAAAGAAGAGGATGAAAGCCGAGCAGGATAAAGAAAGTTTGGAACGTCAAATTGCCATTGTCAGAGAGCTGTTGACTGAGAAGAACAACCAGACAATGCTGAACCAGAGAGATCGTGAGAGACTTGCTTTCCTCAGCTCAACCTGTTTGCCGTCTGTTCCAGCAAACTCGCCCAAGAAGAGCATGTCAAACATTGACAAGTCCGCATCCAATCTGTCTGATTGTGACATCAGTTTTGATGTGACGGGTGAGGACCTTGATGAAACGAGAACTCGTAGTGGAAGACGCCACAAGAAACGCCCCTCAGCTCCCCCTATGGAAGAAGACGATCTCGATACTACTCCACCCGGGAAAAAGGCTAAACGAGATGCACCAGAAAAGCCAAAGCGTTCAAAGCACAGAAGGTCCAAGTCGGCTGTCATTGCAACGGCAACCTTGTCAGTCCAGGACAATGGCGAGATTGAGGCAGACACCCACATCAAGACCCCAGACAGACCTCGCCGTTCCGGCCGCATGAGGAAGATCCACTCTGCTAAAGATGTCAAGGAAATCGCAAAAGAAAATGGGGGTGATTCTGATGATTCTACCATGCGATCTCCCGGTGGTGTCTTCACTCCTGATCGTTCACCACTCCGTCAAATGTCATCAATCGGCAAAGGTCTGAATCGTGCTCATGATTTCGAAACGAAGACAATTATCTGGCCTGAGACTTGTATTCCATGCGGAAAGAAAGTGAGATTCGGCAAGGAGGTGTTGAAGTGCAGAGATTGTTTCACCACTGCCCATGTCGGTTGCCGTGACGATGTTCCTCTACCGTGTGTCCGGGCCAGCGTTCCTAACAGCAGCTCCAGGAGCGCTGCTGCAACATTGTCAGATTTCATCGCAACTTCAGAGCCTCCGATGATTCCAGCGTTGGTGATCCACTGTGTGAATGAAGTGGAGAAGAGAGGACTTGACGAAGCAGGACTGTACAGATTGGTTGGGTCTAAGACTGAAATACGAGCTCTCAAAGACAAGTTCTTACGAGGTCGAGGAACGCCTAACTTGGCTCACATCAACGATGTCAACGTTATTACTGGTTGCTTAAAGGACTTCCTCATGCACCTGAAGGAACCCATCATTACGTTTGCCCTCTGGAAGGATTTTGCTAATGCAGCTGAAATGCCATGCGAGGAAGACAGTGTCAGTTCCACCTACCAAGCAATCAGCCAGTTGCCCCAGGCAAACAGAGACACCCTTGCTTTCTTAATTCTTCACTTACAAAGGGTTGCCCAGAGTCCAGACTGCAAAATGCCCCAGCAGAGTCTGTGCAAGGTCCTAGGTCCAACTCTTGTGGGACATTCGATAGCTGACCCAAGCCCAATGCAACTGCAGGCCGAGATGGTCAAGCACTACAAGGTCATGGACCGATTGATGTCCCTCAGCGGAGACTACTGGGACAATTTCATCTGCGCAGGCGACAACTTGACCTCAATGGGTCAGAACATCATCTCAACACCTCAGACCATCATCTCAACACCTCAGACGCCTGAATCCATGAAAGACCCTGGTCTTCACAGCATGCTTTATCCCCAAGATTCCACAGAAAGGAAGAACTCAACACCCCGATTTGGACTTCACAGGGGTCGTCAGATGCCCCAAAGAAATCCGAAGACGTTCTTCCCTCCGCCTAACCACTATTAATTAATTAAATTTACAAGGACATTGAATTGCATAAACTTTTTACAAGCATATATTTATTTGCAAGTCGGAGAAATATACAAGACTTTCGTATGCTGTGTGTTGTGCTGTACATAGGAGGCTTTTACTGTCTTTCTGAGCTCTGGGTTTCTGTGTTTGGAGAACACTTCTGCTTAGCTAGGCCTGAGATATATTATGTTATATACTTAACTTCTTTTAACTGAGTAATCTTTTATTATGCCCTTGTAAGTAATTTGTATAGTCCCACAAAATTACAATGGAATGTGAAATGTAATAGTTATTCCGAACATTTTCTCCCATGGATGAGACCAGCTGCCTTATTTTGGAATATTTCCACAAGAGGAGTGTATAAATGGACAATACATATTGCCGCGTTTCTATAAAGAATACTTACTAGCGGAAAGAGAAACCGAATGCAAAGGAATAATCTACAAAAATAATTGAATAGGTTATTCTTCACTTCAAATTATACTCTTGTTAGTCTTGGATGCATATTAAAGCAACCTAAAGAACTATAATTGTTTCTGATTATATATGAGATAATAATGACTATTCTGTGTGATTGTAAATATTACCCTATGCAGACTACTATTGTACTTTGTTAGCCTAATCAGGATACTATTGAAATAGTATGTCATAATATGTTTCATTAACTTATCAGGTTAAAGTGCAGGCTGAAAATTAAGCTTTAAATGTGTGAAATTAAGCTGACTAATAGTGTAATTATACAGACTGCATATCATAACATGATGGTTTTAATATTTGTACTATAGTGATTAGAGTTTTATTATGTGACCATGGATCTGATTATTATTCCGAAATCATTGTAATACAATTGTACAATATTTCACAAGTGACGCAAGCCCTCAAAAAGCCTGTTTTAAGGATTTTGGACTTTGCATCAAAATTAGAACATAATTGGGTTCTACAGTTTATGAATTTTTAACCTTCCGTATTGTTGTGGTTGGTAAATATATTATTACTATGGAATTTATATTTTTGTAATAATAGTGGTACCTCACCTCAAATTTAGGCTGCTAAAAATGTTTTGCTATATTTGCTTCTTAGCTGTTTTTCTTCGCAACGGGTTTAGATGAAAAAAAATTTCAATTCATTCCAAATAATCTTTGCAATTATACTGAATAACTGCTGATTTGTAAATTAAAAGTTACATTAATGTGTGTATATAAATCTGTTCTATTTATATTGCATAGGATTTTTTTGGGTGAGGTACCACAAGTCAAATTGTGTGCGAGTTGGGTGCTTGTGACTGCTGAAAGTTTGGTATGATTGTTTCCTGATAAAAAGAAGAAAAGAAAAACACATAGAAATAAAGGCTATAAATAATTTTTCCCCCAAACTTGCAATATCATTATAACAAGTTAACAATGATTATAATAACAAATTATTCATTATTTTCCTCACCTGGGCCCTTAGTCCATTTCCACTGAATATTATTTCTTGTCCATTTAAAAAAGAAGGGTTTCTTATTAAAATCAAATTAGGCCTAGATAATTGCATTTATATAAATTTATGAACAACTATGAAATTATGTTACACAATCCACAAATAAAATACCTTGTCACAA

Protein: 642 aa

>RacG_protein

MGDYQLTLVAEYDDIVRNNAILTAGIETEFLRFLQSQETCRKRWLESEDGAKEMRNRLKNLENENATLTTRLRHARSTIDNEIKKRMKAEQDKESLERQIAIVRELLTEKNNQTMLNQRDRERLAFLSSTCLPSVPANSPKKSMSNIDKSASNLSDCDISFDVTGEDLDETRTRSGRRHKKRPSAPPMEEDDLDTTPPGKKAKRDAPEKPKRSKHRRSKSAVIATATLSVQDNGEIEADTHIKTPDRPRRSGRMRKIHSAKDVKEIAKENGGDSDDSTMRSPGGVFTPDRSPLRQMSSIGKGLNRAHDFETKTIIWPETCIPCGKKVRFGKEVLKCRDCFTTAHVGCRDDVPLPCVRASVPNSSSRSAAATLSDFIATSEPPMIPALVIHCVNEVEKRGLDEAGLYRLVGSKTEIRALKDKFLRGRGTPNLAHINDVNVITGCLKDFLMHLKEPIITFALWKDFANAAEMPCEEDSVSSTYQAISQLPQANRDTLAFLILHLQRVAQSPDCKMPQQSLCKVLGPTLVGHSIADPSPMQLQAEMVKHYKVMDRLMSLSGDYWDNFICAGDNLTSMGQNIISTPQTIISTPQTPESMKDPGLHSMLYPQDSTERKNSTPRFGLHRGRQMPQRNPKTFFPPPNHY

Domains

ERM: 46 – 125

C1: 307 - 355

McgRacGAP: 369 - 561

Clone

Partial ORF

Primers

Forward: CAACTCACTCTGGTGGCTGA

Reverse: CCTGCTTCGTCAAGTCCTCT

Nucleotide# 193 - 1391 = 1199 nucleotides

>RacG_clone

CAACTCACTCTGGTGGCTGAGTATGATGACATCGTAAGAAACAATGCTATTTTGACAGCGGGGATTGAAACAGAGTTTCTGCGGTTTTTGCAAAGTCAGGAGACTTGTCGTAAGCGCTGGTTGGAAAGTGAAGATGGAGCCAAGGAAATGAGAAACCGTCTCAAGAATCTGGAAAACGAAAACGCTACACTCACAACCAGACTGAGACACGCCAGAAGTACGATCGATAACGAGATAAAGAAGAGGATGAAAGCCGAGCAGGATAAAGAAAGTTTGGAACGTCAAATTGCCATTGTCAGAGAGCTGTTGACTGAGAAGAACAACCAGACAATGCTGAACCAGAGAGATCGTGAGAGACTTGCTTTCCTCAGCTCAACCTGTTTGCCGTCTGTTCCAGCAAACTCGCCCAAGAAGAGCATGTCAAACATTGACAAGTCCGCATCCAATCTGTCTGATTGTGACATCAGTTTTGATGTGACGGGTGAGGACCTTGATGAAACGAGAACTCGTAGTGGAAGACGCCACAAGAAACGCCCCTCAGCTCCCCCTATGGAAGAAGACGATCTCGATACTACTCCACCCGGGAAAAAGGCTAAACGAGATGCACCAGAAAAGCCAAAGCGTTCAAAGCACAGAAGGTCCAAGTCGGCTGTCATTGCAACGGCAACCTTGTCAGTCCAGGACAATGGCGAGATTGAGGCAGACACCCACATCAAGACCCCAGACAGACCTCGCCGTTCCGGCCGCATGAGGAAGATCCACTCTGCTAAAGATGTCAAGGAAATCGCAAAAGAAAATGGGGGTGATTCTGATGATTCTACCATGCGATCTCCCGGTGGTGTCTTCACTCCTGATCGTTCACCACTCCGTCAAATGTCATCAATCGGCAAAGGTCTGAATCGTGCTCATGATTTCGAAACGAAGACAATTATCTGGCCTGAGACTTGTATTCCATGCGGAAAGAAAGTGAGATTCGGCAAGGAGGTGTTGAAGTGCAGAGATTGTTTCACCACTGCCCATGTCGGTTGCCGTGACGATGTTCCTCTACCGTGTGTCCGGGCCAGCGTTCCTAACAGCAGCTCCAGGAGCGCTGCTGCAACATTGTCAGATTTCATCGCAACTTCAGAGCCTCCGATGATTCCAGCGTTGGTGATCCACTGTGTGAATGAAGTGGAGAAGAGAGGACTTGACGAAGCAGGACTGTACAGATTGGTTGGGTCTAAGACTGAAATACGAGCTCTCAAAGACAAGTTCTTACGAGGTCGAGGAACGCCTAACTTGGCTCACATCAACGATGTCAACGTTATTACTGGTTGCTTAAAGGACTTCCTCATGCACCTGAAGGAACCCATCATTACGTTTGCCCTCTGGAAGGATTTTGCTAATGCAGC

Cloning info

PCR rxn on 10/19/15 using GoTaq polymerase mix

| Template | Initial denature | Denature | Anneal | Extension | #cycles | Final extension |
| --- | --- | --- | --- | --- | --- | --- |
| 2 hr cDNA | 4min @ 94C | 30sec @ 94C | 30sec @ 55C | 2min 15 sec @ 72C | 35 | 10min @ 72C |

Purified PCR Product with Thermo Scientific kit on 10/20/15, Second PCR from purified template on 11/9/15

Ligated PCR product into Pgem T Easy vector and transformed into competent cells on 11/9/15

Picked colonies for overnight cultures on 11/19/15

Isolated plasmids using Promega miniprep kit on 11/20/15

Confirmed clone RacGAP by sequencing on 12/4/15

**Gai**

Gene model: 2887 nucleotides

ORF: 499 – 1596 = 1098 nucleotides

>Gai_full

CTTTCAGTTTGAGATATTCTTCATGTTCTCTCTTCTCCTGCTCTTCTTTCAATAACTTCTCTTCTTCTTCTTTTCTCGCTTGTTCTGCTTCTTCCTTTTCTTCTTCTTTCTTTTCTTCCGCAACATTTTCTACTTTCTTCTCTGCTTCAGGAGCAGCCTGTGCTGCTGCATTTTCCTCAGCAACTCTGCCACTGTGCCACATCACATGGCAAGTGCCCTTTAAAGTTCTTGAGAAGTTTGCATGCAATGACAGTTTGGCGGTTTGTTCCGCTAGAGTGCGCACCTGCGTAGCAAAGCGAATTCGCCCCTCACTCATCCGGGCATTCAGTTTAAAATCTCAGAGTAGGAGAGAGTTGACCGGTGTGTTAGGAAACGCTGGTTAATTGCAAAATTGTTGGATTATTTGAGTGTTTTCAGGTGATTTGAAGCAACAGACATTTACCTGAAGTTGTTGCGAAGGAGTGGAGCAGCGTTACACAAGGCATAGCCGAGTATTTAATGGTTAAATCGATCTTTAATTGCAAATTCAGAATGGGCTGTGCAGTAAGCTCAGACGATCGGGCTGCCATCGAAAGATCAAAGGCAATCGACAAATCTTTAAGACAGGATGGAGAAAAAGCCTCCAGAGAGGTCAAGCTTCTGTTATTAGGGGCTGGAGAGTCTGGGAAAAGTACCATTGTGAAACAGATGAAGATTATTCATGAAAAAGGTTATACCCAGGAGGAATGTCTACAGTATAAGCCAGTGGTCTATAGCAACACCATACAGAGTATGATTGCTATCATTAGAGCCATGGGCAACTTGCGAATTGATTTCGGCCATGGGGACAGAGCTGAGGATGCTCGCAATTTGTTTGGTTTAGCGGGAACAGCAGACGAAGGAGAACTGACGACGGAACTAGCACACATCATGAAGAGGTTATGGAAAGACTCAGGAGTCAGGGAATGCTTCGGCAGGTCTCGGGAGTACCAGTTGAATGACTCGGCAGAATACTATTTGAATGCGCTGGATCGCATTTCACAGCCTGGATATGTTCCAACAGAACAGGACGTTCTTCGCACCCGAGTCAAGACAACAGGAATCGTAGAAACACACTTCACATTCAAAGACTTGCATTTCAAGATGTTCGATGTTGGTGGACAGCGGTCGGAACGAAAGAAGTGGATCCACTGTTTTGAGGGAGTCACGGCAATCATCTTTATTGTAGCTACGAGTGAATACGATCTTACGCTGGCCGAGGATCAAGAAATGAATCGAATGATGGAGAGTATGAAACTGTTTGACTCCATCTGCAACAACAAGTGGTTCACAGACACGTCGATAATCTTGTTTTTGAATAAGAAAGATCTGTTTGAAGAAAAAATCAAGAAGTCTCCGCTTACTATCTGCTTTCCAGAATACTTGGGTGCCAACACATTTGACGAGGCTGCTGCCTACATTCAGTTGAAGTTTGAAAATTTGAACAAACGAAAGGACACAAAAGAAATCTACACACACTTCACATGTGCCACCGACACAAATAACGTCCAGTTTGTGTTCGATGCTGTTACCGATGTCATTATCAAGAATAACCTCAAAGACTGTGGACTATTCTAATCAAAACTTCAAATGCTCTTGTTATTTATATCTGAAATATGGTACAGTGTTGTTACGAACCCTCCGATGTTCATGAGCTTTTTGAGAAGCTCTCTGAGAGGCCAGGGCTCATCGCAGAGTGACTTTATATTTCGCGATGAATCCAATACATGCACAAGACGGGCTGTCCTCGTCTCCGAAACTACACATCAAAATTTCGCCCTTTTAACGGACTATACAACTGCCAGTTTCACGCTTACGGACAAGCTTACCCTGTCTCCAAACTGACAAATCATCAAAATGACGCTCGTCCGAGTCCGGACTGCTACTTGGGACGATCTTCAGTCTCACCTTACTACATAACTATCATCAATTTACTGCCAGATTATACACAGCTCGATGCGTGATTTATCAAATACATAGCGGATCAGTTGTCAGTCAAGTGGACCAATTCTTAATTTAACGGACCAGTCGTCAAGTTTACGGACCAATTTGTTTCGATTTTGTGGACCAGTATTTTTTGCGGCTTCAAGCTTTGATATCATTCTTTTACTCTGATTTACATTTAAGATATTACGTATACATCTTTAGTACTTACATTGTATTAGATTTATTTCACATTCCGAAGCTTTTTTCCCCCTTTTATACTCCTAAAACAAAGTACACAATCACTGATTACAAAAATGCGTATATTTTGGATAACTTGCATGAGTAATCTAATCGCTTTGAAACAGTAATTGCATTTCTGTTGATACCTTTAATGTACAATGTAAGTGATTTTTGTATTCATGTATGTTACATTTCATTCATTTGCATTAATATGGAATGCTAAAATATCAATTATATCTACCTACCGATTTATATCAAATTTATTATCAATTATATTATTACTATCAAATCTATGTTAAACGAGTATCATTCATATAATTTGTATGATTATTTTGATAAGTCTAATATCTATATCTATATAGATCTCCATACAAACATAATGATAATATTGAAATGAAAGATTAGAAATTTCCTGGCATATGGATAGCAAGCATTTTCTAGGAATCAATAGTAAATTGTAACATAGTAAAGGGCATTTAAGTCTCGCATTTTAGTCTCTCAATGACATTTTAACGGTGACCTATTATCTCAATGATAGGGCTGTGCCGATGCGACGATTATCGCCGACAATCGTCGCCAAAAGTTTCGATTTCGATCATCTCCACAAAATAAGTAAAAATCGATTTTCAGCTGAATGAAGAATTCATGATACATGGCTGTATTTGGTATCGTTTTGAAGCTCTCGAGGAGCTGAACACCACCG

Protein: 365 aa

>Gai_protein

MVKSIFNCKFRMGCAVSSDDRAAIERSKAIDKSLRQDGEKASREVKLLLLGAGESGKSTIVKQMKIIHEKGYTQEECLQYKPVVYSNTIQSMIAIIRAMGNLRIDFGHGDRAEDARNLFGLAGTADEGELTTELAHIMKRLWKDSGVRECFGRSREYQLNDSAEYYLNALDRISQPGYVPTEQDVLRTRVKTTGIVETHFTFKDLHFKMFDVGGQRSERKKWIHCFEGVTAIIFIVATSEYDLTLAEDQEMNRMMESMKLFDSICNNKWFTDTSIILFLNKKDLFEEKIKKSPLTICFPEYLGANTFDEAAAYIQLKFENLNKRKDTKEIYTHFTCATDTNNVQFVFDAVTDVIIKNNLKDCGLF

Domains

G-alpha: 45 - 359

Clone

Full ORF

Primers

Forward: GAGTGGAGCAGCGTTACACA

Reverse: GAACATCGGAGGGTTCGTAA

Nucleotide# 460 – 1668 = 1209 nucleotides

>Gai_clone

GAGTGGAGCAGCGTTACACAAGGCATAGCCGAGTATTTAATGGTTAAATCGATCTTTAATTGCAAATTCAGAATGGGCTGTGCAGTAAGCTCAGACGATCGGGCTGCCATCGAAAGATCAAAGGCAATCGACAAATCTTTAAGACAGGATGGAGAAAAAGCCTCCAGAGAGGTCAAGCTTCTGTTATTAGGGGCTGGAGAGTCTGGGAAAAGTACCATTGTGAAACAGATGAAGATTATTCATGAAAAAGGTTATACCCAGGAGGAATGTCTACAGTATAAGCCAGTGGTCTATAGCAACACCATACAGAGTATGATTGCTATCATTAGAGCCATGGGCAACTTGCGAATTGATTTCGGCCATGGGGACAGAGCTGAGGATGCTCGCAATTTGTTTGGTTTAGCGGGAACAGCAGACGAAGGAGAACTGACGACGGAACTAGCACACATCATGAAGAGGTTATGGAAAGACTCAGGAGTCAGGGAATGCTTCGGCAGGTCTCGGGAGTACCAGTTGAATGACTCGGCAGAATACTATTTGAATGCGCTGGATCGCATTTCACAGCCTGGATATGTTCCAACAGAACAGGACGTTCTTCGCACCCGAGTCAAGACAACAGGAATCGTAGAAACACACTTCACATTCAAAGACTTGCATTTCAAGATGTTCGATGTTGGTGGACAGCGGTCGGAACGAAAGAAGTGGATCCACTGTTTTGAGGGAGTCACGGCAATCATCTTTATTGTAGCTACGAGTGAATACGATCTTACGCTGGCCGAGGATCAAGAAATGAATCGAATGATGGAGAGTATGAAACTGTTTGACTCCATCTGCAACAACAAGTGGTTCACAGACACGTCGATAATCTTGTTTTTGAATAAGAAAGATCTGTTTGAAGAAAAAATCAAGAAGTCTCCGCTTACTATCTGCTTTCCAGAATACTTGGGTGCCAACACATTTGACGAGGCTGCTGCCTACATTCAGTTGAAGTTTGAAAATTTGAACAAACGAAAGGACACAAAAGAAATCTACACACACTTCACATGTGCCACCGACACAAATAACGTCCAGTTTGTGTTCGATGCTGTTACCGATGTCATTATCAAGAATAACCTCAAAGACTGTGGACTATTCTAATCAAAACTTCAAATGCTCTTGTTATTTATATCTGAAATATGGTACAGTGTTGTTACGAACCCTCCGATGTTC

Cloning info

PCR rxn on 11/23/15 using GoTaq polymerase mix

| Template | Initial denature | Denature | Anneal | Extension | #cycles | Final extension |
| --- | --- | --- | --- | --- | --- | --- |
| 2 hr cDNA | 4min @ 94C | 30sec @ 94C | 30sec @ 55C | 2min 15 sec @ 72C | 35 | 10min @ 72C |

Purified PCR Product with Thermo Scientific kit on 11/25/15, Second PCR from purified template on 12/1/15

Ligated PCR product into Pgem T Easy vector and transformed into competent cells on 12/2/15

Picked colonies for overnight cultures on 12/7/15

Isolated plasmids using Promega miniprep kit on 12/8/15

Confirmed clone Gai by sequencing on 1/15/16

Gai is sense to T7. RNA probe generated using Sp6 on 1/20/16

**NuMA**

Gene model: 6582 nucleotides

ORF: 494 – 5566 = 5073 nucleotides

>NuMA_full

GTCTTCTCAGATTGAGGTTCAGACATCACAAATTCAAGAGCAGTCATCTAAAATCGAAGAACAGTCTTCTCAAATCGAAGAGCAATCTTCACAAATAGAAGAGCAGCTGTCTAAAATCAGGCAACAGAACTCCCAAATCGAGGAACACACATCTCAGATTCAGGTCCAGACTTCCCAGATTCAGAACCAGAATGCCCAGATTCAGGAGCAGTTGACCAAGATTCAGAGCCAGGAATCTAAGCTTGTTGACCAGCAGTCTGAAATTCAAGAAAAATCTGTTCAGCTACAGCTACAGTCCGCTCAAATTCAAGAACAGACTGCCCAAATTCAAGAACAATCGGTCCAAATCCAGGAACAGCAATCCAAGATTGAGGTTCATTTGTCGCAAATCCAGCAACACGTCACAGCAATTGAGCAACATGAATCTCAAATCAAACAGCAGGAGAGCCAGATCAATGAACAGCTTAATAGCCTCGATAAGCAGCAGTCAGAAATGGAGAGTCTGCAATCGCAGTATGAAGCGAAGGTGGTTGAATTCGAGCAGAAACAGAAGCAGTTCGAAGCTCAAATTGCGGAACAGAAGAAAGAAAACGAGCAATTGCACGCTGAAGTAGATGAGCAGATGTATCAGATTAATCATAAAGTCCTTCAAATTCAGCAGCTTCAAGAGAGCAATGGAGAAAAGGAAGCTAAGATCGCTCAGCAAGAAGTCGAATTGAGGAGCAGGGGTGTTGAAATTGAGAATGCCCAATCCACCATTCAGCAATTGCAATCCGAGCATGAAGCAAAGGTCGTTGAACTCCAATCGGAGCATGAGGCAAAGGTCATTCACCTCCAATCAGAACATGAGTCATTCGTGAGTCGCATCGAAGCCGAAAAGAGTGAGTTGATGACTCAGATTGAATCGAAAGCTCTTGAGATTAACGAGAAGCTGAGCGACATTGAACTACTGCAGAAGGAGTGTGAATTTAAAATGAATGAGATTGTGAAATGCAATGAAGAAATCGAAGGATGTACTTCAGAGATCGAAATGCTGCAATGGGAGAGGAACGAGATGGCGTCGGAAATGGAATCTCAGGATGCCAAACTGCAAGAATTGCAAGAACAGAAGGTCAAGAGCGAGGAAATGGCACGAGATAAATTACTAACTCTTACAAGAGAATTCGAAGGTCAGATCTCTGAGAAACAAGGTCAACTGGAGAAGCAAATCTCCGTGACTGAGCAACTGAAGTCCGAGTGTGAAACGCAAAAATCTCAACTGGATGACAAGATCGCACACATCACAAAACAAACAGCACAATTAGATGAGCAGACATCCATGTTACACAACCAACAAAACACCATACAAAACCTAAACGTCCAGGTTGAGAGGTTAGTCGAGCAGCACAAGGCGGAGGTCGCGACTCAGCAGAAGGCCTTAGAAGAGACGAGAGCCATTTTGGAGGTGAAGATGGTGGAGAGTAAGAAAGAGACGCAGGACGTCATGGAGCAAAAGATAACAACTATCCAAGAACGCAACATCTTGCTAAAGAAGGAGCTGGACCAGTGCGAACAGCGCAAGAGCGAACTCCAGCAGAAGTTGTGTGTGTTTGAAGAGAGAGCCACGAGCCTAAGAGACAAATGCAACGACATTGAGATTCAGGCCCAGAAGAGAGAGATGGAGTTGGAAGATCAGTTGTCGGCAAAGGTCAATGACCTTGAGAAGGAAGAGAAGAAGGTCAAGAAGCTGGAAGGAGATCTCTCTTCGCAGCAAAAGAAGCTGGAAGAGACTGAGAAGAAACTCCAATCTTTACAATCGCAACTGCAAACCTTGGAGAAGGAGAACGACCGCAAGATGGAGATGCTGGCCGAACAAGAAGAGGCCCACGTCACGGAAAGGTCTCAGTTGGTCTCCATGGAGCAAGAGCTTCAGAAGCAGCTGGAGATGAACCAAGCTCAGTTTGCGGAGGCCCAGAGTCATCTGCAGCAACGCCAAGAGCAGTTGATGGCCGTCAAGCAGGAGTATGTTCAATTGCAGAACCAGCTGCAAGAAACATCCAACCAATTGCACTTGACAAACGAGAGGAGCTCTTGCTTTGAGAAGGAAGTACAGGACCTACAAGTCATCATTCAGCAGACGAAAGAAAGCTACAACAATGAAATTGTTCAGTTGAAAGAGCAGATGCATTCCGCTGAAACAAATCATGCGAATATTGTCGAATCTCTGAAGAAAGATTACGAAGAGAACTTAGAGAAACTTCTCAAGGAGAAAGAAGAACAGTTGAAAGCCTTGCAGAGCGAGAAACAAGACTTAAAATGCTGGGAGATATTATCGCATAAGAATATGCAAGAATTGATGGTCGAAAATGAAGATATAATGAAGAAGTCTAATGAGCGAAAAGACATTATTGATAAGTTGAAGGTTGAAAATGATGAACTGACTCAGAAGCAGGTTGAGATCTCAAAAGAAAAGGAAGAAATTATCAAAGAAAAAGAAGAAATCTTGAAAGAAAAGGAAGAACTACAGATCGCCAAAGAAGAATTGGTCGTCAAGATTAAGGAAATCGAAGAACAGAAGGCGTTGATTGAAAAATCGCTGGAACAGAAGATCTCCGATATTCAACTTGAAAAAGAAGAATTGAAGAAGTCTCTGGAAGAAAAGATTGAGGACATCCAGACTGAAAAAGAAGAAATCAAGAACTCCCTGACAGCCGTTGTCACGGAGATTGAGAATGAAAGAGAAGAGTGGAAGAAGAATGTTCTTAAAGAACAAGAAGAAATGAAGAATAATCTCCTAAAAGAAAAGGAAGAATTCATAGTTAATTCCGAAAAAGCTTTGGAAGTTGAGAAAGGATTGAAAGATTCTCTTGAAAAGGAAAAAGAAGAATTGAGTTCCATGTTGTCACGGTTACAAGATGAGAAGGCCTCAGAGAATGGACAGTTGGCGACTGAATTACAACAAGCCATCAACGACAAGAATGCCATCCAGACGAGGCTGCAGGACTTGCAGCAGAAACTGCAGGAGGAAAACGACGCCATCAAAGAAAAGGTTGAGAAACACCAAGAAGCGAAGAAGACATGGAAACAAGAATTGAAGGAGGCAAGAGATGGAAAGGAGGAGGTGGAAGCTGAGTTGTTGGAGTTGAGAGCTGACTTTGATCGCCTCAAGGAGGAAGTGGAAGAAGCGAGGGACGGCAGAGACAGGTCGCTCAGGAGGATTTCTGAGTTGGAGGAAGATGTCAAGGGGTTGAAGAAACAACTGAGGGATTCCGAGGATACCAATGCTCAGTTGAAGACTCAACTGGCGGAATCTCAGGCTAAGATTGCTGAACTAGACCAGCTCCTTCGCCATGAGAAACAATCGAGAGATGTAGCCAAGAGAGATTGGACGACGGAATATGAACTGCAGCAGAAGAGAGCGGATTCAGAAGTGGCCGAGCTGGAGAGGGAGATTGACGAGGTCAAGAATGAGTACGAACAGAAGATGAAGCATCAACAAGACATCAGCACGAGGCAGTTGGAGATGGAGAGAAGTCGCTGCAAAGAGATGCTGAATGAACTGAAACAACAGGTGGCCGAGATCAGTGAAGTCAAGGAGAAGTTGGAGGCCAAGACGAGGCAGGAGGCCTTGAAAGAACAAGAACTAGAAAGTATGAAACAGAAAGTGAAACGATTTGAAACTGCGAAAGAAAAGCTGCAACAAGAGTTAGACATGTACATGGAACGAGAGAAGAAGATCACGGGCAGCCATGAGCACGATCTCAAGTCCGCCTCGGAAATGAACCAGAGACTACGGACTAAACTGGAGCAGAAGGAGGTTGAGATGCAAAAGGTCAAGACCCAGTGGTCTACGGAGATTGATGAGCTTCAAAGTAAGCTGGACGGCATGCAAGATTCGTATGAAGTGAAATTGGAGCAGTCCGTTCAAACATACCACAAGAAGTTAGACCAGGAGATGAAAAAGTGGGAAGGAAAGATGACCGCCCTCAAAACACAGAGTGAAGCCAACCTTGACACCTTAGAAAGAGAAGTTGCCCACTGCAAAGAACAGCGAGACCAGTGTTTGCAAAAGTTAGAAACAGAGCAGAGATCCAACCAAGAACTCCAGAAGAGGCTAGAGAATTTGGAAAGCAAATGTCAGAACTCAAAATCACAATCGTCAGAGTTAGAAGAGCAGGTGGAATACCTTCAGAGCACGCTCAATAGGCACAAGGAAACTGCCCAAACGTATAGAGATATGAGTGAATCGCAGAGCCTCCGAGCGAACCAGTTGGCAGACAGGTTGAACCTCGAGGAGCAGAGGACGTCTACCCTTGAAGAGAAGTTGTCTCGGACGTGCGATGAACTTTCCACGGTCAGAAATCAGTTGCACGAGGAGACTGAGAAGAGGAAAAGACTAGGCAATCAAATTGCGAGTCTTGAAGCTCAGGTTGAACATGCGAACAGGCAATTAAAGGAAAACACCCATGACGGAGACAACCATGAGTTCAAGCGGCCTACGATGGCTTTCACTGTGGCCACCCCAAGAGATGTCACTCACACGGTCACCAAGATGAAGAAGGAAACCACTTTCGATGCCAGAAATGATCTCAATGATTCCATAGACCCTCAGCGTTCTGTCAGCTTGTACGACCTGCGGCCTCGTTCTGCGAAGAAGAAGAATTCCCCAGTAAGGCACTCTTACCACGACGACAGCAGACATGATGTCTCATTTGGAAATTTCCACGATCCTTTGAAAATGACGCCTGGAAAGTTGTTCGGAAACGCCAATGACGAACCTAACACTAGAAAGTCGACACGCTTAAGTGTGAGCAGTACAACCTCTGCTAGTTTCAATCCTAGAAAATCTCTCAACGTCCCTCGAGGAACCGGAGCCTTATTCTCATCGGAAGATGAACCCGACGTGTTTGAGGACTTCCAGTTTGGTCGTTTATCAGAGTTACAAAGGAGGAACACGCTCTGTTTGCCCCACATGAAATCCAGCTACCCAGTTGAAGTGCAGGTCCGGCCACCTAATGAAATCTCTGAGATTAAATTACAACAAGGTGCGGTCGACACCACGGAGGTGAAGCCGTCTGTCAAAGCAAGACCCATGAAGAGGACGTCCTCTGGAGATGTGAAGTTGGATGGGTCAATTCTCGAACCTCCCAGAACAAAAATGCAGTCTCCTAAAACAAGGATCCAGCCGTCAAGAAAGGTGCTACAAGAAAGGAAGGATTTGACGAACAGTCCGGCACGTACTGTATCTCACAAAGTCTACCCAAAGAAAACCCCCAGGGGAACAAAGACGACACCTGGTAGCGACAAAAAGAGACGTTTCCCTCGATTGACGCCCAGAAAATCAAACAAAGAGTCATCTTCCTCCAGTGATGACATGGGATCGAGCAGTGTGTCATTCAACATCGGTTTCACCCCCAAGAAGTCAAGACGCATGAGCAGGTTCATGGGGATGGGAAAGCGAAAGGACGAGGAGTCGAGCAAGGGAGCTATTAGCAAAAACTTGTTGGATCAAGTTCCTCCGTTCAAAAAAAAGCGTTCAGCCGCCGAGGATGTAGTCGAATTAGTGGCGTTTACGAATTCATTGTGAGAAGAAGAAATCCTAAATACTTGTGGGGCCAATATTGACTGAGAGGAGAAGCTGATAATGAAAAATCTAAAAGACATTCGCTATTATTTGTGTGCTTGCGAGCAGCTGAATTTTGTAAATGCATTTGTTGAAGATATCTCAATCCTTAGAATTTGTCCGAAATTTTATTTATCGAAACTCTATTTGTCAATTTTATCATACACGCACAGTGTGTACATACTGTACTATGTTATGCATATCATTATTTTATCATTTTAAACATCGGCAGCATTTTTTATGTACTTCATCAGATTTATGTGATGAATCTTAATTTTTTTATGACAGACATAATAACTTGAAACTACAGACTTTGAAGGACTGATTTACAAATGAATTCATTATTGTCTCTTTACAAGTTTATTTATGTCCCATCATGTTGTGCCGTGCTTTTACTGGGCTTTAATTAAATTGTATAATATTTATACATATTAATACATAATGGAACACATGTCAGTTTGAAGGATTGGAATTATTTATTTTAGATATTACCACATAAAATTCTACCTTTTAAACAATTTTAACCATTGATTTTTAATTTGGCATAATATAATGTATCTTCTCTCTACATAAATATTATTTTCAATGTAGTCTGGAAAAATACGCTAAAAGAAATGTTAGATTTAATGTAATTGTTAAAGTGTGCCATAAATTTTAGCATTTTAATTTAATTAGAGGTAATTTAATATCTGTCTAATCACATTTAAAGTTGAATAACATTTAGGGTTTAGTGTGTCTTGTATTGTGATATAGGAAGATATATTATTAGACCGATTTGTGAAGCATATGAAAAAATCCTTGTAAGATTAACTTTGATATTATTTGTCGCGTCAACCTCAAGGTTCACATATTTTTATTTTTGAGACTGTTTGGTTATTTTGTTTTAGGTATTTGTAAATCTTGAACTCAGATTTGCTTTTGTTTATTGATGTACATAGAGTAAATAAAATGTAAATATCTGTACAGATGTTAAAAAAAAA

Protein: 1690 aa

>NuMA_protein

MESLQSQYEAKVVEFEQKQKQFEAQIAEQKKENEQLHAEVDEQMYQINHKVLQIQQLQESNGEKEAKIAQQEVELRSRGVEIENAQSTIQQLQSEHEAKVVELQSEHEAKVIHLQSEHESFVSRIEAEKSELMTQIESKALEINEKLSDIELLQKECEFKMNEIVKCNEEIEGCTSEIEMLQWERNEMASEMESQDAKLQELQEQKVKSEEMARDKLLTLTREFEGQISEKQGQLEKQISVTEQLKSECETQKSQLDDKIAHITKQTAQLDEQTSMLHNQQNTIQNLNVQVERLVEQHKAEVATQQKALEETRAILEVKMVESKKETQDVMEQKITTIQERNILLKKELDQCEQRKSELQQKLCVFEERATSLRDKCNDIEIQAQKREMELEDQLSAKVNDLEKEEKKVKKLEGDLSSQQKKLEETEKKLQSLQSQLQTLEKENDRKMEMLAEQEEAHVTERSQLVSMEQELQKQLEMNQAQFAEAQSHLQQRQEQLMAVKQEYVQLQNQLQETSNQLHLTNERSSCFEKEVQDLQVIIQQTKESYNNEIVQLKEQMHSAETNHANIVESLKKDYEENLEKLLKEKEEQLKALQSEKQDLKCWEILSHKNMQELMVENEDIMKKSNERKDIIDKLKVENDELTQKQVEISKEKEEIIKEKEEILKEKEELQIAKEELVVKIKEIEEQKALIEKSLEQKISDIQLEKEELKKSLEEKIEDIQTEKEEIKNSLTAVVTEIENEREEWKKNVLKEQEEMKNNLLKEKEEFIVNSEKALEVEKGLKDSLEKEKEELSSMLSRLQDEKASENGQLATELQQAINDKNAIQTRLQDLQQKLQEENDAIKEKVEKHQEAKKTWKQELKEARDGKEEVEAELLELRADFDRLKEEVEEARDGRDRSLRRISELEEDVKGLKKQLRDSEDTNAQLKTQLAESQAKIAELDQLLRHEKQSRDVAKRDWTTEYELQQKRADSEVAELEREIDEVKNEYEQKMKHQQDISTRQLEMERSRCKEMLNELKQQVAEISEVKEKLEAKTRQEALKEQELESMKQKVKRFETAKEKLQQELDMYMEREKKITGSHEHDLKSASEMNQRLRTKLEQKEVEMQKVKTQWSTEIDELQSKLDGMQDSYEVKLEQSVQTYHKKLDQEMKKWEGKMTALKTQSEANLDTLEREVAHCKEQRDQCLQKLETEQRSNQELQKRLENLESKCQNSKSQSSELEEQVEYLQSTLNRHKETAQTYRDMSESQSLRANQLADRLNLEEQRTSTLEEKLSRTCDELSTVRNQLHEETEKRKRLGNQIASLEAQVEHANRQLKENTHDGDNHEFKRPTMAFTVATPRDVTHTVTKMKKETTFDARNDLNDSIDPQRSVSLYDLRPRSAKKKNSPVRHSYHDDSRHDVSFGNFHDPLKMTPGKLFGNANDEPNTRKSTRLSVSSTTSASFNPRKSLNVPRGTGALFSSEDEPDVFEDFQFGRLSELQRRNTLCLPHMKSSYPVEVQVRPPNEISEIKLQQGAVDTTEVKPSVKARPMKRTSSGDVKLDGSILEPPRTKMQSPKTRIQPSRKVLQERKDLTNSPARTVSHKVYPKKTPRGTKTTPGSDKKRRFPRLTPRKSNKESSSSSDDMGSSSVSFNIGFTPKKSRRMSRFMGMGKRKDEESSKGAISKNLLDQVPPFKKKRSAAEDVVELVAFTNSL

Domains

Smc_ProK_B: 193 – 1054

Clone

Partial ORF

Primers

Forward: GAAGCAGCTGGAGATGAACC

Reverse: CCACTTCTGAATCCGCTCTC

Nucleotide# 1912 – 3413 = 1502 nucleotides

>NuMA_clone

GAAGCAGCTGGAGATGAACCAAGCTCAGTTTGCGGAGGCCCAGAGTCATCTGCAGCAACGCCAAGAGCAGTTGATGGCCGTCAAGCAGGAGTATGTTCAATTGCAGAACCAGCTGCAAGAAACATCCAACCAATTGCACTTGACAAACGAGAGGAGCTCTTGCTTTGAGAAGGAAGTACAGGACCTACAAGTCATCATTCAGCAGACGAAAGAAAGCTACAACAATGAAATTGTTCAGTTGAAAGAGCAGATGCATTCCGCTGAAACAAATCATGCGAATATTGTCGAATCTCTGAAGAAAGATTACGAAGAGAACTTAGAGAAACTTCTCAAGGAGAAAGAAGAACAGTTGAAAGCCTTGCAGAGCGAGAAACAAGACTTAAAATGCTGGGAGATATTATCGCATAAGAATATGCAAGAATTGATGGTCGAAAATGAAGATATAATGAAGAAGTCTAATGAGCGAAAAGACATTATTGATAAGTTGAAGGTTGAAAATGATGAACTGACTCAGAAGCAGGTTGAGATCTCAAAAGAAAAGGAAGAAATTATCAAAGAAAAAGAAGAAATCTTGAAAGAAAAGGAAGAACTACAGATCGCCAAAGAAGAATTGGTCGTCAAGATTAAGGAAATCGAAGAACAGAAGGCGTTGATTGAAAAATCGCTGGAACAGAAGATCTCCGATATTCAACTTGAAAAAGAAGAATTGAAGAAGTCTCTGGAAGAAAAGATTGAGGACATCCAGACTGAAAAAGAAGAAATCAAGAACTCCCTGACAGCCGTTGTCACGGAGATTGAGAATGAAAGAGAAGAGTGGAAGAAGAATGTTCTTAAAGAACAAGAAGAAATGAAGAATAATCTCCTAAAAGAAAAGGAAGAATTCATAGTTAATTCCGAAAAAGCTTTGGAAGTTGAGAAAGGATTGAAAGATTCTCTTGAAAAGGAAAAAGAAGAATTGAGTTCCATGTTGTCACGGTTACAAGATGAGAAGGCCTCAGAGAATGGACAGTTGGCGACTGAATTACAACAAGCCATCAACGACAAGAATGCCATCCAGACGAGGCTGCAGGACTTGCAGCAGAAACTGCAGGAGGAAAACGACGCCATCAAAGAAAAGGTTGAGAAACACCAAGAAGCGAAGAAGACATGGAAACAAGAATTGAAGGAGGCAAGAGATGGAAAGGAGGAGGTGGAAGCTGAGTTGTTGGAGTTGAGAGCTGACTTTGATCGCCTCAAGGAGGAAGTGGAAGAAGCGAGGGACGGCAGAGACAGGTCGCTCAGGAGGATTTCTGAGTTGGAGGAAGATGTCAAGGGGTTGAAGAAACAACTGAGGGATTCCGAGGATACCAATGCTCAGTTGAAGACTCAACTGGCGGAATCTCAGGCTAAGATTGCTGAACTAGACCAGCTCCTTCGCCATGAGAAACAATCGAGAGATGTAGCCAAGAGAGATTGGACGACGGAATATGAACTGCAGCAGAAGAGAGCGGATTCAGAAGTGG

Cloning info

PCR rxn on 5/11/15 using GoTaq polymerase mix

| Template | Initial denature | Denature | Anneal | Extension | #cycles | Final extension |
| --- | --- | --- | --- | --- | --- | --- |
| 2 hr cDNA | 4min @ 94C | 30sec @ 94C | 30sec @ 55C | 2min 15 sec @ 72C | 35 | 10min @ 72C |

Purified PCR Product with Thermo Scientific kit on 5/13/15, Second PCR from purified template on 12/1/15

Ligated PCR product into Pgem T Easy vector and transformed into competent cells on 12/1/15

Picked colonies for overnight cultures on 12/7/15

Isolated plasmids using Promega miniprep kit on 12/8/15

Confirmed clone NUMA by sequencing on 1/15/16

NuMA is sense to T7. RNA probe generated using SP6 on 1/20/16

**Insc**

Gene model: 3468 nucleotides

ORF: 499 – 2088 = 1590 nucleotides

>Insc_full

AGTGTTGCGACACCTCGGATGGCACATTGGTTGGCCACATTGGTTGGCCACATTGGTTAGCCCATTGTTGAATGTTGTTAGCCGGGGAAGCTGGAGTCACTCATTGAGTCATATTGTGATTCTTGAGTGAGGGAGAGGTGTGCGCATAGTTGTGTGAGACCGCTGAGGAGACCATCTGGGCATGCCTCTGCAGTCTGAGTGGCTCAGTACCAGGTCTAAGGTCTGTAATATTTAACCTGTGTCCACCTGGCTCACCGTCATTACTCAATTTTTATCTGGTGGTCACTTCATTACAGTCCTGAGTGCTCCTAATATCTTCTATATGCACACATAATATCTCCTAATGTTAAGGCAGCTTACAATCATTACAGACCTTTGTGAGTTTTCTTGCTCTTTTTTTTTTATAATCAGCGAAATGTTTACAAGACCTTTGGATTTGTTTTCCAGACTCGTTTCATCATTTTCCCCTGAGGAGAGGATGAAGGAGAAAACCTCATCATGACGGAGAGCAGCGCTTTGTACGAATGGCTGATCCATTCAGTCAACAAAATGCAGATGACGGAGGTGGACTCGGTGGCCCAGTGGTTGGAGGACCTCCGATTAACCACTGACACAGAATGCATGTGCGTCCTGCAGGGGAAGTCCCTTGCGAAGGACTCCACGGATGTTGTCTTCAATGCCTCTAAAACCAATAAAGACTATATCGACAGCATCAGGGGTCGAGCGCATGTCATCAGTGCAGAGTTTTCAAAATTATACAGAAAACTGGAAAAGGAGAGATGGAAACATGTGAAGAGCAGTGCCCTGAGACTGACTTGCCAAATCCGCTCCTTACTGCACGAGTGTAATGCGAGCATTCCCAATCCCAAGGCCGACTTTACCAAGTTGCAACAAAAAGTCATGGAAAGCAGCGCCAAGTTGGCTCAGCATGTTGAATTGCACCTGGACATGAATGGTCATCGCCCAATGCAAGAACCTGTTCTTCAACTATTAACCCATCTCGGTCAGGCATTTAGTACTTTGGTGGACAAGACTTTAAGCATTTTAGTAAAGAGGATAATCTTAAGCTTGAAGGATTGTGGGAATGTGTACACCATCAACACCATTTTAAACAGCCTTGTGACCCTCGGGTTGGAGGGAGAGCACATGTGCTACCTCATTTCGAAGGAGGGGGGCGTCGGGGTCCTCTTCAACATTTGTCGGAATCGCAGTCTGCAGCAAGCTCATGGGGCAGCATTGCGAGCTGTCGCTACTGTGTGTTGTGTGCCTGAAAGCATCGCTGAACTTGAAAAGGTTGGAGGCGTTGAATGCTTAAGTGACATATTGTCGGAGGAGAAACAAGCAGAGTGTGTCAGGTGTGAAGCTGCGGGAGTTATAGCTCAGATTACCTCACCATGTTTGGACTATTACCAACACATGACTGGCTTCATTGAACATATGGATGACCTTGTTAAGTCACTAACAGGTCTGTGTATGGATGCTACGAATCATGAAGTGTTCCTCCTTGGGGCAGCGGCTCTTGCTAACATGACCTTCATGGACAGCATGGCTTGCGAATTCCTCGGCTCTTATGGAACTGCTCGGGTTTTAATAGAGGCCTGCCACCTGGAAAAAGCCAGCACGCTATTCGCTAAAGATCAGGTAGCAACCATACTTGCCAACATGTCTGCAATAGAACAGTGCTGTATAGACATATCAGAACAACACGGGGTGGCACTACTGGTGCAGTTCTTGATGGAGGAGGTGCCCTCTGGGAACAATAAAGGAAACTACGAGGCAGAGGCAGCCGCGTGTGAAAGAGTTCAACAAAAAGCTGCCATTGCTTTAACTAGGCTAGCCAGAGATCCTGATAATGCTCAACATGTTGTTGAATTGCAAGGAATTCCGAGATTAGCCGAGCTCTGTCGAGATCCTGCATCAAGAAACCATAGTGATGCCGTCTTAGTGGCATGTTTGGCTGCATTACGGAAGATCTCAGCTGCATGTGGCCACAGTGACTTCGACAAACAAGACATTCAACAACTGATCACCCCAAAACTGACGGACTCATATCACATGTGCTCCAACATGGATGAAAGTTTTGTTTGAAAAGTTGAGAAGTTATTGGCCGATACTAAGTGACATTGGCAGGATTTCCAAGATCTGTTTGATACGACAGTTGTGTGCCGTCACATGGTGACACAAGATTCGGCTGGACAGCTTTTACACTTGTTATTGATTTTTACATGAGATATTTATTATATGATGATCTGTTGTTATCTATCTTAGGCACATGCCGATTGTGGGTTCATAAATTGATTTTCTGTTGAACCCGTTTCGAGATAACATTGCTTTTACGAAATATGTTTAATTTAAAGTCGTTGCCCTTAAAACTTCTGTGTCATGACACCGTCATCTCTAAGCGTTCCCTACATGTTCAGTGACATGTTTGTGTAACATTTTGACTTCTTTGATAAATCAACTCTGCAAAAATTCAATGCCCTGTCTGTAGTGCTGAAATAGTTAATTTTACGTTGTGCAATTAAGTTGCGTTATTGCGCATGTCGGAAGAGGCCACCCCAGGCCCACCAACCAGTAAGCATTTATTACTTTTTCAATACTGTTTTTTCTTGTGACCAACCAAGCTTGTCTTCTGCATGGAAAATCTGGAGGCTTGGCTTGTTAGTTTCCATTGAAACTTTGATATCTCATTTCAAACCAAAATAAAAAAATAAGTGGCATTTAATCTTGAAAGTTTGTGATAAGATTCATTATTGTAGCTAAAACCAGATAAATTAAATTACCGGTACATTACTTTTCTTTATTGTTTTGCTGATATGATTTCAACTGAAAAAGTCAAACTACATGTATACTATGATGAAATTATTATTGACCAATGTAACACTCTTTACTTGCCTAGAATAAGTAAAATAAAAGGTATATTACATCTGTACTATATTGTGTGACACTAAATATATAAACTCTTCTTTACAGTCTAATATGTGACATTGGCCTTAAAATTGCTGGGTCATTGTCCAATGCATACACTTGGTAAACATAGTTTCTAGTGAGTAAACACTTGAGGGAGCTTATGTGAATTTTTCTTAGGGACAAAATAGGCCAGGTATTTTTTATGATGTACTTGTCTCTTATATACACCTACTAGTGCGTACTGCTGATCCACCTTAAAATAGTTGGAGGCAGGGGCGGACTGGCGACATTTAGGTCCCCGTGGCAAAATTAGTCTGGGGGCCCCCAAGTTCCTACCACCGGCGTTATTTCAAAAGACAAAAAGGAAAATACTACAACATAATATAGTTTGGTAAAGAGTTATCAGGATGGGGCCCCATGTAAAAGGCACAAAATCATAAATCTGATGGTAAACTTTTGCTTTTCATTTTTTTTTAGGAATTTATATATTTTTTACATCATGAATGGCCCCCCTGAGTTGATGGGCCCGTGGCACTGCCACTCTGCCACATAGGCCAG

Protein: 529 aa

>Insc_protein

MTESSALYEWLIHSVNKMQMTEVDSVAQWLEDLRLTTDTECMCVLQGKSLAKDSTDVVFNASKTNKDYIDSIRGRAHVISAEFSKLYRKLEKERWKHVKSSALRLTCQIRSLLHECNASIPNPKADFTKLQQKVMESSAKLAQHVELHLDMNGHRPMQEPVLQLLTHLGQAFSTLVDKTLSILVKRIILSLKDCGNVYTINTILNSLVTLGLEGEHMCYLISKEGGVGVLFNICRNRSLQQAHGAALRAVATVCCVPESIAELEKVGGVECLSDILSEEKQAECVRCEAAGVIAQITSPCLDYYQHMTGFIEHMDDLVKSLTGLCMDATNHEVFLLGAAALANMTFMDSMACEFLGSYGTARVLIEACHLEKASTLFAKDQVATILANMSAIEQCCIDISEQHGVALLVQFLMEEVPSGNNKGNYEAEAAACERVQQKAAIALTRLARDPDNAQHVVELQGIPRLAELCRDPASRNHSDAVLVACLAALRKISAACGHSDFDKQDIQQLITPKLTDSYHMCSNMDESFV

Domains

INSC: 22 – 53

Arm: 379 - 472

Clone

Partial ORF

Primers

Forward: CCTGAGGAGAGGATGAAGGA

Reverse: GGAAATCCTGCCAATGTCAC

Nucleotide# 467 - 2137 = 1671 nucleotides

>Insc_clone

CCTGAGGAGAGGATGAAGGAGAAAACCTCATCATGACGGAGAGCAGCGCTTTGTACGAATGGCTGATCCATTCAGTCAACAAAATGCAGATGACGGAGGTGGACTCGGTGGCCCAGTGGTTGGAGGACCTCCGATTAACCACTGACACAGAATGCATGTGCGTCCTGCAGGGGAAGTCCCTTGCGAAGGACTCCACGGATGTTGTCTTCAATGCCTCTAAAACCAATAAAGACTATATCGACAGCATCAGGGGTCGAGCGCATGTCATCAGTGCAGAGTTTTCAAAATTATACAGAAAACTGGAAAAGGAGAGATGGAAACATGTGAAGAGCAGTGCCCTGAGACTGACTTGCCAAATCCGCTCCTTACTGCACGAGTGTAATGCGAGCATTCCCAATCCCAAGGCCGACTTTACCAAGTTGCAACAAAAAGTCATGGAAAGCAGCGCCAAGTTGGCTCAGCATGTTGAATTGCACCTGGACATGAATGGTCATCGCCCAATGCAAGAACCTGTTCTTCAACTATTAACCCATCTCGGTCAGGCATTTAGTACTTTGGTGGACAAGACTTTAAGCATTTTAGTAAAGAGGATAATCTTAAGCTTGAAGGATTGTGGGAATGTGTACACCATCAACACCATTTTAAACAGCCTTGTGACCCTCGGGTTGGAGGGAGAGCACATGTGCTACCTCATTTCGAAGGAGGGGGGCGTCGGGGTCCTCTTCAACATTTGTCGGAATCGCAGTCTGCAGCAAGCTCATGGGGCAGCATTGCGAGCTGTCGCTACTGTGTGTTGTGTGCCTGAAAGCATCGCTGAACTTGAAAAGGTTGGAGGCGTTGAATGCTTAAGTGACATATTGTCGGAGGAGAAACAAGCAGAGTGTGTCAGGTGTGAAGCTGCGGGAGTTATAGCTCAGATTACCTCACCATGTTTGGACTATTACCAACACATGACTGGCTTCATTGAACATATGGATGACCTTGTTAAGTCACTAACAGGTCTGTGTATGGATGCTACGAATCATGAAGTGTTCCTCCTTGGGGCAGCGGCTCTTGCTAACATGACCTTCATGGACAGCATGGCTTGCGAATTCCTCGGCTCTTATGGAACTGCTCGGGTTTTAATAGAGGCCTGCCACCTGGAAAAAGCCAGCACGCTATTCGCTAAAGATCAGGTAGCAACCATACTTGCCAACATGTCTGCAATAGAACAGTGCTGTATAGACATATCAGAACAACACGGGGTGGCACTACTGGTGCAGTTCTTGATGGAGGAGGTGCCCTCTGGGAACAATAAAGGAAACTACGAGGCAGAGGCAGCCGCGTGTGAAAGAGTTCAACAAAAAGCTGCCATTGCTTTAACTAGGCTAGCCAGAGATCCTGATAATGCTCAACATGTTGTTGAATTGCAAGGAATTCCGAGATTAGCCGAGCTCTGTCGAGATCCTGCATCAAGAAACCATAGTGATGCCGTCTTAGTGGCATGTTTGGCTGCATTACGGAAGATCTCAGCTGCATGTGGCCACAGTGACTTCGACAAACAAGACATTCAACAACTGATCACCCCAAAACTGACGGACTCATATCACATGTGCTCCAACATGGATGAAAGTTTTGTTTGAAAAGTTGAGAAGTTATTGGCCGATACTAAGTGACATTGGCAGGATTTCC

Cloning info

PCR rxn on 5/11/15 using GoTaq polymerase mix

| Template | Initial denature | Denature | Anneal | Extension | #cycles | Final extension |
| --- | --- | --- | --- | --- | --- | --- |
| 2 hr cDNA | 4min @ 94C | 30sec @ 94C | 30sec @ 55C | 2min 15 sec @ 72C | 35 | 10min @ 72C |

Purified PCR Product with Thermo Scientific kit on 5/13/15, Second PCR from purified template on 12/1/15

Ligated PCR product into Pgem T Easy vector and transformed into competent cells on 12/1/15

Picked colonies for overnight cultures on 12/7/15

Isolated plasmids using Promega miniprep kit on 12/8/15

Confirmed clone Insc by sequencing on 1/15/16

**Dlg**

Gene model: 5050 nucleotides

ORF: 41 - 2701= 2661 nucleotides

>Dlg_full

ACCTTGGCCACAGTCGGCTGCTTGTTGACACTCTTGAGAAATGCCGGTTAAAAAGCAAGACGCCCACAGAGCCCTAGAGCTCCTGGAGGATTACCATTCGCGATTGACCAAGCCCCAGGACAGACCCTTGCGCAATGCCATCGAGAGGGTCATTCGCATCTTCAAGAGCCGCCTCTTCATGGCCCTTCTAGATATTCAAGAATTTTATGAAGTGACACTGCTGGATGATAGTAAAAGCACACACCAGAAAACTTTGGAAACCCTGCAGATAGCTTCGAAATGGGAGAGGCAGCCACCAATCACCAACAGCCAGCCTGCTATGTTGGAGAAATTCCGATATCCCTCGCAGCAGGATGAGGAGCCGCTGCCTCCCCCTCCTCCGGAGTTGATGGAACCAAAACAGATGATGCACGAAACATTGCACCAACAGTACAGCCACGACGCTCCACACTACGATCAGCCTCAGGCCACAGAACACTACGATAACACAGATTATATTAATGGAGAGGGAGAATGGGAATATGTAGAAGTCACGTTAGAACGGGGAGGGACAGGGCTAGGTTTCAGCATTGCAGGAGGTACAGACAACCCTCATATAGGAGACGATCCATCTATTTATATCACAAAAATCATCCCAGGGGGAGCTGCAGCAGCTGACGGAACACTTCGAATGAACGACATTATTCTCTCGGTTAACGACGTGGATTGCGTGAATGTCACCCATGCTCAGGCGGTCGAAGCTTTGAAACGGGCGGGAAACACTGTCAAATTGGTCGTCAAAAGATTAAAAGCCGCCACAGAAGAAATTGTGGAACTTGAACTGGTTAAAGGAAACAAAGGCCTGGGCTTCAGCATTGCAGGAGGCAAGGGCAACCAACACATTCCTGGTGATAACGGCATTTTTGTCACAAAGATCATCGATGGCGGAGCGGCACAGATAGACGGACGACTGGCCGTGGGCGACAGACTTTTGGCTGTAAATACTGCTAACTTGACGGACGTCACCCATGAAGATGCCGTTGCGGCCCTTAAAGCCACGCAAGAGCGCGTTCTATTGGTGGTTGCCAAGCCAACATACATTACAGGAGAGGGCGACATTGTTATGGACCCTACCACACCATCTGTTCACAGCGAATACACGGGAGATGGACACATTCACACGCCAGCAAGCGGTTACCAGACACCAAGCACGCCCCCTCACAAAATGGCCGAAGTCAACCACGACATAGCGAGAGAGCCTCGGAAGATCTTACTAAAGAAAGGGGGCAGTGGCCTTGGCTTCAACATCGTCGGAGGGGAGGATGGGGAGGGAATCTTCATTTCATTCATCCTGGCAGGGGGTGCTGCCGACCTCAGTGGGGAACTACACAAGGGTGACCAGCTTCTTTCGGTCAATGGTATAGACTTGCGGGATGCCACCCATGAACAGGCAGCGGCTGCTCTCAAAGGGGCCGGCGAAACGGTAGAGGTTATCGCCCAGTATAGACCTGAAGCCATGGAAAATGTGTTTGTAGAATACAATCGGTTTGAGGCGAAGATCTCTGACCTCCGAGAACAACTCATGGATAAGAGTACGGGAAGTTTGCGTACCACCCAGAAGAAGTCTATGTATGTTAGGGCACTATTTGACTACGACCCCACCAAGGACAGTGGTCTGCCCAGCAAAGGATTGGCGTTCAAGTACGGAGACATCCTGCATGTGACAAATGCTTCCGACGACGAGTGGTGGCAAGCCAGGAAACTTGTGCCGGAGGGAGAGGAAGATAACTTAGGGATCATTCCCTCCAAGCGCAGAGTGGAGCGAAAGGAGAGGGCTCGTCTGAAGAATGTGAAATTCCAAGGGAAGGGACAAGAACGCAGCAATTCCACAGATCAACTCATGGGTACGAGTGACAGGAAAAAGAAGAACTTCTCTTTCAGTCGAAAGTTCCCTTTTATGAAGAGCAAAGACCAAAGTGGAAGCGAAGACGCCATCAGTGCTGACGAACAGAATGACTCCCCTACAAAAGGATCCTTGACTGCAATTAATGAAAATGTAACTCTGCCTCAACCCCAGCCTATTCCAGCAGGCGAGGAACCCGTATTGTCGTATGAGGCCGTAGTACAACAAGAGTTGAAATACACACGACCAGTGATCATCCTGGGACCATTGAAGGATCGCATTAACGATGACCTCATCTCAGAATTCCCTGACAAGTTCGGCAGTTGTGTACCACATACAACGCGCCCGAAACGGGACTACGAAGTGGACTCGAGGGACTACCACTTCGTGACGTCCCGAGAACAGATGGAGAAGGACATCCAGAACCATCTGTTCATAGAAGCCGGACAGTACAATGACCATTTGTACGGCACTTCGGTGCAAAGTGTGAGGGATGTAGCGGAGAAGGGCAAACATTGCATACTTGACGTCAGTGGGAATGCTATTAAACGACTGCAGGTGGCGCAACTCTACCCCATCGCCATTTTCATCAAGCCCAAATCAGTGGAAAGTATAATGGAATGGAACAAGAGAATGACGGAAGAACAGACGAGAAAAGCGTACGACAGAGCCTTGAAGTTGGAGCAGGAGTTTGGAGAATATTTCACTGCTGTTGTTACCGGAGATACTCCTGAGGAGATTTATGCCAAAGTGAAGGAGGTGATCCGCGACCAATCTGGACCTACTATATGGGTCCCGGCTAAGGACAAACTCCAGTGAAACGCTTCTGAAAAAATGTATTCCTGACTGAATAGTCAAACTTAGCTGTGAATGGAAACTAATACAAATGGAGATATCACTGTTGAAATACGGATGTGTGAGATATATTCAACGTTATCTGAACTCTGGGCTATCAAACATGTCTTTCCAGCGTTCAGATTACAACAACATAACTTTACTGACATCTTCCCGAATGATTTCTGTTACTCCCGGGCTCATCACATCACCTCGAGAACATTTAAGCAATTTTACCAATACATGATCGGATCAAGGAACACGGAAAACGTTTCTTTAAAGTTTATTAGTGATTCACGAATCATTTGAAGTGGTTCCTAAACGATTGGACACATCGGAAGGCTCTACTATGATCCTACGTGCTCGACAACTCTTATTATCCTCGGCTTATAGATTACTAATCGTTCCAATCCAATCGACTCGTTCTTTTCGCCCTCTTAGTGGAAAAACTGGTTTTGCACAAGGGGGCAGCACCTGTCAGTATGCCATCTAGTGTTCATTTCTGCAGAGCGTTGTTTTATAGGAAATTATGGACTATCTGATCTATTCGATTAGCAATCCGCTGGTTCTTGTTTTAATTAATGAGCGGATACACATATATCGTATTTTATCTGCTTGCTAGCTAACTATTGAGTGACTTGGATTTTTACTGCGCTGCTGTGCCGAAGTCACAGATTTATTTTAAATCATTTGTAAGAAGATTTGTTTCCAACTGTTTTGTCTTGTTATTTAATCAAATTATTTTGTTATGTCTGATTATGGTGGTCCCGAATTACTGCTTTTACCATCCGTACGATATTTGTCTTGTACGTGTAATCATTTATCTTGCAGTTGTTTTTAGATCCCACCATAATGTTTTACGATATACTGGATTTTAATCCTAAAATATTCGATTTTGATCCCGGAAGAAGACCAAAATTTGTTGTTTCCATACATCATTGTAGAACATTGGGATGAAATCATGAAAATGTTGGTTTTGCCATTTTGGGAAAATAGCCTGATGACGCCACTAAGGTTAAAATTAGATTACTTAAAGTGCTGCTTACGTTGCACAAACACTTAATTTTAAATCTACCCCAAGTCGCCTGTAATGATTGGTAGTATTTATGTATCACTTGATAATGTGATTCGGTTACTGTAATTTCCTATTTATAGTTGTGACCTATTTTTAAAATGATCGAGTTGTTTCGTCAGCGAGTCAATGAGTAAATAATCAGGAGTCATAAACTGAGACACTGACTGAAATAAAATTTGCCAAACCTGTTAAAGATTTCAAGACGGCTAAGATTAACGTAGTAAGATTAAATACTTTACAGGGGGGCCGCAGTATGCATCTAGTAAATATGACCATTTCAGTTTAAGTTACTCGAAAGTTCTTTGCTAATCGTAACAATTTCAGTATCAGTTTCATTGCTTTAATTCGATTGTGAGTATTCGAGCTGACTGCTTTTGTGTGTGAGATATGAATGATGAATAATTGGCAGTGGTGATGGGATAGGTAGTTCAATGATGATTAAAACAAATTTCAAAGTGGCTTCGCTTTTTTATGCCGAGTGGTTTCGAGCAGAAGTCTCGGAGCACCCGAAAAGCAAGCGACATGTGTCTCCTCTATGTATTATGATTTTACCAAGTGATTTTAATACTGTACTGCTGTAAAAATACAAGCTTTTATGCCTCGTCCGAGACTGAGCGTTTATTATATTCCTTTTTTGTGGCGTTCTTTTAAAATGCCGACGATTTTTAAGTTGCGTTGAAGTAGCATTTCCTAGATAGCAAGGTTGAAGATTGAACAATTTGATCAGTCGTATGATTGATTCAATTCATTCGATTGATCAATAATTGATAGTGATTGATTTGAAATATTCAATCCCAGTCAATCTGAAATCAATCGAAAAATGTCAGAAGTGTGACAAATGCATTAAATAATAATGACGCAATTTGATGTATTATGGAAATGTATAATTTACTTCAGTCATTGTCCATTGAGTTATTCTGTAGACTTGAAATAGTTTAAATAGTTTTGAAATTGTGAATTATAGAAAGGTGTGAACTGATGACCCCCTTTCCTGCATACAAGTAACCTGCCTTAATTTATTACTAGTTAATAACTAGTTACGTTACTAGTTGACATTATTTAATGAGTAAAATAGTAATATTATGTATAGTATTTTATTACGTAGAAGTGAATTGAATTGTTATTGAATCTTTTGAATTCAATCGGAGATGTGTCACAATTGGTGAAATGTTTCGGATTTAGTAAATTTTAGATTTTAAATCAATTTTAATAATTTTTTTAATGATGTTAAGTCAATTTTGACAATTTTCTTTTTAGGA

Protein: 886 aa

>Dlg_protein

MPVKKQDAHRALELLEDYHSRLTKPQDRPLRNAIERVIRIFKSRLFMALLDIQEFYEVTLLDDSKSTHQKTLETLQIASKWERQPPITNSQPAMLEKFRYPSQQDEEPLPPPPPELMEPKQMMHETLHQQYSHDAPHYDQPQATEHYDNTDYINGEGEWEYVEVTLERGGTGLGFSIAGGTDNPHIGDDPSIYITKIIPGGAAAADGTLRMNDIILSVNDVDCVNVTHAQAVEALKRAGNTVKLVVKRLKAATEEIVELELVKGNKGLGFSIAGGKGNQHIPGDNGIFVTKIIDGGAAQIDGRLAVGDRLLAVNTANLTDVTHEDAVAALKATQERVLLVVAKPTYITGEGDIVMDPTTPSVHSEYTGDGHIHTPASGYQTPSTPPHKMAEVNHDIAREPRKILLKKGGSGLGFNIVGGEDGEGIFISFILAGGAADLSGELHKGDQLLSVNGIDLRDATHEQAAAALKGAGETVEVIAQYRPEAMENVFVEYNRFEAKISDLREQLMDKSTGSLRTTQKKSMYVRALFDYDPTKDSGLPSKGLAFKYGDILHVTNASDDEWWQARKLVPEGEEDNLGIIPSKRRVERKERARLKNVKFQGKGQERSNSTDQLMGTSDRKKKNFSFSRKFPFMKSKDQSGSEDAISADEQNDSPTKGSLTAINENVTLPQPQPIPAGEEPVLSYEAVVQQELKYTRPVIILGPLKDRINDDLISEFPDKFGSCVPHTTRPKRDYEVDSRDYHFVTSREQMEKDIQNHLFIEAGQYNDHLYGTSVQSVRDVAEKGKHCILDVSGNAIKRLQVAQLYPIAIFIKPKSVESIMEWNKRMTEEQTRKAYDRALKLEQEFGEYFTAVVTGDTPEEIYAKVKEVIRDQSGPTIWVPAKDKLQ

Domains

L27_1: 4 - 62

PDZ: 163 - 247

PDZ: 256 - 342

PDZ: 403 - 478

SH3: 524 – 584

GuKc: 702 - 872

Clone

Partial ORF

Primers

Forward: TGCCGGTTAAAAAGCAAGAC

Reverse: GAGGCTCTCTCGCTATGTCG

Nucleotide# 42 – 1241 = 1200 nucleotides

>Dlg_clone

TGCCGGTTAAAAAGCAAGACGCCCACAGAGCCCTAGAGCTCCTGGAGGATTACCATTCGCGATTGACCAAGCCCCAGGACAGACCCTTGCGCAATGCCATCGAGAGGGTCATTCGCATCTTCAAGAGCCGCCTCTTCATGGCCCTTCTAGATATTCAAGAATTTTATGAAGTGACACTGCTGGATGATAGTAAAAGCACACACCAGAAAACTTTGGAAACCCTGCAGATAGCTTCGAAATGGGAGAGGCAGCCACCAATCACCAACAGCCAGCCTGCTATGTTGGAGAAATTCCGATATCCCTCGCAGCAGGATGAGGAGCCGCTGCCTCCCCCTCCTCCGGAGTTGATGGAACCAAAACAGATGATGCACGAAACATTGCACCAACAGTACAGCCACGACGCTCCACACTACGATCAGCCTCAGGCCACAGAACACTACGATAACACAGATTATATTAATGGAGAGGGAGAATGGGAATATGTAGAAGTCACGTTAGAACGGGGAGGGACAGGGCTAGGTTTCAGCATTGCAGGAGGTACAGACAACCCTCATATAGGAGACGATCCATCTATTTATATCACAAAAATCATCCCAGGGGGAGCTGCAGCAGCTGACGGAACACTTCGAATGAACGACATTATTCTCTCGGTTAACGACGTGGATTGCGTGAATGTCACCCATGCTCAGGCGGTCGAAGCTTTGAAACGGGCGGGAAACACTGTCAAATTGGTCGTCAAAAGATTAAAAGCCGCCACAGAAGAAATTGTGGAACTTGAACTGGTTAAAGGAAACAAAGGCCTGGGCTTCAGCATTGCAGGAGGCAAGGGCAACCAACACATTCCTGGTGATAACGGCATTTTTGTCACAAAGATCATCGATGGCGGAGCGGCACAGATAGACGGACGACTGGCCGTGGGCGACAGACTTTTGGCTGTAAATACTGCTAACTTGACGGACGTCACCCATGAAGATGCCGTTGCGGCCCTTAAAGCCACGCAAGAGCGCGTTCTATTGGTGGTTGCCAAGCCAACATACATTACAGGAGAGGGCGACATTGTTATGGACCCTACCACACCATCTGTTCACAGCGAATACACGGGAGATGGACACATTCACACGCCAGCAAGCGGTTACCAGACACCAAGCACGCCCCCTCACAAAATGGCCGAAGTCAACCACGACATAGCGAGAGAGCCTC

Cloning info

PCR rxn on 5/5/15 using GoTaq polymerase mix

| Template | Initial denature | Denature | Anneal | Extension | #cycles | Final extension |
| --- | --- | --- | --- | --- | --- | --- |
| 2 hr cDNA | 4min @ 94C | 30sec @ 94C | 30sec @ 55C | 2min 15 sec @ 72C | 35 | 10min @ 72C |

Purified PCR Product with Thermo Scientific kit on 5/6/15, Second PCR from purified template on 7/29/15

Ligated PCR product into Pgem T Easy vector and transformed into competent cells on 7/29/15

Picked colonies for overnight cultures on 8/7/15

Isolated plasmids using Promega miniprep kit on 8/10/15

Confirmed clone Dlg by sequencing on 8/13/15

Dlg is sense to T7. RNA probe generated using SP6 on 1/20/16

**Crumbs**

Gene model: 8591 nucleotides

ORF: 194 – 7780 = 7587 nucleotides

>Crumbs_full

CTCAGATGTGACGTCAGAGCTGAGGAGGGAAATCCTTAGCTTGGTCACGTGACGCGAAGCTTTGAATGGCTTCAGTGAGCGGCATGAGTTTGTGAAGGGAGGTGCAAAGCTCTGAGAAAACTCCAAATAACATGGATTTACGGTGAATTTAAAGTATGAATCTGTTGTGAATGGACCTGGATGGCTTACCAAGATGCACATGAAGACTACAATATATGCAGCCGGTGTATGGCTGCTCACACTACTGCTGCCCTCTTGTGGGCAAAACGGCAACATGGACAGACGAGACAGAGTGTCATTAGGGAATACATTAGCATATTTAGATTACTCCAAAAACACTTACTTACGTCTAAACACCACTATAGACCTTACCAGTCAAATTGCATTCAGTATAAACTTCCGGACTTGCTCTCACGGGCAGCTTTTATACCAACAAGGGCAGAGCGGAGACTGGTTCAGTGTAGCCCTAAATCACCAGGGGGCCTTAGTGTTCTCCTGGTGGCAAAACTCAACAACATCACAGAAAGACTCACCTTCAAAAGAGACATCAGTCGTCCTAGATAGCAAAAAGGACGAGAGTAGTAAAAACGCTGAAAATTCCGAAAAATCAGACAGTACTCGTGGATTTGCTCATAATAAGTGGTTTACCGCAGATGTACAGTTCCTTCAAGGCGTCATGACGCTCGCTGTTGAGCAGGGAAGTCAGACGCGATACCAAATACTCTTGTCAAACAGTACGTACCGCAGAGAATTGTGGGATCTGGATTTAAGTAACGGCGGGCCGCTGGTCGTCGGCCGGAACTTTACAGGATGTATCCAAGAAGGACTCAGTGTGATGCTCAGTGCTAATGATACGAGAAGCGAAAACATTCTATGGAATACCTGTCCGCTGGAGACCGAGAGGGGTTGTGAGGAGGACCAGCCTGACCCCTGCTGGAATTACCCGTGCCAAAATGGAGGAAGTTGTACGAGGAACGGAGCGAATTTCACATGCAACTGCCCTCTGCCTTATAGTGGACAGATGTGCGAGCTAGATTTGGGTGATAAAGGGTGCGACTTGGCCCCCTGTGAAAATGGAGGAACGTGTCTGCCGAAAACCACAGGAACCAGAAGATATGAATGCAGTTGCCCACCTGGGTTCACGGGCGTCAATTGTGAGATAGAGATCCATGAATGCGATTCCTTTCCGTGTCAAAATGGCGGAAAATGTTTGGACCAAGTCAACAGCTTCATCTGCAACTGTTCAGACACAGGCTACAAAGGCGACCTCTGTGAGACAAACATCAACGAGTGTATCGAGGCTCCCCCTTGTGAGCAGGAAGCTGTGTGCTCAGACACTCCTGGGAGCTTCCGTTGCCAGTGTCCTGTTGGCTACGCTGGGAAAACCTGTGAAGTGAAAATTGAAGAATGCAGCTCGGATCCTTGCCAGAACAACGGAACCTGCGAAGAACATCTGGGGTACTACGAATGCAAGTGTATGGAAGGTCTCACTGGCGTAAATTGTGAGGAGAACATCGATGATTGCACGCCCGATTCTTGCCCGGAGGAAAACTCGGAATGTGTCGATGGAGTCAATGAGTTCTCGTGCGTTTGCAAGGCTGGCTATACTGGTGAACCAGGTTCTTGCCTTGATATAGACGAGTGTGCGTCCGACCCGTGTCTGAACGGAGCAACATGTTTCAACGGAGAAGACTTCTTCAATTGCACATGTGTCCCAGGATTCATTGGTGTGACGTGCGAAACAGAACTGGATGAATGTGAGAGTGAACCGTGTCAAAACGGAGCAAGATGTCGTGACCTTGTAGATGCTTTTGCTTGCGATTGCCTCCCTGGTTTTTCCGGAGAGACATGCCAAGCAAATATCAACGAGTGTGCCTCGGCTCCGTGCAAGAACAATGCTACATGCAATGACATGATCGATGGCTTCAACTGCATCTGTGAGCCTGGCTGGGAAGGGCGCTTGTGTCAATTCAACATCGACGAGTGTGCCTCAAATCCATGTCAGAATGGCGCCAAATGTACGGACAGTGTCAACGGATACAACTGCACGTGCAATGCTGGATATGAAGGAGTGCACTGTGAGATTGATATAGATGAATGCGCGTCTTCTCCGTGTTTGAATGGAGCCACTTGCCAAGACCACGTTGATGGATTCGAATGTGAATGTACTGCGACATGGATGGGAGACACTTGTGACATCATCTACAACGCTTGCTCCTTCGACCCTTGCAAGAACAACGCCAGCTGTGTCACAGTGGCGCCTTCTAGGAACTACACTTGCAACTGCCTGAGTGGATTCGAAGGACAGGATTGCCAAGTCAACATTGACGACTGCGTGGGTCATTCCTGTCCAGAGTACCAAGTCTGTGTCGATGGCGTCAATGAATTCAAGTGTGCTTGTCCTATGGGCTACTCCGGTGACACCTGTGAGACAGAAGTGGACGAATGTCTGTCTTCTCCTTGCAAAAACGGAGGCACATGCGTTGATCAAGTTGGATCCTACAAATGCCTTTGTCCTGGAGCAACCGTCAATTTGACGAGGTACACAAATGATCCTGAGACTGTTTACAAAACTGGATTTTCTGGTGACAATTGTGAGGTTGAAATCAATGAGTGTGAATATGACCCCCCGATCTGCCTCAACGACGGCCTCTGCGAGAACACTAACACCAGCTTCTCCTGCTTGTGTAGAAATTCTGGTGGATTCTACAATGTCGGTGTATACTGTGAGAAGAGGAAGGAATACTGTGACATCGTTGAAGAAAATGACGAATGTCTGAACGGAGCGACTTGCAAGTCGTTCGTGGATGGGTTCGAGTGTGTTTGCGCGCCCGGATATAAAGGATCCAGATGTGAAATGGACATAGACGAATGTCTGTCTAACCCTTGTCAATACAACGGCAACTGCACGGACAAGGTCAATGGTTATTCTTGCGAATGTGTCCCGGGAATCAGCGGTGATAGTTGCGAGATTAACATCAACGAGTGTGAGTCAAACCCGTGCGAAAACGAAGGACAGTGCTTGGACAAAGTTAATGGCACCCTTGAACATTCTGTAGAAGAATCTGCAGTAGCGAGGTGGTTCGAATGCAACTGCACAGACACAGGCTTCACCGGCGACCTCTGTGAGATTAATATAGACGAATGCGAATCATCGCCATGCCAACATGACGGAAACTGTACAGACCTTGTTAAGGGATTCGAATGTGACTGCCACTTGGCATTTACAGGAGAATTCTGCGAGACTGACATTGCAGAATGCGATGCCCAACCATGCGAAAACGGAGGCACGTGTACGGAAAGATCAGCCGATAAGGAGCACCCTGATTACAAGCACGAAAACGCCCATGGCCACGATTGTGCGTGTTTGCCGGGGTTTACTGGAACCAACTGCGAAACGAACATAGACGAATGCGAATCAGACCCCTGCATCCATGGACTGTGCAAAGATGGCGTCAATGAATACGCGTGCGATTGTTCGCCAGGGTACGAGGGAGTCAACTGTGAGACGGAGATAAACGAGTGTGAAGTATATAAGCCGTGCAAGAACGGAGGAACGTGTGTGGACTTGGTCAACGACTACGTATGTGATTGCATTGAGATAGACACCCAACCTGTCAAGTATGGTGGAAAGAATTGCAGTATAGAGCTGACAGGGTGTGCTGTGAATGCCTGCCTCAATGGCGCCACCTGTGTGCCGTATCTAGTATCAGACATCTACGAAATTCATAATTACACGTGCAAATGTCCCGTGGGCTTCACTGACTTCTATTGCAAGACCTCGACGACCATGTCCCTTGACGGAACCGGTTTTGTCAGAATCCCGATGTCGGACGCTCAGCTCTACTTCAATCGGACGTTCATCGATTTCTCGTTCCGTACCACTATCCCGAGCGGGATTTTATTCGTCATCGAAGGTTCTCAACCGACAGAATCACTGACGGTTACCCTCGAAGGAGGAAACAAGTTGATACTAGTATATCTATCGCTGCTCGAACAGGGCCAAGCTCTCCTCAACAGTCCTCCTAATACGTCATTCAATGATTCTAACTGGCAGAACGTCGAAATTCACATGAACGATGAAGAGAGTATAGCGTTAGTCGTCAACCGTACGATGAACGCTCATCCAAAGAAGAGGATTCTGTCTCCGTTTGAATTCAAAACGTTCTTTGTCGGCGGTGATGGCAGTGGTACACAAGGTGGCAGCACAAACTTCATTGGATGCATCAGAGAACTTCATCTAGGTGGCAGCCTCATCAAGCCAAAAGAATATCTCGAAGACTCTACAGGAATGACCGTAGGCTGCAACAGGAAGGACCAATGTCACAACCCTGACCCCTGCCATGGCAACGGCTACTGCGAGGACCTCTGGACAGACTACAAGTGCTACTGCAGACGACCTTTTTTGCAGCCCAATTGCGCCACCAGTTACACTGCCGCAACATTTGGCAAGAATGGTGATTCCAGCATGGCAAAGGTCGCAATCCCTCCCGAAAAGGCCGAAGAACTCAAGACGGCCTTAAAAATCTCTATGTTCCTCCGAACGAGGGAAAAGGAAGGGTTGGTGTTCTACATCGGCAACGACGGACCTTCGCCCTTGACCTACATAGCTATGGCCGTGGCCAACGGATCTCTGGTCGTCCTTGTGAAGTTGCGCCCGGAGAATCAAGTGTTCCAGTTCAATTTCACGGATATTTCCGACGGCCAGCAGCGCCTTGTTCAACTCCATTGGGAGGGTACAGGTTTGGAAGTCTGGGTCGATGACATTCATCACAATTACACGGTCGATAATCAGTACGAGTTAGACGCAACGTACATCCAGTTTGGAATGATGAAAGACAGCCCCAAAGCAGGAGCGACAACGGATGCACCAGCCAAAAGAAAGAGACGCCAAGCTCCGACCGAAGAGAATGAACCGATAAAGCCGTGGGATGCTGGAGCGTTCAAGGGCACTATTCAGGACTTCCGCTTGGGAGATACTCCAATTCAGTTTTACCCCATCAATGGAACGACTGCGGATGACTCGATAATTGTGGGCAATATGACCAACGTACAGGAGGGCGAGGTGACCGATGAAGTTTGTACAAATCGGAATCCGTGTGTCAACAATTCAACTTGCAAGGACGTCTTCTTCAATGATTACGAATGTGTGTGTCCTGTGGGCTTTGTGGGCAAGAATTGTAGTGACATCGATTACTGCTATCAGACAACATGTCCTGGAGAAGAATCAAAATGTGTCAACAGAGATGATGGCTTCGAATGTGTCAGCACTGCAACATTTGACGGTTCCACCAGCTTCGTCTCTTATCAAAACGACCAGACGCATAATATGACATCTTTGAGAGCCACCTTCAGATGGCGCTCACGGAAGATTAATGGCACTATTCTCCACATGGCGGCAGCACCGCATTACATCACAATTGGCATGAACAGGGGCTACGTTGAAGTGCGCTACAATCTTGGAGACAAGGATATGAATATCAGTGTTCATCCTCTTCAGATTGACACCGCAGATGCAGCCTGGAACGAGGGAGAATTTGTCTTTGACAAACAATACATCAGTTACGTCGTGAAGAAACAAGGCTTCAAAACTCCAGTAGTTTCATACCGCCATTCCAAGCAGAACCATGAGTTGGTGTCTTTACTTCGTTCTAACAATAAAATCTTTGTCGGAGGTGTGGAGCAGGGATACTTCAAGCAGAATTCTGACATGTTCATGAACGATGACTTCTTTAAAGGGTGCTTGAGCGAGATACGCATTGGCGGCAGGCTCCTCCCATTCTTCTCATTGGACGCCCTCGGCGACAAGTCAACTCCGAAGCAGTTTCCTGTTACTACGGCCGTTGCTATAAGAGATGGTTGCCATGGCGACCCAGTGTGTGAAACCGATAAATGCGAAAACAACAGCACCTGCGAAGACATCTTTAATGCATACTTGTGTCACTGCGAAGACGGTTTTAACGGGACGTACTGTGAGAATAACATTGATGATTGCTACGAGGACGCTTGTTTGAATAACGGGACGTGTGTTGATGGAATTGAGGACTTCTTATGTGCTTGTCTGCCTGGATACACTGGAGAACAATGTCAAACTGAGATAGATGAATGTGCTTCGTCCCCTTGCATGAACAACGCAACTTGCTTCGATCTCTTGGACGGATTCTTCTGCAACTGTTCGGCCAATTTTACTGGTGATCTCTGCCAAGTCGCTTTAACCGATAACTGTGATACTCAACCCTGTGAAAACAACGGGACGTGCATACAAGTCAATGCCACGGAAAACTCCTTGGTTTCATTCAACTGCAGCTGTCAACCAGGGTACTCGGGTCTTCGCTGTGAGAACGAGATAGATTACTGCGAAGCCTCTCCCTGCCAGAATGGAGGCACATGTACTTCACATACGGACAAGCAGACTTACACTTGCAGCTGTTCTCTTGGCTACACAGGGAGTGATTGCGAATCTGAGATCAACGAATGTGACTCCTCCCCTTGTCAGAACGGAGCGGAGTGTCAGAATCTGATCGGAGACTACGAGTGCAACTGCACCACAGGTTGGTTGGGCAAGAATTGCGATGAGGACATCAATGAATGTAATTCGACCAACAGTCCGTGCCACAAAGGGGTCTGCATCAACAATGAGGGGTCGTACTTCTGCAACTGCGGAGACTCTGGCTATGCAGGACCTCACTGTGAGACCCAGATCAATGAATGCAACACAACTGAAAACTTATGTCAGAATAATTCAACATGTTACGACACGGATGGCTCCTACCAGTGTAATTGCACCCTTGGCTATGAAGGAGTGCATTGTGAGGTGGCCAATTGTACGCACAGTCAGTGTTTTAATGGCGGAGAGTGCTTGACCCGCAATGCAACCCTGACCGAGACTCCTTTTGACGTGACAGAATGGTACTGCGAATGTCCTCAGTTCTACGAAGGTGATCGCTGCGACACCAAAGGACCATGTGTTGATCTCCCCTGCCAGAACAACGCCACATGTTCACAAGACGGAGAGGAGTACACTTGCAATTGCACAAGTGGTTGGGAAGGAAAGAACTGTTCATCGGATATTGACGAATGTTCGTCCGACCCCTGTTTGAATGGAGCGACGTGCAATAATACAGATGGATCTTACGAATGTTTCTGTATCCCTGGATTTACTGGAGATAACTGCGAAACTAATGTTGACGAATGTCTGTCCGAGCCTTGTCAGAATTCCGGAACCTGCGTCGATGACATCAATTCGTTCCGTTGTGTGTGCGCTCCAGGTTACACGGGTACTCTGTGCGAGTCCGACATAGACGAATGCGACGAAGATCCCGATCTTTGCCAGAACAACGGAACTTGCATCAACGAGAACGGAGGATACAAGTGCGAGTGCTACGAGGGATACCTCGGAGATAATTGCGAGTTGAGGAATCCGTGTTATGAGTTTGAACCTTGCCAAAACGGAGGTCTGTGCTTCTTGAAAAATGGAACTGCAGAATTGGAGTTTGGGTGTGATTGTTCAGCTGGTTTCGAAGGGTCCACCTGCGAGAAAGCGGATCCAGCGAGAAAGCCGATTGTACAGGAGGATGATTGGGTCATAGAGGTCATTCTTGGCGTCGTCATCGGAATCAGTTGCCTCATAATCATTTTAGGGATCATCGCTTTTATCATCATGGCCAAAAACAAGCGCGCCACTAGAGGTACTTACAGTCCCAGCCGACAAGAAATCTCCGGTTCTCGGGTGGAAATGGGCAACGTTTTGAAGCCACCTCCCGAAGAGAGATTGATTTAAAGACGTTTTTAAAGGACAGAAGTATGAATGAAAACGTTTCAAAGGTGCCTCCGGAAGAGAGATTTAGTTAAAAACGTTTTAAAAACAGTACGATTGAAAACGTTTTTGAGTGATAAATGTATGAAAGGCATAAAAATAAGTCATCAGCATGCATGCTGCATGAATATATGAGTGTGAAAGTTTACAGTACCCCGTTATAACAACAGTTTAGAGATGGTCCGACTGAAGTATGAAAGCCGAAATGTATTATATTTGTGTATGGTTGTATGAAAGTTATTTCTGTATGATATGAGAGTTATTTTACCTTGTGTACAAAGTGTACAGAAATTAAGATTTTTAAAAGATAATTGAATATTGAGGTTATGTGGACCACTGTAATGTATCATACGAAGTATGATAATGTTAGCGATTTGAACATTTTCATTGTTATATTTTATGAGCTAAAAACCCATTTGTATTTCATAATTTTAGTAATTTTATATTATACTACAGACTGAAATTATGATTCTCGATTTTAGACGATTTACAACTTTTTAATCACTCTTAAGATATATATGATTAAAATATATAAATTAAAGTTTGTTATGATAAAATCATGGAGTCTATCTAAGTGCGATGATTCGATGGATTTATTTTATGGACTGCTTGATCGAAAGCATGTCATATTTTAATGTAAGATACTATTAAATTGTGATAAGGTGCAATTAAATGATTTTAAATTACCTCTGTCTGAATAACAGAGATTTGGATGAAGATTTTAGCTCATTATAACTGCCGCAAAACCATCTAAGCACTGCTGATGCATGGTGCG

Protein: 2528 aa

>Crumbs_protein

MHMKTTIYAAGVWLLTLLLPSCGQNGNMDRRDRVSLGNTLAYLDYSKNTYLRLNTTIDLTSQIAFSINFRTCSHGQLLYQQGQSGDWFSVALNHQGALVFSWWQNSTTSQKDSPSKETSVVLDSKKDESSKNAENSEKSDSTRGFAHNKWFTADVQFLQGVMTLAVEQGSQTRYQILLSNSTYRRELWDLDLSNGGPLVVGRNFTGCIQEGLSVMLSANDTRSENILWNTCPLETERGCEEDQPDPCWNYPCQNGGSCTRNGANFTCNCPLPYSGQMCELDLGDKGCDLAPCENGGTCLPKTTGTRRYECSCPPGFTGVNCEIEIHECDSFPCQNGGKCLDQVNSFICNCSDTGYKGDLCETNINECIEAPPCEQEAVCSDTPGSFRCQCPVGYAGKTCEVKIEECSSDPCQNNGTCEEHLGYYECKCMEGLTGVNCEENIDDCTPDSCPEENSECVDGVNEFSCVCKAGYTGEPGSCLDIDECASDPCLNGATCFNGEDFFNCTCVPGFIGVTCETELDECESEPCQNGARCRDLVDAFACDCLPGFSGETCQANINECASAPCKNNATCNDMIDGFNCICEPGWEGRLCQFNIDECASNPCQNGAKCTDSVNGYNCTCNAGYEGVHCEIDIDECASSPCLNGATCQDHVDGFECECTATWMGDTCDIIYNACSFDPCKNNASCVTVAPSRNYTCNCLSGFEGQDCQVNIDDCVGHSCPEYQVCVDGVNEFKCACPMGYSGDTCETEVDECLSSPCKNGGTCVDQVGSYKCLCPGATVNLTRYTNDPETVYKTGFSGDNCEVEINECEYDPPICLNDGLCENTNTSFSCLCRNSGGFYNVGVYCEKRKEYCDIVEENDECLNGATCKSFVDGFECVCAPGYKGSRCEMDIDECLSNPCQYNGNCTDKVNGYSCECVPGISGDSCEININECESNPCENEGQCLDKVNGTLEHSVEESAVARWFECNCTDTGFTGDLCEINIDECESSPCQHDGNCTDLVKGFECDCHLAFTGEFCETDIAECDAQPCENGGTCTERSADKEHPDYKHENAHGHDCACLPGFTGTNCETNIDECESDPCIHGLCKDGVNEYACDCSPGYEGVNCETEINECEVYKPCKNGGTCVDLVNDYVCDCIEIDTQPVKYGGKNCSIELTGCAVNACLNGATCVPYLVSDIYEIHNYTCKCPVGFTDFYCKTSTTMSLDGTGFVRIPMSDAQLYFNRTFIDFSFRTTIPSGILFVIEGSQPTESLTVTLEGGNKLILVYLSLLEQGQALLNSPPNTSFNDSNWQNVEIHMNDEESIALVVNRTMNAHPKKRILSPFEFKTFFVGGDGSGTQGGSTNFIGCIRELHLGGSLIKPKEYLEDSTGMTVGCNRKDQCHNPDPCHGNGYCEDLWTDYKCYCRRPFLQPNCATSYTAATFGKNGDSSMAKVAIPPEKAEELKTALKISMFLRTREKEGLVFYIGNDGPSPLTYIAMAVANGSLVVLVKLRPENQVFQFNFTDISDGQQRLVQLHWEGTGLEVWVDDIHHNYTVDNQYELDATYIQFGMMKDSPKAGATTDAPAKRKRRQAPTEENEPIKPWDAGAFKGTIQDFRLGDTPIQFYPINGTTADDSIIVGNMTNVQEGEVTDEVCTNRNPCVNNSTCKDVFFNDYECVCPVGFVGKNCSDIDYCYQTTCPGEESKCVNRDDGFECVSTATFDGSTSFVSYQNDQTHNMTSLRATFRWRSRKINGTILHMAAAPHYITIGMNRGYVEVRYNLGDKDMNISVHPLQIDTADAAWNEGEFVFDKQYISYVVKKQGFKTPVVSYRHSKQNHELVSLLRSNNKIFVGGVEQGYFKQNSDMFMNDDFFKGCLSEIRIGGRLLPFFSLDALGDKSTPKQFPVTTAVAIRDGCHGDPVCETDKCENNSTCEDIFNAYLCHCEDGFNGTYCENNIDDCYEDACLNNGTCVDGIEDFLCACLPGYTGEQCQTEIDECASSPCMNNATCFDLLDGFFCNCSANFTGDLCQVALTDNCDTQPCENNGTCIQVNATENSLVSFNCSCQPGYSGLRCENEIDYCEASPCQNGGTCTSHTDKQTYTCSCSLGYTGSDCESEINECDSSPCQNGAECQNLIGDYECNCTTGWLGKNCDEDINECNSTNSPCHKGVCINNEGSYFCNCGDSGYAGPHCETQINECNTTENLCQNNSTCYDTDGSYQCNCTLGYEGVHCEVANCTHSQCFNGGECLTRNATLTETPFDVTEWYCECPQFYEGDRCDTKGPCVDLPCQNNATCSQDGEEYTCNCTSGWEGKNCSSDIDECSSDPCLNGATCNNTDGSYECFCIPGFTGDNCETNVDECLSEPCQNSGTCVDDINSFRCVCAPGYTGTLCESDIDECDEDPDLCQNNGTCINENGGYKCECYEGYLGDNCELRNPCYEFEPCQNGGLCFLKNGTAELEFGCDCSAGFEGSTCEKADPARKPIVQEDDWVIEVILGVVIGISCLIIILGIIAFIIMAKNKRATRGTYSPSRQEISGSRVEMGNVLKPPPEERLI

Domains

LamG: 43 – 210

EGF_CA (16x): 291 – 322, 325 – 361, 363 – 400, 403 – 438, 480 – 516, 520 – 554, 556 – 592, 594 – 630, 632 – 667, 710 – 746, 749 – 802, 856 – 888, 890 – 926, 981 – 1017, 1070 – 1105, 1108 – 1149

LamG (2x): 1198 – 1350, 1429 – 1593

EGF: 1630 – 1663

LamG: 1693 – 1856

EGF (9x): 1930 – 1966, 1969 – 2004, 2009 – 2049, 2092 – 2126, 2129 – 2167, 2170 – 2207, 2292 – 2328, 2330 – 2366, 2368 - 2406

Clone

Partial ORF

Primers

Forward: GAATGGACCTGGATGGCTTA

Reverse: CTCTTCCGGAGGCACCTT

Nucleotide# 270 – 1622 = 1353 nucleotides

>Crumbs_clone

GCAACATGGACAGACGAGACAGAGTGTCATTAGGGAATACATTAGCATATTTAGATTACTCCAAAAACACTTACTTACGTCTAAACACCACTATAGACCTTACCAGTCAAATTGCATTCAGTATAAACTTCCGGACTTGCTCTCACGGGCAGCTTTTATACCAACAAGGGCAGAGCGGAGACTGGTTCAGTGTAGCCCTAAATCACCAGGGGGCCTTAGTGTTCTCCTGGTGGCAAAACTCAACAACATCACAGAAAGACTCACCTTCAAAAGAGACATCAGTCGTCCTAGATAGCAAAAAGGACGAGAGTAGTAAAAACGCTGAAAATTCCGAAAAATCAGACAGTACTCGTGGATTTGCTCATAATAAGTGGTTTACCGCAGATGTACAGTTCCTTCAAGGCGTCATGACGCTCGCTGTTGAGCAGGGAAGTCAGACGCGATACCAAATACTCTTGTCAAACAGTACGTACCGCAGAGAATTGTGGGATCTGGATTTAAGTAACGGCGGGCCGCTGGTCGTCGGCCGGAACTTTACAGGATGTATCCAAGAAGGACTCAGTGTGATGCTCAGTGCTAATGATACGAGAAGCGAAAACATTCTATGGAATACCTGTCCGCTGGAGACCGAGAGGGGTTGTGAGGAGGACCAGCCTGACCCCTGCTGGAATTACCCGTGCCAAAATGGAGGAAGTTGTACGAGGAACGGAGCGAATTTCACATGCAACTGCCCTCTGCCTTATAGTGGACAGATGTGCGAGCTAGATTTGGGTGATAAAGGGTGCGACTTGGCCCCCTGTGAAAATGGAGGAACGTGTCTGCCGAAAACCACAGGAACCAGAAGATATGAATGCAGTTGCCCACCTGGGTTCACGGGCGTCAATTGTGAGATAGAGATCCATGAATGCGATTCCTTTCCGTGTCAAAATGGCGGAAAATGTTTGGACCAAGTCAACAGCTTCATCTGCAACTGTTCAGACACAGGCTACAAAGGCGACCTCTGTGAGACAAACATCAACGAGTGTATCGAGGCTCCCCCTTGTGAGCAGGAAGCTGTGTGCTCAGACACTCCTGGGAGCTTCCGTTGCCAGTGTCCTGTTGGCTACGCTGGGAAAACCTGTGAAGTGAAAATTGAAGAATGCAGCTCGGATCCTTGCCAGAACAACGGAACCTGCGAAGAACATCTGGGGTACTACGAATGCAAGTGTATGGAAGGTCTCACTGGCGTAAATTGTGAGGAGAACATCGATGATTGCACGCCCGATTCTTGCCCGGAGGAAAACTCGGAATGTGTCGATGGAGTCAATGAGTTCTCGTGCGTTTGCAAGGCTGGCTATACTGGTGAACCAGGTT

Cloning info

PCR rxn on 5/5/15 using GoTaq polymerase mix

| Template | Initial denature | Denature | Anneal | Extension | #cycles | Final extension |
| --- | --- | --- | --- | --- | --- | --- |
| 2 hr cDNA | 4min @ 94C | 30sec @ 94C | 30sec @ 55C | 2min 15 sec @ 72C | 35 | 10min @ 72C |

Purified PCR Product with Thermo Scientific kit on 5/6/15, Second PCR from purified template on 12/1/15

Ligated PCR product into Pgem T Easy vector and transformed into competent cells on 12/2/15

Picked colonies for overnight cultures on 12/7/15

Isolated plasmids using Promega miniprep kit on 12/8/15

Confirmed clone Crumbs by sequencing on 1/15/16

**Pcd11x**

Gene model: 5795 nucleotides

ORF : 476 – 4201 = 3726 nucleotides

>Pcd11x_full

ATGCTCCTTAGCATGAAAACAGAACACCATTTGCAGTGATTACAGAGCCCTGGAAGACAGAAATGGCCCAACTTCGATGTTAAAACGATGTCGGAAACCGAGAAACATGACCCTGGAGTGAGCATTCTCCGTTGATCACACGGACAAGAGGCCAGTCAACAGCTGACTGTCTGACCAACAACATCGCACATCAGAGTTGATCACAGTTTTTGGAATATTACGATTGCCTATCAAGGATTTAAGGCTTAATTTAATAGCTGCACTTAAGGATTAGATGCTTGTGTGCTATTCTGGAGTCACTTTGGGTGGCCAGGTGAAAAAGGAGCTCCTAAAATTCTAAGTGCCAACTGAGCTCTGAATTGTGCATTACAATCTTGGAATTTTTACCACAAGGGCATCGCATTATCTAATGTCAGCTAGTGAGAGCAGAAGTGACAGTCGGCGCAGTCGGTGATGTAGCGACCCTTCATAGAAGATGGCTCTAGTGTTGGAATGGCCCGGCATCTTGCTACTGCTGCAGGTGATGGTGGTGGCACTGATGCGATCGGCCATGGCTCAAGACAGCCAGCTGGAGTTGACCTACTCTATCCTGGAGGAAGACATGGAGGGAACCTTCGTGGGCAACGTCCTACGTGACTCGCGTATCCAAGACCGCTACTCCAAATCCATTCTTACCCAACTTGAATTCCGCTTCATAGCGAAGCCGCGGGTGCCCTTCCTGATCAACAAATCCACCGGGGTGATCCGCACGGACGGACGGATAGACCGTGACGCTCTCTGTCCCCGCCAGGACACTTGCCGCGTCATCCTTGACGTCGTGGCCTTCACTCCGGGACAGACTCAGTTCCTCGAGATAATTAAGGTCAAAGTGAACATCGAAGATATCAACGACAACTGGCCTCATTTCCCTGATGCCCGCATCTCCCATCAGATACTGGAGTCAGCCGTGCCTGGGTATGGCTTTGTTATTCCCACCGCAATAGACCCAGACAGCGGTCAGTTCAGCGTGAGAGACTACGAGTTAGTTTCAAGTAGTGATAAGTTCGAATTGAGTGTTAAAACTAAGTTGGATGGGTCCACAGATGTGCGACTCATCTTAAAAGACTCCCTAGACAGAGAGGACGTGGATTTTTACCAAGTGAGAGTGGTTGCCTATGATGGAGGTAATCCGCCCAAGTCAGGTTCCGTGGATGTTAACATTATAGTACAGGATGCTAACGACAATGATCCCATGTTTACCAACGCTACCTACGAGGTTACAATAGCCGAGAATGCTGCTCCGGGGACCACTCTGACCAAGGTTCATGCGTCTGACAAGGACGCTGGCCTGTACGGACAGATCCGATACAGCTTCTCGAGCCGCACCCAGGATACGCATGGTGCCGTGTTCGGCATCAAGAACACAACCGGTGAAATTTACGTCAAGGGCGAGGTGGACTACGAAACGAGCCCTGTGTACCATCTTGTGGTCCTTGCGGATGACAGGGGCTCGGATACAATCCCTGCAGAGGCAACAGTCATTGTACGTGTCCTGGATGTCAATGACAATGCCCCTTTGATAACGGTGAATACCTTAACACAAGCGGGAACTGAGGTCGCAGAAGTGGTTGAGAATGCCGACCCCGAGACCTTTGTGGCCCATCTAACTGTCACCGACCCGGATAACGGGAAGAATGGCAAGTTTAATTGCTCGCTCAATGACGACTCCTTCAGACTACAGCAGTTCTTTGATAGTGAGTATAAGATAGTTACTAGGAAGAAACTCGACAGGGAGACTCACGATAAGTATAACCTATCTGTGTTGTGTCAAGACTTTGGCGACGAGCCTTTGGTAGCCATCAAGCGCTTACAGATTATTGTGACAGATGACAATGACCATGCGCCTGTTTTCAGCCAGTCGACCTACGAGGCCTCGTTAACGGAGAACAACCACATAGGGGTGTTCATCACCCGTGTCAATGCGACCGACAAGGACTCTGGCGAGAACGGCCTCATCGACTACAAACTGCATCCCGATGCTGGGGGCGACTTCCACGTGGACGGCTTGACTGGCACGGTGTCCGCCCGGGCTGTACTTGACCGAGAACAGACCCAGCAGATTCGTTTTAGAGTGATTGCCTCCGATAGGGGCCAGCCCCGGAAGTCGGGAACTGCCACTGTTATTGTCAACGTCCAGGATGTGAATGACGAGAAGCCCATGTTCGCTCAGTCCGCCTATTCTTTCGGAGTCTATGAGAATGAGCCGGAGGGCACGTCAGTGGGAACTGTACATGCTGTAGACCCAGACACAGACCCATACAACCAGTTTCAGTATTCCATATTACCCGGCCACAGTGCTGAGAATAGCTTCGCTATCGATCCCCACACTGGCAAAATCACAACGACGACAGTGCTGGATCGGGAGGACAAGCCTGTCTATTATTTACTGATCATGGCCAGCGACAAGGGTGTCCCGCCTATGAGTAGCACTGCCACTGTGTCCATTTATGTAGCAGACAAAAACGATAACCCCCCTGTTTTTGAATTCCCATCAGACACAAACAATACCATTAGCATATCAAATCAGGTGCCCAAGGGCTACTTGGTCACCAAAGTGAAAGCTATAGATAGAGACATTGGCGGTAATGCCAATTTGACTTATTATATTTCTCGTGGTAACCATTTAAACTTCTTCCACATGGATCCTATATTTGGTGTCATTACTGTTTCCACAGAACTCAAGGATATTGAGGACCATGTATTCCACATGCAGCTGAAAGTTAGTGATAGTGGTCAACCTGAAAAGTATGCCTTTAATAACTTGAACATAGTGGTGAATAAGTCCATACCCTTCCAACTGGCCTCTACCGGCCATATCCTCGGTGGTCATAACTTTACCATAGTGGTGTCGCTGGCCTGTGTGTCCGGGGTCATCATGGTGTGTCTGGTGATTGCGATTGTGGTGATTCGCCGCCAGGACACGGAGCGGCGGAACCACAAGTACAACTGCCGCATGGAGGCCCTGAAAATGCTCCACGCGACTCGGGAGAACCACAAGGAGCCCGAGATGGAGAGTACCTCGAGCAAGGCCCTGTCGAACGGCAGTTGCTCATCAGACTGTGATAAACCTGCCAAAAAGGAGGTCAGCTTTAATCTTGACATGGACGATCGTGATTGTGTGGAGTACGGTCATGTAAACGACCGATTGCAGAGGTCCTGGCCTTCGACCATCGACCACAAGACATTGGAGACACCACAAGCCTGCTCCTCCATGCTAACGACAGCTTCCAGTAACGGGTCCGACATAAGACCGAGTCGAAACAACAACAACGCAAGTCGCCAACCGCCGCCACCAAACAACGCTGCTGCTGCGGGTCGACCGCCATGGCCGTTAACACAACGAGAACTTGAGGTCCACCAACTGCTACAGATGCTGAAGGATCAGGACGCGGACAGCGCCTACTCCGGGGATGGCTCCAACACCGACAGCGGCCGTGGGCCCAGCGAGGAGGGTGAACCCGACAAGTGCGGAGGGGGAAGACGATTGGCGCACAGTCCCCAAGACGAGGGGCTCCAGGTCGTTCCTCAACTGTCAGCCAATAACAGCTTAAACGTGAGCCAGGTCACCAACAACCAGAGCATCCACAGCTACAGTCAGCAACCGCTGTGTCTGCAGACGTTCGCTCCAGCTCCGCGACCCGGAGAAGCCGCGACGCTAGAGAGACGAGGGCAGCTCAACACGGGATTCATCGGAACGACTCCAGGGGGTACAGACATCTCGCCCATCAAGCCCAGCACGTTCGCCAAACAATACTCTCCTGATGGCAAGTCACCCTATCAGCAGGGCCCTCACGGTCACCCTCAAGAAAATCCCTACGGCACAATCCAGAAGAAGAAGGCTCCGCAACAGCAACCCCAGCAGCCTCCGGCATATGGTGGCCACAACAACGCGGTGTCGCCTTACAGCAACGGACATTATGGCGGTCGCGGATATCACAGCGACGCGGAAACGGAAGAGACTCTAGCCTCGGACGACGACGGCGCTATGCAGTATTCGTGTGCCGACATGTCGGACGGCACCTACATACCAACCCCTCGGTCACTGCAAGTAGCAGACAATACGGCTGATGACGCTACCACCACTTCCGGGAGCTACGTGGTCGATCCCCAGGACCTGTGTCAGGAAATTGATGATCTCTTCTTCCGTGACATGGTTGTCTGATTCCTGATTGGCCAGCTCACTGAGCACTGTGTCACGTGCTAAAAGATGGAGGAAATGCAGTCATGTGGTTTAACCAGCTGTGTTGTGATTGGACAGTGTATGATGATATTCTAAACGACGTGCCTCAATCATTCGCCGATTGTTCATATATATATATTTCATTCTCAGGGAGAAATCATTCATAACGATTCATATAAGACTGTTTGCCACCGTTCAATCGATGTCCTATATGGCTTTCGATTGATCTCATCATGTTCATACTCATTCGTTCCCACCAAAACTGACTCCATGCACAATTCACTCTGTATTTCACTGGTTCTGTAAATGTATTAACCAAATGTGTCCCAAATGACTTGTTGTTGAGATTTGTCACACCTGGCATTGTTCATGTGTTCAAATTGTGTTCATTAATTTAATAGCCTTTTTGTTAAGTCCTGAAAGGATTTTAGATGATATTATTATGTGCAATGTTACGTTTTTGTTGTGAACATTTTTTGCTGCTGATTTTTATATACAAACCAAAGTCGATGTAGGCAGTATTGGAAGAAAGATGCACTGAAATCTATGCTATATATTTAGTGGTGGGAAGTTGGCAATAAAAACAAGACATTTGGAGATTCTCTCTATGCTTCTTGGGTTTCTGAAGGGCTGAGACCATCGAATCACTGACTCCCAAGTCATAAAAGAGGAAAGATTTCACGAATCCCGGCTCAAATGCCAGCTACCATTTCTTCTTTCCCTTTATCCTTAGAAATCCTCAGAAACGTATCAAAAGGCAGATTAACCTGACTTACAGCCATAAAACCCAGAAAGAGCAGTCTGCTGCAGCTCTTCTTCGCCTACCCAAGCTTCTGTGGTTTGCTAGCTATACCAGCATGCCTTTATAAGGGGGTTTGGAAGTATAAAAGAAAAGGCCCAATATGTATAGGTGGTTTTTAAGTTTCTTTTGGTGCCAGGGTTTTCTGGCCGGACTAATCCCACCGACATACCCTAGCACTAATTAAACTGCCCCGTCCGCCATATATAATGTAACCGAGACATGCGAAGGGTTTGCTAAGTAACATCATCAGGACCTTTAACCATCCCAGGACTGAGATGGCTGTCATTTGAGTGCCAGGACTCATCCTACCAGTTCTCTTGTGTGTACGAAGGGGGAGGGAGCGTTGGTCTAGTGGTAGCGTGTTAGGGTGTTAGGCTGCCAATCGCTGGGTCCAAGGTTCGAATCCAGCTCTTGCTGGTCACCTGGGCCTATGTTGGATGATCATTTAAATCATCATTCGCCTGATTGCCCTGACCACCTCCTGTCCTAATTAGCATACCTGGAGCATAACTACAGGACTTAACACCCCACATCATCCATCCTAGACCTGAGATGGCTATCAATTTCAATATCAGAACTCATCCCACCAGTTCTATTGTGTGTATGACTCCATTGTCTTCTTTTTAATGCCAACTGCCCACAATACAATTCTATTCCTATAACATAACTGCCCACTCTGAATGTAAACTGCCATTTTTATCTGCCCAAACTGCTATTCAGCTGAGTCTCGATGTCTACAAAATGCTGTAGACTTGTATTCATGTATATATTATC

Protein: 1241 aa

>Pcd11x_protein

MALVLEWPGILLLLQVMVVALMRSAMAQDSQLELTYSILEEDMEGTFVGNVLRDSRIQDRYSKSILTQLEFRFIAKPRVPFLINKSTGVIRTDGRIDRDALCPRQDTCRVILDVVAFTPGQTQFLEIIKVKVNIEDINDNWPHFPDARISHQILESAVPGYGFVIPTAIDPDSGQFSVRDYELVSSSDKFELSVKTKLDGSTDVRLILKDSLDREDVDFYQVRVVAYDGGNPPKSGSVDVNIIVQDANDNDPMFTNATYEVTIAENAAPGTTLTKVHASDKDAGLYGQIRYSFSSRTQDTHGAVFGIKNTTGEIYVKGEVDYETSPVYHLVVLADDRGSDTIPAEATVIVRVLDVNDNAPLITVNTLTQAGTEVAEVVENADPETFVAHLTVTDPDNGKNGKFNCSLNDDSFRLQQFFDSEYKIVTRKKLDRETHDKYNLSVLCQDFGDEPLVAIKRLQIIVTDDNDHAPVFSQSTYEASLTENNHIGVFITRVNATDKDSGENGLIDYKLHPDAGGDFHVDGLTGTVSARAVLDREQTQQIRFRVIASDRGQPRKSGTATVIVNVQDVNDEKPMFAQSAYSFGVYENEPEGTSVGTVHAVDPDTDPYNQFQYSILPGHSAENSFAIDPHTGKITTTTVLDREDKPVYYLLIMASDKGVPPMSSTATVSIYVADKNDNPPVFEFPSDTNNTISISNQVPKGYLVTKVKAIDRDIGGNANLTYYISRGNHLNFFHMDPIFGVITVSTELKDIEDHVFHMQLKVSDSGQPEKYAFNNLNIVVNKSIPFQLASTGHILGGHNFTIVVSLACVSGVIMVCLVIAIVVIRRQDTERRNHKYNCRMEALKMLHATRENHKEPEMESTSSKALSNGSCSSDCDKPAKKEVSFNLDMDDRDCVEYGHVNDRLQRSWPSTIDHKTLETPQACSSMLTTASSNGSDIRPSRNNNNASRQPPPPNNAAAAGRPPWPLTQRELEVHQLLQMLKDQDADSAYSGDGSNTDSGRGPSEEGEPDKCGGGRRLAHSPQDEGLQVVPQLSANNSLNVSQVTNNQSIHSYSQQPLCLQTFAPAPRPGEAATLERRGQLNTGFIGTTPGGTDISPIKPSTFAKQYSPDGKSPYQQGPHGHPQENPYGTIQKKKAPQQQPQQPPAYGGHNNAVSPYSNGHYGGRGYHSDAETEETLASDDDGAMQYSCADMSDGTYIPTPRSLQVADNTADDATTTSGSYVVDPQDLCQEIDDLFFRDMVV

Domains

Cadherin Repeat (7x): 33 – 140, 152 – 250, 258 – 358, 374 – 468, 476 – 571, 581 – 678, 691 - 780

Clone

Partial ORF

Primers

Forward: TGAGAGCAGAAGTGACAGTCG

Reverse: GTCGGCATTCTCAACCACTT

Nucleotide# 421 – 1621 = 1201 nucleotides

>Pcd11x_clone

TGAGAGCAGAAGTGACAGTCGGCGCAGTCGGTGATGTAGCGACCCTTCATAGAAGATGGCTCTAGTGTTGGAATGGCCCGGCATCTTGCTACTGCTGCAGGTGATGGTGGTGGCACTGATGCGATCGGCCATGGCTCAAGACAGCCAGCTGGAGTTGACCTACTCTATCCTGGAGGAAGACATGGAGGGAACCTTCGTGGGCAACGTCCTACGTGACTCGCGTATCCAAGACCGCTACTCCAAATCCATTCTTACCCAACTTGAATTCCGCTTCATAGCGAAGCCGCGGGTGCCCTTCCTGATCAACAAATCCACCGGGGTGATCCGCACGGACGGACGGATAGACCGTGACGCTCTCTGTCCCCGCCAGGACACTTGCCGCGTCATCCTTGACGTCGTGGCCTTCACTCCGGGACAGACTCAGTTCCTCGAGATAATTAAGGTCAAAGTGAACATCGAAGATATCAACGACAACTGGCCTCATTTCCCTGATGCCCGCATCTCCCATCAGATACTGGAGTCAGCCGTGCCTGGGTATGGCTTTGTTATTCCCACCGCAATAGACCCAGACAGCGGTCAGTTCAGCGTGAGAGACTACGAGTTAGTTTCAAGTAGTGATAAGTTCGAATTGAGTGTTAAAACTAAGTTGGATGGGTCCACAGATGTGCGACTCATCTTAAAAGACTCCCTAGACAGAGAGGACGTGGATTTTTACCAAGTGAGAGTGGTTGCCTATGATGGAGGTAATCCGCCCAAGTCAGGTTCCGTGGATGTTAACATTATAGTACAGGATGCTAACGACAATGATCCCATGTTTACCAACGCTACCTACGAGGTTACAATAGCCGAGAATGCTGCTCCGGGGACCACTCTGACCAAGGTTCATGCGTCTGACAAGGACGCTGGCCTGTACGGACAGATCCGATACAGCTTCTCGAGCCGCACCCAGGATACGCATGGTGCCGTGTTCGGCATCAAGAACACAACCGGTGAAATTTACGTCAAGGGCGAGGTGGACTACGAAACGAGCCCTGTGTACCATCTTGTGGTCCTTGCGGATGACAGGGGCTCGGATACAATCCCTGCAGAGGCAACAGTCATTGTACGTGTCCTGGATGTCAATGACAATGCCCCTTTGATAACGGTGAATACCTTAACACAAGCGGGAACTGAGGTCGCAGAAGTGGTTGAGAATGCCGAC

Cloning info

PCR rxn on 4/24/15 using GoTaq polymerase mix

| Template | Initial denature | Denature | Anneal | Extension | #cycles | Final extension |
| --- | --- | --- | --- | --- | --- | --- |
| 2 hr cDNA | 4min @ 94C | 30sec @ 94C | 30sec @ 55C | 2min 15 sec @ 72C | 35 | 10min @ 72C |

Purified PCR Product with Thermo Scientific kit on 5/6/15, Second PCR from purified template on 12/1/15

Ligated PCR product into Pgem T Easy vector and transformed into competent cells on 12/2/15

Picked colonies for overnight cultures on 12/7/15

Isolated plasmids using Promega miniprep kit on 12/8/15

Confirmed clone Pcd11x by sequencing on 1/15/16

Pcd11x is sense to T7. RNA probe generated using SP6 on 1/20/16

**Numb**

Gene model: 4674 nucleotides

ORF: 345 – 2063 = 1719 nucleotides

>Numb_full

GCGCGTGCGCACTCGCAGTGACTCTAATGTATGTGTCGACTCGATTGTTTGTTAGAAAAGCACTGAAATTTCGCCGTATTTGAGGATTGATACGAGAACGGTGGCAGAAATTGGATCGTCTGGACAGTCATAACAAAGTGTCCAGTGATTTGACTTCCTTTGAGGCTATGAAATCGTCTCGGAGGAAAGGTGACTGTCGGTAATTCCGTCTTGGTATTTGGGGGCTGGGTCGACACCCGGACGATCGCAACCTGCATTCTTTGGTTGACCTTTTAATTTTGCACCGTGAATCCCAGGTGTTGACATTCCTTTATACAGGTCAAAGCCTTAAAACCTGATGTGAGATGGAAAAACTGAAACGGAGCCTTAGTTTCCGTAAGAAGAAGGACCATGTGCCAGAGAGTAGCAAGCCTCACCAGTGGCAGGAAGATGAAAGGAAAGTTAGGGAGGGTACATGCAGCTTCCAAGTCCGGTACTTGGGCTGCATTGAAGTTTTTGAATCCCGAGGAATGCAAGTTTGTGAGGAAGCAGTAAAAGCTTTGAAAGCGAGACAAAATAGGAGTGTGTGGTCGCGAATGTCTCTTAGGAGAAAAAAGAAAAAGCCAGAAGAGCAATGCAAAGGAAAATACCAAAGAGCTATTCTTTATGTGTCAGGAGACGCGCTCCGAGTGGTGGATGAAATAAGCAAGGGCCTCATTGTGGACCAGACTATCGAGAAGGTGTCCTTCTGCGCCCCCGACAGAAACCACGAGAAAGGATTTGCCTACATCTGCAGAGATGGTACGACCCGCCGATGGATGTGTCATGGATTTCTTGCTCTCAAAGAATCTGGAGAGAGACTGAGCCATGCTGTGGGCTGTGCTTTTGCAATCTGCTTGGAGAAGAAACAGAAACGTGAGAAAGACGCAGTGCAAGTGCAGTACAATGAGAAGGGAACAAGCTTTTCAAGAACAGGCTCCTTTAGACAAGCCACCTTGACGGAAAGGTTGTGCGATCCTCAGAGCACCATCTTATCAGAGTCAGTGCCAGTGAAGGCGGTGGACAACCCCCATGCAGTGTCGAGACCTCATGCAACGGCCGACATGTTGCAGCGACAGAGCTCCTTCAGGGGATTCACCAAGCTTGGCGACCAGTCCCCCTTCAAGAGACAACTCTCCTTAAGGCTAAGCGATTTGCCATCCACCCTGCAGAGACAACAGGACATGAACAACCCTGCTAACGGAATATCCAATGGCCTGTCTGCCAGTCCCATTCCAGAGGCTTCTCCTACCAAGGAGAATCCAGACACGATTGCGGAAATGTGTCAACAGCTTACACAAGGCTTGACAGCTCTCCAGACAGACGACCCTTTCGCGAATGTCCCCAACGTCACCCAGCGCAACATTACCTCTCCCACGTCAACCGTTTCGCACACTCCCTCTCCGCCTGCGACCGCTCCTCACCAACACACTGTGACCGTTCAAGGAAGAAGCATTCCACTGCCCGACCCCATTCGACAGATGAACCCTTGGGCTCCCTCTGGAGGTCCGCCTGGCAGACGCCCGCCCGGTGGGAGTGTATCTGACGCGGAAGCATGGCTGAGCAACACGGCCAGTGAAGTTGGGCGACCAAGTGAGGTTGGGCGACCAGAGCCATATGCTGCAGCCGGGCAAGCCCCGGGCTACGTGGCAAATGGCAACTCCATGAACCAATACAACGTGGGACCAGGATACAACACGGGGGCGGCCTATCCAGGACAGGTCGCCAGCGTGGCTCAACATAGAGCCCCTCATCTCACTCATCTACGAAGTCATTCCATTGATACAGCCCAGATCTGGGACCAACAACAACAGCAGCAGCGTGCCCCGACACTGCGCCAACTTGCCCACAGTGGTTACCCCATTGGACAGTTCACCTCTCAACAGAATGGAGCTGGGGGCACATGGAGCCCCCCTCCTCACGCCCCCCAACAACAGGCCTTTGACCCGTTCGACGCGGCCTGGGCAGCCAAGAAAGTAGGCGTGGCTAACCCCTTTAACGGCCAGGGAGACACTGTTAGTAAATCATTTGAAGTGAACTTGTAAAAATGAAGATTCACCTCAGACTTTTCATTCCGCACTTCTTGGGATTCCTCATTCAGGTCAATTGTGCAGTCCTGTTGCTCCCCATAACTCGTGCTTCATAGGAAAACTAACACTCTGAAAGCATTTACAGTGTAACAATGTCATATTCGTTTAAAAAATAGCCTTCGGTTCAAAAAGTCTAAAATTTCATCAGATGTGGGGTTTCAGTGCTACTTAGTATTTCTGCTACAATCAGCGATGCTATTAGGTGACCTTCCAGTTCCCTCTGCCTACTACACTGTGCTGTTTCATTAGTGAAATTTCTTGATACTAATGTGCACCCTTATGCAACTTTGAAACGGGAGCAAGGTGTGCCTTTTGTTATCTTTTATATGTATACACATAAGAACAATGGACTTACCAAAGCTTACATGATTTTGGTCTTGCAGTTCCTGTACAGGGAAGGTCACCCATTAGAGCTGACTGACAATTTGTACATTTCTCCTGTTAGTCTTGTACGTGATTTGCATGGCGCGATGCAATAGCATGACAACCAGACAGCAGACTGGTGTTGAAGGTTCAAGTGCAAAGTTTGGTAAGCTGTGATGAGCTGAGGCTGCAGATTTCCCCCAAAAAGAGTTGCTATTCTGGTGCTGTACATAATCTAAGAAACTCCAATATGCGCACAGTAACAATGTGAAGTGAATGTACTGCTAGATGCCGAGTCACACCCGCACGACCTGGTTCCTCAGCCTTGCCCTTTTTTAATTACAGATGTACTGGGGTCTGCAGCCTAGGATGTGATAATTTTTATGATGTTGATTCAGTTCAATGGTTGAATCCTATAGATACGGTAGACTACTACAATCGAAGCAATGAGATATATATTGAAATGAACTTAGACGGCTTAAGTGTGACTTGTAACTTGTGCTGTGAACTTTTTGTAGAGATGAAACGACACTTGCAGAGTGACTTTAACCAGTAAATATTAATTATCAATTTCAAGCCAGTGGCTTTATGGTAATCTTTTTATGTCATATCCTGTGCAAATGCACAAGGTTGCCTTATTATTTATTCTGTTATACCTTTACCGTTATATACAGTGTGGTTTTAAGTTTCTGTTACCAAACATGACCAGCCTAAGGTTGTAATTATCTTTTTGACAAGTGCATGCATTAACAGTTATCATACCAGTCCTTGGTAAATTTTTAGCATATGTACTCTAGAAGTGCTGAAGTTGGGGTTTGATGGTAGGTTTTTAGTTACAAAATGAACATATCATTGCGTTATACATGTGTTTTTAGAAGTAGGCCGTTACAGTGAATGTAACTCCCCTATGACCTTGAACACAGATCTAGCATTTTACGAAGCATTCCCGATATTTTAGCTCAATGTAAGAAGTGGAAGGCAATAAGATTTTCTTCTGATCTTAAGTGATGCCTCTCCAGGTACTCTTCATCTGCATCCTCGCAGGCATTTTAAGTTACGGTGGACCATTTTATCATTACTTCAGTTTTGGTAATCAATCTCATTATTTTGCAATTATTCGGCAGGTTGTTTCTGTTTATGTCTGAATACAAGTGTTTTATCATCAACTTTTATGTTATTGCTTCATAACTCTTAGGCCAGTGTAGGGAATCAACAATTAGTATTATATTCACTTTCATTTGTTCTAACAATTAGTCTACATGTACTAACTAATTTCATACATTTAGGCACTGTTGACTCTATTGAGGTAAACTGGCTTTTATCATCATCTTTTGCATAATGAAGTAATTGGATTATGTGCTGTTTTAGTGCTTTTATCCAGAGACATAACTGTTAGTTAGGATATACTAGCAGAAGTGTTATGTAAATATTATCTAAACGTCAGTTATGTACATTTTTGTAAACCATGTTTTGTTTGTTGAAAAAATTTGGATCAACTTCGAATGACTAGTACCAGTAAACTACGAATCTCCAAAAAATACTGCAATGCTAGTTTTCAGTCCTTTGTCAACGGTGGTATATAGCTCTCAGTTCTGAAAACTTCTTAAGTGTACTTAATTTACATGCATGTACTAATACCCATTTAATAGGAGGAACTCTGCATATTCCAACACCTTGTTAATTGTGGTGATTTGTCTAAAATAGCCTGTGTATCTGTTTCCACACCACGAGTTCCAGATAAGTACTTTGCTGATGGATTAGACTTTAATAGCATATGGCATAGAGTATGGATAAAGTTAGGAATTCTCTGTATCATATTTGTACATAATGGTAAATATAGATTTAGAGGAATAGCACCGTCACATCATAATGTGTAATTGTATCAAATACCAAATTGTATCCGTTGCCATGTCTGTAATGGTCTGCTTATAAATTTAAAGTTGATCATTATATTATTACTTAATAATCAGTAGTCCAATATTGAAGTAAATAATATTACTATACGTCAGAGACATTTGGCAGAAGTATGATTAAGAATGGTAGCATTATTGAATTCCAATTTGATTCCTTTGGCAGTACCACATTAAAAAGGACAAAATTATGTCTGGTATAATGAAATATATATATGCCCCTGCATTAATTATATTTTACATAGATTGGAGAAGGAATAAAAAAGCTTATATAAACATTTGATTTGTTGTCT

Protein: 572 aa

>Numb_protein

MEKLKRSLSFRKKKDHVPESSKPHQWQEDERKVREGTCSFQVRYLGCIEVFESRGMQVCEEAVKALKARQNRSVWSRMSLRRKKKKPEEQCKGKYQRAILYVSGDALRVVDEISKGLIVDQTIEKVSFCAPDRNHEKGFAYICRDGTTRRWMCHGFLALKESGERLSHAVGCAFAICLEKKQKREKDAVQVQYNEKGTSFSRTGSFRQATLTERLCDPQSTILSESVPVKAVDNPHAVSRPHATADMLQRQSSFRGFTKLGDQSPFKRQLSLRLSDLPSTLQRQQDMNNPANGISNGLSASPIPEASPTKENPDTIAEMCQQLTQGLTALQTDDPFANVPNVTQRNITSPTSTVSHTPSPPATAPHQHTVTVQGRSIPLPDPIRQMNPWAPSGGPPGRRPPGGSVSDAEAWLSNTASEVGRPSEVGRPEPYAAAGQAPGYVANGNSMNQYNVGPGYNTGAAYPGQVASVAQHRAPHLTHLRSHSIDTAQIWDQQQQQQRAPTLRQLAHSGYPIGQFTSQQNGAGGTWSPPPHAPQQQAFDPFDAAWAAKKVGVANPFNGQGDTVSKSFEVNL

Domains

PTB_Numb: 24 – 180

NumbF: 235 - 327

Clone

Full ORF

Primers

Forward: CCCAGGTGTTGACATTCCTT

Reverse: GCGGAATGAAAAGTCTGAGG

Nucleotide# 292 – 2096 = 1805 nucleotides

>Numb_clone

CCCAGGTGTTGACATTCCTTTATACAGGTCAAAGCCTTAAAACCTGATGTGAGATGGAAAAACTGAAACGGAGCCTTAGTTTCCGTAAGAAGAAGGACCATGTGCCAGAGAGTAGCAAGCCTCACCAGTGGCAGGAAGATGAAAGGAAAGTTAGGGAGGGTACATGCAGCTTCCAAGTCCGGTACTTGGGCTGCATTGAAGTTTTTGAATCCCGAGGAATGCAAGTTTGTGAGGAAGCAGTAAAAGCTTTGAAAGCGAGACAAAATAGGAGTGTGTGGTCGCGAATGTCTCTTAGGAGAAAAAAGAAAAAGCCAGAAGAGCAATGCAAAGGAAAATACCAAAGAGCTATTCTTTATGTGTCAGGAGACGCGCTCCGAGTGGTGGATGAAATAAGCAAGGGCCTCATTGTGGACCAGACTATCGAGAAGGTGTCCTTCTGCGCCCCCGACAGAAACCACGAGAAAGGATTTGCCTACATCTGCAGAGATGGTACGACCCGCCGATGGATGTGTCATGGATTTCTTGCTCTCAAAGAATCTGGAGAGAGACTGAGCCATGCTGTGGGCTGTGCTTTTGCAATCTGCTTGGAGAAGAAACAGAAACGTGAGAAAGACGCAGTGCAAGTGCAGTACAATGAGAAGGGAACAAGCTTTTCAAGAACAGGCTCCTTTAGACAAGCCACCTTGACGGAAAGGTTGTGCGATCCTCAGAGCACCATCTTATCAGAGTCAGTGCCAGTGAAGGCGGTGGACAACCCCCATGCAGTGTCGAGACCTCATGCAACGGCCGACATGTTGCAGCGACAGAGCTCCTTCAGGGGATTCACCAAGCTTGGCGACCAGTCCCCCTTCAAGAGACAACTCTCCTTAAGGCTAAGCGATTTGCCATCCACCCTGCAGAGACAACAGGACATGAACAACCCTGCTAACGGAATATCCAATGGCCTGTCTGCCAGTCCCATTCCAGAGGCTTCTCCTACCAAGGAGAATCCAGACACGATTGCGGAAATGTGTCAACAGCTTACACAAGGCTTGACAGCTCTCCAGACAGACGACCCTTTCGCGAATGTCCCCAACGTCACCCAGCGCAACATTACCTCTCCCACGTCAACCGTTTCGCACACTCCCTCTCCGCCTGCGACCGCTCCTCACCAACACACTGTGACCGTTCAAGGAAGAAGCATTCCACTGCCCGACCCCATTCGACAGATGAACCCTTGGGCTCCCTCTGGAGGTCCGCCTGGCAGACGCCCGCCCGGTGGGAGTGTATCTGACGCGGAAGCATGGCTGAGCAACACGGCCAGTGAAGTTGGGCGACCAAGTGAGGTTGGGCGACCAGAGCCATATGCTGCAGCCGGGCAAGCCCCGGGCTACGTGGCAAATGGCAACTCCATGAACCAATACAACGTGGGACCAGGATACAACACGGGGGCGGCCTATCCAGGACAGGTCGCCAGCGTGGCTCAACATAGAGCCCCTCATCTCACTCATCTACGAAGTCATTCCATTGATACAGCCCAGATCTGGGACCAACAACAACAGCAGCAGCGTGCCCCGACACTGCGCCAACTTGCCCACAGTGGTTACCCCATTGGACAGTTCACCTCTCAACAGAATGGAGCTGGGGGCACATGGAGCCCCCCTCCTCACGCCCCCCAACAACAGGCCTTTGACCCGTTCGACGCGGCCTGGGCAGCCAAGAAAGTAGGCGTGGCTAACCCCTTTAACGGCCAGGGAGACACTGTTAGTAAATCATTTGAAGTGAACTTGTAAAAATGAAGATTCACCTCAGACTTTTCATTCCGC

Cloning info

PCR rxn on 11/25/15 using GoTaq polymerase mix

| Template | Initial denature | Denature | Anneal | Extension | #cycles | Final extension |
| --- | --- | --- | --- | --- | --- | --- |
| 2 hr cDNA | 4min @ 94C | 30sec @ 94C | 30sec @ 55C | 2min 15 sec @ 72C | 35 | 10min @ 72C |

Purified PCR Product with Thermo Scientific kit on 11/25/15, Second PCR from purified template on 12/1/15

Ligated PCR product into Pgem T Easy vector and transformed into competent cells on 12/2/15

Picked colonies for overnight cultures on 12/7/15

Isolated plasmids using Promega miniprep kit on 12/8/15

Confirmed clone Numb by sequencing on 1/15/16

Numb is sense to T7. RNA probe generated using SP6 on 1/20/16

**Brat**

Gene model: 12225 nucleotides

ORF: 167 – 2494 = 2328 nucleotides

>Brat_full

GCCACAGGTGGCGCACGAGTTTCATTACCCATGCACTCTGAAAACGAGGCAATCATGAACACAAGAAGATGGCGGGTTGAGGAAATTTCGATGTCTTCCTGGTAATTGCTTCAAAGATAACTCTCTAAAGAATTTTTTGCGCTGTATCTGGAGAGAAAAATTCAAGATGTCTTCGCCAACGCTTTCTACAACCGACACAATGAGCCTGACCTCTAACTCATCCGAAGGTGACATTGAAAACAGTCTCAACACCAAGTGCAGCCTGTGCAATGAGACCTATAGCATTCCCAAGGTCCTGCCTTGCTTTCACACGTTTTGCCAGCCATGCCTGGAGAAGATCATTGAGACACCAGATAAACTGTCATGTCCTGAATGCCACCAAGAAACTTTCCTGACTTCCGCAGGAATCGCTGCCTTCCCCCCAGACTTTGCTGTTAACAATGTCCTCGAGGCCTCAGTTCTGGAAGGAGCCACCCTAAGCTGCACTGGATGCAAAAGCAAGCACCTCTTAGCTGTAGCTAGGTGCTTCGACTGTCCCGACTTCTTGTGTCAGGACTGCGAGAGGGCCCACCAGTACATGAAGCACTTTGACGGCCACCGAGTCATCTCCTTGGGAGAATTACAGAACAACAAAGAAGAATACAAAGTCGAGAAGCCAGTCATGTGCAAGAAACATCGCCAAGAGATGCTGCGATTCTTCTGCCACACGTGCAACATCCCCATCTGCAAGGAGTGCACCCTAGTTGAACATTCCAAAGGCCACGAATACGACTACCTGTCAGAATGCGCTCGTCGCGAAGTATACTCCCTTCAACACCTGGCCGAACAAGCCAAGGTCAAGGCCAACGATCTCAGAGGGTCATCAAAAAGTCTCGAGCATTCGTCAAACAGACTCCAGATCCAGTACCACAAGGCGCAGAATGAGATCAACGAAACTTACAACTTCTATCGTTCCATGCTGGAAGAACGCAAACAGGAGGCTCTCAAGGAATTGGATGGCGCCTACAATGCCAAGCAGTCCGGCATCACCAACCTGTCAAGCCGCATGCAAGAGTACATCGAGAAACTGTACCAGGGTGTGGAGTTCATTGACCGCATGACCAAGCATGCCAGCAGCACCGAAGTGCTCATCTTTAAGAAGATGCTAGACACCAATCTCCAGAAGCTCACCTCATTCATGCCCGACACCAACAGCGTCACAAGTTCCTTCGACCTTGAGTTTGTCTCAAACTACCAGGCAATCCAGGTTGGCATCCGCAACACATTTGGCTACGTCCGCACCAGTTCAGAAATGCAATCGCGACAATACCCTCAACCCATCGCTCGTCCTAACGGATTCTCCGCTCCCCCTCGCACCATCACGCCCCCCAACCCCCTCGCTGCCAACATGCCTAACCCCATGGTCAACTGCAACAACCTGTTTGACCCCACTGCCTTGCTCCTCTCCAAGAACAACTTCCCCTCATCTGGGAGCCTGACCCTCGGCAGCTTCACTGACCCCATCATCTCTAACAACCTGAACCCTTACGAGAAGTGGTCCAACGGCGGCCTTGATTTGCTCCACAATGGAGATGTGTTCAGCACCAGCACTGATCCAGTGATCGACCTCACGTCCAAGCTGATCAGCGCCAACATCTACCCACCCAAGTCCCAGATCAAGCGCCAGAAGATGATCTATCACTGCAAGTTTGGAGAGTTCGGAGTGATGGAGGGCCAGTTCACAGAGCCCAGTGGAGTTGCAGTCAATGCCCAGAATGACATTATTGTAGCAGACACCAACAACCATCGTATTCAGATCTTCGACAAGGAGGGTCGATTCAAGTTCCAATTCGGCGAATGTGGAAAGCGCGACGGTCAGCTGCTCTACCCCAACCGAGTGGCGGTCGTCAAGACCTCCGGTGACATCGTCGTCACGGAGAGGAGCCCCACGCACCAAGTGCAGATCTACAATCAGTACGGCCAGTTCGTTCGCAAGTTCGGAGCTAACATCTTGCAGCACCCGAGAGGTGTCACTGTTGACAATAAGGGAAGAATCATCATCGTGGAGTGCAAAGTCATGAGAGTCATCATCTTCAGTCAAATGGGTGACGTCCTGCACAAGTTCGGCTGCTCCAAGCACCTGGAGTTCCCCAACGGAGTCGTCGTCAACGACAAGGAAGAGATCTTCATCAGTGACAACCGCGCTCACTGCGTCAAGGTCTTCAGCTACCAGGGGGTGTTCCTGCGCCAGATCGGAGGCGAGGGAATCACCAACTACCCCATCGGCGTCGGAATCAACCCAGCCGGAGAGATCCTCGTCGCGGACAACCACAACAACTTCAACTTGACCATCTTCACACAGGACGGCCAGATGGTGAGTGCCCTGGAGAGCAAAGTGAAACACGCCCAGTGCTTCGACGTGGCTCTTATGGATGAAGGCTCGATCGTTCTCGCCAGCAAGGACTATCGTCTCTACATATACCGCTATATGCAAATGCCCCCTATGCTCATGTAGGTGGCGTTAGTGAGGAGCCTCCTCGTGTATTGTTGTTTGAGCCAGCTCTAATATTGGTGCCACATTCATAGGGCTATATTTGTTACTAGTATATTCATACATACTTTATATACTTCACTTGACCAGATTTAATATTTTTGATTGAAATGATGGCTGATATTTATATATCTGGTACAGCTGCTCATAATACTCCGGAATCAGGATTTAGATTTTGTTTAGGAAAAAGGTTGTTTCTGATAAAGGATGGTTTCCTCTGGCAAATCGACCCATAGCGGTGGCTGTACCCAGACGTTGTTCAGTACGTGAATTAGGTGACTAGTTTGATATTAATGTGAATGGAGTCATGCCTTCAGTGGCCTTGTGTTGTTAGTTTGGAAAGCAATTATTATTATCATATTATATGAAAAATGAACAAGCTAATGCTGAATGTTATGTGTTGTAATAGTATTATATGAATTGTTTGATTTTTCTGAAATGTGGCTCACTAATCATTGCTTAAAAAGCAATTTTGATAAGTAGCGTAGGTGGGAAGAAGCCATTTTATACAGAGTATTCTCGTCTTTTATGATATTTTCTTAGCTTGTTGAAGATAAAAAGAGAAAATCCTTAAAAAAATGCATTTACCGAACGCCATATCATCAATAAGATTTCAACTTGATGTTATAATTATTTTTTTGTATAATCAGCTGGAAGGTCACTTTTGGAACCTTTGTCGTTTCCAAGGAGATTTTTTGATAGCGTTTGTAATTACTGTTTCTTGTTACACGTAATATAGTTTGTGTCATTCTCAGATTTATTTACTATTGTTCTGTGCAAGTTATGTTTGGTAGAACGAGAGTGGGCTAGAGCAATCTGTATTACTCTAAGCAACAGTAGTTCGGTCTGCTGTTGGTTAAAATACAGTTGCTCTGCCCGCCTCGGTCTGATTGTTGCTATGTGGTATGTGTGATATGTATATTTGAGTAAATTGTGTATCAATGCCATTATTAATTTCGCTACCTCGTTGTAATCGCAATATATTATATTAGTAAGATTACTTTAATCAAGTCGTGTGGGAGTTGCCCTGTTTTAGCATCTTGTTGCTTGAAATGGGCAACGTCGGTTGCCCGAGGAGTGAACATTTCATTATTATGATTATATATATATTGTATTATCTGTTGTGTTTTTGCTAAGTTCTATTTTCATATATTGATGTTATTAAATACTTAATTGCTTTAGTTATTACATTGCAGACCAGGCATTGTTGAATACGACTTGCAGCGTTGAGTTGAATGTTGATGTTTAAGCAAGTCTCATAGAAGGGTCACACTAAGTGCACAGGGTTCTGACGCAATGCCCTATGCTCGCAGGTTGTATACCCATAAGTTGTTTTACCTTGATATTATTTATTAAGATGCTGTCTGTATGTGCCTCGCGTTTATTTATATGTAGCCTTTTTTGTAGTGTGTTGGATCTGAAATTATTTAATTTTTATTAGAAATTATGAAAAAGGCATACAATGTCTTTCCTCAGGTCTTAAATTATGCATAACTTCTGTCTTGGTAAAGATTAGGAAAATATTTTAAATTATTTTATCTTAAGTTATTCTGTTATTGTGTGATGTAAGCTATTAACTGGCTTGGATTGGATTGCTTGGTGTTATATCAAGTTAATGATTTATTTACATGTTATTTTGGTGTTAATTGAACCAAAACTCTTCAGTAAGATCACGGCAACCAAATGGGAGTGTCATTTTTGTTTGGGTAGAATTTTTTACAGCATTTTTGATTACCTAACAATAAGCACAATTACTGTCTCATGTGTGGGCATATGATTCATTGTATGATTCGTCGTAAGGTTGATCACATATGAATATAGCCTCAACCCGTTGTTGATGTGATTATCGTAGTGAATGCTTCAATGGCCTGACAGTTGAACGGACGACCATGATGTTATGGCTGTCGTGCCCGTTTGTGGTAACTTGCACCTCAGCTTGTGTAAGCTAGTCATTCCACAGTTTAGTGATCCTTGCGTCGCCCCCTTAACTCGAGTGAGAGCTGTGACGACGTCGTCTGACGGTGTCTGATGGTGTCACCTGACTCGTCGCGTCTATCGGACTCGTCTTTCGGCGTCGTCAGCTGTTGTTGGCCTTCTCGGGCGGGGGTCGCGGGGAATGTATGTGGCTGAAATGTGATGAGCAGTTGTACTGTTGGTACGTCGCAATAGCAAGGAACCCCGCCGTTGTTGTTGGGTCCTGGAAAAAGTGACGATGCATTTGTTGTTGTCTCATGTGTGAGCTTGTATATTAACCCTTTTCATTGCTGTTATGCGGTTCCAAAGATGAGTTTTATATACCTAGTACTTTGTCATAGAACTAGATGCTTTATATTTAATCGCTGAGGAGTATTAAGTACTATTAACATGTATCATTGTTAAAATGAACACACATAGTTATGATAGTTAGAATGGCAGTAGTTGGTGTATTTGTACTTAGATCGACGGATTGAGCAAGGAGGTGTGCTTGGACGTATGCTTTCTATAGTATTTTTTTTCTACATGAGTGTTGTGGTGTGTCGTAGATCACTTGAATTAGAGCCCCATCTTTGAGATGTCCATGGATGACGTTCTCTCCGTGTTACGGAGATCACGGAGAACATGTGGCTCACGTCACTAAGTTCAATTTACTTGGAAGCTTGAAGGTTGGGATGCAAAACGACTTTGTATTTCAACGTCACCAAAGTATTTATTTTTAATAAGATTTTGTACTGTATGCTTGTATGATTACTTCTGTTCGTATAGTGTTCAGCCATGACACTTTTGCTATAGATTTACAACAGAACGACTTACCGATTGTTTCCTTTTCATATATTGTATTGTTTCTTGTAACTGATAGTTTACAGACATTCAGAATTCTTGCTCAGACCAAAATGCTGTTGAATATATCATACTTTTTGTTGATACTTGATGCTGGCTTAATTTCAATGGTCAGGCTCTGAGGCTCTCTAGCCTCCATGAGATTTCGGATAAGTCTGTCTTTGGATCATGTGCTCTTTTTCCAACTTTGAAAAGCTGTAAGATATCTTCGCTATTTGCAGCTTGATTTGCAAACGCTTAAGTGATCTTTCCTTTTATATCATTAACTATGGTTTGCCAAGCAAATTTTGATAGAAAAAAATCTCGGAAAAGATGAAGAGTTAATTACACACACCTTTTCCTATTGAACAATATTTTATATTCCGAAGGGTTTCCGCTTTTTTTATGCATAACTGTGGATGCCTTAAGTTTTTTATACCTCTCTGACGCTTGATGTTCTCTGAATATTCTTTGGATATTCTGTGAATATTCTTTAGATATTCTCTGAAATTTACTTTTTAGGATTTTCTAACGTGCACCAAGTCGGTCTTTCACGGCCTACCTGACTCTCTTTGCCGAGTCCTTTCTTAACCTACCTTCCTCTCTGATGAGGCACACTTAAGATCTGAGCTTAATTAGTTGGAAAATTCTACTTAATTAACCTGAAGTCTAAAGTCTGCTTTCGATTTTTCTACCAATCATTACCAATACTAATGCGTTCCAAGATGTTGAAATTTTGCGAGTTAAAAGTAAAAAGGTTATGGTGTGCATGTAAATTTTTATAGGGAGTTTTTGCCATGTTAAGTTTCATTGTATTCTTTACATTTTTGGCGTTTTGTTATTTAGTATTTAGCATGTTATGTTGTTTTACACTTGTACAGTAAGGTTTTCCTGAGTGTGAGGCTAATTTACTAATAATTCAAATCAATTGAAATTAATTAATCAAATTATTAATCATTATTAATCATAAGTAATCCGATGATTGTCAGATAATTGTCAGAATTAGCAAGGACTGGGAATTGTCATAGGCTACTGGTTGACGTGAATGAGTCGAGTATTTAATGTTGCATAGTTACCTGTTAATTGATTGTTATACAAAGTTCCTCACTGCCATAATAAGAAATAAGGTGCAAAAATGAAGTTGTTGATTGTGAAGAAGAAATGTAATTTTCTATTTTTGTCAAATTTCTATCTTAGTCATTCATTCAGTTCACCAGACCAATCAAGATCAGAACAAGATCGTTCAGATCTAGCTTGCAAACGAATCATAGTTGTTAATCAGTTGTTTTCTAGGGACCAGAGATGCATTTGCCAAAGTGTTTTTGTGAACATAGCTGGTTGCCACGAGCTGTATATCATGTTGAAAGCAATAAGTGACCCTTTTTGGTTCCCTCTCGCGTTCTGTTATCCAAGATAGAGCCAGGTGAGCGATGGATGTTCTAATTGTCATGGGTCGGAGGTTTCGGAGTGGTCGGTCCTGTGGGATGCGGTCTCTTTAGTTTTCGAAGGTTTTTAATTGTTTCTGTAGCTTCAGCGCAGATTGTTACGCTTCTAATTGCCATCAGCAGCAGCTGCACCCGAAGAATGACCAAGTTTTGGGTAAATTAAATTCAGTATTTGTTACAATTTGTGACCTAAGTACGCACAATTAGACACCTTGTGAGTTTCTATACACTATGCAGCCGCCTTTGGTTATGATTGCAGAAAACTTGTCCCTATCCGTGCACTTTGATGCTTGCGACATTGGACACTTTGTACTCTCAGGTTGTCTTAAAAGCAAAAACGCCCCAGAAGTTAGAATTGCATTCAATTGGTCTGCCTTGCAAGCATAAATTAGTAATAGGGCTTCGCCTAGATAAGCATTCCCATAACTGTGATTATTTTGTAAACGGATGAAACTGTCAAAAACACCACCATACCGTCAGTTTCTCAATCTTGATACGCTCCGCCACATGTACGCTAATCTTTTGTTTATTTGTTTTCGCTATATCGCCTGTCTGCTATGCATAAGGTTTTTTCCCAGTTTGTGGTATTTGGACGTGCCTGCCAAAACTACTTCCCAGTCCTTGTTTAGCTGCCATTCCCTCAGAACCTGCCCTCCTTAAGGTAAAGCCCCTTGGCGTACCCCCGGGGGGAGGTGTTATTTCCATGTTGTTTGGGTCCAATCACAGATATCACAGCAACGCTGATGTTATTGCATTGCCTTCTCTTGTACTTTTAGTAATTCAGCTAATAAGACATTAGGTAGTGTTTCTATATTATATATATTACGAACTTTAGAACAAGTCTTTAGATATGTTTAGCTATTATAGTTGAAAATATATGAAAAAAAAACAAAAAAAAATTATTCTCAAACTTGATAATATTATCTGAATGCCTGTGCTGACAATATGTGGTCTGTTTGGTGATAACTTGCCAATATTCAACAACAAAAATTGTTACTCCATAGTTGTGTGTCTATTATAGCCGTTATGAACATTTGTGTTAAGTGTCGTGTTGAGCTAGGTAGACCGTTGCCTCCATGTAGTCCCAATCATGTTAAACATATCTTTAATATTGTATTAGCTTTGTGGCCGAAGCCATGATGAGTGAAGTATTGTGATGTTTCAGTTGCGAAAGATGTTTATGAAGTCATCTGGATTGGTAGCTTGTTGTGATTGTGCTGGTGCTCGTGGTGTCAAGCATGAGAAGCGCAATGGTCAGAGATTTAAGAGTTACGATCAAATTTAAGAATTATTGAAGACAATCAAGGATTAAGACATCAAATTTAAGATTGAGACATCAAATTTAAGTGTTACGACCAGATTTAAGGATAGAGAGACATAGAAAGTTAGTAGGGGAATGTGAAATCTGAAATGCCAGACACGCTGAGCAGATGTACATCTGTGCTATGGGCATTGTGTATTGTTCAATGTTAGTGAAGTGAAGGGCATTAAATGTTATTCATGGTTCGGCTGAGTTATCCAATGGCCACGTTTCTATCATCTACCTTAATACCACTCTGTCTTGGATATTGTTGACTTTTTGTTGTGGCAATTAAAATATATATATGTCCGTTATTTTATCCATAGTTTAGAATGCACTTGGTGTTGGTGGAAAATGCAGGGCTTGTGTGTTGATATGAATTCTGGTGTTTATACCGATATATAGCATGCACAAGATTGTATATTTGCAAGAATGTGTGAAGTGGCATTTTTTATTAACATTGGACATCATATTAAAATGTCCTTCCCCTTATATTATTAATCGTTGACGTTGGCACCAGATGTCTCGTGTGTGTTCTCTTGACCAGTGCTGCCCTCAAGTGATAAGTGCTTCTGCAAAGTAAGGCCGTTGTTCTTTTTCTCTTGTTAGTTGCTAGGGAGGGTCTCTTCCCCGTTCCGGGTGTCTCTTTCTAGCTGATGATGAATTACGCCAAGTAAATTGCAAAAATGACGGTAGAACAAACTTGCAGGCCTCTGGCTTTGCTCCGGCTTCGCTCCGGCTCAGCTCCGGAGTAGCTCCGGCTTGGCTCCGGTTAAGCCCAGACGATAGCTTTCCAATTTGTTACCCCTCTGAAGTTGAATCATGTCTTGATTTCCTGTCGTATCCAGTTCTTAGCAATCACAACGTATTGTTAACGATGGCAAAGCCAAGGCCATGTCGTAGAATCAGGGTTTACGGATTACATAAATCACATCAAATCTCATGAATCTGAGGGACCCAAGACTGATAGTATTTTCTTGCATATGTAATCAACAATCATGGTTGTTGTTGATGAAATAAGTCTTTTATAATAATGATTTTTCAATTGTGCTCAAGTGTTCAGTGTTAACGACTCACACAAGTTGTCTGTAGATAGTGTTTTGTGATAGTTCTCTTGTTACAGTTGTCCTTTTCTTACTATTAGGTTATTTTGATTTCTATGACATTACACGTCACAAGCCAAAATGTTTCATATATTATATATATATCTATATAAGCTGCTGGTATTTGTTGTTTGCATATTTTAATACACATTATTATAGGTGTTTGAAGTACATGTAAGAGTTACACAATAGACGAGCCTCCTAAATATTTCCTAGATGGCGAGGAGGTCTTAGAATATTTCACTTACCTTATCTAGTCGATGTATGCAGGGTGTTAAGCTATAAACTTGAACAAAGCAGGGCCCTCTCCTGTTTGCTTTAATGTTCCAAGGTTAATTTCGTTAATTTCATTTAAGTTTGTTTATTGACCTGTCATTTCGATCATCTCACTGTGTCAAAAACAGTTGCAGAAGCTTACGTATTATGCAATGAAAGTTCAAGGGTAACTTTACATCGTATATATGATTAATGTGTTCAAAATCCTATGTCTCTTAAGATTTAAGGTTTATAAGTTCTCACATGTTTATTTGTGCAATACAATCTGAAATCAACGGTAATCTCAAACAATTTTCATATGCATTTATATATGACAGTTATGTATTAAATTCCATGTATTACATTATGTTTCAAAGTTAAGTTAGTCTAGGATTAATTCCCATTTGTTACGCGTTCCCCTGCGAAATATTGTTAACTTGTTTAAACGGGAACAAATTATTTATTTCTTTGAATTTTCTAATGATCACCCTCAGTATGTGAACAAATGTGTGGTCCCCAGTGTCCGCTGTCTTTGCTCATTTGTGTGTAGACTTGAGAGAGTTGTTAAATCTAGGCAAGTTCTGTTTTTGGAAATCTGGATTACAGGTGTGCCTTGTGTTGTTGGTCGCATTGTAAATGCATCTTTGCTGAGAATTGTAGACAAGTCGTTTGATGATGATGACAACAGTCGTTTTATGATGATGATAACAGTCGTTTGATGATGATGACGACTCGCAGTTTGCGGGTGTCGCACGAGTGATTTCGTAGAATGAGTCTCCTCGAATATCGAGTAATGCAGGACAATTAATCTGACATCAAAGTATATTGGATCTTGAATTCTGGTCAGTACAATTTGTTCTAAGAGTACGGACAACCAACCGACCGACTTTGTTGAACTGTGTTCATTGGCCGGTATGGCAAAGAATAACAGCAAGTGGCAAGCATTTATTTTGCAAGTGCAAGCTTAATCAATCTAAAGCCCACCTAATAAGAAAAGCAGAATGCTTTTTGATTCGAAACATTCCAATTGATGTTGAGCCTTCTTATTTCTTTGACCAGTTCAAATCTTGTCCCTACTTTGTTCAAGTTAACTCTGATGTATTTTCTCACACCTTTTGTTTTCGCGTGAATTCCAAACAAATCTTTATAACTTTCACTTTTTTGGAAATACCAAATACGATGTTGACTACTTTACCTGTTACAAACCAGGAATGTGCTTTTGATACCACTGTTGTGTTTTATTAAATAAGTGTTAGTATTTTTGACTCATGTACAGACTTAAAGGAGCTTACTTGTAGTTCAGATTGCAAAGAAAGAAGGGGAAATAACCTTAACTGTTATTATGCAATATATTAATCTTTCCTACTAAGGTCTCTTTGAACCACAGGTTTAATATGAACAGAAAGACTTCTTACTGTTGATTTTAATATTTCTGAGTGAAATGTATGTTCAATTGTAACTTGACAAGTTTTCCGTTTTGTAAAAAAAAATTAAAAATTAAAAAAAAAAGCTGCAAATCTGAATCTTCGTAACCTTGGAAACAAATGTGCTTAACACAGTCCGCCTGCATTCAAGTGCCAATTTGAACAACTAACCGTTTGATTTTATTCCTCTGGTGGGTCCCAGCACGTTTGTTGCTCGGCTCGCGTCGGAAACTTTGTTAGAGAAGAAGCATTCAGATTTGCAGAGCTTCTACAGTTCATGTAGTGCCACTCAACATTGTGATTTACTTGAAAACAATAATATTCACAACTTCTGGGAGATGAGGTTAAATTCTGAGTTAAAAAATGATTTTGTATGCTGTAACATTCTAGTGTGATAGATTTCTTATACGAAGAACAAAAAGCATCTGTGTGGGAGCTTGCTATTGACGTTTTCTAACGAATGTATTATGCATGTCCAAATTAGATTTTTCATGATGGTTTTATTTGTGGATATGATAAACTAACAAAAGTTTTATTTTTATACCATTATTAATACGACTCTAACAAGGCTCTAATACATTGCATGCTTGTCTATTGATATTCATACAAATGTTTGTTAATTGTGGATGTACGGCTTGCCAAAGAAATTATATTTCCAAACTGTTAACTTTGCCCTTTGTAAACTCATTCGTATAGGTCCCATGCACAGTAAGAAATGCACAGTCATTTAGAAAAGTCTAGACTACATATTATATACATGTGTTTCGTCTTGTGCTAGCCATGATAATATAGCATAAATATTATATACATACTATTAATATTATATATATGGTTTTGACAGGATTTTGAAATTTTATCTCCAGTGAGTATTGAAGTGAAAGGTTGCAGAGAATTTTGCAGGCTCCGATGATATACGATTTTTTTGCTACTTGATATTTATATATAAATTATCGATTGAGATAAACTACAGTGACCTGCAAAGTTCTCTCAAACTTGTATTGTATACTTTCTGGTTGTTAGAGAAGTCTGTGCTTGTGGTTCCTATAAATAATTGCTCTCGTCTTCACCCAGCAATGTAGATGCCAATGTCGCTTTTTGTGGAATTGTGATTATATATATCTAACCTATGAATAATGGTGTTATATCTTCCATTATTATATCTGATGTTTGTAATAACTGACTGGATCTCCGACTTTTATTGATCATTTTAAATTTTCAATGTTAATTTATATATATAACCATTCACAGTGATGGAGAGAAAGTGGAATATCATATTTTATCTTTCCCACCAGAAACATTTTTGTTATGAGTGTACTTGGATTTCAATAAAGTATTTTTGTTTGCTTCTAAAATTAAGAAAATTTAAAAAAAA

Protein: 775 aa

>Brat_protein

MSSPTLSTTDTMSLTSNSSEGDIENSLNTKCSLCNETYSIPKVLPCFHTFCQPCLEKIIETPDKLSCPECHQETFLTSAGIAAFPPDFAVNNVLEASVLEGATLSCTGCKSKHLLAVARCFDCPDFLCQDCERAHQYMKHFDGHRVISLGELQNNKEEYKVEKPVMCKKHRQEMLRFFCHTCNIPICKECTLVEHSKGHEYDYLSECARREVYSLQHLAEQAKVKANDLRGSSKSLEHSSNRLQIQYHKAQNEINETYNFYRSMLEERKQEALKELDGAYNAKQSGITNLSSRMQEYIEKLYQGVEFIDRMTKHASSTEVLIFKKMLDTNLQKLTSFMPDTNSVTSSFDLEFVSNYQAIQVGIRNTFGYVRTSSEMQSRQYPQPIARPNGFSAPPRTITPPNPLAANMPNPMVNCNNLFDPTALLLSKNNFPSSGSLTLGSFTDPIISNNLNPYEKWSNGGLDLLHNGDVFSTSTDPVIDLTSKLISANIYPPKSQIKRQKMIYHCKFGEFGVMEGQFTEPSGVAVNAQNDIIVADTNNHRIQIFDKEGRFKFQFGECGKRDGQLLYPNRVAVVKTSGDIVVTERSPTHQVQIYNQYGQFVRKFGANILQHPRGVTVDNKGRIIIVECKVMRVIIFSQMGDVLHKFGCSKHLEFPNGVVVNDKEEIFISDNRAHCVKVFSYQGVFLRQIGGEGITNYPIGVGINPAGEILVADNHNNFNLTIFTQDGQMVSALESKVKHAQCFDVALMDEGSIVLASKDYRLYIYRYMQMPPMLM

Domains

Ring: 30 - 74

BBox (2x): 104 – 149, 162 - 201

BBC: 214 – 334

NHL_BRAT_Like: 498 - 768

Clone

Partial ORF

Primers

Forward: GCTAGGTGCTTCGACTGTCC

Reverse: TTGAATCGACCCTCCTTGTC

Nucleotide# 518 – 1821 = 1304 nucleotides

>Brat_clone

GCTAGGTGCTTCGACTGTCCCGACTTCTTGTGTCAGGACTGCGAGAGGGCCCACCAGTACATGAAGCACTTTGACGGCCACCGAGTCATCTCCTTGGGAGAATTACAGAACAACAAAGAAGAATACAAAGTCGAGAAGCCAGTCATGTGCAAGAAACATCGCCAAGAGATGCTGCGATTCTTCTGCCACACGTGCAACATCCCCATCTGCAAGGAGTGCACCCTAGTTGAACATTCCAAAGGCCACGAATACGACTACCTGTCAGAATGCGCTCGTCGCGAAGTATACTCCCTTCAACACCTGGCCGAACAAGCCAAGGTCAAGGCCAACGATCTCAGAGGGTCATCAAAAAGTCTCGAGCATTCGTCAAACAGACTCCAGATCCAGTACCACAAGGCGCAGAATGAGATCAACGAAACTTACAACTTCTATCGTTCCATGCTGGAAGAACGCAAACAGGAGGCTCTCAAGGAATTGGATGGCGCCTACAATGCCAAGCAGTCCGGCATCACCAACCTGTCAAGCCGCATGCAAGAGTACATCGAGAAACTGTACCAGGGTGTGGAGTTCATTGACCGCATGACCAAGCATGCCAGCAGCACCGAAGTGCTCATCTTTAAGAAGATGCTAGACACCAATCTCCAGAAGCTCACCTCATTCATGCCCGACACCAACAGCGTCACAAGTTCCTTCGACCTTGAGTTTGTCTCAAACTACCAGGCAATCCAGGTTGGCATCCGCAACACATTTGGCTACGTCCGCACCAGTTCAGAAATGCAATCGCGACAATACCCTCAACCCATCGCTCGTCCTAACGGATTCTCCGCTCCCCCTCGCACCATCACGCCCCCCAACCCCCTCGCTGCCAACATGCCTAACCCCATGGTCAACTGCAACAACCTGTTTGACCCCACTGCCTTGCTCCTCTCCAAGAACAACTTCCCCTCATCTGGGAGCCTGACCCTCGGCAGCTTCACTGACCCCATCATCTCTAACAACCTGAACCCTTACGAGAAGTGGTCCAACGGCGGCCTTGATTTGCTCCACAATGGAGATGTGTTCAGCACCAGCACTGATCCAGTGATCGACCTCACGTCCAAGCTGATCAGCGCCAACATCTACCCACCCAAGTCCCAGATCAAGCGCCAGAAGATGATCTATCACTGCAAGTTTGGAGAGTTCGGAGTGATGGAGGGCCAGTTCACAGAGCCCAGTGGAGTTGCAGTCAATGCCCAGAATGACATTATTGTAGCAGACACCAACAACCATCGTATTCAGATCTTCGACAAGGAGGGTCGATTCAA

Cloning info

PCR rxn on 11/25/15 using GoTaq polymerase mix

| Template | Initial denature | Denature | Anneal | Extension | #cycles | Final extension |
| --- | --- | --- | --- | --- | --- | --- |
| 2 hr cDNA | 4min @ 94C | 30sec @ 94C | 30sec @ 55C | 2min 15 sec @ 72C | 35 | 10min @ 72C |

Purified PCR Product with Thermo Scientific kit on 11/25/15, Second PCR from purified template on 12/1/15

Ligated PCR product into Pgem T Easy vector and transformed into competent cells on 12/2/15

Picked colonies for overnight cultures on 12/7/15

Isolated plasmids using Promega miniprep kit on 12/8/15

Confirmed clone Brat by sequencing on 1/15/16

Brat is sense to T7. RNA probe generated using SP6 on 1/20/16

**Pins**

Gene model: 4566 nucleotides

ORF: 151 – 2121 = 1971 nucleotides

>Pins_full

CGGGAAGTCAACAGAAATAAACCGTCAAAAAGAAAATCTGCAATTTGGAATGGAAATGGACCTATATTGGCGACGTTAATGCATCAAAACGCTGTTGGTGAAGAATAATAAGAGTGGCTGATCTGTCCGACGAAATTACTGGCTCGCAAAATGACGATGGAAGGAACGTGCATGGAACTGGCGTTGGAAGGAGAACGGTTATGCAAAGCGGGAGATTGCCGAAGCGGAGTGCAGTTCTTCGAAGCAGCCATCCAGGTCGGTACGGATGATTTAAAGACCTTGAGCGCCATCTACAGTCAATTAGGCAATGCATACTTCTACCTTCAAGGCTACGGAAAAGCTCTCGAGTACCACAAGCATGATCTCAATCTCGCAAGAGCATTAAACGACAAACTAGGGGAGGCCAAAGCGAGTGGAAACCTCGGCAATACTCTAAAAGTACTCGGCAAATTTGACGAGGCTGTAGCTTGCTGTCAGCGCCATCTGGACATATCACGGGAACTTATCGACCGGATTGGGGAAGCAAGAGCTTTATACAACCTCGGGAATGTCTACCATGCAAAAGGGAAACACGCCAGCCGTGCAGCCCACCAGGACCCTGGGGAATTCCCCGCCGAAGTCAGGGAGTCATTACAAAAGGCCGTTGAATATTACGAAGCTAATCTTCATATTGTCAAAGAACTCGGGGACAGGGCGGCCCAAGGACGAGCCTGTGGAAACCTGGGGAACACTCATTATTTATTGGGGAATTTTAGCCAAGCTATCAAGTTCCATGAGGAGCGACTTGCCATAGCAAAAGAATTTGGCGACAAATCTGCAGAACGAAGGGCTTTCAGTAACCTAGGCAACGCACATATCTTCCTTGGAGAGTTTGAGGTCGCAGCAGAGCAATATAAACGAACACTGCAAATTGCAAAAGAGCTCGGAGACAAGGCCTTGGAAGCTCAGGCCTGCTACAGTCTTGGAAATACATTTACATTATTAAGAGACTATGAACAAGCTATAGAGTACCACTTACGCCACCTGCAGATAGCACAAGAACTAAATGATCGCGTTGGAGAAGGACGGGCATGTTGGAGTCTTGGAAATGCTCACACTGCTCTCGGTAACCACGAGAAGGCGCTGCAGTATGCAAACCGCCACCTGGAGATATCTCAGGAGATTGGAGATGAAGGAGGCCAGTTGACGGCGCAAATGAACCTGACAGATTTACGAAAACTTCTAGGAATTACTGACAGAACTAATGACCAGGGAGACAAAACTGTAAATAGTAGCAATTCATCAACGAAAACTTCTTCAGACAATGGAGACGGCTCAGACGACAGATTGAGGGCAAGAAGGCCGAGTATGGAACATATGGAGTTAATGAAATTGACACCTGATGCCAAGGCTGAAGGCAAAGGAGCTAGGCCCAAAGCAAACAAAGGCCACAAATTGGAGAAGTCAGCCAGTGTGTCAAATGACTTCTACACATCACGAGAACCTAGGGGAATCAACGCCAACAGACCCAAGTCAGCACGACCCTTGGGAGAAAAGAAGAAATCGGATGAAATGGAGGAGGACGTGTTCTTCGATTTGCTTCACAAATACCAGAGCAGGAGAATTGACGACCAACGATGCTCCTTCAAAATACCTGAGAGATCCAACAGCTTCGAAAATGCTCAACCCATGGCGCCAAATTCCCAACAAGCAGAGGATTTCCTGGACATGGTGGCCGGCATCCAGGGGTCCCGAATGAACGACCAGAGGGCATCTCTTCCCAGTTTCCCAGGCCTCAATAGCAGCCAGGAGGTGTTGAATGAACTGCTTGCAAGCAAAAAGGACACCACAGCCCTTGATGATCAGTTCTTCGAAATGCTCATGCGATGTCAGGGATCTCGAATAGAAGACCAACGAAGTGAATTACCTGCTCGTTCCGCTCCCACCGTTCCCGACGAGGACTTCTTCAACCTTATCCAGAGAATCCAATCGAACCGTCTCGACGAACAAAGAACCACTCTGCCGGGGGGAAAGGTCGGAGCTAATCTAGGCGGAAATCTAGCAGCAAATGGAGGGGGCGAATTCACCGGACGCAGTTCTTCTAAAAAAGGCAAAAAAAGCGGAAAAGAAAATAAGAAATAGGAACGAAACAAATTATAGAAAACAAAATATAAAAGTTTGTAAGATAGATATGTGATAATATATATATTATATATTATATGATGATATATGATGGCTAATGATAGAAACTGAGGTGTACTTTTCAACGCCAATTGACTCTAAATTGGCTTTAGTCTATTAATAGGGTCATAGCATGGTTGGACATGTAAGCCAAAGTTTGAAATTACTTTTAGAACAAATTTCAAACAAAATTCATCCTGTCATTGGGACTAGAATATAACTGATCCCTGAGATGCAAGAACTGGCCTTAGAGGCCTGCACTACATTACCGTGTCCATACCTGCCTATTATAATGAATCCAACCACCTAAAAATGTACATACAAGCATACATATAAATATACATGATATAAATGAGACATCGCATAATACATAATATGATGATGGCACAACCATGAGATTCGCGTAGCAGTTATACTAATTTGCATTGAAGGAAAAGTATGATTGTGGCACCAGCACATTGTGAACAAAGTATTTTTTTTTAAGAAATGGTGGTGGCGCTAGTAGTATTTTAGTGTTTACCACTAGGCGGACTCAAAGCAATCATTGCCATCTACTGGGAATCGGCGTCATTACGTTCACAGAGCCGACCGGTCGGCTGGGAAGGAAAGCAGGAAAGGTTTTTTGTTTATTTTAAAATGCATCAGCTTCTGCTTCGATTTATTATATGGATTACTGCACTGTGACTCAGCGTGACTAGGAAAGTAAAGGACAACTACATGCACTTTAGTCTCAGGCTGGAATCTTGATCTGATTGACTTCACTGTGATGATGATCACGAAGATTGGCGATTTTGCCATATTCTGACATTCGTGGTGGCGCTGCAGTCTCACCTTTGGATTTTCGTTTTGGATTATCGTTTTTGGTGTGTAATGATGCACTGAGCAATCAAAATAATGCATGGAGAGGATACCATAAGACAATATTGGAATAATCGGACATTTTTGGAAAGCAAAACAGTGTCACCGTGACAATGTCTACAGCAGTGATCACTGGCCTATAGACTACACAGGGTGTCGTCTGCAGCCATTAGAGGTGCCCACAGGCGCCACCTGGTGACGTTTGTCCCCATACACATGCTACAGGTGAGCTACCAATCACCAGGTATAAACAAATATGGCGACCAGAAGTTCAGGATGACATGAGTTCAGAGTTTTATGCATATTTCAGAATATGGGGACTTCATCAACAGCAGTCCCTGGTGGAGGGGGTTGCAGATACAAGATGTTTGTAGTGATGAACTACATTCAGATCATCACAGGCCTCAGTCTATTGGTTTATGGTTATGGCAATTACAATTCTGGAGAAGGAGAAAAGGAGAATATCCATAAAGGCAACCCTACCCCCCAGGATACACAGCTGCCCTTCAGTACTATCACTATATTAGCAGGCTTTTTGTGTTCCGGGTGCGGATTACTTGGCAGAGCTATCCCAAACACAAGAGACTCAATAGCGAGATACGGAATGTCAGCAGGGTTCATCATTGTCTGTCTGCTCAACTGTGTGTGTTTTGCCCCTCAATTGGTGGCCATGGGCCTGGTCAACTACTACCAGGGATCAACAGCAGGCCCTAATGCACTCAACTTCAAAGTATTGATAGCCACAGGAGTGGTGGAGCTTCAGGCTGGAATTTGTTCCATGTTTCTTGTCTGTGTGTTCCTCAGTCATCTAAAACCTGCGAAAACACAAATAAAGACGCTGTGATATCAAATGTCTCTGAGTGACCATTCCACAAGAAAACTGACTGTGATTACTTGTATATATTGTTTAGAGGCAAACTACCATTACCTTCTTCATTATTTTCATGATATAATATAAAAATACCTGATTTCATTTGTACCTAATGTGTATAGTGTTTCATCATTAAATCTTGCCAAATTAACTGTTGTTATTTTGATTATACATGTATCAGTTATCTAAAATTCACCTCTGGACGCTTGTTTGGTCATTTCATGCAAAATCCAGGCTAATGAAAGTGAAGCACTGTGTAATATTGACAACTAAGCAGAACCATAAGAAACGGTTTGTAAGCTCATTATCAGCTGAGATCAAACCTGGAAGTATGCAATCAACACCACACTGTAATCCATGATACCCACCGAAAAGTTTCACACAACTGAGTAGGATACCAATTCCGAAAATTGAGAGTTTCATTTTTCAATATTTCAGTCTTTATTAGACCATCTTCAGGAAAGCAATGATTAGGAGTCATACATGTTGAAATGTTAGATACAGTAAAACAATGAAAACAGATTACAATGACAAACAACATTAAACAGTTACAATCATACAGAGTGTGAAATACAGTTGAAATCAATGGAACAATACTAGTTGTGGAACATTAGCTCTAGAACTATTGAGTCCGAGCATGTAAACATAACAAGTAAATATGATAAGACCCTGGAGGG

Protein: 656 aa

>Pins_protein

MTMEGTCMELALEGERLCKAGDCRSGVQFFEAAIQVGTDDLKTLSAIYSQLGNAYFYLQGYGKALEYHKHDLNLARALNDKLGEAKASGNLGNTLKVLGKFDEAVACCQRHLDISRELIDRIGEARALYNLGNVYHAKGKHASRAAHQDPGEFPAEVRESLQKAVEYYEANLHIVKELGDRAAQGRACGNLGNTHYLLGNFSQAIKFHEERLAIAKEFGDKSAERRAFSNLGNAHIFLGEFEVAAEQYKRTLQIAKELGDKALEAQACYSLGNTFTLLRDYEQAIEYHLRHLQIAQELNDRVGEGRACWSLGNAHTALGNHEKALQYANRHLEISQEIGDEGGQLTAQMNLTDLRKLLGITDRTNDQGDKTVNSSNSSTKTSSDNGDGSDDRLRARRPSMEHMELMKLTPDAKAEGKGARPKANKGHKLEKSASVSNDFYTSREPRGINANRPKSARPLGEKKKSDEMEEDVFFDLLHKYQSRRIDDQRCSFKIPERSNSFENAQPMAPNSQQAEDFLDMVAGIQGSRMNDQRASLPSFPGLNSSQEVLNELLASKKDTTALDDQFFEMLMRCQGSRIEDQRSELPARSAPTVPDEDFFNLIQRIQSNRLDEQRTTLPGGKVGANLGGNLAANGGGEFTGRSSSKKGKKSGKENKK

Domains

TPR_12: 85 – 170

TPR_12: 190 – 257

TPR_12: 269 – 337

GoLoco: 471 – 492

GoLoco: 515 – 536

GoLoco: 564 – 585

GoLoco: 596 – 617

Clone

Partial ORF

Primers

Forward: CTCGGGAATGTCTACCATGC

Reverse: TTCCATACTCGGCCTTCTTG

Nucleotide# 541 – 1353 = 813 nucleotides

>Pins_clone

CTCGGGAATGTCTACCATGCAAAAGGGAAACACGCCAGCCGTGCAGCCCACCAGGACCCTGGGGAATTCCCCGCCGAAGTCAGGGAGTCATTACAAAAGGCCGTTGAATATTACGAAGCTAATCTTCATATTGTCAAAGAACTCGGGGACAGGGCGGCCCAAGGACGAGCCTGTGGAAACCTGGGGAACACTCATTATTTATTGGGGAATTTTAGCCAAGCTATCAAGTTCCATGAGGAGCGACTTGCCATAGCAAAAGAATTTGGCGACAAATCTGCAGAACGAAGGGCTTTCAGTAACCTAGGCAACGCACATATCTTCCTTGGAGAGTTTGAGGTCGCAGCAGAGCAATATAAACGAACACTGCAAATTGCAAAAGAGCTCGGAGACAAGGCCTTGGAAGCTCAGGCCTGCTACAGTCTTGGAAATACATTTACATTATTAAGAGACTATGAACAAGCTATAGAGTACCACTTACGCCACCTGCAGATAGCACAAGAACTAAATGATCGCGTTGGAGAAGGACGGGCATGTTGGAGTCTTGGAAATGCTCACACTGCTCTCGGTAACCACGAGAAGGCGCTGCAGTATGCAAACCGCCACCTGGAGATATCTCAGGAGATTGGAGATGAAGGAGGCCAGTTGACGGCGCAAATGAACCTGACAGATTTACGAAAACTTCTAGGAATTACTGACAGAACTAATGACCAGGGAGACAAAACTGTAAATAGTAGCAATTCATCAACGAAAACTTCTTCAGACAATGGAGACGGCTCAGACGACAGATTGAGGGCAAGAAGGCCGAGTATGGAA

Cloning info

PCR rxn on 07/28/16 using GoTaq polymerase mix

| Template | Initial denature | Denature | Anneal | Extension | #cycles | Final extension |
| --- | --- | --- | --- | --- | --- | --- |
| 2 hr cDNA | 4min @ 94C | 30sec @ 94C | 30sec @ 55C | 2min 15 sec @ 72C | 35 | 10min @ 72C |

**Khc-73**

Gene model: 10895 nucleotides

ORF: 291 – 6317 = 6027 nucleotides

>Khc-73_full

GTCCGAATGTCCGTCAATATCCTCTCATGCAGTTTGTGATAAACTTTCTGAACTGACGATGGCTAGTTTATGACATTTTCACCAGAGGGACTGATTTGCACGTGGAGGGGTGCATTGTGGGACAGGTGCCAAAACATGGCCTGCGTTTTGCGAAGTGCTGTGATGTGTTGACATTGGAAAATTGGGCAAAATGGATCTACCAAAAGGAATAGCCTGCAAAATAGGATACATATAGCGAAAGGAGAGTTTCTTCATTGGATTTACCAAAGGGGGAGTGTTAGAAATCGAAAATGGCGTCCTCAGACAAGGTTAAAGTGGCCCTCAGGGTTCGGCCGCTGAACCGCAGAGAAATCGAGATGGGTACAAAGTGTGTGGTGGATATGGACGATACACAGACTGTTTTGTACCACCCCACCACTGGTGTGAAGGAAACACTAAAAATTATTCGCCAAGACAAAGATGCTGCCCCTAACAAAAAGTCGCCGAAGGTTTTTGCCTTTGACCACTGTTTCTGGTCAATCGACAAGAACGTTCCAAAATTTGCAAGTCAAGAAAAAGTGTTCGCATGTCTGGGGCAAGATATCTTAGAACGAGCCTTCGAAGGTTACAATGCCTGTATATTCGCATATGGCCAGACAGGCTCGGGCAAATCATACACGATGATGGGCACGAACACCGAAAAGGGAATAATCCCCCGTCTCTGCGATAAACTATTTGACTCAATCGCCCATGCAACCAACAGCAGCAACAGTTTCAAAGTGGAGGTCTCATACATGGAGATATACAACGAGAAAGTGCGAGATCTCCTCGACCCCAAGGGAGGCAAACAGCACCTAAAAGTGAGAGAGCACAGTATTTTAGGACCGTATGTGGATGGATTGTCAACTCTGGCTGTGTCATCCTTTGAGGACATCGATGGCCTCATGCTGGAAGGAAATAAATCTCGTACAGTCGCAGCCACAAACATGAACAGCGAAAGCAGTCGGTCGCACGCTGTGTTCACCATCATCCTCACACAGACCCTGACAGATCTGGCTTCTGGGGTTTCGGGAGAGAAAGTATCCAAAATGAGTTTGGTTGATTTGGCGGGAAGCGAGCGAGCCCAGAAGACGGGAGCCGTGGGGGAAAGGTTGAAGGAAGGAAGTAACATTAATAAGTCCCTCACAACTCTGGGTCTTGTAATATCAGCCCTTGCAGACTCACCGGCAAAAGGAAAGATCAAATTTGTGCCCTACAGGGACTCCGTATTAACATGGCTGCTAAAGGACAACTTAGGAGGAAACAGCAAAACAGTCATGGTGGCCACGCTGAGTCCTTCTGCAGACAACTACGAGGAAACGCTTTCGACATTACGATACGCAGACAGGGCCAAACGTATCGTCAATCACGCTGTGGTCAATGAAGATCCGAACGCACGTATCATCAGAGAACTGCGGGAGGAGGTGGAGATGTTACGGAAGATGCTTAGTGCAGCTCAGATGAACAGGTCTCCGGACCTCCAGGACAGATTGGAGGAGTCGGAAAAGTTAATGAAGACCATGAGCAAGACGTGGGAGGAGAAATTGCGGGAGACTGAGGAGATCCACCAGGAAAGACATGCTGCCCTGGAGAAGATGGGCATTTCAGTGCAGACTTCTGGGATCAAAGTCGAGAAAAACAAGTATTACTTGGTCAACCTGAACGCTGATCCTTCCTTGAACGAGCTGCTCGTCTACTATTTGAAGGACCACACCCTTATAGGTCGCCCAGATGCCCCTCGGGAGCAAGACATCCAGCTCTCAGGGTTGGGTATCATGAGCGAACATTGCATCATAGACCTGGTGGAAACTGACGTCTATCTCACACCACTAGAGGGCGCCAGAACTTGTCTCAATGGCCGAACGCTGACAGAAAGAGTCAAGGTCAAAAATGGCGACCGTATCGTTTGGGGAAACAACCATTTCTTCAGAATCAACTGTCCCAAAGCTAACAGTCCCTCAGCAGAAACGCCCGAGCAGAAGATTGATTACGACTTCGCCCAGACGGAGTTGATGATGCAAGAATGTGGGGACGACCCAATACAAGAAGCAATCGAGGCCATCGAGAAACAGCATGTCGAAGATAAAGCAGAGGCCCTCGAGCGCCAGAGGCAGATGTACGAGCGTCAAATGCAGATGTTACGGAATCAGTTGATGTCACCAAGCACGCCCAGTCAACCCTATCCTCCTTTCTTCGTCGATCCATTCGGTAAAGGGACCCCTGGCGCCACCACACCACACAGCAGCATGCACCCCAAATACCAAATATGGGCGCAGGAGAGTCTGGACGATTATAGCCGAAAATGGATCGGTACCCAAGAAGTGGGATGGGAAAAACTTTTCAAGTCGAGTTTGGCCAAGTTGAAAGAAGAAGTTGTGAAAGCAAACTCTCTAGTCAGAGAGGCAAATTTCCTCGCAGAGGAGATGGGAAAAGGAACGGAATTTTCCGTCACTCTGCAGATTCCCGCGGCTAACCTTAGTCCAAACAGAAAGCGAGGAGCTTTCGTCAGCGAACCAGCAATTCTCGTCAAGAGGAAAGCCCGCAATAGCCAGATTTGGGCGATGGAGAAGTTCGAGAATAAGATCATCGACATGAGAGATTTATACGAAGACAGAAAAGAAAAGGGGCTGCCCATGAAGGTGGATGATGATGAGGATGATTTGCCCCTTGTTATTCCTAGTGATGGAATGGACCAAGGCCCTCCCAGCAAAGGAGATCCATTTTACGAATCGCAAGAAAACCACAACTTGATAGGAGTGGCCAACGTCTTCCTTGAGGCTCTCTTTTATGACGTCAAACTCGACTACCAAGTCCCCATCATCAGCCAGCAGGGGGAGGTGGCAGGACGTCTGCACATAGAACTCTGCCGAGTGTCCAGTTCCCTTCCTGACAACAATTCGGCCGATTTTGACAGCAACGACCACATCGACATGATCAACAATGAGGATTCTGTTGTTGTGGGCTCAAATATTGTTTGTAGGGTGAGCATCAAAGAAGCGAAGGGCCTCCCTCCAGCCCTCTCCAACTTCGTCTTCTGCCAGTATTCCTTTTGGGGTCACCCTGAGCCGATCGTAGTCCCCCCTCAAGTCGATCCAGATGTCCACAAACACAAGAACGACGGCGTCTCCTTCCGATTCGATCACAAAAAAGAATTCAAAATCAACGTAACAGAGGAGTTCATAGAGCACTGTACAGAGGGCGCTCTCTCCATGGAGGTGTGGGGTCACCGCAGCCCGGGATTCGTACCCACGATGCCAGGATGGGAGATCGACCACCAAGCCAAATCACGCTCTATTATGGACAGATGGAGTGAACTGACGAGAAAGATAGAAATAAAAACGGAGATTCATGAATTGAACGACCAGGGGGAGTACGTTGCCGTGGAGGTCATTCCAAAACCAGACGTTCTAACCGGAGGCGTCTTCCAACTGAGACAGGGTCACTCTCGCAGAATCCAGGTGGAGGTTAAACCAGTCCAGAATTCCGGGACTCTTCCGATAATCTGCGAATCCATCACTCACATCTCCATTGGAGCGGTTTGCGCTCGATCCAAAGTCCAGAAAGGACTAGACTCATACCAAGAAGAAGATTTGAGCCGTCTCCGAGAAAAATGGTCCATGGCTCTCGACAAGAGAAGAGAATATTTAGACGAGCAGTTACATAAGATCATGAACAAGGAAGCCAAAACGGACACGGACATAGAGAGAGAAAAGAGTCTCGTTGACCAGTGGGTTTGCTTAACGGAGGAGCGGAACGCTGTTTTAGTTCCGGCTCCTGGTTCCGGAATCCCTGGAGCTCCGGCAGACTGGAATCCCCCTCCGGAAATGGAACAGCACGTCCCAGTTATATTCCTTGATCTCAATGCTGATGACATGAGCACCCCTGGTGTCAAGGAGGGACTACAAGCGGCGGGAATTAATTCCATCCTTCCAAAAGAACACGGCGCTAAATTCTACTTGCTTCCTTTCGTCAATAATGATACCTGTGCTGTCGTCTCCTGGGACTCCTCAATCCACGACTCTGTTCATCTGAACCGACTGACTCCGACGAACGAACGAGTCTATCTGATCCTAAAAGTGATCGTCCGATTGAGTCATCCTGCGAGTATGGAGCTCGTCTTGAGGAAACGACTTTGTATCAACGTCTACAAGAGACAGAGCATTACCGAGAGGTTGAAGAAAAGGATCGGGAAATCTGTTCTGCAGAGTGTAACGGGGACGGAATATTTCCAGGATACTCTGTGCATCTCGGCCGTCACTTACGAGATCGTCTCCAACATTCCAAAGGCGTCAGAAGACTTAGAAGACCGTGAGAGTTTAGCGCAGATGGCCGCCTCCAATAACGAAGCGAATGCAGTGGACGGAGAGACTTACATCGAGAAATACATCAAAGGGGTCTCTGCTGTGGAGAGCATACTGACCCTCGACAGGCTTAGACAGGAGGTCGCAATAAAAGAACTGCTGGCAAGCAGAGGTCGACCTTTGAGAAAGAGCACCAGCGTTCCCAACATCCATGGGGCCGTTAACATGTCTCCACTGGGTGGCCTCGACAACAAGATGCGGTCAGACAGCATTCAAGACCTCTCTTACAACGCGGAACGTTTTGGCTCCATGCCGAAACACATTTCCCTAGACTCACTTCGCAGGGTCGGTAATGATGAAACGCCCCCCAGGGACAGCGTTTTGCTGCCTAATAAGCCAGCTGGCCCCTTCAATCTAGCCTCTCCAAAAGCCGGAGCGAGCCCTCAACCAACAAAACTAGTCAAACCAATGAGGACTTTGATGGAGGAACAGAGCATACGGGAGAGCAAGCCACTGCTCAGGCAGGACGATGATTCTGACGAAGATGAGTTTGCGAAATTGAAAGAAGCTCCTCCAAAAGAGGGCGACACCAGTCGTACAGGAGAGGACAGCGTAGAATCGGACGAATTCCAAGATTTTGAGTCCTACCAAGGAGGACAGGAGAACGCAGAATTGCCTCATTCTAACACTAACGATAGTCTTGTTGACACACATGCGAAGAGCTACCCCTCCAGTGCGTCATCTAGCGGCTATGGCTCCCAAGCGCAGTCTTCGCAAACACTTTCATCCGAAGACAACAACAGCGTGAAGTCGTTAGAGGAGGGAGAGAGTAATGCAGGCGGGGACCACCAGAGGTCAGCACAGGGCAGGACGACCTCCGAAAGTCAAGGAGAAGAGGAAGGAGGACAGAGACAGAGGTCTGACAGCTTGCTCGACACAGAGATCCCCAGTGGACTGGGAACCCCAACATTACTCAGCCCAGAAGTCATCCATAATAACAAGGAGACAGACGGACAGACGGACACCAGTCAGGCAGACAAAGATACACAGAGGAATGAAGCCAATACTAATACTGCTGAACACAAAGCTGATACAGGTTCCAAGAAACTGAATGTGTCATCCGCAGACCCCTACAGCGAAGACATGTTGCAAGAGCTGGAGCAATTGGAAGGATGCGACGAGTCATTCAACATCTCCAAATCCATGAATAACACGATGAACACGTCGCTAAATACTTCTATTAATACATCAATGAGCACCTCACAGAATTTGGAGGATCACGAGGGGTCAGATGATACCATCAAGTCAGAGGTCAAACTTCGAAAGGGAGGCACAATGGAAGTCTCCCGTCCTGCAAACCTCAAGAAACTGCGGACGGAAACCAGGACGACGAAAGCGTCTCCGTCTCCGGTTAAACCGGCTCCTCTCAAAGCGACTTACCGACCCATGTCCATGCCGATTGATAACAGCCTCCCTGTTCATGAGGATGAGCCTCAGTCCGCTGATGAGAATAACTCTGATAACGAAGATGCAATGAGCATGTACTCGTTCGGCAGTCGAGCTGATCTGTCCCGCGTGGGAGACTCACCCGTCCCCACGTGGGTGCAAGTGGGAGAGCCAGTGATAGTGCTGTCCTCTAGTGGCGGGCAGAAGAACGGAACGGTGCAGTTCATCGGACCAACGGAGTTCGCTACGGGAACGTGGATTGGAGTGGAACTGGATCTGGCTGATGGAAAGAATGATGGCTCAGTGAAAGGAGTACGATACTTCAAATGCAGAGCTCGGCACGGGATATTCGTACGACACGAGAAACTGATGATGGATAAGAAGCGTCGATCGAGTAGCGGCAGTACGGGCAGTCTCAAGATGAAAGCAGTAGCAGGCATGACAACGTCACTTCGTAGGTCTACGGGAAATCTGTCAGCGAGTAGTCCGGGGTCGAAAGATTTGCCCGGGTTCATGAGAGCGACCGCTGCCACTTCGCAGAAGTTCAAGTGAGCTCGAATGGGATGATTTATTGATGCTAAAAATCTTGCGAATGGAATTTTCGAAAGGATTAAAAAGGATTCTTTGAACCTGCAATCTATTTGCTGCAAGTACAAGAGATTTTGTGAAGAAACTATGAAGATATGTGAAGAAGGAAAGTGATTATGTGAAGAAGAAAACAGATGTGGTTTCCCAATATTTCGTGAAGAAAATTGTGATTTCAGAAATTTTGCAAAAGTTTAGGGTCAATTTCGCAACGACTTGCAAAGTTCAAAGTCATTTTTAGGAAGATTTCACAATTTGAACAAAGACTATAATTTATTATAAGATAAGAATCGGTTTATCCATGGATTATTTAATCATAAGATTAGTGTCCTGGGATTTATCCAACATATTTATCAAAATATGGACTAAGCCGGAAATGGCCGCAATATAATATATAAATATTATACAGTCTGACACATTTTAATCTTTCAAAGGGCGTGGTACTGTTGAATAAAATTTCTACTACATAATCACAGTTATGATGATGATATGTCATAAATATGATGATAAATATAATGATGGTTAGGGGGTGGTTTGTAAAAACATGATACGATAAATTGTTCAAAAACCATTTAATGATAATTAATGTCTCTACATCGTTGCCAATTTGTGTCTGTGTTATATTGTCCTGTACATATTAATGTATTATGTAATATTATGTACCATTATCCTAACAATATTTGTGGTATGTTTGATTTTACTCTTAATTGTATTAATGATTATTCATAGTTATTACACAGGTCGTATTTATTGGTGAATTGGTGATGATTTTTTAATCTACAAATCTGGTCGTATGTGAATATTCTTATAACTAATGACTAACTCGAATGATCAATAGATGATATGAACGATATTATGCTACGTGATGATGTTTCACATCTATAAAATTCGAAATGATTTAATAACTTAAATCCACTCAAAATCCTCATTTTATTCTTACAACAATCTGCATGAATTTGAGAATAACCCCGAGATTACGTTCAAATTTACTTTACTGCAGAACTGAGCGGTTGATAAAATTTTACAAAATTTTGTCACCAGGTGCTCGTACGTGAATGCTCACTTATATCAATCAAAGAAGTTCGACTTAAATAAGGAAATATCTTCAATCTACCAACTATGATTAATAAAAGGATTATGGAACGGATAAACCAATACTCTCCCAATAGTTAATATATAAATGTTTTAATTAATTGCAATTAGATTTTAACACTTTAAAGAAATTTAGTTGTCAAGATTTTTTATAGTGATTGCAATTGGTCAAAAAAAATATCTTGATTGATCTTAACTTAAACATTATGAGTAATGATTTCTATCTCAAACGATGTTATTGTACATATTAGCAGTAGAGTTGAAGATTAAAATGCGGAGAGAGTTCGAGAGACCTGGCATTGTGTTGTTGATAAATGGGAGAATTTGTAAATATTTGATTTTAATGAAAGCGTTTTAACTTGTGAAGTTAAGCGCTCAGTATTATCACTGACCTTAGTAGGACTCGTTTAAATAGGGTAGTGTGTTTATATCGCTATATTACTGTCGATCATCCGATATCTGTCAATAACTCTTGTTGCTGCATACAATTGCAGTCCTAATTGCAGTTATAAAGTTTAATGCAATGTGAGTAATTTCGAATAAACAACCAACAACCAATAGTTGTATATAGTATGATTATCAGAATCATAGTCATTAGGAAAAAATCTTTGAACCAACTAGATAACGTCTATAATTGCAGTCACGGAAAATCTAAAGTATTTCGCCTTTGCTATATATCTGATTGCAATTAGCAAATTAATTGGTATTACAATTAAAACCGATAGATTCTATTTCTGTAATGAATACTTTAAAATGGTACATTGTGACTATTTAATAGATACATGTATAATGTAACATTATGTATTATATTGTAAGTAGAAATTATGCACTCAAGTCTAGGTACGTGTATCATAAATTCATCTATCAAATTCAAACTGTCTATGAAACCCATAAATTCAAGTGTACAAATTTGCATTTACCACAAATTCAAGTGTACAAATTTTCATGTACCACCAATTCAAAATGTACCATAAATACAGAAGTCCATCATTAAATTGAAATGCACGGGGATATAAATAATCGGCACATAACTTGCAGACTGTCAATTGATTTTACTTTAGAGCATGTCATTAAGTACACAGTTTTTAATTGCAATTAAATGTTATTGAAATTGTACATACCATTATAGTAACTCGAGAAGTCAGCGCCGATCGGAACTTATTACTCCTAAAAATCTGTCATTTCACGGTTTATCTAACGCCACGGCTCTAGATGTGGAATATTGAAAGCCGTCTTGGTCTATAAAAAGGCTCTTTCAAAATTTCATGAATGATTTTAAGTATTTCAAATGATTTGAAAAAAAAAAATGTCTTCAATGATTTTATTAACAGAATCAGTTCTCTGCGATATTATATAATAAATATATAATATTGATGAATATTGAAGATCAGATTTTAAATATTGTGAAAATCTCCCAATCTCACAGATTTATGAATCATGGTGTAAATATTAATATTGAATATTATATCGAATATTAGTGAATTGTGTGTTCTTTGAATATTTGTGAAGATTGTGTGATGAATTAACTTGCGTGATATCCTCAAATGACATGACTTTTTTCAATATAATATCAATATAACAATTACAACATCAATATAATTTTCAATATTTGAAGGTAATGAGACAAAAAGATGATTAATCTGAAAAGTCTTTGATACAATTGATTTCAAAATTATTTGAATATGATTTCAATATAATATCAATGTATTATCAATTTAATTTCAATGCTATTTCAATACATTCAATATTTGAAGGTATCAAGAAAAGTTTAGTTTATTTGAAATCTGAACAAAAGTCCTAGAAATTTATAATAGAGAGATTGTCAATACAATTAACACCTAAAAACTGAAGATTTATTATGTAATTTTAACAACTAGCATATCCCTGTCCGTATCATATTATATGTAGAATACAACCTCTATATTATAAATATACATTGTTACATTGACATACAATAGGGTGGACTGATCAGCCAAATATCTATGTATTGAATATTGAATTTGATATATTTAATATTTGAATTAACATTGAAAGGAGATTGTATATTGATCAGATATTGAATATATGAATTGGATATTTAATTTTTGAATCAAATATACTGAATATACCAATTAATACTGAGCATATTCATTAAATATTTATTTAAAAAACCATTCCATGTTAAATCTGTCGAGTCATACCCTTGTAACGAGACATACTACTGAGTCAAGACTATGGAATCATGAAGGCTATCGAGTCATACTGCATAACTAATGAGTCTCGCATATATGGCATTTTTATGAGAATAATTATTCTACCCCGTTGTCATGTTTTAACCATTTGAGGTGTGTGATTTCTAGGAAATGAATTTCTGGAAATAAAATTTCTAGGGAGTCAAATTTCTTGCTTGGAATGGGAAAGGACTAATGATATGCTAATTTATGATAATGATATGCTAATGTATATGATTATGTAAATGTGTACTCGTGTATTTTCAACACTGACATATTTGTAAGATAAATATTGATATATATGATTATGTAGATGGCAAGCTGTAATATAGTGTTTCTAGACTCATCATGTGGTGCCCCCTATGGAGTAGGTATTCAAGAGGTCAGAGTCGTCATTTATACTTAGGGTGTAAAAGGGGTTTAACGCAACAGTTGACAGTAATAACACTCGACAGACGGCCTTATAAAAACTTAGGTCAGCATTTATCGCTGGGTGGAATCATTGCTGGGTGGTATTGCAATATACTACAGGTGGCACTCCAGTCGGCCTAAGTATCAGTGTCCTCCACAAGAGGTCAGCACTGAGTAAGCTCTGCGGCTGGCGGGAAGTTTAAATTAGATTTTGATGGAAAAAGATTGAGTGAAGATAGATAATGACCGACCATAAAGATAAAATCTAATGAATGTAGATTGATGTAGATTGATTATTATAGAACGCCCCCCTCCCCAAGTATATCTGACCACTCGCACCTCACTTCTCATTCAATATTTGTGCAAGTGACTATTTATATATATAACTCTTATCCTATTATATTAATGCCTGTCTCTGTCTATCCGTCTATCTTTGTTCCTTTCCGTCTTGTAGTGGTCCGTGTTTCTGTCTGTTTTTCCTGATGTATAAAAATTGTGCAAAGCGGCAAGACAAGCCGATGCTGGGTTTAATAATCGATGAATAATATGGTCAGAAAATGATGTTAGGGTAATTGCCATACAATTTATCATTATCATAAAAAAACACGATTATTTGATTCCTCCCAAAGGTTATGATTTGTTTATGTGCCATTCTAGATTATTTTTTATATTTTGTTTAATTGTTGTTTTCTGTTCATTTTGGTTGTAATGATTAATTACAAAATGAAGGAATAATGATTACAAATTTTGAAGAACTGATCATGTGCTTCATTTTAACTTCGAGCAGTCTGACAAATTGATTCAAATGTTTTTGCATAGTAACATTTGTCTC

Protein: 2008 aa

>Khc-73_protein

MASSDKVKVALRVRPLNRREIEMGTKCVVDMDDTQTVLYHPTTGVKETLKIIRQDKDAAPNKKSPKVFAFDHCFWSIDKNVPKFASQEKVFACLGQDILERAFEGYNACIFAYGQTGSGKSYTMMGTNTEKGIIPRLCDKLFDSIAHATNSSNSFKVEVSYMEIYNEKVRDLLDPKGGKQHLKVREHSILGPYVDGLSTLAVSSFEDIDGLMLEGNKSRTVAATNMNSESSRSHAVFTIILTQTLTDLASGVSGEKVSKMSLVDLAGSERAQKTGAVGERLKEGSNINKSLTTLGLVISALADSPAKGKIKFVPYRDSVLTWLLKDNLGGNSKTVMVATLSPSADNYEETLSTLRYADRAKRIVNHAVVNEDPNARIIRELREEVEMLRKMLSAAQMNRSPDLQDRLEESEKLMKTMSKTWEEKLRETEEIHQERHAALEKMGISVQTSGIKVEKNKYYLVNLNADPSLNELLVYYLKDHTLIGRPDAPREQDIQLSGLGIMSEHCIIDLVETDVYLTPLEGARTCLNGRTLTERVKVKNGDRIVWGNNHFFRINCPKANSPSAETPEQKIDYDFAQTELMMQECGDDPIQEAIEAIEKQHVEDKAEALERQRQMYERQMQMLRNQLMSPSTPSQPYPPFFVDPFGKGTPGATTPHSSMHPKYQIWAQESLDDYSRKWIGTQEVGWEKLFKSSLAKLKEEVVKANSLVREANFLAEEMGKGTEFSVTLQIPAANLSPNRKRGAFVSEPAILVKRKARNSQIWAMEKFENKIIDMRDLYEDRKEKGLPMKVDDDEDDLPLVIPSDGMDQGPPSKGDPFYESQENHNLIGVANVFLEALFYDVKLDYQVPIISQQGEVAGRLHIELCRVSSSLPDNNSADFDSNDHIDMINNEDSVVVGSNIVCRVSIKEAKGLPPALSNFVFCQYSFWGHPEPIVVPPQVDPDVHKHKNDGVSFRFDHKKEFKINVTEEFIEHCTEGALSMEVWGHRSPGFVPTMPGWEIDHQAKSRSIMDRWSELTRKIEIKTEIHELNDQGEYVAVEVIPKPDVLTGGVFQLRQGHSRRIQVEVKPVQNSGTLPIICESITHISIGAVCARSKVQKGLDSYQEEDLSRLREKWSMALDKRREYLDEQLHKIMNKEAKTDTDIEREKSLVDQWVCLTEERNAVLVPAPGSGIPGAPADWNPPPEMEQHVPVIFLDLNADDMSTPGVKEGLQAAGINSILPKEHGAKFYLLPFVNNDTCAVVSWDSSIHDSVHLNRLTPTNERVYLILKVIVRLSHPASMELVLRKRLCINVYKRQSITERLKKRIGKSVLQSVTGTEYFQDTLCISAVTYEIVSNIPKASEDLEDRESLAQMAASNNEANAVDGETYIEKYIKGVSAVESILTLDRLRQEVAIKELLASRGRPLRKSTSVPNIHGAVNMSPLGGLDNKMRSDSIQDLSYNAERFGSMPKHISLDSLRRVGNDETPPRDSVLLPNKPAGPFNLASPKAGASPQPTKLVKPMRTLMEEQSIRESKPLLRQDDDSDEDEFAKLKEAPPKEGDTSRTGEDSVESDEFQDFESYQGGQENAELPHSNTNDSLVDTHAKSYPSSASSSGYGSQAQSSQTLSSEDNNSVKSLEEGESNAGGDHQRSAQGRTTSESQGEEEGGQRQRSDSLLDTEIPSGLGTPTLLSPEVIHNNKETDGQTDTSQADKDTQRNEANTNTAEHKADTGSKKLNVSSADPYSEDMLQELEQLEGCDESFNISKSMNNTMNTSLNTSINTSMSTSQNLEDHEGSDDTIKSEVKLRKGGTMEVSRPANLKKLRTETRTTKASPSPVKPAPLKATYRPMSMPIDNSLPVHEDEPQSADENNSDNEDAMSMYSFGSRADLSRVGDSPVPTWVQVGEPVIVLSSSGGQKNGTVQFIGPTEFATGTWIGVELDLADGKNDGSVKGVRYFKCRARHGIFVRHEKLMMDKKRRSSSGSTGSLKMKAVAGMTTSLRRSTGNLSASSPGSKDLPGFMRATAATSQKFK

Domains

KISc_KIF1A_KIF1B: 5 – 370

FHA: 457 – 555

KIF1B: 767 – 829

DUF3694: 1188 – 1297

CAP_GLY: 1880 - 1948

Clone

Partial ORF

Primers

Forward: CGAAGGTTTTTGCCTTTGAC

Reverse: AGCGTTCAGGTTGACCAAGT

Nucleotide# 484 – 1685 = 1202 nucleotides

>Khc-73_clone

CGAAGGTTTTTGCCTTTGACCACTGTTTCTGGTCAATCGACAAGAACGTTCCAAAATTTGCAAGTCAAGAAAAAGTGTTCGCATGTCTGGGGCAAGATATCTTAGAACGAGCCTTCGAAGGTTACAATGCCTGTATATTCGCATATGGCCAGACAGGCTCGGGCAAATCATACACGATGATGGGCACGAACACCGAAAAGGGAATAATCCCCCGTCTCTGCGATAAACTATTTGACTCAATCGCCCATGCAACCAACAGCAGCAACAGTTTCAAAGTGGAGGTCTCATACATGGAGATATACAACGAGAAAGTGCGAGATCTCCTCGACCCCAAGGGAGGCAAACAGCACCTAAAAGTGAGAGAGCACAGTATTTTAGGACCGTATGTGGATGGATTGTCAACTCTGGCTGTGTCATCCTTTGAGGACATCGATGGCCTCATGCTGGAAGGAAATAAATCTCGTACAGTCGCAGCCACAAACATGAACAGCGAAAGCAGTCGGTCGCACGCTGTGTTCACCATCATCCTCACACAGACCCTGACAGATCTGGCTTCTGGGGTTTCGGGAGAGAAAGTATCCAAAATGAGTTTGGTTGATTTGGCGGGAAGCGAGCGAGCCCAGAAGACGGGAGCCGTGGGGGAAAGGTTGAAGGAAGGAAGTAACATTAATAAGTCCCTCACAACTCTGGGTCTTGTAATATCAGCCCTTGCAGACTCACCGGCAAAAGGAAAGATCAAATTTGTGCCCTACAGGGACTCCGTATTAACATGGCTGCTAAAGGACAACTTAGGAGGAAACAGCAAAACAGTCATGGTGGCCACGCTGAGTCCTTCTGCAGACAACTACGAGGAAACGCTTTCGACATTACGATACGCAGACAGGGCCAAACGTATCGTCAATCACGCTGTGGTCAATGAAGATCCGAACGCACGTATCATCAGAGAACTGCGGGAGGAGGTGGAGATGTTACGGAAGATGCTTAGTGCAGCTCAGATGAACAGGTCTCCGGACCTCCAGGACAGATTGGAGGAGTCGGAAAAGTTAATGAAGACCATGAGCAAGACGTGGGAGGAGAAATTGCGGGAGACTGAGGAGATCCACCAGGAAAGACATGCTGCCCTGGAGAAGATGGGCATTTCAGTGCAGACTTCTGGGATCAAAGTCGAGAAAAACAAGTATTACTTGGTCAACCTGAACGCT

Cloning info

PCR rxn on 05/10/16 using GoTaq polymerase mix

| Template | Initial denature | Denature | Anneal | Extension | #cycles | Final extension |
| --- | --- | --- | --- | --- | --- | --- |
| 2 hr cDNA | 4min @ 94C | 30sec @ 94C | 30sec @ 55C | 2min 15 sec @ 72C | 35 | 10min @ 72C |

**Aurora A**

Gene model: 1990 nucleotides

ORF: 499 – 1443 = 945 nucleotides

>Aurora A_full

TGACATTCTTTGAATCCAGGGTCTCATAGTTTACTGCTGCCATATTCCTTGTGAGTTTCAGCAGCCGATGGCACTTAAGATTTTGTATTCCACTGCAGTCTTCCTATTAGGTTGTTCTCCGTATTTCAATGACTTGAATCTGGGACCCTTTCTTCAGTTTCTTCTGCATGTTCTGTCATCTTCACAGTGCTGCAGTGCGATCGTTGCGTAATTTGTGTACACTCGCTTGGAGCAAGGCGCGCTCACGCGACAGCACCTCAGGCATTTCCCAGCAGGCCACAGCGCGTCATAATACCATTGGGTCAATGGGAGTGTCGCTTCTGTATGTTATTCTGTGGTGCAGCCTCGCCTGGTTCACCGCTACAACGAATCAGCGTCCCCGACACATTCAGCGAACCCTCCACGCGCAGCCAATCACAGCGCGTCTTACAAATTTTCAAACGAAACAGCGGGACGAAAATTTTAGCAGGTTGCCAGTGACAGGAGCTGATTGCAATCATGGGAAATGAAAACGACCCGAACACACAAAATGGATCAGGAGACAACAAGATGGCCGAGGACACAAGAGCAAAGAAAAAGTGGACCCTGGATAACTTTGAGATTGGCAAACCCCTGGGCAAGGGGAAATTCGGAAATGTCTACTTGGCCAGAGAGAAGGAGTCAAAGTTCATTGTTGCCCTAAAGGTTTTGTTCAAGTCTCAGCTTCAGAAGGCCGGAGTGGAGCATCAGCTGAGAAGAGAGATTGAGATCCAGTCCCACTTGAGACACGAAAACATTCTGCGTCTGTACGGCTACTTCTACGATGACAAGCGAATCTACCTTATCCTGGAGTACGCCCCCAGAGGAGAGTTGTACAAGGAGTTGCAGAGGAATGAGAAATTCCCCGAGGGAAGATCTGCCACCTATGTGTACCAGTTAGCAGATGCCCTGGAGTACTGCCATTCCAAGAAGGTCATCCACAGAGACATCAAGCCAGAGAATCTCCTCTTAGGGCTGAGGGGGGAGCTCAAGGTGGCTGACTTCGGCTGGTCCGTGCACGCTCCCACTTCAAGACGTAACACCCTGTGCGGTACCTTGGACTACCTGCCCCCAGAGATGGTCGAAGGAAGACCTCATGACGAGAGAGTTGACCTCTGGAGTTTAGGAGTACTGTGTTACGAGTTCCTAGTGGGACACCCTCCTTTTGAGACTGCTAGCCACAATGACACGTATCGTAGAATCGCCAGAGTGGACCTCCGTTTCCCTAACTTCGTCTCTGAAGAGGCTACTGACTTCATACGCAAGCTTCTCCGCCACGACCCCGCCCAACGTCTCCCCCTCCAACAGGTCAAAGTTCACCCCTGGATCGTAACCAACGCCTCGATGTCAGCGCCCTCTACAGCTAAAGCAGCCACGACCATGAGTAGGATTAATAACAACAACAAGACCACTCCTGCCTCTTGAGGAGAGCAGCGGTACTAAGACTCATAAGTGACTTATCAATGAAAGACTATATCAAATGTGTCAATTAATGTGATGATCGACCCTGCTGTCGCTGGATGCTGCTGCAGAACTTTTTGAAAACAACAAGTTTGTACAACATGTACAAGGGAACTGGTGGGTGGAAATAAGTTTTCTTTTTTCTTGCAAAGTTTTTCTGCTGGATATCAACTGAACTTGAAGTTTCAGAGAGAGCTCTCTAAAATTGTATTGCCGTATATGCCCTACAGTGCACATACTGCCTTAACAAAGTAAAAAATTGTGTATTTCTGGCCTAAATTCAAATTATTCTTAATCAGCTCCTGTATTCCTTAAAATGTTGTGTGTGATGCAAGCCGTAGACACTACTCAGTACTGTGCAATTTTATAATGATCATTGTGAAAATAAGTAGTCATTCCAATATGTAATAGTGTGTGGTATCACAAACTTGTAGTCATATAAATTTACAAGTTGTTGAACAAATGTTAAAGATGACATTTGAATGACTTTTTAGCTAGCCATAGGCGTAGC

Protein: 314 aa

>Aurora A_protein

MGNENDPNTQNGSGDNKMAEDTRAKKKWTLDNFEIGKPLGKGKFGNVYLAREKESKFIVALKVLFKSQLQKAGVEHQLRREIEIQSHLRHENILRLYGYFYDDKRIYLILEYAPRGELYKELQRNEKFPEGRSATYVYQLADALEYCHSKKVIHRDIKPENLLLGLRGELKVADFGWSVHAPTSRRNTLCGTLDYLPPEMVEGRPHDERVDLWSLGVLCYEFLVGHPPFETASHNDTYRRIARVDLRFPNFVSEEATDFIRKLLRHDPAQRLPLQQVKVHPWIVTNASMSAPSTAKAATTMSRINNNNKTTPAS

Domains

STKc_Aurora: 32 - 284

Clone

Partial ORF

Primers

Forward: GGGGAAATTCGGAAATGTCT

Reverse: TGGCGATTCTACGATACGTG

Nucleotide# 621 – 1225 = 605 nucleotides

>Aurora A_clone

GGGGAAATTCGGAAATGTCTACTTGGCCAGAGAGAAGGAGTCAAAGTTCATTGTTGCCCTAAAGGTTTTGTTCAAGTCTCAGCTTCAGAAGGCCGGAGTGGAGCATCAGCTGAGAAGAGAGATTGAGATCCAGTCCCACTTGAGACACGAAAACATTCTGCGTCTGTACGGCTACTTCTACGATGACAAGCGAATCTACCTTATCCTGGAGTACGCCCCCAGAGGAGAGTTGTACAAGGAGTTGCAGAGGAATGAGAAATTCCCCGAGGGAAGATCTGCCACCTATGTGTACCAGTTAGCAGATGCCCTGGAGTACTGCCATTCCAAGAAGGTCATCCACAGAGACATCAAGCCAGAGAATCTCCTCTTAGGGCTGAGGGGGGAGCTCAAGGTGGCTGACTTCGGCTGGTCCGTGCACGCTCCCACTTCAAGACGTAACACCCTGTGCGGTACCTTGGACTACCTGCCCCCAGAGATGGTCGAAGGAAGACCTCATGACGAGAGAGTTGACCTCTGGAGTTTAGGAGTACTGTGTTACGAGTTCCTAGTGGGACACCCTCCTTTTGAGACTGCTAGCCACAATGACACGTATCGTAGAATCGCCA

Cloning info

PCR rxn on 07/28/16 using GoTaq polymerase mix

| Template | Initial denature | Denature | Anneal | Extension | #cycles | Final extension |
| --- | --- | --- | --- | --- | --- | --- |
| 2 hr cDNA | 4min @ 94C | 30sec @ 94C | 30sec @ 55C | 2min 15 sec @ 72C | 35 | 10min @ 72C |

**Bicaudal D2**

Gene model: 3822 nucleotides

ORF: 221 – 2647 = 2427 nucleotides

>Bicaudal D2_full

CGCTTTTCTACCTGCTTTTTAGCAAGTCCCGCGTCTTGCTTTCAAGAACAAAATGCGCGCGAAACATGTTGGGATCTGCGCGAGTTGGATCTCAGGTCACGTGACCCGCTAAGACACAATTTCAATATGGCGGCTGTCCTGGGTGAGGAATTTCAGATCCCCGTCCACTAAAATTCGGTAATTTTCGAGAAAATCGTGCGTCTAGATACACGAAACCAAGATGTCGACAACCGACGACACCCAGGGTACATTTGACGAGCTCAACCAGGAAATCAACCGGCTGCAGAGCGAGCTAGCCGAGGTTACTCAGGAACGAGTGCAGGCTGCTGAATATGGCCTTGCAGTTTTGGAGGAAAAGCAAAACCTGGAACTCAGATTTGAAGAGCTCGAAAACCTGTATGAGACCGCCAAACACGACTTAGAATGTGCGAAAGAGAATCTTAAGCAAGTGCAACAGACACACAAGAAGGCTAACCAAGTGGGCATCCATGCCGAGGAAAGTCTTTTGGCCGAGAGTGCAACCCGAGAGGCCCAGCTTCTAGAGACCATAACGGAGCTCGAAGCTGAGTTGAAACATGCAAGAAACGAGGTGGAGAGACACCGCTCTGACTCGGAGCGGGTTGGCACCCTCAACCAAGACTTCACCCAAAAGATAGAACAACTGGAAACGGAACGGAAGCAGATGAAGAAAGAAATCAAGGAACTGAAATTCAGAGAATCCCGCAATCTTGCAGACTACTCAGAGCTGGAGGAAGAGAACATCTCTTTGCAAAAGGCTGTGCTGCAGCTCAAGCAAAACCAAGTTGACTTTGAAGCAATGAAGTACGAGAATAAAAGATACAAAGAAGAAACAGAAGAGCTCAATGCAGAGCTAGAGGAGTTCGCTAAATTGAAAAATATAGTTGAGAAAAATCTCGAAGAAGCTCTGAACTCATTGCAGCAAGAGCGGGAACAGAAACATGCCTTGAAGAAGGAGCTGGACCAGAGGTTGACCAACGAGTCCATGTACAACTTGCACACGTTAGCAAGCCTCGGCCTAGGAGAGTTTAAGATGCCCAGTAGAGATCGCACGGAATTCAACGATGGCCATGAAGATCAAGACTCCCATGGTAATCCTGCGCTTAAGAGAATAGAAGCAGACTTTTCGTCGCAGAAGAAGAGTCTGGAAGACCAAGAACCTGCACCAGCCTCAGGCCTTGTAGGCGATCTTTTCTCCGAACTACACATGACAGAAATCCGCAAGCTTGAGCAGTTGCTCGAACAGGCAGAGATTGAGAAATCTGCCATGCAATGTTCGCAGCAAGAGATTCGAACAGAACTGGAGAAAACCAAGCTGACAATGTCGGAACAACAGGAAAAGATCGCTCAAATGAAGGCTCACATGAGTGCGATGTCCGCAGGAAGTCACCCAGCCTCTTTACTGGACGGGGAGGAGGCAACGGGTGACGAGAATGACACTTCAGAGGAGGGAGTCAACAAGCGCAACCTGCAACAGGCTGAACGAAGATACGCAGCCGCCCTGACCCAGATCTCTGAACTCCAGGAGCAGATCAAGAAACTGGAGTCGATGAGAGGAAAGGACGATCAGAATGAAGATGACGTCAAGGACGAACTCAAGAAATTGAGAGACCGGGTACAACAGTACGAGGAGACCATCAGCAACCTGGAGGTGGACCTCAAAGCCATGACACAGATGGCTGGAGAGAGTCAGGGCAATCTTAACACAACTCAAGATGAACTGACGAAAGTTACAGAAGTACTCGCTCAGCTGTACCACCATGTGTGTGAAGTGAACGGCGAAACACCAAACAGGGTCGTCCTGGAACACGTCCAAGGCAAGAAGTTCAGACGCCAGGACAGTCCTAAGGACGGAGACGGGAGTGAGAAATCTGGGTCAGGGAAAGAGGAGTCGGATAAAGAGAGCTCGCCGAGCAGAGAAACACCAGACAAGGATAACGACTCCAAAGAAGATCCAATGACCTGCTATAAGCTGTGCGAGACGATCCACGACCAAGTCAAGTACCTGAAGAGGGCGGTCGAGAGAACCATTGAGCAGTCTCGTACACGCCACGTGGATCCCAATCTCAACACAGATGTAGCGGAGTTGCAGGAGCAGGTGGTTAAGCTCAAGGCCATGTTGAACACCAAGAGGGAGCAGATCGCTACATTAAGAAGTGTCCTCAAAGCCAACAAATCAACGGCAGAAATCGCTTTGGCTAACCTAAAGCAGAAGTATGAGAATGAGAAGTGCATCGTCACGGAGACGATGATGAAGTTGAGGAACGAACTGAAGGCTCTCAAGGAAGACGCAGCGACATTCGCGTCCTTGAGAGCAATGTTCGCCCAGCGGTGTGACGAGTATGTCACTCAGCTGGATGAACTGCAACGCCAATTAGGCGCTGCCGAGGAAGAAAAGAAAACATTGAACTCGCTACTTAGGATGGCCATCCAGCAAAAATTAGCCCTCACGCAACGACTTGAGGACTTGGAGTTCGATCGTGAACGTCGCAACATTCGACAAGAGAGAAGAGGAGGGGGAGGCCGCAGCAAAATGGGTTCTGCCAAAGTAAGTTTCAACAATTACAATGATGATTCTTCATACAACCGCTATCAGCCCCCACCTCGATTTGCGCGGAGAGATTACTAAAAGACTAAGAGTGCACGCAGGATGGAAGTTGGCATGACTATCAGTTGCAGTTGGTCACTCGCTATTGACCCCTGGATTTACCCTGGATGAAGTTCCCTGGACGAACCAAAGGTCAATGAACTGCAAGTGATGGTCAGTGCCCTCCTGCGCCAAGCACCAAATTGGGGCGAAAGGGGTTTGATTGGTTGCAGAGTATCTCCTATGCTGCTGTGATAGAATTGTGGGGTATTCTCACAGTACAAGGGGAATGTACATGTTGTATAGCCGCAAAGAGCTAACCTTCTATAGTTGATTTAGGGCTTCCAATCCTTGTGATCATTGTGATATCTATCTCTAAGTGAAACCAAGTAGTTTTCCTAAACCTTGTAGTCTTGCATGACTAACACTAGTTTTAGTTTCATAATTATCTTGTGTGATTTGTGCAGTGTACTGACGCACTAGTTCTTAATCAAATTTCAGTTTTTACTCAAATTTATGAGCATGATTTTGTGCAATACCTCAGTTTTTAATGCATGCTTCACAATTATACAGTTTGTAATATGCTGTCTTTTAATAGAAGTCTTAAAACTTGGATTTTAATTTCGCTCTACTTGTTGTTATAATCTACAATAACATGTTCTTAATTCCTTTGAGATCATTTTATCAATCATACACATTCTTCTGATAAAGACACATTTTAAACATTTGTTTTGGAATGCTCATATCTCAATGTAAATATCAGTATTTATTGTTCGTACTTTGAAACAAGTACCTTGGTGGTTAATTATAACCGTTGATAGACTGACAGTTATTTTTTAAGAAGAGTGAAAGGATCTATTTTAATGAACAAATTTGGCGGGAAATCACATATCTCTCGATATGGAATTGGGTTTTTAACACTCCGGAATTATACGGGACATATGGATCATTATCACGATTATATTAAGCCTGGAATCCGGTTTTGAGATTAAGAAATTATGTACACAACGGAAATTAATGGAATTATTGATATCAATTAATTGATCATGATTTGATAAATTGACAATTGACCTCATGTGACGTGCAGTTGATCTGCACGCACAGGAATACATCATGGACTCCAAATTGCTTCATAACTACTCATTATAACTGCCTTGGAAAAAGGAACCAAGTTCATAACTTGTGTTCTTGACTTTTACTAGTGTTCATAGGTCAAG

Protein: 808 aa

>Bicaudal D2_protein

MSTTDDTQGTFDELNQEINRLQSELAEVTQERVQAAEYGLAVLEEKQNLELRFEELENLYETAKHDLECAKENLKQVQQTHKKANQVGIHAEESLLAESATREAQLLETITELEAELKHARNEVERHRSDSERVGTLNQDFTQKIEQLETERKQMKKEIKELKFRESRNLADYSELEEENISLQKAVLQLKQNQVDFEAMKYENKRYKEETEELNAELEEFAKLKNIVEKNLEEALNSLQQEREQKHALKKELDQRLTNESMYNLHTLASLGLGEFKMPSRDRTEFNDGHEDQDSHGNPALKRIEADFSSQKKSLEDQEPAPASGLVGDLFSELHMTEIRKLEQLLEQAEIEKSAMQCSQQEIRTELEKTKLTMSEQQEKIAQMKAHMSAMSAGSHPASLLDGEEATGDENDTSEEGVNKRNLQQAERRYAAALTQISELQEQIKKLESMRGKDDQNEDDVKDELKKLRDRVQQYEETISNLEVDLKAMTQMAGESQGNLNTTQDELTKVTEVLAQLYHHVCEVNGETPNRVVLEHVQGKKFRRQDSPKDGDGSEKSGSGKEESDKESSPSRETPDKDNDSKEDPMTCYKLCETIHDQVKYLKRAVERTIEQSRTRHVDPNLNTDVAELQEQVVKLKAMLNTKREQIATLRSVLKANKSTAEIALANLKQKYENEKCIVTETMMKLRNELKALKEDAATFASLRAMFAQRCDEYVTQLDELQRQLGAAEEEKKTLNSLLRMAIQQKLALTQRLEDLEFDRERRNIRQERRGGGGRSKMGSAKVSFNNYNDDSSYNRYQPPPRFARRDY

Domains

BicD: 76 – 758

Clone

Partial ORF

Primers

Forward: TCACCCAGCCTCTTTACTGG

Reverse: CCTTGAGAGCCTTCAGTTCG

Nucleotide# 1405 – 2303 = 899 nucleotides

>Bicaudal D2_clone

TCACCCAGCCTCTTTACTGGACGGGGAGGAGGCAACGGGTGACGAGAATGACACTTCAGAGGAGGGAGTCAACAAGCGCAACCTGCAACAGGCTGAACGAAGATACGCAGCCGCCCTGACCCAGATCTCTGAACTCCAGGAGCAGATCAAGAAACTGGAGTCGATGAGAGGAAAGGACGATCAGAATGAAGATGACGTCAAGGACGAACTCAAGAAATTGAGAGACCGGGTACAACAGTACGAGGAGACCATCAGCAACCTGGAGGTGGACCTCAAAGCCATGACACAGATGGCTGGAGAGAGTCAGGGCAATCTTAACACAACTCAAGATGAACTGACGAAAGTTACAGAAGTACTCGCTCAGCTGTACCACCATGTGTGTGAAGTGAACGGCGAAACACCAAACAGGGTCGTCCTGGAACACGTCCAAGGCAAGAAGTTCAGACGCCAGGACAGTCCTAAGGACGGAGACGGGAGTGAGAAATCTGGGTCAGGGAAAGAGGAGTCGGATAAAGAGAGCTCGCCGAGCAGAGAAACACCAGACAAGGATAACGACTCCAAAGAAGATCCAATGACCTGCTATAAGCTGTGCGAGACGATCCACGACCAAGTCAAGTACCTGAAGAGGGCGGTCGAGAGAACCATTGAGCAGTCTCGTACACGCCACGTGGATCCCAATCTCAACACAGATGTAGCGGAGTTGCAGGAGCAGGTGGTTAAGCTCAAGGCCATGTTGAACACCAAGAGGGAGCAGATCGCTACATTAAGAAGTGTCCTCAAAGCCAACAAATCAACGGCAGAAATCGCTTTGGCTAACCTAAAGCAGAAGTATGAGAATGAGAAGTGCATCGTCACGGAGACGATGATGAAGTTGAGGAACGAACTGAAGGCTCTCAAGG

Cloning info

PCR rxn on 07/28/16 using GoTaq polymerase mix

| Template | Initial denature | Denature | Anneal | Extension | #cycles | Final extension |
| --- | --- | --- | --- | --- | --- | --- |
| 2 hr cDNA | 4min @ 94C | 30sec @ 94C | 30sec @ 55C | 2min 15 sec @ 72C | 35 | 10min @ 72C |

**Dynamitin**

Gene model: 1909 nucleotides

ORF: 35 – 1234 = 1200 nucleotides

>Dynamitin_full

CTTTTATCCCCGTGCGGAAGCTCCTGCTGTCAAGATGGCGGATCCAAAATATGCTAGTCTGCCTGGAATAGATCTTAATTCCCCTGATTTGTATGAGACGAACGATCTTCCGGAAGATGATCAAGCGCAGGTTGACCAGTCTGATCAGGGAAATGAGAACGTTGAGAAGATCAATGTCTCCGCCACAGATGCTTATAAGAAGTTTGAGGGTAAAACCTTAGAAGGTGGAAAAGTTGATTTCTCAGACCGAGTCAGTGGTTCCAGGCGCATTGGTTATGATGCGCCTCAAACAGAGTATGAAATGCAAGCGGTTGAGAGAGAGCCTGAGACTCCTCAGCAAAAATACCAACGTTTGCAACATGAAATCAGGGAACTACAAGAGGAGGTCTCCAAAGCTAAGGAGAATGTGAAAGCGGATGCTGAGACTGAGAAAGTGTCTCCAGTTCCTTTAGTGAAGCAAGCAGAGTATCTCCAGCAACAATTAACCGACCTTCATCTGGATAAACTTCTAGGAGATCAAGCTGAGGTCAACTTGGCCGACCCTCAGAGGGCTTTGCATAAGCGTCTTCTGACTGAGCTGGACTCTTACAAACCTACTGAAGCCCAGGCCAAATCTGAGAAAGATGCTCCTTCAGGGAATCAAGTCACATATCAGCTCTACTACAGACCGGAGCAGGCCAAGTTCACCAGCAATGCCAGGGCTGCCAATATTGAGCAACGTCTTGAGCGCATGGAAGCACTCATCGGAAACAATCCAGAAAAGCTTTCTATGCTGACAGCTGAAACAAGCAACAAGAGTATTGTGGGTGCTGTAGGAGTGTTGAGTTCCAAGGTGAAATTGCTGGATGCCACCCAACTGGAACAAGTGGAGGGAAGACTTCACGCTCTGTCTCAGAGACTCACCAAAGTTGTGGAGAACAAAGAGACCGCCGAGGAGGCAGACAAGACTGCCAAGGTCAGTGAGCTTTTTGAAATGGTCAAGAAATGGGATGCAGTTGTTGACACATTGCCCCATGTAGTTGATAGACTTACGGCTCTCAAGGAACTCCATGAACAAGCACTCCAATTTAGTCAGGCCCTCAACCACTTGGATGCGGCCCAACAACAAGTGTCTAGCCACATGAACGCACACAGTGATATGTTGAAACAGGTCCAAACCACGCTGACAGAAAATCTTTCAACACTAAAAGATAATTGCGCGAGTATAGACGACCGAGTGAAGGCTCTCAAGTGAACAAATTGCAGAATTAGCTTATTTATGGAATTTATTTATGTCTTAACTTGAATTAATGAGTACCAAATTCATAACTGGGTATTACCTTCATGTACAGTGTAATTATTTATAATATACATATATTGGTATATAATAATGTACACTACATGGCTTCCGACAATATTATTGGTGACATGAATTTTCGCTTTCAAAAAGTGCATACTTAAGTGCAATAGGTGTTAATTATTACGCAAAGCAGCTGTTTGTATCATGGCTTGTTCCTCTGGTGGCTTAGACGTTTGAGCTGTTGCTGATATGTATTTATAAGCGCTCTAGGTAGGATCTGGCAAAACTGATGTTGGTCTTTCGATTGTTGAATCTATGAAGGGTGGACGGTATCAATTTTTTTATGCATGATTACATTTTGTTTCTGTGCCTTGGAATTTCCAGGAAAGTGGACGCTATAAACCTGCAAGTGACTTTCCACTTGTTTTCCAATTTTTTGAGGATTTGACATCAGAGTATTCCTTGAAGAATCATATTAAAAAATGCGGCTCTCCACAAGTTGAATAAGTTATAATGAGAAGCCTTGGTGTTCGGTGTTCAATACACAATCAAAACACTCTTCTTGTGATATAATGTAAAATATATGAGTTTTGTTTCTTTGCTTTATTGTAACAAATAAAGAATATTAAC

Protein: 399 aa

>Dynamitin_protein

MADPKYASLPGIDLNSPDLYETNDLPEDDQAQVDQSDQGNENVEKINVSATDAYKKFEGKTLEGGKVDFSDRVSGSRRIGYDAPQTEYEMQAVEREPETPQQKYQRLQHEIRELQEEVSKAKENVKADAETEKVSPVPLVKQAEYLQQQLTDLHLDKLLGDQAEVNLADPQRALHKRLLTELDSYKPTEAQAKSEKDAPSGNQVTYQLYYRPEQAKFTSNARAANIEQRLERMEALIGNNPEKLSMLTAETSNKSIVGAVGVLSSKVKLLDATQLEQVEGRLHALSQRLTKVVENKETAEEADKTAKVSELFEMVKKWDAVVDTLPHVVDRLTALKELHEQALQFSQALNHLDAAQQQVSSHMNAHSDMLKQVQTTLTENLSTLKDNCASIDDRVKALK

Domains

Dynamitin: 17 - 398

Clone

Partial ORF

Primers

Forward: TTATGATGCGCCTCAAACAG

Reverse: TTTTCTGTCAGCGTGGTTTG

Nucleotide# 274 – 1173 = 900 nucleotides

>Dynamitin_clone

TTATGATGCGCCTCAAACAGAGTATGAAATGCAAGCGGTTGAGAGAGAGCCTGAGACTCCTCAGCAAAAATACCAACGTTTGCAACATGAAATCAGGGAACTACAAGAGGAGGTCTCCAAAGCTAAGGAGAATGTGAAAGCGGATGCTGAGACTGAGAAAGTGTCTCCAGTTCCTTTAGTGAAGCAAGCAGAGTATCTCCAGCAACAATTAACCGACCTTCATCTGGATAAACTTCTAGGAGATCAAGCTGAGGTCAACTTGGCCGACCCTCAGAGGGCTTTGCATAAGCGTCTTCTGACTGAGCTGGACTCTTACAAACCTACTGAAGCCCAGGCCAAATCTGAGAAAGATGCTCCTTCAGGGAATCAAGTCACATATCAGCTCTACTACAGACCGGAGCAGGCCAAGTTCACCAGCAATGCCAGGGCTGCCAATATTGAGCAACGTCTTGAGCGCATGGAAGCACTCATCGGAAACAATCCAGAAAAGCTTTCTATGCTGACAGCTGAAACAAGCAACAAGAGTATTGTGGGTGCTGTAGGAGTGTTGAGTTCCAAGGTGAAATTGCTGGATGCCACCCAACTGGAACAAGTGGAGGGAAGACTTCACGCTCTGTCTCAGAGACTCACCAAAGTTGTGGAGAACAAAGAGACCGCCGAGGAGGCAGACAAGACTGCCAAGGTCAGTGAGCTTTTTGAAATGGTCAAGAAATGGGATGCAGTTGTTGACACATTGCCCCATGTAGTTGATAGACTTACGGCTCTCAAGGAACTCCATGAACAAGCACTCCAATTTAGTCAGGCCCTCAACCACTTGGATGCGGCCCAACAACAAGTGTCTAGCCACATGAACGCACACAGTGATATGTTGAAACAGGTCCAAACCACGCTGACAGAAAA

Cloning info

PCR rxn on 05/10/16 using GoTaq polymerase mix

| Template | Initial denature | Denature | Anneal | Extension | #cycles | Final extension |
| --- | --- | --- | --- | --- | --- | --- |
| 2 hr cDNA | 4min @ 94C | 30sec @ 94C | 30sec @ 55C | 2min 15 sec @ 72C | 35 | 10min @ 72C |

**Dynein Heavy Chain 1**

Gene model: 15065 nucleotides

ORF: 247 – 14241 = 13995 nucleotides

>Dynein Heavy Chain 1_full

TTTATATTGAAGTTTTTAATGTTAAATAAGTATATTGTACGCCAAATAATTACTTTAATCTAATTATTTTCCCACAGTGGATGACCTCATCTCAACAGGCCCCGGATGACAAAGTGGGAAGCGGCCATTTTCGCGTCAGGCTGCCGACTATGAACGTGGAGAAAAAGTCTTTAAGACAGTGGATTTATGTTAAACTACAGTAATTAGTTGCTGGAGGGTTGGTTGAAGTATTGTTAGCCATTCAGGATGGCTGACAATAGCGATACAGAGGCCGCACAAGGCCAGGGGTCTGCCTCGCAGGCTCTGATGGATCAGTCAGTTTTGGGGAACTACATTCGGAGAATCGTCCCGGTTAATTTGGAAGATTCAGATGACACCCCAGCTGCTCTGGTCGCATCTTTGAAAGATAAAGCAACCGTGGATTGTATGAAAAAGTTCATTTCAGACCCACAGGTGCAGTGTCTGTTAATTCAACGCCAGACAATCAAAGATGAGTCTGTGGATGTCGAGGGTGACGGAGAAGCTGTCAATTACTTCATCACCCCTGAGGTGCTATACATCAACAGTAAAATGAACAGCTTGTGTTTGATGAAGAAAGGACCACTCCTGGAGGCTGACAAGAGTTGCTCGTCTCAGATCCGACTTATGACTCTGAGCGAAGGTTCTCCTTACGAAACTCTGCATGCCTTCGTCAGCAATGCCATGGCTCCGTTCTTCAAGTCATATGTGCGTGCTACTGGGAAAGCTGACAGAGATGGAGACAAGATGGCTCCCTCAGTGGAGAAGAAGATCGCGGAGTTGGAGATGGGTCTTTTGCATCTTCAGCAGAACATTGACATCCCGGAGATCACTCTGACCATCCACCCAACAATCGCTCAGTACATCAAGAAGTGTGGAGAGCAATCGAAAAAGCCCACGGTGGCCGATTTTGACGAGAAAAAGGAAGATGCCGGATTCTTGAACACACTCCAGAATGGAGTAGGAAGATGGATTCGAGAACTTCAGAAGGTCACTAAATTGGATCGTGACCCAGCATCAGGCACAGCCTTGCAGGAGATCAGTTTCTGGCTCAACTTGGAGAGAGCTCTGTTGAGAATCCAGGAAAAAAGGGAAAGTCCTGAAATCACTCTAACTTTAGATATTTTGAAGCAGGGAAAGAGATTCCACGCTGTTGTTAGTTTTGACACTGATACAGGTTTGAACAAAGCAATTGAAATGGTGAATGACTACAATCCTTTGATGAAGGACTTCCCTCTTAATGATTTACTCTCCGCTACTGAATTAGACAGAATCAGGGTTGCTCTTCAAGCCATTTTCACTCATTTGCGAAAGATCAGGAGCACAAAATACCCCATTCAAAGAGCCTTGCGTCTCGTGGAGGCCATATCTCGCGATTTGAGCCAACAGCTTTTGAAGGTACTCGGCACTCGTCGCTTGATGCATATTCCTTTTGATGAGTTTGAGAAAGTCATGGGAGCTTGCTTCGAAGTCTTCGGTACCTGGGATGATGAGTACGAGAAACTGCAAGGACTTCTGCGTGACTTGGTGAAGAAGAAGAGGGAGGAGCATCTCAAGATGGTCTGGAGGGTCAACCCCGCTCACAAGAAGTTGCAGGCCAGGCTCGATCAGATGAGAAAGTTCCGCAGACAGCATGAACAGCTGCGATCTGTCATCATGAGGGTTCTTCGCCCTGCTGCTCCTCGCCCTGGCAGCCCCACCCCAGAAGAGACCGGAGGTGAATCCAAGAATGACGGAGTGGCCCTGGATGCGGCTGATGCTAATGCCATTGAGGAGGTGAATCTTGCTTACGAAAATGTCAAGGAGGTTGACTGCTTGGATGTCTCTAAGGAAGGCACAGACGCTTGGGAAGCTGCTACAAAAAGGTACGATGAACGTATCGACCGAGTAGAAACAAGGATCACTGCTCGTCTGAGGGATCAGCTCGGAACAGCCAAGAATGCCAATGAGATGTTCAGGATCTTCTCTCGATTCAACGCTCTGTTTGTGAGACCTCACATCCGAGGAGCCATTCGAGAATACCAGACGCAGCTCATTCAGAGAGTGAAGGATGACATCGAGTCTCTCCATGAGAAATTCAAGGTCCAGTATCCTCAGAGCAAAGCATGTCGCATGAGTCAAGTGCGCGACTTGCCCCCAGTGTCTGGCTCCATCATCTGGGCTCGTCAAATCGAGAGACAGCTCAATGCTTACATGAGAAGAGTGGAGGATGTCTTGGGAAAAGGATGGGAGAACCACGTGGAAGGACAGAAGCTGAAATCTGATGGAGACAGTTTCAAGATCAAGCTGAACACACAGGACTTGTTTGAGGATTGGTCTCGCAAAGTTCAGCAAAAGAACCTGGGAGTGTCAGGGCGCATCTTTGCGATCGAGAGTGTCAGGAGTCGTCAAGTTGGCGGAAAGGGGAGTGTCCTTCGTCTCAAGGTCAACTTCTTGCCGGAGGTCATCACTCTCTCCAAAGAGGTGCGCAACTTGAAATGGCTAGGATTCAGGGTGCCCCTGACCATTGTGAACAAGGCGTACCAAGCCAGCCAGCTCTATCCTTTTGCCATTTCCCTGATCGAGAGTGTCAGAACTTACGACAGGACTTGCGAGAAGGTCGACGAAAGACCAAGCATCTCTCTGCTGGTTGCAGGAATGAGGAAGGATGTCCAAGGACTTTTGACTGAGGGTATTCACCTCGTCTGGGAGTCTTACAAGTTGGACCCCTACGTTCAGAGATTGGCTGAGAGCGTCTTCAATCTGCAAGAGAAGGTTGATGACCTCCTGGTCATTGAAGAACAAATTGACATGGATGTGAAGAACCTCGAGACATGTCAGTACAGCTTCACGACCTTCAACGAGCACCTGGCCCACATCCAGAAGAACGTGGACGACCTCAGCTTGAGACAGTACTCCAACCTGCCTCAATGGGTCGCCAAGCTGGACCAAGAGGTTGAGAAGAAACTGGCATTCAGGCTGGAATGTGGCTTGGAGGCCTGGACCAAGGCTCTTCTTGAGACTGAGGAGGAACAGATTGACAACAGCATGGACACTGACGCCCCCAAGACACCCAAGCACAAGCCAGGCGGTGAACCCATCATGAAGACCCTGCACCACGAGCTGCATATCACCAACCAAGTGATGTACATCAACCCGTCCCTGGAAGAGGCTCGTTTCAAGACCATCCAGGAGTTGTTCGCTTGGGAGCAGGTCATTCTCACTCTGCCCCGCATCCAGCACTCCAGATACCAGGTTGGACTGGATGTTGAGTCAGAAGCTGCAACCCACTACAAGAACCTCTTGAACAAGTTGCCCCAGCATGGCAACAAATTGAAGGACGCCTACAAAGCTGTAGAGGAAGTCCTCCAGAAGGCTTCAGAATATGTTGACATGTGGATGAGATACCAAGCTCTGTGGGATCTCCAGTCTGAATTGTTGTATGGTCGTCTTGGCGAAAGCAACATTCAACAATGGATGAGAACTTTGGAAGAAATCAAGAGAGTGAGGAAGACCTTCGACACCTCAGAGACAAAACAGGTCTTTGGACCTCTCCATGTGGACTTCGCTAAGGTCCAGGCCAAGGTCAATCTGAAGTACGACTCCTGGCACAAGGACGTCCTCAGCAAGTTCGGAAACTTGCTCGGTGGAAAGATGCAAGACTTCCACAACAGCGTTTCAAGGTCAAGAGGTGAGCTGGAACAGCAGTCGATTGAAACTGCCAACACTGGCGAGGCCGTCAGCTTCATCACCTACGTGCAGTCTCTGAAACGCAAGATGAAGAACTGGGAGAAGGAAGTCGAGATGTACAAGGAAGGTCAGCGCATCCTGGAGCGCCAGAGGTTCCAGTTCCCCTCCAACTGGCTGTACAGCGACAACGTCGACGGAGAGTGGGGTGCGTTCAATGACATCATTCGCCGCAAGGACTCCTCCATCCAATCCCAGGTGGCATCACTGCAGATGAAGATCGTCTCGGAGGACAAGGTCGTCGAAACGAAGACTCACGACCTCCTAGGAGAATGGGAGAAGAGCAAACCAGTTGAAGGTCAGATGAGGCCTGACAATGCCATCAAGGCCCTGACCATCATGGAGGGCAAACTCATGCGTCTCAAGGAAGAGAGGGACAATGTCTCCAAGGCTAAGGAGGCTCTGGAGCTCGCTGAACCAGGCAACCTGAGCCCAACAGAGGAACGCATGCAGGTGGCCATAGAAGAGTTGCAGGACCTGAAGGGAGTGTGGTCTGAATTGGCCACCACATGGGAGCAGATCGATGAGCTCAAGGACAAGCCATGGCTCTCTGTCCAGCCAAGGAAGCTCCGTCAGTCCATTGACACCCTGCTGAACCAACTGAAGGAGATGCCCTCTCGCTTGAGGCAGTACGGCTCCTACGAGCACGTCAAGAAGACCCTGCAGGGTTATGCCAAGGTCAACATGCTGGTCGTTGACATGAAGTCCGATGCCGTCAAGGAGCGCCATTGGAAGACGCTGATGAAGAGGTTGCACGTGAGCTGGGTCCTCTCTGAACTGACCCTCGGCCAAGTGTGGGATGTGGATCTGCAGAAGAATGAGTCCGTCGTCCGAGATGTGCTCGCTGTGGCCCAAGGAGAGAAGGCTCTCGAAGAGTTCTTGAAGCAGGTGAGCGAAGTTTGGCGCAGCTACGAGCTGGACCTGGTCAACTACCAGAACAAGTGCCGCCTGATCAAGGGCTGGGACGACCTCTTCAACAAGGTCAAGGAGCACATCAACAGTGTGGCCGCCATGAAGCTCTCTCCTTACTACAAGGAGTTTGAAGAGGATGCTCTGTCCTGGGAGGACAAACTGAACCGCATCAATGCCCTGTTTGATGTGTGGATTGATGTCCAGCGTCGCTGGGTCTACCTGGAGGGAATCTTCACTGGGAGTGCTGACATCAAGCACCTGCTGCCCATCGAGACCTCTCGCTTCCAGAGCGTCAGCACAGAGTTCCTCGGCCTCATGAAGAAGGTGAACAAATCACCGCTTGTCATGGATGTGGTGAACGTACCGAACGTCCAGCGCCAACTCGAGAGGCTCGCTGACCTTCTGACCAAGATCCAGAAGGCTCTCGGAGAATACCTTGAGCGAGAGAGAGCGTCCTTCCCTCGATTCTACTTCGTGGGTGACGAAGATCTGCTGGAGATCATCGGAAACAGCAAGAACATCAGCCGACTGCAGAAGCACTTCAAGAAGATGTTCGCAGGAGTGGCGTCTATCCTGGTGGATGACGAACAGACTCAGATCAGTGGAATCTCCTCCAAAGAGGGTGAAGAGGTCCTGTACAAGAACATCATTCTGCTCAAGGATCGCAAGATCAACGAGTGGCTTACGATGGTCGAGAAGGAGATGAGATTCACTCTCGCAACTCTCTTAGCTGAAGCCGTGCACGGAATCGCAGAATTCAAGTCGACTTCCATCGACCAAGCCAACTACATGAAGTGGGTGGACAGCTACCAAGCTCAGCTGGTCGTCCTGGCAGCTCAGATCGCTTGGTCAGAACACAGCGACGCAGCTCTCACGACAATCGAGAAGACTGGAGGGACGGATCTCAAGCCCATGGAGGACGTTCTCCAAACTGTGGAGTCCACTCTGACAGTCCTCGCTGACTCTGTGTTGCAGGAACAGCCTCCTGTGAGAAGGAAGAAGTTGGAGCATTTGATCACCGAGCTTGTGCATCAACGTGACGTGACTCGTGAGCTGCTGAAGAACAAGGTTGCGAGCCCCAAGTCCTTCGACTGGCTCTGTCAGATGAGGTTCTACTTCGACCCCAAGAACCCCGACCCCTTGAAGCAGCTGAGCATCCAGATGGCCAATGCCAAGTTCAACTACGGCTTCGAGTACCTCGGAGTGCAAGAGAAACTTGTCCAGACTCCTCTGACCGATCGATGCTACTTGACCATGACTCAAGCGCTGGAGGCGCGTCTTGGAGGATCTCCATTCGGACCTGCTGGAACTGGAAAGACTGAATCCGTGAAGGCCTTGGGTCACCAGCTTGGACGCTTTGTGCTTGTGTTCAACTGTGATGAAACCTTTGACTTCCAGGCGATGGGCAGAATCTTCGTTGGTCTGTGCCAAGTGGGAGCATGGGGTTGCTTTGACGAGTTCAATCGTCTTGAGGAGAGGATGTTGTCCGCTGTCTCCCAGCAGATCCAGACCATCCAGGAAGCCCTCAAGGAGCAGGCCATGCCGGGCAAGGACAAGGATAAATCCCAAACGCTGACAGTGGAGTTGGTCGGCAAGCAAGTGCGTGTCAACCAAGACATGGCCATCTTCATCACCATGAACCCTGGCTACGCTGGACGTTCCAACCTGCCAGACAACTTGAAGAAACTCTTCAGATCCCTGGCCATGACAAAGCCAAACAGGCAGCTCATTGCCCAGGTCATGTTGTACTCTCAAGGGTTCAGGTCGGCCGAGAAGTTGGCTAGCAAGATCGTGCCCTTCTTCCAGCTTTGCGATGAACAGCTGTCGCCCCAGTCTCACTACGACTTTGGTCTGAGAGCTCTGAAGAGCGTGCTGGTCAGTGCTGGAAACGTGAAGAGAGAACGCATCCAGAGGATCAAGGAAGGAAACCTGGAGAGAGGAGAACCAGTGGACGAAGGAGCCATTGCTGAGCAATTGCCTGAACAAGAGATTCTGATCCAGAGCGTCATGGAGACCGTCGTCCCCAAGTTGGTGGCAGAGGACATTCCTCTCCTGAACAGCCTGTTGTCTGATGTGTTCCCAGGAGTGGCATACACCCCTGCACAAATGACGGCCCTCAAGGAAATGATCAAGAGCATCTGCTCTGAGATGTATCTGGTCTATGGTGACGGAGAGGAACAGGGAACAGCCTGGGTTGAGAAGGTCCTCCAACTGTACCAAATCTCTCAGATCCATCACGGTCTCATGATGGTTGGCCCAAGTGGATCTGGCAAGAGCACCGCCTGGAGGGTCCTCCTGAAAGCTCTGGAGAGGCTGGAAGGTGTCGAGGGAGTCGCCCACGTCATCGATCCTAAGTCCATCTCCAAGGATGCCCTGTACGGAAGCATGGATCCCAACACCAGAGAATGGAGCGATGGTCTCTTCACCCACATCCTCAGAAAGATCATTGACAATGTCAGAGGCGAGATCAACAAGAGGCAGTGGATCATCTTTGACGGAGATGTGGATCCTGAATGGGTCGAGAATCTCAACTCTCTGCTGGACGACAACAAACTGCTCACTCTGCCCAACGGAGAGCGTCTGGCTGCTCCTCCAAATGTGAGAATCATGTTTGAGGTACAAGACCTGCGATACGCCACCCTGGCCACCGTCAGTCGTTGTGGTATGGTTTGGTTCAGTGAGGATGTCCTGACCACAGAGATGATCTTCGAGAATTATCTCTCAAGACTGAGAAACATCCCAGTTGAAGATGCTGAGGAGATCGACTTCCAGCCAAGGACTGCTGCTGAAGAAGAAGAGATCTCTCCTTCTCTTCAGGTCCAAAGAGATGCGGCTGCTATCCTGCAGCCCTACTTCGCCCCCGATGGCTTGATCAGCAAGTGCTTGGAGTATGCTGTTGACTTGGATCACATCATGGACTTCACGAGACTACGAGCTCTTGGCTCTCTCTTCTCCATGACCAACCAGGTGGTCAGAAATGTCATCAACTACAACAGCGCACATGCCGACTTCCCCATGCAGCAAGACCAACTGGAGAAGTACCTGCCCAAGGCCCTTGTCTACGCCTTGCTCTGGTCTCTGGCTGGTGACAGTCGTCTCAAAGTGCGCCAAGAACTTGGTGACTACATCCGCAAAGTGACCACTGTTCCTCTGCCTCCTCCCAACATACCCATCATCGATTACGAGGTGAGCATTGGCGGAGAATGGGTGCCCTGGACTTCAAAGGTTCCTGTGGTAGAAGTCGAAACTCACAAGGTGGCGTCACCTGATGTTGTCATTCCGACAATCGACACTGTTCGTCACGAGGCGCTCTTGTACACTTGGTTGGCTGAGCACAAACCTCTCGTACTGTGCGGTCCTCCTGGTTCCGGAAAGACCATGACTCTCTTCAGCGCTTTGAGAGCTCTGCCAGACTTGGAGGTCATCGGCCTCAACTTCTCCAGTGCCACAACCCCAGAGTTGCTGCTGAAGACCTTCGACCACTACTGCGAGTACCGCAAGACTCCCAATGGCCTGGTCCTGACTCCCATCCAACTCAACAAGTGGATCGTCCTCTTCTGTGACGAGATCAACTTGCCAGAGATGGACAACTATGGCACCCAGCGTGTCATCTCCTTCCTGCGCCAGATGGTTGAGCACGGAGGATTCTTCCGAGCATCCGACCAGAGTTGGGTGAAATTTGAGAGGATCCAGTTCGTCGGAGCTTGTAACCCTCCCACAGATCCCGGCAGAAAGCCATTGTCCCACAGATTCTTGCGCCACGTCCCTGTGGTGTATGTGGACTACCCAGGAAAGTTGTCCCTGACTCAGATCTACGGAACGTTCAACAGAGCCATGCTGAGGCTCATCCCAACACTGAGGACGTACGCTGAACCCCTGACCAACGCCATGGTGGAGTTCTATCTGATGTCTCAGGAGAGATTCACCCAAGACATGCAGCCCCACTACATCTACAGTCCTCGTGAAATGACCCGCTGGGTCCGAGGTATCTGTGAAGCTCTGCGACCTCTGGAGTCTCTGCCTGTTGAAGGACTGGTGCGCATCTGGGCTCATGAGGCTCTCAGGCTCTTCCATGACAGGCTTGTCGATGATGAGGAGCGCAGATGGACCAATGAGAACGTGGACAGCGTAGGACTGAAGCACTTCCCCAACATCGACAAGGAAGTGGCTCTCCATCGTCCCATCCTGTACAGTTGCTGGTTGAGCAAGGATTACGTGCCTGTGGAGCAGGAGGATCTGAGGGACTACGTCAAGGCTAGGCTGAAGGTCTTCTACGAAGAAGAACTCGACGTGCCTCTCGTCCTGTTTAACGAAGTCTTGGACCACGTGCTGCGAATTGACAGGATCTTCCGTCAACCTCAAGGTCACTTGCTCCTGATTGGAGTCAGTGGAGCAGGAAAGACAACACTCTCTCGCTTTGTGGCTTGGATGAACGGCCTCAGTGTGTTCCAGGTGAAGGTCCACAACAAGTACACTGCCGCTGACTTCGATGAGGATCTCCGAGGCGTCCTCAGACGTTCTGGATGCAGAGGAGAGAAGATCTGCTTCATCATGGACGAAGCCAACGTCTTGGACAGCAGCTTCCTGGAGAGGATGAACACTCTGCTTGCCAACGGAGAAGTCCCCGGTCTGTTCGAAGGAGACGAGTTGACGACCCTGATGACCCAGTGCAAGGAAGGAGCACAGAGAGAAGGAAACATGTTGGATACCCAAGAAGAGCTGTACAAGTGGTTCACTCAACAAGTCATGAGGAATCTCCACGTCGTCTTCACCATGAATCCTTCGTCCGAGGGATTGAAGGACAGGGCTTCCACTTCTCCTGCCTTGTTCAACAGATGTGTGTTGAACTGGTTCGGAGACTGGAGCAATGGAGCTCTGTACCAAGTTGGAAAGGAGTTCACCAACAAGATCGATCTGGAGAAGAACAACTATGTTGCACCAGAGTACCTACCAGTTGCCTACGAGGGTTTGTCCATGCCTCCAAGCCACAGAGAAGCTGTCATCAATGCCTTTGTGTATGTACATCAGACTCTGCATCAAGCTAACACTCGCGTAGCTCGCAGAGGAGGAAGGACCATGGCCATCACTCCCAGACATTACCTGGACTTCATCAATCACTATGTGAAACTGTTCAATGAGAAGAGAGCAGATCTGGAGGAGCAACAACTCCATCTGAACGTGGGTCTGCAGAAGATCCGAGAGACCGTGGACCAAGTTGAGGATCTGCAGAAGTCTCTGTCGATCAAGAGGAACGAACTGGAGGAGAAGAACATTGCGGCCAACGCCAAACTGAAGCAGATGGTGAAGGACCAGCAGGAGGCCGAGAGGAAGAAGGTTGCCAGTTTGGACATCCAACAAGCCCTTGACGCCCAGGAGACGGTCATCAAGGAGAAGCAGGAGAGTGTGATGGTGGATCTGGCCCAGGTGGAGCCGGCTGTCCAAGAAGCCCAACAAGCTGTGAAGGGCATCAAGAGGCAGCACTTGGTGGAGGTCAGGTCGATGGCCAACCCACCCAGTCACGTCAAACTCGCCATGGAGTCCATCTGCGTCATGTTAGGGGAGGATGCTCAAGACTGGAAGGCCATCAGAGGCATCATCATCAAAGATAGTTTCATCCCGACTATCGTCAACTACAACACGGAAGCCATCCCCGATGAGATCAGAAACAAGATGAAGAGCAAATTCTTGGACAACCCTGACTACAACTTCGAGAAGGTCAACCGTGCCTCCCTCGCTTGTGGTCCTATGGTCAAATGGGCCATTGCTCAGATCAACTATGCTGACATGTTGAAGCGCATTGAGCCATTGAGGAATGAGTTGAAATCTCTCGAGAATGCCGCAAGTGATAACCGCACGAAGAATGATGAGATGCAGAGCGTCATCCAGGAGCTGGAGAAATCCATCGCCAAGTACAAGGAGGAGTATGCCGTCCTCATCTCACAGGCCCAGGCCATCAAGGCTGATCTGGCTGCCGTCGAGGCTAAGGTTGAAAGGAGTGTTGCTCTGCTCAACAGTCTCGGCCAAGAGAGAGACAGATGGGAGGCGAGCAGCGAGACATTCAAGAGCCAGATGTCCACGATTATCGGAGATGTCCTACTCTCTACAGCCTTCATGGCTTACGCTGGTTACTTCGACCAGCACTGGAGGCAGAATCTGTTCACCAACTGGTGCAACCATCTGCAACAAGCCAGCATTCAGTTCAGGACTGACATTGCTAGGGTTGAGTACCTATCCAATGCGGATGAACGCTTGAGATGGCAAGCCAATGCCCTTCCAGCTGATGATCTGTGCGTAGAAAACGCCATTATGCTGAAGAGGTTCAACAGGTATCCTCTGATCATCGATCCATCTGGCCAAGCCACGGACTTCATCATGAACGAGTACAGGGACAAGAAGATCAACAAGACTAGTTTCTTGGACGACTCCTTCCGCAAGAACTTGGAGAGTGCACTTCGTTTCGGCAACCCACTCTTGGTGCAGGACGTCGAGAACTATGATCCAATCCTGAACCCCGTGCTGAACAGAGAACTGAGACGTACAGGCGGACGTGTGTTGATCACTTTGGGAGACCAGGACATCGATCTGTCCCCGTCATTCACGATCTTCCTATCCACTCGTGATCCTACCGTCGAATTCCCGCCCGACATCTGCTCCCGTGTGACCTTCGTCAACTTCACGGTGACTCGCAGCAGTCTTCAGAGTCAGTGCTTGAACCAGGTGCTCAAGTCCGAGAGGCCCGATGTGGATAAGAAGAGGTCCGATCTCCTCAAATTGCAAGGTGAATTCCACTTGAGACTGCGACAACTTGAGAAATCTCTGCTGTCTTCTCTGAATGAAGCCAAAGGAAAGATCTTGGATGACGACAGCATCATCTCTCACTTGGAAACTTTGAAGAAGGAAGCGGCAGAAATTGCCAAGAAGGTTGAAGAGACAGACATTGTGATGGAGGAAGTGGAGACAGTGTCTCACCAGTACATCCCTCTGGCCCAGTCTTGCAGCTCCATCTACTTCACACTGGAGTCCCTCAACCAAGTGCACTTCTTGTACCAGTTCTCCCTTCACTTCTTCCTGGACATCTTCACGGCCGTCTTGATGGAGAACCCTCATCTCAAAGATGTCAAGGACTACAACCAACGTCTCTCTGTGATCACTTCCGACCTTTTCAAGGTCACATACAACAGAGTATCTCGCGGCATGCTGCATCAGGACAGGATCACTTTTGCCATTCTCTTGGCAAGAATCCATCTCCGCCAGTCCGCCAACACCACAGAAGACACTCCCCCTACTCCACAAGAACTCAGCGAGATCACATACGACTTGGAATTCCAGCACTTCTTGCGAGGCCAGGAGAGTATGATCATCGATGTTCCAAACATCGACATCCCAGGCCTTGGTCCTCAACAGCTTATGGCTGTCAAGAGACTGACCACGTTGCCAGCATTTGCCAACCTCGTTAGGAAGACCCAGGGCAATGTTGACTTCCAAGCGTGGTTGAACACAGCCTCTCCTGAGGTGGACATCCCAGTCGTGTGGGATGAGACCAAACAACTCTCTTCCGTCGGACAGTCCATGTATCGTCTGTTGATGATCCAAACCTTCAGACCTGATCGCCTCATAGCTTGTTGCAACCTGTTTGTCCAGTCTGTGCTTGGTAGCACCTTCACTCACGATGCTGAACAAGAATTGGACCTCGGAAAGATCGTAGAGGAAGAAGTGAAAGCAAACTGCCCCGTCCTCATGTGCTCCGTGCCCGGCTTTGATGCCAGCGGACGTGTGGATGACCTCGCAACTGAGCTGAACAAGCCAATCACTTCTATTGCTATTGGATCTGCTGAGGGCTTCAGCCAAGCAGACAAAGCGATCAACTCTGCCACCAAGTCCGGTCGATGGGTGATGCTGAAGAATGTGCACCTTGCTCCTCAGTGGCTCGTGCAGCTGGAGAAGAAGATCCACTCTCTGACACCCCACGCCAACTTCAGACTCTTCCTTACTATGGAAATCAACCCCAAGCTCCCTACCAACCTCCTGCGTGCTGGCCGAGTGTTTGTGTTTGAACCTCCTCCAGGAGTCAGGGCTAACTTGCAGAGGACATTCAGCACTGTGCCTGCTGCTAGGATGTGCAAGGCACCTAATGAGAGAGCTCGCCTGTACTTCCTCCTGGCCTGGTTCCACGCAATTGTCCAAGAACGTCTTCGTTACGCTCCTCTTGGTTGGTCCAAGAAGTACGAGTTCAATGAATCCGACTTGAGATGCGCTTGCGACACTCTAGATACCTGGATTGATGCTGTAGCTATGGGTCGCACAAATCTGCCCCCAGAGAAAGTTCCATGGGACGCCCTTCGTGCTCTCATGTCCCAGTGCATCTACGGAGGAAAGATCGACAATGAATTTGACCAGAGGCTTCTGACAATGTTTGTCAACAAGGTTTTCACCAAGAAGACATTCGAGCAAGACTTTGTCCTAGTGCCTAATATGGACGGCTCCAAGAGCAAGAACTTGACTATGCCTGATGGAATCAGAAGAGAACAGTTCATCCAGTGGACTGATGGACTGCCGGACACCCAAAGCCCCACTTGGATGGGATTGCCCAACAATGCTGAGAAAGTGCTGCTGACCAACCAAGCCAATGAGATGATCGGCAAACTGTTGAAGATGCAACTTCTGGAGGACGACGATGAATTGGCTTACGATCAAAAGGACAAGGACAAAGACAAGGACAAGAGGGAGGCCGATGGACGTCCTTCTTGGATGAGGACTCTCAACAACTCCCTGGCCACTTGGATGCAGCTTGTGCCTAAGTCTGTTCCTGCCTTGCGTCGTACTGTCGAGAACATCAAAGATCCTCTGTTCCGATTCTTCGAGCGTGAAGTGAATGCCGGAGCTCGTCTGCTGAGCGATGTGAGAAGAGACATGTTGGAGATCATGCAGATCTGCCAAGGAGAGAAGAAACCGACCAACCACCATCGCCAGCTCATTTCTCAGCTTGCTAAAGGAATAATCCCCAAGTCGTGGTGTCGCTACACAGTCCCAGCCGGTCTGACGGTCATCCAGTGGATCACTGACTTCAGCCAGAGGATCCAGCAATTGCAATCCGTGTCCCAGGCCTCTCAGCAAGGAGGAACCACTGCTTTGAAGGATATGAACCTCCATGTGTGGCTTGGTGGCTTATTCATCCCTGAGGCCTACATCACCGCAACAAGGCAGTACGTTGCCCAAGCTAACAGCTGGTCCCTGGAGGAACTTCATCTGGATGTGCAAGTGATTGATGATCCCAAGAAATTCAAGTTGGATGACTGTAGTTTTGGTGTAACTGGCCTGCGTCTGCAAGGAGCGGTGTGCAAGAACAACACCCTCGAACTCTCACAAACCATCTTCTCGGACCTGCCTCTGACCATCCTCAGATGGATTAGGTTGGATTCAGTGAAGCCCTCAGTGAACAGAGTGACCCTTCCAGTGTACCTGAACGCGACCAGAGCCCAACTTCTGTTCACGGTCGACTTCGAGACCAGAGGAGAGGGATCCGGAAAGGACCACAGCTTCTACGAGAGAGGAGTCGGAATGATAAGTTCTATTGTCGGAACATAGATCAAACGAACGGAAGATTATTGCCTCAGTTACATTAACTACATTGTGCGAAATTGATATGACTTCTAATACCACTTCTAGCTGTATGTCTTAACTAATTATTGAATTATTTCATTGATTAATAATCTCCGCTACAAGCTTATGTATTGTATTCGATCGACAGGGTGGGCGTTTTAATGTAATTTGCATTTAACATTTAAATAACATATTTAAATATGATTAATTGATTATATATTATTACTCTTGTTATGCATCATGTTTGGCTTTATGGCTATTAACAATTATTAACAATTATATACAATTATATATATTATATTTAGCGAAGATTTCTGTACATGTATCTATCTAGTACTATCTACTGGTCAGGTTATTAGAGCCAACACGTGTGAACATTGTAGCGCTATCTGCACGTCGCTTCTAATTGCATCTATAATTGCATCTATAAAATCTATAAAGTAGAGCGCAGGCTTCTCAATGTTGCTTTTAGCTGATGCTGTATTTCTTCAGCAAACAGGTGATAATGCCTGGCAAAAGGATATTACCAGGGTATATGTAATTTAATGATCTATATTATATCGTAGGACGTCTTTGGGTACAGTTACACAATTAGCTTCAGTCTGAATTACGTCGTAATTAGCACGTTATCAGATAAGGTCTGAGGTAATTAATTGGACTTCTGATAAGCTCTTGTTTAATTTGGTGCAAGGATCATCGGATCCTTCACTTGTAATTGATATGTATAATTAATGGTGATTATGAAATGCGCAAGTTTATTGTAACTGAGGACCAATAAAATCTCCTAATTAATAATAATAAAAAAAAAA

Protein: 4664 aa

>Dynein Heavy Chain 1_protein

MADNSDTEAAQGQGSASQALMDQSVLGNYIRRIVPVNLEDSDDTPAALVASLKDKATVDCMKKFISDPQVQCLLIQRQTIKDESVDVEGDGEAVNYFITPEVLYINSKMNSLCLMKKGPLLEADKSCSSQIRLMTLSEGSPYETLHAFVSNAMAPFFKSYVRATGKADRDGDKMAPSVEKKIAELEMGLLHLQQNIDIPEITLTIHPTIAQYIKKCGEQSKKPTVADFDEKKEDAGFLNTLQNGVGRWIRELQKVTKLDRDPASGTALQEISFWLNLERALLRIQEKRESPEITLTLDILKQGKRFHAVVSFDTDTGLNKAIEMVNDYNPLMKDFPLNDLLSATELDRIRVALQAIFTHLRKIRSTKYPIQRALRLVEAISRDLSQQLLKVLGTRRLMHIPFDEFEKVMGACFEVFGTWDDEYEKLQGLLRDLVKKKREEHLKMVWRVNPAHKKLQARLDQMRKFRRQHEQLRSVIMRVLRPAAPRPGSPTPEETGGESKNDGVALDAADANAIEEVNLAYENVKEVDCLDVSKEGTDAWEAATKRYDERIDRVETRITARLRDQLGTAKNANEMFRIFSRFNALFVRPHIRGAIREYQTQLIQRVKDDIESLHEKFKVQYPQSKACRMSQVRDLPPVSGSIIWARQIERQLNAYMRRVEDVLGKGWENHVEGQKLKSDGDSFKIKLNTQDLFEDWSRKVQQKNLGVSGRIFAIESVRSRQVGGKGSVLRLKVNFLPEVITLSKEVRNLKWLGFRVPLTIVNKAYQASQLYPFAISLIESVRTYDRTCEKVDERPSISLLVAGMRKDVQGLLTEGIHLVWESYKLDPYVQRLAESVFNLQEKVDDLLVIEEQIDMDVKNLETCQYSFTTFNEHLAHIQKNVDDLSLRQYSNLPQWVAKLDQEVEKKLAFRLECGLEAWTKALLETEEEQIDNSMDTDAPKTPKHKPGGEPIMKTLHHELHITNQVMYINPSLEEARFKTIQELFAWEQVILTLPRIQHSRYQVGLDVESEAATHYKNLLNKLPQHGNKLKDAYKAVEEVLQKASEYVDMWMRYQALWDLQSELLYGRLGESNIQQWMRTLEEIKRVRKTFDTSETKQVFGPLHVDFAKVQAKVNLKYDSWHKDVLSKFGNLLGGKMQDFHNSVSRSRGELEQQSIETANTGEAVSFITYVQSLKRKMKNWEKEVEMYKEGQRILERQRFQFPSNWLYSDNVDGEWGAFNDIIRRKDSSIQSQVASLQMKIVSEDKVVETKTHDLLGEWEKSKPVEGQMRPDNAIKALTIMEGKLMRLKEERDNVSKAKEALELAEPGNLSPTEERMQVAIEELQDLKGVWSELATTWEQIDELKDKPWLSVQPRKLRQSIDTLLNQLKEMPSRLRQYGSYEHVKKTLQGYAKVNMLVVDMKSDAVKERHWKTLMKRLHVSWVLSELTLGQVWDVDLQKNESVVRDVLAVAQGEKALEEFLKQVSEVWRSYELDLVNYQNKCRLIKGWDDLFNKVKEHINSVAAMKLSPYYKEFEEDALSWEDKLNRINALFDVWIDVQRRWVYLEGIFTGSADIKHLLPIETSRFQSVSTEFLGLMKKVNKSPLVMDVVNVPNVQRQLERLADLLTKIQKALGEYLERERASFPRFYFVGDEDLLEIIGNSKNISRLQKHFKKMFAGVASILVDDEQTQISGISSKEGEEVLYKNIILLKDRKINEWLTMVEKEMRFTLATLLAEAVHGIAEFKSTSIDQANYMKWVDSYQAQLVVLAAQIAWSEHSDAALTTIEKTGGTDLKPMEDVLQTVESTLTVLADSVLQEQPPVRRKKLEHLITELVHQRDVTRELLKNKVASPKSFDWLCQMRFYFDPKNPDPLKQLSIQMANAKFNYGFEYLGVQEKLVQTPLTDRCYLTMTQALEARLGGSPFGPAGTGKTESVKALGHQLGRFVLVFNCDETFDFQAMGRIFVGLCQVGAWGCFDEFNRLEERMLSAVSQQIQTIQEALKEQAMPGKDKDKSQTLTVELVGKQVRVNQDMAIFITMNPGYAGRSNLPDNLKKLFRSLAMTKPNRQLIAQVMLYSQGFRSAEKLASKIVPFFQLCDEQLSPQSHYDFGLRALKSVLVSAGNVKRERIQRIKEGNLERGEPVDEGAIAEQLPEQEILIQSVMETVVPKLVAEDIPLLNSLLSDVFPGVAYTPAQMTALKEMIKSICSEMYLVYGDGEEQGTAWVEKVLQLYQISQIHHGLMMVGPSGSGKSTAWRVLLKALERLEGVEGVAHVIDPKSISKDALYGSMDPNTREWSDGLFTHILRKIIDNVRGEINKRQWIIFDGDVDPEWVENLNSLLDDNKLLTLPNGERLAAPPNVRIMFEVQDLRYATLATVSRCGMVWFSEDVLTTEMIFENYLSRLRNIPVEDAEEIDFQPRTAAEEEEISPSLQVQRDAAAILQPYFAPDGLISKCLEYAVDLDHIMDFTRLRALGSLFSMTNQVVRNVINYNSAHADFPMQQDQLEKYLPKALVYALLWSLAGDSRLKVRQELGDYIRKVTTVPLPPPNIPIIDYEVSIGGEWVPWTSKVPVVEVETHKVASPDVVIPTIDTVRHEALLYTWLAEHKPLVLCGPPGSGKTMTLFSALRALPDLEVIGLNFSSATTPELLLKTFDHYCEYRKTPNGLVLTPIQLNKWIVLFCDEINLPEMDNYGTQRVISFLRQMVEHGGFFRASDQSWVKFERIQFVGACNPPTDPGRKPLSHRFLRHVPVVYVDYPGKLSLTQIYGTFNRAMLRLIPTLRTYAEPLTNAMVEFYLMSQERFTQDMQPHYIYSPREMTRWVRGICEALRPLESLPVEGLVRIWAHEALRLFHDRLVDDEERRWTNENVDSVGLKHFPNIDKEVALHRPILYSCWLSKDYVPVEQEDLRDYVKARLKVFYEEELDVPLVLFNEVLDHVLRIDRIFRQPQGHLLLIGVSGAGKTTLSRFVAWMNGLSVFQVKVHNKYTAADFDEDLRGVLRRSGCRGEKICFIMDEANVLDSSFLERMNTLLANGEVPGLFEGDELTTLMTQCKEGAQREGNMLDTQEELYKWFTQQVMRNLHVVFTMNPSSEGLKDRASTSPALFNRCVLNWFGDWSNGALYQVGKEFTNKIDLEKNNYVAPEYLPVAYEGLSMPPSHREAVINAFVYVHQTLHQANTRVARRGGRTMAITPRHYLDFINHYVKLFNEKRADLEEQQLHLNVGLQKIRETVDQVEDLQKSLSIKRNELEEKNIAANAKLKQMVKDQQEAERKKVASLDIQQALDAQETVIKEKQESVMVDLAQVEPAVQEAQQAVKGIKRQHLVEVRSMANPPSHVKLAMESICVMLGEDAQDWKAIRGIIIKDSFIPTIVNYNTEAIPDEIRNKMKSKFLDNPDYNFEKVNRASLACGPMVKWAIAQINYADMLKRIEPLRNELKSLENAASDNRTKNDEMQSVIQELEKSIAKYKEEYAVLISQAQAIKADLAAVEAKVERSVALLNSLGQERDRWEASSETFKSQMSTIIGDVLLSTAFMAYAGYFDQHWRQNLFTNWCNHLQQASIQFRTDIARVEYLSNADERLRWQANALPADDLCVENAIMLKRFNRYPLIIDPSGQATDFIMNEYRDKKINKTSFLDDSFRKNLESALRFGNPLLVQDVENYDPILNPVLNRELRRTGGRVLITLGDQDIDLSPSFTIFLSTRDPTVEFPPDICSRVTFVNFTVTRSSLQSQCLNQVLKSERPDVDKKRSDLLKLQGEFHLRLRQLEKSLLSSLNEAKGKILDDDSIISHLETLKKEAAEIAKKVEETDIVMEEVETVSHQYIPLAQSCSSIYFTLESLNQVHFLYQFSLHFFLDIFTAVLMENPHLKDVKDYNQRLSVITSDLFKVTYNRVSRGMLHQDRITFAILLARIHLRQSANTTEDTPPTPQELSEITYDLEFQHFLRGQESMIIDVPNIDIPGLGPQQLMAVKRLTTLPAFANLVRKTQGNVDFQAWLNTASPEVDIPVVWDETKQLSSVGQSMYRLLMIQTFRPDRLIACCNLFVQSVLGSTFTHDAEQELDLGKIVEEEVKANCPVLMCSVPGFDASGRVDDLATELNKPITSIAIGSAEGFSQADKAINSATKSGRWVMLKNVHLAPQWLVQLEKKIHSLTPHANFRLFLTMEINPKLPTNLLRAGRVFVFEPPPGVRANLQRTFSTVPAARMCKAPNERARLYFLLAWFHAIVQERLRYAPLGWSKKYEFNESDLRCACDTLDTWIDAVAMGRTNLPPEKVPWDALRALMSQCIYGGKIDNEFDQRLLTMFVNKVFTKKTFEQDFVLVPNMDGSKSKNLTMPDGIRREQFIQWTDGLPDTQSPTWMGLPNNAEKVLLTNQANEMIGKLLKMQLLEDDDELAYDQKDKDKDKDKREADGRPSWMRTLNNSLATWMQLVPKSVPALRRTVENIKDPLFRFFEREVNAGARLLSDVRRDMLEIMQICQGEKKPTNHHRQLISQLAKGIIPKSWCRYTVPAGLTVIQWITDFSQRIQQLQSVSQASQQGGTTALKDMNLHVWLGGLFIPEAYITATRQYVAQANSWSLEELHLDVQVIDDPKKFKLDDCSFGVTGLRLQGAVCKNNTLELSQTIFSDLPLTILRWIRLDSVKPSVNRVTLPVYLNATRAQLLFTVDFETRGEGSGKDHSFYERGVGMISSIVGT

Domains

DHC_N1: 234 – 830

Dynein_Heavy: 3934 - 4658

Clone

Partial ORF

Primers

Forward: GGGAAAGCTGACAGAGATGG

Reverse: GAGCTGATCCCTCAGACGAG

Nucleotide# 739 – 1944 = 1206 nucleotides

>Dynein Heavy Chain 1_clone

GGGAAAGCTGACAGAGATGGAGACAAGATGGCTCCCTCAGTGGAGAAGAAGATCGCGGAGTTGGAGATGGGTCTTTTGCATCTTCAGCAGAACATTGACATCCCGGAGATCACTCTGACCATCCACCCAACAATCGCTCAGTACATCAAGAAGTGTGGAGAGCAATCGAAAAAGCCCACGGTGGCCGATTTTGACGAGAAAAAGGAAGATGCCGGATTCTTGAACACACTCCAGAATGGAGTAGGAAGATGGATTCGAGAACTTCAGAAGGTCACTAAATTGGATCGTGACCCAGCATCAGGCACAGCCTTGCAGGAGATCAGTTTCTGGCTCAACTTGGAGAGAGCTCTGTTGAGAATCCAGGAAAAAAGGGAAAGTCCTGAAATCACTCTAACTTTAGATATTTTGAAGCAGGGAAAGAGATTCCACGCTGTTGTTAGTTTTGACACTGATACAGGTTTGAACAAAGCAATTGAAATGGTGAATGACTACAATCCTTTGATGAAGGACTTCCCTCTTAATGATTTACTCTCCGCTACTGAATTAGACAGAATCAGGGTTGCTCTTCAAGCCATTTTCACTCATTTGCGAAAGATCAGGAGCACAAAATACCCCATTCAAAGAGCCTTGCGTCTCGTGGAGGCCATATCTCGCGATTTGAGCCAACAGCTTTTGAAGGTACTCGGCACTCGTCGCTTGATGCATATTCCTTTTGATGAGTTTGAGAAAGTCATGGGAGCTTGCTTCGAAGTCTTCGGTACCTGGGATGATGAGTACGAGAAACTGCAAGGACTTCTGCGTGACTTGGTGAAGAAGAAGAGGGAGGAGCATCTCAAGATGGTCTGGAGGGTCAACCCCGCTCACAAGAAGTTGCAGGCCAGGCTCGATCAGATGAGAAAGTTCCGCAGACAGCATGAACAGCTGCGATCTGTCATCATGAGGGTTCTTCGCCCTGCTGCTCCTCGCCCTGGCAGCCCCACCCCAGAAGAGACCGGAGGTGAATCCAAGAATGACGGAGTGGCCCTGGATGCGGCTGATGCTAATGCCATTGAGGAGGTGAATCTTGCTTACGAAAATGTCAAGGAGGTTGACTGCTTGGATGTCTCTAAGGAAGGCACAGACGCTTGGGAAGCTGCTACAAAAAGGTACGATGAACGTATCGACCGAGTAGAAACAAGGATCACTGCTCGTCTGAGGGATCAGCTC

Cloning info

PCR rxn on 05/10/16 using GoTaq polymerase mix

| Template | Initial denature | Denature | Anneal | Extension | #cycles | Final extension |
| --- | --- | --- | --- | --- | --- | --- |
| 2 hr cDNA | 4min @ 94C | 30sec @ 94C | 30sec @ 55C | 2min 15 sec @ 72C | 35 | 10min @ 72C |

**CLIP-170**

Gene model: 7406 nucleotides

ORF: 268 – 4113 = 3846 nucleotides

>CLIP-170_full

GCCATCTCATGAGTATTACTCATCAGTTGTATTGCGCGTGCGCTGTGAGCATTAGTCATCAGCCATATTGGATTTCCTGTTTGAAGTGTCACAATAACAAAAGAAGAGAATCTCAAGTGACCAGGTGGAAATATGACCTGAATCAGACCGAGGAGCACCGTAGTTTAGCATAAAAATACCTTGAAGTGTCATTCAGGTGGCACAGATTTATGCTTGAAGACTGAAAGACACCAAAATTCTGGAAAAGAAGTAAAAAAAAAAGGAAAAATGCAGAAGCCGTCGGGGCTGAAAGCTCCGAGCAAAATAGGAAAACCTTCGGGGCTTCCTGCTCCGAGGACCGGTATTCCGACCCCAGCTTCAAGTAGGGCACAGAGTGCATTAGAAAAGAGCGCTCGTGCCGCAGGCCTGTCTGGTACCACAGCTGAAAAATATGCTGCCGATGTGGTTAAAGTTATTCCAGGTCCGGAGCCTGGAAATGGTGAAAATGCAGAAGATGATTTCATCATCGGAGACAGAGTGTGGGTCGGAGGAACGAAAGCGGGGTTCATTGCATATATTGGAGAGACTAAATTTGCCCCGGGAGAGTGGGCTGGGATAGTGCTTGATGAACCAGTTGGTAAAAATGACGGTAGTGTGGCGGGGGTGCGTTACTTCCAGTGCGAAGCAAAGAAGGGAGTGTTCGCTCGGCCGGCCAAAGTGAGTCGCCATCAAATCATGGACGATGGACTACCCCTCGCAGCCGAAGCGTCTTCAATGGTCACCCCTCGACCTGCTCCCAAAACAAACGGAACAACATCAGGGTTGACCAAGTTATCTCAGCCGACCCCTAAGGTTGGTCTGGGGGTCAGGAAGAGTATATCGGGCTCCTCTGCGAGCCTCAAGACCTCCCCGACTGGCTCTGTGTCCAACATGAACTCCCACGCCAATGCCGCTGGCGGCATGCAGGTCGGGGACAGGGTCCTGGTCAGTGGCACCAAGGCAGGGACATTGCGCTACTATGGCACGACTGCCTTTGCCAAGGGGGAGTGGGCTGGGGTAGAGCTTGATGACCCTCAAGGAAAGAACGACGGCTCTGTCGCTGGAAAAAGGTATTTTGACTGCCGGCCATTGTACGGCCTGTTTGCCCCCATTCACAAAGTGGCCAGACTCGGAGATGTTCCTCCGCCAGCCACGCCTAAGAGGGGGAGCGTTAGCAGCGTGGGGAGAGGATTGACGGGATTGAGCAACTTATCTCGGGAGAGAAGCGGCAGCCAGGATTCCATATCTTCCGTGTCCTCGTCTGCGTCGAGTATTTCTCGGAGCCGGGTAAGGCTGGGAACGAAGACCCTGAACACGAGCAGCTCAGCGTCCCCGGGCGCCTCTCAGGTATTGGCAGCATGGCTACCTCGCTCTTACCTTACACAAGTCAATATGCAGAAAGCGCTGAAGGAGAAGGAGGAGCACATCGAACAATTGCTGAAGGAGAGGGACATGGAGAGGTCAGAGGTCGCAAGGGCAGCCGCGCAGGTGGACGAGGCGGAAACGCTTCTTTCCAACCTGAAGGCTGAGCATGAAACTGCAATTGAAGAATTTGAAGCAAAAGTGGCCGAGTTATCGGAACAGATGGAAGTGTTGCAGAAGGAGAGGGATGAAGCCAAGGAGAAACTGGACGAAGAAGTCAGGAAAGTTGAAGACTTGCAGTTCTCTCTCGAAGAGGAGTCCATTAATAAGGATGACCTTGAAACGAAGACGGCCAGCGACCTTGAGAAGTTACAAGGACTGGAAGACCAGCTGAAGGAACAGAGGGACAGGGCGGACAAGTTGGAGAAGGAACAGCAGTCCTGGAAGCAATGTCTAGCGGAAGAGAAGGGACGTACCAAGGAGGCTGAAGATTCATCCGTTGCATACCTCGATCAGATCGAGGAGTTGACACACAAGTTGCACATGACGGAAGGGAAGCTCAAGGCATTCGAGGAGTCCAAAGCTTCTGATGGCCAGAAGGCCAGCCAGGTAGCAGAAGAACTTACCTCAAAATCGACCAAAATTTCTGAGCTTGAATATGAGGTATCGTCAAAGAAAAAGGAGTACAGTCAGCTTGAAAGCAAATTTGCAGAATTGCAAGAAGAATTAAAAGAATGCCAAACCAAGATATCCAAGGAGCAGGAGCAAATTAGTGAACTTGTCAAGAAACTCACAGCCAGTGATGAAGCTAACAACAACTTGACTGCCGAACTTCAAGATATGAAATCTAAATATGCAGATCTTCAGCGACAATTTGATGCCAGTGAAGAAAAGTCGTCGCAGTTGGAAGATGATAAGGGCAAACTGGAACACCAAATTTCGGAGCTGATGAAAAACTCTGGAGACAGCTCTCAGCAGTTGACCATGATGAACCAAGAAATGCTTGAGAAGGATAGAAAAATAGGGGAGGTGCAAGCAAGTTTGTCCGAAGCATTACAAAAGGTTTCAAGGGCAAATGAGCAGATGGAGAGGAGCCGGGAGGAGGGGGAGAGGGAGATGGAGGTCCTCCTTGGCAAACACGAGGAAGACCTCAAGTCCTTCCAGGAAAGACTCGACGAGACTCAATCGAAACTTGAAAAATCAACAGCTAAATTAGCGAAACTACAAACTGATTCAGAAGCAGAAATTACATCACTAACAACCAAAAAGGAAGGAGAAATAGCAGAATTAAAAACACAAATTCAATCACTTCAAGAACAGCTGAAGACCCATGAAGCATCAATTGAAGAGACCAAGACCAACATCCAGCAAGTCACCAGCGACAGAGACGAACTTAAGTTTGAAAAAGAGAAGTTAGAGAAGCAGATCAAGAAATTAGAAGGCGAGAGAGAAGAGGCGCAGAAAGAGAGAGATGAGGCCAAGACGGAGAAGCAGAAGGTGGAGGAGGAGAAGCTGCAGACCATGTCCAGCATGGGCTCCACGGAGGAGAGGATTGCTGAGCTGGTCAAGGACATCGAGAAGATACAGCAGGAAAAGAAACAGCTGATGGAAGAGAGATCGCAAGAGGCAAGTGCACGCGAGAAGTTGTCCTTGGAGAAAGACGTCCTCGCAAAAGAGAAGCAGGACATGTTGTTGAGCGTGCAGCAGCAGGAGGACAAGGAGATGGAGCTGTTGCAACAGATTGAAGCCATGGCCAAGGAGATGGAGGGCTACAAGGAGCAGTTGATGGAAAGTCAGAAGACGAGCCAGTCAGAGACCAACAACATGGCCATCCTCAAGCAGAGACTAGAGGCAAGTGAGAAGGTTTCAAGTGACTTAGAACAACGATTACAAGCCTCAGACTCCGAGAAGGTCAACCAACAGAAAGAGATAGAAAAATTAAAACAAGTTCACCAAGAAAAACAACAGTTGGAAACTCAGCATGCTGCCTTGAGGACTGAGATAGATGAACTGAAACGAAGGGAGAGTAAGATCGTGGATGACTATGAGGTAGACAAGACGTCTTTGCAGAATACTTTAGATACTTCGAACCTGATGTTGAAGCAGAAGGATGCCGAGATCGAACTGCTGACAAATGAGCTGAGAACTGCTCAGGGAGACTTGGAGAAGGCGAAGAACAAGCAGTCGTCAACATCGTCCTTGGAGAACGAGAAGAAACAGCTGGAGGAGAGGGTGCAACAGTTGGAGTTCAGTCTGGCTGAGATGGAGATTAACGCAAATGTCACAGAGAACACGGGCCAGAACACAGGGCAGAACACAGGGCAGACCACCGGCCAGGGAGACAAGATATTGACCAAGCTCAAGGAGGAGAAGACCGCTGCTGATGGCCAGATTGACTTCTTAAACTCAGTCATCGTAGACATGCAGCGCAAAAATCAGGAGTTGATGAGAAAAGTTGACATCCTGACGACAGGAGACGCCGAAGTGGTCAATGGCAATGGAGATATGGACTACTCTGAGAGTGCCCGACGGAAACTAGCCCCTCGGATGTTCTGCGACATTTGCGACATGTTCGACCTCCACGACACGGACGACTGCCCCACCCAGTGCATGTCCGAGAGCCCCCCTCCTTCACTGCACCACGGAGATCGCACGGCCACTCGACCCTACTGTGAAATCTGTGAAGTATTTGACCATTGGACGAATGAATGTGACGATGAACAGACGTTTTAGAACGGAACACGTTGCCATTTCTCCCCCAATTGTCAGTCAGACTCGGTCCCCATCCCCTGCTAAATACTCAGATTTTGAATGGTGTTTATGAATCGTAATAATGATTGTTGATGCTTAATTTACCATTGATGCCAAATTAGAATCGTGAAAGTAAATCTCTTGAATTATTTTAAGGTGAAATTGTAAAAGTTAGTTTGGTTATAAATGATTTTAATATGGGAGTACATGAATAAGAGTCGTGTGCATGATTAAACGTGTTTCTGTATCTGAATCTTAATTTGAATATTAATTTAGACATTGAATGATTTATCTGTTTTAATGGGATGATAATTATATTATTTGGTGCTAATTATGGTTTTTAATGCGGAGATAAAAAAATCATTATAAAATTGCTGTATTGAAGTATTCTGCAGAAAATCTTGATGATTGACGTTTTTGTCGGTGTAGGTCATTGAACCAAACGGGTGATCTTGTAACTTGTAAGTCTTGTGTGAGAAATACGCAACTGAGGCTGTTGTTTTAACGGGTTATCAATTGACGCTGTGGTCCTGGTTGTTGTTGATCTATCTTACTCTCTTGAATTGTGCGAAATGTGGTCTGTAGAGGAGTGAATTTTTACAAGCATAATCACAAATGCATATATATATATATGGCATTGAGTTGAAGAAAGGGCTTAATTTTGCATTAGGTTGAAAATTTTTACACCAATATCTAAGGCTGTACACCATGTATAGGTTGAATATTGGTTGTCATATTTGCGTCAATAAAATTTACTACTTCTGACTTTGAAAATAAATTTATTTTGTAGGTACTCTGTACAAGTTAATGAGAGTAGAAATTATAATACTATTATATGCTGCCTTCTTATGATGGAAAGACCACAGACGTGTAAAAAATTTGAATTGAAGTGTGGATATATATTTTTTTGGATGTAAATTCATGAGTGGTCATATGCCTTGAGTCATGATTGTAAAATGTTGCATCTCAGTGCTAAGAAATTGTATTATTATGAAATTGAGTGAAATGTTTATCATGATAGTGGAAGTGATGTAAGTTTCTAACCTGATTTGGTAACCTGGCGTTCCCAATGGTAGGAAAGAAAGTTGTGCTGAATGGCGTAACTGGGAATGTGAAAGTGAAGTACATTTTATGACGCGCTTCATGTGACATACTCGTGAGGGTTGTTGAATTTTCAGATTTTTAAGAAGAAGTCTCGTTGAGTGTAGTACTGGAAGGATTTGTTGTTGAATAACTGTGATATTTATTGTGTATTAACTAGTGTCTTAGCCTCTTAGGTAATTATGTCATACCCACGTGTACATATAATACATATGTCATATATTACCTATCTGTGGTTAACATATTGAAGTGACATTAGAATGGCTGGTATTGTCCATCTTTTTGTCAGTGCCCATGTTACAGATGAAGAACGAAAATATAATTGGAAATGATGTAGGCCTACAAGGACTTCAACGTAAATTAATAGTTAGCCGGATTCGGAACGTGTACACTGCATTTCGAGAGTGTTGTTCAGAACACAGTGTTCCTTTATTATCATTGTATAAGCTTCACAACATGTGCAAGTTTAGAAGTCAGACGTGTCATAATAATTATCAGCTTGTCTTTTATGGATATATTCTTTATGTAGCTGTGTAAATATACATTTAAACAAAACATATTTCAGAGCCTTTCAGCTGAAACTAGATAGTAAATTTGCCATGTTATTGTAAGGTTTTACATCCCTGTCATAAATGCATCGCTGGGTAAAGTTGCTACTTTTTAAGTGGGAATTGATACAATTAAACCAATTTAAGTGTTACTTGCATGTTTGGATGGTATATTTATATAATGAATCCTCAGAATATCCTCAGAATATGCATATGATGTCTTTGATGTATTTATACAATTGCTTGAGTTTCATGACAGTTCTGAGTTGAAGCATGCGCTCTTCCATTTAGTGAACGAGTTTTGTAAATTTCAAATATGTGGATATAATGGTAGAATTTGTAAATATTGCATTGTACAGCTTATATTGTATAATTGACGTCTCACTGAAAACTCTTTAGACTACCCTTGTGGTACTAAACGTGGTATAATACTGGTTGAATGGTAGGAAGTAAGTGAATGTGCTTGAAATAGTGGGAGTCACTTTTAAAATTCTAAATTTGTCCTCCTCTGCTCGTGCCTTTTATTCTTTTCCTATAAATATGACCTTTTCTGTATAATATTTAATATATTAATGTAATACATGTACTGCAATTATATTATGCCTACACTCTCCTCAAACTTAGGTTTGACATAAACAGTAAATTATTGGTTCATATTCTATATCATTTATACGCATTGTATTATGACGTGGTTATGCAAGCTTAACTTAAAATAAGTATCCGAACTTGAATTTTTGTTTGATTTACATTATTGAAAAATATCCTTAAAAACGTTTTGTATAGCTGAATTTTTCCGTGTTTTGAAAGACTTTCTCTAAACATGGAAAGACATTCTCTGAAAATTTTACAAAGATTTCTCAATGAAATACTGGAATATGGATGTTATATGAAAGACTGTCACATTTGTTTAGGAGATTAATCTGGAACTGAAGTGATTGAAAATGTCCTTTATTATAGTTTGGTTGATATTTTTATTATTGAAAAAATAAAATAAAAAGGTTGCTTTGATGAATATTATATATCATTATGGGAACATATCGATAGAAAAATATTTAGGAAATTGTACTGGAATGAATCTGAAAAAAAAAAAATGAAAAGGGGAAATCAAAGAAGCAAATCAATGTAACAACTTAGTATATTATATATGTATATTATTTAGACAAATTTATCTTGGGAGCTTTTTTCGATTTGTTTTAGCCCGTTATCAACATGATCAAAAATCATGGCAAATTGAGTTCTATAAATTTCCTTTGATTGGGTGCTACACGTTTCCATGGTTTCCACTGTGTAAAGTGATGTTTCAAGTAATGTCGATAAAAACAAGCACTCCACAATCATGAACCGCAATTTTATTGACAATTTGCATCAGACATCAAATTCTATCATGTGGTCTTTGCATGATTTCATGCAATCATGTGTTGTAAATATTGGGAGTTTACATAATGGTGTATTTTCTATTACAGTAAGAATGTAAGCTTAGTGAATATTATAAAACACCATGACTGATCATCTTGTTTTTCTTATATAACTTCTACCATCCAATAATGTTGAAATTTAATACTGGTTTTGAGAGTTTGATTTATAGAAAACTTTGTTGAATAAACTTGGATGTAAAAAGAAAAAAAA

Protein: 1281 aa

>CLIP-170_protein

MQKPSGLKAPSKIGKPSGLPAPRTGIPTPASSRAQSALEKSARAAGLSGTTAEKYAADVVKVIPGPEPGNGENAEDDFIIGDRVWVGGTKAGFIAYIGETKFAPGEWAGIVLDEPVGKNDGSVAGVRYFQCEAKKGVFARPAKVSRHQIMDDGLPLAAEASSMVTPRPAPKTNGTTSGLTKLSQPTPKVGLGVRKSISGSSASLKTSPTGSVSNMNSHANAAGGMQVGDRVLVSGTKAGTLRYYGTTAFAKGEWAGVELDDPQGKNDGSVAGKRYFDCRPLYGLFAPIHKVARLGDVPPPATPKRGSVSSVGRGLTGLSNLSRERSGSQDSISSVSSSASSISRSRVRLGTKTLNTSSSASPGASQVLAAWLPRSYLTQVNMQKALKEKEEHIEQLLKERDMERSEVARAAAQVDEAETLLSNLKAEHETAIEEFEAKVAELSEQMEVLQKERDEAKEKLDEEVRKVEDLQFSLEEESINKDDLETKTASDLEKLQGLEDQLKEQRDRADKLEKEQQSWKQCLAEEKGRTKEAEDSSVAYLDQIEELTHKLHMTEGKLKAFEESKASDGQKASQVAEELTSKSTKISELEYEVSSKKKEYSQLESKFAELQEELKECQTKISKEQEQISELVKKLTASDEANNNLTAELQDMKSKYADLQRQFDASEEKSSQLEDDKGKLEHQISELMKNSGDSSQQLTMMNQEMLEKDRKIGEVQASLSEALQKVSRANEQMERSREEGEREMEVLLGKHEEDLKSFQERLDETQSKLEKSTAKLAKLQTDSEAEITSLTTKKEGEIAELKTQIQSLQEQLKTHEASIEETKTNIQQVTSDRDELKFEKEKLEKQIKKLEGEREEAQKERDEAKTEKQKVEEEKLQTMSSMGSTEERIAELVKDIEKIQQEKKQLMEERSQEASAREKLSLEKDVLAKEKQDMLLSVQQQEDKEMELLQQIEAMAKEMEGYKEQLMESQKTSQSETNNMAILKQRLEASEKVSSDLEQRLQASDSEKVNQQKEIEKLKQVHQEKQQLETQHAALRTEIDELKRRESKIVDDYEVDKTSLQNTLDTSNLMLKQKDAEIELLTNELRTAQGDLEKAKNKQSSTSSLENEKKQLEERVQQLEFSLAEMEINANVTENTGQNTGQNTGQTTGQGDKILTKLKEEKTAADGQIDFLNSVIVDMQRKNQELMRKVDILTTGDAEVVNGNGDMDYSESARRKLAPRMFCDICDMFDLHDTDDCPTQCMSESPPPSLHHGDRTATRPYCEICEVFDHWTNECDDEQTF

Domains

CAP_GLY: 80 - 144

CAP_GLY: 227 – 291

Smc: 381 – 1120

CLIP1_ZNF: 1220 - 1237

CLIP1_ZNF: 1259 - 1275

Clone

Partial ORF

Primers

Forward: TCATCGGAGACAGAGTGTGG

Reverse: TCAATCCCGTCAATCCTCTC

Nucleotide# 503 – 1222 = 720 nucleotides

>CLIP-170_clone

TCATCGGAGACAGAGTGTGGGTCGGAGGAACGAAAGCGGGGTTCATTGCATATATTGGAGAGACTAAATTTGCCCCGGGAGAGTGGGCTGGGATAGTGCTTGATGAACCAGTTGGTAAAAATGACGGTAGTGTGGCGGGGGTGCGTTACTTCCAGTGCGAAGCAAAGAAGGGAGTGTTCGCTCGGCCGGCCAAAGTGAGTCGCCATCAAATCATGGACGATGGACTACCCCTCGCAGCCGAAGCGTCTTCAATGGTCACCCCTCGACCTGCTCCCAAAACAAACGGAACAACATCAGGGTTGACCAAGTTATCTCAGCCGACCCCTAAGGTTGGTCTGGGGGTCAGGAAGAGTATATCGGGCTCCTCTGCGAGCCTCAAGACCTCCCCGACTGGCTCTGTGTCCAACATGAACTCCCACGCCAATGCCGCTGGCGGCATGCAGGTCGGGGACAGGGTCCTGGTCAGTGGCACCAAGGCAGGGACATTGCGCTACTATGGCACGACTGCCTTTGCCAAGGGGGAGTGGGCTGGGGTAGAGCTTGATGACCCTCAAGGAAAGAACGACGGCTCTGTCGCTGGAAAAAGGTATTTTGACTGCCGGCCATTGTACGGCCTGTTTGCCCCCATTCACAAAGTGGCCAGACTCGGAGATGTTCCTCCGCCAGCCACGCCTAAGAGGGGGAGCGTTAGCAGCGTGGGGAGAGGATTGACGGGATTGA

Cloning info

PCR rxn on 07/28/16 using GoTaq polymerase mix

| Template | Initial denature | Denature | Anneal | Extension | #cycles | Final extension |
| --- | --- | --- | --- | --- | --- | --- |
| 2 hr cDNA | 4min @ 94C | 30sec @ 94C | 30sec @ 55C | 2min 15 sec @ 72C | 35 | 10min @ 72C |

**Ndel1**

Gene model: 2996 nucleotides

ORF: 380 – 1354 = 975 nucleotides

>Ndel_full

TGAAAATTCAAGTTGTGTGCGTAGCAATACGCGCACTACAATCTGCCATCTTGTTGAAATTTCGATCTGGCCGTGGGAGGATGGGTGTCAGTTGAAAGCAGGAATTTCTTTGATTTTAAACTCTGTTGGGGATAATGATGTTTGTTAAGTGAAGACTAACTCTTCAGATACGATGTGACTTATTTTGGTGTCTTTAAATGTAACGTTTTTGAAAATAGGAAGCAGATTTTGGGGCGATTTAAATCTCTGTTTGGCTGACCACTTCTCAGCTGGACGTTTTGACATCAGTGGGAACAGTTTCACAGGATTTTCCATTCTGTGTTTTCATCTGAATTCACAGTGATTCATGAACTTGGATCTTTTACCATAAACTGTCAACATGAATGGGGAGGAAAGGAATTTTTCCTCTCCTCAGGAGGAGCTGCAATATTGGAAGGAACTGGCATCTGATTATAAGCAAAGTTACGAAGATGCAAAAGAGGAGTTAGATGAGTTCCAAGCAAGTAGTCGAGAGTTGGAGCAGGAGTTAGAAGCTCAGTTGGAGCAACAAGAAAAGAAAAACAGAGAGCTGGTCTCATCTAACAGTCGTCTACAGATGGAAGTTGAATCTCTTCGGGAAAAGCTGGAAAGCCACCAGTCAGTGAACCACAAGACGATCTCAGATTTGGAGGACAAGTTGGCGCAAGTCACGGCGTTTAAAGACGAGCTGCAGAAGTACATCCGGGAATTGGAGCAGATCAATGATGACTTGGAGAGAACTAAAAGAGCCACAGTGACCTCCCTAGAGGACTTTGAGGCGAGGTTAAACATGGCGTTGGAGAGAAACGCCTTCCTCGAAAGTGAACTGGAGGAGCGGGACACAATGTCAGTCACAATCCAGAGGCTCAAGGATGAGGCCAGAGACTTGAGACAAGAGTTGGTAGTAAAAGACACTGGCCCAATAAAGTCTCCCACATCCAAGTCAATAGAAAAAGAAGAAATAGACAACAATAACAGCTTAAACATCGACAGTAACAAACTGATTGATAGTGAAATTATCCAGCCCTCCACACCCACCAACAAATCTACACATTCTGCATACTCTAACACTGGGTCTCCCTTCACTGCCTCCGCCAGGATCTCTGCCCTCAACATCGTTGGTGACCTTTTGCGAAAGGTTGGGGCATTAGAATCAAAGTTGGCGTCATGTCGAAACTTTGTGAAAGACCAGCCTCCGAGACCTCTGAAGTCCGGTTCTACTAGTCCTGTCAGTTCACCAAGGGCAAAACGCTTGCACAGGTCGGCGACTGGACCCAGCGGGGGTCCTCCTTCCGCAGCAGGGCCGCAGCAAGGCTTCGTGAAAGTCTCGGTGTGAATGGGCAGACAAGAAACTTGTTTACTGAAAGACTATTATTGAGTTATTACAAACACAGCTGTTTATCTATGGTGCACTAGCTCATCTGGAGTGATCTTTTTGCTTGAACAAAGCGAGGTAAAGATGATGAGGAGATGCTGTAATTTAATCAAATTTAATTAAAAGATTTTGAAGTTGAGGTTTGTTATGAGAGCAAGTTCCGTATGTTTTGTTATTCCCTTAAAAATTTGTTATGAATACTGAAAGCAGTTGGGAATTGGAAATTGTTTTAGTTCAACCACTTCAACCGGAATCAACCCAAGAAACATGATGGTTATCAAGATTAGAACAAAACTGGGAAAGTCAAACTGCTTTAATTGTTTGTATATTTTTCCACTTTACAAAGGAAGTCTGTAAAAGATCTTATAAGTGCATAATGTGTCATTTACCGTTGTACCTCTTATGAATATGACATTGTACGAAGGACATTTATTGATATTACCTGTGAATGACATTTAGAGGCGCTGGGATGCTGCGACACTTGTGCAAGGAGAATGGTCTGTGACAATTGTCGCTATTTTATATGTCAAGATCAAAACTGTACCATCAGCAAACACTTTGTAATATTTTAATATGCTATATATACATTGTATTGTATAAATGATGTATTAAGTTATTCTGAATAAATGGGACTTAGATTTTTGTAACACTTGCAGATCTTCAACAACTTAACATTTTGTGCACGGCTATGCCAGACTGTGCTATAAATATTTCTTCAGTATTTTGCATTCCAATAGATTTGGCATTAAGGAACATTGAGTTGTGTTTGTAGAGGTTAGAGCACTGGCCTGTATTTCACCTGCCAGTGTAATTAGTTGTAATTTATTATGAATTTATGATCTACTTGTAAATTGTACAGATTGTCACCTGTTTATAATGCTGCGTCTCTCCGAAGAATTGCAATGAATGACATACATGTTTCTGCATTGTACATAAGGCCGCATTTAGCTGATGGTTTGCTTTATGGATTTGCTTTTTTAAGGCCAACTTTTGGAAATTTGCAATTCAGTGTGCTATGTGAAGAAACCTAAGATTGAGATAAAAATTGGATCTTCCCTGTAAAAATATTTCATCTCAAAATTTAAGCCAAGGACATATTTCTCCAGGAGGTGAATATTCTGCTGTTTACGTGCAAGTCTAGGAACCATGTTAGTGTGAATCTTGATTGTCAAAACGGTAAAGCAACTTCAATGGAAACTGACTAGTCTGCAACACCACCTTGGCCCCCTGTTTAGCCCTATATAGGCTTTGGAGACTTGGTTTGTTTGTCAGTTTTGTGGCTTGGTTGAAAACCGGGAAAAGCTTATTAAAAGAGTTTAAGATTTCTTCAAGACTTCAAGAACTTTGCAAACGGGTTCTTATTGGCAGATCCAAGGAGCTAAGCATCAGGTAAATGTGGATATGAATCTGGCCTTCAGTAAAGATCTTTCTCTGCAGTTTTGCCTTCAATATTGTTGTTGTAAATCTCACATCTCAAACACAACATAAAACAAAAGCATGTAAATTCCAATGACAATTGACAAAATGTGGGGTTTCAAACTGTTTGTAAAAAGATAAATTTAGAGAATAAATTTATTGTGTATATACTATTAATACAAAAAAA

Protein: 324 aa

>Ndel1_protein

MNGEERNFSSPQEELQYWKELASDYKQSYEDAKEELDEFQASSRELEQELEAQLEQQEKKNRELVSSNSRLQMEVESLREKLESHQSVNHKTISDLEDKLAQVTAFKDELQKYIRELEQINDDLERTKRATVTSLEDFEARLNMALERNAFLESELEERDTMSVTIQRLKDEARDLRQELVVKDTGPIKSPTSKSIEKEEIDNNNSLNIDSNKLIDSEIIQPSTPTNKSTHSAYSNTGSPFTASARISALNIVGDLLRKVGALESKLASCRNFVKDQPPRPLKSGSTSPVSSPRAKRLHRSATGPSGGPPSAAGPQQGFVKVSV

Domains

NUDE_C: 134 - 303

Clone

Partial ORF

Primers

Forward: GTCGAGAGTTGGAGCAGGAG

Reverse: CAGTGAAGGGAGACCCAGTG

Nucleotide# 507 – 1106 = 600 nucleotides

>Ndel1_clone

GTCGAGAGTTGGAGCAGGAGTTAGAAGCTCAGTTGGAGCAACAAGAAAAGAAAAACAGAGAGCTGGTCTCATCTAACAGTCGTCTACAGATGGAAGTTGAATCTCTTCGGGAAAAGCTGGAAAGCCACCAGTCAGTGAACCACAAGACGATCTCAGATTTGGAGGACAAGTTGGCGCAAGTCACGGCGTTTAAAGACGAGCTGCAGAAGTACATCCGGGAATTGGAGCAGATCAATGATGACTTGGAGAGAACTAAAAGAGCCACAGTGACCTCCCTAGAGGACTTTGAGGCGAGGTTAAACATGGCGTTGGAGAGAAACGCCTTCCTCGAAAGTGAACTGGAGGAGCGGGACACAATGTCAGTCACAATCCAGAGGCTCAAGGATGAGGCCAGAGACTTGAGACAAGAGTTGGTAGTAAAAGACACTGGCCCAATAAAGTCTCCCACATCCAAGTCAATAGAAAAAGAAGAAATAGACAACAATAACAGCTTAAACATCGACAGTAACAAACTGATTGATAGTGAAATTATCCAGCCCTCCACACCCACCAACAAATCTACACATTCTGCATACTCTAACACTGGGTCTCCCTTCACTG

Cloning info

PCR rxn on 07/28/16 using GoTaq polymerase mix

| Template | Initial denature | Denature | Anneal | Extension | #cycles | Final extension |
| --- | --- | --- | --- | --- | --- | --- |
| 2 hr cDNA | 4min @ 94C | 30sec @ 94C | 30sec @ 55C | 2min 15 sec @ 72C | 35 | 10min @ 72C |

**Lis1**

Gene model: 5154 nucleotides

ORF: 499 – 1728 = 1230 nucleotides

>Lis1_full

CCAAGTGAATTGAGTGTTTATTTGCTGTGGGATCAAGTGTTTGAACTGTGTTTGACAGGTAGACAGTGTGTATTGGAAGATGACATCTGGAGCTGTCAACACTGACATTTGATTGATTCTCCCCAAATACTCATAATAATCATTGATTCTGAATATTTGCGTGCATTGTCAGATTATTGTCCCATTAGTTCTTTCATATCACTTATTTACAATGCGATTACTACCTGTGGTATGTGTGTCATGTTATGCTATTGCTATGCTCATTACATTATGCTAAGAGAAGCGGTGTCTCAAATGTATTCATGAGTCAACATACTGTGAAAGATCCTTCATAAGTATAACCCAACTTCTTTTCTCCCTCAGAATGGAATGAACAGCTGGTGGATTTCTGAGGAGGAAGTTTACCAGATTGGATTGCACCGATTTTCGAAGAATAGCTGTTAGCATATCTTCCATTGATTATTCTACACAGTAGAACTTAACCCAACTTGAGCCAAAATGGTTCTCTCACAGAGACAGCGAGAGGAACTAAATAAGGCCATTGCCGATTACTTCAGTGCCAATGGATATACAAATGCCTTGGCCGAATTTCAGAAAGAGGCAGATATGCCTGGAGAGATAGAAAAGAAGTATGCTGGTTTACTAGAGAAGAAATGGACTTCAGTAATCCGGCTGCAGAAGAAAGTTATGGACTTGGAGGCGAAATTAGCGGAGACAGAAAAGGAGTACATCCAGGGTGGTGCACCCACGAGGGACAAGCGGAGTCCCCAGGAATGGATTCCTCGCCCCCCTGAGAAGTACAGTCTGAGCGGACACAGGAGTCCTGTTACTAGGGTCGTCTTTCATCCTGTTTTCAGTGTAATGGTGTCGGCCAGTGAAGACGCTACTATCAAAGTATGGGACTATGAAACCGGTGACTATGAAAGAACATTGAAAGGACATACTGACTCTGTACAAGATGTGGCTTTTGACCACACTGGGAAGTTTCTAGTATCATGTTCAGCTGATATGACAATTAAACTCTGGGACTTTCAAGGATTTGAGTGCGTTAAAACTATGCATGGCCATGACCATAATGTGTCCAGTGTAACTTTCATGCCCAGCGGCGATTTCATTGTCTCTGCCTCTCGTGACAAATCTATCAAGATGTGGGAGGTCTCCACAGGGTATTGTGTAAAGACGTTCACTGGCCACCGTGAGTGGGTGCGAAATGTGAGGGTGAACCAGGACGGTTCTCTGCTTGCCAGTTGCTCTAATGACCAGACCCTCCGAGTTTGGGTTGTGGCCACGAAGGAGTGCAAAGCAGAACTGAGAGAGCATGAACACGTGGTCGAGTGTCTTGCTTGGGCACCAGAGTGCGCTCATGCTGCCATCAACGAAGGAGCTGGAGTAGATATTAAGAAAGGACAGCGTTCTGGTCCGTACTTGATTTCTGGATCCCGTGATAAGACCATCAAGATGTGGGACGTTAGTACAGGAATCTGTGTCTTCACTCTGGTTGGTCACGATAACTGGGTGAGAGGGCTCGTCTTCCATCCTGGTGGCAACTACATCATTAGTGCTTCTGACGACAAGACGCTTAGAATTTGGGACATCAAGAACAAGAGAAATAACAAGACCTTGAATGCTCATCAGCATTTTGTCACATCTGTTGATTTCCACAAAAATGCACCATATGTGATAACCGGCAGTGTGGATCAGTCTTTGAAGGTGTGGGAATGTCGCTAAGGGGCTGGGGCGACAGGTAGTTCTAGAGGACGGCGTGAGTGTGGCTGGCGGCTGCAGGTTCAGCAGGACGACGCCTGAGCGGATGATGATGTGATTCCCACTCGCTCGCAAGCCACCCTTTGGCCCGCCCCAGTATTCCACAACAGTCTACATGAATCCACTCGTGATAATTCAGTCACTTAATCATTTCCCATTTTGTGATCCTCAGTGCATTCTATATTGTGCAGTTGGCATTTCAGTGTCATATATATGATGATAATAATCTACCCACATACAACTAATGAATTAGCAACACAATGACAAAGAGTAAAATATATTGGTTTTGTTAATGTCATAATCTGCAACAGAATTGGCTGTTATATGTTTCCATATCGTATTGAAAAGAGCAGCCTGCTTTTATTCTTAGTATGTAAATGACAGCTTGTGTATATTTGTGCATAGTGACAGAGCATGACTGTTTTAGTATGTTACTAGTGGCTGAGTGTGAACGCTACCATGGGCTGGCCCTTCCACTTGCATGGGACGAAGAAGATGACATGTTTTGCAATTAAGTGTGGCTGCTTTTTCCTCCGCACCTCCTGCTGCTCAGCTGCTCTCCGACAAACTAGTTTGTGACGACGAACCAGTTTGTAACAACAAACCAGTTTGTGACGACAAACCAGTTTGTAATAACAAACCAGTTTGTGACAAGCATGTGTGTGGCTCGCACGCACCTGACATGTTGCTGAAGTTATCCTGGCTATGAATAGAATTGATGGAGAATGAATTGCAAGGAAATAGCCAACAAGAAAGGGAGGAGGGCCTTTGACTTTGAGAGAAAGGCGCATGAACGTGTTAGGCGTCCCCATCCAAGGGCAGGCAATGTTATCACTCGATTACCACTGATAGTATTACGTTTGTGTGTGTGCATTGCCATCTGTGTTAATTAGCAGGGGGGACGAGGTTATCACTTACAGCAGTTTATCCAATTCTCATTACAAGTGTGATTTGATCGCACAATCATCACAAGCAACTTCTTGGTCATTGGGACTCAGTTGAATCGTTTCAATGACGACTTGTGAGCTCATCTTTACCTGATTTCAATTTGGAGGGAAAAACATTCATCTGACGTGTGCCGTGCATTTATTTTCTCATATTTTGCTATAACCCAGATATATTTGATGCCATGAAATGTTTTCAGCTAGGTTATTTTGTTGCACAGTTTCATTAATGAACACTAGGAGGGAGGGGAGGTGGCGCTATGTAGATTTGTACAGTATTATTTATATATTGTGTATTTAATTGATGTCTGTGCAAGCAACTGAATTCCTTGAGTAATTGAAATACATTGTGATATATATAGAAGGCGAGGTAACGAGGGAGAAGGGAGGGACAGCTGCTTCTTCACCATTGAGTTGGTTCTCTAATCACCCAAGTCGTTCGGGCTTTCAATGTTAAGTTTGTTGAACAAGCAGCGACCATATATAACAAGACATATATATGCTGAAATTTTTGTGGCATTTTTCGCACTAGTTGTAGGTTATTTTGAATATTTTCAGAAATAAAATTTACATTGAATTTGAAGCGTGGGATGGGGCAGTGAATGTACGTATGACTGAGTGCTATGCAAGTGCAGAAAGTGACTGTTTATTTGAAGAAAAAAAATGTTGCAATAGTAATTTTGTAGTCCTGACAGTGAACTATTTTCAAACTTATTACCGCTTTGCTCTTCCCTGATTGGTGTGGCAATGGCAATTAAAGGTCTCTACGAGATATATAATCCAACCCCTTCGCACTTATGGACTAATTGGACTATGGTTATGTGCGCTATGTTATAATTTATGGCTCGTCAGATGACTAAGACAATGTGTGAGGACTCAAGTATTAGACCTGATCTGCGAAGTCTTTTGTGTTGTGTGTAAAACAGAAGCTCACCCTCTCACCTGTGGTTTGCAAAAATGATCCAAGTGATTTCCTGACTGCCAAAGAAGATCAACTGCATCCTGGCCGTGATGTCTTGACAGCGATTTTAACCTGTTACGAACTCTGTTGCTGCCACGACATTCCCGTCAGGATGGTCCGCCCAGACTAGTAAAGGGAATCACCGACGTGTAGTGTTGTTAGTGAGGCGACTTTTCGCTGAAGCCTTTTGCCGTTGGCCTTCCCTTTCTCCTTTCTCCTTGGTCTCACCCAGTTTTCTCCGATAGAGTTCTCCTATTTTTTTTTCGAAGCAAGTATTGTGGACAACTACGTTGGTGCTTTTCAGTCTCGCGACAATTTTCGACGAGAAAACTGGTTGGGTAGCCAAGGGGCTTTTCATGAGGGGACCGCCGTCGGCAACTCGCTCCAGAAGACGTCCCTTTGTGGAGTAAAAAATCCTACACTGATTGATTGATGAAAACCACCAATAAATGTTTACAGAGTGTAATATATATATACATACATATATATTTATTTATTGTCTTCGAGTATGTACCCGGTATAGCTTTTGTGAGCGCATCCTTTGCACACTAAACCGTCACACTTGTATGGTACAGTTACATGTACTTACTAAACGCTAAAGAAACCCTGAACAAATTTGCCACATCAATTTAATTTTTGTTTTCAAATTAATTAATTTTACTGCTTTATGTTCATTATATTTGAGTGCCACTAATGAAAGACCAATTATAGTCTTCTGTTTTTTGGGTTTTTTCCAAGCCATATGATTTTTTTGTTGTGTATATGTGCTATATGATTTGTAAATATTGCTTATTTAGGATGAAGTTTTCTTCACATTAATTCTGACTTTAAATTATTTTGACAACCATGATGATTTCTGCATTGAACTATATGAGAGCTAATGGTCGTTGTCTTTTTGTGAGACGCAATAGTGCGTTTGATGAATCTATTTAAAATTGACTATCTGCTTTGTGTAATTCTTTATGATGAGCAAGAATAAGTATAGCTTTTAGATTCAATGGTGTATGTCGAGTACACTGGTAATTGATCTTGCATGAAAATGGAGAAGTATTTATGGTTCGTATACTTAATAGAACTAATCAAAACCTGGTACAGATTAAATCCAATTTACGCCTTCGTGTTGTATCAGGGGACTAAGCCGTGCTATTGTAGGCTTGAGTTGTACTAAAGTCGTACTACCGTATAAAAGCTTCTTGGAAATACTTTAGATCTGTGCCGTGCCGCGGCCTGTAGCTAAGAGTGGCTTCTTATTCTTGCTGTTAAAGGATCAGTGATGTTGATGGGAGAAGTCTTTTGCATGAGGGAGGGAGGGAGACCGTCAATGGTCGCACTGGATTCTGGCAAAGATTTTAGACTCGCGATACATAATCAATTTATCATGGTAGTCAGCATAATCGTAACTGTAAATGTGTACATTTTAATGATTACTCCTTGTCAGAAATTACATATATGTTTAATAAATGCATCTTATGCCTCAAAAAAAAA

Protein: 409 aa

>Lis1_protein

MVLSQRQREELNKAIADYFSANGYTNALAEFQKEADMPGEIEKKYAGLLEKKWTSVIRLQKKVMDLEAKLAETEKEYIQGGAPTRDKRSPQEWIPRPPEKYSLSGHRSPVTRVVFHPVFSVMVSASEDATIKVWDYETGDYERTLKGHTDSVQDVAFDHTGKFLVSCSADMTIKLWDFQGFECVKTMHGHDHNVSSVTFMPSGDFIVSASRDKSIKMWEVSTGYCVKTFTGHREWVRNVRVNQDGSLLASCSNDQTLRVWVVATKECKAELREHEHVVECLAWAPECAHAAINEGAGVDIKKGQRSGPYLISGSRDKTIKMWDVSTGICVFTLVGHDNWVRGLVFHPGGNYIISASDDKTLRIWDIKNKRNNKTLNAHQHFVTSVDFHKNAPYVITGSVDQSLKVWECR

Domains

LisH: 7 – 38

WD40: 103 - 407

Clone

Partial ORF

Primers

Forward: TGGCCGAATTTCAGAAAGAG

Reverse: CCCAGTTATCGTGACCAACC

Nucleotide# 581 – 1516 = 936 nucleotides

>Lis1_clone

TGGCCGAATTTCAGAAAGAGGCAGATATGCCTGGAGAGATAGAAAAGAAGTATGCTGGTTTACTAGAGAAGAAATGGACTTCAGTAATCCGGCTGCAGAAGAAAGTTATGGACTTGGAGGCGAAATTAGCGGAGACAGAAAAGGAGTACATCCAGGGTGGTGCACCCACGAGGGACAAGCGGAGTCCCCAGGAATGGATTCCTCGCCCCCCTGAGAAGTACAGTCTGAGCGGACACAGGAGTCCTGTTACTAGGGTCGTCTTTCATCCTGTTTTCAGTGTAATGGTGTCGGCCAGTGAAGACGCTACTATCAAAGTATGGGACTATGAAACCGGTGACTATGAAAGAACATTGAAAGGACATACTGACTCTGTACAAGATGTGGCTTTTGACCACACTGGGAAGTTTCTAGTATCATGTTCAGCTGATATGACAATTAAACTCTGGGACTTTCAAGGATTTGAGTGCGTTAAAACTATGCATGGCCATGACCATAATGTGTCCAGTGTAACTTTCATGCCCAGCGGCGATTTCATTGTCTCTGCCTCTCGTGACAAATCTATCAAGATGTGGGAGGTCTCCACAGGGTATTGTGTAAAGACGTTCACTGGCCACCGTGAGTGGGTGCGAAATGTGAGGGTGAACCAGGACGGTTCTCTGCTTGCCAGTTGCTCTAATGACCAGACCCTCCGAGTTTGGGTTGTGGCCACGAAGGAGTGCAAAGCAGAACTGAGAGAGCATGAACACGTGGTCGAGTGTCTTGCTTGGGCACCAGAGTGCGCTCATGCTGCCATCAACGAAGGAGCTGGAGTAGATATTAAGAAAGGACAGCGTTCTGGTCCGTACTTGATTTCTGGATCCCGTGATAAGACCATCAAGATGTGGGACGTTAGTACAGGAATCTGTGTCTTCACTCTGGTTGGTCACGATAACTGGG

Cloning info

PCR rxn on 07/28/16 using GoTaq polymerase mix

| Template | Initial denature | Denature | Anneal | Extension | #cycles | Final extension |
| --- | --- | --- | --- | --- | --- | --- |
| 2 hr cDNA | 4min @ 94C | 30sec @ 94C | 30sec @ 55C | 2min 15 sec @ 72C | 35 | 10min @ 72C |

**PatJ**

Gene model: 3022 nucleotides

ORF: 195 – 3022 = 2827 nucleotides

>PatJ_partial

CAGGAAATAGCGCCCTAAGCAGGCAATACTTGTGTTTTCGCTCACCCAGGGTTTGTGTTTGGTGTTTCGCTGCGATTGTACGCACAGTTTCCTTTGCCTTGCATAAATTCGGGTGCTGGATTTACATCCATAATATATTTAAACTATTGCTCTCTTGTTCCGTTCTATGGGTTGTGAAGTTTGAACAAGGCAACATGTCTTTGTATGCTGATTCAAAGCATGCATTGAAGATTTTAGAACAGCTCCAGACAAAGCTTTTGAATGGAGACGACTTGAGTGCAGAGTCAGACCTGACCAATCTCATCTATATGCTTGATTCTCCCCTCTTTCTGCAACTGCTTAACATTCAAGATGCTATTCAGATACTCAAGGAAATTCATCTACAACGTCCTCTCAGCCCCAATGACTTTTCGATTAATCCCATCAATGGACAGCTCAGTGTTGAGAATGGCAGTCCTGACAACACGATGGGATCCCTGCACAGCTCTGGGACTGTCAGCTCATCGGCCACAACAGTCAAGAGCCAGCCTGTCAAAACAGAACCAATCCACAGCAAACACAATTCCATTTCGAGTGGAAAGTTTGAGGCAGAAAAACAATTTGAACCAGAACTGCAGGCGGCGGTGGATCGGTTGGCAGGCGGACGACCAGTTGATGTGATAGAACTATACAAGCCGGAGAACACGAGTCTTGGCTTCAGCGTGGTCGGCCTCAAGAGCGACCACAAGGGAGAATTGGGCATCTATGTGCAGCAGATCCAGCCTGGTGGTATTGCTGCAAGAGATGGACGGCTGGTGGAGGGAGACCGCATCTTGGCCATAGACCGGCAGGTGCTGGACACCAACATCTCTCACGAATCGTCCATCCAGATTCTCCAGGCGGCCCAGGGCCACGTGGAGCTGGTTGTAGCCCGGACCCCCCACAGCCCCCCTGCCGCCCTCACCAGCACCACCACTGCCCAGGTGTCAGCCAGTGCCCCTCCTACCCTCCTTGAAGATACTGTGGCTCCCCTGTCTACCTCCATTGAAGCTGTCGACAGGGCCAGCAACCAATCCTCCACCTTGCCTGATTCCTCAGACATGGTCTTAAACACAGAATGGACTCAGTTGGAAGTAATCGATTTAATCAACGATGGTTCTGGACTGGGATTTGGTATCATCGGCGGAAAGAGCACAGGTGTCGTTGTCAAGACGATACTCCCCGGTGGAGTTGCCGACAAGGACGGGCGGCTCCACAGCGGTGACCACATTATGCAAATTGGTGACGTGCACGTTCGAGGTCTTGGTTCGGAGCAGGTAGCCACTGTTCTTCGTCAGTCTGGCAGCCATGTGAGACTGATAGTGGCCCGCAGTGTGACTGAACCCTTCCCCATGTCACACCCCCATGCTCCCATTGTGCCCACTGACCAATTAGACGATCATCTTCACCAACTCTACTCAGCACTCCTGGCTTATGAGAATGCCTCGTCTCTCGGCCTTACACCGGAACAACTTGAACATCTTAGTATGATGGGACAATTGCCCGAAAACTTCTTACATGGAAATGCTCATTCCTTAGAAGAGGCTGATCTCCATTCGCCTGATGTGGAGTTTTTTGAGGCGGAGCTCGTCAAAGGAACACAGGGTCTTGGTATCACCATCGCTGGCTACATAGGAGAGCGAACTACAGAAGAATTATCAGGTATATTCGTAAAAAGTATAGCAGAGGAAAGCGCTGCTGCTCTCGATGGCCGAATACAAGTCAATGACCAGATTATTCAGGTTGATGACCAAGGGCTAGACGGCTTTTCCAACCACCAGGCTGTGGAAGTTTTAAGAAATACAGGTCAAGTTGTTCGGTTAAAACTCGCCCGCTACAAACATGCAACTAAGTACGATCTTCCACAATATCCAGTACAACCGTGGGACCCACCACCTGTGGAGACCACCACTCCCCCACAAACCCTTCCAGTGCAGACCACCCCTCCTCCTGTGGCCATTCCGTACCCTGATGATCTGGAAAACGAGGTCACGGTGCAATCCGAGGCAGCCAGCAACGGACACATCAGTTTGGAGGACATCACTCTCTTGCCTGAATACAGTGACTACAGCAAAGAACTGTCTCCCGAGATTGAAGAAGCCTTGAGAGCACAATGGCAGGCAGTCATGGGAGAAGACATCCAGATCGTGGTTGCACAATTGTCTAAATTTAACCCATATGGTGGCCTAGGCATCAGTTTAGAGGGCACGGTAGACGTAGAAGACGGACAAGAGAAACATCCGCATCATTATATCCGTTCTGTGCTCAGTGATGGGCCAGTTGGTAGGAATGGCAAGCTCAAGAGTGGTGACGAACTGCTGGAGGTCAATGGAGAATCTCTGATAGGCCTCAACCATGTTGAAGTAGTAGCAATATTAAAAGAACTGCCGCAGCATGTGCGTATTATCAGTGCTCGGAACAAGCCCTCACCGTTGCCTGCTCAACCGCCAGACTTTCTTTCTTCTTCCTATGCATCCATATCTGCTCCTGCTGCTGCTCGCCCTGCTCAGCTGGAGACGCCCCCTTCGGCCAGTCCGCTCCTCCCATCCAGCGTATCAAGCAGCCAGGAGGTCTACAGTCCGCAACAGGCTGCCCACCTTGTCAAAGCAAAGTCTGACCAGGCCTTGCACATATCAAACGTTGCCTTGGCATCCTCACTGAACAAAATGAAATCTCGGTCGTTGGAACCACTCACTAGTTTAGCCATGTGGTCCAGTGAGCCCCAGGTCATTCAGCTGCACAAAGGAGACCGTGGTCTTGGCTTTAGCATACTTGACTACCAGGATCCCATGAATCCTGATGAAACGGTGATTGTGATTCGCAGTTTAGTGCCAGGTGGCGTTGCACAGATGGACGGTCGTCTTGTGCCTGGAGATAGACTTTTATTTGTAAATGACATTAACCTTGAAAATGCCACCTTACACGACGCTGTGACGGCACTGAAAGGAGCACAGCCTGGCATCGTGTGCATCGGTGTAGCAAAACCAATTCCATTACCAGAAAT

Protein: 942 aa

>PatJ_protein

MSLYADSKHALKILEQLQTKLLNGDDLSAESDLTNLIYMLDSPLFLQLLNIQDAIQILKEIHLQRPLSPNDFSINPINGQLSVENGSPDNTMGSLHSSGTVSSSATTVKSQPVKTEPIHSKHNSISSGKFEAEKQFEPELQAAVDRLAGGRPVDVIELYKPENTSLGFSVVGLKSDHKGELGIYVQQIQPGGIAARDGRLVEGDRILAIDRQVLDTNISHESSIQILQAAQGHVELVVARTPHSPPAALTSTTTAQVSASAPPTLLEDTVAPLSTSIEAVDRASNQSSTLPDSSDMVLNTEWTQLEVIDLINDGSGLGFGIIGGKSTGVVVKTILPGGVADKDGRLHSGDHIMQIGDVHVRGLGSEQVATVLRQSGSHVRLIVARSVTEPFPMSHPHAPIVPTDQLDDHLHQLYSALLAYENASSLGLTPEQLEHLSMMGQLPENFLHGNAHSLEEADLHSPDVEFFEAELVKGTQGLGITIAGYIGERTTEELSGIFVKSIAEESAAALDGRIQVNDQIIQVDDQGLDGFSNHQAVEVLRNTGQVVRLKLARYKHATKYDLPQYPVQPWDPPPVETTTPPQTLPVQTTPPPVAIPYPDDLENEVTVQSEAASNGHISLEDITLLPEYSDYSKELSPEIEEALRAQWQAVMGEDIQIVVAQLSKFNPYGGLGISLEGTVDVEDGQEKHPHHYIRSVLSDGPVGRNGKLKSGDELLEVNGESLIGLNHVEVVAILKELPQHVRIISARNKPSPLPAQPPDFLSSSYASISAPAAARPAQLETPPSASPLLPSSVSSSQEVYSPQQAAHLVKAKSDQALHISNVALASSLNKMKSRSLEPLTSLAMWSSEPQVIQLHKGDRGLGFSILDYQDPMNPDETVIVIRSLVPGGVAQMDGRLVPGDRLLFVNDINLENATLHDAVTALKGAQPGIVCIGVAKPIPLPE

Domains

L27_2: 6 – 60

PDZ: 155 – 239

PDZ: 312 – 384

PDZ: 465 – 553

PDZ: 666 – 744

PDZ: 849 - 930

Clone

Partial ORF

Primers

Forward: CTCCCCTCTTTCTGCAACTG

Reverse: GGGCAATTGTCCCATCATAC

Nucleotide# 319 – 1523 = 1205 nucleotides

>PatJ_clone

CTCCCCTCTTTCTGCAACTGCTTAACATTCAAGATGCTATTCAGATACTCAAGGAAATTCATCTACAACGTCCTCTCAGCCCCAATGACTTTTCGATTAATCCCATCAATGGACAGCTCAGTGTTGAGAATGGCAGTCCTGACAACACGATGGGATCCCTGCACAGCTCTGGGACTGTCAGCTCATCGGCCACAACAGTCAAGAGCCAGCCTGTCAAAACAGAACCAATCCACAGCAAACACAATTCCATTTCGAGTGGAAAGTTTGAGGCAGAAAAACAATTTGAACCAGAACTGCAGGCGGCGGTGGATCGGTTGGCAGGCGGACGACCAGTTGATGTGATAGAACTATACAAGCCGGAGAACACGAGTCTTGGCTTCAGCGTGGTCGGCCTCAAGAGCGACCACAAGGGAGAATTGGGCATCTATGTGCAGCAGATCCAGCCTGGTGGTATTGCTGCAAGAGATGGACGGCTGGTGGAGGGAGACCGCATCTTGGCCATAGACCGGCAGGTGCTGGACACCAACATCTCTCACGAATCGTCCATCCAGATTCTCCAGGCGGCCCAGGGCCACGTGGAGCTGGTTGTAGCCCGGACCCCCCACAGCCCCCCTGCCGCCCTCACCAGCACCACCACTGCCCAGGTGTCAGCCAGTGCCCCTCCTACCCTCCTTGAAGATACTGTGGCTCCCCTGTCTACCTCCATTGAAGCTGTCGACAGGGCCAGCAACCAATCCTCCACCTTGCCTGATTCCTCAGACATGGTCTTAAACACAGAATGGACTCAGTTGGAAGTAATCGATTTAATCAACGATGGTTCTGGACTGGGATTTGGTATCATCGGCGGAAAGAGCACAGGTGTCGTTGTCAAGACGATACTCCCCGGTGGAGTTGCCGACAAGGACGGGCGGCTCCACAGCGGTGACCACATTATGCAAATTGGTGACGTGCACGTTCGAGGTCTTGGTTCGGAGCAGGTAGCCACTGTTCTTCGTCAGTCTGGCAGCCATGTGAGACTGATAGTGGCCCGCAGTGTGACTGAACCCTTCCCCATGTCACACCCCCATGCTCCCATTGTGCCCACTGACCAATTAGACGATCATCTTCACCAACTCTACTCAGCACTCCTGGCTTATGAGAATGCCTCGTCTCTCGGCCTTACACCGGAACAACTTGAACATCTTAGTATGATGGGACAATTGCCC

Cloning info

PCR rxn on 05/05/16 using GoTaq polymerase mix

| Template | Initial denature | Denature | Anneal | Extension | #cycles | Final extension |
| --- | --- | --- | --- | --- | --- | --- |
| 2 hr cDNA | 4min @ 94C | 30sec @ 94C | 30sec @ 55C | 2min 15 sec @ 72C | 35 | 10min @ 72C |

**Pals1**

Gene model: 4100 nucleotides

ORF: 499 – 2736 = 2238 nucleotides

>Pals1_full

AGTTTACCTGAGATCTCAAATAGCACACCTGTATTTTTCTGATGTCATGATCTTGCCCTATTTTTGCAGTGCATCAAAACTCGAACAATACAACTGAGAGTTAGGAGGAGTTTATCTGGAGAATTCCTGGGGCGGGAAGGCAGAGACATCCAGAATGCCTACGTGGTGGCTGTCGAGGAGACTGCTAGGAAGCGTCTGGAGGACTTGACGGCCCAACATAAGGTGCAGGCGTTTGATTTGGCATTAGTCGAAGAAAGGAATGAGAGAGAAGGAGATGAGGTAGGAGAGGAAGAGAGGGAAGAGAGATCTCCCACTGATAAAGAGGAGGAGAAGGAGGAGGAGGAGGTGGTGCAAGAGGGAGAAGAGGGAGAAGGCAAGCTTACTCCAACTCCCGAAGGTCCTCCACGAGAAGCGTCGTCCCCAAAGACCCCCTCGCCAGTCAGTAGTACCTCAAGGTGCACGTGTGTGGCCTCCTTTGCCAGGGCAGAGGAGCGTAACATGGACAGTGCCATGGTGAATGGCTACATCAACGGGCGCTCTAACGGCCTAATCCACGAGCATGCTGGTTCGCACAAGATGGCCGCCATCAACAGCAACCAGTACATGATCAACCAGCGGTCGGACGACCACATTAATTATGACTCTGACTCGGACAGACGGCCGCATCGAGAAATGGCCATCGATGTGCCCGAGAACTTTATCGGCCAAAAGAAAGAGCGGCCCAGTTACCCCCCAACATTAACTAAAAACTCCTCCCCCCGTAAGAATCCGGATCAGCATCCGGATCAGCATGTGGCTCCCGCTCAAGATGCCACGGCTGACGAGATGCAGAGGATACGCAAATACCAAGAGGACCTCCGGAAGAGGCGGGAGGAGGAGGAACGCCATCTCAAAGAAGAAGAATTCCTCCGAACTTCCATGAGAGGTTCAAAGAAACTACAATCCCTCGAAGAACAAGCGCAACAGCCGGGCTTTGTGAACCCCACGTATGAAGAGGATACAGAATCACGGCCGCACTCCACAGCATATCCAAAGAAACTCTTAGCCGTAGAAGACCTGTTTGCTTCTTTACAAAACCTGCAGAAACAACTGAGGTCTGCTGAAGACCAAAAAGATGTTGCCCAAATCTCAAAACTATTCAGCAATACTCGCTTCCAACAGGCCCTACGCATCCACAATAAGATGGTGGAGGTCAATCTTCAGCGTCACAAACTGTCACCCGTGGCTGACAATTCCCAAGAACTCTGTGGCGATGTCATGAACTCCTTGAATGCTGCCCATGGCCACCCTGTGGCGGAGGAGCTGAGGGCCATTCTACAGACGCCTAACCTTAAGATGCTGATGTACACACACGACCATGTAGCCACCAACAGAGCACGTCTTTCTGCCAGCGCCCCCAATGAAGACGACGACCAAGAGTATCTTTACGAGAGAGTTTCACAGTACAACGCGGACAGCATCAAGATCGTACGACTCCACAAAACTGCTGAACCATTGGGAGCCACAGTACGCAATAACGGTGAATCTGTAATAGTTGGGCGAATAGTCAAAGGGGGCGCTGCTGAGAAAAGTGGGTTACTTCACGAGGGAGATGAGATGCTGGAAATAAACGGGATCGACATCCGAGGGAAGTCTGTCAACGACGTCTGCGATATCATGGCTAATATGACAGGTACTCTGACATTCCTAATCGTACCCGCACAAGACTACATCGCTGACACGTTGTCCTCCGAACCCGAAGACAAAGTGCTGCATTTGCGAGCGTTGTTTGATTACGACCCCGAAGATGACATCTACATCCCGTGCCGAGAGTTGGGCATCTCCTTCCAAAAGCGAGACATCCTGCACGTGATCAGCCAGGAGGACGCCAACTGGTGGCAGGCGTACAGGGAGGGCGAAGACGACCAGGCGTTGGCCGGCCTCATTCCCAGCAAGTCCTTCCAAGAACAACGTGAAGCCATGAGGCAGACAATCGTGAACGACAGTAAAAGCAACAAGGGCAAGAAGAAGCCATTTTGCCACGCATGTCGAGGGGGCAATAAGAAGAAGAAGCAACTCTACGGAACTGCTAATGATGACGCAGAGGCAGAGGATATTCTGACTTATGAGGAAGTTGCCCTCTATTACCCACAACCAAATCGTAAACGACCGATAGTTCTGATTGGTCCCCCTGGCGTCGGACGACAGGAACTACGATCGCGCTTAATGACGAGCGACCAAGAGAGATACGGAGTGGCTGTTCCACACACGAGTCGCCCCCCTCGGGAGGATGAAAAGGACGGCATAGATTACCACTTTGTCTCCCGTCCAATCCTGGAGCAAGACATTGCAAACCAGAAGTTTGTGGAGTTCGGAGAGTATGAGAAGAATCTTTATGGAACGAGTTTGAACGCCATTCGAGCAGTTGTGCAGGCTGGCAAGATCTGCATCCTCAACCTCCACCCACAGTCATTACGCATTCTGAAGAATTCCGACTTGAAGCCCTACATTGTGTTTATAGCCGCTCCTAACATTGACAAACTACGCACAAACAGAATTAAAGAGAAGGTCAAATTTACGGAAGTGGAGTTGAAGGAGATTATTGAACACTCGAGAGAACTTGAAGAAAATTACGGACATTACTTTGACTACATCATAGTTAATTTTGACCTGGATAAAGCGTACAATGAGTTGCTGCAGGAGATCAACCATTTGGAAGTGGAGCCCCAGTGGGTGCCCGCCCAGTGGGTTTTGTGATGAATTAATACGTTGTTACTGATTTGACTTGTGGTGGCGCCACTGGACATTGAACTCACTTGAGATAGACTGTGCTGACCCCTGGTGGATTCTGTGCTCTGCATTTGACTTTAATCTACTTATGACTCTGTACTGTACAGTGTGCGTAGTCTTGGATAATACCGTTATCATATCATGGATATTATTCTCATACAAGCTCCAGGACTCTGCCAAATGTGCATCTCTAAGCTGAAGTAAGCTGGTCTGCTACTGGTTGAAGACTGCATTTTATTTTGCGTTTTTTTGTTACGACTGATTATAGTCTATTGCACTGTTAACACATAGCATGATTGCTAACAGATTGATATTTGGTTTCGTAATCACGGTCTTACGCTGATGTTATACTGAGATAGAGCTGCTGACGGTTTGCACTTCAACATGTTTAAGCAGAGAGAATATTGTGTTGCGATCACTTATAAAATGGTTTAGGTTTATTTCCTAACTTTCATTGATTAATGATATTTTTTGTTATGTATAAATTTGTACCCACCCTGTGAAATATCACAAAGAATGAAGTAATTAAGATACACTGACTTATATCAAAAGTGCGCACTGCCAATCAGGTTTACTTTCTTTGCAAGGTGACAGCTTTCATTAATTAAACACGGCATGATCCTTAATTTTGTAAATGTGATTATTTGACTAAATATTGTACATTTAACACAATGGATTAACATCACTGTCGTATATTCTGAACTGAATACTCATATCAAGGCCTTGCCACATTACTTAACTGATGAGTGTACAAATGCATGATTAAGGCTGTAACACATTTACGCAGGTCTCATTAATGACCACTTTTCATTGTGAATTTATGTTTTAATTGTACAAATAATTTATAACTAATATATATAGCTACATGAATGAGTGCAATAGTTGTCTTGACCATTCCAGAAAGTTTTCTAGGTTAGTCCCGACTGAATTTGATTGATTCAGAGTAGGGATTGACACCACTGTTTCTTATAACTGTTACTCCGTAAGCTAGAGCCCTTCTTTTAACCTAAATTATTGTCGCTTGATGCTACGAATATAGAGCTATATATTCTCGAGACAATTCCGTTTAATCAGCTGAGCTGTCGTTCTCTCTCCTATAGACAACATTATAGTTTCTTGCGTCAGTTAGAAGTGCAGTCCTCATGTCTGTGCTCGTTCTTTTTTATGCTAAGTGTCATTTTTCAATCATATAAAAATATAAAGATGAATATCTATACTCAGTTGTCTTGTATATATATCAGAAGCGGACTGGTGCAAGAGTTTGTATCATTGAATAGTTTTATATGTATTTTACAGAATGTTAATAAACAGAAGTGATCTGAAAAAAAAAA

Protein: 745 aa

>Pals1_protein

MDSAMVNGYINGRSNGLIHEHAGSHKMAAINSNQYMINQRSDDHINYDSDSDRRPHREMAIDVPENFIGQKKERPSYPPTLTKNSSPRKNPDQHPDQHVAPAQDATADEMQRIRKYQEDLRKRREEEERHLKEEEFLRTSMRGSKKLQSLEEQAQQPGFVNPTYEEDTESRPHSTAYPKKLLAVEDLFASLQNLQKQLRSAEDQKDVAQISKLFSNTRFQQALRIHNKMVEVNLQRHKLSPVADNSQELCGDVMNSLNAAHGHPVAEELRAILQTPNLKMLMYTHDHVATNRARLSASAPNEDDDQEYLYERVSQYNADSIKIVRLHKTAEPLGATVRNNGESVIVGRIVKGGAAEKSGLLHEGDEMLEINGIDIRGKSVNDVCDIMANMTGTLTFLIVPAQDYIADTLSSEPEDKVLHLRALFDYDPEDDIYIPCRELGISFQKRDILHVISQEDANWWQAYREGEDDQALAGLIPSKSFQEQREAMRQTIVNDSKSNKGKKKPFCHACRGGNKKKKQLYGTANDDAEAEDILTYEEVALYYPQPNRKRPIVLIGPPGVGRQELRSRLMTSDQERYGVAVPHTSRPPREDEKDGIDYHFVSRPILEQDIANQKFVEFGEYEKNLYGTSLNAIRAVVQAGKICILNLHPQSLRILKNSDLKPYIVFIAAPNIDKLRTNRIKEKVKFTEVELKEIIEHSRELEENYGHYFDYIIVNFDLDKAYNELLQEINHLEVEPQWVPAQWVL

Domains

L27_N: 184 – 227

L27: 248 – 290

PDZ: 321 – 399

SH3_MPP5: 419 – 480

GuKc: 551 - 726

Clone

Partial ORF

Primers

Forward: ACGAGATGCAGAGGATACGC

Reverse: GGCAAAATGGCTTCTTCTTG

Nucleotide# 821 – 2025 = 1205 nucleotides

>Pals1_clone

ACGAGATGCAGAGGATACGCAAATACCAAGAGGACCTCCGGAAGAGGCGGGAGGAGGAGGAACGCCATCTCAAAGAAGAAGAATTCCTCCGAACTTCCATGAGAGGTTCAAAGAAACTACAATCCCTCGAAGAACAAGCGCAACAGCCGGGCTTTGTGAACCCCACGTATGAAGAGGATACAGAATCACGGCCGCACTCCACAGCATATCCAAAGAAACTCTTAGCCGTAGAAGACCTGTTTGCTTCTTTACAAAACCTGCAGAAACAACTGAGGTCTGCTGAAGACCAAAAAGATGTTGCCCAAATCTCAAAACTATTCAGCAATACTCGCTTCCAACAGGCCCTACGCATCCACAATAAGATGGTGGAGGTCAATCTTCAGCGTCACAAACTGTCACCCGTGGCTGACAATTCCCAAGAACTCTGTGGCGATGTCATGAACTCCTTGAATGCTGCCCATGGCCACCCTGTGGCGGAGGAGCTGAGGGCCATTCTACAGACGCCTAACCTTAAGATGCTGATGTACACACACGACCATGTAGCCACCAACAGAGCACGTCTTTCTGCCAGCGCCCCCAATGAAGACGACGACCAAGAGTATCTTTACGAGAGAGTTTCACAGTACAACGCGGACAGCATCAAGATCGTACGACTCCACAAAACTGCTGAACCATTGGGAGCCACAGTACGCAATAACGGTGAATCTGTAATAGTTGGGCGAATAGTCAAAGGGGGCGCTGCTGAGAAAAGTGGGTTACTTCACGAGGGAGATGAGATGCTGGAAATAAACGGGATCGACATCCGAGGGAAGTCTGTCAACGACGTCTGCGATATCATGGCTAATATGACAGGTACTCTGACATTCCTAATCGTACCCGCACAAGACTACATCGCTGACACGTTGTCCTCCGAACCCGAAGACAAAGTGCTGCATTTGCGAGCGTTGTTTGATTACGACCCCGAAGATGACATCTACATCCCGTGCCGAGAGTTGGGCATCTCCTTCCAAAAGCGAGACATCCTGCACGTGATCAGCCAGGAGGACGCCAACTGGTGGCAGGCGTACAGGGAGGGCGAAGACGACCAGGCGTTGGCCGGCCTCATTCCCAGCAAGTCCTTCCAAGAACAACGTGAAGCCATGAGGCAGACAATCGTGAACGACAGTAAAAGCAACAAGGGCAAGAAGAAGCCATTTTGCCACGCA

Cloning info

PCR rxn on 07/28/16 using GoTaq polymerase mix

| Template | Initial denature | Denature | Anneal | Extension | #cycles | Final extension |
| --- | --- | --- | --- | --- | --- | --- |
| 2 hr cDNA | 4min @ 94C | 30sec @ 94C | 30sec @ 55C | 2min 15 sec @ 72C | 35 | 10min @ 72C |

**Lin7**

Gene model: 1381 nucleotides

ORF: 499 – 1095 = 597 nucleotides

>Lin7_full

GCGTGGCCATGGCATGGGCCTTCTTCATGTGTTTCGCATCTTTCCGTGCTTGCAGCAAGAACTCCTTTTTCTTATCTTCTTCAGCTCGTTTCTTGTGTTCCTCCTCATCCTTTTTGTCCAAAAATGCTCGGACATTAGCGGAGAGAATTCCTTTTTTCTCTTTTTCTATCTTCTTTGCTGCTGACACTTTGGTTGAATAGCGCTGTTGGGATTTAGAAAAATCGTTTAGCCTCTGAGTTTGGGCAGCTAGGCCGATCAATTTCCTGAAATCCATATCTAGACTACGTTCTACAGCAGTTTGAACCCTTCCTGATCCCTCTACACCCTCTTATATGGAACTTTCTCGGCTGAAACTTCACAGAAGATCAAAAACTGAAGAATCCACATCACATCGGAAGTCTTGTTTATTACGGAAATGCGACTTCGGCTAAAATCGGGCTCAGTTCGGCCGTCTTCGTCTCAAGGAAATCGGCCAATCAGAGCGCAGACCAAGTGAAAATGGCTTCCACAGCAGAACCCTTGACTTTGGAAAGAGATATCCAGAGAGCTGTAGAGCTCTTGGAACATCTCCAGAAGAGTGGAGAACTAGCCAGTCCTAAGCTTGCCGCGTTACAGAAAGTCCTCCAGAGTGAGTTCTGCACGGCTGTCAGAGAGGTCTATGAACACGTTTATGAAACTGTGGACATCTCTGGGAGTCCCGAGATCAGAGCTAATGCCACCGCTAAGGCTACAGTTGCTGCGTTTGCGGCCAGTGAAGGCCACGCCCACCCAAGGGTCGTTGAATTGCCAAAGACCGAGGAAGGATTGGGTTTCAATGTAATGGGTGGAAAAGAACAAAATTCTCCGATTTACATTTCGAGAATCATCCCAGGGGGCGTTGCTGACAGACACGGAGGATTGAAGCGCGGAGATCAGTTGTTGTCCGTCAATGGTGTGAGCGTGGAAGGAGAACCCCATGAGAAAGCCGTGGATCTCTTGAAGGCTGCTCAAGGCACAGTAAAATTGGTCGTCCGCTACACCCCCAAAGTCTTGGAGGAAATGGAGGCGAGATTCGACAAGCAGAGAACGGCCAGACGCCGACAACAGATGAACTAGGCGTCAAATATCAATATTATTTACACATATTAAAACTTAAAGAATGGACATTCCGAGGCGTCGAAACTGTATTCTGAATGGTTGAATTGTGGTTGAATCATGTTTATTATAATGTCATAATTGAATGAGTTACATCAAATGCTATTGAATTGAATTCACTTGCCTTGAAAATGTTTGACCAAATTAATTGAATGGTGGATCAGGTCCAATATCCAGAAAAATAGTGTCTGAGATTGTTTTGGTATTTTCTCCAAACAGATTATTGCATCATCTCTTTTTATGGTGA

Protein: 198 aa

>Lin7_protein

MASTAEPLTLERDIQRAVELLEHLQKSGELASPKLAALQKVLQSEFCTAVREVYEHVYETVDISGSPEIRANATAKATVAAFAASEGHAHPRVVELPKTEEGLGFNVMGGKEQNSPIYISRIIPGGVADRHGGLKRGDQLLSVNGVSVEGEPHEKAVDLLKAAQGTVKLVVRYTPKVLEEMEARFDKQRTARRRQQMN

Domains

L27: 13 – 64

PDZ: 91 - 172

Clone

Partial ORF

Primers

Forward: CTAAAATCGGGCTCAGTTCG

Reverse: AATCTCGCCTCCATTTCCTC

Nucleotide# 428 – 1052 = 625 nucleotides

>Lin7_clone

CTAAAATCGGGCTCAGTTCGGCCGTCTTCGTCTCAAGGAAATCGGCCAATCAGAGCGCAGACCAAGTGAAAATGGCTTCCACAGCAGAACCCTTGACTTTGGAAAGAGATATCCAGAGAGCTGTAGAGCTCTTGGAACATCTCCAGAAGAGTGGAGAACTAGCCAGTCCTAAGCTTGCCGCGTTACAGAAAGTCCTCCAGAGTGAGTTCTGCACGGCTGTCAGAGAGGTCTATGAACACGTTTATGAAACTGTGGACATCTCTGGGAGTCCCGAGATCAGAGCTAATGCCACCGCTAAGGCTACAGTTGCTGCGTTTGCGGCCAGTGAAGGCCACGCCCACCCAAGGGTCGTTGAATTGCCAAAGACCGAGGAAGGATTGGGTTTCAATGTAATGGGTGGAAAAGAACAAAATTCTCCGATTTACATTTCGAGAATCATCCCAGGGGGCGTTGCTGACAGACACGGAGGATTGAAGCGCGGAGATCAGTTGTTGTCCGTCAATGGTGTGAGCGTGGAAGGAGAACCCCATGAGAAAGCCGTGGATCTCTTGAAGGCTGCTCAAGGCACAGTAAAATTGGTCGTCCGCTACACCCCCAAAGTCTTGGAGGAAATGGAGGCGAGATT

Cloning info

PCR rxn on 07/28/16 using GoTaq polymerase mix

| Template | Initial denature | Denature | Anneal | Extension | #cycles | Final extension |
| --- | --- | --- | --- | --- | --- | --- |
| 2 hr cDNA | 4min @ 94C | 30sec @ 94C | 30sec @ 55C | 2min 15 sec @ 72C | 35 | 10min @ 72C |

**PTEN**

Gene model: 1484 nucleotides

ORF: 499 – 1482 = 984 nucleotides

>PTEN_partial

CTTCAGGAGTAGGAATTTTAAACGATATCAACTCCAGGAGATCTCAAGAGGCTTCACAGTTCCTCCCTGAACCACAGACTGCCCACTGACATAAGTTTCTGTGTTCTCCACACTGACTTATCACACCCCGTCACGATCACTCTCCGCGGTTATGCGTCCAAGTTTTTCACAGTAAAAGCGCTTTTAACAAGTGTTCATGGTCAATGTTATTCATTCAACACGAAAATAAAGCATGAAGAGCTCCAATCTATAAATACATGTTTAAAAAGGGAAGAAATGTTTGATGAAATGGTTCTCCAGCAGTAGAACTATTTTAATCAAATGACGGGCACTCACCTCTCAAAAGTGTTCATGCGCAGTCGACGCCATTATGGTTTGTTGAAGTAATTTTCAGTTTTCAACTACCCGGTACGTTTTTGTGGAAAACTTCGTGCGTCTGCTTTCTACGATATGGAACCAAGATTCACAGTAAGCCAACATCTCTCTTGCTAGGCCGCCATGGCAAATAAACTTAAGGTGTTGGTCAGCAAGAAGAAGCGAAGGTACCAAGAAGATGGCTTCGATTTGGACCTTACTTATATAAATGAAAAGATAATTGCCATGGGGTTCCCTGCAGAAAATCTGGAGAGCGTCTACAGAAACCACATTGATGACGTTGTAAGATTTCTGGAACAAAAGCATCATGGCCACTACAAAGTTTACAACTTATGCTCAGAAAGGAAATATGACAACACAAAATTCAATAACAGAGTGGCTGAGTTCCCGTTCGATGACCATCACCCTCCGAACCTGGAGCTTGTGAAGCCATTCTGTGATGACTTGGATGAGTGGTTGAAAAGACACAAGAAGAACATTGCAGCTATCCACTGCAAAGCGGGCAAGGGTCGGACAGGGGTGATGATATGTGCCTATCTGCTTCACCAGAAGAAGTTTGAGGAAGCTGAGGAAGCCCTTGAGTTTTATGGGCATGCACGGACAAAGGACAACAAGGGCGTGACTATCCCAAGCCAACGGCGTTATGTGCAGTATTACGGTTATTTGGTGAGGAACAACCTGCAGTACAAGCCAGTCACCTTGAGACTTCATGCTATAGAGTTTATCACAATACCAGTTTTCAACAACAATGGAGCATGCAGTCCGCTGTTCATGGTGTATGGCTCGCAGAAAGACAGGAGGTACAACTCGCGCCCGTATGAGGCGAAGAAAGGAGACGAGAACCTCTTCATGGAACTGGAGGGTTCAGTCATGCTCTGTGGAGACGTGCTCGTAGAGTTCTTCAACAAACCCAAGATGATGAAGAAAGAGCGGATGTTCCACATATGGTTCAACACGTTTTTTATAACCAAGAAAGAAACGCTACCAGCAGCTGCAAACGGGAGTAGCCATTCCTCAGGGAACCTGCACTCTTCCTCCTCATCGCACCACCCTCAACTGCATCCCGTACAACATCAAAGTCACCATCACCACAACCAGCATCACTCATA

Protein: 327 aa

>PTEN_protein

MANKLKVLVSKKKRRYQEDGFDLDLTYINEKIIAMGFPAENLESVYRNHIDDVVRFLEQKHHGHYKVYNLCSERKYDNTKFNNRVAEFPFDDHHPPNLELVKPFCDDLDEWLKRHKKNIAAIHCKAGKGRTGVMICAYLLHQKKFEEAEEALEFYGHARTKDNKGVTIPSQRRYVQYYGYLVRNNLQYKPVTLRLHAIEFITIPVFNNNGACSPLFMVYGSQKDRRYNSRPYEAKKGDENLFMELEGSVMLCGDVLVEFFNKPKMMKKERMFHIWFNTFFITKKETLPAAANGSSHSSGNLHSSSSSHHPQLHPVQHQSHHHHNQHHS

Domains

PTPc_motif: 91 – 143

PTEN_C2: 188 - 281

Clone

Partial ORF

Primers

Forward: GTTCCCTGCAGAAAATCTGG

Reverse: TCCTTTCTTCGCCTCATACG

Nucleotide# 606 – 1209 = 604 nucleotides

>PTEN_clone

GTTCCCTGCAGAAAATCTGGAGAGCGTCTACAGAAACCACATTGATGACGTTGTAAGATTTCTGGAACAAAAGCATCATGGCCACTACAAAGTTTACAACTTATGCTCAGAAAGGAAATATGACAACACAAAATTCAATAACAGAGTGGCTGAGTTCCCGTTCGATGACCATCACCCTCCGAACCTGGAGCTTGTGAAGCCATTCTGTGATGACTTGGATGAGTGGTTGAAAAGACACAAGAAGAACATTGCAGCTATCCACTGCAAAGCGGGCAAGGGTCGGACAGGGGTGATGATATGTGCCTATCTGCTTCACCAGAAGAAGTTTGAGGAAGCTGAGGAAGCCCTTGAGTTTTATGGGCATGCACGGACAAAGGACAACAAGGGCGTGACTATCCCAAGCCAACGGCGTTATGTGCAGTATTACGGTTATTTGGTGAGGAACAACCTGCAGTACAAGCCAGTCACCTTGAGACTTCATGCTATAGAGTTTATCACAATACCAGTTTTCAACAACAATGGAGCATGCAGTCCGCTGTTCATGGTGTATGGCTCGCAGAAAGACAGGAGGTACAACTCGCGCCCGTATGAGGCGAAGAAAGGA

Cloning info

PCR rxn on 07/28/16 using GoTaq polymerase mix

| Template | Initial denature | Denature | Anneal | Extension | #cycles | Final extension |
| --- | --- | --- | --- | --- | --- | --- |
| 2 hr cDNA | 4min @ 94C | 30sec @ 94C | 30sec @ 55C | 2min 15 sec @ 72C | 35 | 10min @ 72C |

**Coracle**

Gene model: 2651 nucleotides

ORF: 309 – 2633 = 2325 nucleotides

>Cora_full

ATTGTGTGCAAAATAATGACAAAATGCTTAATCCAAAAGCTGAAATTAATTTAAAAAGTAATGACGACAAGCTTGTTGTTCTAAAGAGCAGTTGTGTGTCAATTTAACTGTTTTCAATTCAAACAATGCTTCTGGAAGCTCAGGTAAACGCGGATGAATCTACTACCTTCACTCAAGCGTGGCCAGGTGTCTGCAAGAGTTTGACCTAGTTTCCACTCAGGTGAAAAGTTTAATGGAAGTGTGACAACTAAAACAACAAGGAATAAGGAGGCAGTCATAGTTTTGGACTTGGGTCGTAAAAACGTACGATGGGTACGGAGAAAAACAACATGCACGATAGTGATGAGGACGTTCCGGGTGAGGCGAACGACGGCCAAACGGCTGGTGACGGCACCAAAGAAGAAATCGAACTAACTCCCTTACCAGGGGACCCCAAGCAGGAGGAGAAGAAACGCAAGGAGGAGGAGAAGAAGAGAAAAGAAGAAGAGAAGAAGGAGAAGAAGCGACTTGAACAAGAGAAGAAGCAGGCGCAGAAGCAAGCCAAGTCTTCTGCCAAATCTACACCGACCAAATCTCCCAAGAAAACGCAGGCCTCCAAAGGGGCCAAACCTTCTTCCGGCATGGTTCTTTGCAGAGTACTTCTTCTTGACCAGACCAACTTCGAGGTCGAAATTGACAAAAAAGCTAAGGGACAGCAACTCATCGACAAATGTTGTGAACACTTGAACATTTTAGAAAAGGACTATTTCGCTATCAATTTCAGAGATGCCAGTGACATTAAGTTCTGGGTCAACGCTGAGAAGAGGATTTCAAAACAGATCGGGAATGGCCCATGGGTCTTTGGTTTTGAGGTGAAATTCTACCCCCCAGACCCTGCCACCCTTCAGGAGGACCTCACTCGTTACCAGCTTACTCTGCAGACAAGGAAAGATATCCTGTCAGGAAAGCTCCCATGTTCATTCGTCACCCACGCCCTCCTGGGCTCCTACATCGTGCAGTCTGAGTTTGGAGACTATGACGTTGAGGAACATGGAGCTGGAATCGAGTACATCGCAAATCTCCACTTTGCTCCCAACCAGAGTGAGGAGCTGCTGGAGAAGATTGCCGACCTCCACAAGACACACAGAGGACAGACACCAGCCGAGGCTGAACTCCACTACTTGGAGAACGCCAAGAAGCTGGCCATGTATGGAGTGGACCTGCACCAGGCCAAGGACAGCGAGAATGTTGACATCATGATCGGCGTCTGCGCCTCTGGACTTCTCATCTACAGAGATCGTCTTCGTATCAACCGCTTCGCATGGCCCAAGATCCTCAAGATTAGCTACAAGAGGAACAATTTCTACATTAAGATTCGTCCTGGAGAGTTTGAACAATTTGAGAGCACAATTGGCTTCAAGCTGGCCAATCACCGTCTGGCTAAGCGTCTCTGGAAGACTGCTGTGGAGCACCACACTTTCTTCAGGCTGAGGGAGCCAGAGCCACCAGCGAAGAGTGGTTTCTTCCCCAGATTCGGATCCAAGTTCCGTTACTCTGGACGCACCCAATACCAGACCAGACAAGCTGCAGCCATGATCGACCGTCCCGCCCCTAACTTTGAGCGGGGAGCCAGTAAGCGTTTCACTGGTTCGCGTAGCATGGATGGAGTTTCAATGGGTTCTGCCACCCTTGGCCGAGGAGGCCCTCTAGGGAGTGTCTCAGAACCTGTCCTCAATGAAACAGGAGTTCACGGCGGCCATGGCTATGTGGAGAGAGCCGAACTCTACCAACCAGACAACTCCAGGACAGCAACACTCGACTTGAAGGGGCGCCGCAAACCAGGCGGCTCAGTCCCCATGGCCGACTACGATGACGACCGTTACCCCCACGGAATGGACCCCAGCGCTTATGACCACGATGACGACGCACAGGTATCTGTAATTGCCGGCACGGGACCCGATGAGAACCGAGTAGCGTATGTAAAGAATGTCAAGCATGGTGGCCCTTACGATCCCAACGCCTCTCAGGATGGTGATGGGTACGGTTACCCAGGCGGTCCTGGATCCATCGGCGGCTACCCTGGACAAGGAGGTTACCCCGGTTCCCCAGCCGGACAGGGAGGTTATCCCGGTTCCCCGGCCTACCCTAACCAAGACGACCATTTGCGGGAACGCGATGGTTACCCCGGCGGTTATGGAGGAGATGGCCAATGGGACCCTAACTACCCTGGCGGTCACCGAACCGTCACCACCACACAACGCGAACCCCCTACTGTGAGGACAGAAGCTTTCAAGTATGACCCCACTGTTGAATCTCGAACGCAGTCAACACGGGACGTACCTTTTGTAAAAACAGAGACGAGAACAGTGACTTATGAATCAGACCGGGCCAATGAGGGCGACGATTTCCCGGGTATTCTAGTAAGTGCCCAATCGCACAGCTCTCGTTCCCAGACCGTCGATACGACAACGTATAAAACCGAAAAAGACGGCGTTGTAGAAACCAGAATTGAACGTAAAATGGTAATCACAACGGAAGGTGAAGAAATCGACCATGACGCTGCATTGGCAGAAGCTATCCGTTCAGTAACAGAAATGGACCCTGACCTTTCTGTGGAAAAGATTGAAATTAAGACAGAAAGTGAGGCACAATAATAGCCCTGAATGAACATA

Protein: 774 aa

>Cora_protein

MGTEKNNMHDSDEDVPGEANDGQTAGDGTKEEIELTPLPGDPKQEEKKRKEEEKKRKEEEKKEKKRLEQEKKQAQKQAKSSAKSTPTKSPKKTQASKGAKPSSGMVLCRVLLLDQTNFEVEIDKKAKGQQLIDKCCEHLNILEKDYFAINFRDASDIKFWVNAEKRISKQIGNGPWVFGFEVKFYPPDPATLQEDLTRYQLTLQTRKDILSGKLPCSFVTHALLGSYIVQSEFGDYDVEEHGAGIEYIANLHFAPNQSEELLEKIADLHKTHRGQTPAEAELHYLENAKKLAMYGVDLHQAKDSENVDIMIGVCASGLLIYRDRLRINRFAWPKILKISYKRNNFYIKIRPGEFEQFESTIGFKLANHRLAKRLWKTAVEHHTFFRLREPEPPAKSGFFPRFGSKFRYSGRTQYQTRQAAAMIDRPAPNFERGASKRFTGSRSMDGVSMGSATLGRGGPLGSVSEPVLNETGVHGGHGYVERAELYQPDNSRTATLDLKGRRKPGGSVPMADYDDDRYPHGMDPSAYDHDDDAQVSVIAGTGPDENRVAYVKNVKHGGPYDPNASQDGDGYGYPGGPGSIGGYPGQGGYPGSPAGQGGYPGSPAYPNQDDHLRERDGYPGGYGGDGQWDPNYPGGHRTVTTTQREPPTVRTEAFKYDPTVESRTQSTRDVPFVKTETRTVTYESDRANEGDDFPGILVSAQSHSSRSQTVDTTTYKTEKDGVVETRIERKMVITTEGEEIDHDAALAEAIRSVTEMDPDLSVEKIEIKTESEAQ

Domains

B41: 108 – 299

FERM_C_4_1_Family: 294 – 387

FA: 399 – 439

4_1_CTD: 669 - 772

Clone

Partial ORF

Primers

Forward: AAGCAAGCCAAGTCTTCTGC

Reverse: CCAGAGACGCTTAGCCAGAC

Nucleotide# 534 – 1433 = 900 nucleotides

>Cora_clone

AAGCAAGCCAAGTCTTCTGCCAAATCTACACCGACCAAATCTCCCAAGAAAACGCAGGCCTCCAAAGGGGCCAAACCTTCTTCCGGCATGGTTCTTTGCAGAGTACTTCTTCTTGACCAGACCAACTTCGAGGTCGAAATTGACAAAAAAGCTAAGGGACAGCAACTCATCGACAAATGTTGTGAACACTTGAACATTTTAGAAAAGGACTATTTCGCTATCAATTTCAGAGATGCCAGTGACATTAAGTTCTGGGTCAACGCTGAGAAGAGGATTTCAAAACAGATCGGGAATGGCCCATGGGTCTTTGGTTTTGAGGTGAAATTCTACCCCCCAGACCCTGCCACCCTTCAGGAGGACCTCACTCGTTACCAGCTTACTCTGCAGACAAGGAAAGATATCCTGTCAGGAAAGCTCCCATGTTCATTCGTCACCCACGCCCTCCTGGGCTCCTACATCGTGCAGTCTGAGTTTGGAGACTATGACGTTGAGGAACATGGAGCTGGAATCGAGTACATCGCAAATCTCCACTTTGCTCCCAACCAGAGTGAGGAGCTGCTGGAGAAGATTGCCGACCTCCACAAGACACACAGAGGACAGACACCAGCCGAGGCTGAACTCCACTACTTGGAGAACGCCAAGAAGCTGGCCATGTATGGAGTGGACCTGCACCAGGCCAAGGACAGCGAGAATGTTGACATCATGATCGGCGTCTGCGCCTCTGGACTTCTCATCTACAGAGATCGTCTTCGTATCAACCGCTTCGCATGGCCCAAGATCCTCAAGATTAGCTACAAGAGGAACAATTTCTACATTAAGATTCGTCCTGGAGAGTTTGAACAATTTGAGAGCACAATTGGCTTCAAGCTGGCCAATCACCGTCTGGCTAAGCGTCTCTGG

Cloning info

PCR rxn on 05/05/16 using GoTaq polymerase mix

| Template | Initial denature | Denature | Anneal | Extension | #cycles | Final extension |
| --- | --- | --- | --- | --- | --- | --- |
| 2 hr cDNA | 4min @ 94C | 30sec @ 94C | 30sec @ 55C | 2min 15 sec @ 72C | 35 | 10min @ 72C |

**Yurt**

Gene model: 4172 nucleotides

ORF: 499 – 3834 = 3336 nucleotides

>Yurt_full

TTCGGGTTTTCCATCCCGCAGCTGTTGCGTAACAGACTGGTAGTAATCGAGCAAAAGAGTGTTTGTGAGGAACGGAAAAGTCCCAACCACGCAAAGAGCTCCTGCGGCCAAGTACTGAGATTTCGCCAACACACGGTTGAGCAGTGCGGCCATTTCTAGTTCAGCCTCGTAAAATAGCTTAAATCGGTGTAGTTCTTCTGGTTTTGCCAAAAACGCCCTTGAAAAGTTTGACAGCTCACTGGTCGGCGTGCTGCATGGGCGCAGACATGTTGGATTGGGAACGAGGCCGCTTCCGGTGACGTCACGGCCAGATACGCAGGTGCGAAAAAGTTGTGAACGGGGAAGATGAGGTAGGGAGTTGCGCCAACATTTCTGCGCTAAAACAGTGGGGATTCTACGTTTGGATGTTTACCAATAAATTCACCAAGATCTTCTCCTGGTTCCCGAACCACACAGTTCATCCATGAGTTAACGCAGGGCTTCTGAAGCGACAGAAAAATGTCGAAATTTTTACGATTCTTGAGCCGCCGCGGTGGCTACAAAAACAGAGATGGACTCCAAGGCGAAAAGCCCAAGAAAGGAATTCTACCTTGTAAAGTCATTCTCCTGGATGGGACAGACATGTCCGTGGATGTTTCAAAAAAGGCCAAAGGAGAAGAATTGTTGGAGCAAGTCTACTATCATCTTGATCTCATAGAAAAAGACTATTTCGGACTGCAGTTCACAGATTCAGCAAATGTCAATCATTGGCTGGACCCCAGCAAGACAGTAAAGAAGCAGATAACGATTGGTCCCCCCTACACCTTTCGTTTACGAGTCAAGTTTTACTCATCCGAACCTAATAATTTACATGAAGAATTGACCAGATATCAGTTTTTCCTGCAGGTGAAACAGGACATACTAGACGGTCGAATAGAGTGCCCGTTTGAAGCAGCCGTTGAATTAGCAGCTTATGCTTTGCAATCTGAGTTGGGCGATTATGAGGAGGATGAGCACTCACCTGGGTTTATTTCTGAGTTCCGATTTGTTCCGGACCAGACTGAAGAAATGGAGCTGGCCTTCGTTGAAAAATTCAAGACTTGCAGGGGAATGACACCCGCTCAGGCTGAACTCAGTTACCTAAACAAAGCCAAATGGCTAGATACATATGGTGTGGACATGCATACAGTCCAGGGGAGAGATGGAATGGACTACCATCTAGGACTGACTCCCACTGGAATCTTAGTATTTGAAGGAGAGCAAAAAATAGGACTGTTCTTCTGGCCCAAAATCACCCGACTAGACTTCAAGAGAAAGAAGTTGATACTCGTAGTGGTGGAGGATGATGAACAGGGACGGGAGCAGGAGCATACATTTGTGTTCAGAATGTTACATGAGAGAGCATGCAAGCATTTGTGGAAGTGTGCGGTGGAACATCATGCGTTCTTCCGACTGCAAGGACCTGTCAAGCACCAGTCCTCTCGGCAGAACTTCTTGCGAATGGGATCGAGATTCAGATACAGTGGGAGAACAGAATTCCAGACGGCCAGCATGAACAGAGCCAGAAGAAGCGTAAGATTTGAAAGAAGAGCTAGTCAGAGATATTCACGGCGACCGACCTTTGAAAGGCAGGACACCACCAGAAGCGTTAAGGTCACTGATATCCGAGAAAGTCAGAGACCTCAACCCAGAGCTAGACCGAGAATTAGCAGTTCATCGAGAACGCTAAGTAAGCCAGTAGCCACGCCAGCGGCTGCTCCCCAAAAGACTGTGATAGACGACCGTGTGCCTGTTATGCCAATTGAGACTCCGGTCGATGGCCCAGCCAGTCCTGCGCCTGCCTCCCCTTCTCCTGCATCCGCAATGGAAAGACTGGACAATCTCATTAAAGGAACCCCTCCTGCTTCCCACATCAACCAGAGACCCCCTTCACAGAGTTCGGAGGCCTCCTCTCTACCGAGAAGTTCCACATCTTCACAGCCGCAGGATGTCTCTCTAAGAGATGCGTCAGAAGCTGCTCAAGCTAAACTGAAAGGTCTTGATGAGAAAAAACCAGTCCAAGCCAAAAAAGTTCAGAAAGATGTGAATACTTTCATAAATAACCAACTCAAGTTTGTCAACACAGCCACTACCATTCCGCCAGAACAGATGAAGTGCAACATACTCAAAGCAAAGGCTGAGGAGAACTACAGAAAAGGTGTGAACACTCCCGAGAAAGGACTTACAGTAGAAATGCCAACCTACTCTGATGAAATAGAAACTGCAAAGGCTGCTGCAGCAGCAGCTTGCCCATCACCTGAACCTGCATCGCCCGCGGCAACCGCAGCATCAGGTGCACCAGCGCTAGTTGCTGCGTCTGCACCAGCACTAGTTGTTGTGTCTGCACCAGTCGCTGTATCAGTCGCTGTACCAGTACCAGTTGCATCAATTGCTGCATCTCCAGTTGTCCCAGCAGCAGCAGCCGCAGCAACGGAAACAGTATTTGAACATGTTGAACCATCACCAACTCCTGAAGAACCAGAATCCAATGATGAGTCCCCTGTTGCGGGTAAGGATCAGTCATTGCCTGAGGAGAAGATGAGACTGATTAAAACTGCTAATGTCACAACAAATGGGTATTCTGCGGCTGATGCTCCTGTCATTCCCATCGTCCCGAAGTCGCCAGTCATGCCGATTACTCCTGCTCCAGCTGATAAGCAGCCGACAAGCTCGGGTTCAGCCAAACCAAAGAAAAAGAAAAAAGCAAAGTCTAAGACACCCAAGGTATCGGAAACGGTGCCATTGGTGTCTGCGGAGGATATTGGCGAGGGTTTATCACCCACGGAGAATTCGCCCGGATCCGAGAAGGCTAGCTCCCCTGAGCAGGAGACTGACCTCACTGAGCCTGCTGCCCCCTCATCCACTAGTGCAGCCTCTGCCAAAACTAATGGAGAAATATTAATCGACTATACGCCTACTTCTGTTAAGTCTAACACTGGAACAAGTGGCGGGGCAAAGCCAAAGACCTCTAATAATCCTTTCATTAATGATCTGTCAGCAGAGAGCAATGGTATCAAGGTCAGCAAATCTAACAATCCTTTCTATAATGATCTGAATGCTGCTTCTTCCCATACAAGCACGACCAATCCTTTCTCAGCTTCTTCTTCATCTATTAAAGCTGAATCTCCTAAGACAAAAGAACTTAACACTTATTCTGCTGGCAAGAGCAGCAAGACTAATGTTAAGCCTCTGCCTAAGCCTGTTGCTGCTCCTCGTACTACTACTAAAGCTGCAAGTGCATCTCCTGTACTCAAGCCTAAGCCTGTTCCTCTCAGTCGCCCCAGCACGGCTCCTGTGCTTCTAATTAAGCCTGCTTCTAGCTCGTCACTGTCGAATAACAATAAAGTCATTCCCGACTTGCCGGTGATCGGCAAGCCTGCCCATGAGTTTGGCATCAAGAAGAGAGAGGAGGAAGAAACCAGTCTGATGATTGCTTCGACTGATTCCACTGCTTCCTTAGAACTACCGCCTTCTCCTCCACGCCCTGCCCCTGTCTCTCGACCCCAACCCTCGCCTCCTGCTGCCTCCGAAGACTCGCCCCGGGGAGCGTCCAAGGAACGCATGGCCAAGGACAGCATGGTCATTGAGACCTCCTTCTCATCTGGAAACGCTGTCACGAGAAAAGTGTCGAAGAGTGTCAAATCAACGGGGAACTCCGAACAGACAGAAGTTATAATGGTCACGAAGGAACCCAAGAAGAAGAAGAAAAAGTCTGCAAAAACTGCCGCCACAGAAGAATTGTCGCCCTGGCATGTGACGATTGACAATGATAACAAACCAAAGGAACGAAGGGTGGCACTCACGACTGAGTTGTAGGCCTAGTTATCATTACACTGTTGAAAGACTGTTGAAGGACTATTAATTAGTCTGACATTTGTAAATTATTTCACTTATTTTTGTAAAGTTGCAATGATGCCTTAAATACTAAGTGTTACGGTTGCTCTTATCATTTATACACTGGATTGTGTAACTGCTTTAAATAGTTTTACTGTTGGTCTTAAAATGTGTAAGAAATTATGGATATGGTTTTAAATCAGAATCTACACGCTTATGGAGTGATCCATATTTTACATCAGCTTGGTACTTTCAGTTCTTATGTTGTTATGCCATGGTAAAGGTGCTACGTTTAATTTGATTCGGTCATACAGTAGTTG

Protein: 1111 aa

>Yurt_protein

MSKFLRFLSRRGGYKNRDGLQGEKPKKGILPCKVILLDGTDMSVDVSKKAKGEELLEQVYYHLDLIEKDYFGLQFTDSANVNHWLDPSKTVKKQITIGPPYTFRLRVKFYSSEPNNLHEELTRYQFFLQVKQDILDGRIECPFEAAVELAAYALQSELGDYEEDEHSPGFISEFRFVPDQTEEMELAFVEKFKTCRGMTPAQAELSYLNKAKWLDTYGVDMHTVQGRDGMDYHLGLTPTGILVFEGEQKIGLFFWPKITRLDFKRKKLILVVVEDDEQGREQEHTFVFRMLHERACKHLWKCAVEHHAFFRLQGPVKHQSSRQNFLRMGSRFRYSGRTEFQTASMNRARRSVRFERRASQRYSRRPTFERQDTTRSVKVTDIRESQRPQPRARPRISSSSRTLSKPVATPAAAPQKTVIDDRVPVMPIETPVDGPASPAPASPSPASAMERLDNLIKGTPPASHINQRPPSQSSEASSLPRSSTSSQPQDVSLRDASEAAQAKLKGLDEKKPVQAKKVQKDVNTFINNQLKFVNTATTIPPEQMKCNILKAKAEENYRKGVNTPEKGLTVEMPTYSDEIETAKAAAAAACPSPEPASPAATAASGAPALVAASAPALVVVSAPVAVSVAVPVPVASIAASPVVPAAAAAATETVFEHVEPSPTPEEPESNDESPVAGKDQSLPEEKMRLIKTANVTTNGYSAADAPVIPIVPKSPVMPITPAPADKQPTSSGSAKPKKKKKAKSKTPKVSETVPLVSAEDIGEGLSPTENSPGSEKASSPEQETDLTEPAAPSSTSAASAKTNGEILIDYTPTSVKSNTGTSGGAKPKTSNNPFINDLSAESNGIKVSKSNNPFYNDLNAASSHTSTTNPFSASSSSIKAESPKTKELNTYSAGKSSKTNVKPLPKPVAAPRTTTKAASASPVLKPKPVPLSRPSTAPVLLIKPASSSSLSNNNKVIPDLPVIGKPAHEFGIKKREEEETSLMIASTDSTASLELPPSPPRPAPVSRPQPSPPAASEDSPRGASKERMAKDSMVIETSFSSGNAVTRKVSKSVKSTGNSEQTEVIMVTKEPKKKKKKSAKTAATEELSPWHVTIDNDNKPKERRVALTTEL

Domains

B41: 32 – 222

FERM_C: 227 – 315

FA: 324 – 364

Clone

Partial ORF

Primers

Forward: GAGTTAACGCAGGGCTTCTG

Reverse: GCTCTGGGTTGAGGTCTCTG

Nucleotide# 466 – 1673 = 1208 nucleotides

>Yurt_clone

GAGTTAACGCAGGGCTTCTGAAGCGACAGAAAAATGTCGAAATTTTTACGATTCTTGAGCCGCCGCGGTGGCTACAAAAACAGAGATGGACTCCAAGGCGAAAAGCCCAAGAAAGGAATTCTACCTTGTAAAGTCATTCTCCTGGATGGGACAGACATGTCCGTGGATGTTTCAAAAAAGGCCAAAGGAGAAGAATTGTTGGAGCAAGTCTACTATCATCTTGATCTCATAGAAAAAGACTATTTCGGACTGCAGTTCACAGATTCAGCAAATGTCAATCATTGGCTGGACCCCAGCAAGACAGTAAAGAAGCAGATAACGATTGGTCCCCCCTACACCTTTCGTTTACGAGTCAAGTTTTACTCATCCGAACCTAATAATTTACATGAAGAATTGACCAGATATCAGTTTTTCCTGCAGGTGAAACAGGACATACTAGACGGTCGAATAGAGTGCCCGTTTGAAGCAGCCGTTGAATTAGCAGCTTATGCTTTGCAATCTGAGTTGGGCGATTATGAGGAGGATGAGCACTCACCTGGGTTTATTTCTGAGTTCCGATTTGTTCCGGACCAGACTGAAGAAATGGAGCTGGCCTTCGTTGAAAAATTCAAGACTTGCAGGGGAATGACACCCGCTCAGGCTGAACTCAGTTACCTAAACAAAGCCAAATGGCTAGATACATATGGTGTGGACATGCATACAGTCCAGGGGAGAGATGGAATGGACTACCATCTAGGACTGACTCCCACTGGAATCTTAGTATTTGAAGGAGAGCAAAAAATAGGACTGTTCTTCTGGCCCAAAATCACCCGACTAGACTTCAAGAGAAAGAAGTTGATACTCGTAGTGGTGGAGGATGATGAACAGGGACGGGAGCAGGAGCATACATTTGTGTTCAGAATGTTACATGAGAGAGCATGCAAGCATTTGTGGAAGTGTGCGGTGGAACATCATGCGTTCTTCCGACTGCAAGGACCTGTCAAGCACCAGTCCTCTCGGCAGAACTTCTTGCGAATGGGATCGAGATTCAGATACAGTGGGAGAACAGAATTCCAGACGGCCAGCATGAACAGAGCCAGAAGAAGCGTAAGATTTGAAAGAAGAGCTAGTCAGAGATATTCACGGCGACCGACCTTTGAAAGGCAGGACACCACCAGAAGCGTTAAGGTCACTGATATCCGAGAAAGTCAGAGACCTCAACCCAGAGC

Cloning info

PCR rxn on 05/05/16 using GoTaq polymerase mix

| Template | Initial denature | Denature | Anneal | Extension | #cycles | Final extension |
| --- | --- | --- | --- | --- | --- | --- |
| 2 hr cDNA | 4min @ 94C | 30sec @ 94C | 30sec @ 55C | 2min 15 sec @ 72C | 35 | 10min @ 72C |

**Scribble**

Gene model: 9295 nucleotides

ORF: 214 – 4785 = 4527 nucleotides

>Scribble_full

CCGCGAGATGTCGCACATGCATGAGACGTGTCGTGCGTGACGTCACGAGGCTTTCGCCAGTGGACGATTTTTGAGGAACGGAGGATTTGATGAGCTGACCTAGAAATCTTCTCAGTGAAATTATCCCAATGTCCTGGCTTACGCGGCCTTGAGTTTGTGTTTATAAGATCTGTGAGGATTTGATCTTATGTTGGAGACAAGGCAGGAGTCGCCATGCTGAAGTGTTTCCCGCTATGGCGGGCTTGTAACCGGCAAGTTGAATACATTGACAAGCGCCATTGCAACTTGACGTCGGTGCCAGAAGATGTTTTGAGGCACACTCGGACCCTAGAGGAATTATTACTGGATGCCAATCAGATCAGAGAACTACCCCGGGGGCTCTTCCGCCTGCTTCAGCTGCGAAAACTCGGCCTCTCTGACAATGAAATCGGAATTTTACCACCGGACATTGCCAATTTTATGAACCTAGTAGAGTTGGACATCAGTAGAAATGATATCTTCGACATTCCCGAAAACATTAAATTTTGTAAAAACCTCCAAGTCGTTGACTTCAGCAGTAATCCCATCACAAAGTTACCGGATGGATTTACCCAATTGCGGAATTTGACGCATCTCAGTCTAAACGATGTTTCCCTCGGGAGACTTCCAGCCGATATCGGCAGTTTATCAAACCTGGTCTCCCTGGAGCTTCGGGAGAACTTGCTCAAGTATTTGCCGGCGTCTATGTCCTTCTTGGTGCGCCTCAAGAGCCTCGATCTGGGCAGCAACTCTTTGGAAGAACTGCCGGAAACCATCGGAGCCCTGCCCAGTCTACAGGAACTATGGCTGGACTGCAACGACCTGGTGGAGCTGCCCAAGGAGATTGGCAACTTGAAGAAGTTGACGCAGTTGGACGTGTCGGACAATAAACTGGAGATGTTGCCGGAAGAGTTAGCCGGACTCTTGGCCCTCACGGATCTCCACCTCTCACAGAACCAGATAGAGTATCTCCCCGATGGTATAGGCAATTTAAGAAAATTGAGTATATTGAAAGTGGACCAGAATCGGTTGATGGAACTGACGCCAACAATAGGAAATTGCGAATGTTTACAAGAAGTTGTCCTAACAGAAAATTATTTATCGGAATTACCAGCGTCAGTTGGACGGTTAAAGAACTTATCGGTTTTAAACGTCGACAGAAATCGACTGGCAGATTTACCCAAAGAGGTCGGCCGGTGTGGAAAGATGGGCGTCCTCTCGTTAAGAGACAACCGCATTATGTATCTTCCTGCAGAAGTCGGCAATCTGAAAGAACTTCATGTTCTTGATGTTTCCGGAAACAGGCTCCAATTCCTCCCGATCACAATCCTCAACTGCGACCTAAAAGCCGTCTGGTTATCGGAGAACCAAGCGCAACCAATGATGCGATTCCAGACAGACACGGACGACAAAACGGGCAACAAAGTCTTGACGTGTTTCTTGTTGCCGCAACAGGCCTACCAGACGGAGAGTATGGAGAATATGTTGATGGGTAGCATTGCCACAGACCAAGACAGTCGCTTGAGTTGGAACGAGGTGCACAGTCGCTCGGCCGTGAAGTTCGCAGGAGAGGAGGATGGAGAGCTGCCCACGGATGACGAGGAAGAGAGTCACTTTGTCCGACACAACACCCCACATCCGAAGGAGTTGAAAGCTCGACACGCCAAACTTTTTAACAAGTCAGAAAACGCCGAAGGGCAAGCTGCACCACACAGCCAGAAAACTACAGCTAAAGAGGACGTCACGTTTATGCCGTCTAGGGAAAAGGACACCAGGTGGAGCACGGAGTCACAGAGCAGCGAAAGTTCCAAAACATCTGGTCTCAGTAAGGCCTCAACTGCAGTCAAAACGCCCTCTGTTGACCACACGATGACCCCACCCAGTCCGCCCCAAGAGGTGCAGGAGACGGCCCCCGTCAGGTCACATGTTCACATCACAAACTCGGTCACTTTGATCGACAACCACGCTCAAGAGACCGCCCTTGATGAGGAATTTGATTCGTCGAGATCTGACGCAGACTACGGAGACAAACACATAGGATTTGCAGATGATACGGAAGACCACATGTCGGAGGGAAAGTTGCGGAGGCGAGACACCCCTCATCACTTAAAGAACAAGAGGATCAACATGACGACGGCTAAGGACGATGCAGAGGAGAAGGTCAGGGCTATTCTGTCACAGGCCAGTCCTGGGCCAAAGCTGCAGCCCTCCCTGGATAGACCTAAGCGAGACTCAACAGATGGCGGCGTAGTGTTGACGCGACAGGTCGCCATGGAGATCAGAGAAGAGGAAGCAACCGTGAAAATAGTCCGAGAACCGGGACAAGGCCTCGGGATTAGCATCGCGGGAGGAATTGGTTCGACCCCTTACAGAGGGGATGACGAGGGGATTTTCATCTCGAAAGTTACGGAGGACGGGCCAGCTGGCATGGCCGGGTTGATGGTCGGTGATAAAGTTATATCGGTGAACGAAAACAACATGGTGGAAGTGGAACACCAGAGGGCAGTGCAAGTGCTGAAAGAGGCGGGAAACATGGTTACTATGGTGGTAACTAGGGAAGTACTTAACCAGGAGAAACACAAAGCAGCAATCAGCGCGAGCCGAGACAAGTTGGAGCAGATCGGAAGCGAGTCGTTGGACCCAGAGTTACAGATTAGGACCGAGTCAATTTCGATGATCCTCAAACGGGATGACAATGGATTGGGATTCAGTATAGCAGGCGGAAGAGGCTCCACGCCTTACAAGGGCTCCGATGAGATCAAACAAGAGGAAATTGCCCCCACCACCCAAGGCAGGGATTCAATTTACATATCACGAGTAACGGAAGGCGGAGCCGCAGAGAATGATGGCAGAATACAAGTTGGAGATAGGATTATTTCGATAAATGCTGTGGACGTCTCAGACGCTCGCCACGACCAAGCAGTGGCCCTATTGACAAGCAGCGGAGACGAAGTCGCCCTTATTCTCCACCGAGAGATCTTACTAGATTCAAACGGGGACGTGGAGGAGCATGAGAGGGTGCCTAGCCCCACCCCTTCTCCCCAGCCCCAGATCACATCTTACGCTCCCGCCCCCCTTCCCCCCGTGAGCAACCCCCCTGGCATGGAGACACACATCACGAGGAGTAGCCCTGCTCATAAGGTGTCACCAAAACCAGACATTGCACCGCGATCAGTTTTAGGAGAGATGCCCTCAAAATGGGGTCCCCCTCCCCCCGCGCCTCCCCGGACTCAGAGCGTCCCTTTACCCTCTCCTAAACCCGCACCCGTTGTGGCGGGTAAGGGTAGTAACTCTCCTAAACCCGCAGTTTCCCCGCGCGGGTCAAGCCTTGTCGAACAGTTCAATATGTTAGCCAAAAAAACCCCCGAAGGCGCCCCTCGAGTTGAAGGGGGAATCCCGAGGAAATCCCCTGGGTCTTCCCCCGCCAGGTCCTCCCTCTCCAGCGACCTCAACCACCAGGTGTCTGAATCTCACGATGATAAGTTCCCCACCGAGATCATCACAATAGTGAAAGCGGGTGGGCCCCTTGGCCTGAGCATAGTAGGAGGAACGGACCATACTAGTCATCCTTTCGGTATTCACGAACCAGGAATTTTCATCTCTAAGATAGTGCCTGATGGAGCTGCTGCCAAAACCCAACTTAGCATCGGGGATCGCATTCTTTCTGTGAACGGCAAGGACGTTACGGGTGCTAGCCACCAGGAGGCCGTCATGTCGCTCATCGCCCCCACCTATGAGATAGTTCTGGAAGTACGACACGACCCGCCTCCACCAGGGCTAATAGAATACAAGCTGACGAAAACCCCTGGGGAGAAACTGGGCATTAGCATAAGAGGGGGCGCCAGGGGTCCCCCTGGTAACCCCCTTGATTCAACCGATGAGGGCATATTCGTAGTAAAGATTCAACCCAATGGTGCTGTAGCCCGAGATGGAAGGCTTAAAGTAGGACATAGAATTTTAGAGGTCAACGGCCAGAGTTTACTAGGAGCAACCCACCAGGAGGGCGTCCGAGCTTTACGAAGTGTCGGTGACAAGATCAGCATCATGGTGTGCGAAGGATTTGATCCAAACCAAGTGGATAGCACGTTACTAGCCATCGCTCGGACGGAATCTGTGTCCTCTGTGGACAGGGACGACGAAGATATGTTGATAATTCAGAAAGAACAGGAGATGCTGAAGGAAGAGAGCGATTGGGAGAAGGAACAGAGTAATAAGATGGAGCGTCTAAGACAAGACCGCAGTATCACGAAATCTCCAGAAATAAAAAAGGCAGCATCTTCACCTAACAAGTTTCTCAACCCGCCACCAGTCGTGGTGTCGCCACCCACACCCACGAACCCCGAGCCCAAGCCGCTCATATCGCCGACCTCCCCGGTGCTTGACCCACCTGTCACTGGGGGGCGCCGTCCCCCCTTGATTGCGGCCTACCCGCGGCCGTACAGGGGTATCACCATTTCCCCCAACCGTTCAATCTTGAGTTCAACCGTATCAAACACAAATAATTCCTCGCAGGGCTCCCAAGAGGGCAACGGAGAATACAATCCCGATATGACGTCGACCCCCAAATCGCAGAGGGCTGATGTGATGATTCCAAAAATTCCTGCGACTCCCGATTACCGCCTTTCGACGTCCGGTCTCGACTCTCCAAGCCAACCGCAGCACGACTCTCCGTTTATAAACCCAATTCTTCAATCCCGATTCCCCTCCGGTAAGGTCGTCCAGCAGCAGAACGGCGCCTACAAGGTCAAGGTCATGCAATATGAGTGACCTCCCAAAGTCAAAGGTCAAAAGGTCAACCTCACTCCTAACTTAATTAACCCAATTTAGGATTTTGTGATGTTATGTCCGAATGATCCTTAGGCTTCATCCAAATTCAGATTTGGGTCAAATTAGGTTATGTGGAATTAACTAGGTCAGGTCATAAGGTCAAAATATAGGTCAAATATAGGTCAAAAGTTTCACAACCAACAATATCATGTGCAGAATCTCTCTTATCGCAAGTTAGTTTTAAGGTTGTCTCCCAATATTTCTACTTGTCTCCCAATATTTCTACTTTATAAGTAAATCATTAAGTAAAACGTAGCAATTGAGATCTGACCTTGATTTATAGGTCAAGGTCACTGTCATTATCTAAGGCTATTGCATTATTGTCACCAAGAAAGGTCTTAATGGAAAATGCAATAGTTAAAATGGTAGCAGGACAATATGAAAGAACTTTAAAAGTTATATTGGAGCAGTACTTATAAAGTAAAACTGCATTTCGAATGGATGACCTTGTAGTTGACCTTACAAATAGGTCAAAGGTCAAATGAAGGTAATTTATACGACCTTTCTGTTTCTTAAGACCCAGTTTTTGTCAGGTTAGACTAGGATGATTATGATGATCTATAAATATAACCAATAAGAACTGCCATACTGCCAAATTGTAATTAATTTAAAAATCTAGATACACGTTCTTCATATTCTCATTTGATATGACTTAAAATATATGAATTTTTCTATGGAGGGATAATTTCATTCTAAGCATATCGTTAATTATTCTTGCAAGCGGAGAACGCTCACGGCAAGAGAAATATGATACAGTTGATACCTTATAAATTATTTTCTATTACTTTAATAAGTGAATAGATTTACATAACGGTTATGTTAGTTAAATTTTGAAATCTTGTTACGGCTATATCATTTTCACAACAATAAGGGACGAGAGACTACGATTGGTTGTCCGCTCTATTGAATAATACAGAATTAAATCTGATTTAATTCATTGAATTGAACTTAAGCGATTACAGGGGGGTCTGTATTGGATATATCTGTGTTTGATGAGGGAAACCACTGTACAGAATGTTGTTTTATGTGTATATTTCCTTGTAAATAATGATGATGTCATAATCATGATGATGTCACAATGGCTGATGTCACAAATTAAGCAGTTACATGCCTGTTATCACTTTAAATATAATTGTAAAATGTATGTACGATGTATATCCAATGGATTTTTATAATTTTTACATTGTTTTAATCGCCTACACACACATGTGAATCATTGTTAATTAGTGGTTTTAAACTTAGGGAGACCGTTATTGTGTTATAAATAAATGTCAGATTTATGTCAAATGCCTGTATACAGTCTTTCTTAACGTAAATTGTGATTCGTCGATTGTAATTCTATGACGATTAGGCATTAATAAGACGTTAATAACCTTTTAATAATTATTATTAAGGTATAAAGTACCTCCAAACCGCTGTCAGCGCCGATCTGACTTTTAAATGGTTGAACAGTTGCAGTTACGGCGTTACTTGGACGTTCAACCAAACGGCGTTAAACGTAGTTTCATTACCGTACTTATACGACAAAATTCCTTTTATCTGATAAATGTGTGTAACTGCTGACTTGTTCGATTATTATTAGGGCTCTCAAGTTGTGAAATAGTTAAAAGATTATGTTTATATCGTAGCGTATTGTTTTAATTTTGAAGAAATGTTTGTGAAATCTGACGCACTGACTGAATGAAGCTGCATCTATGATGTCAATTAGAGTTATTGAGTAAACATGAATTGTGAATTTTCGTGTTTTGTGTAAATTAATTTAATTAATTTTGTTCCTTCCCTTTAATGTGCGAAGAGTGTAAACTTAAAATGTTGAATATGTTTGATAAATAATACTCAGATTTGTTACTTAATTCAATTGAAGTGTTATTTAAATTAGATTTAGACTTCCGTGAAAGTTATTGAATCAATCATGTCCACTCGTGAAGTAGAACTGTTTTAGATGAAATTTACCTCAAGTAATTTGCACATACACCTAATTTGTTCAATCACTGTAGTATTTATTAATGACAATTAATTAAAATGGAATTTCGTTGGTTATTTATGAAATAAATAGTCTAAATTAAGAAATGGTAACTTATGTGATAAGTTATTACAAGAGAATAATTCAGTTCAGTTGAGGTTGTATAATGAAGTTAAATTACTTTGAAAAATTTCTGAATTAATTCATCATTCATACCTAACAATTAACTCGGCAATGAGTCAGCATACAGAATGATGCATAAATATGAAATATCTTTGATGGAATTATACAAATTAGTGTTTAATTATTATGTGATTAATTATCATTATCACATAGAATTGATGATTATTTCAATTGGTGCATTCATTAAGTATCACAAATTACATAATTTCATTATGTAATTGCATTATGTATGTCAGTTATTAATTGTGTAATTGAATTATGGTATAATTCATAATTAGCTCGTCTTAGTTTTAAAATAACATTGTAAATAATAGCATGGACGTCTGTACAGAATATTGTATGGTTGAAAGAACTGAACTTATTGTTAAACTTGTACAAGAAAGGCTTTTTTCTATTAATGCATGGATGAATTTGCATATATGTTAATTAGGCATGGATGAATTGGCATATATGCTAATTAGCGACGCATGGATTTGCATATATATGTTAATTGGTGTACGGGAGTGTATAACTGATATATCTTGCATGGTTAGTTGATTGAAATTAAAATTTTGTATCTTTGGGAAAATGTTTAATAACTGAGGGGAGGGGGGTATACTTATCTCTCAAGAGAGGGCAGATACTTTGTAAATAGCTACATGATGTGTCCTGAGAAATTATGATATTTCTATAATTAAAGGTTTCTCTTCGTAAATTCTTATTTGTTTAAGGGGTATTTTAAAATTATTTGAATCATTATTTCATAAGAATTCTCTAAAGGGAGATTTGAGCTTATAAAAAAGTCTCGTAAAATTATCCTCAATTTTGTAAAAATCAGCGGGCTTCAGTTCAACCTATAATTTTACAATTTTTCCCAGAATTTTCAGTAAATATTTTAAATTTACGCATGATTTGTTCAGTTGAGTTATTGTAAAAATGGCGTATCAGATTTAATGTGACTTAATGCTGACACTGCACTCTATTGCCAAAAAATACGGAGGTTAAAATTGCATCATGTTATGGAAGACTTCTATTAATAGATTGTTATACGTAGTTAGTATAATGTTATTATAAATCACCACGCAGAAGTCTTAATTGTGTCAAATCAGTTGTTGTAACTGATTTGACCTTGGTAATATCAGTAGGTCATGTCATGACCTAACTAGGTCATGACAAAATTAGGTCAGGTCATGAAACTAGGTCAGCTCAGTAAATGTTTTAAAAATGTTAAAATGAAGTCGCACACATTTTGTGAAATATGTCGCTGCTGTTGATCTTTTTTGTATTTGTAAAATATCGCCATGGTTACGGCTGTGGTTACCGAGTCTTACGACTATGTATAGCCATGGTTACGAGTGTTACTGTCATAAACATGTCATAAACGATCGTCATAAACTGTTGTCATGAGAAAGCCATTGATTGTCATGATCTAAGTGTAAGATACGTGTAATAAAAAAGTTCTGCAATTAAAAGTTTTTAACGGTACAAATGTAATTATTACGTGTTTGTAATAATTGCTGTTTGAAAGAGTTGATGGAAAGTGAGCCTTGTTAATTAATCTTGTATAGCAGTCGGACATATATGGTGAAGCGACTGTCTATAAGTCTCTTTTGCATCTGAGATTTTAAATGTTAATAACAATCTTAAGACGCAGATGACTAACAGACTTATAGGTAGTCGGCTTAATGTGTATGATATCATTTCTTGATATGACTCATCAATTGTTTTTGTCATCTTTTTTATCTCGTTAACTAGGTATTCAACTGAATCTGCTGTCTTATTAATTGTTATGAACTCCCTCTGTGAATATGTTTTTACTGCATGAGTCCATGGTGGCCGTTTTAACTTTACGACGACATTATCTGACATTGTGACGCTGAAACTGCAGTGTGACGATCGTGACCTCGTTTGTGGTTTAGATTTTAATTGTAAATTCTTTTCCGGTCCTTTGTTTTAATTGATTAACCAATTAATTAAGTCTTGCTGAAGTTATATTAATATTATATTAATATAAGGAAGATTCAAATGGTCTCAATATTGTAGATACGACACAAACTGTTTTAGGTTAAATGTTACAAATGTTACAAGAAAGTGTGAATAGTCTACCCTTACAAGCCCTGACCATGTGTAAGCTGTTTCATTATTGGAAGTGAAAGTGAAAGTGGGCCATACTGTGCTAATTATAGCTTGATTGAAGCAATCTGATTGGTTGGTACAAGAATGGTATGCAAATCATTGTTGTTAATTGTAAGCTTGAGATATGATCAACTACCAAAATAAAGTTTCTTATATATATACCACTCCAGTGTCGTGTATTTATTCCTG

Protein: 1523 aa

>Scribble_protein

MLKCFPLWRACNRQVEYIDKRHCNLTSVPEDVLRHTRTLEELLLDANQIRELPRGLFRLLQLRKLGLSDNEIGILPPDIANFMNLVELDISRNDIFDIPENIKFCKNLQVVDFSSNPITKLPDGFTQLRNLTHLSLNDVSLGRLPADIGSLSNLVSLELRENLLKYLPASMSFLVRLKSLDLGSNSLEELPETIGALPSLQELWLDCNDLVELPKEIGNLKKLTQLDVSDNKLEMLPEELAGLLALTDLHLSQNQIEYLPDGIGNLRKLSILKVDQNRLMELTPTIGNCECLQEVVLTENYLSELPASVGRLKNLSVLNVDRNRLADLPKEVGRCGKMGVLSLRDNRIMYLPAEVGNLKELHVLDVSGNRLQFLPITILNCDLKAVWLSENQAQPMMRFQTDTDDKTGNKVLTCFLLPQQAYQTESMENMLMGSIATDQDSRLSWNEVHSRSAVKFAGEEDGELPTDDEEESHFVRHNTPHPKELKARHAKLFNKSENAEGQAAPHSQKTTAKEDVTFMPSREKDTRWSTESQSSESSKTSGLSKASTAVKTPSVDHTMTPPSPPQEVQETAPVRSHVHITNSVTLIDNHAQETALDEEFDSSRSDADYGDKHIGFADDTEDHMSEGKLRRRDTPHHLKNKRINMTTAKDDAEEKVRAILSQASPGPKLQPSLDRPKRDSTDGGVVLTRQVAMEIREEEATVKIVREPGQGLGISIAGGIGSTPYRGDDEGIFISKVTEDGPAGMAGLMVGDKVISVNENNMVEVEHQRAVQVLKEAGNMVTMVVTREVLNQEKHKAAISASRDKLEQIGSESLDPELQIRTESISMILKRDDNGLGFSIAGGRGSTPYKGSDEIKQEEIAPTTQGRDSIYISRVTEGGAAENDGRIQVGDRIISINAVDVSDARHDQAVALLTSSGDEVALILHREILLDSNGDVEEHERVPSPTPSPQPQITSYAPAPLPPVSNPPGMETHITRSSPAHKVSPKPDIAPRSVLGEMPSKWGPPPPAPPRTQSVPLPSPKPAPVVAGKGSNSPKPAVSPRGSSLVEQFNMLAKKTPEGAPRVEGGIPRKSPGSSPARSSLSSDLNHQVSESHDDKFPTEIITIVKAGGPLGLSIVGGTDHTSHPFGIHEPGIFISKIVPDGAAAKTQLSIGDRILSVNGKDVTGASHQEAVMSLIAPTYEIVLEVRHDPPPPGLIEYKLTKTPGEKLGISIRGGARGPPGNPLDSTDEGIFVVKIQPNGAVARDGRLKVGHRILEVNGQSLLGATHQEGVRALRSVGDKISIMVCEGFDPNQVDSTLLAIARTESVSSVDRDDEDMLIIQKEQEMLKEESDWEKEQSNKMERLRQDRSITKSPEIKKAASSPNKFLNPPPVVVSPPTPTNPEPKPLISPTSPVLDPPVTGGRRPPLIAAYPRPYRGITISPNRSILSSTVSNTNNSSQGSQEGNGEYNPDMTSTPKSQRADVMIPKIPATPDYRLSTSGLDSPSQPQHDSPFINPILQSRFPSGKVVQQQNGAYKVKVMQYE

Domains

LRR: 80 – 402

PDZ: 701 - 785

PDZ: 828 – 924

PDZ: 1102 - 1187

PDZ: 1195 - 1286

Clone

Partial ORF

Primers

Forward: ATCGGAATTACCAGCGTCAG

Reverse: CACGGTTGCTTCCTCTTCTC

Nucleotide# 1119 – 2319 = 1201 nucleotides

>Scribble_clone

ATCGGAATTACCAGCGTCAGTTGGACGGTTAAAGAACTTATCGGTTTTAAACGTCGACAGAAATCGACTGGCAGATTTACCCAAAGAGGTCGGCCGGTGTGGAAAGATGGGCGTCCTCTCGTTAAGAGACAACCGCATTATGTATCTTCCTGCAGAAGTCGGCAATCTGAAAGAACTTCATGTTCTTGATGTTTCCGGAAACAGGCTCCAATTCCTCCCGATCACAATCCTCAACTGCGACCTAAAAGCCGTCTGGTTATCGGAGAACCAAGCGCAACCAATGATGCGATTCCAGACAGACACGGACGACAAAACGGGCAACAAAGTCTTGACGTGTTTCTTGTTGCCGCAACAGGCCTACCAGACGGAGAGTATGGAGAATATGTTGATGGGTAGCATTGCCACAGACCAAGACAGTCGCTTGAGTTGGAACGAGGTGCACAGTCGCTCGGCCGTGAAGTTCGCAGGAGAGGAGGATGGAGAGCTGCCCACGGATGACGAGGAAGAGAGTCACTTTGTCCGACACAACACCCCACATCCGAAGGAGTTGAAAGCTCGACACGCCAAACTTTTTAACAAGTCAGAAAACGCCGAAGGGCAAGCTGCACCACACAGCCAGAAAACTACAGCTAAAGAGGACGTCACGTTTATGCCGTCTAGGGAAAAGGACACCAGGTGGAGCACGGAGTCACAGAGCAGCGAAAGTTCCAAAACATCTGGTCTCAGTAAGGCCTCAACTGCAGTCAAAACGCCCTCTGTTGACCACACGATGACCCCACCCAGTCCGCCCCAAGAGGTGCAGGAGACGGCCCCCGTCAGGTCACATGTTCACATCACAAACTCGGTCACTTTGATCGACAACCACGCTCAAGAGACCGCCCTTGATGAGGAATTTGATTCGTCGAGATCTGACGCAGACTACGGAGACAAACACATAGGATTTGCAGATGATACGGAAGACCACATGTCGGAGGGAAAGTTGCGGAGGCGAGACACCCCTCATCACTTAAAGAACAAGAGGATCAACATGACGACGGCTAAGGACGATGCAGAGGAGAAGGTCAGGGCTATTCTGTCACAGGCCAGTCCTGGGCCAAAGCTGCAGCCCTCCCTGGATAGACCTAAGCGAGACTCAACAGATGGCGGCGTAGTGTTGACGCGACAGGTCGCCATGGAGATCAGAGAAGAGGAAGCAACCGTG

Cloning info

PCR rxn on 05/05/16 using GoTaq polymerase mix

| Template | Initial denature | Denature | Anneal | Extension | #cycles | Final extension |
| --- | --- | --- | --- | --- | --- | --- |
| 2 hr cDNA | 4min @ 94C | 30sec @ 94C | 30sec @ 55C | 2min 15 sec @ 72C | 35 | 10min @ 72C |

**NaK ATPase**

Gene model: 4682 nucleotides

ORF: 127 – 3223 = 3087 nucleotides

>Nak-ATPase_full

GCGAAGCAGAACGCATCAAAATTCCACCAATCAGCAACGAGCAGTCATTCTGGGATCTGCTTTTGCAGGGCTACATAGTTAGCCTAAAGAAACTCCCAAAAGAATCAGAAATAGAAAACTACAATCATGGGGAAGGACGATCGGAGTGAGAGCTATCGCCATGCGACCTACAAGAGCGTGGCTCAAGATGAGAAGGGGAAGAAGAAGAAGAAGAAGGGAAAGCAAGATTTGGACGAGCTCAAGCAGGAGTTGGAGATGGATACCCATAGGATTAGCCTCGATGAGCTCTGCCAAAGATTTGGTACTAACCTCGATACTGGCTTGACCCCAGAGAAGGCCAAGGAGTACTTTGAGCGTGATGGACCCAATGAGCTGACTCCCCCAAAGACCACCCCGGAATGGATCAAGTTCTGCACCCAGCTGTTCGGAGGTTTCTCTCTTCTGCTGTGGATTGGCGCCATCTTGTGTTTTGTGGCCTACTCCATTCAGGCCTCCACCTATGAGGATCCCCCAGGTGATAACTTGTACCTTGGCGTTGTGTTGACGGCTGTGGTGGTCGTCACTGGCTGCTTCTCCTACTACCAGGAGGCCAAGAGCAGCAGGATCATGGAATCTTTCAAGAATCTCATCCCCCAGTTTGCCCTTGTCATCCGTAACAATGAGAAGTTGAACGTGCACGCTGAGGAGTTGGTTGTGGGAGACGTCATTGAAGTGAAGTTTGGTGACCGTATCCCGGCTGATATCCGTGTCGTCTATGCCAACAGCTTCAAGGTTGACAATTCATCTCTAACTGGAGAATCTGAGCCCCAAACCAGAACAGCCGAATTCACCAACGACAACCCATTGGAGACCAGGAACTTGGCCTTCTTCTCTACCAATGCCGTCGAAGGAACCGCCAAGGGAGTGGTTGTCAGCACTGGAGACCGAACCGTGATGGGTCGTATTGCTGGTCTGGCTTCAGGTCTGGAGACTGGAAAGACACCCATTGCTATTGAGATTGAGCATTTCATCCACCTCATTACTGGTGTGGCGGTGTTCCTGGGAGTGACCTTCTTCGTCATTGCCTTCATCCTCGGCTACTACTGGTTGGACGCCGTCATCTTCTTGATTGGTATCATCGTGGCCAACGTGCCTGAAGGTCTGCTTGCCACTGTCACTGTGTGTCTGACTCTGACAGCCAAGCGCATGGCGTCCAAGAACTGCCTGGTCAAGAATTTGGAGGCCGTGGAAACCCTCGGATCAACGTCCACCATCTGCTCGGACAAGACAGGAACCCTCACCCAGAACAGAATGACCGTCGCTCACATGTGGTTCGACAACCACATCATGGAGGCCGACACAACTGAGGATCAGAGCAGACAAGATGCGGCCTACGACAACACCACCCCGACCTGGAGAAGTCTTGCGCGTATTGCCATGCTGTGCAACAGAGCGGAGTTCAAGGTTGGCCAAGAGAACGTCCCCGTCCTGAAGAGAGAATGTAACGGAGACGCCTCCGAGTCTGCCCTCTTGAAGTGCGTGGAGCTGTCCATTGGAAACGTCACCCAGTACCGAGCCAACAACAAGAAGGCCATAGAGATCCCGTTCAACTCCACCAACAAGTACCAGGTGTCCGTGCACGAGACTGAAGAAGCCGACGACCCTCGCTACCTGCTAGTGATGAAGGGCGCCCCCGAGAGGATCCTGGACAGGTGCTCCACCATCCTGATCAACGGCGAAGAGAAGGCCCTGAACGACGAGTGGAGGGAGGCCTTCAACCAGGCTTACATGGAGCTCGGAGGTCTTGGAGAGCGTGTACTCGGATTCTGCGACTACTTCCTCCCCGCTGACCAGTTCCCCGTTGGCTACCCCTTCGACGCTGATGCCGAGAACTTCCCCCTGACCGGACTGCGCTTCGTCGGCCTCATGTCCATGATTGACCCTCCCCGTGCTGCTGTGCCTGATGCTGTGGGCAAGTGCCGATCTGCTGGAATCAAGGTCATCATGGTCACAGGCGATCACCCCATCACTGCCAAGGCCATCGCCAAGGGAGTCGGAATCATCTCTGAAGGAAACGAGACCGTAGAGGACATTGCCGCTCGCCGAGGAGTCCCCGTGGCTGAAGTGAACCCCCGTGAAGCCAAGGCGTGCGTTGTGCACGGCTCCGACCTCCGTGACATGACCCCCGCCCAGATTGACGACATTCTGTCTAACCATTCCGAAATTGTATTTGCCAGGACATCGCCCCAACAGAAGTTGATTATTGTAGAAGGTTGTCAGCGTCAAGGAGCTATTGTGGCTGTCACTGGAGATGGTGTTAATGACTCTCCCGCTCTGAAGAAAGCCGATATTGGTGTGGCTATGGGTATCGCTGGAAGTGATGTGTCCAAACAAGCTGCTGACATGATCCTGCTGGACGACAACTTTGCCTCCATCGTGACAGGCGTAGAGGAAGGTCGTCTGATCTTTGACAACTTGAAGAAGTCCATTGCTTACACACTGACCTCCAACATCCCTGAGATTTCTCCCTTCTTGCTCTTCATCTTGGCTGACGTCCCTCTGCCCCTCGGAACCATCACCATTCTCTGCATTGATCTTGGAACTGACATGGTACCAGCCATCTCCCTTGCTTACGAACAAGCTGAGAGTGACATCATGAAGCGTCAACCGCGTGACCCTCTGCACGACAAGTTGGTGAACCATCGTCTTATCGGTATGGCTTACGGTCAGATTGGAATGATCCAGGCTTCAGGTGGCTTCTTCGTCTACTTTGTCATCATGGCTGAGAACGGATTCTGGCCGAGGCGTCTACTCGGCATCCGTGAAGAGTGGGATTCCAATGCAATTAACGATCTGCAGGATTCCTACGGCCAAGAGTGGACGTTCGCCCAGCGCAAGGTCCTGGAGTACACTTGCCACACGGCCTTCTTCGTGTCCATCGTGGTCGTCCAGTGGGCTGATCTTATGATCTGCAAGACGAGGAGGAACTCCATCATCCATCAGGGCATGTGGAACCACCACTTGACCTTCGGCCTGTTCTTCGAGACATGCCTGGCAGCTTTCATGGCCTACACTCCGGGTTTAGATACTGGCCTGCGTATGTACCCCCTTCGGTTTACTTGGTGGCTTCCCGCACTGCCGTTCAGTTTGGCTATTTTCGTGTACGACGAAGTGAGAAAATACCTACTGAGGAGAAACCCAGGCGGTTGGCTGGAAATGGAGACGTACTATTAAGTTCGCTCCGTTCCTGTCTTCAAGACCTCCAGTGCTTCGTGTCCCGTCAAAAGTTACACTCCCTGGTTCAGGGCGCCCGACCCAAGATGCAGCGGCTGCGGCGCAGCGCGTCTCATCATCTGATGTTCTTCTGCGTCGTGTTGCTAACGCGATTCACCATCACGCGCAACCACCGGTCGGGATCGGCCATCTTAGCAAACTTTTTCTCTCACAACGTTTTTGTGTGTTACTCCAGTAACAAATGGATCTACAGAACAATCCCCGTAAGATAGAGCCCCTCGCGACATCTTAGGCTCTTCAATTCATCAAATTACATCAAAATCAATTGACATACACATTGTCTATGTACTTCAGGATTGTGATGTATTACATCCGACATCAAACATCATCCTCATCGCAATCGTGATGTACGTTATCGTTACATCGTTACATCTTTATTATTTGTTATACATTGTTAAATTTGTTACAAACCATCATGAATTCTGGCTGCCTGTGGCATTATGTTTTACTATTTATGTACGTGCTACTACGTATGTATATTTCAGTGTTTGTCCAACATCATTTATATATTAATCTATTGGGTAGTCATATCAATTTCATACGACCATATCATTCATGTATTGAATATTATAAAGCTTCGTAAGTCAAACTGTGTTTCGCTAGACGCCAGTCATAGTTGTACATGTGTTCAGTGTACGAATATGATACAAATACGATACGAAAATGATACTAAGTATGATACGCATTATTGCTATGTATCCGTAGTACAGGATCTTGTTGTTATGAATTTGATGCATAATGTTTTTACATTCCATACATAGTCTTACGGGGATCATGAATTACATTTACAATACATTGTCATATTATGATATTACATGATATTCAACAGTAGGTTGTGAACTGTGAATTCATAGCGGGCAATTAAACGCTTTATATCATTTTGCTACATCTGTCAAAAAATCTGTAGACCCATATTGCTGTTGTTACAGACGAGGTTGTGAGATGCAAAGTTAAACCTGCAGTCATTTCTGTGGCATTTCACATTTATTCTGCGGCTTATTTGTCAATGGTAGCAATGCCAATTGGACACATAAGTCGCAAAATATTTGTGATATGCGTGAGAATTATGAAGGCGCTTGAACCTTGGGATAAATTTTTTTTATTCAGGCCATTGTTATTGTTGACACAAGCTTGGCTGGTCTTGGGAGGCCACCAGTTATCGAATGATTAATTTTTCCCTCATTGTTTTTAGTCTAATTAGCGAACCAATTAACTTAGACAGCAATTGTCTATATATAATTCTGAATATTTGTCTTGGTTGCATTTGGCGTATTTCGGACCATTTGGAATTCCTATTGCCTGATTATTCTTTGTTCTATTCCTTCGCGCTTATGGCTTTTATGGAAATATTATGAAAAGAAGGAAATTTTTTAGTATAATAAAAGTTTTACAGCTGAAAAAAAAAAAAA

Protein: 1028aa

>NaKATPase_protein

MGKDDRSESYRHATYKSVAQDEKGKKKKKKGKQDLDELKQELEMDTHRISLDELCQRFGTNLDTGLTPEKAKEYFERDGPNELTPPKTTPEWIKFCTQLFGGFSLLLWIGAILCFVAYSIQASTYEDPPGDNLYLGVVLTAVVVVTGCFSYYQEAKSSRIMESFKNLIPQFALVIRNNEKLNVHAEELVVGDVIEVKFGDRIPADIRVVYANSFKVDNSSLTGESEPQTRTAEFTNDNPLETRNLAFFSTNAVEGTAKGVVVSTGDRTVMGRIAGLASGLETGKTPIAIEIEHFIHLITGVAVFLGVTFFVIAFILGYYWLDAVIFLIGIIVANVPEGLLATVTVCLTLTAKRMASKNCLVKNLEAVETLGSTSTICSDKTGTLTQNRMTVAHMWFDNHIMEADTTEDQSRQDAAYDNTTPTWRSLARIAMLCNRAEFKVGQENVPVLKRECNGDASESALLKCVELSIGNVTQYRANNKKAIEIPFNSTNKYQVSVHETEEADDPRYLLVMKGAPERILDRCSTILINGEEKALNDEWREAFNQAYMELGGLGERVLGFCDYFLPADQFPVGYPFDADAENFPLTGLRFVGLMSMIDPPRAAVPDAVGKCRSAGIKVIMVTGDHPITAKAIAKGVGIISEGNETVEDIAARRGVPVAEVNPREAKACVVHGSDLRDMTPAQIDDILSNHSEIVFARTSPQQKLIIVEGCQRQGAIVAVTGDGVNDSPALKKADIGVAMGIAGSDVSKQAADMILLDDNFASIVTGVEEGRLIFDNLKKSIAYTLTSNIPEISPFLLFILADVPLPLGTITILCIDLGTDMVPAISLAYEQAESDIMKRQPRDPLHDKLVNHRLIGMAYGQIGMIQASGGFFVYFVIMAENGFWPRRLLGIREEWDSNAINDLQDSYGQEWTFAQRKVLEYTCHTAFFVSIVVVQWADLMICKTRRNSIIHQGMWNHHLTFGLFFETCLAAFMAYTPGLDTGLRMYPLRFTWWLPALPFSLAIFVYDEVRKYLLRRNPGGWLEMETYY

Domains

P-type_ATPase_Na-K_like: 65 - 1025

Clone

Partial ORF

Primers

Forward: CGTGCACGAGACTGAAGAAG

Reverse: AATCCCACTCTTCACGGATG

Nucleotide# 1614 – 2815 = 1202 nucleotides

>NaKATPase_clone

CGTGCACGAGACTGAAGAAGCCGACGACCCTCGCTACCTGCTAGTGATGAAGGGCGCCCCCGAGAGGATCCTGGACAGGTGCTCCACCATCCTGATCAACGGCGAAGAGAAGGCCCTGAACGACGAGTGGAGGGAGGCCTTCAACCAGGCTTACATGGAGCTCGGAGGTCTTGGAGAGCGTGTACTCGGATTCTGCGACTACTTCCTCCCCGCTGACCAGTTCCCCGTTGGCTACCCCTTCGACGCTGATGCCGAGAACTTCCCCCTGACCGGACTGCGCTTCGTCGGCCTCATGTCCATGATTGACCCTCCCCGTGCTGCTGTGCCTGATGCTGTGGGCAAGTGCCGATCTGCTGGAATCAAGGTCATCATGGTCACAGGCGATCACCCCATCACTGCCAAGGCCATCGCCAAGGGAGTCGGAATCATCTCTGAAGGAAACGAGACCGTAGAGGACATTGCCGCTCGCCGAGGAGTCCCCGTGGCTGAAGTGAACCCCCGTGAAGCCAAGGCGTGCGTTGTGCACGGCTCCGACCTCCGTGACATGACCCCCGCCCAGATTGACGACATTCTGTCTAACCATTCCGAAATTGTATTTGCCAGGACATCGCCCCAACAGAAGTTGATTATTGTAGAAGGTTGTCAGCGTCAAGGAGCTATTGTGGCTGTCACTGGAGATGGTGTTAATGACTCTCCCGCTCTGAAGAAAGCCGATATTGGTGTGGCTATGGGTATCGCTGGAAGTGATGTGTCCAAACAAGCTGCTGACATGATCCTGCTGGACGACAACTTTGCCTCCATCGTGACAGGCGTAGAGGAAGGTCGTCTGATCTTTGACAACTTGAAGAAGTCCATTGCTTACACACTGACCTCCAACATCCCTGAGATTTCTCCCTTCTTGCTCTTCATCTTGGCTGACGTCCCTCTGCCCCTCGGAACCATCACCATTCTCTGCATTGATCTTGGAACTGACATGGTACCAGCCATCTCCCTTGCTTACGAACAAGCTGAGAGTGACATCATGAAGCGTCAACCGCGTGACCCTCTGCACGACAAGTTGGTGAACCATCGTCTTATCGGTATGGCTTACGGTCAGATTGGAATGATCCAGGCTTCAGGTGGCTTCTTCGTCTACTTTGTCATCATGGCTGAGAACGGATTCTGGCCGAGGCGTCTACTCGGCATCCGTGAAGAGTGGGATT

Cloning info

PCR rxn on 05/05/16 using GoTaq polymerase mix

| Template | Initial denature | Denature | Anneal | Extension | #cycles | Final extension |
| --- | --- | --- | --- | --- | --- | --- |
| 2 hr cDNA | 4min @ 94C | 30sec @ 94C | 30sec @ 55C | 2min 15 sec @ 72C | 35 | 10min @ 72C |

**Neurexin IV**

Gene model: 5225 nucleotides

ORF: 128 – 4150 = 4023 nucleotides

>Neurexin IV_full

CACACCTGAACTTACCTGTTGGCGGGAAAACACCTGTTCTTGTTCCTGTAATTACCACGAGGAGCCGCATCAGTCGGCGACCCATTTTCAGAACGTCTTGGCCAATGAGAAACCACAACAAAGTCACATGGTCCACCAAACGTGCTGTAGACCCAAGCAGACGAACAGGGCTGGAAAGATGGGTCGAAATAAATTCCGAATATTATGGATACTAAGTATATCTGTGATCTACAGTCATGCACAAATTGACATGACAAACCCCTGCCGCTTCCCTGTGGCTGTGGGAATTGAGAAAGAATACATTCCGGCCAGTAGTATATGGGGCACGTCACAGACTAATATCGCAAGAGGACCCCAGTATGCCAGGCTACATGGATACAAAGGCGGTGGTGCCTGGACGGCAGGGATCCCTAACAACAACCAACGTTTGGGGGTCGACCTTGGTTACCGCCATGTGGTAACAGGTGTCGCCACTCAAGGTAGAAGAGGGAGTTACGAGTTTGTGACGGAATATTATCTAGAGTTCTCGTCAGACAACCGAACGTGGAGTGTCTATACTAACGAATATGGCACGCCTTTTATGTTTGAAGGAAATACAGATGACGATGGTGTCGCAAGAAACAACCTTGACTACCCCATCGTTGCTAGGTACATTCGCTTCAACCCTCAGAGGTGGCACATGTTCATTTCCATGAGGGTGGAGGTCTACGGCTGTCGCTTTGATGGTGAATCAGCAACTTTTGATGGAACCAGCAGAATTTCTTACGACGTCAGTGGAAGTAACGAATACATGCAAACGAGATCGGACCAATTGAAATTGAGGTTCAGAACGAGTAGCGCTAATGGATTATTATTCTTTGCGGATTCTAATCAGGGAGACTACGCCATCCTGGAAATGCTCAGAGGCAGATTATACTTTCATATTGATCTTGGTACGACGGCCATGGCATCTGGTGACACGACCTTGAAAGCCGGAAGTTTATTAGACGATAACCAGTGGCATGACGTAGAAATTAACAGAAATGGCCGAGAGGTCCATTTCACAGTCGATAGATTAACTGTGACCAATATTACCAATGGAGACTTTTACCAGTTAGATATCGACAGACAGATTCATTTAGGAGGTATCAACAGCTATTTACAACCAGGAAAGAGATTGTACACTCGAGAAGGATTCACAGGGTGTATGGAGAATGTGTGGTTCAATCACATGAATATCATAAGAGACGCTAGAATGCAGCAGCCGAGGTTTACTAGTTTCAACATAATGAATGGCGAATGTCAGCTACGACAAGTCATCCCTTTCACTTTTCCGACAACAGACGCTCATCTGGAGGTCGCTACAGCGTCAAGCAACAGGCTACGTGTCTCCTTCGATTTCCGATCGTACAACAGAGACGCCATGTTGTTCTATTCATCGCTCTCCCCTGACGGATATGTCTCGATCAAAGTTGACGCAAATGGCTACTTGGAGTACAGCGTCAAGGCTAGCAATCAGCCCGAAGTGACCAGTATATTAACTAATCTGGATCCTTTGTCAGAAGTGGACGTTTTCACGGACGGCCTCTGGCATTCCCTGCATGTGGACATAGATTCCGGAGGGAACGACCGAGTGGGAAAAATCAATATCACCATTGACGGGAGGGTGGATCATTCCAACAGGCAGTTGACATTCACGACAGGGAATACTTATTTTATCGGAGGCGGACAAGAGGATACAGGCCAGATAGGATTCCTTGGTTGTATGCGTCTTCTGGAAATTAATGGTGAACCTATGGACATCATCCCTGCTGAATTTAACGAGGGAGTTATCAACGGAACATGTTCTGTTCAAGACAGATGTGACCCCAACCCCTGTGAACATGGCGGCGTATGTTCTCAAGATTACATGACCTTCTCTTGTGACTGTACTGGAACTGGTTATGAAGGAGCAGTCTGTCACAGATCCGAATACCTGGTGTCTTGTGAAGAGGCTCGTCTTTTGAATCCTCTGATTCCTACTAAAGAACTGATGATCGACATTGACGACTCTGGGCCCCTGGATCCCATCCCTGTGACTTGTGAATTCCAACACGACATTGGCCTCTCTATTACTCGAGTCCACCACGCCAACGAAGAACCAACCCTGGTCAAAGGATACCAAGAGCCAGGGTCTTATGTCCGACCAATTGCTTATCCTGCTTACAGAGAACAGTTTGATGAACTCATTATGCGAGCGATGACCTGCGAACAGAGGATTAAATGGGAGTGCATGAATGCAAGGCTTCTGTCTGACGCAGGAGGCTCCGATCCTAATAAGCCATCATGGGGATGGTGGGTCGGCCGTACCAACTTGAATATGCGCTACTGGGGCGGCAGTTCCCCGGGGTCAGGCAAGTGCGCCTGTGCATTGAAGAACGAATGCCGAGAGAACAACCCCTTCTGCAACTGTGACGCTGGCCTGGTTGCCGACGATATCTCCGATGACGGGTTCATTACTCAGAAGGAGCATCTTCCAGTAATGGAGTTGAGGTTCGGAGACACGGGAACATTCGCTGAGACTAACAAGTGGGGTAAACACACTCTCGGACCCCTTCGCTGCACTGGAGATAACCTCTTTGACAACGTAGTGACCTTCCGCAAGACTGACGCCACCATTGAGTTTGAATCTTTTGATGCCGCAACCTCAGGAGACATCAGACTGCAGTTCAAGACCACAGCTGAGAATGGCATCATCTTGCAGAACACTGGAAAATACAACTTCATTGAGCTCAAACTTGTTTTTGGCAACACTCTGCATTTCCGTTTTGATGTTGGCAACGGAATCCAGACTTTGGAGAAAGTGACTTCATATCCTTTGAATGACAACATGTGGCACACAGTCCACATAGAAAGAAACAGGCGGCAAGCCATTCTGCAAATCGATCTGCAAGCTGACGTTGTACTTAACGAGCCGGTTGATCAAGGATTTAGGACTCTGGAATTGAGCAGTCCTTTGGTTATTGGAGCGGCTGTCGATTATAAGGATGGTTTCGTGGGCTGCATCAGGTCCCTGATGGTGAACGGCAAGATTCTGGACTTGCGAGGCAAGGTGGAACGAGGAGAGGTGACGTACGGCGTCTCCGCAGGGTGCCACGCTCAGTGCGACTCCAACCCCTGTCTCAATAACGGCATTTGCATCGAGTGGTATTCCCACTATCAGTGTGATTGTGCTTATACGCCATTCCGAGGGTGGATTTGCGGAAGAGAGGTGGGAGTCAACCTCCAACCGGAAGAGATGATTCGCTATGAGTTTGACGAAGGAAGCGGAAACATTGCCACGGACGAAGAAACCATTATCATTGGCTTCTCCACCCAGTTAAAGAGGGGTATCTTGCTCCAGATTACGAACGGAAATTATCAATCCCAGGAGTACATTTCAGTGGAGATGAACAATAACGGTGGAGTAAAAACTCGTATTGACGTCGGCTGGGAGATCGGACCACAAGAAGTGAATAATGACGTCGATAACGTGGATCTTGCTAATGGGCAACAGCATGTCGTCACTGTGAAGAGGTTCAACAGAGGAAGATCCATATCTTTGGCTATTGATGACTATCCTGTGGAGATCTTGGATTTCCCTAATCTGGACCCAAGTACCGACACCAAGCTAGACAATCCAAAATACATTTACTTCGGAAGGAACGAGACTACCCCAGCCGGTCAAGGGTTCAAAGGCTGTCTTTACAGGGCGCAGTTTGATAATCTCTTCCCCCTAAGGAGGGTGTTCCAGGATCCTCGTCCCAGCTACATTACATTCCAACCTTCAACATTTAACGAATCCATCAGGGAGGACATGTGTGGATTTGAAGAAGTGACCCATGCACCAGAACCTGCCGAGTACCGTCCGACACCCACCCCTGACCCCAATGCCACCGTCCCAACTTATATTGGACGGGAGGAGGGTCTGGATCAGGGCGAACAAGCTCTATTAGGAGGTATTTTAGCTGTGGTGCTTCTACTACTTATTGGTCTCGCATTATTAGTCGGAAGATTCTTTACCCGCCACAAGGGAGAATACAGAACGTACGAAGCGAAAGACGCCCAATATTACGATAACGCTGACTTCGCTTTGGCGGAGGGTAACACCAATCAACCGGGCGTTCAGCAGAAGAAAGAATGGTTCATTTAATGTCAAGATGTATTTAGTTTTACAAATTGTGTGAATGGATGATGTAAAATTATGAGAATGAATGCAAAATTTAAGTATGAGTGAATGAAATTTTTGAAGAATATGTGAGTTGATTCTTATTTGTATTTTTATTGAGAATGAATCAAGAATTATTTGTTGTGAGAGGCGAGAAAAATTTTAAGGCTATTCTGAATTTATGAAGAATGACAATTTTATATAATATCTCTGAAGACTGATGTCAATGAGAATATAAAGCTGTGTCTGTAAATTTGAAAGTGAAATATACTTTGTCCGTCATTTATTTTTAATGATGAAAGTGTCAGATTATGAATTTGCAATTTTTATGAGGTCACCATGCTGGTGTCTGCTGGCTATGCAAGATTGATACATGTGTGAGTGATTTTGATTTTAACAAGTGATCTTATAATTTTTTTTCCCACTCGAAACATTGCCAAAAAATTGTTACAAAATTGGTTGATTTATTTGTTCTATTACAAGTCATTGTATGTTACCATGATGTTATCTATAGAAGAACTTGAATATCTGAACTTATGTTATTTTCAAATGAATATTTGCCGATGTTGAAGTAACGAAGTACCATAAAACTTAATTGACAGTAATTATTTTCAATTCTTCTTCAGCATTTACAAATTACACATTCCTTTTACTTTAATACGCAAATAGAAAATTGTAATTCATGAAATTAGTCTGGCGATAGGCCCTATACATGAACTGTTCATACAACTCTATGAACCATTTCTCGTTATTGGTATTTGTACATATCAATGTTTGTAAAGTTTTTAGATTGCTCTGCATTTTAATCTGTAAATAGCTCTTATTAATGATATCAATTTACATGCATTCCAATTGAACTAGGACTTGAATTTATTTATACAAGTGAATGGATCGAAATAAGTTGGTAGCCATGTTTTAATTATAAAATTATGATCGCAAAAGGAAAATTTCCATCTTCGCATCAATTACATGGATCATATACAAAATATTTTTTATTACTTATGATTCAACTGCTCTGTATATATGAGAATAAATTTTGTAAAGAAATTATTATAAAAAA

Protein: 1340 aa

>Neurexin IV_protein

MVHQTCCRPKQTNRAGKMGRNKFRILWILSISVIYSHAQIDMTNPCRFPVAVGIEKEYIPASSIWGTSQTNIARGPQYARLHGYKGGGAWTAGIPNNNQRLGVDLGYRHVVTGVATQGRRGSYEFVTEYYLEFSSDNRTWSVYTNEYGTPFMFEGNTDDDGVARNNLDYPIVARYIRFNPQRWHMFISMRVEVYGCRFDGESATFDGTSRISYDVSGSNEYMQTRSDQLKLRFRTSSANGLLFFADSNQGDYAILEMLRGRLYFHIDLGTTAMASGDTTLKAGSLLDDNQWHDVEINRNGREVHFTVDRLTVTNITNGDFYQLDIDRQIHLGGINSYLQPGKRLYTREGFTGCMENVWFNHMNIIRDARMQQPRFTSFNIMNGECQLRQVIPFTFPTTDAHLEVATASSNRLRVSFDFRSYNRDAMLFYSSLSPDGYVSIKVDANGYLEYSVKASNQPEVTSILTNLDPLSEVDVFTDGLWHSLHVDIDSGGNDRVGKINITIDGRVDHSNRQLTFTTGNTYFIGGGQEDTGQIGFLGCMRLLEINGEPMDIIPAEFNEGVINGTCSVQDRCDPNPCEHGGVCSQDYMTFSCDCTGTGYEGAVCHRSEYLVSCEEARLLNPLIPTKELMIDIDDSGPLDPIPVTCEFQHDIGLSITRVHHANEEPTLVKGYQEPGSYVRPIAYPAYREQFDELIMRAMTCEQRIKWECMNARLLSDAGGSDPNKPSWGWWVGRTNLNMRYWGGSSPGSGKCACALKNECRENNPFCNCDAGLVADDISDDGFITQKEHLPVMELRFGDTGTFAETNKWGKHTLGPLRCTGDNLFDNVVTFRKTDATIEFESFDAATSGDIRLQFKTTAENGIILQNTGKYNFIELKLVFGNTLHFRFDVGNGIQTLEKVTSYPLNDNMWHTVHIERNRRQAILQIDLQADVVLNEPVDQGFRTLELSSPLVIGAAVDYKDGFVGCIRSLMVNGKILDLRGKVERGEVTYGVSAGCHAQCDSNPCLNNGICIEWYSHYQCDCAYTPFRGWICGREVGVNLQPEEMIRYEFDEGSGNIATDEETIIIGFSTQLKRGILLQITNGNYQSQEYISVEMNNNGGVKTRIDVGWEIGPQEVNNDVDNVDLANGQQHVVTVKRFNRGRSISLAIDDYPVEILDFPNLDPSTDTKLDNPKYIYFGRNETTPAGQGFKGCLYRAQFDNLFPLRRVFQDPRPSYITFQPSTFNESIREDMCGFEEVTHAPEPAEYRPTPTPDPNATVPTYIGREEGLDQGEQALLGGILAVVLLLLIGLALLVGRFFTRHKGEYRTYEAKDAQYYDNADFALAEGNTNQPGVQQKKEWFI

Domains

FA58C: 59 - 195

LamG: 202 – 360

LamG2: 418 - 548

EGF: 572 - 601

LamG: 849 - 974

EGF: 999 - 1028

LamG: 1047 - 1198

Clone

Partial ORF

Primers

Forward: GCTACAGCGTCAAGCAACAG

Reverse: TATCCTTTGACCAGGGTTGG

Nucleotide# 1340 – 2139 = 800 nucleotides

>Neurexin IV_clone

GCTACAGCGTCAAGCAACAGGCTACGTGTCTCCTTCGATTTCCGATCGTACAACAGAGACGCCATGTTGTTCTATTCATCGCTCTCCCCTGACGGATATGTCTCGATCAAAGTTGACGCAAATGGCTACTTGGAGTACAGCGTCAAGGCTAGCAATCAGCCCGAAGTGACCAGTATATTAACTAATCTGGATCCTTTGTCAGAAGTGGACGTTTTCACGGACGGCCTCTGGCATTCCCTGCATGTGGACATAGATTCCGGAGGGAACGACCGAGTGGGAAAAATCAATATCACCATTGACGGGAGGGTGGATCATTCCAACAGGCAGTTGACATTCACGACAGGGAATACTTATTTTATCGGAGGCGGACAAGAGGATACAGGCCAGATAGGATTCCTTGGTTGTATGCGTCTTCTGGAAATTAATGGTGAACCTATGGACATCATCCCTGCTGAATTTAACGAGGGAGTTATCAACGGAACATGTTCTGTTCAAGACAGATGTGACCCCAACCCCTGTGAACATGGCGGCGTATGTTCTCAAGATTACATGACCTTCTCTTGTGACTGTACTGGAACTGGTTATGAAGGAGCAGTCTGTCACAGATCCGAATACCTGGTGTCTTGTGAAGAGGCTCGTCTTTTGAATCCTCTGATTCCTACTAAAGAACTGATGATCGACATTGACGACTCTGGGCCCCTGGATCCCATCCCTGTGACTTGTGAATTCCAACACGACATTGGCCTCTCTATTACTCGAGTCCACCACGCCAACGAAGAACCAACCCTGGTCAAAGGATA

Cloning info

PCR rxn on 05/05/16 using GoTaq polymerase mix

| Template | Initial denature | Denature | Anneal | Extension | #cycles | Final extension |
| --- | --- | --- | --- | --- | --- | --- |
| 2 hr cDNA | 4min @ 94C | 30sec @ 94C | 30sec @ 55C | 2min 15 sec @ 72C | 35 | 10min @ 72C |

**Neuroglian**

Gene model: 5116 nucleotides

ORF: 90 – 4421 = 4332 nucleotides

>Neuroglian_full

CCTTGCTGCAATGTGCAGACACACAGGACCCACCTCCGGGAGTACGAGAGGTGACCATACCCCCTTTAATAACAGTAGAACCGCCGAAAATGTTAGCCTTTCGAATTGGGGAGAGTATAGAGATGCCATGTGTTGCGACTGGCCAGCCAGATCCTAGATATCTCTGGCAAATGAATGGAAAGGACTTTGATCCGTCAGGTAACGATGGGCGTGTTGCCATTCAGCCAGGTGTGGGAACGCTCATCTTCAGCAACCCCATTGAGAGGGATGAGGGCATCTACCAATGCTTAGCCGACAATGGCCATGGTGTGGCAGTCACCATCAAAGTTGATCTCAGGGTTTCTTATTTGGAGAAATTCCCTTCCATGACACCCAGGCAATTTTACCCAAATTTGGGCGAAAAACTAACACTCTCTTGCACACGACCAGCCAGTTTTCCATCTCCCGAAGTGTTTTGGGCGATTGTTTTGGACGATCAGAGTTACAGTCCAGTCAGTTTGTCTGACAGAATCAGCATTGATCCCGAAGGAAATATGCACTTTGCCAATGTGCTTTCATCTGATTCCTACAGCAACATGCGGTATGCTTGCTTTGTCCAAAATCAAGTCATGAGACAGTTTGTGGAAGGAAGCTACGCATTCATCAATCCTATTGGAAATCAAATCCAGAGGTTTCCTCCTACATCAGTTTACAGGGATCCCACTTATCAAATTGCTCTTGTTGGCGAAACATGGAGGGTCAAATGCATTTTCTCCGGATACCCTACACCAAGGGTGAGCTGGGACAGGGAGGGTATGCCCATGCCCCCCAGGAAGAGGGAGGAGAGTTTCGGCCAAGAACTTGTCATCGAAAATGTGCAGCTCAGTGACGCTGGCAAATATGAGTGCTTAGGAATCAATGATGACGCCACAGTCCCCGACAGATATTCTTTTGAACTCGCTGTGGAGTCTGCCCCCTTCTGGGACCAGAAGCCAGAGTCCTTTGACGCTGCTGACAATGACACGGCCGTGTTTCACTGTTCTGCCATCGGAGATCCTGAACCTCTCATAGATTGGTTCATCAATGGAATACCAATTTCAAAAGCTGAGCCGAACCCCCGTAGAGTTGTGGAGAGGAACGTCATCACCTACTATAATGTGATAAAGGACGATGCTGCCGTGATCCAGTGCAACGCTACTAACAAACATGGCTACATCTTCACTAACGCCTACTTGAATGTTCTCTCCGAGCGACCAGTGATCACTGAGCCCCCAATGGTGGGCATGAGAGCTGCGGAAGGACAAACCGTCAACTTGACGTGCCATGTGTTCGGATCTCCGAAACCAGTTGTCGTGTGGAGGAAAAGTGGCGAGCAGTTGACTGGTGGGCGTTTCAGTGTCACAGAAGACGGGCATTTGATGATAGTGGACTTGTCTCTCGTTGATGCTGGATCTTACACATGCAATGCCACGAATAAGTTAGGCTCAGTCGCGGCCACGGGGTCGTTGATTGTGCGGCGAAGGACTACAATCCAAATGGCGCCTATGGACGTCATGGTGTATGAAGGGACAGAAGCGAAATTCACATGTACTGCTTCGACTGACCCCGAAGAAGTGGAAAATTTAATCATCCAATGGAAAAAAGATGACCAACTGATTGATTATCGAGCCGCTCAGAGAATGTTTCAAAATTCTATGGACAATTCGTTAACCGTGTCTGGAACACTCACCTTGGACACAGCCAAGTACACATGTGTGGCGACCAATGGTCTGGACAGTGAGGAGGCTGATGCCCAGTTGATTGTGCAAGGCGTTCCTGACCCCCCCGACACACCAGAAGTGCGTTGCCGTGAGCAGGAGAAGATCGCTGAAGTCGAATGGCAACCGGGGAAGGAGAACTACGCTCCCATTCTCAATTTCATCATCCAATTCAACACGTCATTTGCACCCGATACGTGGATCGACATTGCTTCTAATTTGTCTCAGAACACAAGACGTTATGATGTCGAGCTCTCCCCGTACTGCAATTACACGTTCCGAGTTCTTGCTCGTAATAAGATCGGAATGAGCATGCCTAGTAACCCATCTGCTGAAACATGCAGAACAGAACCGGAAGCGCCCCACAAGAACCCTGAAAATGTCATAGTGGAGGGAGACTACCCCGATGAACTGGTCGTTTACTGGACGCCCATGCCTCCAATAGAATGGAACGGTCCTGACTTGAAGTATATCGTTACCTGGGAGAGGATCGACCTGGAGCCTGGCGAAACACCCAATTCGGGCAACTCAGGTGTAGAACTGCCGGACGTCTACCACCTGGTCATTCCAACCGAACAAGGCCTTCATGGGATATACAAGCCCTACAGGGTGACTGTCAAGGCAAAGAATACTGTGGGAGAGTCAAGCGTACCTGCTGAGAATGTTATCGGGTATTCAGGAGAAGACACTCCCCTTGACAGTCCTCAGGACATTATCTACGATCCAGACACCCTCACGAGCACCTCGGTCATGCTAACCTGGCCGCAGGTCAACACCGAAACCGATCGCATTAGAGGGTACTTCAGAGGATACAGAATTCAATTCTGGAAGACTGCGGAGGGTAGAGACCTGATGAGAGAAAAAGACTTAATCCTGAACGTGTCCACCCCTTATCCGAGGCCCAGAAATAAGAGGGACATCGAGATGGTCGTTTACGAACTGACCAACCTGCCCCCATATGCCGAGATCGAAGTGCAGATCCGAGTGCTTAACAAGTACTACGCAGGTCCTCCCAGTGCTCCCGTCATTTTCACTACACTAGAGGGCGAACCAGGTCCTCCTGCGGCCTTTGACGTCCTCGCACGAGGCCCCACCCACTTTGACCTTATTTGGGAACTACCCCTCGAGCCAAATGGAGAGCTGGTCGGGTTCAATATTTCCTATCAGTCCATCACAGGCTTGAATCTTGGAAGACTGAAGTACAGAGAACCCATCGCAAATCCAGAGCAGAAACGTGCAAAACTGACTGGCCTCGTTCCCGAAACTGAATACAGAATTTACCTGTCTGCTGCGACCAAACTTGGAAAGGGAGAAGACATTTTCTTAGATGCTAAGACGACTCCTGCTGGACCCCCCAGTCCTCCAACGTTCGACATTCTTGAGGTTGATGAAAGATGGACCGTTTTGCAATGGGAACCATCCCGAACTGGCAGTCCCGGCAGCGTCTTTTATGTCCAATACAGGGCTAGAGGGTCGTACCTCTGGCTTAATGGGGCGGATGAGTCCCTGCATTACCGAGCAAATGTCACAGAACTAGATCCAGGGACCACCTATCAAGTCAGGGTGGTTGCAAAGAACGGAGAGGGCTATGAAGCCGCTTCTGAGTGGCAGGAGTTCACCACTGGAGGAATTGCCCCTGGTCGAATGGATCTTGGCTTCTCAGGCTGGTTCTACGGAATCTGGGCGTGTCTCATCATCATCATCGTGGCCGTGGTTCTCTTCTACTTCTGTAAGAGGAAGAGGGACGTTTGGTGGGAGAAGAAAGAGGGAGAAATTGCTGAGGCCATGTTGCAACTGCAAGCCGAAGATGCTGTGCGACAGTTGGGAATTGAAAATCAGTATCACTTGGGAAGCAGAGAAATGCTCCAGAGTGATATGAGCCATGCGTCAATGCCAGGGAGCGACTTTAAGGCACCTGTTGATAACATCGAAGATGATGATGAAGAAGACGATGATGATGAGGAGGATGAGGAAGAAGAAGAAGAAGAAGATTTAGGAGACAGTTTCATCGCCAAAGAAACCAAAGTAAAAACACCAAAATCGGAAAAGAAGAAAAAGGGTGAAGAGGTACAGAAAGCTGCTACTCCAACGACAAATGGTGCTCCAGCCTCTGGAAAGAAGAAGGACAAAACGCCCAAGGGAAATTTAACAGTTAAGCAGGAGAAACAGACGCCGAAGGCAGAGAAGTCGCCAAAGCAAGAGAAGTCGCCAAAGCAAGAGAAGTCGCCTAAGGGAGAAAAAACGCCAAAGGGAGAAAAAGGACAAAAGCAGACGCCAAAGGGTGAAAAGACCCCAAAAGAACAGAAGGAACAGAGTCCCAAGGGAAAAGGACAAACCCCTGGTAAAGGAGCTAAGAGGGTAATCGCTGGTGGAATCACTTTGGAAGAGGTCAAGGAAGGCCATGGCCCAGAAGCCAAGACTGGCAAAATGGTTCAAATGTACTACTTAGGAAAGTTGGTGAATGGACAGAAGTTTGATTCATGCTTAAATGGAAAACCATTCAAGTTCAGATTAGGAAAGAAGGAAGTCATCAAAGGATGGGATTTGGGAATTCAAGGAATGAAAGTTGGAGGCAAGAGGAAGCTCACAATTCCACCATCATTTGCATACGGAAGCCAAGGCATTGGACCCATCCCCCCAAACAGCACACTCGTCTTCGATGTAGAACTCAAAGCCGTCAGTTGAGAACTTAAGACTATTTAAAATTTTATGTGATTGACTTGCTGGTTATGTTCAATTGTTGATGAATTCTGAATTGAGTGCCTTTTAGCATTTATTATCTCATTAGAGAGTCATTAGAGTCGTGATACATTCAGTTATACTCATCTTTAATCTCATGTCATGATTTTAGTTCAAATGCGTTGGTGTACGTGATTTTGAGTATGGGAATGTCAGGTTTGAATGCATGGTTTACGTAATACGTATGTTATAATATTATATGCAAGTAATGGAGAAATCTAAATGGAGTCTCCCAATGGATCAATAAAGACTAAATGATTGGTGGATCCAAGTCATAAGCAATTCAATGAAGACCTCTTGTTCCTTCTTATTAATTAAAGCTCACTATATTTTATGGAGTCCCGGTAAAGCGTTTATATAAAGAGAATTGTGATTTAAATTTACTTGAAAATTATGTACTTTATAGGAGGATGAAAATTCAGGGATATTATAAAAACTGCATGTACTTACTATAAAGAATTTTGTCCTAATGAATTACACAATCTCGGAGAGTCCTATGCGTATTTTCATATTATTGTTTTTGACTTTATTAAAACTTCCAGGGAAGACTTACTGATATGCAGCTTGTTCAACTGCTATGTGATAATGTTTATAAGAAATGTCAAATAGAGATGTGAAAATGAATAAAAATATATCAAAAC

Protein: 1443 aa

>Neuroglian_protein

MLAFRIGESIEMPCVATGQPDPRYLWQMNGKDFDPSGNDGRVAIQPGVGTLIFSNPIERDEGIYQCLADNGHGVAVTIKVDLRVSYLEKFPSMTPRQFYPNLGEKLTLSCTRPASFPSPEVFWAIVLDDQSYSPVSLSDRISIDPEGNMHFANVLSSDSYSNMRYACFVQNQVMRQFVEGSYAFINPIGNQIQRFPPTSVYRDPTYQIALVGETWRVKCIFSGYPTPRVSWDREGMPMPPRKREESFGQELVIENVQLSDAGKYECLGINDDATVPDRYSFELAVESAPFWDQKPESFDAADNDTAVFHCSAIGDPEPLIDWFINGIPISKAEPNPRRVVERNVITYYNVIKDDAAVIQCNATNKHGYIFTNAYLNVLSERPVITEPPMVGMRAAEGQTVNLTCHVFGSPKPVVVWRKSGEQLTGGRFSVTEDGHLMIVDLSLVDAGSYTCNATNKLGSVAATGSLIVRRRTTIQMAPMDVMVYEGTEAKFTCTASTDPEEVENLIIQWKKDDQLIDYRAAQRMFQNSMDNSLTVSGTLTLDTAKYTCVATNGLDSEEADAQLIVQGVPDPPDTPEVRCREQEKIAEVEWQPGKENYAPILNFIIQFNTSFAPDTWIDIASNLSQNTRRYDVELSPYCNYTFRVLARNKIGMSMPSNPSAETCRTEPEAPHKNPENVIVEGDYPDELVVYWTPMPPIEWNGPDLKYIVTWERIDLEPGETPNSGNSGVELPDVYHLVIPTEQGLHGIYKPYRVTVKAKNTVGESSVPAENVIGYSGEDTPLDSPQDIIYDPDTLTSTSVMLTWPQVNTETDRIRGYFRGYRIQFWKTAEGRDLMREKDLILNVSTPYPRPRNKRDIEMVVYELTNLPPYAEIEVQIRVLNKYYAGPPSAPVIFTTLEGEPGPPAAFDVLARGPTHFDLIWELPLEPNGELVGFNISYQSITGLNLGRLKYREPIANPEQKRAKLTGLVPETEYRIYLSAATKLGKGEDIFLDAKTTPAGPPSPPTFDILEVDERWTVLQWEPSRTGSPGSVFYVQYRARGSYLWLNGADESLHYRANVTELDPGTTYQVRVVAKNGEGYEAASEWQEFTTGGIAPGRMDLGFSGWFYGIWACLIIIIVAVVLFYFCKRKRDVWWEKKEGEIAEAMLQLQAEDAVRQLGIENQYHLGSREMLQSDMSHASMPGSDFKAPVDNIEDDDEEDDDDEEDEEEEEEEDLGDSFIAKETKVKTPKSEKKKKGEEVQKAATPTTNGAPASGKKKDKTPKGNLTVKQEKQTPKAEKSPKQEKSPKQEKSPKGEKTPKGEKGQKQTPKGEKTPKEQKEQSPKGKGQTPGKGAKRVIAGGITLEEVKEGHGPEAKTGKMVQMYYLGKLVNGQKFDSCLNGKPFKFRLGKKEVIKGWDLGIQGMKVGGKRKLTIPPSFAYGSQGIGPIPPNSTLVFDVELKAVS

Domains

(3x)Ig: 10 – 77, 212 – 270, 489 – 562

(5x) FN3: 569 – 665, 673 – 764, 782 – 895, 900 – 987, 1000 – 1090

FkpA: 1299 - 1442

Clone

Partial ORF

Primers

Forward: CTGATGCCCAGTTGATTGTG

Reverse: TGACATTTGCTCGGTAATGC

Nucleotide# 1765 – 3264 = 1500 nucleotides

>Neuroglian_clone

CTGATGCCCAGTTGATTGTGCAAGGCGTTCCTGACCCCCCCGACACACCAGAAGTGCGTTGCCGTGAGCAGGAGAAGATCGCTGAAGTCGAATGGCAACCGGGGAAGGAGAACTACGCTCCCATTCTCAATTTCATCATCCAATTCAACACGTCATTTGCACCCGATACGTGGATCGACATTGCTTCTAATTTGTCTCAGAACACAAGACGTTATGATGTCGAGCTCTCCCCGTACTGCAATTACACGTTCCGAGTTCTTGCTCGTAATAAGATCGGAATGAGCATGCCTAGTAACCCATCTGCTGAAACATGCAGAACAGAACCGGAAGCGCCCCACAAGAACCCTGAAAATGTCATAGTGGAGGGAGACTACCCCGATGAACTGGTCGTTTACTGGACGCCCATGCCTCCAATAGAATGGAACGGTCCTGACTTGAAGTATATCGTTACCTGGGAGAGGATCGACCTGGAGCCTGGCGAAACACCCAATTCGGGCAACTCAGGTGTAGAACTGCCGGACGTCTACCACCTGGTCATTCCAACCGAACAAGGCCTTCATGGGATATACAAGCCCTACAGGGTGACTGTCAAGGCAAAGAATACTGTGGGAGAGTCAAGCGTACCTGCTGAGAATGTTATCGGGTATTCAGGAGAAGACACTCCCCTTGACAGTCCTCAGGACATTATCTACGATCCAGACACCCTCACGAGCACCTCGGTCATGCTAACCTGGCCGCAGGTCAACACCGAAACCGATCGCATTAGAGGGTACTTCAGAGGATACAGAATTCAATTCTGGAAGACTGCGGAGGGTAGAGACCTGATGAGAGAAAAAGACTTAATCCTGAACGTGTCCACCCCTTATCCGAGGCCCAGAAATAAGAGGGACATCGAGATGGTCGTTTACGAACTGACCAACCTGCCCCCATATGCCGAGATCGAAGTGCAGATCCGAGTGCTTAACAAGTACTACGCAGGTCCTCCCAGTGCTCCCGTCATTTTCACTACACTAGAGGGCGAACCAGGTCCTCCTGCGGCCTTTGACGTCCTCGCACGAGGCCCCACCCACTTTGACCTTATTTGGGAACTACCCCTCGAGCCAAATGGAGAGCTGGTCGGGTTCAATATTTCCTATCAGTCCATCACAGGCTTGAATCTTGGAAGACTGAAGTACAGAGAACCCATCGCAAATCCAGAGCAGAAACGTGCAAAACTGACTGGCCTCGTTCCCGAAACTGAATACAGAATTTACCTGTCTGCTGCGACCAAACTTGGAAAGGGAGAAGACATTTTCTTAGATGCTAAGACGACTCCTGCTGGACCCCCCAGTCCTCCAACGTTCGACATTCTTGAGGTTGATGAAAGATGGACCGTTTTGCAATGGGAACCATCCCGAACTGGCAGTCCCGGCAGCGTCTTTTATGTCCAATACAGGGCTAGAGGGTCGTACCTCTGGCTTAATGGGGCGGATGAGTCCCTGCATTACCGAGCAAATGTCA

Cloning info

PCR rxn on 05/05/16 using GoTaq polymerase mix

| Template | Initial denature | Denature | Anneal | Extension | #cycles | Final extension |
| --- | --- | --- | --- | --- | --- | --- |
| 2 hr cDNA | 4min @ 94C | 30sec @ 94C | 30sec @ 55C | 2min 15 sec @ 72C | 35 | 10min @ 72C |

**N-Cadherin**

Gene model: 10523 nucleotides

ORF: 499 – 7755 = 7257 nucleotides

>N-Cadherin_full

TATAAACTGAACTATGAAGCTCCAATGAAAGGGACACTGGTACGTGCTCCGACTGCCTCGCCTACAAGTTTTCGCCTGAATAACCAGGGAAGCCAGAGGAATGGTGCTACGCTCTCCTCAATGGAGATTTCATGAGAGACTCCAGGAGAATCTCGATCGCCGGTCCAGCAAGTGCCAGAGCAAACCAAATCACCAGTCTCTTCTGTTACAAAATAGACGTTGCTAGGAACTGTTTTAGCTCTAGTTTTTAGAAGTAGTTTTTATAGTGATGTTTTTAACCTGGATTTTAATATGTAACGCAACTCCCTAAATAGGAGGTTCGTCTAACATAAAGGCGTAATATAAACAGAAAAAGAAGAAGAACTCACTTAGATTGTTGGTTTCTAATCAAGAAGTTTTGCAAGATTAATGATATAATATAATCGTCATTTCATATTTTGCAGGATTGCGGAAGTGAAAGCCTACTCCCACCAAGGCTACCCCATCACCTATGAGCTGATGACAGACGCAGGGGGAAGTTCTAATGAGTTTGCGATCAATCCAAATACAGGAGTGATAGATTTATTGCGAGAATTGGATTACGAGAAAGACCCACAGCAATATCATCTGGTCGTCAGGGCAACCGAGAATGGAAGGCCGCCTAGATACAGCACTGTCAATCTGATCATCAACCTGGTGGACATAAATGACAACTACCCCATGTTCCCTCTCTCCCAGTATATTGTGCAGGGAATTGCGGAAACCGTCCCTAATGGATCTCATATCATTCAAGTGACTGCGACTGACCTGGATTCCGTAACTAACTCGCAACTGACTTACAGTGTCAGCAATGGAAATTTCACTGTGGAGAGTCTCAACAATATTGGGTACATCAAGACTGCCAGAACCCTCGACTTTGACCACATCTCAGACCACACTTACAACTTCACGGTCACAGCTACGGACAATGGCAAACCTCCGTTAAACGGCACAGCGATGGTACGAGTGACAGTCACAAACGTCAATGATGAAGACCCCCAATTTACTCAGGCTGTAGAACACGTCCAAGTCAGTGAGGACGCCACTCCCAACACTGTTGTCCATGTTGTTCAAGCTTATGATCCGGACGGAGATGACGTGACCTACAGTTTTGCTGGAGAACGCCTGACGGCAGGACCGTTCAGAATCGACCCGACGTCAGGAATCATTACTCTCGTTGGGCGCTTGGACAAGTCAAAAGTGTCTTACAAATTGAACATAACTGCGACTGACAACGGGAGATGTTGTGGCGGAAAGACGAGCAGAAGTAATCGTGGTTTAGTAATCGTTGAGGTCAAAGACGTGAACAACAATGCGCCAAGGTTCCCCGAGTGTTCCAAATATAAGCCGACTGTATTAGAAAGAGCTGATGTTGGGACTTCTGTGACGCAGGTGCGAGCATTGGACTCGGATACTGGAAACAACGGAAACGTCACATATTCCTTGGTCAAATCATTAGACCAAGATAGCGATAGATTTGGAATCGACCCGATCAGTGGAATACTGGAAACTTCAGAAGTGTTCGATCGAGAAGCGAGAACCGGACACACGGATTACGGAGTCACAGTGAAAGCGGAAGATCAGGGGAGTCCGAGATTGGCAGGATTCTGCACGTTTAGAGTCCACATAGGGGACGTTAATGATAATCCGCCTGTCTTTGATTTCCCCAGCTATTCCACCAGCTTGGAAGAGAGTTCACCAATCGGAAAGAGAGTTTTGCAGGTTTATGCAACAGACAAAGACGCAGGTGAAAACGGCCAGGTGGAGTATTTCATGAAGAATGACCCCTCAGGGTTCTTTGAAATTAACAAACACTCCGGGTGGATCACTGTGGAGAGGGCTATGACTGGCACTGAGGAGGTGAAGCTCATCATTGAAGCTAGGGATCAAGGAGCGATCCCAATCTCCTCAGATGTGGAGGTTGACATTGAGATCACACAAACAGTGAACGCATACCCCCAATGGGTGCAGGACTATTCGGACGAACCAATCAAATTCAGCGAAGGTGTCCCCGTCAACTACATCGTCAAGAGATTGAAGGCAATCTCAGCAGTCCCGGATGGCACCGTCAACTACATCATCAAACCCGGAGAGACCCCAGAACAGAACGGAAGCCCCCAGAGTTTCTACCACACCATCAATGCCTCAACTAACGAAATGTTGCTCAAAGTTTACCGTGCACTTGACTACGAATCTATTCCCAGATACACCCTCACTATTAAGGCCTCTAATCGTGCCACAAATTCCTTGCACAACGCCACCCGTATGACCATTGAGTTGATTGACAAGAATGACGAAATCCCGCAGTTTGTTGGTCTGGATGAAAACGGTCGTTACCCTGGAAGTGTCTCTGAAAATGTGTCACCAGGAGCAGAAGTCATTGCGATCACCGCTACTGACAGAGACATGTTCCCCGACTATAGGAAGATCACATACAGTATCAAACCGTATGGTATTGACTACGACAAATTTGCCATCAACCCAGAAAGTGGCATAATCACCACCCGAGAAATGTTCGACCGTGAAAAGAAAGATGAATATTATGTCACAGTAATTGCACAAGATGGCGCTCCATCCAACCGACCTTTCCACTATCCACCAGGGTCCCCTAATCAAGGAGAAGCGGAGGTGCAAATCAGAATCACAGACAAGAACGATAATGCTCCGTACTTCAAACAGGAGTTATACCAGGCGTCTCTACCAGAAAATACTGATGTCGGAACTATTGTTATATCTGTCACGGCAGAAGATGAGGACGAAGAAAATCAGCTGACGTACTCCATTACAGGAGGTAACATGGGAAATGCCTTCGAAGTCATTCCAGACCTCGGCCAGATCAAAGTACGGGGTAATCTGGATTACGAAGACGGACCTAGGGAATACCATCTGACGTACCGAGTGTTTGACGACAAGTTTTCCAACACGGCGACAGTCGTAGTTCGTATCACGGATGTGAACGACAATCCTCCGAGGTTTGAACCAGCCGAGTATGAAGTGACGGACATCGTGGAGGAGGATCCCACAGTTGGCCCTACTAATCCCAAATTCCTTGTCAGGGTGAACGCTGAGGACCCAGACATAGATCGCCGCTCTAACATTCGCTACAGCTTGACTGGACAATTTGCAGACCAAGGATACTTCACCATAAACGAAGTCACTGGAGATATCTACGTCACAAAGAGGCTTGACAGAGATAAGCCAAGTGGGCGCTCAGTGTGGAATTTTAACGTCTTAGCTCACGATGAGCCTGGATCCGGACAGTCGTTGACAGGTTATGCAATAGTTCAAGTGAAGCCCAAGGATATAAATGATAACCCTCCTGAGTTTGATGGCAATCGATTAGTCGGACGAGTTTTGGAGCATTCGAATGCTGGAGTGTCTGTTCTAACAGTCATCGCCACTGACGTTGACAATGGCCATAATGGAACAGTGGCGTATTCTCTGAAACAAGTCCCGGTAAAGGACGGCACAGCCCTATTTGCTATCAACCCCAACACTGGCTTAATCACCACAACAATGAGTAATGCTCTAGACAGAGAGACGCAACCAGAGTTCAAGATTATTGTCCAAGCAAGAGATCAAGGAACACCGGAAGCAAGATCAGCGAGTGCCACTGTCACTATTCACGTGCAAGACATCAACGACCACCCTCCGAGCTTCACCCAAACAATTTACCACACTTCCATGTCTGAGAATTACCCTGTAGGGTCGTCAGTCACATCAGTATCTGCGTCTGACCCTGACGTAGGAGCAAATGCTAAGATGACATACACCTTAAAAGAACAAGATAGGGAGTACTTCTACATGTCCAGTGTGGAGGCCACTAATACTGGAGTGCTCAAAGTGTTCAAGCCTGTGGACTTTGACACCCTCGCCAACCCTTTCTTCAACCTGACGGTGTACGTCAGTGATCCAAACACGAGTCATCTGCACACGGCTTACATAGAAGTGGAGATCACAGATTACAATGACAATCCGCCGGTGTTTGCTCCGAATAGTAGGAAGATTAACATTGAAGAAGACGTGGAAGTTGGATCGGCCCTCGCTACTTTCTCCGCTAATGATAAGGACACGGGGCTTAATAAGGAGTTTGACTATGCTATCGACAGAGACACAGACTCCCGAAGACAGTTCTCCATTGATGCCAATGGCATGGTCAGAGTGGCCAGACCTCTCGACAGGGAGACTACCCCAGTCCACAGAGTGCATATCCTGGCTATGGATAAAGGTGAACCAGCACAAACAGGAACCGGAACTTTAATCGTCACATTGAAGGACGTCAATGACAATCACCCAGAGTTTGACCAAGATTACCGCCCCGTGATTTACGAGAACGAAGCCGAGGGCTTAACGGTGGTTACGATAAGTGCTGTAGATAAAGACACCGCATCCAACGGACCTCCGTTTGAATTCTGGTTACCATGCAAAGGGAGCTGTCCGTGTCCGCAAAATCCGACTTGTCACGATTTTGGATTCAAGTTCATACCAGGTGGAGGTCTGAACGGCCAGGGCTCAGGAATCGTCACAACTAAAAGGACGTTTGACCGCGAGAAGCAGAAGTATTTTTTGATGCCCATAGTCATGAAAGATACAGGCAACCCCCCTGTATCCGGAACCAACACTTTGACGATTGTCATTGGGGACAGGAATGATAATAAGCACAACCCTGGACATAAGAATATATTCGTCTACAACTACAAAGAAGAGTTCACTAATGCTCGCTTAGGAAATGCTTTTGCCGAGGATCCTGATGATTGGGACAGAGATGACAAAGCCTTTACGTTTGTCGGCCCCGAAGCTGGCAAGTCGTTCCACCTTGACAAGTTAACAGGTGAACTCTCGATGAAGGGGAAAGTACGAGAAGGAAACTACAACTTCCAGGTTCACGTTCATGACAACGCTTGGAAGAGAGACGTCGTGTCCTCAATCACCGTCAAAGTCCGAGAGATCACTGATGATGCTGTGCACAACTCTGGATCCGTGAGATTCTCTGGTATCACTGCTGAAGAATTTGTACAGCGTCCCAAGGTCGGCCCGTTAATAACCGATTACGGATCCAGCAAATATGACATGTTCCGAGAGTTAGTGGCTGCAAAATTGGGCGTTCCTATTCATAACGTTGATATATTTACTGTGATGAACCACCCCACCTTACCCTTGACTTGCGACATCCGATTTGCTGCTCACGGATCTCCGTATTATCGTCCTGCGAGGCTGAACGGAATTGTCAATGAAAATAAAGACGAGTTCGAGAACTTGCTTGGCGTCCAAATCTTGATGGTCCCGATTGACGAATGTATGGAAGAAAAATGCGAAGGAGGTGGCTGCTCCAATCGTCTTGTTACTAGTGACCAACCTCTGCTTGTTAACACCAACGGAACGTCATTAGTTGGTGTGACTGCATTTATCGAAGCCGACTGTCAATGCTCCGCAAGGGTTTTTGAAGAAGACAATTCATGCAAGCCAATGTCATGCCTGAATGGGGGAACATGTCATAAGAGACTCAATGACCACTACTGCGAATGTCCGCTTGGTTTCGATGGCCCCCGGTGCCAGCAGACCCACCACAGTTTCCACGGTGAAGGATGGGCGTGGTACGAGCCTCTAGCCCAATGCGAATCAAGCCACACTCACTTTGAATTCATCACAACTCGAGAGACTGGCCTGCTGCTATACCACGGCCCCATGAGAGAACTGGAACAAGGGGAACAGAGGGATTACATGCTGTTGGAGTTGAAGTTGGGCTATCCAGTGTTGAGGATTAACCACGGTACGGGAGAAGCCAAGTTGACCATCAACGGCAAGGATCATCGCAATCAGTTGAGGTTGGATAGGTTAAATGACGGCAAGTGGCACAGGATCGACATCTTCAGAAATGGAAAGCGAGTTTCGATGGTAGTAGACCAGTGTCGTTCGGCCGTGATTTACGAGAACGAGAACGACCAGACGTCAATTGAAGACAGGGAGCCGTGTGAAGCTACAGGGGACACTCAAGGAGACAACTCATTCTTAAACGTGAATGCCCCTCTCCAATTGGGAGGGAGGAGTAACCCCTTCATTACTTACCCCGACAATGTCCTTCTGCAGAATTTCCACGGCTGCATCAAGAATCTTGTGCATAACGGAGAGGTGTATGACTTGAGCATCGGCCGTACTGGGGAGCACTTCAATTCCCAAGATGGCTGCCCTGCAGAGGAAGAGATATGTGGTTACAATAGCATGAATGGTCCGAAATGTGGCTACCACGGGAAATGTATCGCTACTCTCGTTGGCGATTATACTTGTGTATGCAAACCAGGGTATAGAGGAATGACATGCGATATACCAACAATGATCAAAGACTTCAGAGACAGGAGTTACATAGAGTGGGACTTCAAGGAAGGGTTCTACCGCCAGTTGGAGAAGAGACCTTTCAGGACAAATTTACAACTTCAGTTCAGGACTCGCACGGACTCCGGTCTGCTGTTCAAAGTCCAAAATATTCAGAAATCAGAGTACATGATACTGGAGCTCAAAGACAGAAAAGTTCGGTTCCGTTACAACCTTGGCAGTGGGGAGAACGAGATCGGCTTGAACCAAGTGAATGTGTCGGACGGCTATTGGCATACAATATATGTGGAGAGGATTGGTAAAATGGCCGTTATAAAACTAGACAGCGGAGAAGGACGCTACTACAACATGAGTATTGGAACTATAGGGGGACATCTGCAGATTCTGGTTGCTAGGCGACAGTTCTATGCTGGTGGCGACGTCAAATTCCCATCGTCGAAAGATCCAGCCATTGTTGATTACGACTACAGAGATAGTTGTATGCAAGACGTCAGGTACGATGGCGAGTGGTTGCCGATGGATATCGCAGAAAATTCGCAAAGCAGAGCAGCGGAGCTCACGAGATTTGAGAATTTGCGAGACCATTGCAATTCTTCGTCTTGTGAACAGTTAGCGTGCCAAGCTCCGAGGGTCTGCTGGGACATGTGGAGGCAAGCAAAGTGCAGATGTCCCCACGGTCAACGCTGGATCGGACTTCACGGGGAATGCGTCAACATTAACGAATGTGTGGAGAAAGACCCCTGTTACCATGGCGACTGCATAGATTTACAGAGAGGCTTCCGTTGCCGTTGCCATCATGGTTGGCGTGGTGACCTGTGCGATGTCCAATTAGCAGCTGCGGTGGCTTTCATGAGTACCGGGGCCATCTTCGCCATTTTAATATGCATCTTAGTTCTGCTTTTGCTCATAATCCTATTCATAGTATACAGTCGTCGACGGCGACCGGAAGCTTTAGTGGTTGCCATGGATCCTGACGATGATGTCAGAGAAAATATTATACATTACGATGAGGAGGGAGTTGGAGAGGAAGATCAAGATGCTTATGACATCAATCGGCTTAAAAAACCGGTGGATGAAAACGCTCCAAAAAAGCCACTTATAGATGAACCCATCATGGCCCCCAAGCAGAAGGCCAGGAGAAATTTTATCCCAGGGGAAATGACGGACGTCGGGGACTTCATCACGAGTCGGCTGGACGACGCAGATGAGGATAACTCCGCCCCTCCCATCGATACCCTGCACCACTTTGCCTACGAGGGCGAGGGTAGCATAGCGGGTTCATTATCGTCTCTAGCTTCGACTACGGAGAGTGAAGACCAAGCTTACGACTACTTAAAAGATTGGGGTCCACGATTCCATAAGTTGTCTGAAATTTACGGAGGTTCAGGCGCCGACGATACGTTATAGTACACCTTGCACAAAACGGACGGTGCTGTATGAACGTTTAGTGACTTTAATCTGATTCAGTGAATAAATGAAACATTCAATTGATACTTATTATTATTTAATAATGCCACTCTGCAACAATGTGAGGAGTGATGTATGATATCATCGATACGTCTTAGTCATATTACGATGATAAACTTCAACTCTCTCGGATTACTCGATGAGGAATTTATAAAAAGTCTACAACTGGTGTTTATGTGAATTCTAATGGTCAACCATTACTTCTGCGGCATCAGCTATATCAATATATCTGTGAGATATGGACATGTTTGATTTTCCAGAACTTAATGAGATATTAAGAGAGACTCCCGACTTCTCGTCTTCTGAAAACGCAAGTAAAACTTGTTGGAACTTCTTCACAAACTTTAAGGATAAACTTAAGAGAAAACTTGGAGATAAACTAAGGGAGAAAACTTGCGGTTCAAGCTTGTCAAACTATCTTGTCATCTCATGAACTACTTTATATTTTACATGAGCTGAAATGATCTGAACGGGAGAACAAATGCCGGAATATCAAATTAACATGTGGTGGACACTGTTGCATTCCAGGATTTAAACGATTACTCAAATCAAACAACAATGTATTAAACTGTGGAACTACGTCTTATGAAGTTTAAAATGTCTTCATTTGTCTTTAACTGTCCAGAAATCAAATGTTCTGTCTTCGTCTAAGGTTTTAAGTATGGTACAAATGTTCATACGCACATTTGTGGTTCGAATGACTTTCATGCGACCTCTAGCAGCAACGCATATGCCCTCTAGTGTATACATAGACAACTAGCGCTCCTAGTGGATATAAATGGATAATAGAGACAATGCCAAATAGTCAAATTGCTCGCTAGAAGGTCTCCATGAGTGAGAATCTCAAATTCTCCATTTATAAAGGCTTCATAAAGATGCAAACCTAGTGGATAATATGTACCATAGAGTCATTTTGGAACATAGATCTTCAGATTCGTTTCCATGACAATCACGATTCTTTATGTCACAAATTCTCATAAGCTACTCTTAGCTTGATTCTCGTTCCTCCTTTCGACAATCTACGTATGAACTCGGAAGAATGGCTTCTTACGAACTGGGAGATCTCATGGTCACTTCAAATTTGATTTTGTCGTTATTTACTCCTCCAAGACTACTTAATTCCACGCAATTGTAAACTGAATTTCACCAACACTCCCGAATTGTACAGGGACCATTTGTTCACAGATGATCAAATCGAACTGAATCAAAATGATTTCACACCTTGCCTGAAAATTGGTTATTCTATTCCACTTGTGAAATTTTTGTATTCATAAATGCAGCCATTTCGATCCTCAAATTCATGTATGTTACTTCTGAAGTAAAGTTACAAATTGTTGACGAACGATATCCGTACAAACTGTTTACGAAAAGTCCTGTTTTTGAAAATGTTTACAAAAAGTCCTGTTTAGGAAAAGTACTGTTAATGAACTGTTAAAGCATTTTTGAAGATGTTACAGTGTTTATATAATTATTCAAATTTTGTGACCTTTCTATGTTCTGAGCCATGCAATGGATTCTCATAACAAATCGATGCATTTATATTAAACAATTGATGATATTTGTCGAAAAATAAAAATTGAAATGTATAGAGAACACTTCGAAACTTAAAATACTTAGGTCTTATCATTTTGATATAAAATTGCTCGTGCATTTGAAGTAATTTTGAAAATTGTCAGTTGTTGAAATTCCTCCCATTTTCTTGATATTCAAAATTACTAGTATGTTCACGTTCAAACACAAGAACTGAATGGTATATCGACTGAAAGTCATCATTTTAAAATGTACATATTTTCATATTTACTTCTATAGGTAATTTTCATGCAGTTTCACAAATAATCTGTAATGTTAATTTTCAATTCGCCGTTTATGTTTTAAGAAATGCAAAAACTGGTCATCAGCTGTATGAAAATATAAAATGTAAAAACGATGCTTTATGTACGATCAGTATTAGCACAAAAGGCATGTAGACCAATCAGGTATGCTAAAAAATAATCATCAAATATGTAATTCTTTATCATTTGCTTATATGTGTCATGTAATAGAGATATACGAGTTTAGAGATAAGCGAGGTTTTGCTTCACGTATATGTATCCCATATACTTGTCCCGTATACGTGTGCAGGTGCTATCTAATTACATTTAATTGTAGTCATGGCAACCTGTATGTTCCAGGGACCCGTATACGTGTATCAAAACCTCTCCATGCTGTCATTGTGGCTGGAATAATTCAACTCAATTAATTAATATCTTAATAATTAGTTGCTTTAAAATGCACATAGAGTCATACACGCGGCTAAACACTCAGTATTATATCCACAAACGTTATTCATATTACCATATTGCTCTGATTGTGATCTTGTGCAAGAGCAGAATCATTATCAAATATATTAATTTGATGATATATCTTAATTCACTGATTTCAATCGCAGGAATACCGTACAAATAAGTGCCACGCCAGATAAGTGCTTATATCCGAGTATATACGGTAATTTACATGTTCCGTGTTTCAACAGTTGATTATTCCGCAATTAATTGATTAAGTTCCGTGTGATGAGTCATTGTGTGAAGATTATGAACTTCGCAACCATGACAGTAATTTGTTATTTGCAAAATATTAACTGAATCAATTTAGCATATGCCCACATGCCCCACAAGAACAATTTAATTCAATCAACTATGTATGACATGCATGATATCATATTGAATCAA

Protein: 2418 aa

>N-Cadherin_protein

MTDAGGSSNEFAINPNTGVIDLLRELDYEKDPQQYHLVVRATENGRPPRYSTVNLIINLVDINDNYPMFPLSQYIVQGIAETVPNGSHIIQVTATDLDSVTNSQLTYSVSNGNFTVESLNNIGYIKTARTLDFDHISDHTYNFTVTATDNGKPPLNGTAMVRVTVTNVNDEDPQFTQAVEHVQVSEDATPNTVVHVVQAYDPDGDDVTYSFAGERLTAGPFRIDPTSGIITLVGRLDKSKVSYKLNITATDNGRCCGGKTSRSNRGLVIVEVKDVNNNAPRFPECSKYKPTVLERADVGTSVTQVRALDSDTGNNGNVTYSLVKSLDQDSDRFGIDPISGILETSEVFDREARTGHTDYGVTVKAEDQGSPRLAGFCTFRVHIGDVNDNPPVFDFPSYSTSLEESSPIGKRVLQVYATDKDAGENGQVEYFMKNDPSGFFEINKHSGWITVERAMTGTEEVKLIIEARDQGAIPISSDVEVDIEITQTVNAYPQWVQDYSDEPIKFSEGVPVNYIVKRLKAISAVPDGTVNYIIKPGETPEQNGSPQSFYHTINASTNEMLLKVYRALDYESIPRYTLTIKASNRATNSLHNATRMTIELIDKNDEIPQFVGLDENGRYPGSVSENVSPGAEVIAITATDRDMFPDYRKITYSIKPYGIDYDKFAINPESGIITTREMFDREKKDEYYVTVIAQDGAPSNRPFHYPPGSPNQGEAEVQIRITDKNDNAPYFKQELYQASLPENTDVGTIVISVTAEDEDEENQLTYSITGGNMGNAFEVIPDLGQIKVRGNLDYEDGPREYHLTYRVFDDKFSNTATVVVRITDVNDNPPRFEPAEYEVTDIVEEDPTVGPTNPKFLVRVNAEDPDIDRRSNIRYSLTGQFADQGYFTINEVTGDIYVTKRLDRDKPSGRSVWNFNVLAHDEPGSGQSLTGYAIVQVKPKDINDNPPEFDGNRLVGRVLEHSNAGVSVLTVIATDVDNGHNGTVAYSLKQVPVKDGTALFAINPNTGLITTTMSNALDRETQPEFKIIVQARDQGTPEARSASATVTIHVQDINDHPPSFTQTIYHTSMSENYPVGSSVTSVSASDPDVGANAKMTYTLKEQDREYFYMSSVEATNTGVLKVFKPVDFDTLANPFFNLTVYVSDPNTSHLHTAYIEVEITDYNDNPPVFAPNSRKINIEEDVEVGSALATFSANDKDTGLNKEFDYAIDRDTDSRRQFSIDANGMVRVARPLDRETTPVHRVHILAMDKGEPAQTGTGTLIVTLKDVNDNHPEFDQDYRPVIYENEAEGLTVVTISAVDKDTASNGPPFEFWLPCKGSCPCPQNPTCHDFGFKFIPGGGLNGQGSGIVTTKRTFDREKQKYFLMPIVMKDTGNPPVSGTNTLTIVIGDRNDNKHNPGHKNIFVYNYKEEFTNARLGNAFAEDPDDWDRDDKAFTFVGPEAGKSFHLDKLTGELSMKGKVREGNYNFQVHVHDNAWKRDVVSSITVKVREITDDAVHNSGSVRFSGITAEEFVQRPKVGPLITDYGSSKYDMFRELVAAKLGVPIHNVDIFTVMNHPTLPLTCDIRFAAHGSPYYRPARLNGIVNENKDEFENLLGVQILMVPIDECMEEKCEGGGCSNRLVTSDQPLLVNTNGTSLVGVTAFIEADCQCSARVFEEDNSCKPMSCLNGGTCHKRLNDHYCECPLGFDGPRCQQTHHSFHGEGWAWYEPLAQCESSHTHFEFITTRETGLLLYHGPMRELEQGEQRDYMLLELKLGYPVLRINHGTGEAKLTINGKDHRNQLRLDRLNDGKWHRIDIFRNGKRVSMVVDQCRSAVIYENENDQTSIEDREPCEATGDTQGDNSFLNVNAPLQLGGRSNPFITYPDNVLLQNFHGCIKNLVHNGEVYDLSIGRTGEHFNSQDGCPAEEEICGYNSMNGPKCGYHGKCIATLVGDYTCVCKPGYRGMTCDIPTMIKDFRDRSYIEWDFKEGFYRQLEKRPFRTNLQLQFRTRTDSGLLFKVQNIQKSEYMILELKDRKVRFRYNLGSGENEIGLNQVNVSDGYWHTIYVERIGKMAVIKLDSGEGRYYNMSIGTIGGHLQILVARRQFYAGGDVKFPSSKDPAIVDYDYRDSCMQDVRYDGEWLPMDIAENSQSRAAELTRFENLRDHCNSSSCEQLACQAPRVCWDMWRQAKCRCPHGQRWIGLHGECVNINECVEKDPCYHGDCIDLQRGFRCRCHHGWRGDLCDVQLAAAVAFMSTGAIFAILICILVLLLLIILFIVYSRRRRPEALVVAMDPDDDVRENIIHYDEEGVGEEDQDAYDINRLKKPVDENAPKKPLIDEPIMAPKQKARRNFIPGEMTDVGDFITSRLDDADEDNSAPPIDTLHHFAYEGEGSIAGSLSSLASTTESEDQAYDYLKDWGPRFHKLSEIYGGSGADDTL

Domains

(13x) Cadherin_Repeat: 5 – 65, 73 – 170, 181 – 278, 288 – 389, 397 – 486, 503 – 601, 619 – 727, 736 – 828, 836 – 945, 955 – 1056, 1064 – 1165, 1176 – 1270, 1278 – 1392

EGF: 1658 – 1692

LAM: 1697 – 1881

EGF: 1919 – 1946

LAM: 1955 – 2117

EGF: 2188 – 2273

Cadherin_C: 2261 - 2412

Clone

Partial ORF

Primers

Forward: CATGAATGGTCCGAAATGTG

Reverse: TTTTTGGAGCGTTTTCATCC

Nucleotide# 6237 – 7438 = 1202 nucleotides

>N-Cadherin_clone

CATGAATGGTCCGAAATGTGGCTACCACGGGAAATGTATCGCTACTCTCGTTGGCGATTATACTTGTGTATGCAAACCAGGGTATAGAGGAATGACATGCGATATACCAACAATGATCAAAGACTTCAGAGACAGGAGTTACATAGAGTGGGACTTCAAGGAAGGGTTCTACCGCCAGTTGGAGAAGAGACCTTTCAGGACAAATTTACAACTTCAGTTCAGGACTCGCACGGACTCCGGTCTGCTGTTCAAAGTCCAAAATATTCAGAAATCAGAGTACATGATACTGGAGCTCAAAGACAGAAAAGTTCGGTTCCGTTACAACCTTGGCAGTGGGGAGAACGAGATCGGCTTGAACCAAGTGAATGTGTCGGACGGCTATTGGCATACAATATATGTGGAGAGGATTGGTAAAATGGCCGTTATAAAACTAGACAGCGGAGAAGGACGCTACTACAACATGAGTATTGGAACTATAGGGGGACATCTGCAGATTCTGGTTGCTAGGCGACAGTTCTATGCTGGTGGCGACGTCAAATTCCCATCGTCGAAAGATCCAGCCATTGTTGATTACGACTACAGAGATAGTTGTATGCAAGACGTCAGGTACGATGGCGAGTGGTTGCCGATGGATATCGCAGAAAATTCGCAAAGCAGAGCAGCGGAGCTCACGAGATTTGAGAATTTGCGAGACCATTGCAATTCTTCGTCTTGTGAACAGTTAGCGTGCCAAGCTCCGAGGGTCTGCTGGGACATGTGGAGGCAAGCAAAGTGCAGATGTCCCCACGGTCAACGCTGGATCGGACTTCACGGGGAATGCGTCAACATTAACGAATGTGTGGAGAAAGACCCCTGTTACCATGGCGACTGCATAGATTTACAGAGAGGCTTCCGTTGCCGTTGCCATCATGGTTGGCGTGGTGACCTGTGCGATGTCCAATTAGCAGCTGCGGTGGCTTTCATGAGTACCGGGGCCATCTTCGCCATTTTAATATGCATCTTAGTTCTGCTTTTGCTCATAATCCTATTCATAGTATACAGTCGTCGACGGCGACCGGAAGCTTTAGTGGTTGCCATGGATCCTGACGATGATGTCAGAGAAAATATTATACATTACGATGAGGAGGGAGTTGGAGAGGAAGATCAAGATGCTTATGACATCAATCGGCTTAAAAAACCGGTGGATGAAAACGCTCCAAAAA

Cloning info

PCR rxn on 07/28/16 using GoTaq polymerase mix

| Template | Initial denature | Denature | Anneal | Extension | #cycles | Final extension |
| --- | --- | --- | --- | --- | --- | --- |
| 2 hr cDNA | 4min @ 94C | 30sec @ 94C | 30sec @ 55C | 2min 15 sec @ 72C | 35 | 10min @ 72C |

**Echinoid**

Gene model: 3184 nucleotides

ORF: 484 – 3183 = 2700 nucleotides

>Echinoid_Partial

TTTGATGGCTGCTCAGTTGTCTTCTTGCAGTGAAGGGGTACGGACGCATGATTACAAAATTGCTGGATTTGCGTGGTTTTAAGGATGTGACATCGTCGGAAAACATTGCTAGGATGTAGCTAAACATCTCCAAAATGGATGCCTGTGTTTTTAAAGGCAGACGAAGTGTTTCAGTGGAGTTTTTGGACCAAAACATCTACACGTGTTTTGGCTGTGACAGTGTGAGGTGACCAGCATGGATTTGTGAAAAATTTTCAAGAAATCAGTATTTTCAGTGAATTTCTGAATTAGACACAACATACACTTGTCTTGTTATTATTATTACACAAAACTGGATTGTGCCATCAGAGTGGATACCTGGAGCTGGATAAATACGTCATCATATTTTGTGTAATAATCAATTAACAATTAAGAACCAATTAGTCACTTTTGAGGTCCTCATTATTGAAAAAATTGTGATAATTCTTGAATTTTCCCAATACTATGGATCCTGCAAGTATGAAAATGGAAATATTACGATTTGTGGTGTTTTTGATATCAACAGTGGCTGTCTACAGCCAAGATCCAGGGTGGATCGAAAAACCAATTAACACATTCGTTAATGAGGGGGGAAATACGACTTTGAAATGCAGAATTTCAAATAGGGGGTCAAGACAATTGTCTTGGAACAAGCAAAATGGAGGTTTACTGTTTGTGGATGGACAGAAGTGGACCACCAATGAGAGGTATTCGATTTTACCCCATCCAGAGGGATATGACCTAAGGATTACCAATTCACAGAGGGAAGAAGATGGGGAATACAGTTGTTCCTTGCAACTCAGTGATCTGCAGGAATCAGTGAGGGTTACTGTTGTGGTGATTCCTGGCCAACCTTCAATACAACCTCCTGGACCTTTAGCTGAAGGAGAGACACTCAGTCTAACATGTTCTTCTATAGGGGGCAACCCCCCTCCTAGTGTCCTCTGGAAAAAGAATGACGTCACAGTGTCAGAAGGCATTGTGACATCACCACCAGGTGACACATTCGGGGTGACATCTAGTGTATTAACGACAGTCGTTTCTCGACTGGATCACAGAAGTAATTTCACTTGCAGCGTCGAGAATGACGCAAACATTGGATCGCCCCGAAGGGTCTCCGCCATCATTGCCGTTCAGTATTCACCAGTTATCTCATTTGGACCTTACAATCCGTTGACAGTCATAGAAGAGGAGAGGACCACCTTGACCTGTGTTATAGATGCCAACCCACTCGCCACCGATGTCCGATGGATTAAAGATGGCACCGTTATTGGAACGGGAGTGGAGTACATGTTTCCTTCAATTCAAAAAGCAAATTCCGGAATTTTTTCCTGCCAAGCAGGCAATTCCATCGGGACCAAGCGGGAGAATGTGACGCTACATGTGCAATATGCCCCAAAGGTGGCGCTGCGCATGTCAAATTCTGTAATTGGGTCAAGGTCATTAACAATAAACGAAGGTCAGAGGATTAACTTAACGTGTGTCGTAGACGCCAACCCAAAAATATCTGGCGACATAGAATGGACGTATAATTACGGACCTGTGATTTCTTACGGACCCTCTTACATAATTGAGTCCGTGAGTCGGATGAATGCTGGGAATTACTCGTGTAGGGCACGGAATACGCTAACGCCCTCTGGCAGACCGTCGTTGCAACAGACGGGAAGTACGACGCTCGAGATACTTGTTCAATATTCACCCGGGGCAGCAGAAGTTTCAGGACCGGATTTCGGATTAAAAGGACAATCATCAACGTTAGTCTGCAAAATTAATGATCCTGGTCTACCAACAGCATCTTTACAGTGGACCAAATTTGGGTCAAATGAGGTCATCAGGGAGGGATCAACCTTGACCTTTGACCCAGTTTCCCTTTACGATAATGATGATTACATTTGCACTCCCAGAAATGAAGTTGGTGATGGAGCGCCCGATACATACAGGCTTATTGTTAACGAAATACCTGAATTTGTGCCAACCGAATTCCCCGCCACAAAAACCGTCCAAATCCACCAGAGGGGGTTGGTATTAACATGTAAAGCTAGAGGGCGCCCTGAACCAAGGATCACATGGTACAAGGACAACAACGCCCTCACTGAATTAGACGGGTTCTACAAAATTGAGACGACCACAAGACCGGTTGATACCCACAGCTGGAATGTTACCAGCACACTATATTGGAAAGGTCCATCAAGAACACCAGAGCTGGACTCCTTACGAATAGCAGATGCAGGAACGTACAGTTGCCGTGCGAGCAGCGAAATAAGCCAGGATGTCCTCTCTCAGGAGATGAGGCTCAATATACAATATCCCCCTGTGATCAACATCAGCATGGACAAAGTGGCCATTGACATCAACCAAACGGCCATCTTGAATTGCCAAGCTCATAGTGTGCCCCCCTCAAACTTCACTTGGTACAGGGGCAGTCAGGTGCTGACCCCTGGTGGTAGAATGAGCATAATGGAAGCGATGGTTCCGAGTGCCTTCCCCTACAGGACAACCTTGTCCATTCAAGATGTCGTCATGAGTGATCTGGCGTCGTATCGTTGCGAGGCTATAAATGCCATTGGGCAGAATAGTTTCAGTATCCAGCTTGTAGTGAGAACAATTCCAGACCCCCCTTCAAACCTAAACGCCCTCGCTCGTACTTGGGAGTCTGTAGAGCTGCAGTGGCAGGCTGCTTTTGATGGAGGATACAGTCAACATTTCATTGTGGCTTTTGAGTCATCGCATCATTCCAGAAACAGAGTCAGAGTCACCCCTCCCCATGCTACTATCTTCAATGTTACAGGATTATACCCAGATACAAACTACACTTTTAGGATATTTGGAGAAAATGAATTAGGAGGTGGAGATTATTCCAAAAGTGTTACCATGACAACTTCTGCATTTTCCATTGACCCTCCACTGGATGTCTTATACCATAAGAGTGACAGAACTCTCACTTTTAAATCTCCAAGTTCTAACTATTGCGGACGAGTGGAAAACTTTGACGGCAGACAGTGGAGACTTTACGAACAGTGCGCAAATTCCTCCGGTAGCAAAATCAAGGTCGCTGAGCAAGTCCATGTGAAGGAGATACGGGTGGCCCTATGTTTGCAGAGGAGGCAGGACATCTGTAGTGAAGCTGTCGTGGCAGAAGCAGTGTCTGGAGGAAACGAAGACCTGC

Protein: 900 aa

>Echinoid_protein

MDPASMKMEILRFVVFLISTVAVYSQDPGWIEKPINTFVNEGGNTTLKCRISNRGSRQLSWNKQNGGLLFVDGQKWTTNERYSILPHPEGYDLRITNSQREEDGEYSCSLQLSDLQESVRVTVVVIPGQPSIQPPGPLAEGETLSLTCSSIGGNPPPSVLWKKNDVTVSEGIVTSPPGDTFGVTSSVLTTVVSRLDHRSNFTCSVENDANIGSPRRVSAIIAVQYSPVISFGPYNPLTVIEEERTTLTCVIDANPLATDVRWIKDGTVIGTGVEYMFPSIQKANSGIFSCQAGNSIGTKRENVTLHVQYAPKVALRMSNSVIGSRSLTINEGQRINLTCVVDANPKISGDIEWTYNYGPVISYGPSYIIESVSRMNAGNYSCRARNTLTPSGRPSLQQTGSTTLEILVQYSPGAAEVSGPDFGLKGQSSTLVCKINDPGLPTASLQWTKFGSNEVIREGSTLTFDPVSLYDNDDYICTPRNEVGDGAPDTYRLIVNEIPEFVPTEFPATKTVQIHQRGLVLTCKARGRPEPRITWYKDNNALTELDGFYKIETTTRPVDTHSWNVTSTLYWKGPSRTPELDSLRIADAGTYSCRASSEISQDVLSQEMRLNIQYPPVINISMDKVAIDINQTAILNCQAHSVPPSNFTWYRGSQVLTPGGRMSIMEAMVPSAFPYRTTLSIQDVVMSDLASYRCEAINAIGQNSFSIQLVVRTIPDPPSNLNALARTWESVELQWQAAFDGGYSQHFIVAFESSHHSRNRVRVTPPHATIFNVTGLYPDTNYTFRIFGENELGGGDYSKSVTMTTSAFSIDPPLDVLYHKSDRTLTFKSPSSNYCGRVENFDGRQWRLYEQCANSSGSKIKVAEQVHVKEIRVALCLQRRQDICSEAVVAEAVSGGNEDL

Domains

IG_Like: 34 – 125

IGc2: 140 – 207

IG_2: 235 – 307

IG: 324 – 406

IG_3: 426 – 481

IG: 519 – 605

IG_Like: 631 - 711

FN3: 715 - 805

Clone

Partial ORF

Primers

Forward: CGAGAATGACGCAAACATTG

Reverse: AATTCGGTTGGCACAAATTC

Nucleotide# 1098 – 2000 = 903 nucleotides

>Echinoid_clone

CGAGAATGACGCAAACATTGGATCGCCCCGAAGGGTCTCCGCCATCATTGCCGTTCAGTATTCACCAGTTATCTCATTTGGACCTTACAATCCGTTGACAGTCATAGAAGAGGAGAGGACCACCTTGACCTGTGTTATAGATGCCAACCCACTCGCCACCGATGTCCGATGGATTAAAGATGGCACCGTTATTGGAACGGGAGTGGAGTACATGTTTCCTTCAATTCAAAAAGCAAATTCCGGAATTTTTTCCTGCCAAGCAGGCAATTCCATCGGGACCAAGCGGGAGAATGTGACGCTACATGTGCAATATGCCCCAAAGGTGGCGCTGCGCATGTCAAATTCTGTAATTGGGTCAAGGTCATTAACAATAAACGAAGGTCAGAGGATTAACTTAACGTGTGTCGTAGACGCCAACCCAAAAATATCTGGCGACATAGAATGGACGTATAATTACGGACCTGTGATTTCTTACGGACCCTCTTACATAATTGAGTCCGTGAGTCGGATGAATGCTGGGAATTACTCGTGTAGGGCACGGAATACGCTAACGCCCTCTGGCAGACCGTCGTTGCAACAGACGGGAAGTACGACGCTCGAGATACTTGTTCAATATTCACCCGGGGCAGCAGAAGTTTCAGGACCGGATTTCGGATTAAAAGGACAATCATCAACGTTAGTCTGCAAAATTAATGATCCTGGTCTACCAACAGCATCTTTACAGTGGACCAAATTTGGGTCAAATGAGGTCATCAGGGAGGGATCAACCTTGACCTTTGACCCAGTTTCCCTTTACGATAATGATGATTACATTTGCACTCCCAGAAATGAAGTTGGTGATGGAGCGCCCGATACATACAGGCTTATTGTTAACGAAATACCTGAATTTGTGCCAACCGAATT

Cloning info

PCR rxn on 07/28/16 using GoTaq polymerase mix

| Template | Initial denature | Denature | Anneal | Extension | #cycles | Final extension |
| --- | --- | --- | --- | --- | --- | --- |
| 2 hr cDNA | 4min @ 94C | 30sec @ 94C | 30sec @ 55C | 2min 15 sec @ 72C | 35 | 10min @ 72C |

**Afadin**

Gene model: 7563 nucleotides

ORF: 278 – 7171 = 6894 nucleotides

>Afadin_full

TGGATACCAGCTTGCTGTCCACATAACTTTTGGACTTACTAACGTTAATAAATTACGAGTTAAATTATGTTTTGGAAGCTTGTGCTTTGGTGTTCTCTGGTACACGTGGCAGACCAAATCGAGTTCTTCCTGCACTTGTAAAATTCTCCGTGGCGTACGCCATCATGCGGGAAGCTTGCTAAACAAACGATTTCAATATGTGTTTCTCGTGATATTTGGAGATTAATTTACCTTTGAGAGGTGAAAATTAATAAGGCAGACTCTGAAATTCAGCACAATGGGCTTGTCATACAAGGAAGAGGAGAGGGAGCGGCTTATTGCTCAAATCAACGAATGGAATGCGAATCGGCTGGATCTTTTCGAGTTGAGCCTGCCAAACGAGAATCTTGAGTTCTATGGGGTTATGCGATTTTTCCACCAAGATGAAGGATCCAAGGTTTCCACCAAATGCATCAGGGTCTCCAGCACGGCAGCAACACAGGATGTTCTGGCCACCTTGATCGAGAAATTTCGACCGGATATGCGTATGCTAACAAGAGGAGAATATTCTCTGTACGAAGTCCACGTCAATGGAGAGGAGAGGAAGTTAGACGGTCAGGAGAGACCACTGTTTGTGCAATTGAATTGGGGCAAAGATGACAGAGAAGGGAGGTTTTTATTGAAACGAGAAGACCTCAAGACTGTCAGCTTGAACGTTATGATGCAACAGACAAATCTAGCGCCACAGCCGACAGAAGAGCCTCAACAATTTAAGAGAAAACTCTCGAAGAGAGAAAAGAAAGAGTTGAAGAAAAAGGAGAAAGAGGCAAAGATTAAGGAACAAGAGAAAGAAAACGTTGCATCAAAACTTTACAATGAGGTCCCAGACACAGGATTTACTCGTAGTATTTCCAATCCTGAGGCTGTCATGCGGCGGAGAAGGCAGCAGAAGCTAGAGAAGAAACTGCAACAGTACAAGAGCAAGGATGGGGGACCAGACACAGGAGGTACATTGAAAATCTATGGAGAATCCCTCAATGCAGATGTGCCATACAAAACATTGCTTCTCTCCATCACTGACAGTGCAGCTTATGTGGTCAAAGAGACACTTGAGAAGTATGGCCTTGACAAGGAGGACCCCAATAACTACATGCTTGTCCAGGTTGTCGTACCCCCTGGAGGCAGTGAGTTCCACGGAGGACAGGGTTGGCCAGGACAGAGGGACATTATTCTGGATGAGAATGAGTGTCCACTGCAAATCCTTGTTCAGTTCCCTCCATCTAAAGGAACGATCATGTTCCACATCCGTCGACGGCCTGACACCATGCCAAAGAAGAAGAAAAAGAAAGGACCGAGAGGAGAGATGGAGGATCCAGGAATACCCCGATATGACGGCCCCGAGAAAATGCCGTTTCTCGTAGAAATCAAGAACGATCTGAGTGAGCTGCCCAATGGTATGCGATACACATTGCCGCTGAATGTCACAGAAGTCGGCTCAGACAAAAACATGGCAATCAGAGGCCAACACATCCAGCTGTACGGCAATGACATTAAGCCTCGCCACTGTTTGATTGCACATACTGCTGGGGTCGTGACTGTTACTCCTGCCAGCAAAGAAGCCTTGACTTTTGTTGATGGGCAAGTCATTCATGACACAACTATGTTGCAGCATGGCATGACCGTCAAGTTTGGAAACAAGCATTTTTACCGGTTCATCGACCCTGGCTTTGAAGAGAAAATGATGCAGCCTCCCTCAGTTGTCAGAGAGGGGATTCCAAGAGGAGGTCCTCCCCCATATGAGGGCAATATGGAAACGGCTAATTTCGAGACAAGTTTCAATCTTGACGGCCAGGTGGAGACTGTTGCTGGTCAAGATCCCGCAATGAGTCCTCCCTATGGAAACATTCCGCCCCAGATCGATGACATCCTGCCGGCAACACTGGAATTCAGAGAAGATGGTGAAGACGCATTCCTTGCCGCAGTCATCAGCGAAGTCAATGGAGCAGCTGTCCAATTCAAACTTGGCCCAACATACACAATTTACATGGCTACAAGGTTTCGTATCTCCATGGCGTACAGACCTAAAATGAGCCCAGAAGAAAGAGCTGATCGTCTGACTGCCCTGGTGAATAAGGTCGCCAATCTAGTTCTACAGACAATCCAGGAAAACAAAGACAATGCAGTGCAGTTGTCGTTCTGGATGGCCAATGCCTCTGAGTTGCTTCACTTCTACAAACAAGATGGAGACATCAGTCGATTCAGCCTGGACGCCCAAGACACCCTTGCCAATGCTGTGCAGATGGCTTTTCGCTACTTGGTTAACTGTATGCAGAGTGAGTTGCATCACACCATGCTGGCTTTCCTCAATCCAAGTGATTTCGACATGAACGAAGATGAATTTGGAGGTGGCTACGAGCACAACCGCCCTAATCTGATGGACGTCATCCAAGTATTATCATCCGCGATGTCTCTGCTTCGGAGGTGTCGTGTAAATGCTGCTCTCACCATCCAGTTATTCTCCCAGCTCTTCCACTTCATCAACATGTGGTTGTTCAACAGACTTGTCCTTGAGCCAGACCTTCAGTTGTGCACTCGCGCCTGGGGCGAGAGGCTGAGAAGACGTTTAGCTCGCATTGAAGCATGGGCGGAGAAACAGGGATTGGAACTAGCAGCCGATTGTCATCTGGCTAGGATCATTCAGGCTGCTCATTTATTGAAAGAACCAAAAGCATCTAGGGATGATATCACAAATATCAGTTCCGCATGCTTCAAGTTGAACTCCCTTCAGTTGAGGGCTTTGCTGCAGAACTACATTCCTGGACCAGGAGAACCTCACATCCCTCGGGATTTAATCGAGAATTTAGTCGGAGTAGCCCAGAGCACCGCTGATGAGTTGACTCGCAGTGACGGCAGAGAAGTCATGTTGGCTGAAGAGCCAGATCTGCACTTACCCTTCTTGCTACCGGAAGATGGGTATTCCTGCGACATTATCCGTTTGGTGCCCAACGGATTGGCGGAATTCCTCAAACCTCTGTCACAGACTGGTATCTGTCATATGACTGAGCAACCTTTGTCGTCAGGATCTTGGACGATCTACATGCTTATGGATGAGAGTGCACAGTCGGGACAACCCCCTAGAACTGACGGCCCTCCACAAGGCATGCCTAAAGAACCACAAGTCATCAATATCACATTCAACAAAGTCAAAGGAAGCATGGGACTTAGCATAGTCGCTGCAAAGGGAGAAGGCCAAGAAAAGAAAGGCATCTATGTCAAATCTGTTGTGACTGATGGTGCTGCTGCCCAGGATGGACGTCTTCAAACTGGTGATCAGCTGCTTGAGGTTGATGGCAAGAGCTTGATAGGTGTCACCCAAGAAAAAGCTGCAGAGCTAATGACGAGAACTGGACAAGTGGTCACACTGAAGGTGGCCAAGCAAGGAGCTATCTATCATGGTCTGGCCACACTACTTAGTCAGCCTTCTCCTGTGATGCAGAGAGCGGCCAACAACAGAAAGAGTGGCGGTGCTGGCAACAAGAGTCCTGGTCCACCAAGGTCTCGTAGCGAAGAATCCATGCCTCCTTTCATGGAAGGATCCCCTCGTTCTTCATCAGGGAAACCCCCTGGTCCAGGAGGGGATTTCTCGCCATACAACAACAAGCCTAACACTCCCAACATGCAGAGGCCCCTGCAGCAGCAGTACGGAGCCAGGTCATCTCCTGTGCTTGGTCCAGGTCCAGTCATCAGAGGACCAGAGCCTGCTGCTCCTCCTCCAGAGATGTACAAGCAGCCTCCTCCGATGGGCAGTCCAGCCATGTACAGTCCCCAGGAGCCACATCCGAGGGATAACATGCGCTCCAAATCAACATCCAACTTGGACCAGCAACCCCCTGAGAAGGGCATGGTTGGACCAGGATATCAACCCAGTCCACCAGCTGGCCAACCGCAAATCAGACACGCCTCCCAACCAGATTTGCAGCAGCCAGGTTATTCGCGTTATGATGATTACCCACCGGACAGGAACTCGTACCAACCGAATTACGTCAACCAGATGGAACTGCGCAACCAGAGCGGCTACTGGCCCAACCAAGAGCAGCAGCCGCCGTACGTCAAGACGTCTCAATCTACGCCCAACATACAGGCTGACATGTACGAAAGGAGTGAGCCTAAGCCACCCACGGAAATGGACCACTCACGAGTCCACGAATGGCAGCAGAGGAATGAGATGGAACAAGCCAAACAGAGGTGGCAGCCAGAAGGATTACACTCAAGAGAAGAAGATGACATCCCGAGAGGGCCGCAACAAGGAAGTTTCTACAACAGACCAAGAGAGAATACTCCTCCGTACGAGCAAGGCCAGCGCCCGGGCGATCCCATGGCGAAACAACCGGTTGCCCCCAAGCCAAATCTGGCACCTAAACCAGGAGTAAAACCAAAAGACATTCCTAAAATTCCAGGAGATAACAGACAGTCACAGTCCAGCTTCTACGAACAACCGAGGGGTCAGCAATATCCAGGGCAAAGAGATTTTCCAAACCAACAACCTCAAAACCCAAACACATCACAATACTTGCCAACATCACAGTTTCAACCGACATCTCCATACACTAGCAATGCTCCTTATGGCTCTGGTCCGCAGTTCATGCCACAGTCAACCATGGATGACTCTCGCATGGGTTACAATTCTCCACACGACTCTTACGGTGCTCATCAGAAGATGTTGTCACCAGCTGGTCAACCTCATCCTTACGACAGACGGCAGCAGTCTCCAGAGCTGCCTCCTCCCCCTGACATGTTACCTGACATGCCTGCTCCCCCCGTGGGAGCTGCCGCCGAAGATCTGCCTCCCCCTCCTCCTCCCCCTGCGGAGTACGAACGCTTGATGCAGGAGGAGCACTTGAGGAAGATGGGGGTTGATCCAAGTCGCATCAGAGATCGGATGGATCCCCAGGGGGCTGACTCTTTCGGTTACCCGGACAACAAACCCCAGAACGCAAGCTTTCGTGGACCTTCTCCAATGTACATGGGCCAAGACCAGAACAGGCAGGGCCCAAAACCTCCAGGTGCTCCAGGACCCCAACAACCCCAGCAACCTGGCTATCAAGGCTTTATGCCTCCAACTCAATCCAACTACCACAACTACCAGAATTTACCTCCTGTTCAGAATAGGCCCTATGACATACCCCCTGGACAAGGTCCTAATTTCGCTAACAAGCCAGATGGTGCTCCCGGCTACGGCTACAGACCTAATGACTCTATGATGCGCGAAGGTGGTCCTCCTCCAAGACCAGACTCCAAGCAAATGAACAAACCTCAAGTCAATCCCAAGCCTGATTTCAAGAAGCCAACCTCTTCCCCATGGGAACGAGAGCAGAAAGAAAAACAGGAGAAGATGAGACAGGAAGACATGAGTCGTGTCAGACTACAACAGATTGGAGATCTAGAAAACAAACCTCATTTGAGTCAAGAAGAACACGACAGGCTGAGGCGACTGCGCTTGGATCAAGAGTTTGAGAAGAGAGTGGCCGAGGCCCAGAGCAAGGAAGACGAAGATTCTGATACTGAACTGACTGACAGGCCTGCTGGTCGTGCACACATGTTGAGAATGCTCCAGGATGATTTGTCGAAGAAACGGCAGATTCAGGCAGAAGAGAAACAACAGCAGCAGATCCAGAGAGACCAGGCTGAAGCTGAGAAGAATGAGAGAGCTGAACGTCGCCTTGCCATGTTTGAGAAAGAAAGACAAGAACAGAAAGAAAGAGAAATGAAACGTCAGGAGAGAAGGGAGAAGGAACAAGAAGAGATCTTGCGGAAACAAAGGGAATTGCGAGAAAGACAGAGACAAGAGCTGGAAGAAAGTCAACGCCAGGCTCGTCTGGAAGAAGAGAGGAAGAAAGAGCTTCAGAGGGAAGAACTGCGGAAGAAAAAACTTGCTGAGCAAGAACATATGCGGGACCTGCAACTCCAGCGAGAAGCAGAAGAGCGCAGCATGAGAAAGGCCATGCAGATGAACAGACCTGATGACCGATACAATGTCATGGGATACCAACCGTATGGAGACAGGCAGGAGATCCCCAAAGGTCCAGATCACATGGCTCCCGGAGTGGATAGAATGCCTCCAGGCCCAGAAAGAATCCCTCCCGGTCCAGAAAGGATCCCTCCGGGGCCGGAGAGAATCCCTCCAGGTCCAGAGAGAATGATTCCAGGCATGGACAGGAGAGATATGCGTCCGAGTCCAGATAGACGAGACCCTTCTGGTCCCGCTGACCACGACAGAAGAGAGCAGTCTCCAGGTTTGCCGTACGCCCCAAGAGACGCTCCTCCAGGGTTGCCGTACGCTCCAAGAGAGGCCCCTTCGATGAGTATATCGTCAAGTAGCATCCACCACTACGGTCCATCAGTAGATATGCCACCCTCTAGCGCTGCTCAACCGTACAGCAGTGGAGCTCCAGTTAGTGGAAGTCACGCTCCTCCCAGGGACTCTTACATGTCAGTAAACGCCCCTTTTGCTCCACCTCCACCCCAACGAGGGTCGTCTTACGACGTAGCTAACCAGCACAGCTTCCGTGGCCCTCCTGGTCCAGGGACCCCAGAGGGTTACGGTTGGGATCCCCGACAGGGTGGCAATACCCCGTCAGCCATGCGTCATAATATGGACACCACACCTGTTCCTAAAAAGAGTGTATCCTTTCACGACAACATAGCGACCGAGATTCGGGACTCTCGCTATGGCTCGACGTCGTCAGAAGCGACAGATCGTAACTCGGGACGTTTCGGCTCGACGTCCTCCGACAACGGATTCAACCAATACCAGAACAACAACGGCAACTCGATAAGCCAATCGCCCTTCCAGAGACCCAACTACGGACCCTCGGCTGCTCACGGGAACCCTCGGCAAGAGGAGGTGTTTGAGATGCGCACTCCCGAGAACCAAAGCACCGCATCTGCCACTCTAGTCCCTGGCCCCACCCCCGGAGTAGTCGGCGCACAAGAGGTCTACCTTGATCCCAGAGAGCGTATCGCGGCCCAAAAGGCTGCTGCTGGCAACCCCGGACAAGCTAACGCAGACAGAATGTCGTTTAGAGACAAAATGAAAATGTTCGCACAAGAGGCTGGAGAGAACACGCCCAAAGAAAAGCCCAAAATCTCCCGAGCCCAGAGACAGATAGAGAATATTATAAATGGCCAGTGAGACTGAATAACCTTTTAACAACATGGTGCATTATCTTTTTTAGCATTTTAATAACATTTGTAAGATACTATTTGTTTATGAATTATATTTACATGTGTTTTATGTTTTGACCGAAGTACCACTAGCCTAGTAATTTATAATCGTAAACTAAGTGTCATAAACAAAACTATTAACTTGTAAATATGTATGTGTGGTAGGAGTTTTAGTATTCTCATTTATTTTTACTGTACATTGTACAAGCTTGGTTGGCAAATCCATATATATTATATGAATGATGTTATCGCATTTAGTTTTTACTTGATTTACTTGACAAGGCAGATGTTTTAGGTTTGTTTTTTAGATACCAAAATATCTTGACAGAGAATCTGACAAAATTTATGTGACATTTTAAA

Protein: 2297 aa

>Afadin_protein

MGLSYKEEERERLIAQINEWNANRLDLFELSLPNENLEFYGVMRFFHQDEGSKVSTKCIRVSSTAATQDVLATLIEKFRPDMRMLTRGEYSLYEVHVNGEERKLDGQERPLFVQLNWGKDDREGRFLLKREDLKTVSLNVMMQQTNLAPQPTEEPQQFKRKLSKREKKELKKKEKEAKIKEQEKENVASKLYNEVPDTGFTRSISNPEAVMRRRRQQKLEKKLQQYKSKDGGPDTGGTLKIYGESLNADVPYKTLLLSITDSAAYVVKETLEKYGLDKEDPNNYMLVQVVVPPGGSEFHGGQGWPGQRDIILDENECPLQILVQFPPSKGTIMFHIRRRPDTMPKKKKKKGPRGEMEDPGIPRYDGPEKMPFLVEIKNDLSELPNGMRYTLPLNVTEVGSDKNMAIRGQHIQLYGNDIKPRHCLIAHTAGVVTVTPASKEALTFVDGQVIHDTTMLQHGMTVKFGNKHFYRFIDPGFEEKMMQPPSVVREGIPRGGPPPYEGNMETANFETSFNLDGQVETVAGQDPAMSPPYGNIPPQIDDILPATLEFREDGEDAFLAAVISEVNGAAVQFKLGPTYTIYMATRFRISMAYRPKMSPEERADRLTALVNKVANLVLQTIQENKDNAVQLSFWMANASELLHFYKQDGDISRFSLDAQDTLANAVQMAFRYLVNCMQSELHHTMLAFLNPSDFDMNEDEFGGGYEHNRPNLMDVIQVLSSAMSLLRRCRVNAALTIQLFSQLFHFINMWLFNRLVLEPDLQLCTRAWGERLRRRLARIEAWAEKQGLELAADCHLARIIQAAHLLKEPKASRDDITNISSACFKLNSLQLRALLQNYIPGPGEPHIPRDLIENLVGVAQSTADELTRSDGREVMLAEEPDLHLPFLLPEDGYSCDIIRLVPNGLAEFLKPLSQTGICHMTEQPLSSGSWTIYMLMDESAQSGQPPRTDGPPQGMPKEPQVINITFNKVKGSMGLSIVAAKGEGQEKKGIYVKSVVTDGAAAQDGRLQTGDQLLEVDGKSLIGVTQEKAAELMTRTGQVVTLKVAKQGAIYHGLATLLSQPSPVMQRAANNRKSGGAGNKSPGPPRSRSEESMPPFMEGSPRSSSGKPPGPGGDFSPYNNKPNTPNMQRPLQQQYGARSSPVLGPGPVIRGPEPAAPPPEMYKQPPPMGSPAMYSPQEPHPRDNMRSKSTSNLDQQPPEKGMVGPGYQPSPPAGQPQIRHASQPDLQQPGYSRYDDYPPDRNSYQPNYVNQMELRNQSGYWPNQEQQPPYVKTSQSTPNIQADMYERSEPKPPTEMDHSRVHEWQQRNEMEQAKQRWQPEGLHSREEDDIPRGPQQGSFYNRPRENTPPYEQGQRPGDPMAKQPVAPKPNLAPKPGVKPKDIPKIPGDNRQSQSSFYEQPRGQQYPGQRDFPNQQPQNPNTSQYLPTSQFQPTSPYTSNAPYGSGPQFMPQSTMDDSRMGYNSPHDSYGAHQKMLSPAGQPHPYDRRQQSPELPPPPDMLPDMPAPPVGAAAEDLPPPPPPPAEYERLMQEEHLRKMGVDPSRIRDRMDPQGADSFGYPDNKPQNASFRGPSPMYMGQDQNRQGPKPPGAPGPQQPQQPGYQGFMPPTQSNYHNYQNLPPVQNRPYDIPPGQGPNFANKPDGAPGYGYRPNDSMMREGGPPPRPDSKQMNKPQVNPKPDFKKPTSSPWEREQKEKQEKMRQEDMSRVRLQQIGDLENKPHLSQEEHDRLRRLRLDQEFEKRVAEAQSKEDEDSDTELTDRPAGRAHMLRMLQDDLSKKRQIQAEEKQQQQIQRDQAEAEKNERAERRLAMFEKERQEQKEREMKRQERREKEQEEILRKQRELRERQRQELEESQRQARLEEERKKELQREELRKKKLAEQEHMRDLQLQREAEERSMRKAMQMNRPDDRYNVMGYQPYGDRQEIPKGPDHMAPGVDRMPPGPERIPPGPERIPPGPERIPPGPERMIPGMDRRDMRPSPDRRDPSGPADHDRREQSPGLPYAPRDAPPGLPYAPREAPSMSISSSSIHHYGPSVDMPPSSAAQPYSSGAPVSGSHAPPRDSYMSVNAPFAPPPPQRGSSYDVANQHSFRGPPGPGTPEGYGWDPRQGGNTPSAMRHNMDTTPVPKKSVSFHDNIATEIRDSRYGSTSSEATDRNSGRFGSTSSDNGFNQYQNNNGNSISQSPFQRPNYGPSAAHGNPRQEEVFEMRTPENQSTASATLVPGPTPGVVGAQEVYLDPRERIAAQKAAAGNPGQANADRMSFRDKMKMFAQEAGENTPKEKPKISRAQRQIENIINGQ

Domains

(2x) RA: 41 – 132, 236 – 341

FHA: 370 – 472

Myo5p-like_CBD_afadin: 553 – 885

PDZ: 960 – 1048

Clone

Partial ORF

Primers

Forward: AAATGCATCAGGGTCTCCAG

Reverse: CCCCTCTCTGACAACTGAGG

Nucleotide# 446 – 1750 = 1305 nucleotides

>Afadin_clone

AAATGCATCAGGGTCTCCAGCACGGCAGCAACACAGGATGTTCTGGCCACCTTGATCGAGAAATTTCGACCGGATATGCGTATGCTAACAAGAGGAGAATATTCTCTGTACGAAGTCCACGTCAATGGAGAGGAGAGGAAGTTAGACGGTCAGGAGAGACCACTGTTTGTGCAATTGAATTGGGGCAAAGATGACAGAGAAGGGAGGTTTTTATTGAAACGAGAAGACCTCAAGACTGTCAGCTTGAACGTTATGATGCAACAGACAAATCTAGCGCCACAGCCGACAGAAGAGCCTCAACAATTTAAGAGAAAACTCTCGAAGAGAGAAAAGAAAGAGTTGAAGAAAAAGGAGAAAGAGGCAAAGATTAAGGAACAAGAGAAAGAAAACGTTGCATCAAAACTTTACAATGAGGTCCCAGACACAGGATTTACTCGTAGTATTTCCAATCCTGAGGCTGTCATGCGGCGGAGAAGGCAGCAGAAGCTAGAGAAGAAACTGCAACAGTACAAGAGCAAGGATGGGGGACCAGACACAGGAGGTACATTGAAAATCTATGGAGAATCCCTCAATGCAGATGTGCCATACAAAACATTGCTTCTCTCCATCACTGACAGTGCAGCTTATGTGGTCAAAGAGACACTTGAGAAGTATGGCCTTGACAAGGAGGACCCCAATAACTACATGCTTGTCCAGGTTGTCGTACCCCCTGGAGGCAGTGAGTTCCACGGAGGACAGGGTTGGCCAGGACAGAGGGACATTATTCTGGATGAGAATGAGTGTCCACTGCAAATCCTTGTTCAGTTCCCTCCATCTAAAGGAACGATCATGTTCCACATCCGTCGACGGCCTGACACCATGCCAAAGAAGAAGAAAAAGAAAGGACCGAGAGGAGAGATGGAGGATCCAGGAATACCCCGATATGACGGCCCCGAGAAAATGCCGTTTCTCGTAGAAATCAAGAACGATCTGAGTGAGCTGCCCAATGGTATGCGATACACATTGCCGCTGAATGTCACAGAAGTCGGCTCAGACAAAAACATGGCAATCAGAGGCCAACACATCCAGCTGTACGGCAATGACATTAAGCCTCGCCACTGTTTGATTGCACATACTGCTGGGGTCGTGACTGTTACTCCTGCCAGCAAAGAAGCCTTGACTTTTGTTGATGGGCAAGTCATTCATGACACAACTATGTTGCAGCATGGCATGACCGTCAAGTTTGGAAACAAGCATTTTTACCGGTTCATCGACCCTGGCTTTGAAGAGAAAATGATGCAGCCTCCCTCAGTTGTCAGAGAGGGG

Cloning info

PCR rxn on 07/01/15 using GoTaq polymerase mix

| Template | Initial denature | Denature | Anneal | Extension | #cycles | Final extension |
| --- | --- | --- | --- | --- | --- | --- |
| 2 hr cDNA | 4min @ 94C | 30sec @ 94C | 30sec @ 55C | 2min 15 sec @ 72C | 35 | 10min @ 72C |

Purified PCR Product with Thermo Scientific kit on 12/01/15, Second PCR from purified template on 12/01/15

Ligated PCR product into Pgem T Easy vector and transformed into competent cells on 12/01/15

Picked colonies for overnight cultures on 12/07/15

Isolated plasmids using Promega miniprep kit on 12/08/15

Confirmed clone Afadin by sequencing on 01/15/16

**Van Gogh Like**

Gene model: 4680 nucleotides

ORF: 499 – 2166 = 1668 nucleotides

>VangL_full

ATTAGACACACTACTAGTCACTAGCTATCTATACCAATGTGACCATGACTTCTAGGAGTGCAAAGCCACCAAGGAGGGAATTTGATACTTTTCAAAACACATGTTACCCCTATTTAGCTCTGGGGGTTCTTCGATGATTCTTACCTGTGCATTTTAATGCAGTTTAGAAGTTTTTGAAAGTCACCCGAGCGTGACCTGTTAACTCCAGCTCTGAAGATTTCTAAGTAGGTCACGCAGTGCATTGTGGGAGCTGAAAGGAAGCCAAGCCAGAAGCTCCAAATATCGTTCGAAACTTGGAATTGTCAAATTGGAATGCATTCAGGAATGTGCAGAAATTACGGATTATACTCCCAACAGGTGATGAGAATGTGAAGGAATGGTGAAGACAAGATTGAAAAACAGCTGACCAACCATATATTACCTTGAATCTTCGGATTAACCGCCTACAAAATGAGCATGGTTCTGCCGACGGTGGCATAGCAACCAATACCCACCAAGATGGACACTGAGTCCGTACGTTCGGGGCGCAGTGAGCGCTCGGAACGCTCTCAGAGGAGTAACCGTCCTTATCGCAACAAATCGGGGCGCCACTCGAGCAGAGAGAGGAGCCACGACCGCCATAGGGAGCGTCACCATGGCGACAACCATAGAAACGGCGGCGGCCACGACGACACAGACCGCGACGACCGCTCGGTGACGATAGCCCCGTTGCCCCGGTCGCACCATCACCACAGCGATGTGACTGTGACCCAAGGGCGGAACGGGGAGGAGCGCATAGAGGTTCAAATCATGCCTCAAGATGAGAACTGGGGCGAAACGACGACTGCCATCACGGGCAACACGAGTGAGACAGGTTTCTCGATGGAGGACATGTCTCGTATCAACAAGGAGATGGAGGAGGGAATAGGATTTAACTGTGAACGATATATGGGCTCTATAGTGGCTGGGATGTTAGGAGTCCTGGCCTTTCTATCTCCTATAGCAATGGTGATTTTGCCCAAATTAGGCTTAGAGGAATGGGAGCAGGATCCCTGTGGACCTGAGTGTGAAGGATTATTAATAAGCTTTGGCTCTAAACTGTTTATTCTTTTAATCGGCACGTGGGCGTTATTTTTCCGTCGCCCCAAGGCCACCATGCCTCGGATTTTTATCTTCAGGGCAGTGATTTTATTTCTAGTGTTTATTTTTACCTTTGCTTTTTGGCTCTTCTACGGATTCCGCATTTACAAAGCCAAGGAGAAGAGTTACCACAATATAGTGCTGTACGCCGTGTCCCTTGTGGACACGCTTCTGTTCATCCATTACATCGCCATCATTCTTCTGGAGGTGCGGCAGTTGCAGCCTCAGTACGCTGTTCGCATCACCCGCTCACCAGATGGCGAGTCGCACAACTACACGGCGGGACAGTTGAGCATCCAGCGCTTGGCCGTCTGGTGCTTAGAACAATACTACCGTGATTTCCAGGTTTATAACCCTTACTTGGAGCACGTTTCTCGGCGGGTGCCCAAACTCTCGGGATTTAAAGTTTACGACATAGATGGAGTGCAAGGATCTCAAACGCCGGCTACTCGGTCGCGGGCGATATTCGCGGCTGCTGCTAGGCGGCGTGACCAGTCGCACAACGACAGGTTCTACGAAGAACAGGATTACGAGAGGAGGGTTCGCAAGAGGAAGGCGAGATTGTTCACGGCAGCGGAGGAGGCGTTCACCCACATCAAGAGGATGCAGGAGGAGTCAGGCCCAGCCATCCCCATGGACCCCTTGGAAGCGGCCCAAGCGATCTTCCCTTCAATGGCCCGAGCTCTTCAAAAATACCTGAGGATCACACGCCAGCAGCCGAGGTACACCATGGAGAACATCCTGCAACACTTGGCCACCTGCATCTCGTGCGACCAGACGCCAAGGGCCTTCTTGGAACGATACCTCACTCAGGGACCTGTGATCTGGAACGACAAGGACCTGCGCTCCACTCAGACGTGGGTCTTGATCTGCGACCAGCTGCTCAGCAGGGCTGTCAAGGACGGCACTGTGTTCCAACTGCGCCAAGGGGACGTCACCCTGCTCATTGCTGTCCGCCATTTACCCCACTTCAACGTCACTGAGGAGGTTATCCACCCCAAAAATAACAAATTTGTACTTCGCCTCAACTCCGAGACGTCTGTATGAGTGAAAAGTTGCTGAAAATGTAACATAGACAATTGATATTGGCTATTACCTTGGTGATTATATTGAAAATACGGACATTATATAGCAAGTACAGACTTATGCTTATAGCAAGCACGTACACCGTATGGATCTTCTGAAATGGAGCTATAACAAATGAAAATACGGACTTCAGATTAAGTCTGGCTTAACATGAACTTGCATTTTTGTCGTAAACCAATCAAAATATCTCGCAGCTTCGTGTGCCTGCTGAACCAATGCTGATCATGAAACGAATAATCTGCTAATTAATGAATAATGATTCAAATTGTTGTAATTTGTAGCAATGACCAATCATGAATATTTATTCAATCGCTTGTATCAAGTCACTTGGACATCAGGAAGATCTTGGAACAATAATTTGTCAATCAACAATCTTGAACACTATGAACAGACTTAATATATATTGCTATTCAACAGTGTCCGACCGTCTGACGCTATTAACGATATCAGGAAACTTAATGCTACGTCATCAGGGAGGTGTCTTCACAAATTGTGTGTTCAAATCACTCTTCAATCAGTAGATATCCCACTAGATGATAGACGTCCTAAAACTCCACCAATTTTACATTTGTGTCCTTAAGTATTTGGTATTATACCACAGTATCATTTATTGAACTCTATATCTTTGAAAAGCTCTCTTGTTATTTTTACAAAGAAAGGCGGGTACGAGTTACTATTGTGTTCATTATCAATTTGAGTTACATATAAATTTCTTGCAAATCAACAAGACAATGAGTTGATTCCTATAACTTGTAGTGCCAGTACCACGCTCATAGTAGGATATTTGTGAAGAGATATTTTAGTTAGTTTTCAGTTTAATTACAACGCCCACCCCGTATTATGAATTTATGATAGTGTTCTTTTGGCTCACAATATTTGTATTATATTCAGTGCTTTGCAAAAAGGTGGAAAAGCACCGTTTTAGTCGGTAATGCTGTAGCCATGGTTACTCCTATTATTTATGTGTTGCCTAGCAACATTAATGGGTCACAATAATGTAAACCAAAAATGACAGTGTGGTCTGACCGTGACTTATGGTTAATAAGATTATGTAATATATAATTATGCGCTGAAATTTACTGCCGTTATATCTGTCTGCCAATGAGTTATGTTCAATGAGAAGTGTAATTAAACTGTAATTATTATGCTGTAATATTTTGTAATTATTATGTAATTACTATGTAATTAGTACATTTAACTAATTCTTGTAAATAATTCTCGGATATTTTTGCGCCATTAGTTGCTCAGGAAGAGAGATGAGCTCACGATTGGTTTTTGTAAGACTTGAAAAAAAAAACTGGAATCTAATTTCAGTTATTTTATACAATTTTGTACAAGCATTTTTGTAGCGTTTTTGCCTAGTGTATCATTTGTACATTTATACGTCTATTTATGTCTCGTTTGCTTGCTTCCGTATCAAGTATCGTAGATCGCTCGTAAATAATAATTTCTTCTTTTGTAGTGTCAATTTTTAGGAGGATGAATCGAACTTTTTTTCTATTGCTGATTTTTTTAATAAACTTTTATTTCAAATGTGATTCGCACTGTATTGTTTATCTTTCTTTAAGTTTAGTTTTTTTTGGATTTAGCAAAAAAAAGAACTTTATGTTGAAATGGAACCTATCTTCACAGAAAAATATGCTCGAGGGTATAAGATGTTAAATGATTGAATTATGAATGTTGCTCTTTGGAGTTGAATAGAATATTTTAGATAATATTTCATCAAATTTCTAATTAAGACTCGTGAAAACCTTTAAATTATGCTGAAAAAGTATGGAATAGATTGATTTAGTGAATCGTAACGTAGGCCTGAAAGGCTGCCTGCCTGTAAAGAATACAATAAATGAAGAATGTAAACTGCATAATTATACTGAAGTACTGTAAGGTATAGGTCTGGAAGGCTGCTGGTCTCTGTAAGGTACTACAACGGTACAGAAGTTTATACTGAAGTAATGTAAGGTACTATAACGGTACAGAAGTTTATCCTGAAGTACTGCAAGGTACTAAAACAGTACAGAATTTTACTGGCTCTAATTATTATAAAACATGTGATTTCAGTTCATCTGTTATGAGGTGTGATTGTAAATGGATGATGTCACTTCTGATATTAGTCACATGTCAGTGTACATTAAACTGTCCTTTCTGTACAGTCCAAGATGTCCAGTGTTAACTTGTTGATGTAAACTGCCAAAAAGTAAATGATATGCCTGTGTAATTGCATGTTACCATGACGATGCATTATGGAATCTGTGGATCTTGTAGTTTGTCCTGTGTGACTGCAACCAAAATGAACCATTTCCGTGGTAAGGAAAAGTCAAATACAGCTCAGGAGATCTTTGTGTGAAAGATCGAGTTGGTAGATTTAATAGATTAAATGTAATATATAAAAACTCTTATGTATGTAACAGTCAAGTATTGATCCATTGTTTATTGAAGGCAGTAGCCAGGATTAGTGAAATTGTTTCCATATCTGAT

Protein: 555 aa

>VangL_protein

MDTESVRSGRSERSERSQRSNRPYRNKSGRHSSRERSHDRHRERHHGDNHRNGGGHDDTDRDDRSVTIAPLPRSHHHHSDVTVTQGRNGEERIEVQIMPQDENWGETTTAITGNTSETGFSMEDMSRINKEMEEGIGFNCERYMGSIVAGMLGVLAFLSPIAMVILPKLGLEEWEQDPCGPECEGLLISFGSKLFILLIGTWALFFRRPKATMPRIFIFRAVILFLVFIFTFAFWLFYGFRIYKAKEKSYHNIVLYAVSLVDTLLFIHYIAIILLEVRQLQPQYAVRITRSPDGESHNYTAGQLSIQRLAVWCLEQYYRDFQVYNPYLEHVSRRVPKLSGFKVYDIDGVQGSQTPATRSRAIFAAAARRRDQSHNDRFYEEQDYERRVRKRKARLFTAAEEAFTHIKRMQEESGPAIPMDPLEAAQAIFPSMARALQKYLRITRQQPRYTMENILQHLATCISCDQTPRAFLERYLTQGPVIWNDKDLRSTQTWVLICDQLLSRAVKDGTVFQLRQGDVTLLIAVRHLPHFNVTEEVIHPKNNKFVLRLNSETSV

Domains

Strabismus: 35 - 555

Clone

Partial ORF

Primers

Forward: CGATGTGACTGTGACCCAAG

Reverse: CGTAGAACCTGTCGTTGTGC

Nucleotide# 735 – 1636 = 902 nucleotides

>VangL_clone

CGATGTGACTGTGACCCAAGGGCGGAACGGGGAGGAGCGCATAGAGGTTCAAATCATGCCTCAAGATGAGAACTGGGGCGAAACGACGACTGCCATCACGGGCAACACGAGTGAGACAGGTTTCTCGATGGAGGACATGTCTCGTATCAACAAGGAGATGGAGGAGGGAATAGGATTTAACTGTGAACGATATATGGGCTCTATAGTGGCTGGGATGTTAGGAGTCCTGGCCTTTCTATCTCCTATAGCAATGGTGATTTTGCCCAAATTAGGCTTAGAGGAATGGGAGCAGGATCCCTGTGGACCTGAGTGTGAAGGATTATTAATAAGCTTTGGCTCTAAACTGTTTATTCTTTTAATCGGCACGTGGGCGTTATTTTTCCGTCGCCCCAAGGCCACCATGCCTCGGATTTTTATCTTCAGGGCAGTGATTTTATTTCTAGTGTTTATTTTTACCTTTGCTTTTTGGCTCTTCTACGGATTCCGCATTTACAAAGCCAAGGAGAAGAGTTACCACAATATAGTGCTGTACGCCGTGTCCCTTGTGGACACGCTTCTGTTCATCCATTACATCGCCATCATTCTTCTGGAGGTGCGGCAGTTGCAGCCTCAGTACGCTGTTCGCATCACCCGCTCACCAGATGGCGAGTCGCACAACTACACGGCGGGACAGTTGAGCATCCAGCGCTTGGCCGTCTGGTGCTTAGAACAATACTACCGTGATTTCCAGGTTTATAACCCTTACTTGGAGCACGTTTCTCGGCGGGTGCCCAAACTCTCGGGATTTAAAGTTTACGACATAGATGGAGTGCAAGGATCTCAAACGCCGGCTACTCGGTCGCGGGCGATATTCGCGGCTGCTGCTAGGCGGCGTGACCAGTCGCACAACGACAGGTTCTACG

Cloning info

PCR rxn on 07/28/16 using GoTaq polymerase mix

| Template | Initial denature | Denature | Anneal | Extension | #cycles | Final extension |
| --- | --- | --- | --- | --- | --- | --- |
| 2 hr cDNA | 4min @ 94C | 30sec @ 94C | 30sec @ 55C | 2min 15 sec @ 72C | 35 | 10min @ 72C |

**Fat**

Gene model: 16325 nucleotides

ORF: 486 – 15485 = 15000 nucleotides

>Fat_full

CAAGGCAGGAGACGGAGCAGGGAAAGACAGGCAGCAGTGGAGGAACAACAGAGCTCCAGCAACATCCGAATTCCTTCAAAACAGCTCCATGTTGTTTCTTTAAATGAAGTACACACGTCTAGAAGGATATTACGCATAGCTGCAACGAGTTTGGATGAGCTGTCTTGCAGGAGAGATCGTTTAAACTGCAAATTAGACGGGATTACCTGAGTTTGGCAGCGCATGGCTAGAGCTCTGGCACAACGTCACAGCTCTGCTGAGTAGCTGAACTCACTTCACTGCCACCTGAGTCTGTAGAAGGAAGAGCAAGCAGTGGAGCAGACGAGAGTTAGTATGCAGGAAGCACACGGTTTCTTGGTGTGTCACAAGGCATGACACCATGAGGAGTTAATAATTCCACTGTGGATTCGGAGGAGAAGTGTTTATCAAGGAGCAAGGGAGGGATTCGGCTCCGGGAGGACGGCCGTCTCACCTGCCGGCTCGTCATGGCGCGCCGTGACCGGTGGATATGCAGAAAAGCCCTGAATTGGACTATGTTTCGTGGGCTCTTGAGCCATGTGTTAATACTGCAGTTTTTGTTGACGTTTGCCCAAGGACAGGGCCATGGAGAAGCTGGGGTACCCCCAACTGACGAGAGAGTATCTTTTACTGTCCAAGAGGAGTTACCTGCGGGGACCGAGGTTGGCACAATATCCACCCGGGCTGGACTGACCTACCGATTTGCAGAGGACCCAGCCCTGTTCCACCTGGACCCCCGGTCTGGTAGAATTACAACAACTACAAAAATAGACAGAGAGGCTATTCGTTCTGATAATTTCGACCTTTTTATACAAAGTTTGCCTTCTGCTAGACATCTCATAGAGGTGCGCATCACAGTATTGGACATCAATGATAATTCTCCACAATTTGGCTCAAACACCATCCAAATTTCATTTTCTGAAAATGACAAACCTGGCACTCAAGTCATTCTTGACACTGCCACAGATAAAGATACTGGAATATTTGGTGTGACAACTGATTATGTGATAGTTTCTGGGAATGAGGAGGGCAAATTTCGACTAGTGCCCCTTTTGGATACCTCTAAACCTCTGTTGTACTTGGAGAATATGGTTGATCTGGACAGGGAAGAGAAGGATTTTTACACTCTACAAATCTCTGTGCCTGATGGTGGATCCCCTCCCAGATATGGATACCTAACAGTGAACGTGACAGTCTTGGATGTGAATGACAATGGACCTCTCTTTGACCAGAGCGACTACTGGGTCTCTGTCAATGAGACCATACCTGTGCGCTCATCTATTATACAGATACGAGCCACAGACCAAGACATGGGAGCCAATGGAGACATCACATACTTCATAGTAACTGACGACTACAACCAATTCAGCGTTGATGCCAAGACCGGAGTCCTGAGGAACAGTGACAAGCTGAAATGTCCCACGACTTGCAGTCCTGGGGAAGTTGCCAGCAACAGCTGCAAGCCAAGGACTTGCTTGATCATTGTAGAGGCAAGAGATGGAGGGAGGCCCATACCAATGAATGGGAGGGCTTACATCCATGTGAGTCTCATAGATGAGAATGATCATGATCCAACTATAACGGTCCAGTACACCCCGAGTGGTTCCACCTATGCCATGGTGGATGAAGGAGCGAAAGAAAGCATCATAGCGGTGGTCAGTGTGACAGATGATGATGATGGAGTAAATGGCCAAACGGATCTCCAGATTACAAGAGGCAACGAGAGAGGTCACTTCAGGATGGTTTCGATCACGTTCGCCAATGTCATCAGAGTCGTCGGGAAGTTGGATCGAGAGAGGGTCAGCAAATACAACTTGACAGTGGAGGCAAGAGACAGGGGCTTCCCCCAAAGGAGTTCCACAGCTTACGTCATAATAGTGGTTAATGATGTCAATGACCATGAGCCAGTCTTCCAGATGAAGAGCTACTCCACGAGATTGAGCGAACTGGTTCCGATCGGAAGCTTCGTCGCGAGTGTCACAGCAACGGATAACGACACTGGTATCAATGCCCTCATCACGTACAGCATCACAGCCGGCAATAACTTGGGATGGTTCACCATCAACAATGCCACAGGGCTGGTGACCACCAAAGCCCAGTTGGACCACGAACAATTGTCCACTGTGGTGCTGACAATCAGGGCTCAAGATGGAGCTACAGAGCCAACCCAGACATCCACCAACCTGACCATCAGCATCTGGGATGAGAATGATGAGGTCCCTAACTTTTCTGAGGACACCTACAGAGTGACCTTGATGGAGGGCCTTGGAGCAGGTAGAGAGGTCATCACAGTCAAAGCAGAGGACAATGACCAGGGATTGAATGGCTCTGTCACATACGCCCTTGACCCCGATGTGGATCTCATGTACCCCAATATGTTCAGTATTGACACAGTATCCGGTCGGATCACCACAAGGAGTATTTTAGATAGGGAAGTGCAGTCTACTTACAATCTTGTAGTCCGAGCTCAGGATGCTGGCAACCCACCTCAATCATCCACTGCCACTGTCATCCTAAATGTAACTGACATCAATGATAATGCCCCTGTCTTCTACCCCAAGAACTATTATGCCAGAGTGTTGGAGAATGAACCGATCCACACTTCAGTGGTACAAGTCCAAGCCAGTGATCCCGATTTTGGTGATAATGGAACTGTGTTTTACTCAATTCAAAGTGGAGCTGAACGACTCTTCGCCATCCATATTACTAATGGCTGGATCAGTACCATCGGAGATCTAGACAGAGAGAAGAAGGCTTCGTACAGAATCACAATTAGTGCGGAAGACAAGGGTGGACTAAGAGCTCTGGAGAATGCTATTGTGGAAATCGGTGTGTCTGATGTTCAGGATAGCCCTCCAGAATTCCCTTCTTCTGGATACCGATTCAACATCTTGGAAGATGACAGCAGGCGAAACGCAAGAACTGGCCGAAGAGTTGGACAAGTGAAAGCTACCTCTGCGGATACCTTGGGTACTATCACTTATGCCATCTCAGGTGGAGACAAGGGAGGTCATTTCAACATCAATGAAAACTCTGGAGTCATCTCCACTTCGAAAGGAATTGACAGAGAATTACAGGAAGTCTTCAGTGTCCAGATTGTTGCTAAAGCAGGAGGAATGTATGGCCATACTACAGTGAATATATCAGTGATGGACGTGAATGATAATGTGCCTACATTCAATCATGATCAACTTGAAGGATATGTTGTGGAAAACTGGCCTGTTGGGCATGAAGTCTTCTTAGCTAGTGCAATGGATGCTGACAATGGTATCAACTCTTCTCTGACTTACAGCATGCTTCCTGGATCAAGTGGGGTTTTCGCAGTCAACAGAACCACTGGTATGATCTACTTAGCGAAATCTCTCAGCCAGTCTACCCAGAATAGTTACACCGTCCAAATAGAAGCCAGAGATGCTGGTTCGCCAAGTTTGAGTAGTTCTACATCAGTCTCAATCATCTTGAGGGATGTGAATGACCACACTCCAGATTTCGAGCAGACCACTTACGAAGTGTCCGTGTTGGAGTCGAGGCCTGTTAACGAGAGATTCTTCTCTGTCTCAGCAACTGACGCAGATGCTGGCCTCAATGGGGAAGTCGTTTACTCTATCAAGGATGGCAACACCAGAGGGAGCTTCGGTATCTTTCCAGACGGGGTTCTGTACATCGCTTCTGAACTGGACAGAGAGACAACCGATCTGTATTTGCTAACTGTCATTGCTAAGGACAAAGGGGTTGAGGCTAGGTCGTCTTCAGCTAATGTCACAGTCCATGTCTTGGATGACAATGACAACAGACCGGCCTTCCTCAATGACACTTACACAATGTCCGTCAAGGAAGGGAGCCCGGTGCAAACATTTGTGGGGCTGGTGTCTGCAAAAGACCTTGACCTTGGTCGCAATGCCGAGGTCACCTACAGCATTGAGGGAAAGGACATCCGATTCCAGGTCCATCCCAAGTCTGGCGCCATCACGACGAACAGAGTCTTCGACCGGGAAGGCCAAGTTGAGGACATGGGCAGTGATGTCATCAGCATTGTTGTTATAGCAAGTGACTCCGGCTTGACCAAACAGGAAGACAGAGCAGTGGTCAATATTGATATCTTAGATGAGAATGACAATGATCCAGTCTTCGCTAGGAATTACTATGAACCGTCTCTTTATGAAGATGCAGAGATCAACACCCAGGTTGTCAGAGTTGGAGCAACTGATGTTGATAAAGGGGACAATGCCAAGCTGACTTATGAAATTGTCAGTGGAAATGAAGCTGGGAGATTTAGCATCAATGAGGTCACAGGACAGATCACTCTCACTAGCAGATTAGATAGAGAAGTTGTGTCTGAATACCAATTGACTGTCTTAGCTAAGGACAATGGCAAACCTTCAAGAAACAGCACCACTCAAGTCAAAGTGAAGATCCTGGATAACAATGACAACATGCCTAGATTCAGTGAGACTCAGCTGACTGTAGATGTGGAGGAGACTTCTCAACCAGGAGCTTACATCACTCAGGTCCATGCCACAGATATGGACATTGGGGTCAATTCAGAGATCTCTTACAGCATCAGTGCAGGAGATCCTGGAGTCAAATTCCGCATCGATGGAAGCACCGGAAAGATCTACGTTGCCGATTACTTGGATTATGAGAGCCAACGTCAGTATGTGCTCAATGTGTCTGCGAAAGATATGGGAAGTCCTTCTTTATCATCAGGAATGACCTGTAAGATCAATGTCTTGGATTCGAATGATAATGCTCCATCTTTCACTACCGCTTCTTTGGTGAGACAGATCGAAGAAGACACTCGTATTGGTACTTCAGTCATAACAGTCAGAGCGACCGATCCAGATTCAGGTCCCAACGGCCAACTGACTTACAGTATCATCCGACAAGAGCCAAGAGGCAACCACTTTGTGATAGACCCGGATTCCGGATTAGTGAGGACTGCCTCTGCCATCGATAGGGAACAAGTATCCTCCTTCAAGCTGACCATCCAAGCAACTGATCAAGCTCTGCAGGTGACAAGCAGAAAGTCAGCTCTGAAGCAAGTGACTATCATTGTTGTGGATGTCAATGACAACAGTCCTCGGTTCGTATCCATGGATGCTGTTGTCTTGAAGCAATCTACTTCCGCTGGATCTGTGGTTGCTCGGGTGGAAGCTGTAGATCCGGATGAGGGACCTAATGGCCAAGTTGAATATGACATCTCTCAAGGAGACACTACATTATTTTCCATTGATACCAACACTGGTGATTTGACCATCAACAGCGATACCAATGCCCTTTCGAGGGGACCTGTCCTGTTGACAATCAGAGCCAGAGACAAGGGCACTGCGGATCGCTTGGGACCTCAATCCTCCAGCTTCCGGATGACTGTGTTCGTTGAATCCGGACAACCGGGACCTTCTTTCGTGGAGACCTCATACCGGGGTCAGTTGTACGAGAACGAGGCTTCGGGGACGAGTATTGTGACATTGCAGGCGGCTTATGCTGACGCTCGCAGTGGCAACGTCCAATACTACGTCACGAACGTCACCGGAGAGGGACGAGGTCAATGGAGATACTTCCAGGTCAATCCGACGTCCGGAGTCCTCAGCAGCCAAGGAGTTCTGGATAGGGAACGAGGAGTCCAGGAATTCACCGTGGATGTTTACGCTGTGGACACTTCGTCTCCTGCCATCAGAACTACTGCCACCAAGGTTCTTGTGACCCTTCTGGATGAGAACGACAGTCCTCCCCTCTTCACCCCGGACACTTACGACAATGTGCGAGTCTCGGAAGATGCGGAACGAGGATACGTCATCATGTCGGTCGTGGCTTCGGATGCTGACCAACAAGGATTGCTACAGTATTCCATCGTCAGCGGAGATGATGATTCGCAGTTCGCTATCAACCCAACTTCTGGTGAACTCCGGGTCGTCCAGCCATTGGACAGAGAAGTCCACTCCACCTACAACTTGATCGTCCAGGCTACGGACGGAAAACAAACTTCCGTCGCAAGTGTCCACCTCCACGTTAGTGACGTCAACGACAACGCTCCCCACTTTGACACTCCCTATTACGTTTTCGAAGTGTCAGAAACAGCGAGACAAGGAACGACAGTCGGCTTAGTGTCAGCTACTGATGAAGATGAAGAGTTCAACGCCCAAGTCACTTACGAATTGCAATCGCAGTGGGCCAAAGAAAGATTCAATCTGCATCCCAACACAGGCGTCATCACTCTGCAATCTGCAATGGACTATGAAGAGAGGCCATTTTACCTCCTGACAGTGATGGCGAAGGACAACGCTGTCGACTCGATGTCCAGTTCCGTGACGGTGTATGTGAATGTGGAAGATGTCAATGATAATGCTCCTGTGTTCGACCCAAGTTCTTACAACAATGAAATCTGGGAGAACGTCACCATAGGATCAAGGATTCTCACAGTGACTGCTACAGATCCTGACTCAGGAGTGAATGGTCAAGTCCGTTATCGTCTCGGCAGTGGTGGAGCAGAAGTCTGGGGACCGTTCAAAGTTGACGCTGTAACAGGAGAATTGACCACTGCCGGGAAATTGGACAGGGAAAAACAGGGATACTACAATGTTCCTGTGGTTGCTTATGATATGGCATCCAACGTTATGGAGTCGCTGTCAAGCACTGCTATGGTCACTGTCATCTTGAAGGATGTCAACGACAATGCTCCGATGTTTGTCTCAAACAAGGAAGTGGTTGTCATGGAGAATCTTCCCTCTGGAATGTCAGTCTTCCAAGTGGAAGCTGAAGATGCTGATGAGGGAAGGAACAGTTACATCGAATACAGTCTGCGAAGGTCTGACCAGAGTCTGTTCGAGGTCAACGTAGTCGATGGCACTGTCAAGGTCAAACAGTCTCTTGACAGGGAAAGAAGGGATTTGTACCAAATTCAGGTCACTGCTATGGATAAAGGAGTACCCCCTCAGTCTTCCACCATGGATTTAACCATCAAAGTTGGAGATGACAATGATAATTCACCTGTTTTCTCACCTCGATCGTACAGGAAGACCGTGTCAGAGGATCTCAAGGTGGGAAGTATACTGATGACCTTGACAGCGACTGATAAAGACACCGGTCTCAATGCAGATTTGCGATACATCATAACATCCGGAGATGACAATCAGGATTTGTGGCTGGACTCTCACACTGGTCAGCTATACATCCAGAAACGTTTGGATTACGAACGAAAGAGGACTTACAACGTATCCGTCCTCGTGGAAGATCTCGGCGACCCTCCGAAATCTGATAAAGCAGTAGTCACGATTACTGTGACTGACGTGAACGACAATGCTCCAGTCTTCATAGATTCTCCGTATGTGGCTTACGTGAGAGAGAATCTGGATACCTTCCCAGTGCATGTCGCTCAGGTGTCCGCCAGGGATGAGGACTCTGCCCCTAACTCTGTCATCTCGTACAGCATGTTGACTGGAGATCGGTCGCTTTTCAAGGTCAATTCGAGTACTGGAGAGATCGAAGCTTTACGGACACTGAACAGAGAAGAGAGGGAGGAGTACAGCATCACTATCCGGGCTATGGATTCAGGATCGCCCAGACTTTCCAGCACAGGGACAGTGCTAATCTTGGTGGAGGATGTGAATGACAACACCCCAGTGTTTCAACATCAGATGTATCAAGCCATCATCAGTGAAAATGCCCCTCCCAGCTCCCCAGTCATCACTGTGCAGGCCTCTGATGAGGATGCTGGCATCAACGCTCACCTCAGGTACTCTTTGAGCGATGATGGCAATTCGTATTTCACAGTCGTCCCAGAAACTGGAGTGATCAAAACTGCGACCAACTTGGATCGCGAAGTCCGGGACAAGTACACTCTTGTAGTAGTAGTTGCTGATGGAGGTCTGATGGCCAGAAGTGCTTCCGCTATAGTTGTAGTAGACGTCGAAGATGTGAATGATATGAAGCCCGAATTCACTCAGAGGACTTACACCACGTACATTCGTGATCCTACTAGTGCTGGTGTCTTTGTCCTCGGAGTAACAGCTGTCGATCCAGACCTCGGCATGAACGGTGAAGTGACTTACTCCTTGTTTGGCAGTCAGGACGTCAATAACTTCAATATCAATCCAGATACTGGAATCATTACTGCCAAGAACAACATGATTGGTCAGCGATCTTACCGCTTTATGGTGCGAGGAACTGATAAGGGCCAAAATCCACTCTCAGATGAAGCAACAGTGGAGGTCAACGTCCTGCCTTCAACAACAGTCGGCTTTCCCAATCTCCAAACGCCAGAAACGGATTTCAGTCTCTCGGAAGGAACTGCCACAGGAACAACTTTAGTCACAGTGACAGGAACGTCAAGTAGACCCTCCGTGTCTTTGACTTACTTCATTGCTGGTGGAAACATGGGACAAGCCTTCGAGGTGACTTCTGCTGGAGTTGTGAGACTGAGAAATCAACTTGATTATGAGAAGACTCATCAATATGAGTTGTGGATTGGGGTAAGGGATGATGGCAGCCCTTCATTGACTCATTACATCAAATTGATCGTCAATGTTGAGGATGAGAATGACAACAGTCCTCGCTTCAGGACCACATACTTCAGTGCCACTGCTACTGAGGAGGAGAATCCTCCAACAATCGTTACCACGGTAACAGCTACTGATGCAGACTCTGGTAGTAACGGAAGAATAAAGTACACCATCGCTGGAGGAGACAACAACAATGACTTCGCCATTGACGCTAATACTGGGGTCATTCGAACCAACCAGAAGTTGAACAGGGAGTACCACAGCCAGTATACTCTGGAAGTCACAGCCACTGATCATGGTAGCCCAGCTCGTACTTCGAACGCCATAGTAGACATTGCCATCCTGGACAAGAATGATGAGCCCCCAGTGTTCACTCAGCACTTTCATACTCAGATCCCTGAGAATACCCCTCCAGGATCTTTCGTGATGAAAATCACTTCTACAGACGCTGACGATGGCAATAATGCCCAACATCAGTACAGTTTCACAGAGAACCCCGGACAGAGGTTTGCCATCAATCGAAACACCGGAGACGTGTCCGTAGCCGGTCCTCTGGATTACGAGAGCAAGAACGAATACATCTTGAAAGTGTCTGCAAATGATCGCGCTTACAGCGTTGAGACTACTGTGTCCATCTACATTCTGGATGTGAATGATAACGCCCCAAGCTTCCTATCCAGCCAGTACATGTTCATGGTCGTGGAGAAGCAGCCCCAGGGTACTTCCGTTGGTACTGTCTCCGCGATGGATCGTGATTCCAACGGAGTCTTCTCTCAAGTATTCTACTTGATGAAAACCCCCAGCAAGCACTTCCAGCTTGATTTTGACACTGGAGAAATCAAAACGAGGCAACAGCTAACCCACAAGTCCCTCCCATCCGGTGATAGCCCCGAGAATAAACACGTTCTGACAATCGCAGCTATGGATGGAGGGGATCCACCGAGATCATCTGAAGTGCAAGTTACCATCAAGATTGTCTCTGCCAACCAGAAGGCTCCAGTGTTCATAATGGAATCCAACTCATCGGCTGTTTCTATTGATGCAACGATAGGACAGACTGTGTTTACTGTCAGTGCAAGTGATGACTCCAATGGCGGCAAAGTAACCTACAGCATCATAGGAGGAAATGACACACGTTTCTTCCAACTGAACGAAGACACAGGAGTGATTTCTCTGAGGAATATTGTCAGAGGTCAACAAGGCAGGATGTACCAAGTGCTAGTCAGAGCTACGGACGAAGGAGTTCCCCAATTATCGAGTACTGTGGCAGTCTCCATCTACATCGAGGAGATCAATTACAATTCTCCAAGATTCCTCGATAACCAGGCGTTCCAGGTTACCATTGCTGAGAACCGCGAAGTTGGTGATGTCATTGGAAGTGTTGCTGCAACAGACTCAGATGCTGGAAACAATGGCATGGTGTCATACTTCCTCACAGCTGGCAATGATGATGAGCTGTTCTCTATTGACGAGAGCCAAGGCTTCCTTAAAGTCAACAAACCGCTTGATTTCGAGATGCAAGCAGTCCACACTTTGACAGTCACGGCAAGAGATCGTGGAAGATTGCCACGTGAAACATCTCGCGACTTCATGGTCTATTTGACAGACATCAATGACAATGAGCCAAAGTTTAACAGTACTTATTACGATGCCTACATCCAAGAGAACTCTCCCAGTGGAACAACAGTGTTCAAGGTAATCACCTCTGACAAAGACAAGGGCAGCAACACGATCACCGAGTACTCCCTCAGAGGCGAGGCTTCAGTAATGGAGAAGTTTAACATCCAGAAGGACACTGGCACCATCCGCTGCCAAGGGAGCATTGATTACGAAAACAGGAGAGACTACCAAGTTATCGTCACTGCAAGAAACCCTGGCACTTCCCTCAGCAGCTCTGCTACAGTCAACATCCATGTGACAAGTGCTAACGAATACACTCCAGAGTTCGACCAGTCCGAGTACGGCTTCTTTGTCAGCGAATCTGCATCCGCTGGTTATAATGTTGGCACGGTCCATGCCTCAGACCAAGACCAAGGAGCTGATGGAATAGTCTTCTACTACTTAGTCGGTGATAGCAACAAGAAGGGATTCATTGTCGAGCCAGACAGTGGAGTCATCCAGGTGGCTGCTGGAGTCGACAGGGAGTCAACTGAGCAGGTTACTCTCAATGTGCTTGCTAAGAACAGAGGGCCCATCAGGGGTAACGACACTGCCATGTGTGTGGTGAGAATTGGAGTAAGCGATGCCAACGACCCTCCTAAATTCACCCAACTCATTTACCGAGGATCTGTGAAGGAAGGGCAAGACCCCGGTACATCTGTCATTCAGGTTTCAGCAAAGGATATCGACTTGAAACCAGAATTCCGAGTGTTCAGCTACAGGATGCTTGCTGGCGCGAACATGTTCGACATCAACTCAAACACAGGTCTTGTCACCACCAAGGTTAAACTAGACAGAGAATCTCAGGGCGTCATCAATATCACTGTGGCTGCTGTTGACACAGGAGCGCCACCAATGACTGGTACTGCACTGGTCTCTGTTGTGGTTTCTGATGCTAATGACAACGCCCCACAGTTTGCACCAACTATCCCAGAAGGTCACGGAAGAGAGAATATCGCCCCATATAGCGAAATCTTGATCAGTTTGACATCGTTCACCACTGACCAAGATATTCCTCCGAACCAAGGTCCTTATCAATACACCTTGCTCAATAATCTGGATGTATTTGATGTGGGACTCAACTCGGGACTTGTACAAGCAAGAGTCACTTTGGATAGAGAAACCATTCCATCATATCTGACGAGGGTCCGAGTCACGGATAATGGCAATCCGACTATGACGTCCACCTTGAGTTTTAGGATCTCTGTTTCGGATGACAATGATAATCCATCCAAACAGAGACCATTGGAGATCATCCTGCAGACCTTCAATGGAGAATTCCCTGGAGGAAAAGTCGCGGATGTGATTCCTTTGGATATAGATGATGAAGGAGATTACCAGTGTGAGCTTGTGCAGGGAAATTCGCAAGTGTTCTCACTCCAAGATGGTTGCCAATTGCACATGACAAGATGGAATAACCCCATTGCCCAGTCGTACCAGCTGAACATCAGTGGCTTCGATGGAGTTCATAATGTTGTCTACTCTCCTATGATGGTCAGGACAGCTCAGTTTGACCAAGCAGCTGCAGAAAACAGCATAGTAATCCAACTGACCGATGTTACTCTTGAAACTTTCCTGTCCGACAGTTACTCCTCCTTTGTAGAAGCTTTGCGAACGTCATTTGGACCATCAGCGTTAATTATTCTATTCTCTGCCAAGAGCGAAGATAATACAGTTGAGGTATTCCTAGCAGTAAAGCAAGGCAACGACTACCTGACACGTACATCATTAGCTGACAATCTGCGTGACAACAAACAAAACATAGAGTCAAGCTCCAGTCTCAATATCGGCATGGTCGACTTCAGTGCTTGTACTAACAGCCCTTGCAAGAATGGTGCTCAATGTGACAGCATCACTGACATCTTTGAGAATGAATTTCACTTCGCAGAGAGCTCCAGGAGGATCTTGACTTCACCTCGCATTGAACTAACAGCTAAATGTATCTGCAGAGATGGTTTTGCTGGTGATAGGTGCGAGACACCACTGAACACCTGCAAGAGAAATTACTGTAGCAATGGTGGTATCTGCATCGCTCATTCCGGAGAAGATTTCACCTGTAACTGCCCAGATGGATGGACTGGAAGAACTTGTCAAGTCGACGTAAATGAATGTGACCAGCAGATTTGCCAAAATGGAGGACGCTGCAGCAATACAAGAGGTTCATTCAGATGTCTGTGTGTTGATGGCTTCGAAGGACCTAAGTGCGAGACCAACAGTGACATCTGCAGGTCAGCTCCATGTTTGAATGGCGGAGAATGTAAATCTCAGGGAGACACACATTCATGCGAATGTCCTTTTGGAGCGTGGGGCAAGAACTGCGAAGAAACAAGCAAAGGATTCGGCGAGTTGTCTTACATGGAGTTCAATGGAGGAATTGTTGGGTCGGGAAGGTTCAAGTTCACTTTGCAAGTTGCAACCCTGGATATGAATGCCCTCCTCGCCTACAGCAGTGCTAGGAATGGACATTTCTTCGCTCTACAAATCATCCAAGGCTATCCTGTATTCTCCATGTCGTCGCAGAGTGGTGGAGACATTGAAGTCACCGTTGCTAAATACATCGCTGATGGGATGTGGTACAGGATCCTTGTGGAACAAAACGAACAGAATATTCTGTTGCGAGTCGAAGAATGTGGTCCTGCTGGAGAGATTTGCCAACCTTGCCAAATGAATGACCAATCATGTTCCAGCAAACTTGTCACTGCAACCATAATAAACCTCCAACTGGGGTCAGTTTCTCTGTGGGTTGGTGGAGTGCAAGACTTGTCAACAATCCTAGAGGGCAATGGCCATGTGGTGACCCACGACTTTGTCGGCTGTGTGAAGACCATGGTCATGAACAACCAGAACCTGTTGTCCGCTACAGCTAGGACGTCTTCAGGGATCACAAACTCTTGCCCAAGGAGGAGAGCTCAATCTGCTTGTGACTTGTCGCCTTGTGTCCACGGCAGGTGTGAAGACACTTGGAAGGGACATCAGTGCCACTGCAAGGAGGGCTACATGGGTCCAGCCTGCGATCAAGTATGGCAGCCATTGGCCTTCAGCAACAATGCTCTTGTTGTCTACCAGCCGAAGGAGACCTACATCAGAGATGAGAGGATGAAGAATGTCCGTTCCAAGCGAGCTGCTGATGGTTCTAGCATCGTGTCCTTGACCTTCAGAACAAACCGTGATCACGGACTCTTCTTCTATTCCGTTTCAAGTCAGGATTTCACTGCATTGGAGGTTGTGTCCAATGGCATACAATACACCAGCAAGGTCGGCAGGCAGACATTCTCCATGCCTCCCGAGGAGGACTTGAAAGTCAGCGACTCGAAATGGCACAATGCAACCCTGATTGTCAAGGGGAACATTGTTACTCTGATGGTTGATGAAAAGGTCCAGAGCCGAACTTTCAGCATGGCTGTCCATGACCCTCTGGACATCGAACTGACCTCCATGTCTGTCGGTGGGACTGAGCAGGTTGTGCGAACCATTGATGGTGTTCAGGCAGAAAATTTGGTCGGGTGCATAGAAGAATTCCGCCTGGATGGAAATCTAATGCCATTCTCAGGATCGACAGCAAGGTTCGAGATCAAACCCCTTGCTGGTGAGGAGGAATCTTGCGAGGCTGTCATGGGAGCCGCCACAGGCGGCAAATCGTTTAAATTAGCGTACATCGTCATCATTGTGTTTTTCGCACTCGTCGTCATTGTCATAATCGTGGTCTTCCTGGTTCTCAGACATCGTCGCAAGCAAAAGGCAGCAGACGGACCAAATATGAATGGCACCGTCAAAAGCAAGAGCAATGGGGGAATACTCGATAAAACCAACAACGACAATCGCAGTCACCAGGACTCTGGCTTCACTGAAAGTGGAGACATATGTGAGGAGAACATCATTCGGCAACACATTGCTGATGAACTCGCTACCCAGTCTTTCAACGAACGGGAGGTGTCCGATCGCCTCCCTTCATCCCGTCCCGACATCATTGGCCGAGAGATCGGTTCCTCACATTCCAGAGACAATAGCCTGCTGCTGGATGGGTCAGGCATGGACAATATAGCGTACTGTGAAGAGCCTCCTGAACACTATGACATTGACAATGCCAGCAGCATTGCGCCGTCAGACCTTGTAGATGTAGTGGGCCACTACAAGAGATACCGCAGTGGCCTGCTGCACAACAAGTACAAGGGAGGGTCCGGTAAGCATCCTCACGCCTCGGGGCTTGGTGGTCAGCCCATCAGAGAAAGCCCCGGCCCCATGCTCAACCACAGAGACAGTCCGTCAATACTGCAGAGCACCCCCTATAACAGAGCTAGTCCAGCCAGTCTCAGCAACCGACAGCAGAGTCCCCTCACCCTGGCTGGCACCGCAAGCCCACTGACTGTAAATAATGTGAATAACCTGTCTAATCCATTGAATCAATTATCCAGGAATAGTCCCCTGAACCAGCTCAATCGCCAGACTCCCACCAGCCACCTGCATGAGAAACACATCTTAGACAAACGCATGAGCCCCTTGCCAAATGCGAACAGTATGAGGTCCACCCCAGTGAATGGGTTGTACCCAAGCAACGTGAGCAGCAGTTCGTACAGTGACCATCCAGAGAGAGGCAGACCGGCCTCGAGATTAAAGCAGCCCATCAACCAATTAGGGTTGCGAGGGACTCCAACGAGGGGTCTGACGGTGGAGGAAGTAGAGCGTCTCAATTCACGCTCCAAGAGTCCGCACAATCCAGTGGACGCTCTGTCTTCCTCCAGCGAGGCCCCCTCCAGGGCAAATAAGTTCCCTCTAAGGCAGAACTTGGCTGTTCACCCAGCTGCGAGGGTCCCTGTGTTCAATGCAGGCAGTATGATGCCTCCCCCTGACACCTCATCAGATGAGGGCTCCAATGACTCTTTCACATGTTCAGAGTTTGAAGACGAGAACTTTAAAGTGCGCAATGACTTTCAACCGAACCCCATCAGGTTCCCTCATCTGCCGGAGACCGATGAGAATGAGGATACAGATGCCAGTCGGACTTTTACTCACGATGGCTCCGTGTCCAACCAAGACTCTCTGAGCACGTTCTTTGCTTCGGAGGATGAGCGCCAACCGAAACACGGCAATAAGCTATTGAACGGTGCTCTGAATCTGGACTACTTCCTCAACTGGGGTCCCAACTATGAGAAGTTAGTCGGGGTCTTCAGAGACATTGCTGAATTACCTGATGCTGGGGTGGCCAGTGCTACTAAAGTCCGAGCTATGGCCTCTCCTGCTCCGGTCGTGCACCTTTCTCCTCCTCCCTCTCCGAGCAAAGAGGACGATACTGTGGGCGAGGAGTACGTATAGCCCACCCCTAACTTAGACATATTGTTATTTTAATATTTTTCATATTCCATTTTGTATTGTACACATGTAAAGGACAAAGTCCACACAGGTTTATGAATACACTGAATTAATGAAGCAAAATCCTGCCAAACTTTTTGTACAAATCTTAACCATACATTTTATACAGTTTGTGTGTACTTACCATTTCTAAAAGAAATGGACCTGTAAATAGTGAATCATCCATCGATCCATTTGTTGATAGTTCATGATCATGATGCTTCTATGCATGTTTCTCCACCTGATATCCTATGTTACTTTCCTTGTACATGTACTACTGTGTTGTGGGCTCATGGAGCCCTAAATTTATAAATGTCATTTGGTTGGTTATTTATACCTGTACAACTTAATGTTTTGTTGATGATTCTTGATAAGTCAAAAGTTTGTTAAGTATCGCAAGGATGAAGATAACTGGGTTTTAGCAATTTTATCAATAAAATTAATTTTAATGTAACTTTTAAAGTGAGGGCCGGACCCCATATCAAGTTGTGAAACACTGTGAATGTATAAGCCTTGTATTTGTACAAATGTGTAAAAGGCTCATCTGTCTCCAAAAATCTGTATTTTGATGGTATCTGTATTAACACTATTTCTTTCAATTATATTTTCATTTAAGGTAATGTGATTAAATAAAAATAATTTTGAACAGTTATGTTAGAAAAAATAAGCTTGAAAAGCTATTTTGGTTTTAATGTATACTGAAATCAGGGAAAGTTTTTACGACATAAAAGTTCTTACTCAATGCAGAATATTTTTTATAATAAAAGAATATTATATATTGTAATGGTGTAGTTTGTACTTTTT

Protein: 4999 aa

>Fat_protein

MARRDRWICRKALNWTMFRGLLSHVLILQFLLTFAQGQGHGEAGVPPTDERVSFTVQEELPAGTEVGTISTRAGLTYRFAEDPALFHLDPRSGRITTTTKIDREAIRSDNFDLFIQSLPSARHLIEVRITVLDINDNSPQFGSNTIQISFSENDKPGTQVILDTATDKDTGIFGVTTDYVIVSGNEEGKFRLVPLLDTSKPLLYLENMVDLDREEKDFYTLQISVPDGGSPPRYGYLTVNVTVLDVNDNGPLFDQSDYWVSVNETIPVRSSIIQIRATDQDMGANGDITYFIVTDDYNQFSVDAKTGVLRNSDKLKCPTTCSPGEVASNSCKPRTCLIIVEARDGGRPIPMNGRAYIHVSLIDENDHDPTITVQYTPSGSTYAMVDEGAKESIIAVVSVTDDDDGVNGQTDLQITRGNERGHFRMVSITFANVIRVVGKLDRERVSKYNLTVEARDRGFPQRSSTAYVIIVVNDVNDHEPVFQMKSYSTRLSELVPIGSFVASVTATDNDTGINALITYSITAGNNLGWFTINNATGLVTTKAQLDHEQLSTVVLTIRAQDGATEPTQTSTNLTISIWDENDEVPNFSEDTYRVTLMEGLGAGREVITVKAEDNDQGLNGSVTYALDPDVDLMYPNMFSIDTVSGRITTRSILDREVQSTYNLVVRAQDAGNPPQSSTATVILNVTDINDNAPVFYPKNYYARVLENEPIHTSVVQVQASDPDFGDNGTVFYSIQSGAERLFAIHITNGWISTIGDLDREKKASYRITISAEDKGGLRALENAIVEIGVSDVQDSPPEFPSSGYRFNILEDDSRRNARTGRRVGQVKATSADTLGTITYAISGGDKGGHFNINENSGVISTSKGIDRELQEVFSVQIVAKAGGMYGHTTVNISVMDVNDNVPTFNHDQLEGYVVENWPVGHEVFLASAMDADNGINSSLTYSMLPGSSGVFAVNRTTGMIYLAKSLSQSTQNSYTVQIEARDAGSPSLSSSTSVSIILRDVNDHTPDFEQTTYEVSVLESRPVNERFFSVSATDADAGLNGEVVYSIKDGNTRGSFGIFPDGVLYIASELDRETTDLYLLTVIAKDKGVEARSSSANVTVHVLDDNDNRPAFLNDTYTMSVKEGSPVQTFVGLVSAKDLDLGRNAEVTYSIEGKDIRFQVHPKSGAITTNRVFDREGQVEDMGSDVISIVVIASDSGLTKQEDRAVVNIDILDENDNDPVFARNYYEPSLYEDAEINTQVVRVGATDVDKGDNAKLTYEIVSGNEAGRFSINEVTGQITLTSRLDREVVSEYQLTVLAKDNGKPSRNSTTQVKVKILDNNDNMPRFSETQLTVDVEETSQPGAYITQVHATDMDIGVNSEISYSISAGDPGVKFRIDGSTGKIYVADYLDYESQRQYVLNVSAKDMGSPSLSSGMTCKINVLDSNDNAPSFTTASLVRQIEEDTRIGTSVITVRATDPDSGPNGQLTYSIIRQEPRGNHFVIDPDSGLVRTASAIDREQVSSFKLTIQATDQALQVTSRKSALKQVTIIVVDVNDNSPRFVSMDAVVLKQSTSAGSVVARVEAVDPDEGPNGQVEYDISQGDTTLFSIDTNTGDLTINSDTNALSRGPVLLTIRARDKGTADRLGPQSSSFRMTVFVESGQPGPSFVETSYRGQLYENEASGTSIVTLQAAYADARSGNVQYYVTNVTGEGRGQWRYFQVNPTSGVLSSQGVLDRERGVQEFTVDVYAVDTSSPAIRTTATKVLVTLLDENDSPPLFTPDTYDNVRVSEDAERGYVIMSVVASDADQQGLLQYSIVSGDDDSQFAINPTSGELRVVQPLDREVHSTYNLIVQATDGKQTSVASVHLHVSDVNDNAPHFDTPYYVFEVSETARQGTTVGLVSATDEDEEFNAQVTYELQSQWAKERFNLHPNTGVITLQSAMDYEERPFYLLTVMAKDNAVDSMSSSVTVYVNVEDVNDNAPVFDPSSYNNEIWENVTIGSRILTVTATDPDSGVNGQVRYRLGSGGAEVWGPFKVDAVTGELTTAGKLDREKQGYYNVPVVAYDMASNVMESLSSTAMVTVILKDVNDNAPMFVSNKEVVVMENLPSGMSVFQVEAEDADEGRNSYIEYSLRRSDQSLFEVNVVDGTVKVKQSLDRERRDLYQIQVTAMDKGVPPQSSTMDLTIKVGDDNDNSPVFSPRSYRKTVSEDLKVGSILMTLTATDKDTGLNADLRYIITSGDDNQDLWLDSHTGQLYIQKRLDYERKRTYNVSVLVEDLGDPPKSDKAVVTITVTDVNDNAPVFIDSPYVAYVRENLDTFPVHVAQVSARDEDSAPNSVISYSMLTGDRSLFKVNSSTGEIEALRTLNREEREEYSITIRAMDSGSPRLSSTGTVLILVEDVNDNTPVFQHQMYQAIISENAPPSSPVITVQASDEDAGINAHLRYSLSDDGNSYFTVVPETGVIKTATNLDREVRDKYTLVVVVADGGLMARSASAIVVVDVEDVNDMKPEFTQRTYTTYIRDPTSAGVFVLGVTAVDPDLGMNGEVTYSLFGSQDVNNFNINPDTGIITAKNNMIGQRSYRFMVRGTDKGQNPLSDEATVEVNVLPSTTVGFPNLQTPETDFSLSEGTATGTTLVTVTGTSSRPSVSLTYFIAGGNMGQAFEVTSAGVVRLRNQLDYEKTHQYELWIGVRDDGSPSLTHYIKLIVNVEDENDNSPRFRTTYFSATATEEENPPTIVTTVTATDADSGSNGRIKYTIAGGDNNNDFAIDANTGVIRTNQKLNREYHSQYTLEVTATDHGSPARTSNAIVDIAILDKNDEPPVFTQHFHTQIPENTPPGSFVMKITSTDADDGNNAQHQYSFTENPGQRFAINRNTGDVSVAGPLDYESKNEYILKVSANDRAYSVETTVSIYILDVNDNAPSFLSSQYMFMVVEKQPQGTSVGTVSAMDRDSNGVFSQVFYLMKTPSKHFQLDFDTGEIKTRQQLTHKSLPSGDSPENKHVLTIAAMDGGDPPRSSEVQVTIKIVSANQKAPVFIMESNSSAVSIDATIGQTVFTVSASDDSNGGKVTYSIIGGNDTRFFQLNEDTGVISLRNIVRGQQGRMYQVLVRATDEGVPQLSSTVAVSIYIEEINYNSPRFLDNQAFQVTIAENREVGDVIGSVAATDSDAGNNGMVSYFLTAGNDDELFSIDESQGFLKVNKPLDFEMQAVHTLTVTARDRGRLPRETSRDFMVYLTDINDNEPKFNSTYYDAYIQENSPSGTTVFKVITSDKDKGSNTITEYSLRGEASVMEKFNIQKDTGTIRCQGSIDYENRRDYQVIVTARNPGTSLSSSATVNIHVTSANEYTPEFDQSEYGFFVSESASAGYNVGTVHASDQDQGADGIVFYYLVGDSNKKGFIVEPDSGVIQVAAGVDRESTEQVTLNVLAKNRGPIRGNDTAMCVVRIGVSDANDPPKFTQLIYRGSVKEGQDPGTSVIQVSAKDIDLKPEFRVFSYRMLAGANMFDINSNTGLVTTKVKLDRESQGVINITVAAVDTGAPPMTGTALVSVVVSDANDNAPQFAPTIPEGHGRENIAPYSEILISLTSFTTDQDIPPNQGPYQYTLLNNLDVFDVGLNSGLVQARVTLDRETIPSYLTRVRVTDNGNPTMTSTLSFRISVSDDNDNPSKQRPLEIILQTFNGEFPGGKVADVIPLDIDDEGDYQCELVQGNSQVFSLQDGCQLHMTRWNNPIAQSYQLNISGFDGVHNVVYSPMMVRTAQFDQAAAENSIVIQLTDVTLETFLSDSYSSFVEALRTSFGPSALIILFSAKSEDNTVEVFLAVKQGNDYLTRTSLADNLRDNKQNIESSSSLNIGMVDFSACTNSPCKNGAQCDSITDIFENEFHFAESSRRILTSPRIELTAKCICRDGFAGDRCETPLNTCKRNYCSNGGICIAHSGEDFTCNCPDGWTGRTCQVDVNECDQQICQNGGRCSNTRGSFRCLCVDGFEGPKCETNSDICRSAPCLNGGECKSQGDTHSCECPFGAWGKNCEETSKGFGELSYMEFNGGIVGSGRFKFTLQVATLDMNALLAYSSARNGHFFALQIIQGYPVFSMSSQSGGDIEVTVAKYIADGMWYRILVEQNEQNILLRVEECGPAGEICQPCQMNDQSCSSKLVTATIINLQLGSVSLWVGGVQDLSTILEGNGHVVTHDFVGCVKTMVMNNQNLLSATARTSSGITNSCPRRRAQSACDLSPCVHGRCEDTWKGHQCHCKEGYMGPACDQVWQPLAFSNNALVVYQPKETYIRDERMKNVRSKRAADGSSIVSLTFRTNRDHGLFFYSVSSQDFTALEVVSNGIQYTSKVGRQTFSMPPEEDLKVSDSKWHNATLIVKGNIVTLMVDEKVQSRTFSMAVHDPLDIELTSMSVGGTEQVVRTIDGVQAENLVGCIEEFRLDGNLMPFSGSTARFEIKPLAGEEESCEAVMGAATGGKSFKLAYIVIIVFFALVVIVIIVVFLVLRHRRKQKAADGPNMNGTVKSKSNGGILDKTNNDNRSHQDSGFTESGDICEENIIRQHIADELATQSFNEREVSDRLPSSRPDIIGREIGSSHSRDNSLLLDGSGMDNIAYCEEPPEHYDIDNASSIAPSDLVDVVGHYKRYRSGLLHNKYKGGSGKHPHASGLGGQPIRESPGPMLNHRDSPSILQSTPYNRASPASLSNRQQSPLTLAGTASPLTVNNVNNLSNPLNQLSRNSPLNQLNRQTPTSHLHEKHILDKRMSPLPNANSMRSTPVNGLYPSNVSSSSYSDHPERGRPASRLKQPINQLGLRGTPTRGLTVEEVERLNSRSKSPHNPVDALSSSSEAPSRANKFPLRQNLAVHPAARVPVFNAGSMMPPPDTSSDEGSNDSFTCSEFEDENFKVRNDFQPNPIRFPHLPETDENEDTDASRTFTHDGSVSNQDSLSTFFASEDERQPKHGNKLLNGALNLDYFLNWGPNYEKLVGVFRDIAELPDAGVASATKVRAMASPAPVVHLSPPPSPSKEDDTVGEEYV

Domains

(34x) Cadherin: 53 – 137, 145 – 249, 258 – 367, 382 – 478, 486 – 582, 591 – 691, 700 – 794, 804 – 900, 910 – 1004, 1012 – 1108, 1116 – 1217, 1226 – 1322, 1332 – 1427, 1439 – 1536, 1546 – 1638, 1650 – 1752, 1764 – 1854, 1863 – 1959, 1967 – 2069, 2078 – 2172, 2180 – 2277, 2285 – 2382, 2391 – 2480, 2494 – 2584, 2627 – 2692, 2701 – 2796, 2804 – 2897, 2905 – 3006, 3021 – 3111, 3121 – 3217, 3226 – 3321, 3330 – 3428, 3436 – 3532, 3546 - 3639

(3x) EGF: 3886 – 3928, 3930 – 3968, 3970 - 4004

LamG: 4010 - 4176

EGF: 4207 - 4231

Lamin_G_2: 4282 - 4408

Clone

Partial ORF

Primers

Forward: GTACCCCCAACTGACGAGAG

Reverse: TGGTCTGTGGCTCGTATCTG

Nucleotide# 618 – 1324 = 707 nucleotides

>Fat_clone

GTACCCCCAACTGACGAGAGAGTATCTTTTACTGTCCAAGAGGAGTTACCTGCGGGGACCGAGGTTGGCACAATATCCACCCGGGCTGGACTGACCTACCGATTTGCAGAGGACCCAGCCCTGTTCCACCTGGACCCCCGGTCTGGTAGAATTACAACAACTACAAAAATAGACAGAGAGGCTATTCGTTCTGATAATTTCGACCTTTTTATACAAAGTTTGCCTTCTGCTAGACATCTCATAGAGGTGCGCATCACAGTATTGGACATCAATGATAATTCTCCACAATTTGGCTCAAACACCATCCAAATTTCATTTTCTGAAAATGACAAACCTGGCACTCAAGTCATTCTTGACACTGCCACAGATAAAGATACTGGAATATTTGGTGTGACAACTGATTATGTGATAGTTTCTGGGAATGAGGAGGGCAAATTTCGACTAGTGCCCCTTTTGGATACCTCTAAACCTCTGTTGTACTTGGAGAATATGGTTGATCTGGACAGGGAAGAGAAGGATTTTTACACTCTACAAATCTCTGTGCCTGATGGTGGATCCCCTCCCAGATATGGATACCTAACAGTGAACGTGACAGTCTTGGATGTGAATGACAATGGACCTCTCTTTGACCAGAGCGACTACTGGGTCTCTGTCAATGAGACCATACCTGTGCGCTCATCTATTATACAGATACGAGCCACAGACCA

Cloning info

PCR rxn on 07/28/16 using GoTaq polymerase mix

| Template | Initial denature | Denature | Anneal | Extension | #cycles | Final extension |
| --- | --- | --- | --- | --- | --- | --- |
| 2 hr cDNA | 4min @ 94C | 30sec @ 94C | 30sec @ 55C | 2min 15 sec @ 72C | 35 | 10min @ 72C |

**Dachsous**

Gene model: 11517 nucleotides

ORF: 275 – 10234 = 9960 nucleotides

>Dachsous_full

TCGACAGGTGAGAATGACCCAGTTCTATGGAGCAGCAGACGAACTATTGGCAGTGGCCTATGAGAACTGGGTCTGGCAGGCCGCGTTTCACACACAGGGAATATAAACTGTCTCTGTGAGGGGTAAAAGAGGATAATACACATGGAATACCACACTAGACAACACTAGCATGCATGTAGGAGGGAGTTGCAGTAAATAATGATTTTGCTGGTGCACACCTGAGTGACAGGACTTCCCACCTGCGTTGGGGGCAAGCAGGGGCAGTAAGCTAGACATGAAGGGTAGGAGGACCAAGGGGGTTAGGGTGGTTGCCCCCTGCCTTCCCCTGCCCACAGTCTTCTGCTTGCTATTCACATTCTTGTTGTTGGTCAAGCATGGGACATGCAATAATGAGGAGGATTTCACACAAGACTTCCATGTGGAAGAGGAACAGGGCCAAGTTATAGTGGGCACCATAGCTGCTGGTCAGCCTTGGGCGTCCCAGGTTCCATCCCCGTACTTCATAGTATCAAGCCAGAACAGCGGCGTAACGGATCACTTCGAAGTGAATGAAAGCGATGGCATCATTCGGACTAAGACCAGCTTGGATTACGAAGGACAGAAGCAGTATACATTCGTCGCTATTTCGATATTCAACAGTCGAAACGTCAGGGTTAATATTTACGTTGAAGACATCAACGACCACAGCCCGAGTTTCAAGAATATCGAAAAGGTTTTACCATTATTAGAATCTGCTCCAAGAGATGCCAAATATACTTTAGGATCAGCGTCTGACCCTGACAGAGATCTGAACTCAACGCAAAGGTATGAAATCGTCGAAGGCAACATCAACAACGCTTTTAGGCTTGAGACGAAGCCGAGACAGAATGACGCTTTACTATTCGATCTTGCTGTTAATGGGGAGTTAGATTTTGAAGAAGTGCCTTTGTACAGCCTTGTCATCAGAGCCTATGATGGCGGGAGACCTCCACTGTATGGCTCTATGCGCTTGAACATCACTGTCATAGATGTGAATGACAACCAGCCCATATTCAACCAGAGTAGATATTTTGCTAAATTAAAAGAGAACGTAACGTTGGGGACCCCTGTCCTCCAGGTCCAAGCGACGGACAGAGATTCCGGAGAGAACGGAAAAGTGCGTTATCAGATCGACCGCCAAAGAAGCGACCAAGAACAGCATTTCGACATCAACCCGGTCAGCGGTGTCATCAGCGTCAACAAACAATTAGATTATGAGATGAAGAATGTTTATGAATTAATAGTTGTCGCTAGAGATAATGGGACCCAAAGGTTACAAACTACTGCTGTTGTTTCTATACAAGTTGAAGATGTGAATGACAATGAACCCGTTATAAACATCATCTTTCTAACGGAAGATGAGTCGCCTACTATATCTGAAGATTCGGAACCAGGCGATTTTGTCGCAAGGATATCTGTGAGCGACCCTGATATAGATACATATATTTCTCATGTGAATGTCACTTTACAGGGAGGAGAAGGAAGGTTCGGTTTGACTACCAGAGACAATGTTGTGTATTTGGTTATTTTAGCGAAACCTTTAGATTTTGAGATACGTTCATATTACACTTTAACCGTAGTTGCCATTGATTCTGGAATACCACCAAGGATCGCTCGCAAGAATTTTACGATTCATGTGGGAGACGTAAACGACAACCCACCGCAATTTACTCAACAGTCTTATGAGGTGGAAATCCAGGAAGTCCTTTCAGTGGGCGCTTCCGTCATTCAAGTCACGGCGAGGGACCGTGACGGAGGGGACAATTCAAAAGTGTTTTACTCCCTCGTCCCCTCTCCGGATACCCAGTATTTTACCATCAGTAATACCACCGGTCTTGTGACCACTAAATCCAGAGTGTACTGTGATACGTCTTCGGAACTGTCCTTCCAAGTTCGAGCCACGGATTTCGGATTACCTAGGTTGAGTTCGACCGCTAATGTCGTAGTGAAAATCAAGGACGCCAACGACAATCAGCCGGTGTTCGACCAGACCTTCTACAATGTGTCCATCCCCGAAGACCGTGCTGTTGGATCCTGTATTCTTAAGGTGTCTGCATCTGACCCTGACTGCGGTCGCAATGGCGAGGTCACCTACTCCTTTGCCAGGAACCTTGGATTCGAGGTCCCCTTGGAGCTGCGCATTGACGGAGCCTCTGGCGAGGTCTGCATCGATCAGGCTTTGGATTACGAGACAAAAAGCAGCTACGAGTTCCCAGTTATGGCCAGTGATCATGATGGTTTGCACACCACCGCCATGATCAAAGTGGTCGTCCGAGATGTGAATGACAACGCCCCTGAGTTCTACCCCATCCAATACAATGCCAGCCTCGACGAAGCAAGTTCTGTTGGTCAAGAAGTTGTGGCTGTGCATGCAAGAGATGCAGATTCTGGGGTCAACGGATTTGTCAATTATCGGATCACCAGTGGAAATACTCAGGGATACTTCTCCATCAACTCTGACACTGGTGTAATAACAGTGAGAAGAACCTTGAGCCTTACCCAGCGATTGTACACCTTGACTGTGGAGTCAGAGGATGGCAGTGGACGAAGGTCAAGCAACCAAGCTGTCATTAGAATCAGTATTCTTGATGCTTCTCAGCAACCTCCTACATTCTCTACCTCATTGTACCAGTTCAGAGTAGCGGAAGGAAGACCCACTAACTCATTGGTCAACAGAGTGTATGCAACAAACAGTAATCCAGCTGCTGGGTTCATCAGATACAGGATTGTGGCAGGAAATGCTGATGGTCTGTTCAAGATTGAGTCAAGAAGTGGTGAGATTAAGACACAAGCTGTCCTGGATCACGAGTCTGTTCCTTACAGCTTGCTGACAGTGCAGGCGGAAAGTGGCAACCCTGTGTCTTACAGCCAAGCTCAAGTTAACATCAGCATTGATGACATCAACGACAACAGTCCTGTGTTCCCTGTGCGAATGTTGGAAATAGCTGTTCAAGAAAATGCCGCTGTCAATTCTGTTGTCTACTCCGTTCTTGCGACTGACCAGGATTCCGGAATGAACGGCAAAGTGCAGTATTCTCTACAAGATTCCAGTGGGGTGTTCAAGATTGACTCGGCAACAGGCCAACTGAAGATTGCCCGTAGCCCCGATTATGAGAGCCAAAGTTCATACAGGATGTCCGTCACTGCCAGTGATCAGGGTGCTCCTCCTCAACAAGCCACAATGCAATTAATAGTTAACATTCAGGATGTCAATGATAATTCACCAGTATTCTCCAAGTCGCAGTATAATTTCAAGCTCGCTGAAGGTGCTCCTGTCAACACAGTCATCGGTAAACTCAATGCTACAGATGCTGATAGTGGAAACAATGGAAAGATTAGTTACACCCTTCAAAACGGACCTGATTCGGATATGTTCGGAATCTCTTCGGACTCCGGAAACCTCTTCAACAAAGTTGTCCTTGACAGAGAAGATAAAGAAGAACGAGTATTAACAGCCGTTGTAGTTGATTCAGGAATCCCAGCTAGGTCCTCATCTGTAGTTGTGATCCTGACTGTTCTGGATGAGAATGATAATCACCCTCGTTTCTTAGAAGATGCTTACGTTTTCAAGGTAAATGAGAACTTGCCAAGTGGCGCTGATATCGGAGTCGTCCAAGCTACTGATCCGGATTTCACCGACAACCAAAGGCTCGTCTATTCTCTGCTGCTACCAAGCGACCAGTTCCAGATAAACGAGAACACAGGTGCCATAACAACCAAGCAAGCTTTGGACCGTGAAGTGGGAGACAAGTATCAACTGACTGTGAAGGTGTCGGATGTTGGTTTTCCAACTGCTACTGCAATTGTTAACATCGAAATCCAAGTCCTTGATGTTAACGATAACTCTCCGGTGTTTGACCAATCCGGAACTTATGTGGTGTACGTGGAAGAGAATCAACCTAAAGGACACATTGTCACCACCGTAACAGCCCACGATGCTGACGAAGGCAAGAATGCCGAGATTTCTTACTACCTCCGAGAAGACTCACAAGAGAATTACAAATTGTTCAAGATCCATCCTAGGACGGGACTTCTCACGACCCAAGAAGTATTAGACCACGAGGAGAGGAGTCTGTACACCCTCCAAGTGTATGCCATGGATGGAGGAGCACCTGCAAGAACCGCCATGACAACTGTTGAAATAAACGTCCAAGACATTAATGAAGGTGGACCCGAATTTGATGCTCCTACTGTAACATTTAACATCAAGGAGAACTCTGCACCAGGTACTGTCGTGGGCGTCATCAAAGCTCGTGACCAAGATTCCGGAGATAATGGAGTGATCCATTATTACATCATTGGAGGTAACCTCTTCTCGATGTTCGGCATCAATCAAACTACAGGCAGCTTGTACTTGGAACAATCTCCTGACTACGAAGTTGCTTCATCTTATTCCGTTATTGTGAAAGCAGTGGATAACAGCATCACCAATCCTCTGAGCAATGACATCAAAGTTCTCGTTAACGTCATTGACGTCAATGACAATGCTCCTGTATTTGATACTGATCCTGTGGTGGTCACCTTCAGCGAAAATGAACCTGTCGGATCTACTGTCTACGTCTTCAATGCTTTTGACGTCGATGGTGGAAATGCTGGGCTTGTTCATTACAGCATTGTGTCTCAATCTCCGGAAAGCCAGTGGTTCTCGATTGGAAGAACTGACGGTAAATTGGTATCCAGCAAAGTGATAGATTATGAAGAAACCAGCCAGATCTCTCTCGTAATTGAAGCTCGGGACCAACCTGAAAACAAGAGAGACTCCCTATCTCGCACTGTGACTGCTGTTATCATGATCAAAGACCTGAATGATAATGCTCCTAAATTCATCCAGGAGCGGACAGAATTCAACATCCTGGAAGATGAACCTCTCGGTTACCCTGTCATGCATCTTGTAGCAAGAGATGACGATTCTGGTCAAAACGGAAAGGTCACTTACTCCATAGAGTCTGGAAACAACGGCAAATACTTCAGTCTCAATCCTGAAACTGGTTTACTTGCTATAGCGAAGGGCTTAGATCGAGAGGCAGTGGAGTCTGTGAGGTTGCAAATTATCGCTAGAGACCACGGCTACCCCCAGCTCTCATCTGAAGTCTACATCACTGCAAACATCATTGATGTGAATGACAACAAGCCTAAGTTTACTCAGAAAGTCTACAATGCAAGCATCATGGAGAACCAACCAGTTGGGACTTTTGTGACTGCCGTTTCTGCTGTAGACCCTGACCAAGGCGCTAATGGGAAGATAGTGTACCTAATACCCTTTGGCATGGCTGATTACCACTTTACCCTTGACGAGAAGACGGGTCGTATCATGTCGAATGCAACTCTTGATCGAGAGGTTGTGGATAAGTGGACTCTGACAGCCTACGTTAAGGACAGTGGTTACCCGTCATTATATGACACGGCTACAGTGGTGATTGATGTGCTGGATGAAAACGATCACAGTCCAGAGTTCAGGGATCCCGTCTACAGAGTTGAAGTCGCAGAAAATGAAAAAATGAAGGTTGTTCACACAGTTATTGCTACAGACAAGGACTCCGGAGAAAATGGAAAAGTGTCATACTCCATCATAGGTGGAAACACTGATGGAAAGTTCAGCCTTGATTCATCGACTGGCATTCTAAGTTGCAAACCTCTGGACAGGGAAACCCAATCCTCTTACAACTTGACCATCGTGGGGCGAGACGGAGGAAGACCATCTCGCAGCGCCACCTGCACAGTCCTTATTGAAGTAATGGACGAGAATGACAACGATCCAGTATTCAACAAGCCCCTGTATTCTAAGTCCATCAGGGAATCGATCCGGATTAATACGTCAATACTAAGAGTCTCTGCCACTGACGATGATGTCGGAAACAATGCTTTGATCACTTACTCCTTCGATAATGATGCCGATGGTTTGTTCAAGATCGATAATCAGACTGGAGTAATCAGGACAGCAAGTGAATTTGACAGGGAGACCAAATCCAGCTACAGCTTCCAGGTTTTCGCTAGTGACGGTGGAATGTACGGCCCTCGGTCTCGCTCTGTGCAGGTCGACATTTCAGTTGAGGATGTCAATGACAATGCTCCTGTCTTCGAGCAAGTTCCTTACAGAGTGAACGTTTCTGAAAACCATGGGGTCGGTCAGTATGTGTTGCAAGTATCTGCCAAGGACAAGGACCAGGGAAGCAACGGAGAAGTGATGTACAACTTTGCCCTAGAGTCTCCCTACTTCGATATGGACTCCTTCTCTGGTGTCATCGTGACTCGTCAGTCCTTAGACTCGGCAGCTGTTCGTGTTCATCGGCTGGAGATCATCGCAAGGGACAAAGGACAGAATCCCATGTCTTCCACTGGTTTGGTTGAGATTCGGGTCGGCAATGCAGCTGGTTACGCAACCCTCATCTTCGACCGTGATGAGTATTCTGCAGACGTTCAGGAAAATGCCGCTGTTAACACTCCAGTTATTAGAGTCCGGGCTCGTCATCCTAACACTCCCACTGCTGCTATTTCTTACACCTTTGCTAGTGGCAATGAAGCCAACATCTTCAGTATTGACAGAATTTCAGGTCAAGTCACGGTGAGAAATTCCTCCACTTTGGACTACGAGTCTCTGAAACAGCTTCGCTTGGTCATCTCGGCCACAGCTAGCGCCGCCTATGGCTACGCTACTGTCTTTGTCAACTTACTAGACATGAATGACAACCCGCCTCACTTCACTCAAGACCGTTATGTATCTTCTGTCTGGGAGGGATCGCCGAGAGGAACGTTCGTCACTCAGATCCGAGCCATGGATGCTGATGAAGGAAGAAACGCTCGTGTGACTTACAGTATTATTGGTGGAAATGTGCAGAACGCCTTCGTCATTGATCCTCCTGAAACTGGAATCATCAAAGCGAACATCATCCTCGACAGAGAAATCAGAGATTCGTACAGATTAGAGATTGAAGCGATCGATGGAGGGTCTCCTCCTCTTAGCACTCACTGCGTACTCAGGATTCAGATCATTGATGTCAATGATAATGCTCCTTTCTTCCCTCAATACAGTCCCATCTCTATTCCTGAAGATGCCCAACCAGGCAGAGAAATCCTCCAGGTTACAGCCAATGACAGAGACATGAACCCAACGTTGTCGTACCATCTTGTACCTGACAGAAGCTACGAGAATAAATTCAACATCGATCGCTTCAGTGGTCGTGTGTCCATCACTAAACCTCTGGACTATGAAGAGATGCAGCAGTATGACCTTCGGATCATGGCCAGCGACAACGAGTACAATGCTACAACATCAGTCAAGCTGAACATCGTGGATGTCAATGACAACCCTCCAGTATTTTCACAACAGTCCTACCTCGCCACTGTCGCTGAGATGACTCCACCGAACTCTCCCATCATCCAAGTCAACGCAACTGACGAAGATGCTGGCCAGAATGCTGCTGTCACCTTCTCAATGCTTCCTATGAACGGTTTTTACATTGATGAGATTTCAGGCATAATATACACAGACAAGACCCTGACATTCTCCCGCAGTCAGCCCATCACTCATCTTGTTGTGACCGCAAAAGACGCGGGAACTCCTTCTCAAACTTCTGTTACTGCTATCCAACTGCAGGTTGTTGACGTCAACAATAATGCGCCTGTCTTCAGCAACACTGTTTATAGGGTGTCTGTCCCAGAAGATGAAAGAAAAGGACATGTCCTAATTTCTCTGACTGCGACAGATGAGGACGATGCGGTCGATAACAAGAAGGTGGATTACAGCATTGTCAGCGGAAATGACAACGGATTATTCGATATCAGTTCTAGCACTGGAGATGTCATGCTTGTTGGGAGCTTAGACAGGGAGACGTCTCCTGACTTTGATTTGCAAATCATGGCCAAAGATCGTGGCGACCCTCCTCTGTCTTCGACTTGCAAAGTCCTCATAACTGTGGATGATATTAACGATAACTCTCCCGTATTCAGTCAGAAGTATTACCAAACAACAGTGCCTGAAGATTTAGAAGAAGGAACTGACATCATAACAATCACTGCTGTAGATATGGATAGTGGAGAGAACAGTCGTGTAACTTATATCATCGAGTCCGGCAATGATGACAACAAGTTTGAGATCGATCGTGATTCTGGCCTGATGAAAGTCCGTCTCCCGTTGGATTACGAAACTCGCAAACTCCATCGACTCATCATCAAAGCCTCCGATTCGAGCAAGACCCACCAAAGATCTGCCTTTACGACTGTCACTATCAATATAACTGATGTGAACGAGTTCGATCCTGACTTCCCTGTGTTCATGTACATGGAGACTGTACTCGAAAATGAACCTCCAGGAGCGTACGTTTTCACTGCTCACGCTAATGACAAGGATGCTGGCAATTACGGCCAAGTATCGTATGAACTGATTAGTGATTTGCCCAGGTTTGCGATCGATCAACTAACTGGAGTGGTCACAACGAAGGAGATGTTTGATTACGAAAGTTCTCAGGAGACGTATTCCTTCTACATCCGTGCCACAGATCTCGGCGGCCGTTCGACAACTATTCCTGTCATGGTGTCAGTGTCATCAGTTGATGAGTTCAAACCAAGATTCGGCAAAGCTCGTTATCAGTTTGAAATCCCAGGCAACGCAGAAAAGGGCTTCATTGTCGGTCAGGTGCAAGCCTATGATGACGATCAAGGCAGATCTGGTGAGATCAGATACTCGTTTTCGGAGGAGCATGAATATTTCCATGTCGTACCAGATACTGGTGTGATCATGGTCATCAAAGACTTGCAAGATAACGGAGCTAAGAGAGAAAAGAGACATACATTCTTCCTCAGTCGTCAGAAAAGAGCTCTCGAAGGCAATAACATCACATTAATCATCGTAGCGTCATCCTGGCTGTCAAACTCTTTAGTGGATCGTACGAAAGTTGAACTGGCTATCAATCGTACTTGTGTTGGATGTGCGTATCTTGGCCAAGTGGCTACTGGAGATCTTAGCGGTACGCCACTCGTTCTCGTCAGCCTGTTTGTCATTGTTGTGGTGGTCCTGGTGGTCATTGTATTAATTGTTTACTTGAGGAGCCGTGAACGCAAGCGCAGACTTCCGAACAGCCAGTATGACAGGTCGTTTGACAGCATGAACATGCCAGCCGCCCTGCCCAGGGACCTCGCTCCTCCTACTTATAACGAGATTCACCAGAACCACTACAACCGATCTCACGACCACAACATCACCACTTCGGAGATCTCCGACCAGTCCCACTCCGCATCCAGCGGAAGAGGGTCCGCGGAAGAGGCTGATGATGTGGACGAAGAGATCCGCATGATAAACGCAGCTCCCATGCAAACTAAAGGGCTGCGGATAGCCCAGGATTCGGGCATCCAACAGGACGATGATGCCGTGTCCGAACACTCAGTGCAAAATCACCAGGAATACTTAGCTCGTTTAGGAATAGACACTTCAAAACTAAAGAGCATTCCTCAACAGCAGCCTGCCAAACCTCCCGTAGGCAGCTCTGTCGAGAGTATGCACCAATTTGCAGAGGAAGGAGGCGGTGAAGGCCCTCCTCCCAAAGTACCTGCAGTTAACGCGATCAAGACTCGCGAATCTTCCAGAGACCCTGATGGGACCCATGAGTTCCAGTACACTGAGCCTTCGAACAATACTGCAGCTTCCTTATCAAGTGTGATAAACAGTGAGGAGGAGATCAGTGGCTCCTATAACTGGGACTATTTATTGGACTGGGGTCCCCAGTACCAACCTCTTGCTCATGTTTTCTCGGAGATAGCTAAACTCAAAGACGATAGCATTAAGCCGAAAAATAAATCGCAATTAGTGCCACATAAACAGAACTTGAACGCACTTAATCAGAACAAATCGTACCCACCGCCTTTGCTAACTAGTGCTCCCCCAAAAGTCCTGTTGGCTCAGCCTGTGTCCACGAGACCGTCCAACAACTCCTCGTCAGGGGGATCCAGCCGAACCAGCCAAATGGTCTCCCTGCCCAGCCTGCCACGGTCGCCCATTAGTCACGAGTCCAGCTTCACTTCTCCTGCTCTCACGCCCTCGTTCACTCCATCCCTATCACCGCTTGCCACCCGCAGTCCGTCTATATCACCCATAATCACCTCCAAAGATGGCAGCACTAACCACTTGAACCACCACCACCGGCGCCCTCCTGTGGTAATCGAGAACAGACCTGTGGTTAATAAGCAAGCATCCTCTGAATCAGAGCAGGAGATACACATCTAGACTTTTATAAATTTATCATTATAAAATTGACTTGTTAGTTTAATTGACCAATGTGTCTTGCACAAGCTCAAATCATCACAGTTATTATACACATATTTTTTAACATAGCGACTATGCATGACATATCAAAGGTGCCTCTCCATTAAAAATAGTCTTTTTATGACATGTATTTATTTTCATCTAAAGGCACTTGCAGTCATATTTTTGAGCTATTGCTAACCTGCATAGATTTCTAATGAATAGAATAGTCGTATTTTTATATGAGTTACATCGTAAAATGTTTACAAGCACCATCTTATATTGTGACAATCATTTGATAAAATTTTCTTTGCATTTAAATGTACAAGCCTAATAGTAAGAGACATGTAGTTGAAATTTATTTGCAATGCTTCTGACATGAGTTTTTTCATTTTGGTTTAATATCAAAAATGTTGGAATGAATGAATTGATTGCCATATTTTGTAAAATGAACAATTTTGATTTGAATACATGTACTTGTATGTCAGAGATATCATTTAATTGATTTCAATAATTTCCTTTTTTGTGTAAAGTAAGTTGGGCTGCATGATTTGATGCATGTAAGATTTTAAATGAACTGATTTTAAATGTGCATTTATGGCATCAAGTTTGTTTGGATTCATAAATTTTTAAAGGTTCTCCAGTGCTGGTCATTGGACATATTCCTGTGGTGCGCTTAAGTTAGACATTTTAAGAATAAAACGTAACTTGCATATTGATGAAATTGTATAGTTATTCCTGGAAGATCTGTTCTCATTGTAATGTGTATGTACTGCATTTGTGATTTTATGGTGATTTCATTTTACTGTCTTGTATGTGTGTATTCATTTCATTGCGTTCTATCTTTCAATAATTCAGTTAATCTATGTGATTTAATGTAATTTGGACTTTTGTGACTTAATTTAAATCTTAAGTATGAATTAAATCACAAGTTAATTATTTGGATTTTAGTGACGATTTTATGACTAATTTTTATGTGGTCTAAATGAGTAAGAAAAGTAATTACATGTGAAATTTTAAATCAGTACTTTTAGCTCTTATTAATTTTTACCGTAAAGAACTTCCAAACCTAAGTTATGTAAAGAATGATTGTTATGTATATATTTTATGTAAATTTTTGTATATAACATACAAATACCTCATAATAAAGTGAGAAAATTCATGCATGACTGAGGGACATTTATAAAATGTCATGTTTTTGTACAGGCATTATAATGCCACAGTATGAAATACACAAATATATTTGTAAAAAAAGAAAAAAAAAA

Protein: 3319 aa

>Dachsous_protein

MKGRRTKGVRVVAPCLPLPTVFCLLFTFLLLVKHGTCNNEEDFTQDFHVEEEQGQVIVGTIAAGQPWASQVPSPYFIVSSQNSGVTDHFEVNESDGIIRTKTSLDYEGQKQYTFVAISIFNSRNVRVNIYVEDINDHSPSFKNIEKVLPLLESAPRDAKYTLGSASDPDRDLNSTQRYEIVEGNINNAFRLETKPRQNDALLFDLAVNGELDFEEVPLYSLVIRAYDGGRPPLYGSMRLNITVIDVNDNQPIFNQSRYFAKLKENVTLGTPVLQVQATDRDSGENGKVRYQIDRQRSDQEQHFDINPVSGVISVNKQLDYEMKNVYELIVVARDNGTQRLQTTAVVSIQVEDVNDNEPVINIIFLTEDESPTISEDSEPGDFVARISVSDPDIDTYISHVNVTLQGGEGRFGLTTRDNVVYLVILAKPLDFEIRSYYTLTVVAIDSGIPPRIARKNFTIHVGDVNDNPPQFTQQSYEVEIQEVLSVGASVIQVTARDRDGGDNSKVFYSLVPSPDTQYFTISNTTGLVTTKSRVYCDTSSELSFQVRATDFGLPRLSSTANVVVKIKDANDNQPVFDQTFYNVSIPEDRAVGSCILKVSASDPDCGRNGEVTYSFARNLGFEVPLELRIDGASGEVCIDQALDYETKSSYEFPVMASDHDGLHTTAMIKVVVRDVNDNAPEFYPIQYNASLDEASSVGQEVVAVHARDADSGVNGFVNYRITSGNTQGYFSINSDTGVITVRRTLSLTQRLYTLTVESEDGSGRRSSNQAVIRISILDASQQPPTFSTSLYQFRVAEGRPTNSLVNRVYATNSNPAAGFIRYRIVAGNADGLFKIESRSGEIKTQAVLDHESVPYSLLTVQAESGNPVSYSQAQVNISIDDINDNSPVFPVRMLEIAVQENAAVNSVVYSVLATDQDSGMNGKVQYSLQDSSGVFKIDSATGQLKIARSPDYESQSSYRMSVTASDQGAPPQQATMQLIVNIQDVNDNSPVFSKSQYNFKLAEGAPVNTVIGKLNATDADSGNNGKISYTLQNGPDSDMFGISSDSGNLFNKVVLDREDKEERVLTAVVVDSGIPARSSSVVVILTVLDENDNHPRFLEDAYVFKVNENLPSGADIGVVQATDPDFTDNQRLVYSLLLPSDQFQINENTGAITTKQALDREVGDKYQLTVKVSDVGFPTATAIVNIEIQVLDVNDNSPVFDQSGTYVVYVEENQPKGHIVTTVTAHDADEGKNAEISYYLREDSQENYKLFKIHPRTGLLTTQEVLDHEERSLYTLQVYAMDGGAPARTAMTTVEINVQDINEGGPEFDAPTVTFNIKENSAPGTVVGVIKARDQDSGDNGVIHYYIIGGNLFSMFGINQTTGSLYLEQSPDYEVASSYSVIVKAVDNSITNPLSNDIKVLVNVIDVNDNAPVFDTDPVVVTFSENEPVGSTVYVFNAFDVDGGNAGLVHYSIVSQSPESQWFSIGRTDGKLVSSKVIDYEETSQISLVIEARDQPENKRDSLSRTVTAVIMIKDLNDNAPKFIQERTEFNILEDEPLGYPVMHLVARDDDSGQNGKVTYSIESGNNGKYFSLNPETGLLAIAKGLDREAVESVRLQIIARDHGYPQLSSEVYITANIIDVNDNKPKFTQKVYNASIMENQPVGTFVTAVSAVDPDQGANGKIVYLIPFGMADYHFTLDEKTGRIMSNATLDREVVDKWTLTAYVKDSGYPSLYDTATVVIDVLDENDHSPEFRDPVYRVEVAENEKMKVVHTVIATDKDSGENGKVSYSIIGGNTDGKFSLDSSTGILSCKPLDRETQSSYNLTIVGRDGGRPSRSATCTVLIEVMDENDNDPVFNKPLYSKSIRESIRINTSILRVSATDDDVGNNALITYSFDNDADGLFKIDNQTGVIRTASEFDRETKSSYSFQVFASDGGMYGPRSRSVQVDISVEDVNDNAPVFEQVPYRVNVSENHGVGQYVLQVSAKDKDQGSNGEVMYNFALESPYFDMDSFSGVIVTRQSLDSAAVRVHRLEIIARDKGQNPMSSTGLVEIRVGNAAGYATLIFDRDEYSADVQENAAVNTPVIRVRARHPNTPTAAISYTFASGNEANIFSIDRISGQVTVRNSSTLDYESLKQLRLVISATASAAYGYATVFVNLLDMNDNPPHFTQDRYVSSVWEGSPRGTFVTQIRAMDADEGRNARVTYSIIGGNVQNAFVIDPPETGIIKANIILDREIRDSYRLEIEAIDGGSPPLSTHCVLRIQIIDVNDNAPFFPQYSPISIPEDAQPGREILQVTANDRDMNPTLSYHLVPDRSYENKFNIDRFSGRVSITKPLDYEEMQQYDLRIMASDNEYNATTSVKLNIVDVNDNPPVFSQQSYLATVAEMTPPNSPIIQVNATDEDAGQNAAVTFSMLPMNGFYIDEISGIIYTDKTLTFSRSQPITHLVVTAKDAGTPSQTSVTAIQLQVVDVNNNAPVFSNTVYRVSVPEDERKGHVLISLTATDEDDAVDNKKVDYSIVSGNDNGLFDISSSTGDVMLVGSLDRETSPDFDLQIMAKDRGDPPLSSTCKVLITVDDINDNSPVFSQKYYQTTVPEDLEEGTDIITITAVDMDSGENSRVTYIIESGNDDNKFEIDRDSGLMKVRLPLDYETRKLHRLIIKASDSSKTHQRSAFTTVTINITDVNEFDPDFPVFMYMETVLENEPPGAYVFTAHANDKDAGNYGQVSYELISDLPRFAIDQLTGVVTTKEMFDYESSQETYSFYIRATDLGGRSTTIPVMVSVSSVDEFKPRFGKARYQFEIPGNAEKGFIVGQVQAYDDDQGRSGEIRYSFSEEHEYFHVVPDTGVIMVIKDLQDNGAKREKRHTFFLSRQKRALEGNNITLIIVASSWLSNSLVDRTKVELAINRTCVGCAYLGQVATGDLSGTPLVLVSLFVIVVVVLVVIVLIVYLRSRERKRRLPNSQYDRSFDSMNMPAALPRDLAPPTYNEIHQNHYNRSHDHNITTSEISDQSHSASSGRGSAEEADDVDEEIRMINAAPMQTKGLRIAQDSGIQQDDDAVSEHSVQNHQEYLARLGIDTSKLKSIPQQQPAKPPVGSSVESMHQFAEEGGGEGPPPKVPAVNAIKTRESSRDPDGTHEFQYTEPSNNTAASLSSVINSEEEISGSYNWDYLLDWGPQYQPLAHVFSEIAKLKDDSIKPKNKSQLVPHKQNLNALNQNKSYPPPLLTSAPPKVLLAQPVSTRPSNNSSSGGSSRTSQMVSLPSLPRSPISHESSFTSPALTPSFTPSLSPLATRSPSISPIITSKDGSTNHLNHHHRRPPVVIENRPVVNKQASSESEQEIHI

Domains

(27x) Cadherin_repeat: 75 – 137, 150 – 249, 258 – 356, 372 – 467, 475 – 572, 581 – 678, 686 – 779, 791 – 885, 895 – 988, 996 – 1093, 1102 – 1196, 1205 – 1303, 1314 – 1410, 1418 – 1519, 1529 – 1624, 1632 – 1729, 1737 – 1832, 1841 – 1937, 1945 – 2034, 2049 – 2144, 2153 – 2250, 2260 – 2350, 2358 – 2453, 2461 – 2559, 2568 – 2664, 2674 – 2766, 2776 – 2881

Cadherin_C: 3102 - 3159

Clone

Partial ORF

Primers

Forward: CAACCAGCCCATATTCAACC

Reverse: GCTCGAACTTGGAAGGACAG

Nucleotide# 1018 – 1917 = 900 nucleotides

>Dachsous_clone

CAACCAGCCCATATTCAACCAGAGTAGATATTTTGCTAAATTAAAAGAGAACGTAACGTTGGGGACCCCTGTCCTCCAGGTCCAAGCGACGGACAGAGATTCCGGAGAGAACGGAAAAGTGCGTTATCAGATCGACCGCCAAAGAAGCGACCAAGAACAGCATTTCGACATCAACCCGGTCAGCGGTGTCATCAGCGTCAACAAACAATTAGATTATGAGATGAAGAATGTTTATGAATTAATAGTTGTCGCTAGAGATAATGGGACCCAAAGGTTACAAACTACTGCTGTTGTTTCTATACAAGTTGAAGATGTGAATGACAATGAACCCGTTATAAACATCATCTTTCTAACGGAAGATGAGTCGCCTACTATATCTGAAGATTCGGAACCAGGCGATTTTGTCGCAAGGATATCTGTGAGCGACCCTGATATAGATACATATATTTCTCATGTGAATGTCACTTTACAGGGAGGAGAAGGAAGGTTCGGTTTGACTACCAGAGACAATGTTGTGTATTTGGTTATTTTAGCGAAACCTTTAGATTTTGAGATACGTTCATATTACACTTTAACCGTAGTTGCCATTGATTCTGGAATACCACCAAGGATCGCTCGCAAGAATTTTACGATTCATGTGGGAGACGTAAACGACAACCCACCGCAATTTACTCAACAGTCTTATGAGGTGGAAATCCAGGAAGTCCTTTCAGTGGGCGCTTCCGTCATTCAAGTCACGGCGAGGGACCGTGACGGAGGGGACAATTCAAAAGTGTTTTACTCCCTCGTCCCCTCTCCGGATACCCAGTATTTTACCATCAGTAATACCACCGGTCTTGTGACCACTAAATCCAGAGTGTACTGTGATACGTCTTCGGAACTGTCCTTCCAAGTTCGAGC

Cloning info

PCR rxn on 07/28/16 using GoTaq polymerase mix

| Template | Initial denature | Denature | Anneal | Extension | #cycles | Final extension |
| --- | --- | --- | --- | --- | --- | --- |
| 2 hr cDNA | 4min @ 94C | 30sec @ 94C | 30sec @ 55C | 2min 15 sec @ 72C | 35 | 10min @ 72C |

**Celsr1**

Gene model:8158 nucleotides

ORF: 235 – 8157 = nucleotides

>Celsr1_partial

GGAAGGGGCAGTCTCAGTGCGCTGCGTTTTGAACGTCAGTTCAAGCGTCGACGTGCTGGAGGACGCTGTAATTCATGTGGAACACACAACTTTAAACACAGCACTATGGATTTACCGGGCTCATAGCACCATGTGAATGTTACCAGAGTATCACACACTGTCTTAGGACTTGTTAGAAGACAGTAGATGAGTGGACAATACTTAAAACTGGAGTTTACCTGACGATTTCTCAACATGGAGCTGAGGGGGAGGCCGTGTTTTTACACCACAGCGTTGCGTCTTTTGGTGACATCGCTGTGTCTGATATTTTCACGGTCTCTTGCGCTGGATCTGCACTTTAACAGCCTTCCAAAAGGAACGATATTGTTCGATGCAAGCTTACAAGGTGATTGGAAGTACCATTTGGATAAGTTGAAAAGTTCCTTCAGTGTTTTGAGACATGTTGATTTGACTGCCGATGGCAAGGTGGTGATTGCCTTGCCCATTGACTGCAGAGAGTTGAAAACAAACCCCTTTAAGTTGAGTGTGTCAGCTCACACAGATTCAATTAGTCCATCCAATTTTACGGTGATTCCTCTCTCAGTTCATCTGCATGGCCAGGGTTGTTTATTTGATCACAAAGCCATGGAAATGCCATCTTCCATGACTTTTGATGCCTTTCAACAAAAAATATTGCTTCACACATGGCTAAACCAGGACAGTTGTTTAACAAGTGAAGAACCCATAGCCAAACTATCTGATTTCATTCCTAGAGGCTTGGATTCATGTAAATGCTCTCATAGGATAGACAGTGATGATGTGTTCTATGTAGGAGAAGAGAATGAATTGAGGACTTCACAGTTTGTGTGTTTTGAAAGAAGCCAAATGCATATCACAGGAAATGTGTCAGTGTCCTGTATTTCAAGGAAAACCATCCTTTACCCATTTTCATTGACTTTGATGTTCAAGAAATCCACCCTACCTGAGTTACACCTGTATCAGAAAGCTCACAGGAGACACAAACGTCAGACTGGAAGTTTCATGCCCCCAGATTTCCCCCAAAATCTGTATGTTGCCCATATTAATGAAGAACAGGGAGCTGGGGTGGTTGTTGGGACTTATGCCATCATTGACAAAAACAGGGATGTTTCCTATTCCTTGGTAGCATCCAGAGATGGCAGAAGTCAAAGTCTGTTCACCATTGATGCGACAAGTGGGCAAGTCACGACGATTGATAATTTAGACCGAGAAGGAATAACTGTCCATTATTTTGTGATTTACGCTACGGATGCAGTGAACACCCGAGTTAACGGACAGACCGCCCTCACAATTTATGTAGATGATGTAAACGACCATGCTCCTGAATTCGAAAGACAAAGTTATGCCAGAGAAGTATCTGAGTCCGTCTTAGTTGGAGCAACCATATTAACAGTCCGAGCAAGTGATGGAGATACCGGGCCAAACGCTGATATCGAATATTCTATCGTTAATCCTTCGGGTGACAATGAGGTGTTTAGGATTGATTCACGGACAGGTAGCATCATGACAAGAGCAACTTTAGACAGAGAGAAGACCCAGTTTTACACCTTAGAAATCCAAGCTGCCGACAAAGGTCCTGTTACGAACCGTCAAAAAGCAAATGCGATCGTGGAAATTACTGTTACAGATGAAAATGACAACTATCCTCAATTCGAAAAGAGTTCGTACACTGTAGATGTGAGCGAAGACCTCGATCCAAGTGGGCGACCTCTAGTAGCCGTCATCAAAGCAACTGATGGAGATGCTGGCGACAACTCGGCCATCCGCTACAGCATCACTGGAGGCAACTCACAAAATGCTTTCAATATTGACACCATATCTGGGCAACTATCTTTGCTGATTCCTCTAGATTATGAGCTAACCAGTAGCTACAGGTTGAGAATACGGGCCCAAGATAGCGGCTCTCCTCCGAAGAGCAACAGCACTACTGTATTAGTCCGAGTGATTGATGTCAACGATAACGAACCTCGGTTTCTAACCCATATCTTCCAAGAAGCGGTGTTAGAAAATGTCCCAACTGGATTCACTGTGATGAGAGTGCAAGCTTATGATGCTGACTCCGGCAGCAATGCAGCTTTGCTTTACAGCATCATCAACCCTCCATCTGGGCTACCTGTGAGTCTGGACAGACACACAGGCATAATAACCACCAACCAGTTGCTAGATAGAGAAATACAGAAGAGATATAGCTTCCAAGTTGAGGCGAGAGATCAAGGAAGGCCTCCCAGGACTGCTACGACGTCTGTTGAAATTAATATCAGGGATGTTAACGATAATGCTCCAACTTTCAACCCGAGAGTGTATTACGAGACAGTTTCGGAAGAAGATAGTCCTGGTACTCATGTATTAACCGTCACAGCCAATGATGCCGATGCAAATGAAAATGCCCGAGTGACCTACACCATAGCATCTGGAAACACCCGGGGTGCCTTTAGTATCATGAGTCAAATGGGACAAGGTTCCTTGACTGTTGCGAGGACTTTGAATTATAAAGAACAGAGCCATTATATTCTTACCATCACTGCTTCTGATCCTGGGAATTTGGTTGATACTGCAACAGTTTTCATCAATGTCACTGATGCCAATACTTACAGGCCTGCTTTCCAAGGCACTCCGTACAAAATAAGAGTAGCCGAAGATGCTGAGATCGGTACGAGCGTCTACAAAGTTTTAGCGACTGATGGAGATGTCGGCGAAAATGCCCGAGTTACGTACACTATGGATGAGAATGAAATTTTCGGCATGAATCCCTCAACTGGAGTGATTTCGGTTACAGGAGAGTTAGACCGAGAAAAGACGGCTGGTTACGCCATTAGTGTCACAGCTACTGATCACGGTAGACCACCAAAATCCGACACGACTGATGTTGAGATTATTGTCAGTGACATCAATGACAATGATCCCAAATTCTCTGAAGCAGTTTACAATGGAAGGGCCTCAGAGGATGCAATTGTTGGTTCTAGCATCTTGACAATCATTGCGACTGATGAAGATGAAGGTTTGAACGGAAGAATAAGGTACACTTTCGAAGGAGGTAACAACGGAAATGGCGATTTTGTCCTTGAGCCGACTTTGGGAATCTTACGAGTTTCTAAACCTTTGGACAGAGAGAGAACTTCCATGTATGAACTGAAAGCTTATGCCATAGACAGAGGAGTCCCAGAGAGATCTACATCAGTTTTGATCAACATCTACATCGATGACATCAACGACAATTCTCCTCAATTCGGATCGAAGGAGATCCATTTTGATATTTTGGAGAACAGTCCGATTGGATCTACGGTGGACAGTTTGGTTGCTGAAGATCCGGATGAAGGAGTAAACGCTGAAGTGGATTACAGTATCGTGGGTGGAGTGGATGCGGATTCGTTTGCTCTTACGACTAGACCGAACGAACCGGCTGTCCTCACGACACTCGTGGATCTGGATTACGAAAGTGGCAAGACGGAATACAAGGTGATGGTCCGGGCGAGGTCATTCCATCTATTCGAAGATGTCACTGCTGTCATTCATGTGGTTGACGTCAATGACAACATGCCTCAACTGTCAGATTTCACCATCATATTCAACAATTTCCGGAATCATTTCCCTACCGGGTACATCGGACGCATTCCGGCCATGGATCCGGATGTCAATGACCGTCTGTCGTACCGGTTTATATCTGGAAATGATGCGAACATTCTTCAACTTAACCCGACTACTGGGTTTATTCAGTTGGATTCTCGACTCAATTCGGATGTACCCACTAATGCCAGTCTGCAAGTCAGCGTTTCAGATGGCATCAATGAAGTGAAGGCAAGGTGCAAGTTGTATGTGAGATTGGTCACCCAAGAAATGCTATTCAACAGCATCACAATAAGGCTCCAAGACATATCCCAAGAAGCCTTCCTCTCCCCATTATATCAGAGATTCACCTCAGCTCTTGCTTCGATCATACCAACTTCTGAAGACAATGTTTTCCTCATCAATGTCCAGGATGACACTGATGTGACAGAACCCATACTCAATGTCAGCTTCAGTGTCAGGAAGACAATTCACAACAACAGGGATGTATTTTACAGTCAACAATTCTTGAGGGAAAGGGTTTACCTACAAAGGATTTTACTGGCCAAGCTGTCTACCTTACAGGTTCTCCCGTTTGATGACAACCTGTGCCTGAGAGAACCCTGCCTCAACTATGAAGACTGTGTGTCCATCCTCCAGTTTGGAAATGCATCCTCAGTCGTCTCCTCTCCAACGATGCTCTTCAGACCTATTCATCCGATTAATGCTGTGTCTTGCAAGTGTCCAAGTGGATTTACTGGCCGAGACGCTGTGTACCTGTGTGACATTGAGGTGAACCTGTGCTACTCTTCTCCATGTCAGAATAATGGATCATGTATTCAGAGGGAAGGAGGCTACACGTGCAAATGCGGCGACGGCTTCACTGGAGACCATTGTGAGATCGACATGAAGCACAAGAAGATCACTCACTGCGACTTCGACGTCTGCAAAGGAGGATCACGCTGCGCTCCCTTGATCAGAGGAGGGTTCCGTTGCGAGAAATGTCCTCCTCTTTCTCAGAACAGTCTCCAGGATGAGTTCTGCATGGACTGCAAGGATTTGGAATACCACACACAATTCTGCGGATTGACAACCAGAAGTTTTGAGAAAGGATCCTTCTTGATGTTTCCTTCGTTGAAGAGGCGACATCGGTTTACCATCTCCCTTGAGTTTGCCACCACAGATCGCAATGGCCTCCTCTTCTACAATGGCAGATACAATGAGAGACATGACTTCATCGCTCTGGAGATTGTCGATGGCCAATTGCAGTTTTCCTTCTCCCTTGGAACGGATGTGTCGAGAGTTAGCGCAAGCATCTCTGGAGGTGTAGCTGACGGAGACTGGCATACTGTTACCATTGATTACTTCAATAGGACTGCAAGCTTGACAGTTGGCGAATACTGTGACACTGAGCTGGCCGTTCAGCATGGACGTCAAATTGGAAATTACTCCTGTGCTGCTAGGTTCCACAAGGTCTTGGAAGACAGGTGTCACAAGATGACAGAGCCATGTCACAGATTCTTAGATCTGACAGGGCCTTTGCAAATTGGTGGTCTGCCGAGCTTACCAACAGAATTCCAAGTGCGCCATACGTCGTACCAAGGTTGCATTCGTAACATCAACATCGACCAAAAGTTCCTCGATTTGAATGATTTTGTGTACAACAATGGCACCGTCCAGGGATGCAGAAGTAAGAAGTCCTACTGCGCTGCTGAAACTTGCAAACACGGAGGGATCTGTCTTGAAGCCTGGGGCACTTACCAGTGTGAATGTACTCCCGAATGGGGTGGAAAGGATTGTAGTCAAGTTATTGAACCACCAAGGAGACTAAAAGGAAGCAGCTACTTGCTCTACACCAAGGGATTTCAAACAATTCAGCTGCCCTGGTACAGTGGAATTGGATTCAGGACTCGGCAGTCATCTGGCCTGCTCATGTCTGTGGTTCTGAGCAATGGTGATTCAGTGAACACTGAGATTAAGAATGGTGCTATTAACTTAGTACACCCTCAGAAGACTGTGGTTCTGGATTACACTTTTGTGAATGACGGAAAATGGCATTTTCTGGAAATGAGATGGCGTCGAGGAAAGTTGATTCTCTTGTTGGATTATGGACAATCTCAGGTTGAAGTTGACATGGGAAGCTGGGTGAATGGACAATCCATCTCTCGTGTCTATGTTGGAGGACTGGCGGAGCAAGTCTCAGGGACGCGTGTTGTGTTGAAGGGACTAGTCGGCTGCTTCAAGGATGTGAGAGTTGGAAGCAGTCCCAATTCCATTCTCAACGAACCCCAAGAATACAATGTGGAGCCAGGATGCCCTGTGTCTGACCCTTGCAGTAGCTCACCGTGTCCAGAAAATTCCTATTGCATCAACCAGTGGGAAAAACACATCTGTGCTTGCGAACCTGGATTCATTGGCCCTCATTGCATTAGTGTATGCAACAGTTACAACCCGTGTATGAATGGCGCAACGTGCCTCAACAGCACAGTGACCTCTAGTGGATACACCTGCGAATGTTCCGAAGGATTCCAGGGTCAATACTGCCAGAGTAAGATCGACCAGACTTGTGCTGCCACCTGGTGGGGTAGTCCTATTTGCGGTCCTTGCAACTGCCCCACTGACAGAGGATTCGACCCAGTCTGTAACAAACGCAATGGAGAGTGTTCTTGTCAGAAACACCACTTCAAACCAAACAACACAGACACCTGCTACCCATGCAACTGCTACTCTGTGGGTTCCAAAAGTTTAGCCTGTGACCCAGTCACCGGTCAGTGCCCCTGTGCAAGAGGAGTAATTGGGAGAATGTGTGACGCTTGCTCCAGTCCTTTTGCTGAAGTTACATCGAGAGGATGTGAAGTGTTGTATGACTCTTGTCCGAGATCGTTTGCGATGGGAATCTGGTGGGACCGCACAGACCACGGAGATAATGCCACCCAGGATTGCCCGAGGGGGTCAGTTGGTGATGCTGTGCGCCATTGTGAGACAGGAGGGTGGAAGGCCCCAGACCTCAGCAGGTGTCTAACTTCAGGATTAGTAGATGTGGAGAAACAGGTGAAGTACTTTGAAAGTGACACTATGCACGTCAACACCTTCTTATCAAAGGAGTTGATGGAGAAGATGGCGAAAGCTGCTACATCCGTAGAATCTCTGTACCCCCATGATGTCAACGTGATTCACCGTACCCTCCTCCAGATCTTAGAATACGAATCTCAACAAGCTGGTCTCAATCTCACTCATGCCCAAGACAGAGATTTCATCCAGAATCTAGTGAACACCATTGGAAGAACTTTGAATCCTCTTCATTTGTCTGGTGCTTTGAGAGATCAGAAAATTCCCGAGGTCATCGAAGCATTAGAAAGATACATGACGACCTTAGCAGCTAATATGAGGACCGTGTTCACCAACCCCTTTGATTCTGTCAGTGATAATATCATTGTAGGATTGGACGATGTGGTAAAACAGAATCTCTCTACTTTCAACATTCCAAAATTCAACAACAAAGTGCCCAACCCTGATATGTTTGACAAAGACACCAAGGCACGCTTGTCAAAGACTGTCCTTTACCCAAACTCTATGGCGGACATGGTTCAGAATATCATTGGTCGTAACAAGCCTTCTGCTTTCATCGGATACATCATTCATAAGGACCTTGGCTTGCTGCTACCTCATCGATATGCACCCACTCTGAGATATGACACCACTATACCTTTAGAAGTAAATGCCCCAATATTTACGTTGAACATTGTGGATGGCAACAAGACAATATCAGGACCAACCAACGCTCCCATCATCATTGATTTCAAGATCTTCAACAGTGTCAACTATTCCAATCCTCAGTGTGTTTACTGGGACTTTGATAATGGACAATGGTCTCCGGATGGTTGTAAACTTGTTGCCGTTCAACATTACACAGAAAAGGATATTGTCAGCTGTGAATGCAACCATCTGTCAACCTTTACAGTCGTCATGGACAGATTTGATCTTGCCAAAGCCAGTGCTTATGCCGTTACCATCCAAGCTGTGACTTATGTTGCCGTGGCGATTGGTCTGTTGCTATTGTTTGTTGCCATGGTGCTGTTCTGTTGCCTCCGAAACCTGCATTCCAACTCTAATTCCATCCACATCAATCTCGTGTTCAGCATCATCGCTGCTTCTCTCATCTACATCACTGGCATCAACCAAACACAGCCAAAGGTGCTCTGCAAAGTTGTGGCCATTGTTCTTCATTATTTCTTCATGTGCACTTTCGCCTGGATGTTCGTGGAAACCCTTCACTTGTACAGAATGCTGACAGAAATAAGAAACATCAACCACGGAGCTATGAAGTTCTATTACGTCATTGGATATGTAATACCTGGGATAATTGTTGGACTGGCAGTGGGTCTAAGCACAGATGGATATGGCAACATGCGATTCTGCTGGCTACAAACTTCTGATCTAATCATTTGGAGTTTGGCAGGACCCATCATTTGCATCATTGCACTCAACATCATTGTCTTCTTCATGGCTGTTGCAGCCAGCTGTCGTGGCAAAGCAGCTGATCCTGAGTTCTCCAGGCTCAAGTATGGTCTGAAAGCAGCCTTGGTGCTCCTCCCCC

Protein: 2641 aa

>Celsr1_protein

MELRGRPCFYTTALRLLVTSLCLIFSRSLALDLHFNSLPKGTILFDASLQGDWKYHLDKLKSSFSVLRHVDLTADGKVVIALPIDCRELKTNPFKLSVSAHTDSISPSNFTVIPLSVHLHGQGCLFDHKAMEMPSSMTFDAFQQKILLHTWLNQDSCLTSEEPIAKLSDFIPRGLDSCKCSHRIDSDDVFYVGEENELRTSQFVCFERSQMHITGNVSVSCISRKTILYPFSLTLMFKKSTLPELHLYQKAHRRHKRQTGSFMPPDFPQNLYVAHINEEQGAGVVVGTYAIIDKNRDVSYSLVASRDGRSQSLFTIDATSGQVTTIDNLDREGITVHYFVIYATDAVNTRVNGQTALTIYVDDVNDHAPEFERQSYAREVSESVLVGATILTVRASDGDTGPNADIEYSIVNPSGDNEVFRIDSRTGSIMTRATLDREKTQFYTLEIQAADKGPVTNRQKANAIVEITVTDENDNYPQFEKSSYTVDVSEDLDPSGRPLVAVIKATDGDAGDNSAIRYSITGGNSQNAFNIDTISGQLSLLIPLDYELTSSYRLRIRAQDSGSPPKSNSTTVLVRVIDVNDNEPRFLTHIFQEAVLENVPTGFTVMRVQAYDADSGSNAALLYSIINPPSGLPVSLDRHTGIITTNQLLDREIQKRYSFQVEARDQGRPPRTATTSVEINIRDVNDNAPTFNPRVYYETVSEEDSPGTHVLTVTANDADANENARVTYTIASGNTRGAFSIMSQMGQGSLTVARTLNYKEQSHYILTITASDPGNLVDTATVFINVTDANTYRPAFQGTPYKIRVAEDAEIGTSVYKVLATDGDVGENARVTYTMDENEIFGMNPSTGVISVTGELDREKTAGYAISVTATDHGRPPKSDTTDVEIIVSDINDNDPKFSEAVYNGRASEDAIVGSSILTIIATDEDEGLNGRIRYTFEGGNNGNGDFVLEPTLGILRVSKPLDRERTSMYELKAYAIDRGVPERSTSVLINIYIDDINDNSPQFGSKEIHFDILENSPIGSTVDSLVAEDPDEGVNAEVDYSIVGGVDADSFALTTRPNEPAVLTTLVDLDYESGKTEYKVMVRARSFHLFEDVTAVIHVVDVNDNMPQLSDFTIIFNNFRNHFPTGYIGRIPAMDPDVNDRLSYRFISGNDANILQLNPTTGFIQLDSRLNSDVPTNASLQVSVSDGINEVKARCKLYVRLVTQEMLFNSITIRLQDISQEAFLSPLYQRFTSALASIIPTSEDNVFLINVQDDTDVTEPILNVSFSVRKTIHNNRDVFYSQQFLRERVYLQRILLAKLSTLQVLPFDDNLCLREPCLNYEDCVSILQFGNASSVVSSPTMLFRPIHPINAVSCKCPSGFTGRDAVYLCDIEVNLCYSSPCQNNGSCIQREGGYTCKCGDGFTGDHCEIDMKHKKITHCDFDVCKGGSRCAPLIRGGFRCEKCPPLSQNSLQDEFCMDCKDLEYHTQFCGLTTRSFEKGSFLMFPSLKRRHRFTISLEFATTDRNGLLFYNGRYNERHDFIALEIVDGQLQFSFSLGTDVSRVSASISGGVADGDWHTVTIDYFNRTASLTVGEYCDTELAVQHGRQIGNYSCAARFHKVLEDRCHKMTEPCHRFLDLTGPLQIGGLPSLPTEFQVRHTSYQGCIRNINIDQKFLDLNDFVYNNGTVQGCRSKKSYCAAETCKHGGICLEAWGTYQCECTPEWGGKDCSQVIEPPRRLKGSSYLLYTKGFQTIQLPWYSGIGFRTRQSSGLLMSVVLSNGDSVNTEIKNGAINLVHPQKTVVLDYTFVNDGKWHFLEMRWRRGKLILLLDYGQSQVEVDMGSWVNGQSISRVYVGGLAEQVSGTRVVLKGLVGCFKDVRVGSSPNSILNEPQEYNVEPGCPVSDPCSSSPCPENSYCINQWEKHICACEPGFIGPHCISVCNSYNPCMNGATCLNSTVTSSGYTCECSEGFQGQYCQSKIDQTCAATWWGSPICGPCNCPTDRGFDPVCNKRNGECSCQKHHFKPNNTDTCYPCNCYSVGSKSLACDPVTGQCPCARGVIGRMCDACSSPFAEVTSRGCEVLYDSCPRSFAMGIWWDRTDHGDNATQDCPRGSVGDAVRHCETGGWKAPDLSRCLTSGLVDVEKQVKYFESDTMHVNTFLSKELMEKMAKAATSVESLYPHDVNVIHRTLLQILEYESQQAGLNLTHAQDRDFIQNLVNTIGRTLNPLHLSGALRDQKIPEVIEALERYMTTLAANMRTVFTNPFDSVSDNIIVGLDDVVKQNLSTFNIPKFNNKVPNPDMFDKDTKARLSKTVLYPNSMADMVQNIIGRNKPSAFIGYIIHKDLGLLLPHRYAPTLRYDTTIPLEVNAPIFTLNIVDGNKTISGPTNAPIIIDFKIFNSVNYSNPQCVYWDFDNGQWSPDGCKLVAVQHYTEKDIVSCECNHLSTFTVVMDRFDLAKASAYAVTIQAVTYVAVAIGLLLLFVAMVLFCCLRNLHSNSNSIHINLVFSIIAASLIYITGINQTQPKVLCKVVAIVLHYFFMCTFAWMFVETLHLYRMLTEIRNINHGAMKFYYVIGYVIPGIIVGLAVGLSTDGYGNMRFCWLQTSDLIIWSLAGPIICIIALNIIVFFMAVAASCRGKAADPEFSRLKYGLKAALVLLP

Domains

(9x) Cadherin_repeat: 272 – 367, 375 – 475, 483 – 582, 595 – 687, 695 – 790, 800 – 894, 902 – 1000, 1008 – 1106, 1125 – 1203

EGF_CA: 1374 – 1409

LamG: 1474 – 1652

EGF_CA: 1683 – 1709

LamG: 1744 – 1862

EGF: 1885 – 1919, 1922 – 1958

EGF_Lam: 2014 – 2052

HormR: 2064 – 2115

GAIN: 2142 – 2354

GPS: 2385 – 2433

7tmB2_CELSR_Adhesion_IV: 2445 - 2641

Clone

Partial ORF

Primers

Forward: ATGGCGATTTTGTCCTTGAG

Reverse: GAAGAGCATCGTTGGAGAGG

Nucleotide# 3065 – 4266 = 1202 nucleotides

>Celsr1_clone

ATGGCGATTTTGTCCTTGAGCCGACTTTGGGAATCTTACGAGTTTCTAAACCTTTGGACAGAGAGAGAACTTCCATGTATGAACTGAAAGCTTATGCCATAGACAGAGGAGTCCCAGAGAGATCTACATCAGTTTTGATCAACATCTACATCGATGACATCAACGACAATTCTCCTCAATTCGGATCGAAGGAGATCCATTTTGATATTTTGGAGAACAGTCCGATTGGATCTACGGTGGACAGTTTGGTTGCTGAAGATCCGGATGAAGGAGTAAACGCTGAAGTGGATTACAGTATCGTGGGTGGAGTGGATGCGGATTCGTTTGCTCTTACGACTAGACCGAACGAACCGGCTGTCCTCACGACACTCGTGGATCTGGATTACGAAAGTGGCAAGACGGAATACAAGGTGATGGTCCGGGCGAGGTCATTCCATCTATTCGAAGATGTCACTGCTGTCATTCATGTGGTTGACGTCAATGACAACATGCCTCAACTGTCAGATTTCACCATCATATTCAACAATTTCCGGAATCATTTCCCTACCGGGTACATCGGACGCATTCCGGCCATGGATCCGGATGTCAATGACCGTCTGTCGTACCGGTTTATATCTGGAAATGATGCGAACATTCTTCAACTTAACCCGACTACTGGGTTTATTCAGTTGGATTCTCGACTCAATTCGGATGTACCCACTAATGCCAGTCTGCAAGTCAGCGTTTCAGATGGCATCAATGAAGTGAAGGCAAGGTGCAAGTTGTATGTGAGATTGGTCACCCAAGAAATGCTATTCAACAGCATCACAATAAGGCTCCAAGACATATCCCAAGAAGCCTTCCTCTCCCCATTATATCAGAGATTCACCTCAGCTCTTGCTTCGATCATACCAACTTCTGAAGACAATGTTTTCCTCATCAATGTCCAGGATGACACTGATGTGACAGAACCCATACTCAATGTCAGCTTCAGTGTCAGGAAGACAATTCACAACAACAGGGATGTATTTTACAGTCAACAATTCTTGAGGGAAAGGGTTTACCTACAAAGGATTTTACTGGCCAAGCTGTCTACCTTACAGGTTCTCCCGTTTGATGACAACCTGTGCCTGAGAGAACCCTGCCTCAACTATGAAGACTGTGTGTCCATCCTCCAGTTTGGAAATGCATCCTCAGTCGTCTCCTCTCCAACGATGCTCTTC

Cloning info

PCR rxn on 07/28/16 using GoTaq polymerase mix

| Template | Initial denature | Denature | Anneal | Extension | #cycles | Final extension |
| --- | --- | --- | --- | --- | --- | --- |
| 2 hr cDNA | 4min @ 94C | 30sec @ 94C | 30sec @ 55C | 2min 15 sec @ 72C | 35 | 10min @ 72C |

**Four-Jointed**

Gene model: 2155 nucleotides

ORF: 499 – 1731 = 1233 nucleotides

>Four-Jointed_full

AAGGGAACGAATGGGGGGGAGAGCTAGGAAGGGGAGGGGGTACCAGGCACTGAGCCGTGGCGGCCGCCGACAGATATTACTGAGGGAGATCGGGGTGCAGTGTCATTTAATAAGTGTTCCGAGGCAGATCGAGTGAAGTGAATCACACAGGCAGATTTATGCAGTCAGTAGACACGGCTGGCCAATCCGTCTAGGAAACTACCTATGGGAAGAAGGTCCTAAGCAGCTTACGAGTCGAAATACAAAATATGAGAAAGGAGGCACTGCTAGCCAGACACTAGCTCCAACCAGCAGCAGCAGAGTGACACGACAAGACTCAAACAGGAGTCAACATTGCATGTGCCTGCCATGCTGCGCCGTATTGCAATTAGGATGGTAGTGGGAGTTTGACCACAAGTGACAGTCGCTGTTTGACAATTATGCAGACCACTGGGACATTGTGATACATAAGACAGTGCTCCACGGCACAAGACAGCACACAGCAAACTGATTCTAGTGATGGGAGACATGAGGATACCTCTGGTGCTGCTGACGGGCATGGCGTTTTGCCTCGGCTTGTTGCTGGGCCTGCTGTCTCACCTGCCGGGGGCCCAGTCTCCCCTCCAACGGACCGTTCTGCTCCGCCACAGCCGCGACCTCCGCCAGGCTGTGTCCCCGGAGCCCGTGCTGGGAAATGCCAGTGAAGAAAACCCAGCCTTGGAACAGATAGAACACATCCAACAGAGGATAGAACAGACTCAGGAGTCGATCAAAGCTCTGAAGGAAAACCACCACAGCGTCCACGTGCACAAGGAGCCGTCCACAGACATTTATGATGGAATTTACTGGAGCAACGTGATAACTGCTAACCCTGGTTTTACCGAGTCTCAAAGTCATTTATGGAGGAAACAGCTCCAAGAAGAGAGGGTGGTTGGCCTCCAGGAAGGCTGTGGGAGGATGCATAATCGCTTAGTAACTTTGGCTAACGGTAACCAGGCCTGTGCCAGGTACAGGATCAACAGGGATCAGATGCAGGGGGAGGTTTACTCTTATTATCTAGCAAGAGTCTTGGCTATTTATAATGTACCCCCTGTTGTGTTAGCTCTCCCTGACGCTAAAATGACTACCTGGAATTCTGTGGCGGAGCAGTTGACCAGTGCCCAGTGGAGCAGTGGCAGGGTAGTTGCCCTCACCCCCTGGTTAAAGGACCTTACCTCTGCTTACGTGCCTGTGGAGATGCAACCAGGAGGAAAGGGGCTTTACCCCAACCAAGATCTTGCCAAAAACAGAACTCTAGAGGAGTTAAGTGAAGTATCTCAGTGGTCGGACTTAATCATTCTGGATTACCTGACAGCCAATGTGGATCGAGTCGTCAATAACATGTTCAATCAGCAGTGGAATGATGACATGATGAGGAGTCCGGTGCACAACCTGGAGAAGACAGGGAATACTCTAGTGTTCCTGGACAATGAATCCGGATTATTCCACAGCTATAGACTTCTTGACAAGTACTGGCATTACCATGACACTCTCCTTAAATCCCTGTGCATATTTCGCAAGGAGACTGCTGACATTGTGAAAAGACTGTATGCCAGCAGAACTGTTGCTGAGGAGGTGGTCTCTCTGATGGAGAGGGAAGAGCCATTGAATGACAGAATTGCAAGGTTCAATGAAAGGACTATTAAGACCTTGCAATCAAGACTTGACGATGTTTATAAACAGATAATATCATGTGAATCCAAATACCATTGAATCTGTGATGTCATATTTTCTATATTTTCATTTTCAAGCTAGTCTGGTTTAACTTGAATGTAAATACATTTTCACACATGTTATATCTCCTAGTATGTCCCATACACTTATCTTGTTTCATACACAATTTGATGCACTTGAATTGCATTTAAAACCACATTTCACATTTTACCAAGCATGCAGCTAGCTGCACTAGTCTGTCAAATATAAACTGTCTCAATTTTGTTGAAGAGTGAATGTTATTGCCTTTTATGTAAATATAGTGAGGCTATGATGATTTTCAATTGTAAAGAGTGCTATATATATTTATTATTTAATAAACAAAGTTTATGATTGGAATTTTCGGTATTTGTGATATTTTGGCTGCTCAAACTTTGAAAAATACTTCTATTTCAAAATTTGATAGGGAACGAAACTCATGAGA

Protein: 410 aa

>Four-Jointed_protein
[truncated: 48,255 more chars]
