# Supplementary figures and images for: The asymmetric cell division machinery in the spiral-cleaving egg and embryo of the marine annelid Platynereis dumerilii
Source: BMC Dev Biol. 2017 Dec 11;17:16. doi: 10.1186/s12861-017-0158-9 (PMC5725810; doi:10.1186/s12861-017-0158-9)

# Negative control *in situ*

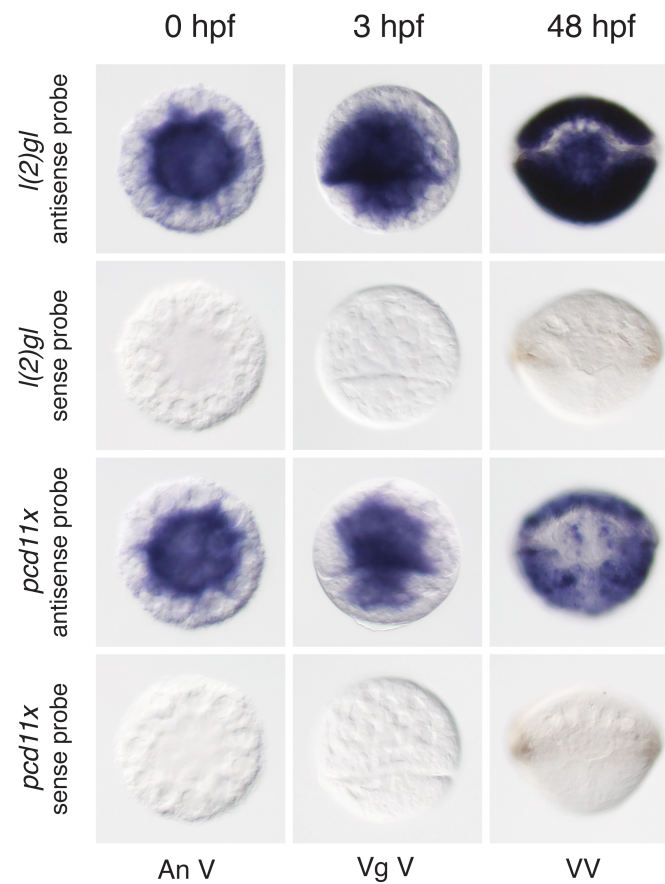

VV = Ventral View,  
An V = Animal Pole View, Vg V = Vegetal Pole

**Figure S2**

Supplement: Supplementary file 3 — Late stage WMISH for ACD genes shown in Fig. 8 as specificity controls at 2-day old P.dumerilii larvae. (PDF 6099 kb) [file 12861_2017_158_MOESM3_ESM.pdf]
